# A comprehensive analysis of teleost MHC class I sequences

**DOI:** 10.1186/s12862-015-0309-1

**Published:** 2015-03-06

**Authors:** Unni Grimholt, Kentaro Tsukamoto, Teruo Azuma, Jong Leong, Ben F Koop, Johannes M Dijkstra

**Affiliations:** Soeren Jaabaeksgate 10B, 0460 Oslo, Norway; Institute for Comprehensive Medical Science, Fujita Health University, Toyoake, Aichi 470-1192 Japan; Fisheries Technology Division, National Research Institute of Fisheries Engineering, 7620-7, Hasaki, Kamisu-shi, Ibaraki Japan; Centre for Biomedical Research, Department of Biology, University of Victoria, PO Box 3020 STN CSC, Victoria, Canada

**Keywords:** Teleosts, MHC class I, Evolution, Phylogeny

## Abstract

**Background:**

MHC class I (MHCI) molecules are the key presenters of peptides generated through the intracellular pathway to CD8-positive T-cells. In fish, MHCI genes were first identified in the early 1990′s, but we still know little about their functional relevance. The expansion and presumed sub-functionalization of cod MHCI and access to many published fish genome sequences provide us with the incentive to undertake a comprehensive study of deduced teleost fish MHCI molecules.

**Results:**

We expand the known MHCI lineages in teleosts to five with identification of a new lineage defined as P. The two lineages U and Z, which both include presumed peptide binding classical/typical molecules besides more derived molecules, are present in all teleosts analyzed. The U lineage displays two modes of evolution, most pronouncedly observed in classical-type alpha 1 domains; cod and stickleback have expanded on one of at least eight ancient alpha 1 domain lineages as opposed to many other teleosts that preserved a number of these ancient lineages. The Z lineage comes in a typical format present in all analyzed ray-finned fish species as well as lungfish. The typical Z format displays an unprecedented conservation of almost all 37 residues predicted to make up the peptide binding groove. However, also co-existing atypical Z sub-lineage molecules, which lost the presumed peptide binding motif, are found in some fish like carps and cavefish. The remaining three lineages, L, S and P, are not predicted to bind peptides and are lost in some species.

**Conclusions:**

Much like tetrapods, teleosts have polymorphic classical peptide binding MHCI molecules, a number of classical-similar non-classical MHCI molecules, and some members of more diverged MHCI lineages. Different from tetrapods, however, is that in some teleosts the classical MHCI polymorphism incorporates multiple ancient MHCI domain lineages. Also different from tetrapods is that teleosts have typical Z molecules, in which the residues that presumably form the peptide binding groove have been almost completely conserved for over 400 million years. The reasons for the uniquely teleost evolution modes of peptide binding MHCI molecules remain an enigma.

**Electronic supplementary material:**

The online version of this article (doi:10.1186/s12862-015-0309-1) contains supplementary material, which is available to authorized users.

## Background

The classical major histocompatibility complex class I (MHCI) molecules are key players in initiating an immune response against intracellular pathogens such as viruses. The mature classical MHCI molecule is divided into three alpha domains where the two most distal domains are involved in peptide binding and the membrane proximal domain provides stability and interacts with beta2-microglobulin. A major characteristic of these classical MHCI molecules is the immense polymorphism (differences between alleles) predominantly mapping to the two distal domains i.e. the alpha 1 and alpha 2 domains.

In classical MHCI molecules, these alpha 1 and alpha 2 domains provide a groove for binding of peptides where eight residue positions anchoring N- and C-terminal peptide ends are highly conserved throughout evolution, i.e.Y7, Y59, Y/R84, T143, K146, W147, Y159, and Y171 [1-3]. The residue Y84, found in mammalian and some reptilian classical-type class I molecules, replaced residue R84 which is common in birds, amphibians, sharks and bony fish. In contrast, many of the residues defining the pockets that accommodate the various peptide side-chains are highly variable thus enabling different MHCI alleles to present different sub-populations of peptides.

In humans, there are also a considerable number of non-polymorphic MHCI molecules that have various non-classical functions where most have retained the molecular characteristics of a membrane anchored molecule with three extracellular domains. Some of those also retained the ability to bind beta2-microglobulin and/or peptide ligands. Examples of non-classical human MHCI molecules are the HLA-E molecule that binds peptides derived from leader sequences of other MHCI molecules, CD1 molecules known to bind lipids, and MR1 that can present microbial vitamin B metabolites [4,5].

For teleost fish MHCI genes, our knowledge has grown rapidly since their first identification in the early 1990′s [6-8] and much is similar to what is found in mammals. The U lineage defined through phylogenetic analysis, consists of both classical highly polymorphic genes showing conservation of presumed peptide-termini anchoring residues, as well as non-classical genes with fewer classical-type anchoring residues and/or low variability. Classical type molecules have been shown associated with peptide and beta2-microglobulin [9], and were linked to allograft rejection [10] as well as resistance to pathogens [11]. There have also been a few intriguing discoveries. One of the surprises was the lack of linkage between classical MHCI and II gene loci in all teleosts studied so far, resulting in some authors using an “MH” nomenclature to emphasize the lack of structural continuity [12]. A second surprise was the finding that in some teleosts classical MHCI variability was considerably enhanced through retention of multiple ancient alpha 1 domain lineages, which are represented in distantly related species [13-16]. Although the exact mechanisms are still unclear, both allelic recombination as well as interlocus recombination events are likely contributors to classical teleost diversity [13].

A third surprise was the lack of MHC class II in Atlantic cod [17], although preliminary analyses had suggested the concept for quite some time [18]. The loss of the entire class II system in cod appears to be one extreme within a broad teleost MHC class II plasticity [19]. Malmstrøm et al. [20] suggested that cod MHCI molecules have sub-functionalized into two clades where one clade including some sequences with an endosomal sorting motif could have replaced the MHC class II function of exogenous antigen presentation. Although this model may be true, a reminiscent functional divide among MHCI molecules has also been described or suggested for other species. Typical endosomal sorting motifs are found in a number of classical as well as nonclassical MHCI molecules of mammals and teleost fish, and at least in mammals have been functionally associated with a number of differential intracellular transport and loading routes [21-25]. Even without obvious endosomal sorting motifs some MHCI molecules can be transported to endosomal compartments with help of the invariant chain [22], a molecule better known for transporting MHC class II. Thus, even from the distribution of typical endosomal targeting motifs, differences in MHCI transport routes between species can’t be predicted with certainty.

Previous studies have described four different MHCI lineages in teleosts i.e. Z, U, S and L, where sequences are classified into each of the four lineages based on phylogenetic analyses and lineage characteristic motifs. Only the U lineage includes genes with classical type polymorphism [6,8,26-28]. The U lineage also harbors non-classical MHCI genes with varying degree of conserved peptide-binding residues, low polymorphism and/ or transcription in restricted number of tissues [23,29]. In salmonids, medaka and zebrafish there is one major MHCI region with one or a few classical genes. Atlantic salmon and rainbow trout have one classical gene defined as *UBA* while medaka has two classical genes in this region defined as *UAA* and *UBA*. For zebrafish, haplotypes differ in gene copy number (one to three) and allelic polymorphism is harder to assign [25]. The classical U lineage genes in cyprinids, salmonids and medaka display profound polymorphism which in part has been generated through point mutations. However, ancient alpha 1 domain lineages shared between divergent species are shuffled between alleles through recombination and thus also add to the variation [13,14,16]. The alpha 3 domain tends to be more homogenized in a species-specific manner, possibly due to co-evolution with CD8 and beta2-microglobulin sequences, although some variation can be found in particular in the peptide connecting the alpha 3 and transmembrane domains [15].

While salmon, rainbow trout and medaka have around ten U lineage genes defined through phylogenetic clustering, other species show considerably more expansions of this lineage. Atlantic cod was reported to have 83 different expressed U lineage sequences in one individual, which translates to a minimum of 42 different genes assuming they are all polymorphic [30]. One wonders if this expansion could compensate for the complete loss of MHC class II genes. Similarly, although not as extreme, expansions have also been published in other species such as tilapia with 28 U lineage genes or gene fragments [31]. As tilapia has not lost its MHC class II function, we cannot explain the biological benefit from such an expansion [19].

For the other three lineages, information on phylogeny and genomic location is rather limited. The first MHCI sequence to be identified in teleosts, a genomic fragment from goldfish (*Carassius auratus*, GenBank accession AAA72345.1), belonged to the Z lineage [6], which was later substantiated as an expressed MHCI lineage [32]. Kruiswijk et al. [33] expanded on this in identifying a related, but distinct, new lineage in cyprinids which they defined as ZE. ZE-type have since been found in several teleosts [29,34,35], while the sequences described by Okamura et al. [32] are considered unique to carps. Since the publication by Lukacs et al. [29], nomenclature incorporates both types of sequences in the “Z lineage”, and newly identified ZE-type loci have been given a “Z” identifier (and not ZE) in their name (eg. [34]). Although most known Z lineage genes encode the typical peptide anchoring residues, these genes are considered non-classical due to low levels of polymorphism and more restricted tissue expression patterns [29,34]. Compared to the peptide anchoring residues of classical MHCI, the Z lineage molecules have an Y171F substitution, which in modified human classical molecules was found to reduce peptide affinity but still to allow peptide binding [36]. As noted by Nonaka et al. [13] and others the Z genes evolve differently from U lineage genes with higher sequence diversity in the alpha 3 domain and considerably better conserved alpha1 and alpha 2 domains. Remarkably, the teleost Z sequences were described to cluster with lungfish MHCI upon phylogenetic analysis [26,37].

The third MHCI lineage, defined as S, was initially identified in salmonids where the single locus was denoted *UAA* [27], but later renamed to *SAA* due to low sequence identity to U lineage genes [29]. S lineage fragments have also been found in catfish [26,29].

Salmonids in addition to some cyprinids [26] and some cichlids [38] also have genes belonging to the fourth MHCI lineage defined as L. Dijkstra et al. [26] found five L lineage genes in trout and one gene in Atlantic salmon, where most trout genes have a rather unusual gene organization lacking introns between the alpha 1, 2 and 3 domains. Both the S and the L lineages do not have the typical peptide N- and C-terminal anchoring residues which suggest that they bind non-peptide or no ligands [29].

Using available genome sequence databases, we here set out to take a closer look at the various MHCI lineages in teleosts. It became evident that we have still only scratched the surface of teleost MHCI. We found genes belonging to two of the lineages, Z and U, in all investigated species suggesting they cover essential core functions. The remaining lineages, L, S, and a new fifth lineage P, are absent in many teleost species which questions whether they provide essential functions.

## Results and discussion

**Figure 1:**
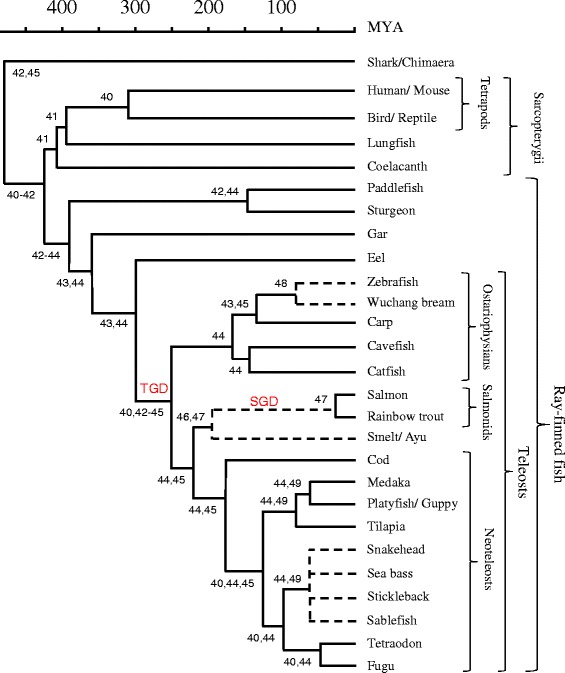
**Phylogeny of relevant species.** A timescale is depicted in millions of years ago (MYA). The tree is a gross summary of references listed below, with reference 44 given particular importance. In our study the relative phylogeny of the species is more important than the exact times of separations, and we did not precisely calibrate time-scales between the referenced studies. Relevant literature for the respective branch knots [40-49] is indicated. Dotted lines relate to phylogenetic branch knots where the referenced literature was not informative on the absolute time of the event. Whole genome duplication events in a teleost ancestor (TGD), and early in the salmonid lineage (SGD), are indicated in red font.

To perform a comprehensive analysis of MHCI in teleosts, we first identified all MHCI genes in sequenced teleost genomes available in the Ensembl database. We found a total of 253 genes or gene fragments in the species cavefish (*Astyanax mexicanus,* AstMex102), zebrafish (*Danio rerio* ZV9), medaka (*Oryzias latipes,* Medaka1), platyfish (*Xiphophorus maculatus,* Xipmac4.4.2*),* tilapia (*Oreochromis niloticus,* Orenil 1.0), stickleback (*Gasterosteus aculatus,* BROAD S1), fugu (*Takifugu rubripes,* Fugu4.0) and tetraodon (*Tetraodon nigroviridis,* Tetraodon8.0) [Additional file 1: Figure S1, Additional file 2: Table S1]. For our model species Atlantic salmon and rainbow trout that we have analyzed intensively from various angles, we use the accepted MHC nomenclature e.g. Sasa-UBA for *Salmo salar* U lineage locus B [39] for the identified sequences. For the two other well-studied species, i.e. medaka and zebrafish, existing nomenclature is shown alongside our temporary nomenclature relating to species and consecutive location in the unique Ensembl genome (e.g. OL1 for *Oryzias latipes* and gene number 1). We have refrained from assigning definite MHCI gene names for those species that we do not experimentally investigate ourselves, as a correct nomenclature requires a thorough analysis of the quality of data, allelic relationships, expression levels, etc. The phylogenetic relationship between included species is shown in Figure 1. Predicting leader sequences as well as transmembrane and cytoplasmic domains is often difficult, leaving many of the 5′ and 3′ gene predictions incomplete. In addition, some genomes are more fragmented than others as seen in for instance tetraodon where 18 of 25 MHCI gene sequences are partials. Many of the gene fragments may still represent complete and functional genes, but they need further studies. We also investigated our model species Atlantic salmon (*Salmo salar,* AGKD00000000.3), where the final genome sequence was recently made available at NCBI. Here we add nine bona fide MHCI genes and five pseudogenes or gene fragments to the twelve genes previously reported in salmon (Additional file 3: Text S2) [29]. For Atlantic cod (*Gadus morhua*, NCBI GadMor_May2010) we only tried to identify non-U lineage genes, and for U lineage genes relied on previous reports, as genomic assembly of U lineage genes has been hampered by high sequence identity between loci [17].

To trace the evolution of teleost MHCI sequences we also investigated the genome of spotted gar (*Lepisosteus oculatus,* Ensembl LepOcu1*),* a species that branched off from the lineage leading to teleosts around 360 MYA [44] (Figure 1). We found 13 gar MHCI sequences residing on eight different scaffolds, of which five are complete sequences and eight are partial genes or gene fragments (Additional file 4: Text S1, Additional file 2: Table S1, and Additional file 1: Figure S1). Using available SRA (NCBI; Sequence Read Archive) reads we supplemented the 13 sequences with an assumed Z lineage gene consisting of alpha 1 and alpha 2 exons from unknown, possibly separated, genomic locations. Further definition of the functional status of these partial genes awaits additional cDNA sequencing. When relevant, we also investigated database resources for teleosts and other fishes without published whole genome sequence databases.

### Contrasting modes of evolution- the U lineage

Genes from the U lineage constitute 56% of the teleost genes summarized in the present study (Table 1). We found considerable U lineage expansions in tilapia and stickleback with 45 and 29 genes and gene fragments, while platyfish and tetraodon each showed medium expansions with 19 genes or gene fragments (Additional file 4: Text S1, Additional file 2: Table S1, Table 1). The U lineage expansions have previously been reported with 28 or more genes in tilapia [31], and approximately half of that in stickleback [17]. The discrepancy in stickleback may be due to the use of Q-PCR analysis for generating the previous estimate. However, the Ensembl genome estimate may also be questionable with stickleback scaffold 58 not yet linked to a chromosomal region and containing a myriad of genes for highly similar proteasome subunits and transport associated proteins in addition to multiple MHCI genes with high sequence identity, thus opening the possibility of assembly errors (Additional file 1: Figure S1). The remaining teleosts have a lower number of U lineage genes or gene fragments ranging from 4 in zebrafish to 13 in cavefish. The number of genes in each species may also vary as haplotypic variation has been reported in for example zebrafish, medaka and Atlantic salmon [25,29,50].

**Table 1:** **Number of MHC lineage genes in teleosts and spotted gar**

**Class**	**MHC Class I**						**MHC Class II**			
Species\Lineage	U	Z	L	S	P	Total	DA	DB	DE	total
Atlantic salmon	7	7	10	1	1	26	2*	5*	4*	11
Zebrafish	4	10	16	-	-	30	14*	7*	-	21*
Cavefish	13	18	2	7	2	42	4	1	-	5^3^
Medaka	13	5	-	-	-	18	6*	5*	-*	11*
Platyfish	19	3	-	-	-	22	n.a.	n.a.	n.a.	n.a.
Tilapia	45	6	1	-	-	52	33*	16*	-*	49*
Stickleback	29	1	-	-	-	30	11*	3*	-*	14*
Tetraodon	19	1	-	-	5	25	7*	-*	-*	7*
Fugu	8	2	-	-	24	34	7*	-*	-*	7*
Cod	100*	1	-	-	1	2	-*	-*	-*	-*
Spotted gar	5	1	2	-	4	12^1^	2^2^*		4*	6*
Total	162	55	31	8	37	293				

Number of genes and gene fragments identified in this study are shown for each species and each lineage. Data from other studies are marked with * and not counted (Cod U lineage gene estimate and lack of MHCII [17] and the remaining MHC class II data [19] where MHCII alpha and beta genes are counted separately).^1^Two of the Spotted gar MHCI sequences are of unknown lineage origin. ^2^Two Spotted gar MHCII sequences could not be defined as DA or DB and are thus shown as DA/DB here [19]. ^3^No thorough study was performed on cavefish MHC class II genes and platyfish MHC class II genes were not analyzed (n.a.).

The majority of U lineage genes reside within one syntenic region alongside typical MHC region “scaffold” genes such as *TCF19*, *RXRB, PSMB*, *ABCB3* and *TAPBP* genes (Additional file 1: Figure S1, Additional file 5: Table S2) as previously noted [29,51-55]. A few U lineage regions outside of this major MHC region show some regional syntenies between fish species, which we will not discuss further (Additional file 1: Figure S1, Additional file 5: Table S2).

We already know the number, genomic location and classification of most U lineage genes in several salmonids [14,15,29,55-57], medaka [13,43,58] and zebrafish [25]. For the U lineage molecules from platyfish, tilapia, stickleback, tetraodon and fugu, a number are expected to bind peptide termini in a way identical or similar to most classical MHCI based on conservation of predicted groove residues (Additional file 6: Text S3). Although defined as classical by Star et al. [17] , and both classical and non-classical by Malmstrøm et al. [20], the cod genes do not comply with the classical definition of high polymorphism within locus. Instead, cod seems to define a new way of providing MHCI variability, not in polymorphism within one or a few genetic loci, but instead using a high number of classical-similar genes with some variability, hereafter defined as polygenic variability. This is not very unlike the emerging picture for MHC class II evolution in some neoteleost fishes [19,59]. Defining classical loci in the remaining teleosts investigated here is problematic in part due to lack of transcript information and in part due to high sequence identity between reported sequences.

**Figure 2:**
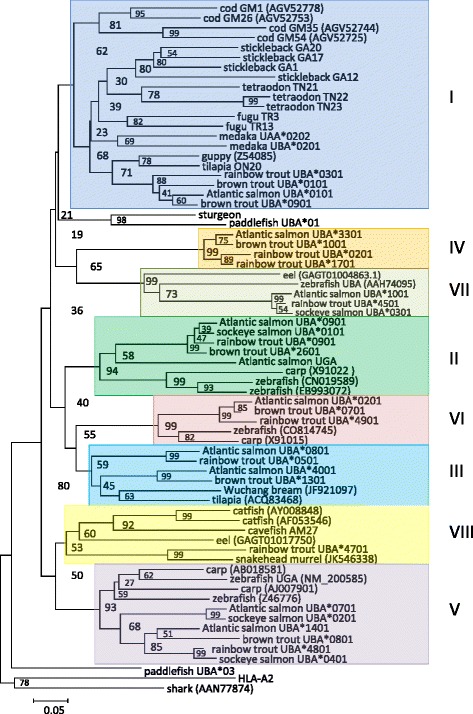
**Phylogeny of selected teleost MHCI U lineage alpha 1 domains.** Phylogenetic tree based on handmade alignment (shown in Additional file 6: Text S3) of selected alpha 1 domain amino acid sequences. The tree is made using Neighbor-joining P-distance and pairwise deletions. The tree is drawn to scale, with branch lengths representing the number of amino acid substitutions per site (see scale bar). Bootstrap values in percentage from1000 trials are shown. The various U lineage alpha 1 domain lineages [13,57] are shown as shaded boxes with Roman numbers outside each lineage cluster. Sequence GenBank references are as follows: Atlantic salmon (*Salmo salar*): UBA*0101 AAN75113, UBA*0201 AF504023, UBA*0701 AAN75109, UBA*0801 AAN75115, UBA*0901 AAN75119, UBA*1001 AAN75118, UBA*1401 AAN75110, UBA*4001 AEW27162, *UGA* ACX35601. Rainbow trout (*Oncorhynchus mykiss*): UBA*101 AF287483, UBA*401 AF287487, UBA*0501 AAG02508, UBA*0901 AAG02512, UBA*4501 AY278451, UBA*4701 AY278449, UBA*4801 AF318188, UBA*4901 AF318190. Brown trout (*Salmo trutta*): UBA*0101 AF296374, UBA*0701 AF296380, UBA*0801 AF296381, UBA*0901 AAG02528, UBA*1001 AF296383. Sockeye salmon (*Oncorhynchus nerka*, unpublished data): UBA*0101 KM085986, UBA*0201 KM085987, UBA*0310 KM085988 and UBA*0401 KM085989. Medaka (*Oryzias latipes*): UAA*0202 AB450991, UBA*0201 BAB83850.2. Paddlefish (*Polyodon spathula*): UBA*01 ACV87421, UBA*03 ACV87423 and sturgeon (*Acipencer sinensis*) ACV87437 [60]. Human: HLA-A2 AAA76608.2. Stickleback (GA; *Gasterosteus aculeatus*), tetraodon (TN; *Tetraodon nigroviridis*), fugu (TR; *Takifugu rubripes*), cavefish (AM; *Astyanax mexicanus*) and tilapia (ON20; *Oreochromis niloticus*) references are shown in Additional file 4: Text S2. Zebrafish (*Danio rerio*), carp (*Cyprinus carpio*), catfish (*Ictalurus punctatus*), cod (GM; *Gadus morhua*), tilapia lineage III, Wuchang bream (*Megalobrama amblycephala*), guppy (*Poecilia reticulata*), snakehead murray (*Channa striata*), eel (*Anguilla japonica*) and shark (*Squalus acanthias*) sequence references are shown in parenthesis in the figure. Alpha 1 domain phylogenies with more teleost sequences in addition to alpha 2 and alpha 3 domain phylogenies can be found in Additional file 6: Text S3.

When we analyzed the sequences separated into individual alpha 1, alpha 2 and alpha 3 domains, we found that sharing of highly divergent classical type MHCI alpha 1 domain lineages among species is an old teleost trait. There is also ancient variation in alpha 2, but the alpha 1 situation is much more pronounced so in the present paper we have therefore chosen to concentrate on alpha 1. Four of the alpha 1 domain lineages [13,57] date back to before the time a zebrafish ancestor separated from a salmonid/neoteleost ancestor, i.e. lineages II, III, V and VI (Figures 1 and 2). Two other alpha 1 lineages can be traced even further back to before an eel ancestor branched off from the major teleost lineage which may have occurred about 300 million years ago, i.e. lineages VII and VIII. Two remaining lineages are either found in salmonids only (lineage IV), or shared between salmonids and neoteleosts (lineage I). A suggested ninth lineage defined by the tilapia UAA and UBA genes [13] here defined as lineage IX, seems in part shared between the neoteleosts tilapia, medaka and platyfish (see Additional file 6: Text S3).

Interestingly, as in cod, all stickleback alpha 1 domains form one tight cluster as opposed to several other species where multiple alpha 1 domain lineages are found in common in even distantly related species. Further analysis of alpha 1 domains from cod and stickleback show that these two neoteleost species only have alpha 1 domains from lineage I (α1-I) (Figure 2, see Additional file 6: Text S3b for trees with inclusion of additional stickleback, cod and other neoteleost sequences). Although the bootstrap value supporting this α1-I clade in addition to the α1-III and α1-VIII clades are fairly low (48-60%), they are robust when including various sequences and reproducible between different studies ([13,57] and this study). In the present study we highlight the evolution of the alpha 1 sequences, but other regions of the U lineage molecules in cod and stickleback show a similar species-specific clustering upon phylogenetic analyses (examples in Additional file 6: Texts S3b2 and S3b3), indicative of relatively high turnover rates of the entire MHCI loci.

The α1-I lineage is also the predominant MHCI lineage in salmonids, being represented in 38% of the identified alleles and may thus define a lineage with some important evolutionary qualities, possibly in the establishment of new peptide binding groove variation (Additional file 6: Text S3c1). Divergence among salmonid α1-I lineage sequences is fairly high (70-97% identity), as is found among salmonid α1-III and α1-V sequences (67-91% and 65-97% identity, respectively) which are represented in fewer alleles than α1-I. The remaining lineages are less diverse (90-97% identity in the α1-II lineage and 95-97% identity in the α1-VII lineage), and are also fairly well retained in the investigated salmonid species. The big question therefore is, if some lineages like α1-I are superior in creating new binding groove variation, why are lineages such as α1-II and α1-VII, which show far less plasticity and do not appear to be extensively used for creating new alleles, not lost during evolution? In some other fish species such as cod and stickleback they are indeed lost, but why do salmonids and also cyprinids maintain these ancient lineages? A possible answer may be found in the fact that some of the “variation-poor” lineages comprise highly unique and, within that lineage, highly conserved residues, which are expected to interact with a peptide ligand (yellow shading in Additional file 6: Text S3a highlights lineage-specific residues, which in the case of lineages α1-II and α1-VII concern putative peptide binding residues). Thus these lineages may provide unique peptide binding properties that uniquely widen the spectrum of pathogen peptides that a species can present. However, for the relatively variation-poor lineage α1-VI such unique peptide binding features are not predicted, and analysis of MHCI evolution in mammals has shown that quite different peptide binding pockets can occur in a set of relatively similar sequences [61,62]. Possibly the highly divergent alpha 1 domains are readily distinguished by different natural killer cell receptor family molecules [63].

The fact that stickleback and cod share an evolutionary mode distinct from other investigated teleosts spurred us to look for more similarities between cod and stickleback. Molecules of one of the defined cod U lineage clades have a putative endosomal sorting motif in their cytoplasmic tail, which was hypothesized to optimize cross-presentation of exogenous peptides by MHCI, thus replacing the class II function [20]. When we analyzed stickleback genes, we found that 11 of 29 stickleback U lineage genes have a seventh exon encoding putative endosomal sorting motifs (Additional file 6: Text S3e-g). Although only one stickleback EST confirmed this exon sequence as an extension of the exon 6 sequence, the exon 7 sequences are highly conserved and without any functional selection one would have expected accumulation of point mutations and sequence divergence over time. In cod, assembly problems for the short reads of many almost identical genomic sequences from the 100 or more MHCI loci prohibit a detailed analysis of exon intron structures, but available evidence suggest a similar gene organization as stickleback based on alternate termination of cod cytoplasmic domains (data not shown and reference [20]). Although sticklebacks have several expressed MHC class II alpha and beta genes, including polymorphic ones (Table 1 and [19]), perhaps evolution is leading them down the same path as Atlantic cod, where the class II will eventually disappear alongside a continued expansion of class I genes. However, in mammals it is evident that the segregation into distinct MHC class I and II intracellular peptide loading compartments is not as complete as once thought [22,24], suggesting that the picture may also be more complex in teleosts.

### An ancient groove- the Z lineage

We found that all teleosts studied here have at least one expressed Z lineage gene while some have many (Additional file 4: Text S1, Additional file 2: Table S1). For Atlantic salmon we add three Z lineage genes (*ssZBAa*, *ssZCAa*, *ssZDAa,* Additional file 3: Text S2) to the four previously reported [29]. The three new genes reside in the major MHC class IA region on chromosome 27 in a location extending from the region with the previously identified Z lineage gene ss*ZAAa* (Additional file 3: Text S2). This region constitutes a duplicate of the previously identified IB region on chromosome 14 with the *ssZBAb*, *ssZCAb* and ss*ZDAb* genes. Five of the salmon Z lineage genes have functional support from gene expression assays while *ssZBAa* and ss*ZDAb* may be pseudogenes (Additional file 3: Text S2). In zebrafish, Dirscherl et al. [34] reported ten Z lineage genes with both allelic and haplotype variation. Cavefish, also belonging to the Ostariophysi, has an identical number of bona fide Z lineage genes (Additional file 2: Table S1, Additional file 4: Text S1). In medaka, Nonaka et al. [13] reported five Z lineage genes while we found that other investigated neoteleosts have from one detected Z lineage gene in cod, stickleback and tetraodon to four bona fide genes in tilapia (Additional file 4: Text S1, Additional file 2: Table S1, Table 1).

Atlantic salmon have all Z lineage genes within the duplicated MHCI regions IA and IB in between typical MHC region scaffold genes such as *TNXB* and *ATF6* (Additional file 1: Figure S1). Medaka and stickleback also have their Z genes linked to *TNXB* and *ATF6*, but here they reside in a region about 13 Mb outside the MHC region on the same chromosome. Two other neoteleosts i.e. platyfish and tilapia, both have their Z lineage genes linked to *LHX9*, *TNXB* and *ATF6*, but possible linkage to the classical MHCI region has not been clarified. Zebrafish also has a 10 Mb region separating classical U lineage genes from some of the typical MHC region scaffold genes *RPS18* and *VPS52* on Chr.19, but Z lineage genes reside either on Chromosome 1 or 3. We assume that the Z lineage genes originally resided in the extended MHC region, but have been distanced from the major MHC region through a large insertion or translocation in zebrafish and some neoteleosts. This organization of classical vs non-classical genes in medaka and stickleback resembles the situation in chicken and frog where the non-classical Rfp-Y and XNC genes are located far apart on the same chromosome as their classical counterparts, but segregate as unlinked loci [64-66].

When performing sequence alignments and phylogenetic analysis of teleost Z lineage genes, we found one major cluster within the Z lineage, here defined as Z1, containing members from all investigated teleosts (Additional file 7: Text S4). Cavefish and carps [6,32], also contain highly divergent sequences here denoted sub-lineage Z2 and Z3 (Additional file 7: Text S4). The cavefish Z2 group forms an out-group in both the alpha 1 and alpha 2 domain phylogenies while all cavefish Z alpha 3 domain sequences cluster together. This suggests sequence conservation or interlocus recombination driven by interaction with other molecules. Also contrary to most teleost Z1 lineage sequences, the Z2 and Z3 sequences might have lost their ability to bind peptides as most of the conserved peptide anchoring residues are missing. Both the cavefish Z1 and the Z2 groups are expressed as we found one EST supporting expression of the Z2 sequence AM2, while two transcriptomes contained expressed matches also for AM4 (Z2), AM8 (Z1) and AM19 (Z1) (Additional file 4: Text S1, Additional file 2: Table S1, *Astyanax mexicanus* Surface fish; SRX212200 and *Astyanax mexicanus* Pachon cavefish; SRX212201). This neo-functionalization may be unique to carps and cavefish where cavefish has its Z2 sub-lineage while the Z3 sub-lineage prevail in goldfish and carp. Why the Z lineage has been chosen for neo-functionalization in these species lines remains to be answered.

**Figure 3:**
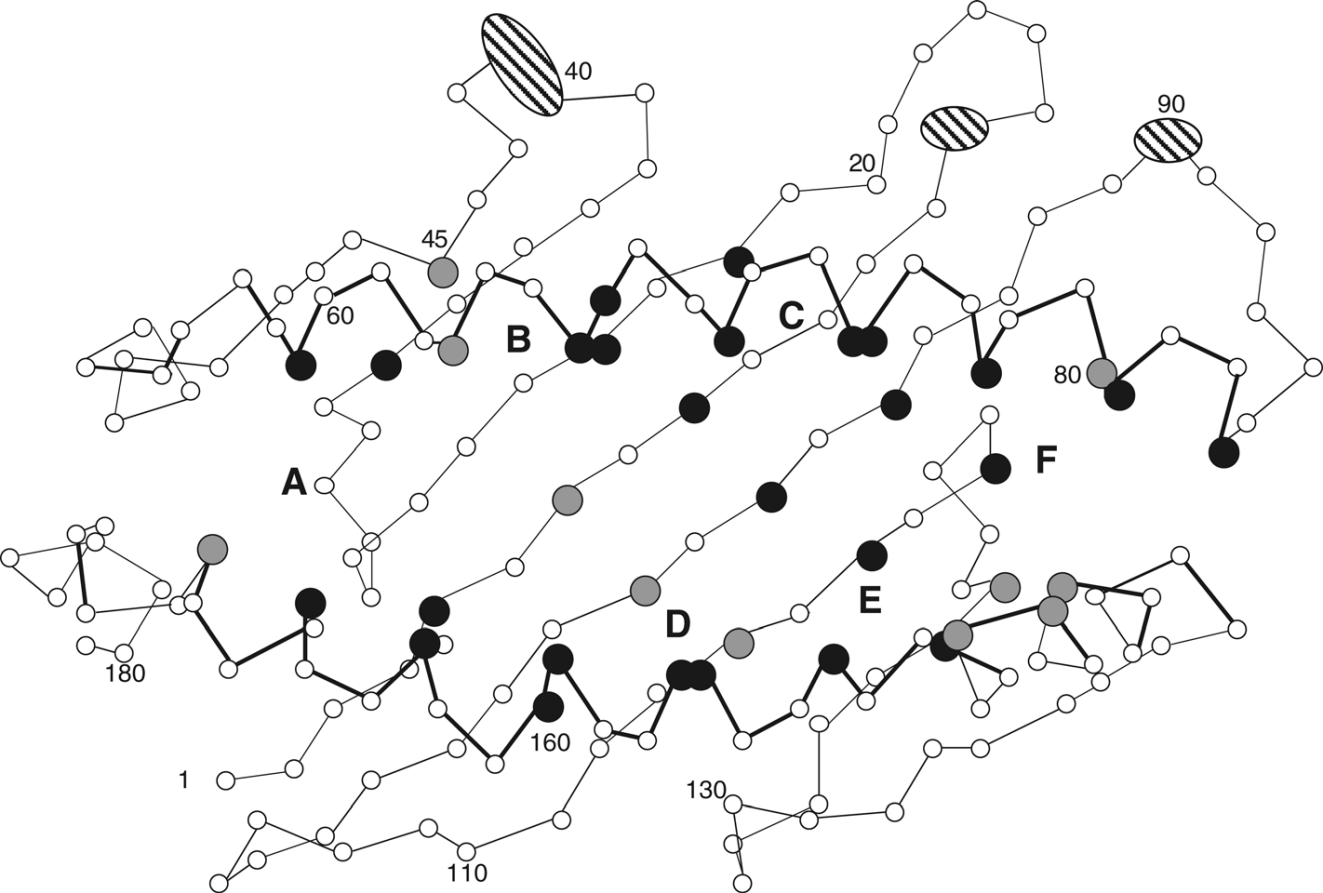
**Residue conservation of the Z1 sub-lineage α1 and α2 domains among teleosts.** Schematic presentation of the human HLA-A2 peptide binding groove defined by the alpha 1 and alpha 2 domains [3]. The 37 residue positions in HLA-A2 known to contribute to the six A through F pockets [1,3] are enlarged and colored black when completely conserved in the aligned teleost Z1 sub-lineage sequences while grey represents teleost sequence identity of 90-99,7%. Ellipses cover those HLA-A2 residues that, according to our sequence alignment, have few or no matching residues in the aligned teleost sequences. The figure is based on 31 Z1 lineage sequences from Atlantic salmon (*Salmo salar*), zebrafish (*Danio rerio*), medaka (*Oryzias latipes*), stickleback (*Gasterosteus aculeatus*), tilapia (*Oreochromis niloticus*), tetraodon (*Tetraodon nigroviridis*), fugu (*Takifugu rubripes*) and Amazon molly (*Poecilia formosa*), but excluding the Z1 lineage sequences from cavefish (*Astyanax mexicanus*). The alignment can be found in Additional file 7: Text S4.

A remarkable feature of Z lineage sequences appeared when studying the alignments in detail. Most teleost Z1 lineage sequences, including in eel, have an almost complete conservation of residues at the 37 positions known to provide the HLA-A2 molecule with its six A through F pockets that collectively comprise the peptide binding groove (Figure 3, Additional file 7: Text S4) [1,3]. These residues are conserved in sequences from the ray-finned fishes spotted gar and sturgeon in addition to one sequence from the lungfish, belonging to the Sarcopterygii. It should be noted, however, that the gar sequence is based on an assembly of possibly unlinked alpha 1 and alpha 2 genomic sequences, and the sturgeon sequence on an assembly of SRA reads of different related species (see Methods section). Thus, although typical sequences were the only sequence fragments found, future experiments will have to ascertain the existence of full-length typical and /or atypical Z lineage sequences in these primitive ray-finned fishes. Three Z1 lineage sequences from the recently published Amazon molly (*Poecilia formosa*) genome also comply with this sequence conservation (Additional file 4: Text S1, Additional file 7: Text S4). The majority of variation is seen in the cavefish Z1 lineage sequences where only 79% of the residues in the 37 positions are completely conserved (Additional file 7: Text S4). If disregarding cavefish Z1 sequences, but including the Amazon molly sequences, 99,9% of all residues in the 37 positions are conserved among 31 sequences from eight teleost species. How unusual this is, is shown in Additional file 7: Text S4b, which highlights the differences in conservation of (presumed) peptide binding residues among various categories of vertebrate MHCI sequences. It seems fair to assume that all Z1 molecules bind a highly similar or identical ligand. Exactly which ligand that is remains to be established, but this does suggest an important and highly conserved function for the typical Z molecules in all ray-finned fish and lungfish. We have so far not been able to detect Z lineage sequences in sharks or tetrapods. Whereas for lungfish previous studies only reported a Z sequence [67], our analysis of the SRA database retrieved a lungfish classical MHCI sequence (Additional file 4: Text S1), underlining the long co-existence of the Z and classical lineages.

### L lineage genes - a hydrophobic groove?

Through phylogenetic analyses we found Atlantic salmon orthologs of the *LCA* and *LDA* genes found in trout [26] in addition to four bona fide salmon genes with no published trout orthologs here denoted *LFA*, *LGA*, *LHA* and *LIA* (Additional file 3: Text S2). Three regions containing Atlantic salmon L pseudogenes *LJAΨ, LKAΨ* and *LLA/LMAΨ* were also identified (Additional file 1: Figure S1). We found matching Genbank ESTs for the three salmon genes *LDA*, *LFA* and *LGA* while a TSA transcript from skin confirmed expression of the fourth salmon gene *LCA* (GenBank accession JT833250, Additional file 3: Text S2). The salmon *LFA* and *LGA* genes are also present in trout as we found matching trout ESTs (GanBank accessions CA372488 and CA356147) while salmon lacks the trout genes *LAA*, *LBA* and *LEA*.

Five of the salmon regions show syntenies i.e. the *LCA-LGA/LFA* regions and the *LIA-LKA-LLA/LMA/LJA* regions. Because the salmon scaffolds and their physical locations are not yet publicly available, we tested the L gene regions against published markers [68]. We found markers placing the salmon *LIA* region on chromosome 21 (Additional file 3: Text S2) while none of the markers matched the remaining L regions. The salmon MHCI genes *UHA1* and *UHA2* also reside on chromosome 21, approximately 14,6 cM downstream of the LIA gene according to the female map.

L lineage genes are also present in zebrafish and tilapia. Zebrafish has 16 L lineage Ensembl genes, and 15 of these were described by Dirscherl et al. [38]. Thirteen of these genes are closely linked on Chr.25, two are closely linked on Chr.8 next to an MHCII alpha gene and the last gene is located on Chr.3 (Additional file 1: Figure S1). DR20 residing on Chr. 25, was not identified in the Dirscherl et al. study and has here been assigned the gene name *LPA*. Cavefish (*Astyanax mexicanus*) belonging to the order Characiformes, which like the Cypriniformes (e.g. zebrafish) and Siluriformes (e.g. catfish) are included in the superorder Ostariophysi, has one L lineage pseudogene (AM12) located in a region syntenic to the zebrafish L lineage genes DR17-29 on Chr.25 and another L pseudogene (AM32) located in a region syntenic to a zebrafish region lacking MHCI genes on Chr.15 (Additional file 5: Table S2). The single tilapia L lineage gene is located on scaffold GL831385 and is expressed according to a transcriptome shotgun assembly (TSA) match (GenBank accession GAID01031757.1), but lacks synteny with other L gene regions (Additional file 5: Table S2). Clustering of an L locus and MHC class II on zebrafish Chr. 8 suggests that the L lineage was established in an evolutionary period where the classical class I and class II genes were still linked. Such linkage of classical class I and II presumably exists in gar [19], and the linkage may have been lost after the whole genome duplication in a teleost ancestor around 350 MYA [19,69,70]. As none of the other teleosts with sequenced genomes discussed in this paper have L lineage genes or gene fragments, this lineage appears to have been lost in the majority of neoteleosts.

All the salmon sequences reported here comply with the unusual two exon gene organization reported for most trout L lineage genes [26] (Additional file 8: Text S5d), while the zebrafish genes display a traditional gene organization. The tilapia ON9 gene has a somewhat intermediate gene organization with three exons of 57, 746 and 286 base pairs respectively. Dijkstra et al. [26] suggested that the trout genes with unusual exon intron organization could have originated through retro transposition of partially spliced mRNA. This does not concern a very ancient event as for example zebrafish genes and the trout *LAA* gene have a traditional gene organization. The fact that tilapia only lost the intron between the alpha 1 and 2 domain exons is indicative of multiple events and complicates the explanation. The phylogenetic tree in Additional file 8: Text S5c suggests that a common ancestor with the tilapia type gene may have been the template from which further introns were lost in the salmonid lineage. The L lineage variability distribution resembles U lineage molecules in regard to having the highest similarity in the alpha 3 domain, and more divergence in the alpha 1 and 2 domains (Additional file 8: Text S5b).

**Figure 4:**
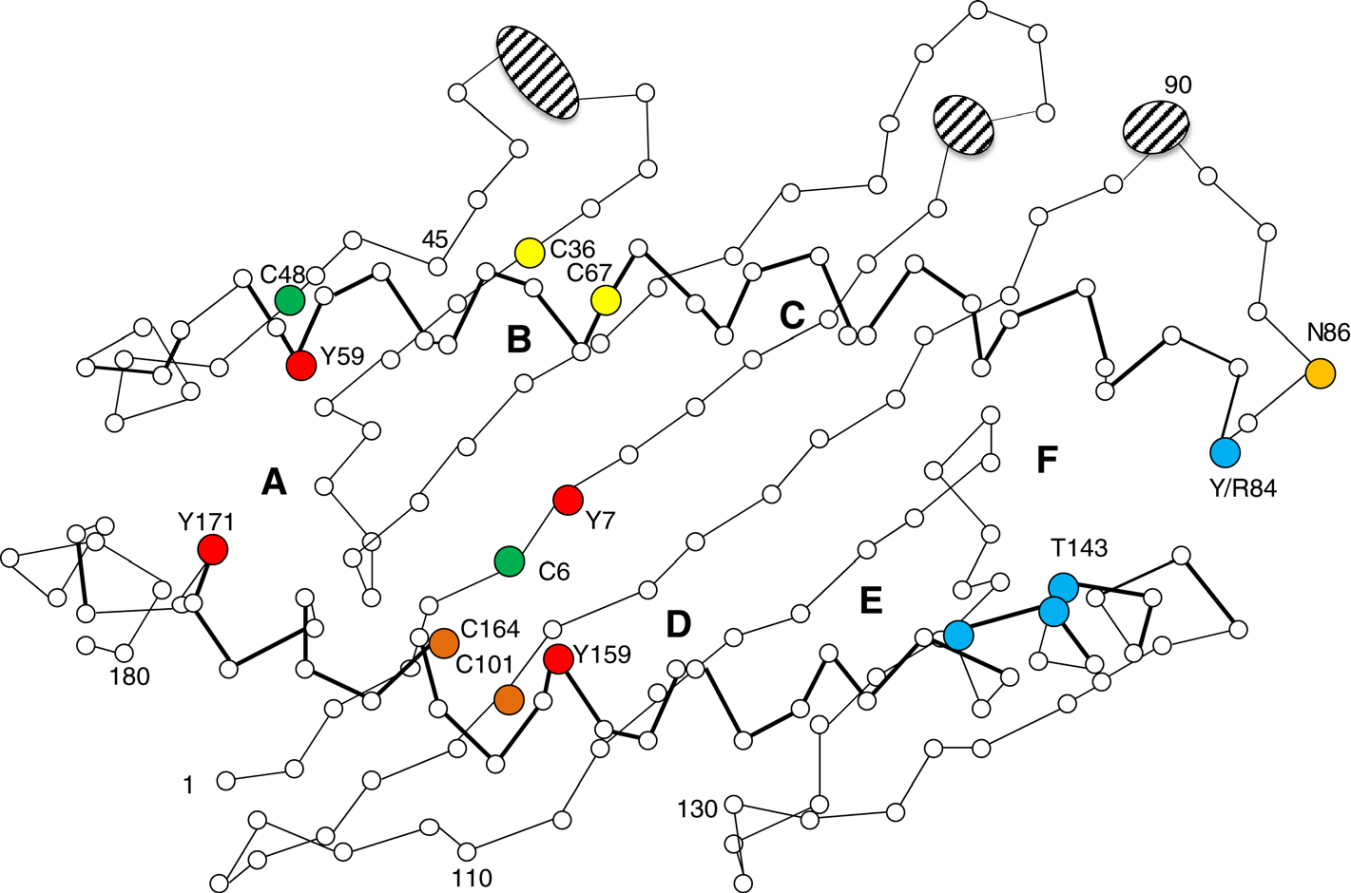
**Schematic figure of peptide binding groove.** Schematic presentation of the human HLA-A2 peptide binding groove defined by the alpha 1 and alpha 2 domains [1,3]. Round red circles indicate HLA-A2 amino acids anchoring peptide N-terminus and cyan circles are residues anchoring peptide C-terminus. Yellow circles show positions of the additional U and P lineage alpha 1 domain cysteines, green circles indicate positions of S lineage cysteines, and brown circles indicate the cysteine pair conserved in most MHC molecules. Orange circle shows the N-linked glycosylation site conserved in most classical MHCI molecules (N86). Ellipses cover those HLA-A2 residues that, according to our sequence alignment, have few or no matching residues in the aligned teleost sequences. The alignment can be found in Additional file 11: Text S8.

As noted previously, L lineage molecules do not contain the typical peptide-anchoring residues (Additional file 8: Text S5, Figure 4). This suggests that L lineage molecules most likely have other ligands or no ligands as noted previously [26]. An analysis of sequences from the five teleost MHCI lineages showed that L lineage molecules have the highest hydrophobicity within the two peptide-binding domains (Additional file 8: Text S5e-g) with an average hydrophobicity of -0,352. For comparison, the human HLA-A2 has a hydrophobicity score of -0,902 while the hydrophobicity of human CD1molecules range from -0,056 to -0,448. Although only a three dimensional structure can determine whether L molecules have a groove and whether the observed hydrophobicity maps to this groove, it is tempting to speculate that L lineage molecules may bind (glyco-)lipids or other hydrophobic ligands similar to for example mammalian CD1 molecules.

### S lineage genes- not only S after all

S lineage genes, initially identified by Shum et al. [27] in salmonids and defined as UAA, were later also found in catfish belonging to Siluriformes and then defined as a separate lineage called S for the species they were identified in [29]. Cavefish broadens this lineage providing six bona fide S lineage genes and one gene fragment (Additional file 4: Text S1, Additional file 9: Text S6). We also found transcribed S lineage sequences in ayu, belonging to the suborder Osmeridae related to salmonids (Figure 1). There is no published catfish genome, but the reported expressed sequences suggest that there are multiple S lineage loci also in this species. There are no syntenies between the two cavefish S regions and regions with MHCI in other teleosts, including the salmon S region (Additional file 5: Table S2, Additional file 1: Figure S1).

The cavefish S lineage sequences display two clusters corresponding to genomic locations represented by the sequences AM33 and AM38, suggesting the genes AM33-AM34 and AM38-AM42 were derived from intra-regional duplications. The AM33 vs AM38 duplication is old based on low sequence identity (Additional file 9: Text S6b). The catfish sequences cluster with the AM33/34 sequences while salmonids and ayu sequences form a separate cluster. Distribution of the S lineage in these species, place its origin back to before the split between the Ostariophysi (including e.g. cavefish and catfish) and Protacanthopterygii/Neoteleostei (including e.g. salmonids and ayu). Sequence identity is distributed similar to U lineage genes with highest identity in the alpha 3 domain and lowest identity in the alpha 1 domain (Additional file 9: Text S6b).

Hallmarks of MHCI such as the alpha 2 and alpha 3 domain cysteines are conserved in all S lineage sequences, but in addition the sequences have some uncommon cysteines in the alpha 1 domain (Additional file 9: Text S6a). Some have one or two cysteines at position 6-9 (numbering according to HLA-A2*)* while some have an additional cysteine at position 48. The cavefish AM37-AM41 sequences also have an additional cysteine in their alpha 2 domain at position 100. The positioning of the alpha 1 domain cysteines do not align with the cysteine present in some U lineage sequences such as ON3 (data not shown). Structural importance of the C6 and C9 residues is difficult to imagine, but the C48 residue may be involved in dimer formation as found for HLA-G dimers (Figure 4) [71].

As opposed to other lineages, S lineage sequences have fairly short cytoplasmic domains (Additional file 9: Text S6). Such a short cytoplasmic tail has in the human HLA-G molecule been shown to result in retention in the ER and a much longer half-life at the cell surface [71]. The S lineage seems functional in species such as ayu, catfish and five of the cavefish genes which have matching expressed support (Additional file 4: Text S1, Additional file 2: Table S1). However, the peptide anchoring residues typical for classical MHCI are not conserved in S lineage sequences, suggesting they have non-peptide or no ligands.

### A fifth teleost MHC class I lineage- P for penta

During the course of this study we discovered genes belonging to an as yet un-described teleost MHCI lineage, here defined as the P lineage as we first detected it in pufferfishes and it defines the fifth (penta) identified teleost MHC class I lineage. This lineage is present in Atlantic cod, tetraodon, fugu, Atlantic salmon, sablefish, seabass and cavefish (Additional file 4: Text S1, Additional file 1: Figure S1, Additional file 10: Text S7). A vast expansion of this lineage has occurred in fugu displaying 24 genes or gene fragments whereof eight genes contain α1 through α3 domains. Four genes were found in tetraodon, where two contain complete mature ORFs while cod has only one P lineage gene here denoted Cod *PAA*. For P lineage in striped seabass one TSA transcriptome report was found (GenBank accession GBAA01146398) and for sablefish, we found one P lineage EST report which had a stop codon disrupting the ORF (GenBank accessions GO625557 and GO625558). This stop codon was verified in a TSA transcriptome sequence (GenBank accession JO689867) and may thus define a transcribed pseudogene. Cavefish has one P lineage gene with expressed support (AM5, SRA dataset SRX212201) in addition to a likely pseudogene (AM6). The single P lineage gene in Atlantic salmon is a pseudogene.

Tetraodon and fugu share one P region with partial synteny (Additional file 1: Figure S1, Additional file 5: Table S2). In tetraodon, the genes TN3-TN7 are flanked by the genes *CHST12*-*IL2RB*-*MPST*-*ASRGL1*-*SMCR7L*-*ATF4B2* on UnR: 6.4 Mb. In fugu, these genes reside on Scf.209 (Chr.5 in the Fugu5 assembly) approx. 250 kb outside of the TR4-TR5 genes, suggestive of a local rearrangement. The Atlantic salmon P pseudogene does not display any regional synteny with the Tetraodontidae regions, but is instead located alongside Immunoglobulin light chain genes on Chromosome 7 (Additional file 3: Text S2, Additional file 1: Figure S1). This link between MHCI and IgL genes is also seen in other teleosts where Medaka has some IgL chain genes linked to the UIA and Z lineage genes on Chr.11 (Additional file 1: Figure S1) [72] and stickleback has IgL genes linked to its Z lineage gene on Chr.10 [73]. As previously noted, as IgL genes are also linked to some typical MHC region genes in elephant shark [74], this linkage is probably the remnant of the primordial MHC [75-78].

The sequence identity between P lineage sequences and the four other teleost lineages is fairly low ranging from 11-33% in each of the three extracellular domains (Additional file 10: Text S7). The within P lineage sequence identity is 20-99% with cod and cavefish as the species with most divergent sequences. Within fugu the sequence identity is higher within all domains reflecting recent gene duplications (82-100%), but the alpha 2 domain has slightly more variable residues than the alpha 1 domain while the alpha 3 domain is most conserved.

P lineage molecules have the classical conserved alpha 2 and alpha 3 domains cysteines, but in addition they have two conserved cysteines in the alpha 1 domain (C36, C67; Additional file 10: Text S7). The positioning of these cysteines align with those found in some U lineage sequences (Additional file 6: Text S3) [13], but not with those found in S lineage sequences (Additional file 9: Text S6). The average distance between cysteines in alpha 2 and alpha 3 domains is 55-60 aa, while alpha 1 cysteines in U and P lineage sequences are only 21-29 aa apart. When aligned or visualized in three dimensions using the human HLA-A2 sequence as reference, these cysteines reside in close physical proximity, at a position where they could form a bond between the beta sheet and the alpha 1 helix and thus influence the flexibility and shape of the ligand binding groove (Figure 4). The second cysteine at position C67 also aligns with the alpha 1 domain cysteine in HLA-B27, and alternatively might be involved in dimerization of the molecule. In humans, the HLA-B27 monomer is recognized by the inhibitory receptors LILRB1, LILRB2 and KIR3DL1, while the dimer molecule is recognized by different receptors [71]. Future studies are needed to clarify the role(s) of teleost alpha 1 domain cysteines.

Although one may question the accuracy of a linear alignment against the HLA-A2 sequence, P sequences do not have the typical peptide-binding residues, and even seem to have a deletion surrounding the otherwise conserved N-terminal anchoring residue Y59 (Additional file 10: Text S7a, Figure 4). These molecules are thus non-classical MHCI molecules potentially binding non-peptide or no ligands. Their expression signatures underline their non-classical nature. In cod, we found one transcript originating from a beard library (GenBank accession GW844691.1, Additional file 4: Text S1) and one match from a brain transcriptome library (Additional file 4: Text S1, SRA dataset SRX148752) while transcriptomes from the immunologically important tissues spleen, hindgut and head kidney in addition to the organs heart, gonad and liver (SRA053026) were all negative. Also tetraodon displayed P lineage expression in the brain (SRA dataset SRX191169) suggesting P genes have a biological role in teleost brain potentially in line with the roles emerging for mammalian classical as well as non-classical MHCI molecules in brain development and function [79-81]. Expression of classical MHCI in developing fish brain was suggested by linkage analysis to be associated with behavior such as level of boldness [82,83], but no studies have yet focused on the function of non-classical MHCI in fish brains. Fugu does not have brain transcriptomes, but displayed P lineage transcription (SRA dataset SRX363279) in gills suggesting the gene may also have other roles.

### Lineage distribution and deeper phylogeny

All teleost species studied have both U and Z lineage genes although the number of genes within each of these lineages varies dramatically with 7-45 U lineage genes and 1-18 Z lineage genes. Both U and Z lineages encompass genes which (probably) encode peptide-binding molecules, but only the U lineage contains highly polymorphic classical genes. It seems superfluous to have two lineages with peptide binding abilities, but with the complete conservation of the predicted peptide binding groove in typical Z lineage sequences, a specific conserved and important functional role emerges for this lineage.

The only species we found with all five teleost MHCI lineages were Atlantic salmon and cavefish, although the P lineage was only represented by a pseudogene in salmon while the L lineage seems to be dysfunctional in cavefish (Table 1). The S, L, and P lineages are unlikely to bind peptides and are unevenly distributed in the studied teleosts (Table 1). Stickleback and medaka completely lack all these three lineages, fugu and tetraodon lack L and S lineages while zebrafish and tilapia lack S and P lineages. Cavefish has L pseudogenes but an expanded S lineage. So what function do these lineages hold and how can these functions be maintained in species lacking these lineages? A similar picture also emerged when studying teleost MHC class II [19]. The class II DA lineage was present in all studied teleosts with the exception of Gadoids and contained the classical polymorphic MHC class II alpha and beta genes in addition to non-classical genes. The other class II lineages DB and DE lineages only contained non-classical genes and were unevenly distributed amongst teleosts. One would expect genes with vital functions to be present in all species so why are some lineages of seemingly non-vital importance maintained in some teleosts? Could the various MHCI lineages perform identical biological roles despite not sharing sequence characteristics or have various teleost species developed different ways to handle immune responses perhaps uniquely adapted to their different environments and pathological pressures? It is easy to forget that teleosts are a highly diversified branch where individual species such as zebrafish, salmon and various neoteleosts have had considerable time to develop different immune strategies suitable for their various environments.

**Figure 5:**
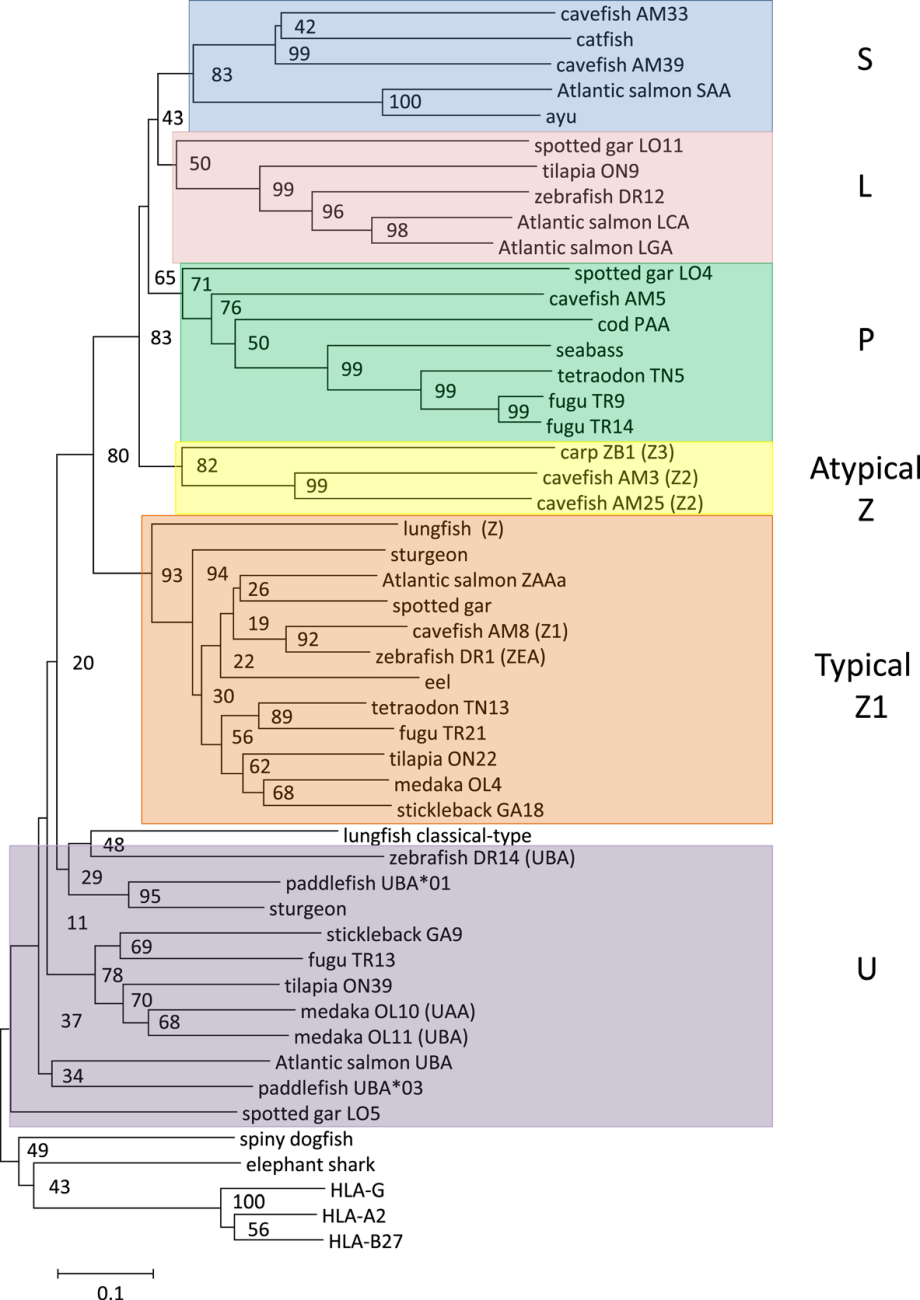
**Phylogeny of alpha 1 domain sequences from all five MHCI lineages.** Phylogenetic tree of selected alpha 1 domain amino acid sequences from the five defined lineages. The tree is base on hand-made alignment shown in Additional file 11: Text S8a and rooted using the human, shark and chimaera sequences. The tree is drawn to scale, with branch lengths representing the number of amino acid substitutions per site (see scale bar). Bootstrap values in percentage based on 1000 trials are shown on each node. GenBank sequence references not found in Additional file 4: Text S1 or Additional file 3: Text S2 are as follows: [S lineage] ayu (*Plecoglossus altivelis*) is JP747954, catfish (*Ictalurus punctatus*) is JT320438; [P lineage] seabass (*Morone saxatilis*) is GBAA01146398; [Z lineage] carp (*Cyprinus carpio*) ZB1 is L10420, lungfish (*Protopterus aethiopicus*) is AAF15304.1; [U lineage] zebrafish (*Danio rerio*) UBA is AAH74095.1, medaka (*Oryzias latipes*) UAA is AB450991and UBA is BAB83850.2, salmon (*Salmo salar*) UBA is AAN75116.1, paddlefish (*Polyodon spatula*) UBA*01 is ACV87421 and UBA*03 is ACV87423 while sturgeon (*Acipencer sinensis*) is ACV87437. The remaining sequence references are: spiny dogfish (*Squalus acanthias*) is AAN77874, elephant shark (*Callorhinchus milii*) is AFM85876.1, human (*Homo sapiens*) HLA-G is EAX03236.1, HLA-A2 is AAA76608.2, and HLA-B27 is CAA83876.1. The five lineages are shown on the right of each colored box. Alternative locus designations and the atypical Z lineage definitions Z2 and Z3 are shown in parenthesis behind the sequence names.

Phylogenetic analyses of individual domains showed U lineage sequences to be present in teleosts in addition to the ray-finned fishes spotted gar, sturgeon and paddlefish (Figure 5, Additional file 4: Text S1, Additional file 11: Text S8). The Z lineage appears to be older than the split between ray-finned and lobe-finned fish as Z lineage sequences were found in all teleosts in addition to spotted gar, sturgeon, and lungfish. Z lineage identity of the isolated lungfish sequence was already proposed by Stet et al. [37] and Dijkstra et al. [26], but the model is now corroborated by the Z sequences found in primitive ray-finned fish. Hence, it can be concluded that the classical MHCI (in ray-finned fish represented by U) and Z lineages separated more than 430 Mya ago (Figure 1). Although previous studies had already postulated Z lineage identity of the hitherto single lungfish sequence based on phylogenetic tree analysis [37,67], only the finding of Z in primitive ray-finned fish and the analysis of the conserved putative peptide binding groove in the present paper makes this lungfish Z identity a solid observation. This is older than can be solidly concluded for separation from the classical branch for any non-classical lineage found in tetrapods such as for example CD1 (even when studying as CD1/PROCR lineage), which if judging by presence in extant species (reptiles, birds and mammals) can only be traced to 312 MYA ([84] and unpublished data).

Spotted gar is as far back as we were able to trace the L lineage and potentially also the P lineage although the bootstrap value is low and the LO4 sequence lacks an alpha 3 domain potentially obscuring a definite lineage definition (Figure 5, Additional file 11: Text S8). S lineage sequences could be traced to a common ancestor of Ostariophysi and Protacanthopterygii, but were not found beyond these teleost superorders. Probably lineages L, P, and S originated from duplications of genes of the older U and/or Z lineages. But it is currently impossible to reconstruct from which of the two lineages they originated. From the unclear evolutionary scenario at deeper ray-finned fish levels it also follows that at these deep levels in some instances our working definitions of U, Z, P, S and L, which are based on phylogenetic tree analysis, may not correctly represent genuinely separate lineages. More sequence information from primitive ray-finned fish would be needed to properly determine the origins of the highly divergent L, P and S lineages.

## Conclusion

Our study of the teleost MHCI revealed five highly distinct MHCI lineages where only the lineages U and Z include molecules with predicted peptide binding residues and are present in all studied species. The remaining lineages S, L and P are found in some, but not in other species promoting questions as to their functional relevance. In most teleost fish species the U lineage appears represented by both classical as well as non-classical genes, but two very different modes of evolution can be observed. U lineage sequences in teleost species like for example medaka, zebrafish and salmon are characterized by multiple highly divergent alpha 1 sequences representing ancient domain lineages, and these can be shuffled onto variable alpha 2 plus downstream sequences to increase U allelic variation; in these fishes only one or few highly expressed classical type genes are found. On the other hand, in Atlantic cod and stickleback, all of the many detected U lineage genes were derived from a multitude of relatively recent duplications of genes having alpha1-I lineage sequences and older diversifications appear to have been lost; at least cod has a rather large number of expressed classical type sequences, and we suggest to describe this model of classical MHCI as “polygenic”. Whereas most tetrapod and fish species have classical MHCI and related nonclassical MHCI (in teleost fish classical and nonclassical U lineage members), all teleost fish seem to have typical Z and only a few teleost fish species groups seem to have atypical Z. Thus, in regard to the ligand binding characteristics, the Z lineage is hardly used for generation of derived nonclassical/ atypical molecules as opposed to the U lineage. Typical Z molecules appear to have the most ancient peptide binding groove conserved until today, because, from before ray-finned fishes and lungfish separated, they almost completely preserved their 37 residues that match the peptide binding residues of human HLA-A2. In summary, instead of understanding MHCI evolution within teleosts as “classical MHCI plus varying distribution of nonclassical MHCI” as known for tetrapods, we should understand teleost MHCI evolution as “classical U, plus typical Z, plus varying distribution of nonclassical/ atypical MHCI”. Besides clarification of the MHCI situations at the single species level, future research will have to elucidate the reason for this fundamental difference between the animal classes.

## Methods

### Data mining and bioinformatics

A mixture of annotated and un-annotated MHCI sequences were identified using Ensembl’s Biomart and the GO/IPR term for class I (GO: 0042613/ IPR001039) supplemented with various blastN and TblastN searches of Ensembl and NCBI databases using evolutionary diverged as well as species-specific sequences. It should be noted that the analysed genomic databases from cavefish (*Astyanax mexicanus,* AstMex102), zebrafish (*Danio rerio* ZV9), medaka (*Oryzias latipes,* Medaka1), platyfish (*Xiphophorus maculatus,* Xipmac4.4.2*),* tilapia (*Oreochromis niloticus,* Orenil 1.0), stickleback (*Gasterosteus aculatus,* BROAD S1), fugu (*Takifugu rubripes,* Fugu4.0) and tetraodon (*Tetraodon nigroviridis,* Tetraodon8.0), Atlantic salmon (*Salmo salar,* AGKD00000000.3), Atlantic cod (*Gadus morhua*, NCBI GadMor_May2010) and spotted gar (*Lepisosteus oculatus,* Ensembl LepOcu1*)* each represent one or a limited number of animals so more genes or other alleles may exist in other haplotypes/ animals. Potential genomic assembly errors would also influence our analyses. For Atlantic salmon, we supplemented the 12 known Atlantic salmon MHCI genes [29] with blastN and TblastN searches using preliminary salmon genome sequences available at either cGRASP [85] or NCBI [86]. Open reading frames were predicted using GenScan [87], Fgenesh [88] and Augustus [89] and/or by aligning with expressed sequences using Spidey [90]. Some smaller pseudogene remnants that did not contribute to evolutionary understanding were neglected. Expressed match was either identified through TblastN search against EST resources using MHCI alpha 3 domains or when this approach was negative expressed match was sought using the entire coding sequence in GenBank nucleotide (cDNA) and subsequently available TSA/SRA resources. The transcriptome (TSA/SRA) accession numbers used are as follows: tetraodon (Brain: SRX191169), fugu (Testis: SRX363280, gills: SRX363279, liver: SRX362038, various organs: SRX189142, SRX188889 and SRX188888), Atlantic cod (eggs: SRX148753, brain: SRX148752, head kidney: SRX148751, liver: SRX148750, hind gut: SRX148749, gonad: SRX148748, spleen: SRX148740), stickleback (brain: SRX146601), cavefish (surface fish: SRX212200, Pachon cavefish: SRX212201) and African lungfish SRX152529. The Z lineage sequence identified in spotted gar (*Lepisosteus oculatus*) derive from individual brain transcriptome reads (SRX543528) assembled using the CAP3 [91] program. The sturgeon Z lineage alpha 1 domain sequence is assembled from near identical genomic reads primarily from the sturgeon species *Acipenser persicus* (SRA dataset ERX145719; ERR169830.1125422.1) with a 14 bp gap filled using a *Acipenser baerii* sequence (SRA dataset ERX145721; ERR169832.3958173.2). The sturgeon alpha 2 domain sequence is assembled from the near identical sequences primarily from *Acipenser persicus* (SRA dataset ERX145719; ERR169830.5438448.1, ERR169830.5438448.2, and ERR169830.5083693.2), with a 10 bp gap filled using a *Acipenser gueldenstaedtii* sequence (SRA dataset ERX145720 sequence ERR169831.3185933.1). Three dimensional structures were aligned against the HLA-A2 structure using the Swiss PDB-viewer [92,93].

### Phylogenetic analysis

All alignments of MHCI amino acid sequences were performed using ClustalX for initial analyses [94] and later manually curated based on structural aspects and alignment with tetrapod sequences. The phylogenetic trees were inferred using the Neighbor-Joining method [95] with bootstrap testing according to Felsenstein [96]. The evolutionary distances were computed using the p-distance method [97]. Evolutionary analyses were conducted in MEGA5 [98].

### Availability of supporting data

The Atlantic salmon genomic scaffold sequences were deposited in the DRYAD repository [99,100] with the following identifier: doi:10.5061/dryad.928fj.

## Additional files

Additional file 1: Figure S1. Ray-finned fish MHCI regions. Additional file 2: Table S1. MHCI gene ID, genomic location and EST match. Additional file 3:
**Text S1.** Additional Atlantic salmon data. Additional file 4:
**Text S2.** MHCI amino acid sequences. Additional file 5: Table S2. Regional syntenies in selected MHCI regions. Additional file 6:
**Text S3.** Additional U lineage data. Additional file 7:
**Text S4.** Additional Z lineage data. Additional file 8:
**Text S5.** Additional L lineage data. Additional file 9:
**Text S6.** Additional S lineage data. Additional file 10:
**Text S7.** Additional P lineage data. Additional file 11:
**Text S8.** Comparison of all lineages.

## Abbreviations

MHC: Major histocompatibility complex
MYA: Million years ago
TGD: Teleost-specific whole genome duplication
SGD: Salmonid-specific whole genome duplication
