# Supplementary material for: A comprehensive analysis of teleost MHC class I sequences
Source: BMC Evol Biol. 2015 Mar 6;15:32. doi: 10.1186/s12862-015-0309-1 (PMC4364491; doi:10.1186/s12862-015-0309-1)
Supplement: Additional file 1: Figure S1. — Ray-finned fish MHCI regions. [file 12862_2015_309_MOESM1_ESM.pdf]

Additional file 1: Figure S1. Ray-finned fish MHC class I regions

Table of Content	Page
Atlantic salmon (*Salmo salar*) MHC class I regions	2
Cavefish (*Astanyx mexicanus*) MHC class I regions	4
Zebrafish (*Danio rerio*) MHC class I regions	5
Medaka (*Oryzias latipes*) MHC class I regions	6
Platyfish (*Xiphophorus maculatus*) MHC class I regions	7
Tilapia (*Oreochromis niloticus*) MHC class I regions	8
Stickleback (*Gasterosteus aculeatus*) MHC class I regions	9
Tetraodon (*Tetraodon nigroviridis*) MHC class I regions	10
Fugu (*Takifugu rubripes*) MHC class I regions	11
Spotted gar (*Lepisosteus oculatus*) MHC class I regions	12

Figure legend: The genomic surroundings of MHC class I genes in ten fishes are shown with genes represented by blocks. Data were derived from the Ensembl database, except those of Atlantic salmon (Additional file 3: Text S1). Several genes flanking the MHC class I genes were identified using the Ensembl genome browser and, in some cases, gene prediction software. Identities of all genes without Ensembl annotation were investigated by blasting their deduced products against GenBank. When identity was uncertain, phylogenetic analysis (Neighbor Joining method) was performed. Sequences with somewhat questionable sequence identity and members of complex gene families were given the name extension "–like" (_L), while those without good match were defined as not determined (n.d.) and mostly omitted for space purposes. Names of genomic regions and MHCI genes used in this study are often simplified references to species and Ensembl scaffolds/contigs or linkage groups/chromosomes, with details given vertically. For precise location of individual MHC genes see Additional files 2:Table S1, 3:Text S1 and 4:Text S2. Yellow arrows indicate availability of information on extensions of the depicted region fragments, while green stars indicate absence of such information. Black line linking rectangles represent regional gaps where the distance is shown. Red line linking rectangles represent a continuous region introduced due to space problems. Please be aware that some gene families such as the zinc finger protein family (ZNF) can be extensively dispersed throughout the genome and that their shared presence is not a good indication for regional synteny. Further colorings of individual genes are as follows: orange= MHC class II, red= MHC class I, blue= MHC region scaffold genes as found in human, gray= other Genes and black=genes found in the fugu5 assembly. Presumable pseudogenes are marked with ψ, and names of MHCI genes with evidence of transcription (Table S1, Text S1) are in red font. Zebrafish D8.46A refers to an MHC class II gene described in Dijkstra et al. [main text reference 19]. The black arrow pointing at region rTR4 indicates position of P-gene in syntenic tetraodon rTN3 scaffold. Regions published previously are shown with grey boxes and reference. The Atlantic salmon region boxed and shaded yellow represents an overlap between the previously published BAC sequence (Lukacs et al.[main text reference 29]) and the genome scaffold sequence. Lineages or alternative gene names are shown in parenthesis. When information on chromosomal location is missing, the region has been provided with an R prior to the first MHCI gene of that region. Regions with syntenic genes are boxed with identical colors, syntenic genes as rectangles in same color and marked S1 through S11. Regions with one MHCI gene only are not shown.

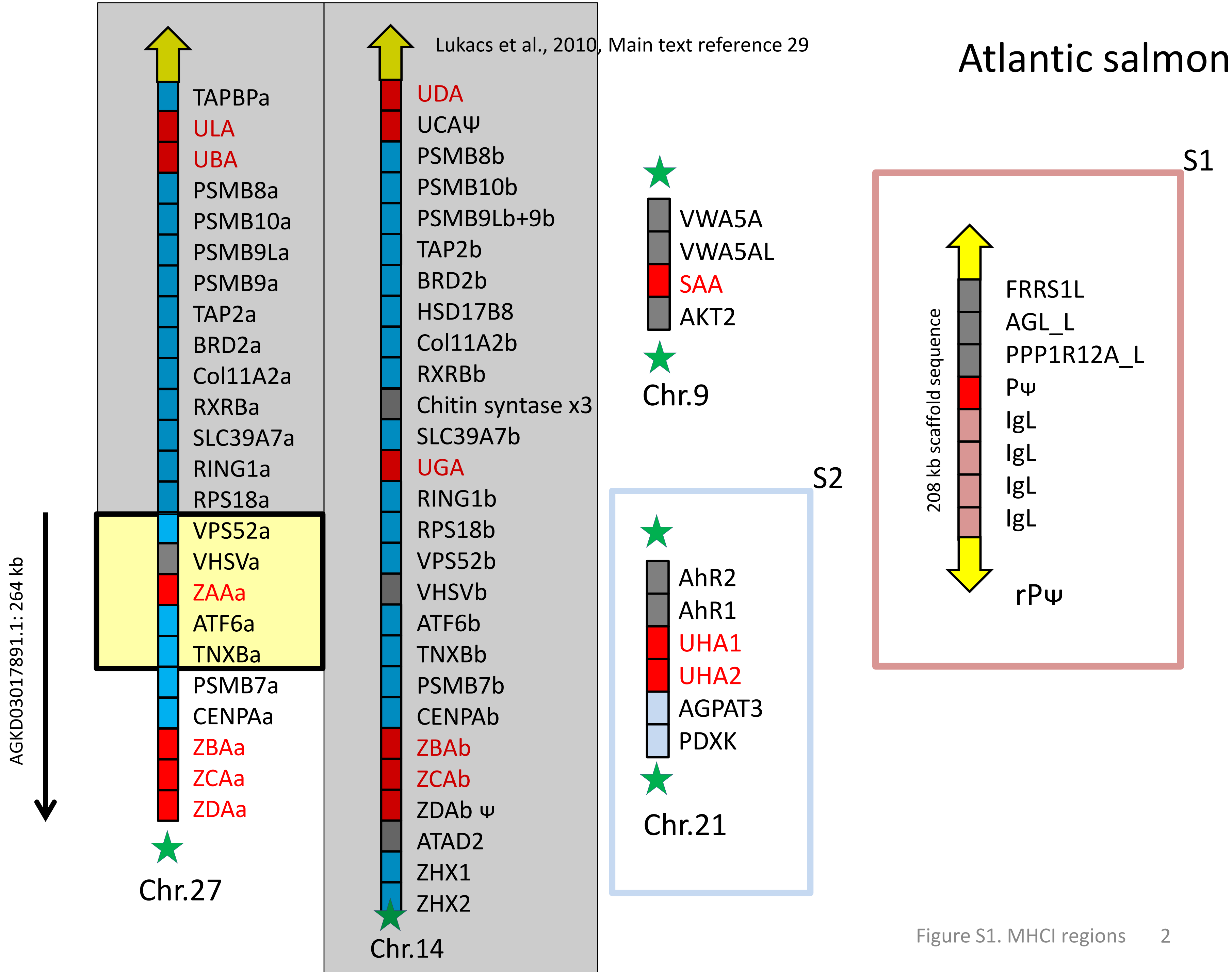
Atlantic salmon
Lukacs et al., 2010, Main text reference 29
TAPBPa
ULA
UBA
PSMB8a
PSMB10a
PSMB9La
PSMB9a
TAP2a
BRD2a
Col11A2a
RXRBa
SLC39A7a
RING1a
RPS18a
VPS52a
VHSVa
ZAAa
ATF6a
TNXBa
PSMB7a
CENPAa
ZBAa
ZCAa
ZDAa
AGKD03017891.1: 264 kb
Chr.27
UDA
UCAΨ
PSMB8b
PSMB10b
PSMB9Lb+9b
TAP2b
BRD2b
HSD17B8
Col11A2b
RXRBb
Chitin syntase x3
SLC39A7b
UGA
RING1b
RPS18b
VPS52b
VHSVb
ATF6b
TNXBb
PSMB7b
CENPAb
ZBAb
ZCAb
ZDAb ψ
ATAD2
ZHX1
ZHX2
Chr.14
VWA5A
VWA5AL
SAA
AKT2
Chr.9
S2
AhR2
AhR1
UHA1
UHA2
AGPAT3
PDXK
Chr.21
S1
208 kb scaffold sequence
FRRS1L
AGL_L
PPP1R12A_L
Pψ
IgL
IgL
IgL
IgL
rPψ

Atlantic salmon cont.

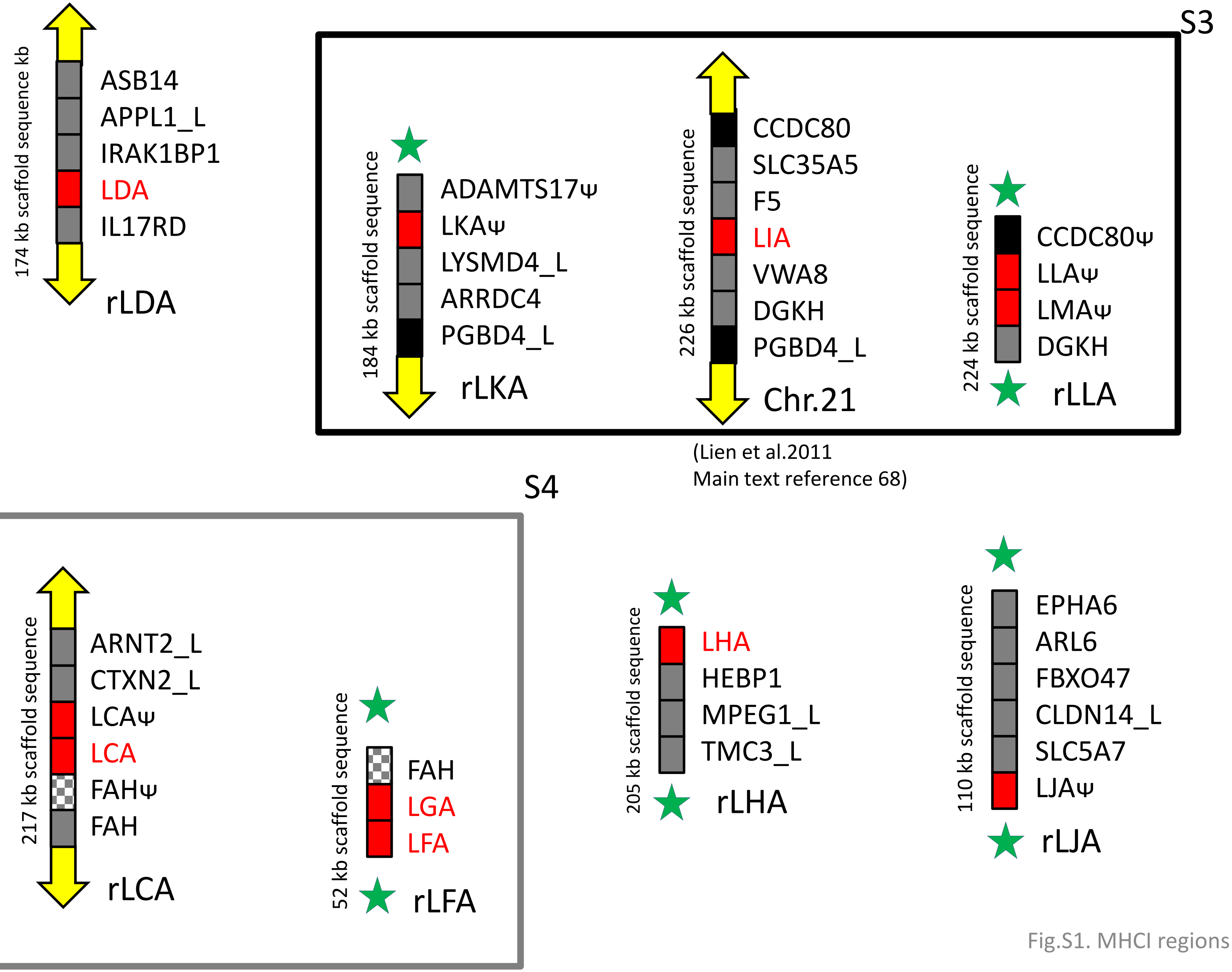

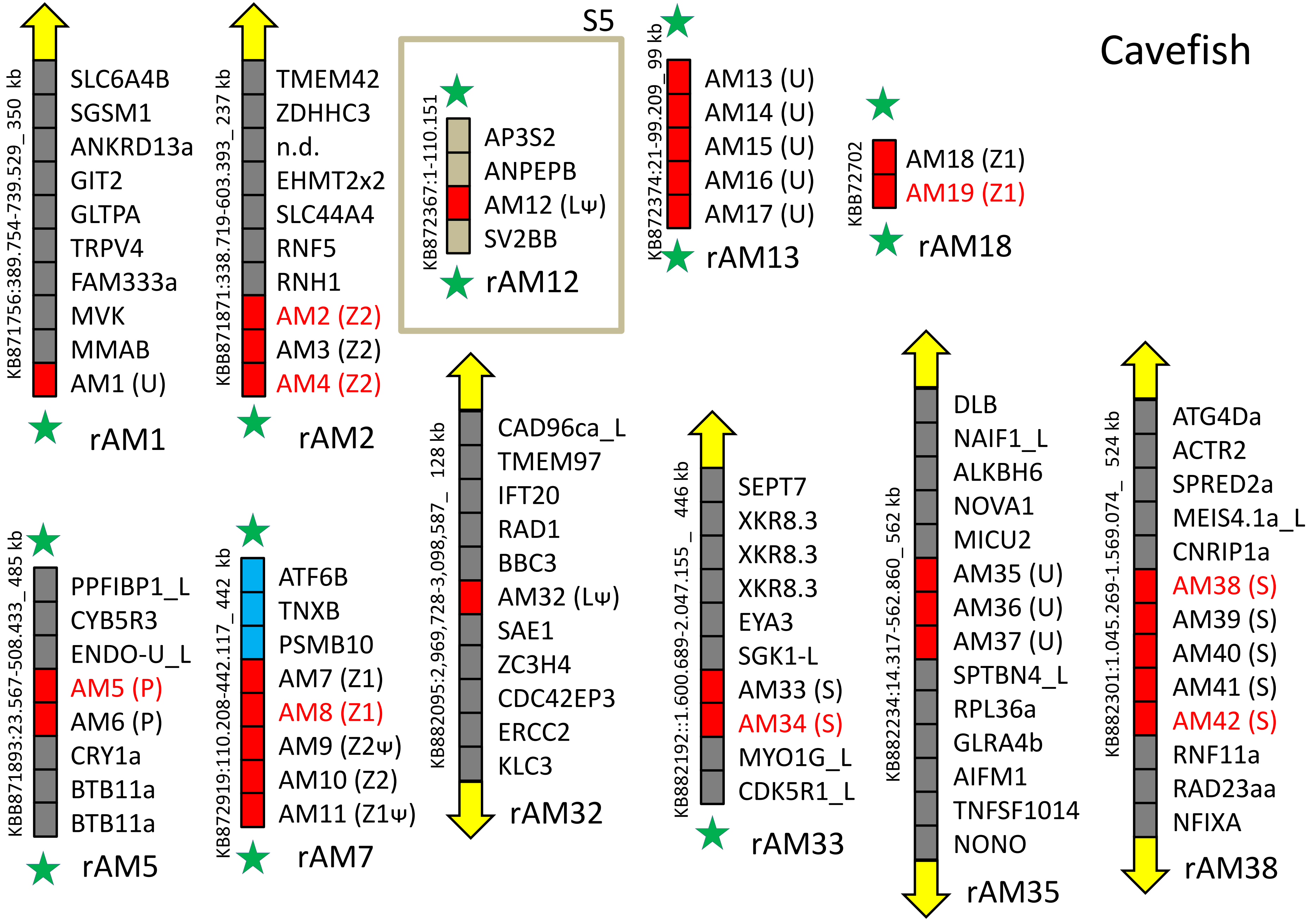
Cavefish
S5
KB871756:389.754-739.529_ 350 kb
SLC6A4B
SGSM1
ANKRD13a
GIT2
GLTPA
TRPV4
FAM333a
MVK
MMAB
AM1 (U)
rAM1
KB871871:338.719-603.393_ 237 kb
TMEM42
ZDHHC3
n.d.
EHMT2x2
SLC44A4
RNF5
RNH1
AM2 (Z2)
AM3 (Z2)
AM4 (Z2)
rAM2
KB872367:1-110.151
AP3S2
ANPEPB
AM12 (Lψ)
SV2BB
rAM12
KB872374:21-99.209_ 99 kb
AM13 (U)
AM14 (U)
AM15 (U)
AM16 (U)
AM17 (U)
rAM13
KB872702
AM18 (Z1)
AM19 (Z1)
rAM18
KB871893:23.567-508.433_ 485 kb
PPFIBP1_L
CYB5R3
ENDO-U_L
AM5 (P)
AM6 (P)
CRY1a
BTB11a
BTB11a
rAM5
KB872919:110.208-442.117_ 442 kb
ATF6B
TNXB
PSMB10
AM7 (Z1)
AM8 (Z1)
AM9 (Z2ψ)
AM10 (Z2)
AM11 (Z1ψ)
rAM7
KB882095:2,969,728-3,098,587_ 128 kb
CAD96ca_L
TMEM97
IFT20
RAD1
BBC3
AM32 (Lψ)
SAE1
ZC3H4
CDC42EP3
ERCC2
KLC3
rAM32
KB882192::1.600.689-2.047.155_ 446 kb
SEPT7
XKR8.3
XKR8.3
XKR8.3
EYA3
SGK1-L
AM33 (S)
AM34 (S)
MYO1G_L
CDK5R1_L
rAM33
KB882234:14.317-562.860_ 562 kb
DLB
NAIF1_L
ALKBH6
NOVA1
MICU2
AM35 (U)
AM36 (U)
AM37 (U)
SPTBN4_L
RPL36a
GLRA4b
AIFM1
TNFSF1014
NONO
rAM35
KB882301:1.045.269-1.569.074_ 524 kb
ATG4Da
ACTR2
SPRED2a
MEIS4.1a_L
CNRIP1a
AM38 (S)
AM39 (S)
AM40 (S)
AM41 (S)
AM42 (S)
RNF11a
RAD23aa
NFIXA
rAM38

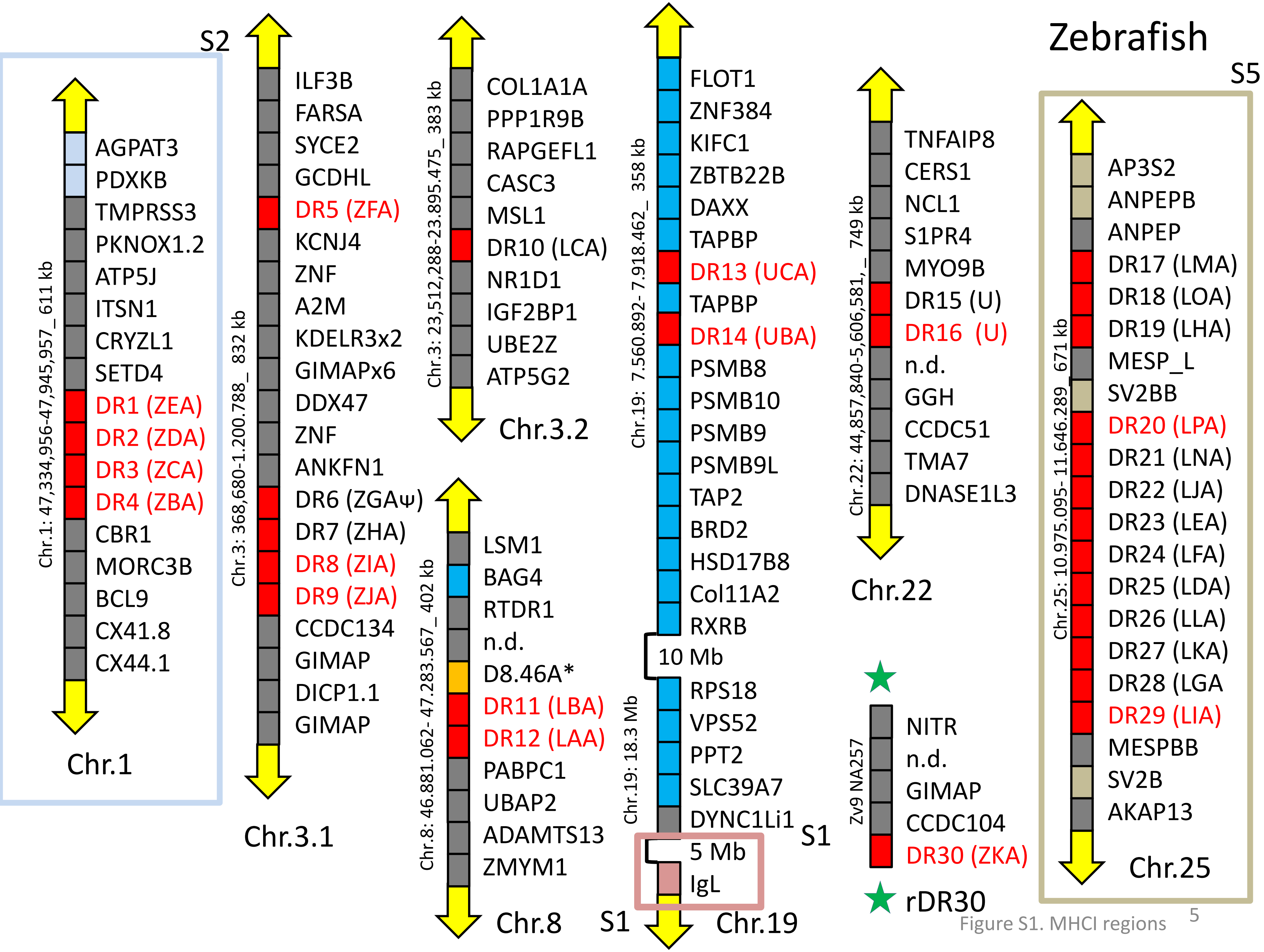

Figure S1. MHCI regions

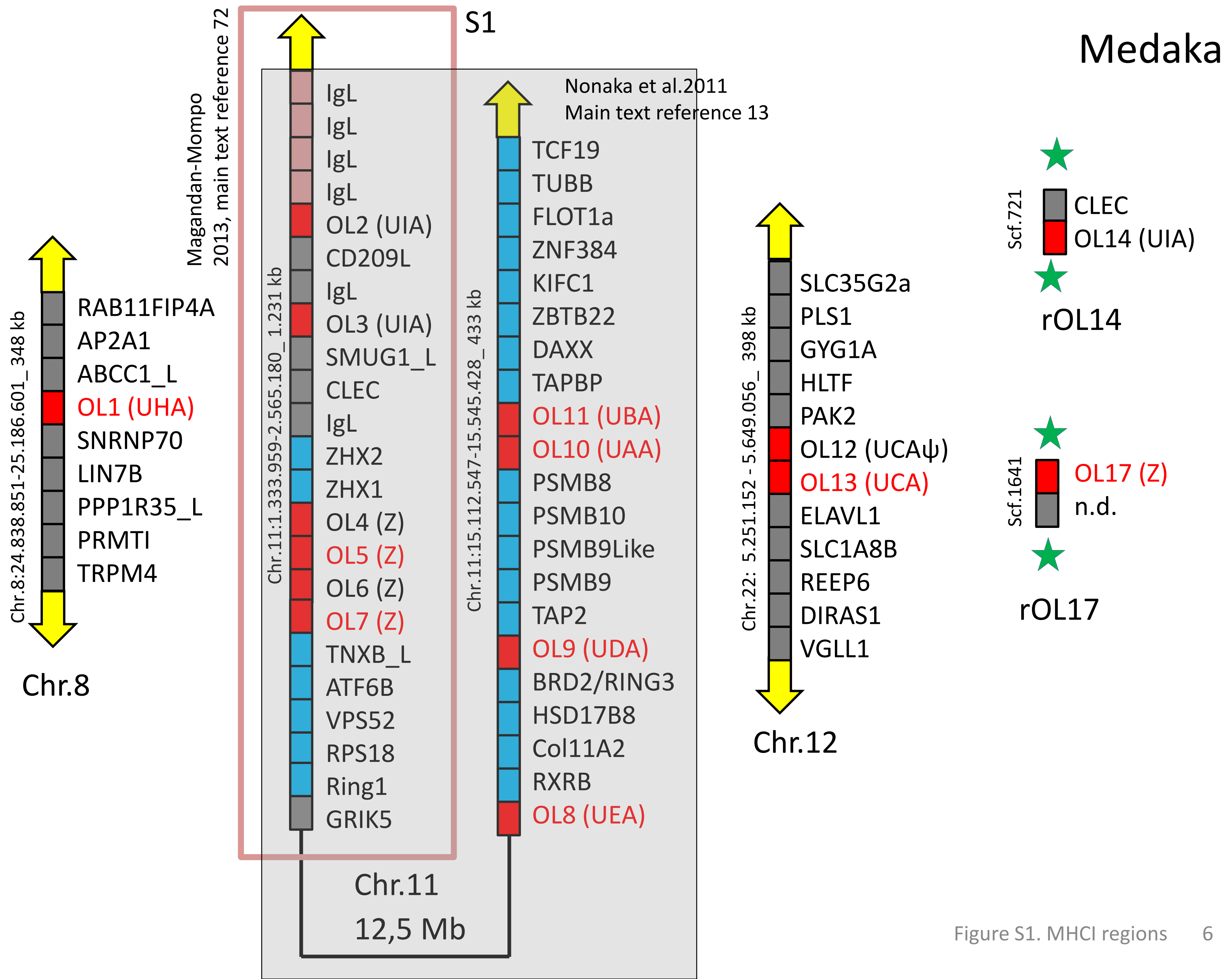
Medaka
S1
Magandan-Mompo 2013, main text reference 72
Nonaka et al.2011
Main text reference 13
Chr.8:24.838.851-25.186.601_ 348 kb
RAB11FIP4A
AP2A1
ABCC1_L
OL1 (UHA)
SNRNP70
LIN7B
PPP1R35_L
PRMTI
TRPM4
Chr.8
Chr.11:1.333.959-2.565.180_ 1.231 kb
IgL
IgL
IgL
IgL
OL2 (UIA)
CD209L
IgL
OL3 (UIA)
SMUG1_L
CLEC
IgL
ZHX2
ZHX1
OL4 (Z)
OL5 (Z)
OL6 (Z)
OL7 (Z)
TNXB_L
ATF6B
VPS52
RPS18
Ring1
GRIK5
Chr.11:15.112.547-15.545.428_ 433 kb
TCF19
TUBB
FLOT1a
ZNF384
KIFC1
ZBTB22
DAXX
TAPBP
OL11 (UBA)
OL10 (UAA)
PSMB8
PSMB10
PSMB9Like
PSMB9
TAP2
OL9 (UDA)
BRD2/RING3
HSD17B8
Col11A2
RXRB
OL8 (UEA)
Chr.11
12,5 Mb
Chr.22: 5.251.152 - 5.649.056_ 398 kb
SLC35G2a
PLS1
GYG1A
HLTF
PAK2
OL12 (UCAψ)
OL13 (UCA)
ELAVL1
SLC1A8B
REEP6
DIRAS1
VGLL1
Chr.12
Scf.721
CLEC
OL14 (UIA)
rOL14
Scf.1641
OL17 (Z)
n.d.
rOL17

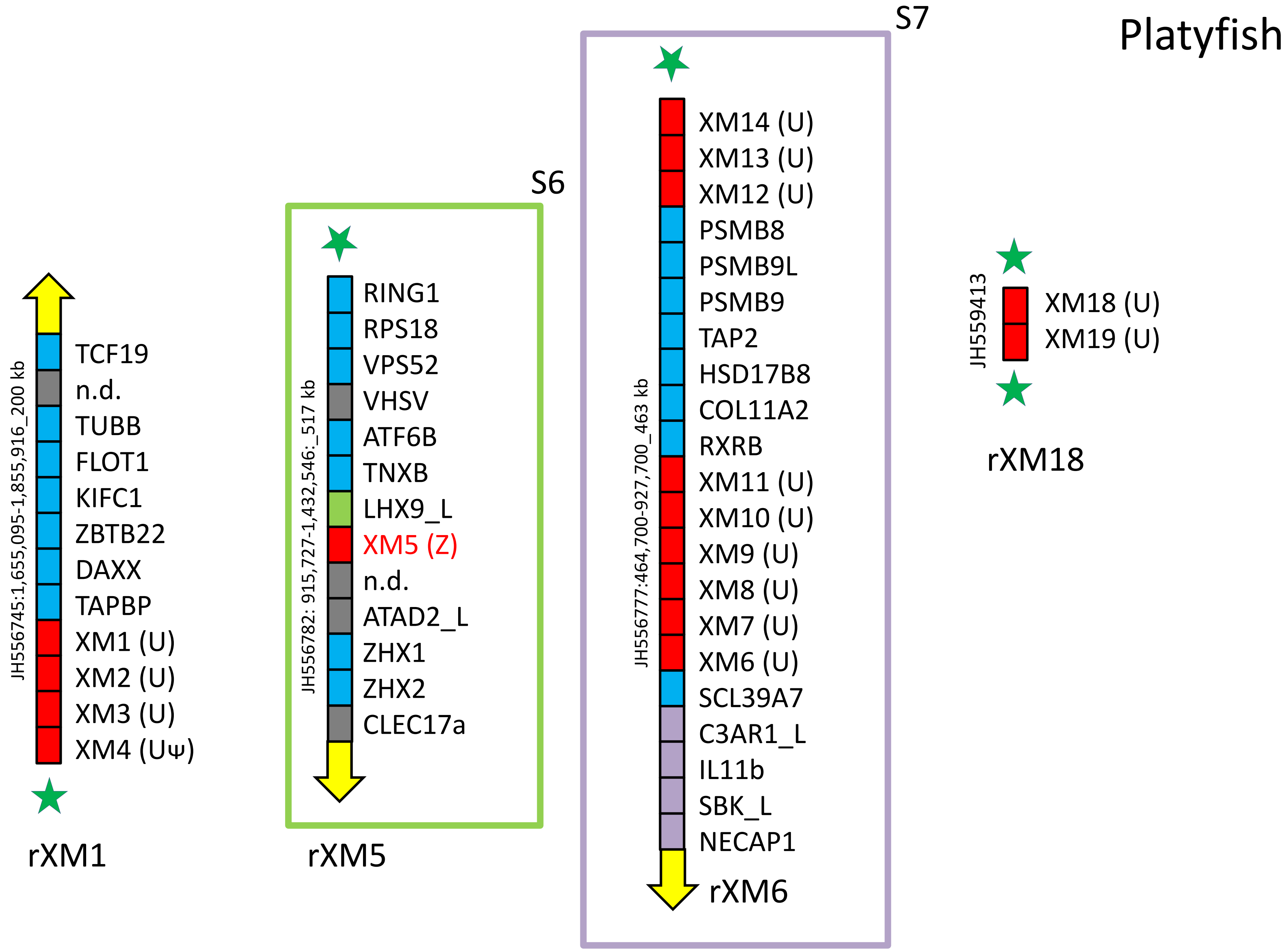
Platyfish
S6
S7
JH556745:1,655,095-1,855,916_200 kb
TCF19
n.d.
TUBB
FLOT1
KIFC1
ZBTB22
DAXX
TAPBP
XM1 (U)
XM2 (U)
XM3 (U)
XM4 (Uψ)
rXM1
JH556782: 915,727-1,432,546:_517 kb
RING1
RPS18
VPS52
VHSV
ATF6B
TNXB
LHX9_L
XM5 (Z)
n.d.
ATAD2_L
ZHX1
ZHX2
CLEC17a
rXM5
JH556777:464,700-927,700_463 kb
XM14 (U)
XM13 (U)
XM12 (U)
PSMB8
PSMB9L
PSMB9
TAP2
HSD17B8
COL11A2
RXRB
XM11 (U)
XM10 (U)
XM9 (U)
XM8 (U)
XM7 (U)
XM6 (U)
SCL39A7
C3AR1_L
IL11b
SBK_L
NECAP1
rXM6
JH559413
XM18 (U)
XM19 (U)
rXM18

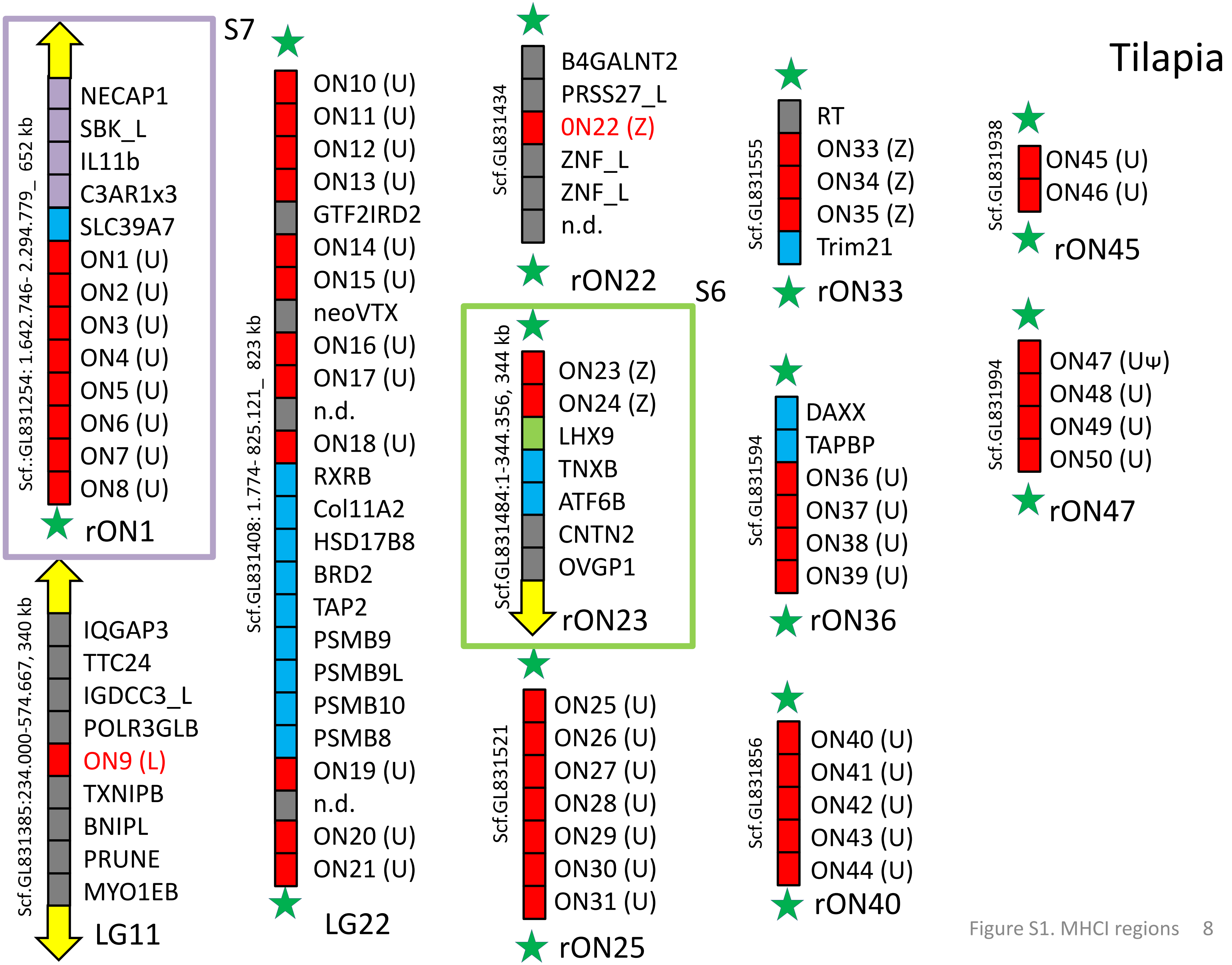
Tilapia
S7
Scf.:GL831254: 1.642.746- 2.294.779_ 652 kb
NECAP1
SBK_L
IL11b
C3AR1x3
SLC39A7
ON1 (U)
ON2 (U)
ON3 (U)
ON4 (U)
ON5 (U)
ON6 (U)
ON7 (U)
ON8 (U)
rON1
Scf.GL831385:234.000-574.667, 340 kb
IQGAP3
TTC24
IGDCC3_L
POLR3GLB
ON9 (L)
TXNIPB
BNIPL
PRUNE
MYO1EB
LG11
Scf.GL831408: 1.774- 825.121_ 823 kb
ON10 (U)
ON11 (U)
ON12 (U)
ON13 (U)
GTF2IRD2
ON14 (U)
ON15 (U)
neoVTX
ON16 (U)
ON17 (U)
n.d.
ON18 (U)
RXRB
Col11A2
HSD17B8
BRD2
TAP2
PSMB9
PSMB9L
PSMB10
PSMB8
ON19 (U)
n.d.
ON20 (U)
ON21 (U)
LG22
Scf.GL831434
B4GALNT2
PRSS27_L
ON22 (Z)
ZNF_L
ZNF_L
n.d.
rON22
S6
Scf.GL831484:1-344.356, 344 kb
ON23 (Z)
ON24 (Z)
LHX9
TNXB
ATF6B
CNTN2
OVGP1
rON23
Scf.GL831521
ON25 (U)
ON26 (U)
ON27 (U)
ON28 (U)
ON29 (U)
ON30 (U)
ON31 (U)
rON25
Scf.GL831555
RT
ON33 (Z)
ON34 (Z)
ON35 (Z)
Trim21
rON33
Scf.GL831594
DAXX
TAPBP
ON36 (U)
ON37 (U)
ON38 (U)
ON39 (U)
rON36
Scf.GL831856
ON40 (U)
ON41 (U)
ON42 (U)
ON43 (U)
ON44 (U)
rON40
Scf.GL831938
ON45 (U)
ON46 (U)
rON45
Scf.GL831994
ON47 (Uψ)
ON48 (U)
ON49 (U)
ON50 (U)
rON47

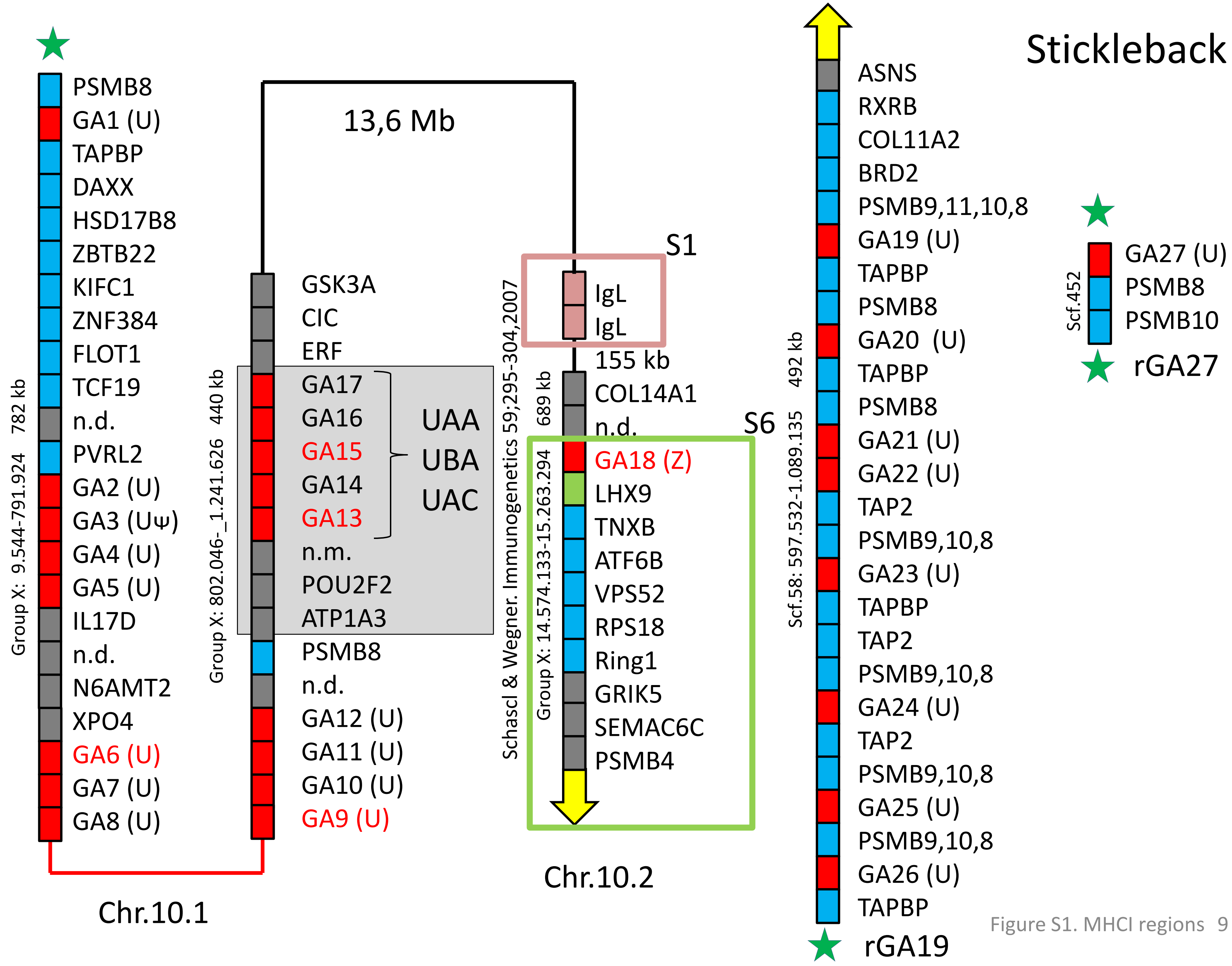
Stickleback
PSMB8
GA1 (U)
TAPBP
DAXX
HSD17B8
ZBTB22
KIFC1
ZNF384
FLOT1
TCF19
n.d.
PVRL2
GA2 (U)
GA3 (Uψ)
GA4 (U)
GA5 (U)
IL17D
n.d.
N6AMT2
XPO4
GA6 (U)
GA7 (U)
GA8 (U)
Group X: 9.544-791.924 782 kb
Chr.10.1
13,6 Mb
GSK3A
CIC
ERF
GA17
GA16
GA15
GA14
GA13
UAA
UBA
UAC
n.m.
POU2F2
ATP1A3
PSMB8
n.d.
GA12 (U)
GA11 (U)
GA10 (U)
GA9 (U)
Group X: 802.046-_1.241.626 440 kb
Schascl & Wegner. Immunogenetics 59;295-304,2007
S1
IgL
IgL
155 kb
COL14A1
n.d.
S6
GA18 (Z)
LHX9
TNXB
ATF6B
VPS52
RPS18
Ring1
GRIK5
SEMAC6C
PSMB4
Group X: 14.574.133-15.263.294 689 kb
Chr.10.2
ASNS
RXRB
COL11A2
BRD2
PSMB9,11,10,8
GA19 (U)
TAPBP
PSMB8
GA20 (U)
TAPBP
PSMB8
GA21 (U)
GA22 (U)
TAP2
PSMB9,10,8
GA23 (U)
TAPBP
TAP2
PSMB9,10,8
GA24 (U)
TAP2
PSMB9,10,8
GA25 (U)
PSMB9,10,8
GA26 (U)
TAPBP
rGA19
Scf.58: 597.532-1.089.135 492 kb
GA27 (U)
PSMB8
PSMB10
Scf.452
rGA27

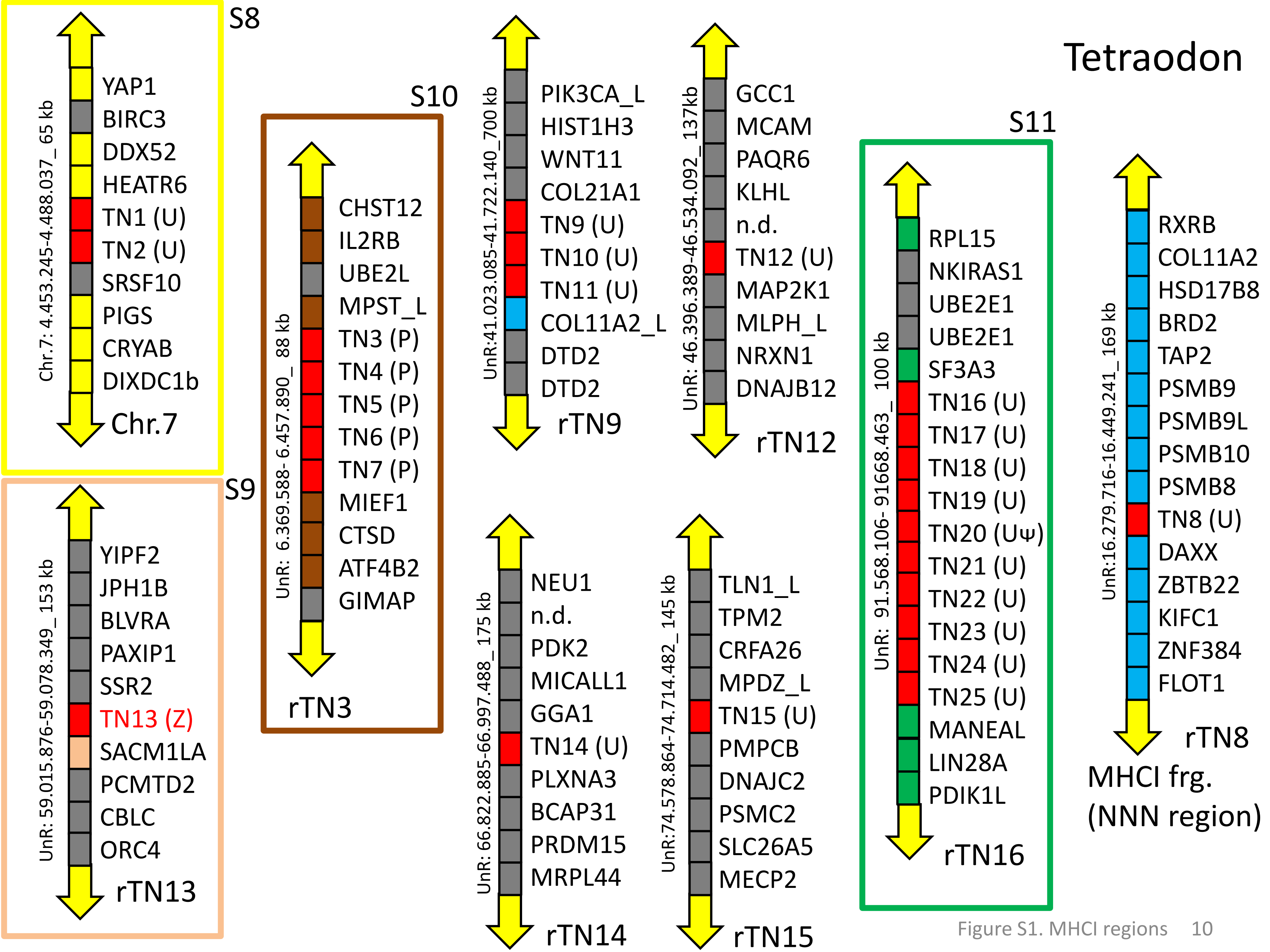
Tetraodon
S8
Chr.7: 4.453.245-4.488.037_ 65 kb
YAP1
BIRC3
DDX52
HEATR6
TN1 (U)
TN2 (U)
SRSF10
PIGS
CRYAB
DIXDC1b
Chr.7
S9
UnR: 59.015.876-59.078.349_ 153 kb
YIPF2
JPH1B
BLVRA
PAXIP1
SSR2
TN13 (Z)
SACM1LA
PCMTD2
CBLC
ORC4
rTN13
S10
UnR: 6.369.588- 6.457.890_ 88 kb
CHST12
IL2RB
UBE2L
MPST_L
TN3 (P)
TN4 (P)
TN5 (P)
TN6 (P)
TN7 (P)
MIEF1
CTSD
ATF4B2
GIMAP
rTN3
UnR:41.023.085-41.722.140_ 700 kb
PIK3CA_L
HIST1H3
WNT11
COL21A1
TN9 (U)
TN10 (U)
TN11 (U)
COL11A2_L
DTD2
DTD2
rTN9
UnR: 46.396.389-46.534.092_ 137kb
GCC1
MCAM
PAQR6
KLHL
n.d.
TN12 (U)
MAP2K1
MLPH_L
NRXN1
DNAJB12
rTN12
UnR: 66.822.885-66.997.488_ 175 kb
NEU1
n.d.
PDK2
MICALL1
GGA1
TN14 (U)
PLXNA3
BCAP31
PRDM15
MRPL44
rTN14
UnR:74.578.864-74.714.482_ 145 kb
TLN1_L
TPM2
CRFA26
MPDZ_L
TN15 (U)
PMPCB
DNAJC2
PSMC2
SLC26A5
MECP2
rTN15
S11
UnR: 91.568.106- 91668.463_ 100 kb
RPL15
NKIRAS1
UBE2E1
UBE2E1
SF3A3
TN16 (U)
TN17 (U)
TN18 (U)
TN19 (U)
TN20 (Uψ)
TN21 (U)
TN22 (U)
TN23 (U)
TN24 (U)
TN25 (U)
MANEAL
LIN28A
PDIK1L
rTN16
UnR:16.279.716-16.449.241_ 169 kb
RXRB
COL11A2
HSD17B8
BRD2
TAP2
PSMB9
PSMB9L
PSMB10
PSMB8
TN8 (U)
DAXX
ZBTB22
KIFC1
ZNF384
FLOT1
rTN8
MHCI frg.
(NNN region)

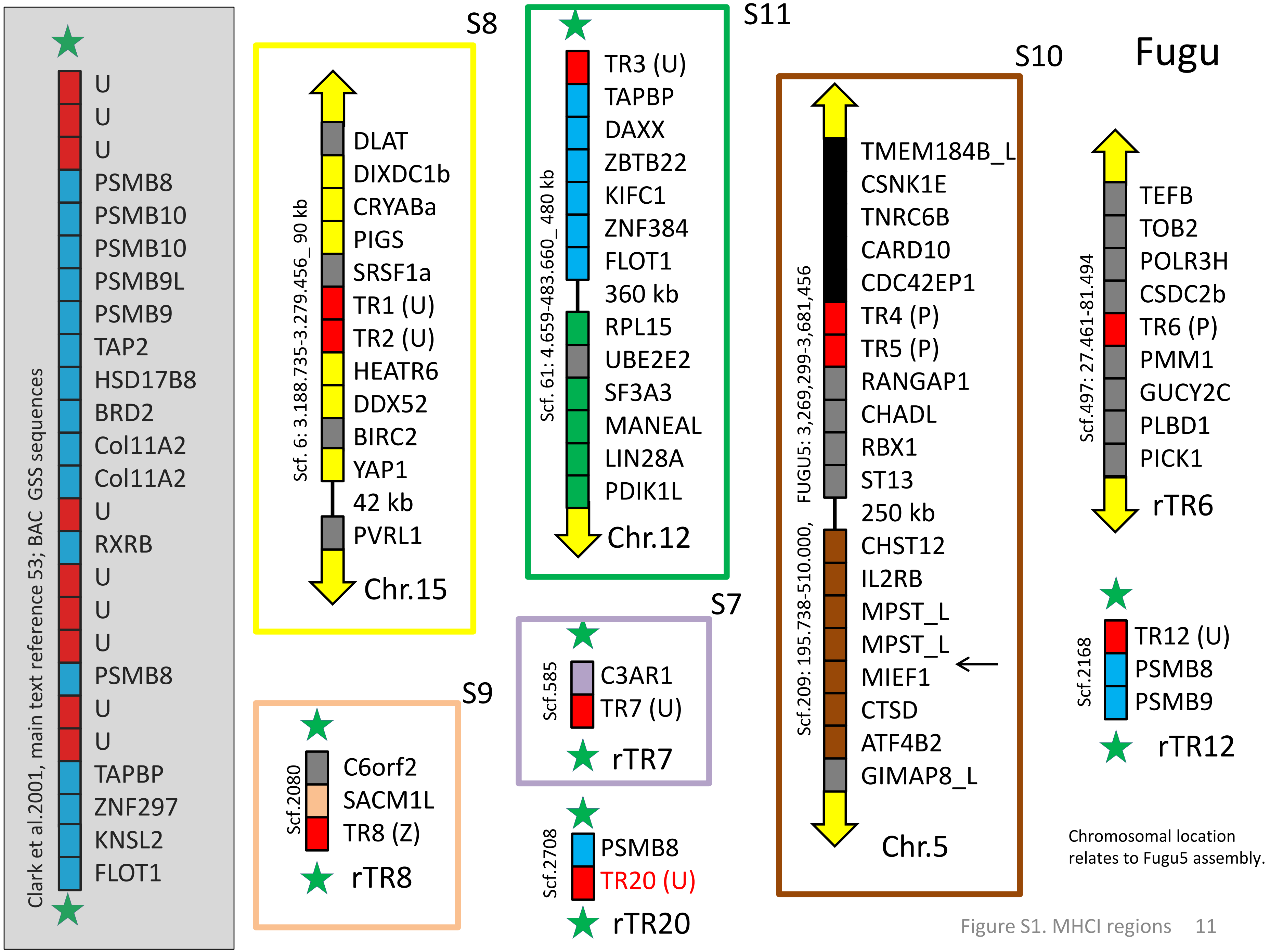

Figure S1. MHCI regions

Spotted gar

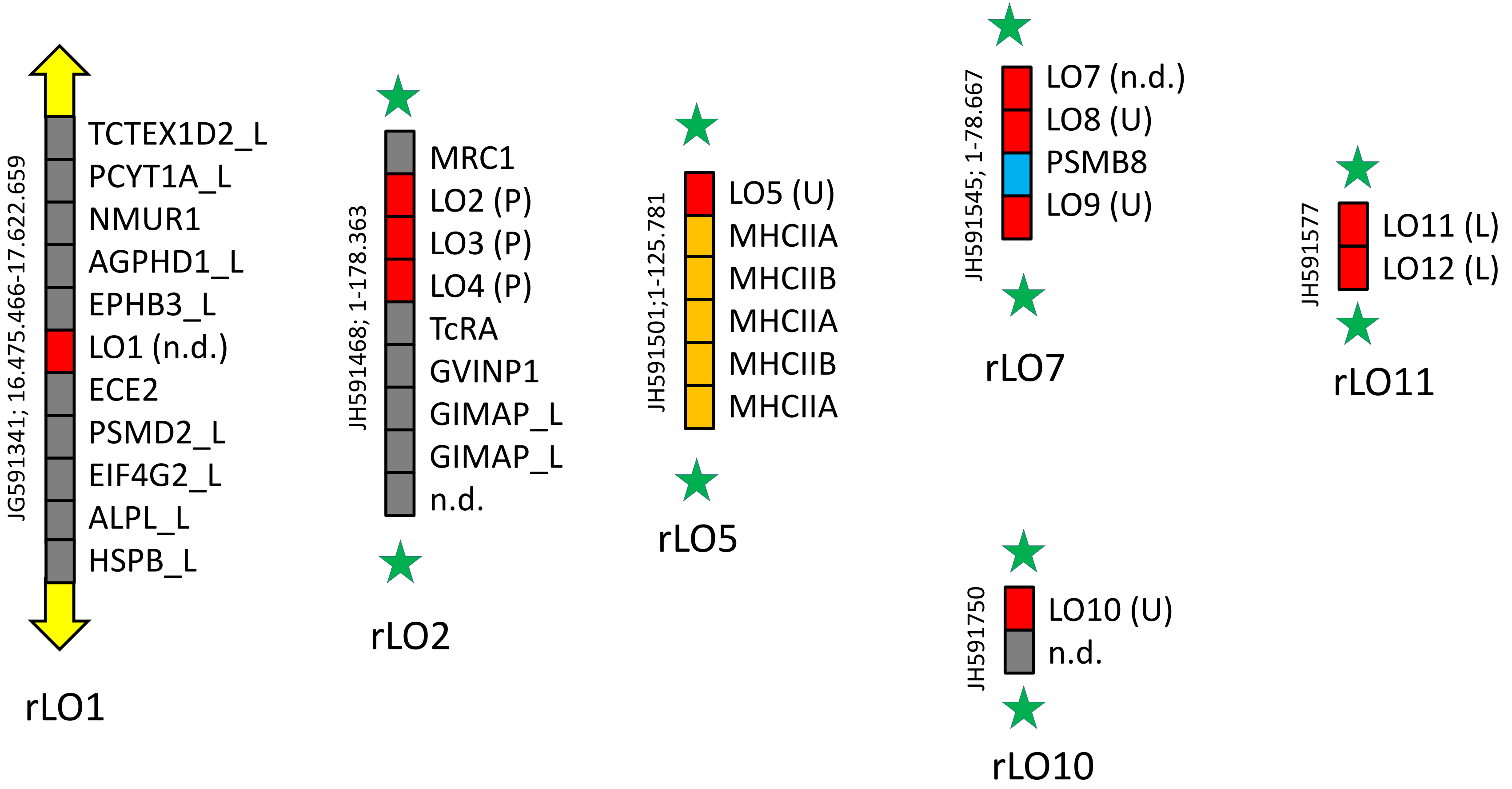

JG591341; 16.475.466-17.622.659
TCTEX1D2_L
PCYT1A_L
NMUR1
AGPHD1_L
EPHB3_L
LO1 (n.d.)
ECE2
PSMD2_L
EIF4G2_L
ALPL_L
HSPB_L
rLO1
JH591468; 1-178.363
MRC1
LO2 (P)
LO3 (P)
LO4 (P)
TcRA
GVINP1
GIMAP_L
GIMAP_L
n.d.
rLO2
JH591501;1-125.781
LO5 (U)
MHCIIA
MHCIIB
MHCIIA
MHCIIB
MHCIIA
rLO5
JH591545; 1-78.667
LO7 (n.d.)
LO8 (U)
PSMB8
LO9 (U)
rLO7
JH591750
LO10 (U)
n.d.
rLO10
JH591577
LO11 (L)
LO12 (L)
rLO11
