# Supplementary material for: A comprehensive analysis of teleost MHC class I sequences
Source: BMC Evol Biol. 2015 Mar 6;15:32. doi: 10.1186/s12862-015-0309-1 (PMC4364491; doi:10.1186/s12862-015-0309-1)
Supplement: Additional file 2: Table S1. — MHCI gene ID, genomic location and EST match. [file 12862_2015_309_MOESM2_ESM.pdf]

Additional file 2: Table S1. MHCI gene ID, genomic location and EST match for nine ray-finned fishes

**Species**	**Page**
Cavefish (AM; *Astyanax mexicanus*) MHC class I sequences	2
Zebrafish (DR; *Danio rerio*) MHC class I sequences	3
Medaka (OL; *Oryzias latipes*) MHC class I sequences	4
Platyfish (XM; *Xiphophorus maculatus*) MHC class I sequences	5
Nile tilapia (ON; *Oreochromis niloticus*) MHC class I sequences	6
Stickleback (GA; *Gasterosteus aculeatus*) MHC class I sequences	8
Green pufferfish (TN; *Tetraodon nigroviridis*) MHC class I sequences	9
Japanese Pufferfish (TR; *Takifugu rubripes*) MHC class I sequences	10
Spotted gar (LO; *Lepisosteus oculatus)* MHC class I sequences	11

Legend to table S1. MHC class I lineage is defined in parenthesis after gene name where gene name represents the species and our given MHCI gene number within that species such as DR1 defining the first gene in *Danio rerio*. Ensembl gene ID in parenthesis reflects that multiple genes were identified within that region. Grey shading defines genes missing 5', 3' or internal parts of mature extracellular protein where some have typical pseudogene characteristics. Green shading defines small assumed tetraodon U-lineage gene fragment in $(NNN)_n$ region. Some Medaka genes have locus specific names provided by Nonaka et al., [main text reference 13]. Blue shading for zebrafish sequences define U lineage genes published by McConnell et al., 2014 [main text reference 25] while zebrafish Z and L lineage genes have additional locus specific names according to Dirscherl et al.,2014 [main text reference 38]. Abbreviations are as follows: Id= amino acid identity, cov.= coverage, EST= expressed sequence tag, LG= linkage group, ORF= open reading frame, n.m.= no match. # = Number of ESTs available in NCBI EST Databases 10.04.2014. Red font= bona fide gene sequence with EST match above 92% aa identity in alpha 3 domain shown with GenBank accession number. When this EST approach was negative, expressed match to the entire open reading frame was sought in GenBank nucleotide resources (cDNA) and subsequently available SRA/TSA resources if the nucleotide search was negative. Analysed SRA/ TSA accession numbers can be found in the main manuscript Material and Methods section. When sequence identity is too high between loci to match ESTs correctly, the match is shown in parenthesis.

Species/ Gene Name	Ensembl gene ID	Genomic location Ensembl	EST match (Genbank)
**Cavefish (*Astyanax mexicanus*) n=42/21 bona fide**			**ESTs=190.196, NT= 11.248**
AM1 (U)	ENSAMXG00000004456	KB871756.1:731.095-739.372	(378 aa) <38%ID 95% cov.
AM2 (Z2)	ENSAMXG00000020385	KB871871.1:534.379-556.328	(321 aa) 1 EST 100% ID 100% cov.FO227761
AM3 (Z2)	ENSAMXG00000020387	KB871871.1:577.693-587.920	(329 aa) <64%ID 100% cov.
AM4 (Z2)	ENSAMXG00000020391	KB871871.1:596.826-603.393	(353 aa) <75%ID 100% cov. but SRA match
AM5 (P)	No Ensembl ID	KB871893.1:99.729-104.236	(284 aa) <23%ID 65% cov., but SRA match
AM6 (P)	ENSAMXG00000005391	KB871893.1:114.151-120.095	(257 aa) 3' missing
AM7 (Z1)	ENSAMXG00000005172	KB872010.1:338.894-344.058	(300 aa) 5' missing <100%ID 100% cov.
AM8 (Z1)	ENSAMXG00000005186	KB872010.1:368.671-398.678	(322 aa) <57%ID 100% cov. but SRA match
AM9 (Z2 ψ)	ENSAMXG00000005205	KB872010.1: 401,043-407,580	(172 aa) Lacks a1a2 domains
AM10 (Z2)	ENSAMXG00000005212	KB872010.1:408.806-417.593	(337 aa) <50%ID 95% cov.
AM11 (Z1)	ENSAMXG00000005228	KB872010.1:440.801-442.117	(143 aa) 5'3' missing
AM12 (Lψ)	ENSAMXG00000020806	KB872367.1:53,356-56,275	(188 aa) lacks a2 and 3' , Pseudogene
AM13 (U)	ENSAMXG00000012137	KB872374.1:21-6.096	(354 aa) <39%ID 92% cov.
AM14 (U)	ENSAMXG00000012141	KB872374.1:13.042-14.417	(134 aa) Lacks a1 domain, 3' missing
AM15 (U)	ENSAMXG00000012146	KB872374.1: 19.497-33.140	(234 aa) Lacks 5'3', pseudogene
AM16 (U)	ENSAMXG00000012154	KB872374.1:68.347-77.177	(231 aa) 3' missing
AM17 (U)	ENSAMXG00000012163	KB872374.1: 90.215-99.292	(128 aa) Lacks 5'3'
AM18 (Z1)	ENSAMXG00000005091	KB872702.1:16.324-18.628	(348 aa) <73%ID 100% cov.
AM19 (Z1)	ENSAMXG00000005129	KB872702.1:26.615-47.799	(218 aa) 3' missing, but SRA match
AM20 (U)	ENSAMXG00000005824	KB872935.1:26.693-36.705	(193 aa) 3' missing
AM21 (Z1)	ENSAMXG00000015427	KB873225.1:8.381-9.023	(185 aa) 3' missing
AM22 (Z2)	ENSAMXG00000019749	KB873312.1:6.494-10.392	(336 aa) <83%ID 100% cov.
AM23 (Z1)	ENSAMXG00000004123	KB873343.1:1.019-15.294	(332 aa) <60%ID 100% cov.
AM24 (Z1)	ENSAMXG00000008310	KB873443.1:82-1,518	(233 aa) 5' missing <69%ID 100% cov.
AM25 (Z2)	ENSAMXG00000004532	KB873495.1:347-10,940	(349 aa) <79%ID 100% cov.
AM26 (Z2)	ENSAMXG00000019161	KB873598.1:6,446-7,932	(205 aa) 5' missing <82%ID 100% cov.

AM27 (U)	ENSAMXG00000012522	KB873642.1:4,536-14,534	(238 aa) 3' missing
AM28 (Z1)	ENSAMXG00000010423	KB873648.1:4,727-10,381	(281 aa) 5' missing <75%ID 100% cov.
AM29 (Z1)	ENSAMXG00000001165	KB874195.1:2-2,918	(261 aa) 5' missing <67%ID 100% cov.
AM30 (U)	ENSAMXG00000009348	KB874440.1:2,653-7,379	(218 aa) 3' missing
AM31 (U)	ENSAMXG00000013144	KB881373.1:21-1,063	(169 aa) Lacks a1 domain, 3' missing
AM32 (L ψ)	No Ensembl ID	KB882095: 3,016,286-3,019,577	(98 aa) pseudogene
AM33 (S)	ENSAMXG00000017444	KB882192.1:1.855.138-1.867.212	(338 aa) <37%ID 100% cov. but SRA match
AM34 (S)	ENSAMXG00000017459	KB882192.1:1.870.383-1.874.078	(323 aa) <38%ID 93% cov. but SRA match
AM35 (U)	ENSAMXG00000006709	KB882234.1:282.644-295.597	(268 aa) 3' missing
AM36 (U)	ENSAMXG00000006716	KB882234.1:294.383-308.558	(279 aa) 3' missing
AM37 (U)	ENSAMXG00000006739	KB882234.1:321.252-330.732	(326 aa) <31%ID 82% cov.
AM38 (S)	ENSAMXG00000003502	KB882301.1:1.316.715-1.326.631	(334 aa) <40%ID 100% cov., but SRA match
AM39 (S)	ENSAMXG00000003509	KB882301.1:1.336.985-1.396.062	(327 aa) <41%ID 100% cov.
AM40 (S)	ENSAMXG00000003518	KB882301.1:1.343.262-1.346.695	(199 aa) 3' missing, but SRA match
AM41 (S)	ENSAMXG00000003529	KB882301.1:1.350.690-1.351.925	(272 aa) <38%ID 100% cov.
AM42 (S)	ENSAMXG00000003539	KB882301.1:1.398.925-1.401.916	(308 aa) <39%ID 100% cov., but SRA match
**Zebrafish**	**(*Danio rerio*) n=30/27 bona fide**		**# ESTs=1.773.542, # Nucleotides= 128,922**
DR1 (ZEA)	ENSDARG00000001470	Chr.1: 47,613,228-47,619,236	(350 aa) 2 ESTs 10% ID 100% cov. eg.EH570470
DR2 (ZDA)	ENSDARG00000086877	Chr.1: 47,626,631-47,632,322	(393 aa) <95%ID 98% cov. but KC607845
DR3 (ZCA)	ENSDARG00000069471	Chr.1: 47,637,895-47,648,897	(364 aa) <100%ID 100% cov. but KC607835.1
DR4 (ZBA)	ENSDARG00000036588	Chr.1: 47,652,531-47,659,103	(398 aa) 4 ESTs 100%ID 100% cov. eg. CK018140
DR5 (ZFA)	ENSDARG00000088022	Chr.3: 502,131-512,626	(371 aa) <74% ID 97% cov. but KC607855
DR6 (ZGAψ)	(ENSDARG00000092162)	Chr.3: 1,070,329-1,076,000	(284 aa) pseudogene
DR7 (ZHAψ?)	(ENSDARG00000092162)	Chr.3:1,076,000-1,080,000	(280 aa) 1 EST 100%ID 100% cov. EB942259, pseudogene?
DR8 (ZIA)	(ENSDARG00000092162)	Chr.3:1,083,984-1,093,108	(311 aa) <93%ID 96% cov.
DR9 (ZJA)	ENSDARG00000074765	Chr.3: 1,109,672-1,128,564	(383 aa) <99%ID 100% cov. but KC607868
DR10 (LCA)	ENSDARG00000055813	Chr.3: 23,762,869-23,765,495	(350 aa) <65% ID 100% cov.
DR11 (LBA)	ENSDARG00000016227	Chr.8: 47,093,433-47,098,053	(355 aa) 1 EST 100% ID 100% cov. EH473102
DR12 (LAA)	ENSDARG00000016056	Chr.8: 47,110,756-47,116,918	(359 aa) 4 ESTs 100% ID 100% cov. eg. EH558607

(UDA)	n.m.	Chr.19:7,633,924-8,728,374	1 EST 100% ID 100% cov. DV586777.1
(UEA)	n.m.	Chr.19:7,633,924-8,728,374	6 ESTs 100% ID 100% cov. eg. GW711669
(UFA)	n.m.	Chr.19:7,633,924-8,728,374	5 ESTs 100% ID 100% cov. eg. EB987672
(UIA)	n.m.	Chr.19:7,633,924-8,728,374	1 EST 100% ID 100% cov. CO813803
(UJA)	n.m.	Chr.19:7,633,924-8,728,374	17 ESTs 100% ID 100% cov. eg. CD285014
DR13 (UCA)	ENSDARG00000092731	Chr.19: 7,667,569-7,673,062	(353 aa) 1 EST 100% ID 100% cov. CN322833
DR14 (UBA)	ENSDARG00000075963	Chr.19: 7,695,390-7,723,636	(348 aa) 24 ESTs 100% ID 100% cov. eg. BQ450582
DR15 (U)	ENSDARG00000039164	Chr.22: 5,239,117-5,243,568	(342 aa) <71%ID 100% cov.
DR16 (U)	ENSDARG00000059039	Chr.22: 5,243,817-5,258,961	(344 aa) 2 ESTs 100% ID 100% cov. eg. EH477854
DR17 (LMA)	ENSDARG00000086127	Chr.25: 11,063,589-11,066,365	(328 aa) <91%ID 97% cov.
DR18 (LOA)	ENSDARG00000087161	Chr.25: 11,071,636-11,074,578	(342 aa) <82%ID 97% cov.
DR19 (LHA)	ENSDARG00000046057	Chr.25: 11,082,589-11,085,195	(326 aa) <98%ID 100% cov.
DR20 (LPA)	ENSDARG00000051710	Chr.25: 11,250,338-11,263,782	(321 aa) 1 EST 100% ID 100% cov. DR717949
DR21 (LNA ψ)	No Ensembl ID	Chr.25:11,276,832-11,277,543	(176 aa) 5'3' missing, pseudogene
DR22 (LJA)	ENSDARG00000096830	Chr.25:11,280,398-11,285,009	(357 aa) <89%ID 97% cov.
DR23 (LEA)	ENSDARG00000051711	Chr.25: 11,300,140-11,301,469	(321 aa) <91%ID 97% cov.
DR24 (LFA)	ENSDARG00000051712	Chr.25: 11,314,469-11,317,472	(348 aa) <83%ID 100% cov.
DR25 (LDA)	ENSDARG00000023203	Chr.25: 11,323,244-11,327,705	(329 aa) <90%ID 79% cov.
DR26 (LLA ψ)	ENSDARG00000096977	Chr.25:11,338,870-11,351,127	(308 aa) orf error, pseudogene
DR27 (LKA)	ENSDARG00000096940	Chr.25:11,354,781-11,367,096	(346 aa) <95%ID 85% cov.
DR28 (LGA)	ENSDARG00000051713	Chr.25: 11,371,003-11,372,257	(328 aa) <91%ID 100% cov.
DR29 (LIA)	ENSDARG00000097766	Chr.25:11,375,070-11,380,737	(343 aa) 2 ESTs 100% ID 100% cov.eg.EH478662.1
DR30 (ZKA)	ENSDARG00000076734	Scf.Zv9_NA257: 89,256-94,080	(374 aa) <93%ID 100% cov. but KC607870
**Medaka (*Oryzias latipes*) n=19 /14 bona fide**			#ESTs= 668.016, # Nucleotides= 78,749
OL1 (UHA)	ENSORLG00000017153	Chr.8: 24.973.752-24.985.505	(349 aa) <86% ID 100% cov. but AB604113
OL2 (UIA ψ)	ENSORLG00000000970	Chr.11: 1.350.825-1.351.562	(165 aa) 5' missing_pseudogene
OL3 (UIA)	ENSORLG00000000983	Chr.11: 1.378.045-1.380.276	(332 aa) 5'missing <70% ID 97% cov.
OL4 (Z)	ENSORLG00000001044	Chr.11: 1.867.734-1.871.932	(335 aa) <59%ID 86% cov.
OL5 (Z)	ENSORLG00000001056	Chr.11: 1.873.262-1.877.310	(324 aa) <59%ID 86% cov. but SRX377644 match

OL6 (Z ψ)	ENSORLG00000001058	Chr.11: 1.943.702-1.945.742	(172 aa) 3’ missing_pseudogene
OL7 (Z)	ENSORLG00000001067	Chr.11: 1.971.417-1.974.810	(344 aa) 1 EST 100% ID 86% cov. DC269633
OL8 (UEA)	ENSORLG00000006435	Chr.11: 15.112.547-15.116.458	(387 aa) <61% ID 100% cov. but AB183488
OL9 (UDA)	ENSORLG00000006575	Chr.11: 15.263.065-15.277.733	(341 aa) <71% ID 97% cov. but AB450998
OL10 (UAA)	ENSORLG00000006772	Chr.11: 15.359.941-15.374.285	(353 aa) 7 ESTs 100% ID 81% cov. eg.BJ902860
OL11 (UBA)	ENSORLG00000006798	Chr.11: 15.417.434-15.446.882	(360 aa) <85%ID 100% cov. but AB183488
OL12 (UCA ψ)	No Ensembl ID	Chr.11:15,412,645-15,420,668	(302 aa) 5' missing, pseudogene
OL13 (UCA)	ENSORLG00000012414	Chr.22: 5.541.178-5.547.621	(355 aa) <65%ID 97% cov. but AB033381
OL14 (UIA ψ)	ENSORLG00000019579	Scaffold721: 105.268-108.455	(339 aa) a2 orf error, pseudogene?
OL15 (UIA1)	ENSORLG00000020349	Scaffold1100: 46.629-48.948	(331 aa) <71% ID 97% cov. but AB604117.1
OL16 (U ψ)	ENSORLG00000020236	Scaffold1612: 6.403-9.764	(262 aa) 5’ missing_pseudogene
OL17 (Z)	ENSORLG00000020395	Scaffold1641: 9.205-13.258	(322 aa) <66%ID 86% cov.
OL18 (UIA2)	ENSORLG00000018729	Scaffold3269: 4.511-6.197	(372 aa) <71% ID 97% cov. but AB604119.1
OL19 (UGA)	No Ensembl match	Genbank accession #BAK26816.1	(359 aa) <89% ID 81% cov. but AB604102
**Platyfish (*Xiphophorus maculatus*) n=22 /7 bona fide**			**# ESTs=9.441, # Nucleotides=5.059**
XM1 (U)	(ENSXMAG00000016854)	JH556745.1:1,781,732- 1,783,290	(282 aa) 5’missing, <43%ID 35% cov.
XM2 (U)	(ENSXMAG00000016854)	JH556745.1: 1,835,219-1,836,654	(284 aa) <36%ID 20% cov.
XM3 (U)	(ENSXMAG00000016857)	JH556745.1: 1,848,144 -1,849,911	(283 aa) <50%ID 14% cov.
XM4 (U Ψ)	(ENSXMAG00000016857)	JH556745.1 1,854,049-1,854,360	(183 aa), orf error, pseudogene
XM5 (Z)	ENSXMAG00000001537	JH556782.1: 1.261.763-1.263.624	(262 aa) <28%ID 40% cov. but SRR073430.265648.2
XM6 (U)	ENSXMAG00000014268	JH556877.1: 584.294-586.879	(115 aa) 3’ missing
XM7 (U)	ENSXMAG00000014269	JH556877.1: 622.448-624.584	(196 aa) 3’ missing
XM8 (U)	ENSXMAG00000014271	JH556877.1: 626.861-631.044	(338 aa) <32%ID 26% cov.
XM9 (U)	(ENSXMAG00000014273)	JH556877:651.665-655.118	(255 aa) missing internal and 3' seq.
XM10 (U)	(ENSXMAG00000014273)	JH556877:658.701-668.042	(338 aa) <30%ID 28% cov.
XM11 (U)	(ENSXMAG00000014273)	JH556877:668.437-671.686	(300 aa) missing internal seq., <40%ID 33% cov.
XM12 (U)	ENSXMAG00000014655	JH556877.1: 860.225-876.295	(166 aa) 3' missing
XM13 (U)	ENSXMAG00000014656	JH556877.1: 898.266-902.441	(119 aa) 3’ missing
XM14 (U)	ENSXMAG00000014659	JH556877.1: 911.478-927.700	(249 aa) 5' missing, <28%ID 52% cov.

XM15 (U)	ENSXMAG00000005036	JH558542.1: 21-4.011	(271 aa) 3' missing
XM16 (U)	ENSXMAG00000009441	JH558870.1: 2.136-4.694	(175 aa) 3’ missing
XM17 (Z)	ENSXMAG00000007599	JH559400.1: 2.276-4.121	(188 aa) 3’ missing
XM18 (U)	ENSXMAG00000016595	JH559413.1: 21-2.267	(196 aa) 3’ missing
XM19 (U)	ENSXMAG00000016596	JH559413.1:2.228-9.853	(325 aa) Corrected ORF, <54%ID 14% cov.
XM20 (U)	ENSXMAG00000000946	AGAJ01050710.1: 21-4.522	(337 aa) <32%ID 37% cov.
XM21 (U)	ENSXMAG00000014517	AGAJ01052725.1: 21-1.100	(160 aa) 3’ missing
XM22 (Z)	No Ensembl ID	AGAJ01058203.1:1-795	(95 aa) 5’+3’ missing
**Nile tilapia (*Oreochromis niloticus*) n=52 /36 bona fide**			**# ESTs =121.224, # Nucleotides=82.872**
ON1 (U)	ENSONIG00000007074	GL831254.1: 1.934.802-1.939.135	(344 aa) <70% ID 100% cov
ON2 (U)	ENSONIG00000007085	GL831254.1: 1.942.502-1.944.822	(318 aa) 3'error
ON3 (U)	ENSONIG00000007090	GL831254.1: 1.948.887-1.979.577	(366 aa) <72%ID 100% cov
ON4 (U)	ENSONIG00000007093	GL831254.1: 2.157.445-2.160.180	(337 aa) < 88% ID 82% cov
ON5 (U)	ENSONIG00000007094	GL831254.1: 2.171.187-2.174.216	(354 aa) < 85% ID 99% cov
ON6 (U)	ENSONIG00000007098	GL831254.1: 2.183.675-2.186.455	(331 aa) <78% ID 82% ocv
ON7 (U)	ENSONIG00000007101	GL831254.1: 2.265.696-2.268.042	(342 aa) <70% ID 82% cov.
ON8 (U)	ENSONIG00000007104	GL831254.1: 2.282.142-2.294.806	(366 aa) <84% ID 99% cov
ON9 (L)	ENSONIG00000001466	GL831385.1: 447.962-465.059	(336 aa) <32% ID but TSA match:GAID01031757.1
ON10 (U)	ENSONIG00000019989	GL831408.1: 1.437-4.505	(100 aa) 3’ missing
ON11 (U)	ENSONIG00000019990	GL831408.1: 25.538-26.924	(251 aa) 3' missing
ON12 (U)	ENSONIG00000019991	GL831408.1: 45.731-50.156	(354 aa) <88%ID 99% cov
ON13 (U)	ENSONIG00000019992	GL831408.1: 54.395-57.192	(328 aa) <84% ID 98% cov
ON14 (U)	ENSONIG00000019994	GL831408.1: 90.628-92.246	(318 aa) <84% ID 99% cov
ON15 (U)	ENSONIG00000019996	GL831408.1: 94.716-97.113	(267 aa) 5’ missing_<86% ID 100% cov.
ON16 (U)	ENSONIG00000019998	GL831408.1: 147.376-148.979	(320 aa) <87% ID 99% cov.
ON17 (U)	ENSONIG00000019999	GL831408.1: 190.452-192.562	(210 aa) 3’ missing
ON18 (U)	ENSONIG00000020001	GL831408.1: 380.634-381.888	(308 aa) <72% ID 97% cov.
ON19 (U)	ENSONIG00000020051	GL831408.1: 690.605-705.735	(353 aa) <81%ID 84% cov
ON20 (U)	ENSONIG00000020058	GL831408.1: 746.600-760.601	(344 aa) <72% ID 94% cov

ON21 (U)	ENSONIG00000020061	GL831408.1: 820.845-825.121	(306 aa) <91 % ID 83% cov.
ON22 (Z)	ENSONIG00000018185	GL831434.1: 429.549-435.583	(345 aa) <99%ID 79% cov. but GR664657
ON23 (Z)	ENSONIG00000005063	GL831484.1: 36.038-44.524	(351 aa) <75% ID 91% cov
ON24 (Z)	ENSONIG00000005069	GL831484.1: 61.315-63.813	(333 aa) <66% ID 91% cov
ON25 (U)	ENSONIG00000003532	GL831521.1: 20.492-21.483	(249 aa) 5’ 3' missing
ON26 (U)	ENSONIG00000003533	GL831521.1: 29.315-34.240	(360 aa) <81 % ID 99% cov
ON27 (U)	ENSONIG00000003537	GL831521.1: 71.123-73.681	(359 aa) <100%ID 100% cov.
ON28 (U)	ENSONIG00000003541	GL831521.1: 150,998-186.211	(348 aa) <81% ID 99% cov.
ON29 (U)	ENSONIG00000003542	GL831521.1: 195.472-207.579	(344 aa) <80% ID 98% cov.
ON30 (U)	ENSONIG00000003547	GL831521.1: 263.545-270.469	(334 aa9 <82% ID 99% cov.
ON31 (U)	ENSONIG00000003548	GL831521.1: 355.060-357.370	(325 aa) <88% ID 100% cov.
ON32 (U)	ENSONIG00000004918	GL831531.1: 134.793-150.127	(355 aa) <67% ID 99% cov.
ON33 (Z)	ENSONIG00000017388	GL831555.1: 88.057-89.960	(260 aa) 3' missing
ON34 (Z)	ENSONIG00000017389	GL831555.1: 218.140-223.162	(259 aa) 3' missing
ON35 (Z)	ENSONIG00000017390	GL831555.1: 264.632-277.102	(403 aa) <73%ID 91% cov.
ON36 (U)	ENSONIG00000008406	GL831594.1: 94.645-96.811	(188 aa) 5’ missing, <79%ID 100% cov.
ON37 (U)	ENSONIG00000008407	GL831594.1: 105.890-108.506	(305 aa) <79% ID 99% cov.
ON38 (U)	ENSONIG00000008409	GL831594.1: 143.390-148.282	(305 aa) <67% ID 99% cov.
ON39 (U)	ENSONIG00000008413	GL831594.1: 162.192-169.616	(351 aa) <92%ID 84% cov.
ON40 (U)	No Ensembl ID	GL831856_1:8.763-10.036	(182 aa) 5’3’ missing
ON41 (U ψ)	No Ensembl ID	GL831856_1:10.398-11.153	(120 aa) 3’ missing, pseudogene
ON42 (U)	ENSONIG00000012499	GL831856.1: 43.354-51.626	(341 aa) <89% ID 97% cov.
ON43 (U)	ENSONIG00000012501	GL831856.1: 66.127-71.145	(304 aa) <82%ID 83% cov.
ON44 (U)	ENSONIG00000012502	GL831856.1: 76.815-85.004	(336 aa) <93% ID 84% cov.
ON45 (U)	ENSONIG00000015360	GL831938.1: 36.313-41.071	(312 aa) <78% ID 99% cov.
ON46 (U)	ENSONIG00000015362	GL831938.1: 49.095-52.875	(293 aa) <73% ID 82% cov.
ON47 (U ψ)	ENSONIG00000012482	GL831994.1:15.609- 23.582	(179 aa) 5’ 3' missing, orf error, pseudogene
ON48 (U)	No Ensembl ID	GL831994.1:26.035-26.301	(89 aa) 3’missing
ON49 (U)	ENSONIG00000012483	GL831994.1:27.876-28.142	(89 aa) 3’ missing,
ON50 (U)	ENSONIG00000012484	GL831994.1: 43.947-46.959	(246 aa) 5’ missing, <94%ID 84% cov.

ON51 (U)	ENSONIG00000011081	GL832190.1: 10.155-16.215	(326 aa) 5’ missing, <72%ID 82% cov.
ON52 (U)	ENSONIG00000018290	GL832444.1: 49-5.673	(355 aa) <77% ID 99% cov.
**Stickleback (*Gasterosteus aculeatus*) n=30 /22 bona fide**			# ESTs= 277.188, # Nucleotides=5,153
GA1 (U)	ENSGACG00000001837	Group X: 15.422-20.706	(369 aa) (1 EST shared with GA5+23, DN656534)
GA2 (U)	ENSGACG00000001910	Group X: 425.779-432.445	(370 aa) <98% ID 100% cov.
GA3 (U ψ)	No Ensembl ID	Group X: 460.641-468.080	(261 aa) ORF error, Pseudogene
GA4 (U)	ENSGACG00000001913	Group X: 481.025-492.757	(374 aa) (6 ESTs shared with GA8,17, 27 eg.DN658147)
GA5 (U)	ENSGACG00000001919	Group X: 520.053-527.028	(341 aa) (1 EST shared with GA1+23, DN656534)
GA6 (U)	ENSGACG00000001932	Group X: 662.965-665.020	(245 aa) Lacks 5’; 1 EST 100% ID, DT956491
GA7 (U ψ)	No Ensembl ID	Group X:678.166-682.178	(93 aa) A2 only, Pseudogene
GA8 (U)	ENSGACG00000001935	Group X: 779.138-791.924	(316 aa) (6 ESTs shared with GA8,17, 27)
GA9 (U)	ENSGACG00000001937	Group X: 802.046-805.613	(347 aa) 7 ESTs 100% ID eg.DW035296
GA10 (U)	No Ensembl ID	Group X:849.550-861.373	(295 aa) Lacks A1 domain, <100% ID 100% cov.
GA11 (U)	ENSGACG00000001941	Group X: 891.779-894.040	(313 aa) <100% ID 100% cov.
GA12 (U)	No Ensembl ID	Group X:916.154-926.899	(378 aa) <99% ID 100% cov.
GA13 (U)	ENSGACG00000001973	Group X:1.058.136-1.070.262	(359 aa) 1 EST 100% ID 100% cov. DN677624
GA14 (U)	ENSGACG00000001976	Group X: 1.090.323-1.093.673	(350 aa) <99% ID 100% cov.
GA15 (U)	No Ensembl ID	Group X:1.096.590-1.102.571	(304 aa) 2 ESTs 100% ID DW605527, DW039424
GA16 (U)	ENSGACG00000001978	Group X: 1.119.796-1.124.476	(320 aa) <100% ID 100% cov.
GA17 (U)	ENSGACG00000001979	Group X: 1.144.188-1.151.431	(347 aa) (6 ESTs shared with GA8,17, 27, DN658147)
GA18 (Z)	ENSGACG00000009731	Group X: 14.983.618-14.987.400	(368 aa) <94%ID 98% cov. but DT950082
GA19 (U)	ENSGACG00000000116	Scaffold_58: 785.995-791.571	(340 aa) <99% ID 100% cov.
GA20 (U)	(ENSGACG00000000122)	Scaffold_58:823.228-835.972	(343 aa) (2 ESTs shared with GA21+30 eg. DW600052)
GA21 (U)	(ENSGACG00000000122)	Scaffold_58:865.091-877.650	(345 aa) (2 ESTs shared with GA20+30 eg DW600052)
GA22 (U)	(ENSGACG00000000122)	Scaffold_58:893.246-902.081	(320 aa) <100% ID 100% cov.
GA23 (U)	(ENSGACG00000000122)	Scaffold_58: 949.938-954.473	(358 aa) 1 EST shared with GA1+5, DN656534
GA24 (U)	ENSGACG00000000141	Scaffold_58: 996.326-1.001.749	(363 aa) <100% ID 100% cov.
GA25 (U)	No Ensembl ID	Scaffold_58:1.034.716-1.038.858	(117 aa) Lacks 3’,
GA26 (U)	ENSGACG00000000151	Scaffold_58:1,078,466-1,084,416	(342 aa) <99% ID 100% cov.

GA27 (U)	ENSGACG00000001288	Scaffold_452: 3.492-8.260	(345 aa) internal NNN region; (6 ESTs shared with GA8,17, 27)
GA28 (U)	No Ensembl ID	Scaffold_653:7.716-9.769	(116 aa) Lacks 3'
GA29 (U ψ)	ENSGACG00000000124	Scaffold_854:596-5.501	(357 aa), orf error, pseudogene
GA30 (U)	ENSGACG00000002179	Scaffold_1223: 3.225-5.871	(270 aa) Lacks 5'; 2 ESTs shared with GA20+21
**Green pufferfish (*Tetraodon nigroviridis*) n=25 /7 bona fide**			#ESTs=9,748, #Nucleotides (NT) = 108.803
TN1 (U)	ENSTNIG00000009992	Chr.7: 4.474.277-4.475.964	(323 aa) <98%ID 100% cov.
TN2 (U)	No Ensembl ID	Chr.7:4.476.384-4.479.173	(208 aa) ORF error, <96%ID 100% cov.
TN3 (P)	No Ensembl ID	Un_R: 6.397.631- 6.399.093	(303 aa) <27%ID 90% cov.
TN4 (P)	No Ensembl ID	Un_R: 6.406,602-6.407,214	(163 aa) Lacks 5'3'
TN5 (P)	No Ensembl ID	Un_R: 6.412,622-6,415,839	(324 aa) <30% ID 81% cov.
TN6 (P ψ)	No Ensembl ID	Un_R: 6,420,283-6,420,651	(88 aa) pseudogene
TN7 (P)	No Ensembl ID	Un_R: 6,421,741-6,422,362	(166 aa) lacks 3'
TN8 (U)	No Ensembl ID	Un_R:16,359,958-16,359,902	(19 aa_ NNN region MHCI region)
TN9 (U)	ENSTNIG00000003024	Un_R: 41.327.814-41.329.045	(251 aa) Lacks 3'_ < 83%ID 100% cov.
TN10 (U)	No Ensembl ID	Un_R:41.339.884-41.345.128	(180 aa) Lacks 5', < 81% ID 100% cov.
TN11 (U)	No Ensembl ID	Un_R:42.551.598-42.552.372	(166 aa) Lacks 3'
TN12 (U)	ENSTNIG00000001973	Un_R: 46.457.180-46.457.895	(194 aa) Lacks 5' <88%ID 100% cov.
TN13 (Z)	ENSTNIG00000003449	Un_R: 59.040.025-59.042.948	(333 aa) <87%ID 100% cov. but CR726246
TN14 (U)	ENSTNIG00000004145	Un_R: 66.895.563-66.896.295	(205 aa) Lacks 5'_<85%ID 100% cov.
TN15 (U ψ)	ENSTNIG00000004925	Un_R: 74.651.122-74.651.415	(89 aa) Lacks 3'
TN16 (U)	ENSTNIG00000001016	Un_R: 91.605.519-91.607.947	(307 aa) Lacks 5' <79% ID 96% cov.
TN17 (U)	No Ensembl ID	Un_R:91. 91.609.131-610.586	(173 aa) Lacks 3'
TN18 (U)	(ENSTNIG00000005106)	Un_R:91,614,129-91,615,547	(238 aa) Lacks 5', < 88% ID 100% cov.
TN19 (U)	(ENSTNIG00000005106)	Un_R:91,620,258-91,620,889	(162 aa) Lacks 5', <85% ID 100% cov.
TN20 (U ψ)	(ENSTNIG00000005106)	Un_R:91,622,837-91,625,870	(345 aa) ORF error, pseudogene
TN21 (U)	(ENSTNIG00000005106)	Un_R:91,632,905-91,635,261	(284 aa; Lacks 5', <87%ID 100% cov.
TN22 (U)	No Ensembl ID	Un_R:91,638,301-91,640,135	(347 aa), <87% ID 100% cov.
TN23 (U)	ENSTNIG00000001085	Un_R: 91.641.494-91.644.322	(340 aa) <89% ID 100% cov.
TN24 (U)	ENSTNIG00000000474	Un_R: 91.647.017-91.647.905	(222 aa) Lacks 5'_<87%ID 100% cov.

TN25 (U)	No Ensembl ID	Un_R:91,656,239-91,657,762	(285 aa) Lacks 5', <88% ID 100% cov.
**Pufferfish (*Takifugu rubripes*) n=34/ 16 bona fide**			#ESTs=27,914, # Nucleotides=39,062
TR1 (U)	ENSTRUG00000017062	Scaffold 6: 3,198,531-3,200,246	(333 aa) <68%ID 98% cov.
TR2 (U)	ENSTRUG00000017063	Scaffold 6: 3,205,239-3,210,655	(321 aa) <68%ID 98% cov.
TR3 (U)	ENSTRUG00000002296	Scaffold 61:4,244-8,738	(342 aa) <97%ID 100% cov.
TR4 (P)	No Ensembl ID	Scaffold 209: 508,917-510,010	(206 aa) 5'+3' missing
TR5 (P)	No Ensembl ID	Scaffold 209: 516,289-517,881	(198 aa) internal parts missing
TR6 (P)	No Ensembl ID	Scaffold 497:44,782-49,340	(321 aa) <33% ID 60% cov.
TR7 (U)	ENSTRUG00000003539	Scaffold 585: 69,748-72,643	(340 aa) <100%ID 100% cov.
TR8 (Z)	ENSTRUG00000004298	Scaffold 2080:23,720-25,204	(219 aa) 5' missing, <100%ID 90% cov.
TR9 (P)	No Ensembl ID	Scaffold 2108:8,529– 12,205	(308 aa) <33 % ID 60% cov.
TR10 (P ψ)	No Ensembl ID	Scaffold 2108:15,381-16,480	(294 aa) pseudogene, ORF error
TR11 (P)	No Ensembl ID	Scaffold 2108:22,382-22,702	(87 aa) 5' missing
TR12 (U)	ENSTRUG00000001978	Scaffold 2168: 9,134-10,367	(111 aa) 5' missing
TR13 (U)	ENSTRUG00000002529	Scaffold 2183:6,228-13,379	(362 aa) <94%ID 100% cov.
TR14 (P)	No Ensembl ID	Scaffold 2190:6,635-8,502	(337 aa) <33% ID 59% cov. but TSA match
TR15 (P)	No Ensembl ID	Scaffold 2190:12,606–15,159	(258 aa) 3' missing <33% ID 75% cov.
TR16 (P)	No Ensembl ID	Scaffold 2440:1,665–4,373	(269 aa) <33% ID 68% cov.
TR17 (P)	No Ensembl ID	Scaffold 2627:1,321-3,402	(182 aa) 3' missing
TR18 (P)	No Ensembl ID	Scaffold 2628:2,060-8,810	(313 aa) <33% ID 59% cov.
TR19 (U)	ENSTRUG00000002033	Scaffold 2693: 10,282-11,984	(203 aa) 5' missing; <100% ID 100% cov.
TR20 (U)	ENSTRUG00000000486	Scaffold 2708: 7,649-9,458	(270 aa) 1 EST 100% ID 100% cov. CK829467
TR21 (Z)	ENSTRUG00000003955	Scaffold 3060:3,567-5,990	(320 aa) <95%ID 90% cov. but CA590706
TR22 (P)	No Ensembl ID	Scaffold 3394:4,970-7,539	(323 aa) <31% ID 70% cov.
TR23 (P)	No Ensembl ID	Scaffold 3485: 6,558-6,833	(86 aa) 5' missing; <33% ID 66% cov.
TR24 (P)	No Ensembl ID	Scaffold 3928:258-976	(166 aa) 3' missing
TR25 (P)	No Ensembl ID	Scaffold 3975:600-7,309	(151 aa) 5' missing; <34% ID 55% cov.
TR26 (P)	No Ensembl ID	Scaffold 4784:5,138-6,243	(253 aa) <33% ID 65% cov.
TR27 (P)	No Ensembl ID	Scaffold 5016:1,270-3,336	(166 aa) 3' missing

TR28 (P)	No Ensembl ID	Scaffold 7638:1,280-3,354	(182 aa) 3’ missing
TR29 (P)	No Ensembl ID	Scaffold 7656:92–2,346	(337 aa) <33% ID 59% cov.
TR30 (P)	No Ensembl ID	Scaffold 8439:1,979-3,842	(158 aa) 3’ missing
TR31 (P)	No Ensembl ID	Scaffold 8559:3-1,449	(201 aa) 5’ missing; <31% ID 70% cov.
TR32 (P)	No Ensembl ID	Scaffold 9266:50–2,297	(182 aa) 3’ missing
TR33 (P)	No Ensembl ID	Scaffold:9833:1,141-2,380	(138 aa) 5' missing, 33%ID 59% cov.
TR34 (P)	No Ensembl ID	Scaffold 9991:1–1,073	(243 aa) <33% ID 65% cov.
**Spotted gar (*Lepisosteus oculatus*) N=13/ 8 bona fide**			#ESTs= 0, # Nucleotides=62,071
LO1 (n.d.)	No Ensembl ID	JH591341.1:1.421.886-1.426.233	(324 aa) n.m.
LO2 (P)	No Ensembl ID	JH591468.1:12.330-13,136	(184 aa) 3’ missing
LO3 (P)	No Ensembl ID	JH591468.1:29,332-29,660	(112 aa) 3’ missing
LO4 (P)	No Ensembl ID	JH591468.1:44.285-45,296	(219 aa) 3' missing
LO5 (U)	No Ensembl ID	JH591501.1: 9.921-24.254	(330 aa) n.m.
LO6 (P)	No Ensembl ID	JH591523.1:90.771-91.013	(81 aa) 3’ missing
LO7 (n.d.)	No Ensembl ID	JH591545.1: 5.102-9.058	(374 aa) n.m.
LO8 (U)	No Ensembl ID	JH591545.1: 10.915-12.600	(99 aa) 5’ missing
LO9 (U)	ENSLACP00000013939	JH591545.1: 58,488-73,129	(185 aa) 3’ missing
LO10 (U)	No Ensembl ID	JH591750.1: 302-10.078	(276 aa) A2 domain missing (NNN region)
LO11 (L)	No Ensembl ID	JH591577.1: 7.422-11.660	(381 aa) n.m.
LO12 (L)	No Ensembl ID	JH591577.1: 51.693-56.541	(380 aa) n.m.
LO13 (L)	No Ensembl ID	AHAT01044524:117-1.888	(209 aa) 3’ missing
LO14 (Z)	No Ensembl ID	Unknown location	Z lineage alpha 1 and alpha domain, Assembled from genomic SRA reads
