# Supplementary material for: A comprehensive analysis of teleost MHC class I sequences
Source: BMC Evol Biol. 2015 Mar 6;15:32. doi: 10.1186/s12862-015-0309-1 (PMC4364491; doi:10.1186/s12862-015-0309-1)
Supplement: Additional file 3: — Text S1. Additional Atlantic salmon data. [file 12862_2015_309_MOESM3_ESM.pdf]

Additional file 3: Text S1. Additional Atlantic salmon data

Table of contents

		Page
Text S1a	Summary table of salmon MHCI genes	2
Text S1b	1. Salmon MHCI amino acid sequences	3
	2. Translated EST matches to new Atlantic salmon MHCI genes	7
	3a. Expression of Atlantic salmon MHCI genes	9
	3b. Expression of Atlantic salmon MHCII genes	10
	3c. Expression of selected human MHCI and MHCI-like genes	10
Text S1c	Gene organization of Atlantic salmon MHCI Z lineage genes	11
Text S1d	Location of genes within newly identified Atlantic salmon MHCI regions*	12

*Preliminary Atlantic salmon scaffold sequences have been deposited at Dryad with the following identifier: doi:10.5061/dryad.928fj.

Text S1a. Summary Table of Salmon MHCI genes

Gene	Location	Contig or Genbank Accession #	Expressed match (ESTs=528.289)	References
UBA	Chr.27	BAC #EF441211 (868O01)+ BAC #EF210363 (714P22)	(355 aa) 4 ESTs 100% ID 100% cov. e.g. EG647673	Grimholt et al.1993 Lukacs et al.2007
UCA ψ	Chr.14	BAC #EF427379 (8I14)	(335 aa) pseudogene	Lukacs et al.2007
UDA	Chr.14	BAC #FJ969490 (439J08)	(316 aa) 1 EST 100% ID 100% cov. CK898732	
UGA	Chr.14	BAC #GQ505859 (439H13)	(363 aa) 6 ESTs 100%ID 100% cov. eg. CK883511	
UHA1	Chr.21	BAC #FJ969489 (184H23)	(312 aa) <98%ID 100% cov. e.g. EG787974	
UHA2	Chr.21	BAC #FJ969489 (184H23)	(236 aa) <99%ID 100% cov. e.g. GE766956	
ULA	Chr.27	BAC #EF441211 (868O01)	(306 aa) 5 ESTs 100%ID 100% cov. eg. BG935668	Lukacs et al.2007
SAA	Chr.9	BAC #FJ969488 (114L13)	(315 aa) <99%ID 100% cov. e.g. DY713846	Lukacs et al.2010
LCA	n.d.	AGKD03039122.1:3,341-4,520 (21 kb)	(360 aa) <100%ID 100% cov. but transcriptome match	Dijkstra et al.2007 and this study
LDA	n.d.	AGKD03037778.1:12,196-13,372 (16 kb)	(366 aa) <90%ID 100% cov., TSA match JT833250	
LFA	n.d.	AGKD03073275.1:8,761-9,894 (14 kb)	(345 aa) 1 EST 100%ID 100% cov. EG847842	This study
LGA	n.d.	AGKD03064454.1:3,875-6,336 (15 kb)	(366 aa) 2 ESTs 100%ID 100% cov. eg. EG931865	
LHA	n.d.	AGKD03049350.1:3,883-6,976 (17 kb)	(347 aa) <99%ID 100% cov. but transcriptome match	
LIA	Chr.21	AGKD03025882.1:26,896-28,189 (31 kb)	(360 aa) <82%ID 100% cov. but transcriptome match	
LJA ψ	n.d.	AGKD03038500.1:1,831-3,283 (3 kb)	(260 aa) Pseudogene	This study
LKA ψ	n.d.	AGKD03020594.1:773-1,321 (11 kb)	(183 aa) Pseudogene	
LLA ψ	n.d.	AGKD03683246.1:93,581-96,980 (114 kb)	(67 aa) Pseudogene	
LMAψ	n.d.	AGKD03030462.1:91,197-91,772 (114 kb)	(136 aa) Pseudogene	
ZAAa	Chr.27	BAC #GQ505858 (129P21)	(348 aa) <95%ID 100% cov. e.g. DQ099914	Lukacs et al.2010
ZBAa	Chr.27	AGKD03017891.1: 51,744-55,896	(339 aa) <98%ID 100% cov. but transcriptome match	This study
ZCAa	Chr.27	AGKD03017891.1: 38,609-40,974	(346 aa) 3 ESTs 100%ID 100% cov. eg. EG858590	
ZDAa	Chr.27	AGKD03017891.1:9,374-13,096	(382 aa) 4 ESTs 100%ID 100% cov. eg. GW914341	
ZBAb	Chr.14	BAC #GQ505860 (68019)	(346 aa) <69%ID 100% cov. e.g. DY730127	Lukacs et al.2010
ZCAb	Chr.14	BAC #GQ505860 (68019)	(364 aa) <72%ID 100% cov. e.g. DY740683	
ZDAb ψ	Chr.14	BAC #GQ505860 (68019)	(294 aa) pseudogene	
PAA ψ	n.d.	AGKD03005975.1:1,496-2,200 (34 kb)	(157 aa) pseudogene	This study

Summary of Atlantic salmon MHCI genes, chromosomal location, expressed match and reference when previously published (shaded grey). Expressed match relates to amino acid sequence identity to the alpha 3 domain using tBlastN. Previously published sequences were subjected to similar treatment for comparison. Sequence references are: Grimholt et al. Immunogenetics 37:469-473, 1993; Lukacs et al. BMC Genomics 8:251, 2007; Lukacs et al. BMC Genomics 11:154, 2010; Dijkstra et al. Immunogenetics 59:305-321, 2007.

Text S1b1. Atlantic salmon (*Salmo salar*) MHCI amino acid sequences

>UBA*0301 U lineage AAN75116.1
MKCFILLLLGIALHSSSAATHSLRYVYTATSGIPDFPEFVTVGLVNGEPISYYDSIIRRE
TPRQDWMAKTEGSDYWESQTQVSIGSEQTFKANIDVAKQRFNQTGGVHVNQKMYGCEWDD
ETGVTEGFDQDGYDGEDFLAFDLKTLTWIAPTPQAVITKLKWDSNTAQNEYRKNYLTQTC
IEWLKKYLDYGKSTLMRTVPPSVSLLQKTPSSPVTCHATGFYPSGVMVSWQKDGQDHHED
VEHGETLQNDDGTFQKSSHLTVTPEEWKNNKYQCVVQVTGLQEDFIKVLTESEIKTNWND
PNIVLIIGVVVALLLVVVAVVVGVVIWKKKSKKGFVPASTSDTDSDNSGRAAQMT
>UBA*0901 U lineage AAN75119.1 AF504025
FILLVLGIGLLHTASAVTHSLRYFYTATTGIPDFPEFVDVGVVNGKVISYYDSIIKRKVP
KQSWMEENLNQQYWNQGTDQLKGTEQSFKANIQVAQTRFNQTGGVHIFQYMYGCTWDDDS
GVTDGLRQYGYDGEDFLVYDMKAFTWIAPKLQAEITTRKWNNEPAQMEYLKSYITQECVE
WLKKYVDYGKNTLMRTVPPSVSLLQKSPSSPVTCHATGFYPSGVMVFWQKDGQDQHEDVE
YGETLPNHDGTFQKSSHLTVTPEDRKNNKYQCVVQVTGIKDDFIKVLTDLDDPTPNIVPI
IVVVVALLLVVVAVVVGVVIWKKKSKKGFVPASTSDTDSENSGKGAQKI
>UCAΨ U lineage (BAC FJ969490.1) pseudogene
MKSFILIFLGIVEVHETFAVTQSLKHFYTASSKVTNFPEFVVVGMVDGVQMVLYDSNIQK
VVPEQVWMNKADAEYWEREREKFLHSQHDFKADVDILKQSFNQRGGVHVLQYIYGCSWDD
ETEQRDGFGQLGYNGEDFLVYDMNTLTWKALKQQADVMRDKWNRDISRLVFWKTYFSQTC
IECLKKQVVNGKSTLRTAAPPSVSLLQKTPSSPVTCHATGFYPSGVMVFWQKDGQEQHED
VEHGEILHNDDGTFQKSTHLRVTPEEWKNNKYQCVVQVTGIKEDFIKVLTESEIQTNWGN
PAPIIVPIIGGVVALLLVIVDVVGVVIWKKKSKKG
>UDA U lineage (BAC FJ969490.1) ACY30371.1
MKGFILMFMGTCHLFEAFGVTHSLKHFYTASSKVTNFPEFMVVGMVDGVQIDHYDSNIQR
MVPKQDWMNKQTEAEYWERETGIAFDSQQVFKDDVNILKQRFNQSGGVHVLQYIYGCSWD
DETEQRDGFGQLGYNGEDFLVYDMNTLTWKALKQQADVMRDKWNRDISRLVFWKTYFSQT
CIECLKKQVVNGKSTLRTAPPSVSLLQKTPSSPVTCHATGFYPSGVMVFWQKDGQEQHED
VEHGEILHNDDGTFQKSTHLRVTPEEWKNNKYQCVVQVTGIKEDFIKVLTESEIQTNWGD
PAPIIVPIIGGVVALLLVVVVVGVVIWKKKSKKGFVPASTNDTDSVYSGKDLLKT
>UGA U lineage ACX35601.1
MKTRLISAMKIYFVLLSCIHGALSVIHSLRYFYTSSSGISDFPEFVDMGMVNDQVISHYD
SITKRKVPKQSWMGKVFDQQYWDSTTEDLRGAEKVFKNNLQTAQKRFNQTGGMHISQDMY
GCEWDDETGLTEGFHHIGYDGQDLLVFDLKRATWIASVPQALHSKMKWEGDPSSIESEKR
YLTQDCIVWLKKYLEYGKTTLQRTVPPSVSLLQKTPSSPVTCHATGFYPSGVMVFWQKDG
QDHHEDVENGETLHNDDGTFQKRTHLKVTSEEWKNNKYQCVVQVTGIKEDFIKVLTESEI

QTNRGVNTIGSAPIIGVVVALLVVVVVVVGLVMWRRKKSKKGFVPASTSDTDSENSGKGA
QKI
>UHA1 U lineage ACY30367.1 Chr.21 with match SNP marker ESTNV_36193_1646
MIVLNMYSVSNRIVHISTLLVTYLIPMTFAANHSLKYFYTALPQSTGLPEFSAVAYLDEE
PMYFYDSSTKEVVARQEWVKGAVDPDFWRRNTQILKENEMVFKDNMDTARDRFNQTSALV
LQKMYSCDWDEVTGATDEREQYGYGGEDFLLFDLKNKRWIVPGRQGLITKMKWDANVIKL
EAKIHYLTHTCIEWLKKYVSNWRRNLERTVPPQVSLLQKEPSNPVTCHATGFYPNAIMIF
WGRDGVEIHADVVHEETLPNGDGTYQKRIHLTVSPEDLQQHNYTCTVQHVRGDDVVLSAN
RDSIRSNSRNTQAKTAEMEKPKKRHSADTLQIQLVRIFIPVFV
>UHA2 U lineage ACY30368.1 Chr.21 with match SNP marker ESTNV_36193_1646
MGGQILVLWLCLSLRTANSATHSLKYFYTALPQSTGLPEFSAVAYLDEEPMYFYDSSTKE
VVARQEWVKEAVDPDFWRRNTHIFKETEKVFKYNMDSARDRFNQTSALALQKMYSCDWDD
VTGAATDGREQYGYGGEDFLSFDLKNERWIAPGRQGLITKMKWDANVIKLKAKIHYLTHT
CIEWLKKYVSNRRRNLQRTVPPQVSLLQKEPSNPVTCHATGFYPNAIMILWGRDGVEIHD
DVVHEETLPNGDGTYQKRIHLTVSPEDLQQHNYTCTVQHIRGDDVVLSANRDSIRSNSRN
TQGHYITIVLVTLAFSVIVVFIMTKVCKGKEPTAGKEKQCPSEY
>ULA U lineage (BAC 30C23) ABQ13870
MKCFILLLLSISLHAASAAMHSLRYVYTATSGMPDFPEFMTVGLVNGEPISYYDSIIRRE
TPRQDWMKEAVDPDYWNRNTQTSIGDEQTFKANIDVAKQRFNQTGGVHVYQNMYGCEWDD
EAGVTEGFDQYGYDGEDFLAFDLKTLKWIAPTPQSLITKLKWDNNMAQIQQDKHYLTQTC
IEWLKKYLDYGKSTLMRTVPPSVSLLQKTPSSPVTCHATGFYPSGVMVSWQKDGQDHHED
VEYGETLQNDDGTFQKSSHLTVTPEEWKNNKYQCVVQVTGVKEDFIKVLTESEIKTNWGN
TNIGFVPANTSDVGSNSSHNTVPKE
>SAA S lineage BAC: FJ969488.1, ACY30362.1
MITTILISFMQFSIVAPHSLHRHCIATQGTLYPKNIQLVMIDDVTVYYYNSSAEQEAVVP
EWLNHLEGIEFWQEVNRNLKFSRFVMDTAVRVTSEHYNHSHDHFYQAHGRCGWKSDGTTE
AFMSHAYDGKDFVSFDVSTRTWTAAVSHAVFYKRKRETDLEDLVRLVIHYESGCIRWLKK
LLQFSVTFREPKVPAVSLFERPPHGNSEVEVTCHVTGFYPRAVQVEWLGAEGLPMVDGVS
SGEVLPNGDGSYQLRKSLTVPQEAQDTQSYSCLVLHSSIAGNITVTWAPKKNLANVLMAI
VIIVSVVLILTVLFKYLVRRRAVGKSQS
>LCA L lineage AGKD03039122.1:3,341-4,520, transcritpome match
MGKLSVFLFVLSFYTIVNSGSGSHSLWALATYISGETPFPEFTVVVMLDDVQVAYYDSNM
KHFIYRGHNTPNKIHDDEAKNGDFVFGVMYHHMKERYFHLKHHLNLTEGVQVQQRMAGCE
MFDNGEPALIMTKNTFNAVFADHAIYYNITHFTYDAGKLLQGWDGMRQAQEKILYENVLL
TLCIRTLKTILKREKNIVMRKVPPRLRLIKKEVSGGFQVSCLVFGFYPRHINLTLLRDGQ
PVAEQELTGGEVLPSGDGTYQLRKSLEVSTEELKKRHNYTCTASHLSLDNKLDVSWESGA
ERVHLSTLSVLLVMLLILILLVTFICVKRRWSNTASQSELANVDAKVSEEMNLSSDSEN
>LDA L lineage AGKD03037778.1:12,196-13,372
MGKLSIFLFVLSFYTIVNAGSGSHSLWALATYIIGETPFPEFTVVLMLDDVQIGYYDSNI

KQSVYRGYHITDKMNDEAQDGTYVLGTMYDHMKERSFRLKHHLNLTEGVHVQQRIGGCEI
LHNGEPALIMTKNSFNAIFEDYAVYYNMTHFTYDSGKLLLGYNWIRQATERTLYANVWLP
ICINTLKKCLKRENFVMRRVPPRLRLIKKAVSGDIQVICLAFGFYPRHINLTLLRDGHPV
AEQELTGGEVLPSGDGTYQLRKSIYVSTEELRERHNYTCTASHLSLNNKLDVSWESGAER
VHLFILSAPLVMALIVILFCIFICLVRRIRAASQNLLQLASVDALEADEMNLSSDSEKT
>LFA L lineage AGKD03073275.1:8,761-9,894 and EG847842
MGKLSVLFFILFYTIGNAGSGSHSLWALATYINGETPFPEFTVVVMLDDVQVGYYDSNMK
DFIYRGHNPTDKIHDDVAQDGAYVFGIIYQSIKERSFHLKHQLNLTEGVQVQQRMSGCEM
LDNGEPALIMFKETFNGIFVDHAIYNSMTHFTYDSGTLLLGYYGIRQAYEKALFENVLLP
ICIKNLKTILKREKNVVMRKVPPRLRLIKKEVSGGLQVSCLAFGFYPRHINLTLLRDGQP
VAEQELTGGEVLPSGDGTYQLRKSLEVSTEELKKRHNYTCTASHLSLDNKLDVSWESEAE
RVHLSTLSVLLVMLILILLGIFICVKRRWRCTASHLKLVNVDAKA
>LGA L lineage AGKD03064454.1:3,875-6,336 and DY733800
MGKLSVFLFVFSFYTIVSPGSGSHSLWALATYIVGETPFPEFTVVVMLDDVQVAYYDSND
KQSVYRGQHITKTKDDEAQDGAHVFRVIYQSMKDRSFELKHRFNLTEGVQVQQKITGCEM
LNNGESALVMYKDVFNAIYTDRTLYYNMTHFTYDAGKLLLGWDGIRQAYERTLYENVYLP
ICIKSLKRLLKREKNIVMRKVPPRLRLIKKEVSGGFQVSCLAFGFYPRHINLTLLRDGQP
VAEQELTGGEVLPSGDGTYQLRKSLEVSTEELKKRHNYTCTASHLSLDNKLDVSWESGAE
RVHLSTLSVLLMMLLILILLGIFICVKRRWSNTASQSELANVDAKVSEEINLSSDSET
>LHA L lineage AGKD03049350.1:3,883-6,976, transcriptome match
MGKLSVFLFVLSFYTIANAGSGSHSLWALATHIIGETPFPEFTVVVMLDDVQVGYYDSNM
KHFIYSGHNPTDKIHDDVAQDGAYVFGTMYQSIKERSFHLKYHLNLTRGVQVQQRMAGCE
MLNNGEPALIMSKNTFNAIYTDRTLYYNMTHFTYDAGKLLPGWDAMRREYLRILFGNVFL
PICIKTMKTFLKMEKNVVMRKVPPRLRLIKKEVSGGLQVSCLAFGFYPRHINLTLLRDGQ
PVAEQELTGGEVLPSGDGTYQLRKSLEVSTEELKKRHSYTCTASHLSLDNKLDVSWEPGA
ERVHLFTISILLMMLLIVILLGIFICVKRRRCTASQAFVTSCQHGGH
>LIA L lineage AGKD03025882.1: 26,896-28,189, transcriptome match
MAKLCFFLILLSLYTIVNAGSHSLWAFATCISGEAPFPECSVVLMQDDIQVGYFDSNKEQ
FIHKGPYAPDETEVEEAQDAAYVFGHMFLSMKRRLSDLRYRFNSTGNIDVQQRMAGCEML
DTGEPGLILSTDAFNAILADLIYYNMTHYSYNSGNLLSPWSEVHQTYTKWHYQTIYLPVC
IKTLKRFLERLKNFVMRKVRPRVRLIQKAMSGGACVSCLAFGFYPRHINLTLLRDGQPIV
EQEMTGGQLLPNGDGTYQMRKSLEVNTEELRERHNYTCTTSHLSLDNKLDVSWIPESGMD
RVGLYVKSAPLATVAIIILLSIFVCVRRRNTAGSQTLSQLSNANDAQVAEQISLSSHSET
>LJAΨ pseudogene L lineage AGKD03038500.1:1,831-3,283
SGSHSLWAFATYIIGDTPFPEYTVLLLLDDIEVGYYERQFVYRGHNALDEKEMGIVLDIA
SVFGAMSFSMKGRSYDLKQLFHFTEGIHVQQRMACCEMLDNDKLVLILSRSTFNKIVADG
MCYNMTQNTYYTGNPQLAWDEVKLEYVKMLYAHVYLPLCIKTLNIFLEREKNIVMRKVRP
RIRLIKKAKSGGLQENDRLTARIAVLQAQLQTQSLGKGNFSVGKDETASVPP
>LKAΨ pseudogene L lineage AGKD03020594.1: 773-1,321

ECSPSHLHQNTEDSPEEREERCDAKVPPRLRLIKKEISGGFQVSCLVFGFYPRHINLTLL
RDGQPVAEQELTGGEVLPSGDGTYQLRKSLEVSTEELKKRHNYTCTASHLSLDNKLDVSW
ESGAERVHLSTLSVLLVMLLILILLVTFICVKRRWSNTASQSELANVDAKVSEEMNLSSV
SET
>LLAΨ pseudogene L lineage AGKD03683246.1:93,581-96,980
HPLSGSGSHSLWAFATYISG*TPFPTVVLMLDDIQVGYFDSNIKRYIHKGYNASEETEVE
EAYHIAFIFGPMSFSLRARSSELKRFNS*EGIHVQQRLAGCEMSDNGEPALIVSSLNGIY
ADSAIYYNMTHYSYNSGKLFPPWSEVHQTYS
>LMAΨ pseudogene L lineage AGKD03030462.1:91,197-91,772
YVKGLYQTIYLNICMDTLKTFLEKEKKCIMHKVHARVRLIQKDMSGGVQQMSCLPFGFYP
RHINLTLLRDGRPIAEQVLNGGAAAAQWRRHVPAEEESGGQYTGTKRDTTTPALPPTSVW
TTRWMSAGYLSLEQTE*VCLSCQLYWSITILICISVCLRKRSEAGSQTLSQLSNAVDAQV
DEQISLSSHSAT
>ZAAa Z lineage IA region ACX35596.1 GQ505858.1 AGKD03017891.1:178,619-195,734
MNISHLTVFVLYFSLECICQSDTYSLSYIYTALSKPVDLPGIHEFTAMGLMNNQQIDYYD
SVSKKKIPKQDWMREKLPADYWEKGTQSRKSKEQWFKVNVDILMKRMRHNNTDVHVLQWK
VGCEIDQQSDGTLKFIKGIDQYSYDGDDFLAFDDVTMQWVAPVDQALPTKRKLDDVQILN
TYTKGYLEKECVDWLSKFMEYEDKEFSWADSAPKVYAFAKKAKTAGHVRLTCMATGFYPK
DVVMHIKKNGVPLTDRDGVQSAGLLPNDDETYQIRMSVQIPEADKETYECYVNHRALKEP
IVVKWDGKCCDCSSGGAVVIGAVVIAFIVVLILVGLFVLHRRGTIGRS
>ZBAa Z lineage IA region AGKD03017891.1:51,744-55,896
MNSGAAMARRPVTSTAECRTPPEQGEEPTSAEVVTSDIYSLNYIYTALSKPVDLPGIHEF
NAMGLMNNKQIDYYDSVSKKKIPKQNWMREKLPADYWEKGTQSRKSKEQWFKVNVNILMD
RMRHNNTNVHILQWKHGCEIDQQRDGTVKFIKGIDQYSYDGDDFLAFDDVTMQWVAPVDQ
ALPTKRKWDGVQILNQYTKGYLEKECVDWLSKFMEYGDKEFSRADSAPKVYAFAKKAKTA
GHVRLTCMATGFYPKDVEMNIKKNGVPLTKHDGVQSAGVLPNDDETYQIRMSVQIPEADK
ETYECYVHHRTLEEPIVIKWDPLKHCQVEWGASIHIYFQVSRDVRSGSSLGSCWVTQGHS
EPCPEANPALSWLCA
>ZCAa Z lineage IA region AGKD03017891.1: 38,609-40,974
MSAFKMYVVALLLLFATLSTEDTVETWSLNYIYTALSKPVELPGIHEFTAMGLMNDKQID
YYDSVAKKKIPKQDWMREKLPADYWEKGTQSRKSKEQWFKVNVNILMDRMRHNNTDVHIL
QWKHGCEINQQSDGTLKFIKGTDQYSYDGDDFLAFDDVTMQWVAPVDQALPTKRKWDGVQ
ILNQYTKGYLEKECVDWLSTFMEYGKKHLRMDDSAPKVYAFAKKAKTAGHVRLTCMATGF
YPKDVVMHIKKNGVPLTKHDGVQSAGVLPNDDESYQIRMSVQIPEADKETYECYVYHRTL
EEPIVEKWDGKFYDCNQVTGVIIGVVAVVLLFIVVTPLLVLWKKGK
>ZDAa Z lineage IA region AGKD03017891.1: 9,374-13,096
MEIIVEAAEQFLGLKDSAKEYDGRYGELKEVVRVLFYIYSLNYIYTALSKPVDLPGIHEF
TAMGLMNNQQIDYYDSVSKKKIPKQDWMREKLPADYWEKGTQSRKSKEQWFKVNVNILMD
RMRHNNTDVHILQWKHGCEIDQQSDGTLKFIKGTDQYSYDGDDFLAFDDVTMQWVAPVDQ

ALPTKRKWDGVQILNQYTKGYLEKECVDWLSKFMEYGEKEFSRPDSAPKVYAFAKKAKTA
GHVRLTCMATGFYPKDVVMHIKKNGVPLTDRDGVQSAGVLPNDDETYQIRMSVQIPEADK
ETYECYVHHRTLEKPIVIKWDGICCDCSSFNAVVIGAVVITFIVVLILVVLFVLHRRGTI
VIPGLRTTATGNGVAFSGVNTS*
>ZBAb Z lineage IB region ACX35613.1 GQ505860.1
MYTFMLFVFYFSTECIVQSQSEIYSLNYIYTALSKPVELPGIHEFTAMGLMNDIQIDYYD
SVDKKKIPKQDWMREKLPADYWEKGTQSRKRKEQWFKVNVNILMERMRHNNTDVHVLQWR
HGCEVDKQPDGTLKFMKGIDQYSYDGDDFLAFDDVTMQWVAPVDQALPTKRKWDGVQILN
QYTKGYLEKECVDWLSKFMEYGEKHFSSTDSPPNIYVFTKKAKPAGNVHLTCMVTGFYPK
DVIIHFKKNGVQLTEDDGVLSTGARPNNDDTYQIRISVQIPEADKDMYECSVSHAMLKEP
IVEKWGAGNTGAPPPTGIQASLIGNGNGATNLNLTTTPSKTLFQYL
>ZCAb Z lineage IB region ACX35618.1 GQ505860.1
MYTFMLFVFYFSTECIVQSQSEIYSLNYIYTALSKPVELPGIHEFTAMGLMNNRQIDYYD
SVDKKKIPKQDWMRDKLPADYWEKGTQSRKSKEQWFKVNVNILMERMRHNNTGVRILQWK
HGCEVDKQPDGTLKFIKGTDQYSYDGDDFLAFDDVTMQWVAPVDQALPTKRKWDGVQILN
QYTKGYLEKECVDWLSKFMEYGEKHFSSADSPPDINVFANKAKTAGNVHLTCMATGFYPK
DVIIHFKKNGVQLTEDDGVLSTGARPNNDDTYQIRISVQIPEADKQTYECSVSHITLVQP
IVVKWGKVVLVFNILYIFSTNQLFAWLSIMVKSISKMFVCYKVTNADLNVNQQLFQAHCI
GFCC
>ZDAbΨ Z lineage IB region GQ505860.1 pseudogene
EIHSLNYIYTALSKPVELPGIHEFTAMGLMNDKQIDYYDSVDKKKIPKQDWMREKLPADY
WEKGTQSRKSKEQWFKVNVNILMERMRHNNTGVHILQWRHGCEVDTQPDGTLKFMKGTDQ
YSYDGDDFLAFDDVTMQWVAPVDQALPTKRKWDGVQILNQYTKGYLEKECVDWLSKFMAY
RDKEFIRADSPPKVYAFAKKAKTAGHIRLTCMATGFYPKDVLMHIKKNGVQLTKQDGVQS
DGVLPNDDESYQIRMSVQIPEADKETYECYVSHRTLKEPIEVKW
>PΨ pseudogene P lineage AGKD03005975.1:1,496.2,200
VLQRRRGCTYITN*SSTGFGQ*GLNGENFLTFDPTSHTWMSESLQANPIEQSWNSNKVRS
HMFKEFLQHDCPH*EDVSLRCHVTSTDLSGLKVHLTRDRGVMTDRARVIGPLPNVDGSVL
LRLSVEIPTGHTKS*RYHCKVQTSTSNTAA

Text S1b2.Translated EST matches to new Atlantic salmon genes

>DY713845 Salmo salar cDNA clone ssal_rgb2_612_323_fwd 3', mRNA sequence SAA match
HGNSEVEVTCHVTGFYPRAVQVEWLGAEGLPMVDGVSSGEVLPNGDGSYQLRKSLTVPQE
AQDTQSYSCLVLHSSVAGNITLTWAPKKNLANVLMAIVIIVSVVLILTVLFKYLVRRRAV
GKSQS*
>JT833250.1 TSA: Salmo salar isotig20196.Sasaskin mRNA sequence LDA match

NLRHATQGYTQNHGQTVNLLFVLSFYTIVNAGSGSHSLWALATYIIGETPFPEFTVVLML
DDVQIGYYDSNIKQSVYRGYHITDKMNDEAQDGTYVLGTMYDHMKERSFRLKHHLNLTEG
VHVQQRIGGCEILHNGEPALIMTKNSFNAILK
>EG84784 Salmo salar cDNA clone ssal_eve_567_025_fwd 5', mRNA sequence LFA match
GKETFNGIFVDHAIYNSMTHFTYDSGTLLLGYYGIRQAYEKALFENVLLPICIKNLKTIL
KREKNVVMRKVPPRLRLIKKEVSGGLQVSCLAFGFYPRHINLTLLRDGQPVAEQELTGGE
VLPSGDGTYQLRKSLEVSTEELKKRHNYTCTASHLSLDNKLDVSWESEAERVHLSTLPLL
LVMLX
>EG931865 Salmo salar cDNA clone ssal_evf_544_381_fwd 5', mRNA sequence LGA match
GYDAGKLLLGWDGIRQAYERTLYENVYLPICIKSLKRLLKREKNIVMRKVPPRLRLIKKE
VSGGFQVSCLAFGFYPRHINLTLLRDGQPVAEQELTGGEVLPSGDGTYQLRKSLEVSTEE
LKKRHNYTCTASHLSLDNKLDVSWESGAERVHLSTLSVLLMMLLILILLGIFICVKRRWS
NTASQSELANVDAKVSEEMNLSSDSET*
>EG858590 Salmo salar cDNA clone ssal_eve_570_330_fwd 5', mRNA sequence ZCAa match
PLGWLSTFMEYGKKPLRMDDSAPKVYAFAKKAKTAGHVRLTCMATGFYPKDVVMHIKKNG
VPLTKHDGVQSAGVLPNDDESYQIRMSVQIPEADKETYECYVYHRTLEEPIVEKWDGKCY
DCNQVTGVIIGVVA
>EG777219 Salmo salar cDNA clone ssal_evd_503_317_fwd 5', ZCAa match
GGELSWL*KPVVTLFGSPWNLTQTLFRCILFLLFSGHLTIMSAFKMYVVALLLLFATLST
EDTVETWSLNYIYTALSKPVELPGIHEFTAMGLMNDKQIDYYDSVAKKKIPKQDWMREKL
PADYWEKGTQSRKSKELHHLLGSRNL*
>GW914341 Salmo salar cDNA, mRNA sequence ZDAa match
RGEKEFSRPDSAPKVYAFAKKAKTAGHVRLTCMATGFYPKDVVMHIKKNGVPLTDRDGVQ
SAGVLPNDDETYQIRMSVQIPEADKETYECYVHHRTLEKPIVIKWDGKCRDCSSFNAVVI
GAVVITFIVVLILVVLFVLHRRGT
>GO056020 Salmo salar cDNA clone ssal_rgh_505_248 5, mRNA sequence ZDAa match
MYRPNLMFFVLYFFLECICRSQSDIYSLNYIYTALSKPVDLPGIHEFTAMGLMNNQQIDY
YDSVSKKKIPKQDWMREKLPADYWEKGTQSRKSKEQWFKVNVNILMDRMRHNNTDVHILQ
WKHGCEIDQQSDGTLKFIKGTDQYSYDGDDFLAFDDVTMQWVAPVDQALPTKRKWDGVQI
LNQYTKGYLEKECVDWLSKFMEYGEKEFSRPDSPPKVYAFAKKAKTAGHVRLTCMATGFY
PKDV
>GW915210.1 Salmo salar cDNA, mRNA sequence ZDAa EST match
RGEKEFSRPDSAPKVYAFAKKAKTAGHVRLTCMATGFYPKDVVMHIKKNGVPLTDRDGVQ
SAGVLPNDDETYQIRMSVQIPEADKETYECYVHHRTLEKPIVIKWDGKCRDCSSFNAVVI
GAVVITFIVVLILVVLFVLHRRGTIVIPGLRTTATGNGVAFSGVNTS*
>DW559010 Salmo salar cDNA clone ssal_rgb2_538_019_fwd 3', ZCAb match
PKDVIIHFKKNGVQLTEDDGVLSTGARPNNDDPYQIRISVQIPEADKQTYECFVSHITLV
QPIVVKWGKVVLVFNILYIFSTNQLFAWLSIMVKSISKMFVCYKVTNADLNVNQQLFQAH
CIGFCCKSWNP*

Text S1b3a.Expression of Atlantic salmon MHCI genes

Expression of salmon class I genes was estimated by transcriptome analysis. The transcriptome data showed that the classical UBA gene was expressed abundantly and ubiquitously as expected. Surprisingly, also the nonclassical genes UGA, ULA, ZDAa and partly ZBAb displayed an abundant and ubiquitious expression pattern. For comparison, expression of Atlantic salmon MHC class II genes and human MHCI genes are shown in tables S1b3b and S1b3c respectively.

S1b3a. RPKM values for salmon MHC class I transcripts in various tissues

Tissues\ Gene	Glll	Gut	Head kidney	Kidney	Spleen	Brain	Eye	Heart	Liver	Muscle	Nose	Ovary	Pyloric caecum	Skin	Testis	Contig length (bp)
UBA	253,57	361,72	125,65	66,97	260,43	17,65	11,75	16,79	12,92	20,14	60,70	1,25	99,21	4,84	60,84	1068
UDA	5,65	4,27	7,40	4,05	6,63	1,75	0,79	1,35	0,78	1,68	5,44	4,29	2,57	0	6,31	1068
UGA	148,60	65,86	122,13	54,90	194,53	15,44	17,49	23,11	20,31	23,76	59,84	24,18	38,31	3,63	24,29	1092
UHA1	5,98	4,41	10,00	3,81	10,19	1,02	0,64	1,10	2,72	1,39	1,76	0,02	2,80	0	4,09	1032
UHA2	11,44	7,24	20,13	6,96	21,55	1,33	1,10	1,50	1,79	2,56	5,03	0,13	4,50	0,19	4,75	1035
ULA	63,72	108,02	39,44	18,10	91,99	6,07	2,85	6,49	4,37	6,83	17,64	1,11	24,53	2,07	35,41	978
SAA	5,52	3,50	10,33	5,64	7,68	0,17	0,09	0,90	1,47	0,37	2,21	0	2,05	0	0,48	975
LCA	0,87	0,28	5,96	3,38	4,89	0	0,23	0,26	0,33	0,08	0,28	0	0,23	0	0,14	1080
LDA	3,36	1,86	1,93	4,17	2,72	0,30	0,15	1,24	1,23	0,68	2,02	6,08	1,85	0,09	1,14	1080
LFA	2,93	0,10	0	0,21	0	0	0	0,23	0,18	0,03	0,55	0	0,19	0	0,02	1038
LGA	1,93	0,73	1,16	0,87	0,99	0	0	0	0,08	0,03	0,33	0	0,71	0	0,06	1077
LHA	1,51	0,82	2,08	0,89	1,53	0	0,13	0,33	0,31	0,06	0,56	0	0,56	0	0,20	1044
LIA	0,40	0,91	1,19	0,61	0,87	0,06	0	0,06	0,03	0	0,14	0	0,54	0	0,04	1083
ZAAa	1,98	0,45	1,31	0,70	2,44	0,31	0,59	0,26	0,28	0,53	0,79	0,60	0,19	0	1,56	1047
ZBAa	43,46	18,57	10,64	9,42	19,47	3,34	2,76	2,62	4,30	4,84	8,61	0,11	8,59	0,08	2,70	1128
ZCAa	118,47	16,24	0,05	9,45	0,05	0,07	0,72	0	0,03	4,94	45,19	0,02	1,25	0,73	0,26	1038
ZDAa	54,38	15,10	63,81	57,05	61,77	8,50	5,72	28,08	38,97	11,95	24,46	6,21	15,03	1,24	14,46	1080
ZBAb	16,50	78,59	17,90	19,13	21,38	2,61	1,97	8,11	6,96	5,35	7,86	0,26	47,59	0,34	8,37	1041
ZCAb	10,65	58,61	26,55	12,83	30,34	1,81	1,09	4,88	4,13	2,69	4,60	0,12	37,28	0	9,70	1095
Total # reads	59793962	59806348	59084708	61054936	60203316	58939250	60380888	58163180	58784272	61426586	59545012	84051830	61602874	270961444	185311952	

RPKM = Reads Per Kilobase Per Million mapped reads. Reads were mapped with high stringency i.e. greater than 95% identity over more than 90% of the total length of the query read. Tissues with RPKM values below 1 are shaded grey while those with RPKM values above 50 are shaded pink. The transcriptome was based on analysis of tissues of a single one-year old individual and contained > 70,000 non-redundant contigs.

Table S1b3b. RPKM values for salmon MHC class II transcripts in various tissues

Tissues\ Gene	DAA	DAB	DBA	DBB	DCA	DCB	DDA	DEA	DEB	Total # reads
Gill	394.69	234.79	0.52	0.04	0.36	0	6.50	0.45	0.05	59793962
Gut	259.73	221.51	0.16	0.11	8.10	19.60	1.54	0	0.02	59806348
Head kidney	238.07	155.41	1.67	0.99	0.23	0	8.28	0	0.07	59084708
Kidney	104.31	61.50	0.89	0.25	0.29	0.04	4.25	0	0.05	61054936
Spleen	677.01	291.31	4.40	2.12	0.07	0.04	27.90	0.02	0.23	60203316
Brain	5.99	3.54	0	0.04	0	0	0.29	0	0	58939250
Eye	9.03	6.01	0	0	0.02	0	0.63	0	0.02	60380888
Heart	15.61	8.36	0.14	2.61	0.05	0	0.76	0	0	58163180
Liver	15.11	9.22	0.25	2.28	0.02	0	0.43	0	0	58784272
Muscle	20.56	12.18	0.09	0.04	0	0	0.60	0	0	61426586
Nose	130.25	72.91	0.16	0.09	0.30	0	1.76	0.02	0.05	59545012
Pyloric caecum	41.36	26.77	0.18	0.12	7.75	12.84	1.11	0.15	0	61602874
Contig length (bp)	708	738	740	783	735	789	705	744	708	

RPKM = Reads Per Kilobase per Million mapped reads. Reads mapped with High Stringency i.e. greater than 95% identity over more than 90% of the total length of the query read. Mapping was done using CLC v 5.1.5. The transciptome was based on analysis of tissues of a single one-year old individual and contained > 70,000 non-redundant contigs. DAA and DAB are the two classical alpha and beta genes while DBA, DBB, DCA, DCB, DDA, DEA and DEB are nonclassical MHCII genes. Shading as in Table S1b3a.

S1b3c. Tissue distribution of selected human MHCI EST reads

	blood	brain	intestine	kidney	liver	lung	lymph node	muscle	pharynx	skin	spleen	thymus	tonsil	uterus
HLA-A	883	276	1646	844	652	677	189	112	98	464	2902	1091	1116	331
HLA-B	1856	327	2491	237	935	1744	1348	282	810	645	4457	915	1762	1688
HLA-C	8	4	0	0	0	5	0	0	49	0	0	0	0	8
HLA-E	703	164	491	294	121	570	289	282	294	284	1442	351	1880	275
HLA-G	0	1	34	0	0	2	0	0	0	0	0	12	0	0
MICA	24	11	25	42	48	35	0	18	0	33	0	50	0	25
MICB	57	5	8	14	9	8	0	0	0	0	56	37	0	8
CD1c	24	0	8	0	19	8	55	0	24	0	56	388	0	4
FCGRT	220	137	172	137	77	704	11	103	0	33	299	37	0	43
PROCR	16	5	68	18	19	95	0	28	0	128	37	0	0	30
AZGP1	0	4	81	42	472	29	0	9	0	52	0	0	0	0

EST numbers according to "EST Profile Viewer" at Unigene, NCBI http://www.ncbi.nlm.nih.gov/unigene. Numbers indicate gene-specific ESTs per million ESTs.

Text S1c. Exon intron structure of Atlantic salmon Z lineage genes

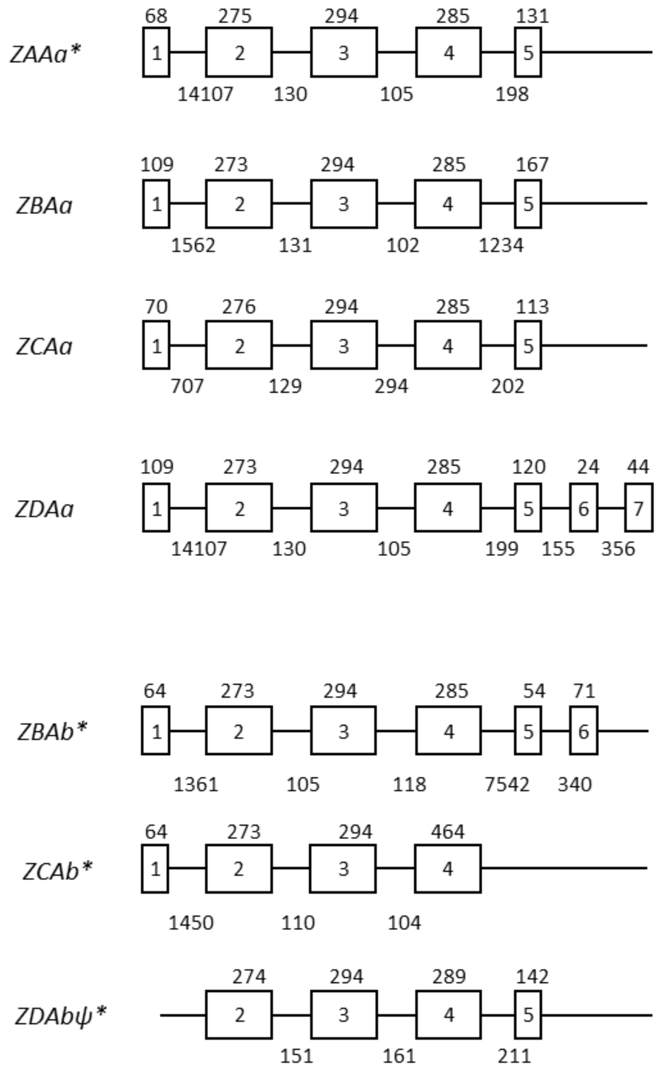

Genes marked with a star (*ZAAa*, *ZBAb*, *ZCAb*, *ZDAb)* were published by Lukacs et al.[main text reference 29] and the a and b represent the two duplicated MHC regions IA and IB. Exons sizes are shown above and intron sizes are shown below.

Text S1d. Location of genes within newly identified Atlantic salmon MHCI regions

Region	Contig location	Gene	Short name	Marker*
rLCA<br>217 kb scaffold sequence<br>(AGKD03039122.1: 21 kb)	1,339-52,035	Aryl hydrocarbon receptor nuclear translocator 2 like	ARNT2_L	No marker match
	132,569-132,751	Cortexin-2-like	CTXN2_L	
	146,134-146,781	MHCI pseudogene	LCAΨ	
	190,231-191,172	MHCI gene	LCA	
	210,302-202,111	Fumarylacetoacetase	FAH Ψ	
	202,205-215,525	Fumarylacetoacetase	FAH	
rLDA<br>174 kb scaffold sequence<br>(AGKD03037778.1:16 kb)	1,995-5,077	Ankyrin repeat and SOCS box protein 14	ASB14	No marker match
	6,282-28,384	DCC-interacting protein 13a like	APPL1_L	
	37,284-44,383	Interleukin-1 receptor-associated kinase 1-binding protein 1	IRAK1BP1	
	73,508-78,351	MHCI gene	LDA	
	145,911-163,550	Interleukin 17 Receptor D	IL17RD	
rLFA-LGA<br>52kb scaffold sequence<br>(LFA: AGKD03073275.1_14 kb<br>LGA: AGKD03064454.1_15 kb)	5,358-19,228	Fumarylacetoacetase	FAH	No marker match
	21,696-22,634	MHCI gene	LGA	
	42,446-43,348	MHCI gene	LFA	
rLHA<br>205 kb scaffold sequence<br>(AGKD03049350.1: 17 kb)	101,281-111,500	MHC class I	LHA	No marker match
	120.331-132,120	Heme binding protein 1 like	HEBP1_L	
	144,980-145,273	Macrophage-expressed gene 1 protein pseudogene	MPEG1_L	
	169,415-204,339	Transmembrane channel-like protein 3	TMC3_L	

rLIA (ssa21)<br>226 kb scaffold sequence<br>(AGKD03025882.1: 17 kb)	18,890-30,928	Coiled-coil domain-containing protein	CCDC80	Match to ssa21 SNP markers # 36 (GCR_cBin10561_Ctg1_206) and 37 (ESTNV_33891_846)
	48,306-53,783	Solute Carrier Family 35 Member A5	SLC35A5	
	54,260-70,808	Coagulation factor V	F5	
	76,518-77,811	MHCI gene	LIA	
	125,845-205,062	von Willebrand factor A domain-containing protein 8	VWA8	
	209,111-209,855	Diacylglycerol kinase eta-like pseudogene	DGKH ψ	
	213,736-214,904	PiggyBac transposable element-derived protein 4	PGBD4_L	
rLJA<br>110 kb scaffold sequence<br>(AGKD03038500.1:3 kb)	19-24,908	Ephrin type-A receptor 6	EPHA6	No marker match
	29,590-31,696	ADP-Ribosylation Factor-Like 6	ARL6	
	34,203-39,025	F-Box Protein 47	FBXO47	
	47,519-48,494	Claudin 14_like	CLDN14_L	
	59,012-63,556	Solute Carrier Family 5 Member 7	SLC5A7	
	108,491-110,241	MHCI pseudogene	LJA ψ	
rLKA<br>184 kb scaffold sequence<br>(AGKD03020594.1:11 kb)	31,499-38,087	ADAM Metallopeptidase With Thrombospondin Type 1 Motif, 17 pseudogene	ADAMTS17_Lψ	No marker match
	39,916-57,015	MHC class I pseudogene	LKA ψ	
rLLA_LMA<br>224 kb scaffold sequence<br>(LLA: AGKD03683246.1:114 kb<br>LMA: AGKD03030462.1: 114 kb)	4,637-14,864	Diacylglycerol kinase eta-like	DGKH	No marker match
	167,536-182,292	MHCI pseudogene	LMAψ	
	184,230-187,629	MHCI pseudogene	LLA ψ	
	209,653-220,223	Coiled-Coil Domain-Containing 80	CCDC80_L	

rZ_IA (ssa27)<br>AGKD03017891.1: 264 kb<br>(243 kb scaffold sequence)	16,001-18,655	Zinc Fingers And Homeobox 1	ZHX1	Not analysed
	25,300-39,521	ATPase Family AAA Domain-Containing	ATAD2	
	85,451-85,717	MHCI gene	ZDAa	
	90,075-121,348	MHCI gene	ZCAa	
	129,954-138,275	MHCI gene	ZBAa	
	140,265-141,073	Zinc Finger, BED-Type Containing	ZBED1a	
	148,599-151,890	Histone H3	HistH3	
	156,742-163,080	Proteasome subunit 7	PSMB7a	
	164,000-181,941	Tenascin XB	TNXBa	
	187,363-193,103	Tenascin XB pseudogene	TNXB Ψ	
	233,713-240,549	Activating Transcription Factor 6	ATF6a	
rP<br>208 kb scaffold sequence<br>(AGKD03005975.1: 34 kb)	109-3,023	Ferric-Chelate Reductase 1-Like	FRRS1L	No marker match
	26,351-36,913	Amylo-1, 6-glucosidase, 4-alpha-glucanotransferase-Like	AGL _L	
	42,671-98,002	Protein Phosphatase 1, Regulatory Subunit 12A	PPP1R12A _L	
	139,000-141,000	MHCI pseudogene	P Ψ	
	175,139-175,695	Immunoglobulin Kappa Variable domain	IgK V	
	176,803-183,614	Immunoglobulin Kappa Variable domain	IgK V	
	201,246-201,788	Immunoglobulin Kappa Variable domain	IgK V	
	205,655-207,933	Immunoglobulin Kappa Constant domain	IgK C	

Individual regions are shown with an r representing region prior to the gene name(s). Indicated gene locations refer to position in scaffolds with the exception of the duplicate rZ_IA region where position relates to contig (AGKD03017891.1). Marker refers to blastN match (E-value 0.0) between scaffold sequences and markers published by Lien et al., 2011 [main text reference 68]. The duplicate rZ-IA region was not tested for presence of markers as it overlaps with a region on Chromosome 27 previously published by Lukacs et al. [main text reference 29]. Preliminary Atlantic salmon scaffold sequences have been deposited at Dryad with the following identifier: doi:10.5061/dryad.928fj.
