# Supplementary material for: A comprehensive analysis of teleost MHC class I sequences
Source: BMC Evol Biol. 2015 Mar 6;15:32. doi: 10.1186/s12862-015-0309-1 (PMC4364491; doi:10.1186/s12862-015-0309-1)
Supplement: Additional file 4: — Text S2. MHCI amino acid sequences. [file 12862_2015_309_MOESM4_ESM.pdf]

Additional file 4: Text S2. MHC class I amino acid sequences:

Deduced ray-finned fish MHC class I gene sequences and expressed matches

Table of Contents		Short name	Page
Species			
1a	Cavefish (*Astyanyx mexicanus*) Ensembl genes	AM	2
1b	Expressed matches		6
2a	Zebrafish (*Danio rerio*) Ensembl genes	DR	7
2b	Expressed matches		11
3a	Medaka (*Oryzias latipes*) Ensembl genes	OL	12
3b	Expressed matches		14
4a	Platyfish (*Xiphophorus maculatus*) Ensembl genes	XM	14
4b	Expressed matches		16
5a	Nile tilapia (*Oreochromis niloticus*) Ensembl genes	ON	16
5b	Expressed matches		22
6a	Stickleback (*Gasterosteus aculeatus*) Ensembl genes	GA	22
6b	Expressed matches		26
7a	Green pufferfish (*Tetraodon nigroviridis*) Ensembl genes	TN	28
7b	Expressed matches		30
8a	Pufferfish (*Takifugu rubripes)* Ensembl genes	TR	30
8b	Expressed matches		34
9	Spotted gar (*Lepisosteus oculatus)* Ensembl genes	LO	34
	No expressed match		
10	Atlantic cod (*Gadus morhua*) P and Z lineage sequences	GM	35
11	Sablefish (*Anoplopoma fimbria*) P lineage sequences		36
12	MHCI sequences from other teleost and primitive ray-finned species identified in this study		37
Relevant previously published sequences:			
13	Zebrafish (*Danio rerio*) sequences		39
14	Atlantic cod (*Gadus morhua*) U lineage sequences		42
15	Tilapia (*Oreochromis niloticus*) MHCI sequences		44
16	Salmonids (*Oncorhynchus mykiss and O. nerka*)		45
17	Cyprinids (goldfish, common carp)		46
18	Paddlefish (*Polyodon spatula*) and Sturgeon (*Acipenser sinensis*) MHC class I sequences:		46
19	Shark, lungfish, chicken, Human, MHC class I sequences		47

All sequences are shown as (translated) protein sequences. Many Ensembl gene sequences have been manually edited, in particular 5' and 3' regions (alterations not shown). Gene sequences have been assigned abbreviated species names, e.g. DR for *Danio rerio* and lineages are indicated after assigned gene name. Alternative names are also shown when defined. Star defines stop codon. Some sequences are assemblies of short shotgun transcriptome or genome reads using the CAP3 sequence assembly program [Huang, X. and Madan, A. (1999) CAP3: A DNA sequence assembly program. *Genome Res.*, **9**, 868-877]. Grey shaded regions represent relevant previously published sequences.

1a. Cavefish (*Astyanax mexicanus*) Ensembl gene sequences:

```
>AM1_U ENSAMXG00000004456 KB871756.1:731,095-739,372
MCSMDCLCNTLQWKMIKTLIFLIFTTQLTSATISLQYYYTGVTPGISFPEFTATGQLNEH
QIGYYNTTSQRVIVTADWINRSKDEEHWKMMGHHAANDQELVRVRLANQIMLLNQTEGVH
TWQERYVCELDDDGTRRGYKQFGYDGEDFLSLDVNDEYWTAATPQAVPFKRKWDGLVSIQ
KKILETECFERIKKYVEYGKSVFERKVAPEVSLFQKDSSSPVVCHATGFFPKAVMVSWEK
NGEDLNEDVELRETLPNQDGSFQKRSILTLSPEELNRNKYTCVVQHEGLKKEIILQVSDR
RVLSDKGSVGIMVGIIIGVVLAVLLLGILGFAGGLMWKKRKKNSDFSLPVIFTSASAQDS
QTPAPTQTPGLDAVAAPH
>AM2_Z2 ENSAMXG00000020385 KB871871.1:534,379-556,328
KHSLLYLYTMQSKNSDSHIYNCTAVTLLNDRQIDFYSSSSDKPRTAKQNWLKNISESDWK
DSTEKLQYDRELLNILIVTQMSEFGHSQSGVHVLQWRHGCEGEQSSDGSLTVLNSIxxxx
xxxxXXXXXXXXXTPPVVHIFAKKSVRDSRKLILTCLITGFYPKHVKMSLRKFTTEIPDH
LITSSGIRPNDDETYQLKKSVEIQEDDPAYYDCYVSHSSLTEPVIKQWGKYNPTLLTYSS
VSSAEKREEFVEHVQVAAPLK
>AM3_Z2 ENSAMXG00000020387 KB871871.1:577,693-587,920
MMRSITALLLFSFLRIVIGEKLSLNYCYTMQYTNSESNLYDCTVVTLLNDTQIYFYNSAD
PNKTPKPHWLKISETDWIKSTTWSDGDRQWMNKVINIQSSVNKSVGHVLQWNLSCEGERH
APVFSSNEFGFDGENLIYFNCTSKTWLTPENKKNPEMEQLWNQQYRQLAVEKCVQCEEML
KMYLNYNTTDLTPSHSPTVYIFQNKSVSDSSELILTCMATGFYPKDVKMSLMKSGTEIPD
HLITSSGVRPNHNGTYQLRKSVEIQEDEKDQYECYVSHSSLTEPKIEKWEDKVSVGFIVG
VAVAVVAVVILFCGLVLFCALKNRKKKRE
>AM4_Z2 ENSAMXG00000020391 KB871871.1:596,826-603,393
MKCYIFKISIRMLKGVFAAMAADNHSLLYLYTMQSKNSYSNIYDCTAVTLLNDRQIDFYS
SSSDKPRTAKQNWLKNISESDWIISTEKLQYDTQLLKSLIDMQLNEFSPNQSDGHVLQWR
HGCEGEQSSDGSLTVLNSINEFGYDGEDLIQFNCTSKTWITPEENRERERERKWNKDHQY
RADKKCEQCLEMLKMYLKYNTTDITPKHTPPAVFIFAKKSKNDLKKLNLTCLITGFYNRD
VKMSLRKLTTEIPDHLITSSGFRLNDDETYQLRKSVEIQEDDSVDYSCYVSHSSLTEPVI
KQWVGRCTDCPDNAYQSLIIGVVVLGGLAAIYFLILGVLMMIIKQKSGEQSAC
>AM5_P No Ensembl ID KB871893.1:99,729-104,236
MRVLLLCVFCFTGETASAEKHSLQYLYTLRSAPEDDTEFEITTVFDGLIISHCKSPRFRD
HSREDWISQTFTNAEWKNRDLFCEGEYYLHNTLKKKIEDVINTTNGIIQREISCTEDDSV
VRMSDRWGVNGEDFLTLDPKTLKWSSDSPLAAPVQSDWNQMKFMTPSLKDFKLNQCKPSL
MKLKMKKEEYLQENMYPKMYIFGKSSHDGDAVSMRCYISHKYLSGVRVRLTLDGVAVDNV
NISSPAPNMDGSVQIRLETKTNIKEPNRYHCVVDTDYLHIFTAW
>AM6_P ENSAMXG00000005391 KB871893.1:114,151-120,095
MIFILLSVYCSVLETTSAEKHTLQYLYTLRSAPEDDTEFEITTVFDGLIISHCKSPRFRD
QSRYDWISQTFTDAEWKNMDLFCESEYYFHKTLKKKIEGVYNTTNGIIQRERSCTEDDSV
VLMSDSWGVNGEDFLTLDPKTLKWSSDTPLATPVQSGWNQMKFMTPSLKDFKMYQCKPSL
MKLKKKKEEYLKGNLRPKIYIFGKPSHDGDAVSLRCYVSHKYLSGVRVRLTLDGVVVDNI
NISSPAPNMDGSVQIRL
>AM7_Z1 ENSAMXG00000005172 KB872010.1:338,894-344,058
IPKQSWMMEKMQEDYWDKGTLSRKSKEQWFKVNVDILMKRMNHNNTDLHVLQWRHGCEIE
ESNGQVKFLRGIDEYSYDGSEFLSFDDENMRWIAPVQAAQITKRKWDDVAILNQYTKGYL
EKECVDWLTKFMEYGKESLRKHSAPEVHAFEKKSVTDPGKLTLTCLATGFYPKDVKLCLR
KFTTSLPEHMLTSSGIRPNDDGTYQLRKSVEIHEDDKAKYDCYVSHSSLPEPVIKPWVPR
KSVSAEMGIYIGGGIGALVLLGLIAGVVVFVVKKKGKYRIYTLLRTNDKPPQGSDSTPSS
>AM8_Z1 ENSAMXG00000005186 KB872010.1:368,671-398,678
MGVSTAVCVLLLNAVFISTLAERHSLYYIYSALNKDVSLPGIYEFTALGLLDGREIDYYN
SKEQKKIPKQSWMMEKMQEDYWEKGTQSRKSKEQWFKVSLETVMQRMNHSNTDLHVLQWR
HGCEIDESNGEVKFLRGISEYGYDGSDFLSFDRQTMTWIAQVPAAVITKMKWDNVAILTQ
YTKGYLEKECVDWLTKFLKFRKESQQKDAPLDVQVFAKPSVSDSSKLTLTCLATGFYPKD
ATVIWRRSSSPLSEDLITSSAVRPNDDGTYQLRKSVEILGAEKDQYECYVSHRTLKEPVI
KKLGGAPINTDLPEELGMLPPF
>AM9_Z2 ENSAMXG00000005205 KB872010.1:401,043-407,580 pseudogene
MGESEVWVLLVFGVFLPSYGVSEPPVVSMFIDSPPPGPNLQSLTCVATGFYPKSLKMILR
ISGVPISEHQIKSTGVRPSGDGTYNLSKTLEVLENEKLNEYDCYVTHSSLKEPIIVHLGV
HWVFWLLLSVGLLIGIIVLVCCCLCVENRKDAYAIFVCSDLICSCLSLCDSV
>AM10_Z2 ENSAMXG00000005212 KB872010.1:408,806-417,593
```

MGESEVWVLLVFGVFLPSYGDEHSLYYLHTSQFKHNDSIHEFTALTLLDDVQIDFYNSTD
GVRTPKQDWLKEINGSDWERGSAELRDNGQYLNFLLNKQMMVFGHIESGDHILHWKYGCE
AVEHQNGSMTVFSFTNDYSYDGENFISYDWNLQLWSASVKQAYETEKKWNTEHHQNRVLQ
TCVDWLKIYLKKSPEIEKKPPVVSMFITSSPPGPNLDSLTCVATGFYPKYLKMILRISGV
PISEDQIKSTGVRPSGDGTYNLSKTVEVLGHEKFEYDCYVTHSSLEEPIIKNVRWIYWLV
GGVFPLAMIILIVCIHHQEKIMDACEEISWCCCCIEY
>AM11_Z1 ENSAMXG00000005228 KB872010.1:440,801-442,117 pseudogene
KTEVRYEYFFVLKGTLFIIVPKQSWMMEKMQEDYWEKGTQSRKSKEQWFKVNVDILMKRM
NHSDPDLHVLQWRHGCEIDESNGTVTFLNGISEYGYDGSDFLSFNNSNKVWVAPVPAAEP
FMKKWDAILNQYTQGYLEKECVD
>AM12_L ENSAMXG00000020806 KB872367.1:53,356-56,275, pseudogene
MFVLYSNICFSLFLCSVSGSHSLGLIIAYIKGETPFSEFSFTVLLDDITVGHFDSEKKMY
FSRGLSDVEEDDGVVDPNQARSMSSLMHSDLDERWLYVKHVLNQTEQCIKTLRGFLKKRS
NQVKRKVKPRVRIIQRRRSVSGWDGVTCLATGFYPRHINLTILRDGQPVPDHLITGVSCC
PMETGRIR
>AM13_U ENSAMXG00000012137 KB872374.1:21-6.096
MGEIIVLKVLLFLTVTVPLSSAATHYLQFLLTGVTPGTSFPEFSAVGQLDGEQGGYYDST
IRTVILKADWVKKSKDTEHWKMMTQRIFEDQQLVMERLGNIMKHFNQTEGVHTVQWMYRC
ELDDDGSKRGYMQCGYDGEDFLSLDLSSLSWIAPVPQALITKHKWEETGHVLFQKHFLLN
ECIGWLQKYVEYGRSTLERKVAPEVSVFQKDSSSPVVCHATGFFPKAVMISWQKNDEELN
EDVELRETLPNQDGTFQKRSILTLSPEELNRNQYTCVVQHEGLTKKLQVSDRRVLSGGES
VGIIVGAAMAVLLVIIGCAGVFISKKKTTSGFRLVSGSFSSDTSSLKLHALMQM
>AM14_U ENSAMXG00000012141 KB872374.1:13.042-14.417
GLHTLQTTIGCQLDDNGSRRGFAQFGYDGDDLLTLDLKSETWIAAVPQILPFINSSSSAQ
GTKQFILYQCIDCLLMYVEFGKSVLERKDPPEVSVFQKDSSSSVVCHATGFFPKAVMISW
QKNGEELNEDVELR
>AM15_U ENSAMXG00000012146 KB872374.1:19.497-33.140
MVVLKYLIFLTVTVHPASAVTHSLQYFYTGVTGFNFPEFTAVGQMDGEPFVYYDSNIRKM
IPKTEWIQKVVGDDPDYWDRNIQKLQGTQESFKVNMDTVKQRFNQTKGLHTVQMMYGCEL
DDDQTERGYRQLFLPNQDGSFQKRSILTLSPEELKNGDEYTCVVHHSSLKNGLVLLVSDR
RILHGVQDGRLSCVSVGKGVAVLLIVLIICFAGVLSWRKKSGFVPLSRSSSSES
>AM16_U ENSAMXG00000012154 KB872374.1:68.347-77.177
MKLLIFLFTTVHLTSPGSHSLQYSYTVVSPGTNLPEFTAVGQLDGEQIVYYDSNISEIIP
KTEWIKNVDDYAEYRNEETTRLQCDQQRAIDDLTRAVQFSNHTEGAGVHTFQSVYGCEKD
DDGTERGYKRNSFDGDDYISLDLNTETYNAVVPEAVPNKWLERADTVRNQKAYLLNECIE
LLQKYVKYGRSTLERKGGDGESVGVIIGVVVAVLLLVFIACFGVFYIWKKK
>AM17_U ENSAMXG00000012163 KB872374.1: 90.215-99.292
APGVSLFQKDSSSPVVCHATGFFPKAVMISWQKNGEELNEDVELRETLPNEDGTFQKRSI
LTVSPEELKNNKYTCVVQHSSLEKEKVPDIKFDPDGGMGGVIIGAVVAVLLLVLLAAIVG
VVVWKKKK
>AM18_Z1 ENSAMXG00000005091 KB872702.1:16,324-18,628
ENHFLYYIYIGLSKDLNVKGLYEFTALGLLDDREIDYYNSKEQKKIPKQSWMMEKMQEDY
WEKGTQSRKSKEQWLKDFLDTIIQQMNHSNQDLHVLQWRTGCEIEEFNGTVKFLRGISEY
GYDGSDFLSFDIENMRWIAAVPQAEPIMRQWDNETIVNQDAKDSLEKECVDWLTKFLDDR
KETLRNHSSPAVHGFVKKSVRDPKKLTLTCLITGFYPKDVKMSLRKFTTEIPEHLITSSG
VRPNDDGTYQLRKSVEISGDDPTDYDCYLPHSSFKKPVMKKWVNTKNSAGTALIGGAAVG
VMVILLGVVLTVLIKKRKNDTDRQNLHKGCRNFVYSREETSRSENIEH
>AM19_Z1 ENSAMXG00000005129 KB872702.1:26,615-47,799
MGVSTAVCVLLLNAVFISTLAERHSLYYIYSALNKDVDLPGIYEFTALGLLDDREIDYYN
SKEQKKIPKQSWMMEKMQEDYWEKGTQSRKSKEQWFKVNLEILMQRMNHSNQDLHVLQWR
TGCEISIYRGEVNFVRGISEYGYDGSDFLYFNDKNMSWIAAVPEAEPIQKKWNEMPILNQ
YTLGYQDTECVDWLLKFLDYRKEFCKLLKYLSIKMIIN
>AM20_U ENSAMXG00000005824 KB872935.1:26,693-36,705
MKLLLFLTVTIHLSSAACNRPSHLQFRYTENTDGETAEALLDGEQFVYYEKSNETVIPTT
EWMKKIENNDLVHWGRLTNKLKDGLQSARELVGTAVSSSNRNEGDHRVELMYGCKLDKGI
ESGYWKFGKYFLSLNLKAGAWTAENNESEPIKQKMEKEGNVQKAFLLNQCILWLQKYMDC
KSNNKTDVKDPRE
>AM21_Z1 ENSAMXG00000015427 KB873225.1:8,381-9,023
KHSLFYIYSALNKDVSLPGIYEFTALGLLDDREIDYYNSKEQKKIHKQNWMIEKMQKDYW
EKGTLSRKSKERWFKVNVDILMKRMNHNSTDLHVLQWRTGCEVEESNGEVKFLNGIDQYS
YDGSDFLSFDNSNSVWIAPVQAAEETKRKWDGVAILNQYMQGYLEKECVSWLKKFMEYGK

ETLRK
>AM22_Z2 ENSAMXG00000019749 KB873312.1:6,494-10,392
MGTNYPNPTILEKCIIVLGEHHFFTGKHSLLYLYTMESKNSKSNIYDCTAVTLLNYRQID
FYNSTDESRTAKQNWLKNISESDWKTSTEKLQNDRQLLNRLLDIQMSEFRHNQSDGHVLQ
WRQGCEVDRSLTVVKSISELAYDGIELIYFNCTSEMWLTSGKPTDRMLEKWNKDYRRTAI
KKCEECVRVLEMYLQYRTANTTDINTTPPAVHVFANKSVRDSRKLTLTCLITGFYPKDVK
MSLRKSGTSLPDHLITSSGIRPNDDGTYQLRESVEIQEDDKAQYDCYVSHSSLQTPVIKK
WDGKCRNCTSGRSWTGFIGVAVLVVAGILFICVLIC
>AM23_Z1 ENSAMXG00000004123 KB873343.1:1,019-15,294
ENHFLYYIYTGLSKDLNLKGVYEFTALGLLDDREISFFNGVQQLQDDIKKRTFFNEKQRR
TRLKKGTQSRKSKEQWLKDFLDTIIQQMNHSNQDLHVLQWRHGCEIDESSGEVKFLRGIS
EYSYDGSDFLYFDYENMRWIAAVPQAEPTKRKWDNETIVNQDAKRYLEKECVDWLIKFME
YGKETLRKHSPPAVYGFAKKSVSDSKKVSLTCLATGFYPKDVDLSVRKFGTSIPDHLITT
SGVRPNEDGTYQLRKSAEISDDPTDYDCNVHHSTIEEPAIKKWEQKCLTNPKTGSGLVLI
GGVAGGVIVILLIVLAVLFFVLKKKRMNGEYK
>AM24_Z1 ENSAMXG00000008310 KB873443.1:82-1,518
XEDYWEKGTQSRKSKEQWFKVNLDILMKRMNHSDQDLHVLQWRHGCEVEESNGEVKFLRG
ISEYGYDGSDFLSFNNSDRVWVAPVQAAEESKRKWDGVAILNQYTQGYLEKECVDWLTKF
MEYGKESLRNHSPPDVHAFATKSVRDPKKLILTCLATGFYPKDMEMGLTWFRTSIADHLI
TSSGVRPNDDGTYQLRKSVEILEDDKADYYSYVTHSSFKKPVIIKWGNYICDW
>AM25_Z2 ENSAMXG00000004532 KB873495.1:347-10,940
EHLLEYLYTMQSKNCTIYDCTAVTLLNDRQIDFYNSSSDKPRTAKQNWMKNISESDWKTI
TEKLQSDRSLLNKLLDIQMDESGHSNSDGHVLQWRHRCAGERQSDGSVTVSRSINEFAYD
EEELVNYNCTSNTWYNSDNQKKEEIEKLSAVLMKKCSQCEEMLKIFLQHSTFNITPSQSA
PPAVHVFSKKSVHDSSKQILTCLATGFYPKDVKMSLRKSGTSLPEHLITSSAVRPNDDET
YQLRKSVEIQEDDPADYDCYVNHSSLQTPVIKQWDKKCSNWTDGRSWTGLIIVLILVVLV
FICVLIYKKPQCVKYSSAPSDPGSPDELPSNSNGGVPNGK
>AM26_Z2 ENSAMXG00000019161 KB873598.1:6,446-7,932
SVSGGHVLHWRHGCEGEQSSDGSLIVLNSINEFGYDGEDLIQFNCTSKTWITSVIQKKGE
MKKWNTKLRLVKKCVQCEEILKMYLHYSTADITQSHTPPAVHIFAKKSVRESRNLTLACL
ITGFDPKDVKMSLRKSGTEIHDHLITSSGIRPNHNGTYQLRKSVEIQEDDSADYYCYVSH
SSLTEPVIKKW
>AM27_U ENSAMXG00000012522 KB873642.1:4,536-14,534
MEQLILLIVSPQLLVTSMTHSMQYFYTGVTPGINFPEFTSVGLVDGEPFIYYDSDIREDI
PKTDWIKKVLDYEAGFFDRQTQIHQGSQESFKVNVQTAMQRFNQTKGVHTVQWMYGCELN
DDGSVSGYSQYGYDGEDFVILDMKTLTWTAPTSEAVVTKHKWEGLGVGARFKAYMENTCI
DWLKKYVGYGRSTLERKVRPEVSLFSSDSSSPVVCHATGFFPKAVMISWQKNGEDLNE
>AM28_Z1 ENSAMXG00000010423 KB873648.1:4,727-10,381
DYWEKGTQSRKSKEQWFKVNVDILMQRMKHNNTDLHVLQWRHGCEIDESNGEVNFLRGIS
EYGYDGSDFLYFDNSHRVWVASVPAAEETKRKWDNLTQNTHDYLEKECVDWLIKFLEYGK
ETLRKHSPPDVYVFATKSVRDSKKLRLTCLATGFYPKDVELCVRKFGTSLPEHLLTSSGV
RPNEDGTYQLRKSMEISGDDPTDYDCYLHHSSLKKPVIKKWVKPNNGSGQGLIAGAVGGL
IVILLILLAVLFFVLRRRRMSGGAGYGQVAYAVPQAPDAQV
>AM29_Z1 ENSAMXG00000001165 KB874195.1:2-2,918
SKEQWFKVNVDILMKRMNHNNQDLHVLQWRHGCEIEESNGTVKFLRGIDQYSYDGSEFLS
FDDENMRWIAPVQAAVITKNKWDNVAILNQYTKGYLEKECVDWLTKFMEYGKETLRNHSP
PKVYAFAKKSLRDPRMLTLTCLATGFYPKDVVMRVRKFGTSFPEHLITSSAVRPNDDGTF
QLRKSVEIQEDDKAQFDCCVNHITLTKPIIVQWEYRYLLSSATAGITTCVLLIIIAIIVF
ICVYFLKKKSEIVTKPWTFVI
>AM30_U ENSAMXG00000009348 KB874440.1:2,653-7,379
MFLKMYSISQKLHVASEFITTHLQTSFTIVTPVISIPEFTAVCQVDEEQVGHYDSNNRTV
VITADWLKKDKNEEQWELPSEHVVHQETLFRTFLAAALEAFNWTEKELHTLQRTIGCQLD
DDGSKRGYDQFGYDGEDLVRLDLNSGTWITVVPQVLPLTQTSSTQLSIQFIQHTCIDWLQ
KCVKYGKSVLERKDPPEVSVFQKNSSSPVVCHATGFFP
>AM31_U ENSAMXG00000013144 KB881373.1:21-1,063
VHTVQRMYGCELDEDGTKRGYFQDGYDGKDFISLDLKTETWTAPVPQAFITKQKWDRTGD
AAFQKAYLETTCIEWLQKYVGYGKETLERKVAPEVSVFQKSSPSEVVCHATGFFPKAVMI
SWQKNEEDLNEGVELRETVPNQDGSFQKRSILTVSPEELKNNQYTCVVQ
>AM32_LΨ KB882095.1:3,016,286-3,019,577 pseudogene
GSHSLAMLATYIKGNTPFPEYSFTMVLDDITVGYYSSETKTFVS*AVKPRVRLIQRRCSV
SGWDGVTCLATGFYPRHINLTVHRNGLPVPDHLITRGG

>AM33_S ENSAMXG00000017444 KB882192.1:1,855,138-1,867,212
MEKMKESYIHCVLLLLLLLHLLQLSGGEVNSITAYFIGIQGLNLPDYMERIAVNDVTMFYY
DSSMKDEVSCPDWLNTSSGKQHWMDINLISLHNKHSMATALKSAILQFNQTASSSDVNIY
QGYGRCSVYPNGTLKALLTHAFNGKDFLTFDVDRKSYIASVPQAVIYKRQREANPVWLEI
MASFYKKTCFERLKMFLQHASVHITKKVPEVHLFKSFKSGSSVLACHVTGFYPKEVQVEW
IGAGLQPVDGEVIEVLPNGDGTYQTRRSVIRPEENPEKHSYSCVVQHSSIAGNITKTWVA
EEHSLLAVWISLVCILVIIGTGLVLRKFCRCGQRDTGI
>AM34_S ENSAMXG00000017459 KB882192.1:1,870,383-1,874,078
MKKLYCLFFCFHLLQISTGGEFHSFSFFFIATQGLNFPSYIERVTVDDVTMFYYDSSMTV
EPLCPQWLNTTEGLQQWKNMNDRAKYNNHYLSSALESIVKQFNQTETNIYQGYSHCNIFP
NGTRKAAFTQAFRGKDFFSLDIDRKTYVASVPQAVVYKRQREANTVLLETVVSFYRTTCF
ERLKMFLEHAPEVRVKKGWIFERAESSSSVLTCHVTGFKPKQVQVEWIGAGLQPVDGEIT
DMLPNGDGTYQTRRSVIRPREENPEKHSYSCVVQHSSIGGNITKTWVTETRIRMGVLASL
VCIVLAVIGCGLVFRQFCRTKSE
>AM35_U ENSAMXG00000006709 KB882234.1:282,644-295,597
MWCLHPKKRWTDRIRTVMLVAVHLLSVFSESSGVHFHQATFIYTTRTTILPKCMATTFTD
GLQISYYDSNSKTFKSSWGWVRESMNKSWELFKNSIYRSMEDLNMTSTLLWSTLEVRKIF
QWISNCTWDDKSGDCSGYEKYGRNGKDFITLDVKKRSYISHTPEAIHITDLWNKDKARLE
SVIHHYTSACAELVKISVPKLQRAVPPEVYLWQKNSSVVCLATGVYSKTTKVMWQKNSED
IHENVDEGETLCNGDGTFQKRSSLRGEA
>AM36_U ENSAMXG00000006716 KB882234.1:294,383-308,558
MIYVMILIIGSVRAAQKIYIYSVKYTAAVGISGLPEFTATSFLHGKQIDYYDSKDHILLP
RKDWAKKILGVPYWEKTTRLRFSESVQLQQELSSVMESSGHTEGMHTFQRMYGCLWDGKT
GESDGFDVYGYDGENFLSLDVNSRKFTALTPQAKSIVQEWNRNTTRLEVLQLYYKLECVA
WLKTFFPEKKGSILKSIKIIPTFNHYLKRSYPAAVMCHVRDLSSSSVRMSWQRNEKDFND
GFVVIGDTLPSGDGTFQKTIYLYVGVEDLMRNQYKCVLE
>AM37_U ENSAMXG00000006739 KB882234.1:321,252-330,732
MTRPIMLIQQEEQLYQTEKYVLSTMYTVTQVSCQPEFTAVTVVNEQQITRFDSEHNMLIP
TQDWVKEALKDYHWNNYSQIHLSEFARLREELNATMKSLGHTDSVHTFQRRSGCEWDDKT
SRVQGFDEYGYDGVDFISLDLDQMKYVPRTALAKATAERWNSDDNLLKFQRHYYNTDCSN
WLKRIGRVHLKKTGNITFYQKKAFSPVVCHVTGFCPKAMKISWRKNGKDTTKNVSIGDIL
PDGEGTYIRDISIFSDELDVSKFTCVVGDSFKNLSRESLNKPGNSFNTGPNVAVISSTVI
LFTLGFSITVGFFYFRRRRPENFKHV
>AM38_S ENSAMXG00000003502 KB882301.1:1,316,715-1,326,631
MCAEAMKKSNLLCLLLVYLQQDSGSLNLCISVVKTSGFNTIPEYIQKNTLNDVTIFHYDS
DMDSKMPCPDWINSTAARREWNYMQFQTDRISYHITQGFESAVQQFNRTGSLADRNIYQA
RGCCVLYPNGTSASSLTHTFNGKDFIGFDFDRKTFVAPVHEAVVYKHQRETRSSLFNVLH
FFGTQCVQNIQLFKTAPAILERKVPEVRIFQQKKRAGSVTVTCHVTGFYPREVQVFWLGS
DLQPVDEGVTEILPNGDGTYQTRKSVIVPEEDVGKQNYSCVVLHISIPNNITTVWVGQKC
GGFALWIPLVCISLLACGTGFGVWWRCQTRDAVI
>AM39_S ENSAMXG00000003509 KB882301.1:1,336,985-1,396,062
MKYICILLLLLQQVKTDDLRYDSCAYYMVSKGLGLPEFSERRVLNDVTVYHHDSSLDSKM
PCPDWINTTAGKEHWKIIYHWTDYNKYVGTLGLQSATEQFNQTGSLTDGNIYQAFGCCSL
YPNGTYRTLLAHAFNGKDFASFDFQTKTFVAAVPQAVLYKTLREKDPANMEEIATLYKKT
CLERLQILKEAPRVNIKKVPEVRIIEKKRTGSVTVTCHVTGFYPREVQVIWLGSDLQPVV
KGVTEALPNDDGTYQTRKSVIVPEEDVGKHTYSCVVLHISVPNNITTVWEVKAGGVAVWI
PLLCISLLASVIGIGVWWRCKTRDAVI
>AM40_S ENSAMXG00000003518 KB882301.1:1,343,262-1,346,695
MKNANLLFLLLYLQQVTTDLRYDNCCYWMVSKGYDLPEFTERRVLNEVTVYYHDSSLKSK
MPCPDWINTTAGIYEWGQIQYWSNHNRFVGTLGLQSAINQFNLTGTLTDRNIYQGFGCCS
LYPNGTYSAFLNHAFNGKDFTSFDINSKTFVAAVPQAVVYKTLRESDQTTINYVASYYQK
TCLDRLKILKEAPRVRDKR
>AM41_S ENSAMXG00000003529 KB882301.1:1,350,690-1,351,925
IRYDSCAHWTYSKGYGLPEYTERRVLNDVTVYYHVSRQKSKMPCPDWINTTAEKELWGTI
HDWTDHNIEITTLGFQTATQQFNLTGSLSDRNIYQVTGCCSLYPNGTYTAFMNHAFNGKD
FTSFDINTKTFVAAVPQAVRYKTLRESQPLTIDEVAEFYKKTCLERLKMFKQAPEIHRRK
DPNVWILQQKKRAGSVTVTCHVTGFYPREVQVFWLGPDLQPVDEGVTEILPNGDGTYQTR
KSVIVPEEDVGKQNYSCVVLHISVPNNITTVW
>AM42_S ENSAMXG00000003539 KB882301.1:1,398,925-1,401,916
IRYDNCAYWTFSKGFGLPEFTERLALNDVTVYYHDSSLKSKMPRPDWINTTAGKDMWRII
HDWTENNRYGGTLQFHTATQQFNLTGLLTDQNIYQASGCCSVYPNGTYTAFLTYAFNEKD

FASFDINTKTFVPAVPQAVFYKNLRERDSLRVDMAVKYYKKTCLERLKILQQDPRVLIRK
VPEVQILEQQKKKSGSVTVTCHVTGFYPREVQVFWLGSDLQPVDEGVTEILPNGDGTYQT
RKSVIVPEEDVGKQNYSCVVLHISVPNNITTVWERACGGVWIAILGCILLSAAAYISLKN
FKHHCLVI

1b. Cavefish (*Astyanax mexicanus*) Expressed translated matches:

>FO227761 *Astyanax mexicanus* whole embryos and larvae cDNA clone ARA0AAA41YA01, cDNA sequence AM2_Z2 match
REEKWSRIFNAVKKCLHCEEMLKIYLQYKTTDITPSNTPPVVHIFAKKSVRDSRKLILTC
LITGFYPKHVKMSLRKFTTEIPDHLITSSGIRPNDDETYQLKKSVEIQEDDPAYYDCYVS
HSSLTEPVIKQWGKYNPTLLTYSSVSSAEKREEFV
>SRR639085.396984.2 SRX212201 AM4_Z2 match
LLTLAVTQFNSSSDKPRTAKQNWLKNISESDWKYSTEKLQYDTQLVNKLLDTQINEFRNT
HSDGHVLQWRHGCEGEQSSDGSLTVLNSINEFGYDGEDLIQFNCTY
>SRR639085.617728.2 SRX212201 AM5_P match
MRVLLLCVFCFTGETASAEKHSLQYLYTLRSAPEDDTEFEITTVFDGLIISHCKSPRFRD
HSREDWISQTFTNAE
>SRR639085.760083.2 SRX212201 AM5_P match
GIIQRERSCTEDDSVVRMSDSWGVNGEDFLTLDPKTLKWSSDSPLATPVESAWNQMKFMI
PYTRR
>SRR639083.123788.2 SRX212200 AM8_Z1 match
SYDLGCHSVKGYLEKECVDWITKFLKFRKESEQKAAPLDVHVFAKPSVSDSSKLTLTCLA
TGFYPKDATVIWRRSSSPLSEDLITSSAVRPNDDGTYQLRKSVEILGAEKDQYECYVTHR
TLKEPVIKKL
>SRR639085.450509.2 SRX212201 AM19_Z1 match
STDLGCHSVKVKMGVSTAVCVLLLNAVFISTLAEKHSLFYIYSALNKDVSLPGIYEFTAL
GLLDDREIDYYNSKEQKKIPKQSWMIGEDAGRLLGKRNPVSQE
>SRR639083.344158.2 SRX212200 AM33_S match
RHWMDINLISLHNKHSMATALKSAILQFNQTASSSDVNIYQGYSRCSLYPN
GTLKALLTHAFNGKDFLTFDVDRKSYIASVPQAVIYKRQREANPVLLEIMASFYKKTCFE
RLKMFLQHASVHIS
>SRR639083.120834.2 SRX212200 AM33_S match
MEKMKESYIHCVLLLLLLLHLLQLSGGEVNSITAYFIGIQGLNLPDYMERIAVNDVTMFYY
DSSMKDEVSCPDWLNTSSGKRHWMDINLISLHNKHSMATALKSAILQFNQTASS
>SRR639085.558865.2 SRX212201 AM33_S match
VSVLACHVTGFYPKEVQVEWIGAGLQPVDGEVIEVLPNGDGTYQTRRSVIRP
EENPEKHSYSCVVQHSSIAGNITKTWVAEEHSLLAVWISLVCILVIIGTGLVLRKFCRCG
QRDTGI
>SRR639083.339410.2 SRX212200 AM34_S match
ERLKMFLEHAPEVRVKKVPEVRLFERAESSSSVLTCHVTGFKPKQVQVEWI
GAGLQPVDGEITDVLPNGDGTYQTRRSVIRPREENPEKHSYSCVVQHSSIGGNITKTWVT
ETRIRMGVLASLVCIVLAVIGCGLVFRQFCRTKSV
>SRR639083.115309.2 SRX212200 AM38 match
SKMPCPDWINSTAARREWNYMQFQTDRISYHITQGFESAVQQFNRTGSLAD
RNIYQARGCCVLYPNGTSASSLTHTFNGKDFIGFDFDRKTFVAPVHEAVVYKHQRETRSS
LFNVLHFFGTQCVQNIQLFKTAPT
>SRR639083.252767.2 SRX212200 AM38 match
SKMPCPDWINSTAARREWNYMQFQTDRISYHITQGFESAVQQFNRTGSLAD
RNIYQARGCCVLYPNGTSASSLTHTFNGKDFIGFDFDRKTFVAPVHEAVVYKHQRETRSS
LFNVLHFFGTQCVQ
>SRR639085.567835.2 SRX212201 AM40 match
STDLGCHSVAVVYKTLRESDQTTINYVASYYQKTCLDRLKILKEAPRVRDKRVPDVRILE
PKKNAGSVTVTCHVTGFYPGRFR
>SRR639085.207547.2 SRX212201 AM42 match
QFNLTGLLTDQNIYQASGCCSVYPNGTYTAFLTYAFNEKDFASFDINTKTFVPAVPQAVF
YKNLRERDSLRVDMAVKYYKKTCLERLKILQQDPRVLIRKVPEVQILEQQKKKSGSVTVT
CHVVRGLPPRRFEVFVARFRSSACG
>SRR639085.737172.2 SRX212201 AM42 match
SGSVTVTCHVTGFYPREVQVFWLGSDLQPVDEGVTEILPNGDGTYQTRKSVIVPEEDVGK

QNYSCVVLHISVPNNITTVWDAVI

2a. Zebrafish (*Danio rerio*) Ensembl genes:

Published zebrafish genes and locus definitions can be found in McConnell et al.2014 (U lineage, main text reference 25), Dirscherl & Yoder 2013 (Z lineage, main text reference 34) and Dirscherl et al., 2014 (U, Z, L lineage, main text reference 38).

```
>DR1_ZEA ENSDARG00000001470 Chr.1:47,613,228-47,619,236 Dirscherl and Yoder 2013 KC607850-KC607854
MAVFAALFSVVMLFTVVPAWTEKHSLYYIYTALSRPVHLPGIYEFTAMGLLDDRQIDYYN
SQEQKKIPKQHWMKEKMQEDYWEKGTQSRKSKEQWFNVNLKILMERMRHNNNSDVHVLQW
RHGCEIDSQGNDVRFSKGIDEYSYDGRNFLAFDDADSQWVAPVEEALPTKRKWDNVPILN
QYTKGYLEKECVDWLNKFREYGDQELREGSPPDVHVFAKKIISGKAKLKLTCMVTGFYPK
DVILTIRKYRTALSDNEVESSGVRPNPDGTFQLRKSTNIYEKAEYDCYVAHRTLKEPIIK
KWDGECQDCSSGTPIGTIFGALIGVLLVLAVIGGAVYFLANTRMGWRNAL
>DR2_ZDA ENSDARG00000086877 Chr.1:47,626,631-47,632,322 Dirscherl and Yoder 2013 KC607843-KC607849
MAVFVVLLLAVMLLDVEPATTEKHSLYYIYTALSRPVNLPGIYEFTAMGLLDDRQIDYYN
SQEQKKIPKQHWMKEKMQEDYWEKGTQSRKSKEQWFNVNVNILMDRMRHNKSDVHVLQWR
HGCEIDSDDDVRFSKGIDEYSYDGENFLSFDDADSQWVAPVDAALPTKRKWDNVPILNQY
TKGYLEKECVDWLNKFREYGDEELREGSPPDVHVFARSIINGKAKLKLTCLATGFYPKDV
YLTIRKYRMALSDKLESSGVRPNHDGTFQLRKSTYIYEDEKAEYDCYVKHRTLGAPIIKK
WDGKCSDCSKVTFLGMIVGAIIGAVLVLTVIGLVILVLTRKKAPKKPFYKNGIGDNDPSA
IPLNNNQHIEPSVKESKNKPTAEGQSTSGDLKS
>DR3_ZCA ENSDARG00000069471 Chr.1:47,637,895-47,648,897 Dirscherl and Yoder 2013 KC607835-KC607842
MAVLAVLFSAVMLLSVVSVRTEKHSLYYIYTALSRPVDQSGIYQFTAMGLLDDRQIDYYN
NIDQKKIPKQHWMKEKMQEDYWEKGTQSRKSKEQWFNVNVNILMDRMRHNNSDLHVLQWR
HGCEIEIQGNEHRFSKGIDEYSYDGRNFLSFDDAESQWVAPVDAALPTKRKWDNVPILNQ
YTKGYLEKECVDWLNKFREYGDQELRDGSPPDVYVFARRIISGKIKLKLTCMATGFYPKD
MILTIRKYRTTLPDNDLDSSGVRPNQDGTFQLRKSTNIYEDEKAEYDCYVNHRTLKEPII
VRWNGEYLSEPPIAIIAAIIGVLILLVAIGVTVWILKKKNIIGNKDEKRSMPNGSANYGR
GSSA
>DR4_ZBA ENSDARG00000036588 Chr.1:47,652,531-47,659,103 Dirscherl and Yoder 2013 KC607831-KC607834
MGSFAVLFSAVMLVAVVPAWTEKHSLYYIYTALSRPVNLPGIYEFTAMGLLDDRQIDYYN
SEQQKKIPKQHWMKEKMQEDYWEKGTQSRKSKEQWFNVNVNILMERMRHNKSDVHVLQWR
HGCEIESQGNDVRFSRGIDEYSYDGRNFLSFDDAESQWVAPVEEALPTKRKWDNVPILNQ
YTKGYLEKECVDWLNKFREYGDKELREGSSPEVHVFAKRIINGKIKLKLTCLATGFYPKD
VILNIRKYRITLPDNEVESTGVRPNEDGTFQLRKSINIYEDEKAEYDCYVSHTTLKEPII
KKWDGECLDGPESGSPIGIIAGAIIVVLVVLAAIGGAVYFLRKRSGNNNVKPSSVPTISG
NKDEKCSMLPGSDDSGQGSSDGSSKSSPTNSQEKMDIV
>DR5_ZFA ENSDARG00000088022 Chr.3:502,131-512,626 Dirscherl and Yoder 2013 KC607855-KC607865
MIMDVKVLTLLCVFLLYGALPLCRAEKHSLYYIYTGLSRPVDLPGIYEFSAMGLLDDRQI
DSYNSREQRKIPKQQWMKEKMQEDYWEKGTQSRKSKEQWFNVNVHILMDRMRHNRLDLHV
LQWRHGCEVEIKGSEVKFSKGIDEYGYDGENFLAFDEAESQWVAPVEEALPTKRKWDSVP
ILNQYTKGYLEKECVDWLNKFREYANEELRNGSPPEVHVFAVKSISDKTKLKLTCLATGF
YSKDTMLVIRRNRLPEKKTESTGVRPNHDQTFQLRKSVEIEQDETDEYDCYMTHRTLKGP
VIARWDGKCKDCLPNLNWIWVVAGAVLMLGVVALLLVLLKKKIIDLRQRLSGSQSPLYQI
QADSESEDYNH
>DR6_ZGA (ENSDARG00000092162) Chr.3: 1.070.329-1.076.000 pseudogene Dirscherl and Yoder 2013 AJ420975
EKHSLYYIYTGLSKPLDLPGIYEFSAMGLLDDRQIDSYNSRDQRKIPKQQWMKEKMQEDY
WEKGTQSRKSKEQWFNVNVNILMDRMRYNKSDLHILQWRHGCEVEIQGSEVKFSKGIDEY
GYDGENFLAFDEAESQWVAPVEEALPTKRKWNNIPILNQYTKGYLEKECVDWLNKFREYA
DEELRKASSPDVYKLTKKSTKDETKLKLTCLATGFYDKDVMLNIRRNCLPEDETESTGVR
PNHDQTFQLRKSVEIKEDQIDEYDCHLTHRTLKNPVTATRAKQR
```

>DR7_ZHA (ENSDARG00000092162) Chr.3:1.076.000-1.080.000 Dirscherl and Yoder 2013 XM_001919256.3
EKHSLYYIYTGLSKPLDLPGIYEFSAMGLLDDRQIDSYNSEEQRKIPKQQWMKEKMQEDY
WEKGTQSRKSKEQWFNVNVNILMDRMRHNMSDLHILQWRTGCEVEIQGSEVKFSKGIDEY
GYDGENFLSFDEAESQWVAPVKEALPTKRKWDNVPILNQYTKGYLEKECVDWLNKFREYA
DEELRNGSSPDVYKLTKKSTKDETKLKLTCLATGFYDKDVMLNIRRNCLPEDETESTGVR
PNHDQTFQLRKSVEIKEDQIDEYDCHLTHRTLKNPIIVTQ
>DR8_ZIA (ENSDARG00000092162) Chr.3:1.1083.984-1.093.108 Dirscherl and Yoder 2013 AJ420977
MMSGGVASGICALLCVFLLCGDLPSAQGEKHSLYYIYTSLSKPLDLPGIYEFSAMGLLDD
RQIDSYNSEEQRKIPKQQWMKEKMQEDYWEKGTWSRKSKEQWFNVNVHILMERMRHNMSD
LHILQWRHGCEVEIQGSEVKFSKGVSEYGYDGENLLYFDDAESRWVAPVEEALPTKRKWD
NIPILNQYTKGYLEKECVDWLNKFREYADEELRKASPPDVYKLTKKSTKDETKLKLTCLA
TGFYPNDVMLNIRRNCLPEDETESTGVRPNHDQTFQLRKSVEIKEDQIDEYDCHLTHRTL
KNPVTATRAKQ
>DR9_ZJA ENSDARG00000074765 Chr.3:1,109,672-1,128,564 Dirscherl and Yoder 2013 KC607866–KC607869
MTSFDRSFTLLYLCLFHVIMSSFRAEKHSLYFIYTGLSRPVDLPGIYEFSAMGLLDDRQI
DSYNSEEQRNIPKQQWMKEKMQEDYWENRTQSRKEKQLWFYDNVHLLIDRNRQSTSVLHV
LQWRHGCEVEKQGNEHSFNKSIDEYGYDGEDFLYYDDAESRWVAPVEEALPTKRKWDNLP
ILNQYTKDYLEKECVDWLNKFREYADEELRKASPPDVFKLTRKSTKDETKLKLTCLATGF
YPNDVMLNIRRYRVYLPEQETISTGVRPNHDQTFQLSKSVEIKEDQIDNYDCSVSHRTFK
QPIIIKWDGTDLDGIYTGHPPETVPVVIGSVLIFLAILGVVVFLVMKCGEQDSSSTDTTE
CTSILLGFIADHLNKELTEGENW
>DR10_LCA ENSDARG00000055813 Chr.3:23,762,869-23,765,495 Dirscherl et al.2014 XM_001340377.4
MMYLVLAVCILSASVFSQSGSHSLWVFATFLTGDISVQFPEFSAVVMLDDIVIGHYNADD
RSFVPSTVQESVNITEQITISTVCKGIHEGMKTKAYYLIDYLNHTRGLHVQQRLVGCELL
QNEPGQMMTLEAFNGESGFERRYDIQGDQQTHWKWPVIKSRAQLEYDAWLYAHFYRPLCI
SQLRKYLKKEKKRVMARVKPRVRVIQRTCSKTGKIQMTCLATGFYPRHINLTLLQDGQPV
NEERVMGGELLPNADGTYQMRKSVELSAEEQRERRTYTCTVNHLSLDNKLDISIEPGLDP
VIIFPSVLLLLCVFGVLAGFLMWRKKYKQPEQVTYTPTSATDQTTEQSQL
>DR11_LBA ENSDARG00000016227 Chr.8:47,093,433-47,098,053 Dirscherl et al.2014 XM_001920752.3
MKTMLFLLYLLSCLTFTDAGFHSLLVLATYVDGQTPFPELSVVVMLDDVQIIYYDSDTWR
VFHRSPSDSKYYDEDQSDADAVFHDTYDEMKYRVLHLKNQLNHTDGITVLQRIVGCELFN
DKPGIYHLWDAHDGKTIEKFTFNIYNHEFQLKNQWFRTWDQVMIQQKRIVHENIYYPVCI
KVLRRYLNVEKNSVMRKVKPRVRLMKKKLPDSQGLQISCLATGFYPRHINLTLFRDAEPV
DDDQIIGGEILPNGDGMYQMRKSLIVSKEELDEGHEYTCTMKHLNLDNKLDIVFDVSGTV
PGCFSVSVVISVLVFMCVSVFIITKLIMRRKRQDTGRGSEKCDYSPTISSSQDEI
>DR12_LAA ENSDARG00000016056 Chr.8:47,110,753-47,116,956 Dirscherl et al.2014 XM_005167247.1 and NM_001017904.1
MFLRMFCFVCLLPFIGVVNAGSHSLMALATYIVGQTPFPEFSVVVMLDDLQLAYYDSIGW
KTIYRSGSDSKYYDEEQSDAGIVFRDMFYDMKDRAFYLKDHQNHTDGVHVHQRLVGCELL
NNEKTGPLHYWDAFGGQNMEEFIFDTEKHTIQVKMPWVITWDQLKRLHENFMYDNVYHPI
CIKTLRRYLNMEKKNVMRKVKPRVRLMQKKLSDSQGLQISCLATGFYPRHINLTLFRDAE
LVDDDQITGGEILPNGDGTYQMRKSLIVSEEELQKGHKYNCTANYLNLDNKMDIVFDVAE
SDPGSFSVSVVMGVLVFVGLSVLSITALIMRRKRRDTGSVSGTSQNQYVYAQTSVQDAT
>DR13_UCA ENSDARG00000092731 Chr.19:7,666,170-7,674,017
MEYILLLVVCLPYVDGATHTWNAYYTATTGLSQFPEFVAQNLIDGQLMGYFDSKTNCFKS
QFQWMEDKLGTAYDEQQTNILQRHTAKFKNNIKVAIERFNQTQGVHTLQEIYGCEWDDQT
GNLNAFRQYGYNGEDFLTLDFMEMRWITPVQQAMITTQKWNNDRGFIESDMNYFRSECIE
WLQKYLEYGKSSLMKTVSPQVSVLQKYYFSPVVCHATGFYPSGIKISWQKNGQDHDEDVE
LGELLPNADGTFQKRSTLNVKPEEWKNSKFSCVVEHQGERILTEDEIRTNNPSATIGIII
GIVLSAVLLLFIAVAGFVVYWKKKGFKPVPANPVNSDNDSGCGSGSDSVSHKS
>DR14_UBA ENSDARG00000075963 Chr.19:7,695,390-7,723,636 and AAH74095.1
MQSLIGLLLVVCLQYASGATHSWKAYYTGTTGLTEFPEFVALNLIDDQLMGYFDSKTNRF
KSQFQWMEDNLGKEYDEQQTNILLGYPEVFKNNIKVVMERFNQTQGVHTFQFMYGCEMDD
DGNKQVHWQIGYDGEDFISLDKKTLTWTAANSQAMTTKVKWDSTGAEANYWKGYLENECI
EWVQKYVGYGKDTLERKVSPQVSLLQKSSSSPVVCHVTGFYPSGLKISWQRNGQDHDEDV
ELGELIPNEDGTYQRTSTLNVKPEEWKKDKFSCVVEHQSKTINSILTEDEIRTNNPTAPL

GIIIGIVVAAVLLVAIAVAGFVVYRRHKGFKPVPQNTSDGGSDNSSRT
>DR15_U ENSDARG00000039164 Chr.22:5,239,117-5,243,568
MSIMKFIIFFFNLPFVYSELHTFVVTYTAMGNQTFFAAATLDGQQIGHYDKNTKTLVPKQ
DWNEQIKSKTPWKVDTLIETVQRTYNNNMHDLIEQFPQSNGAHTYQGRHGCSWEDKTNYH
DEFHDYAYDGDDFITLDVKKIKYRASVSKAKYIVKKWNNDREQNKTLKEYYKLGCIYWLI
EFLVFTKNAFGGTAPKMFLLQKSPNSDVKCHVTGFYSNNIVILWMKNGQEVSNSALLKSG
EILPNEDGTFQRTVTLRVSLQYWRKEQYTCVVRYMEKTIRRNLTEEEIKSNYSEMSITAS
DSSTSILVPIIVVVLVVSLILWWMSCHQNCRLGQCFHLENAN
>DR16_U ENSDARG00000059039 Chr.22:5,243,817-5,258,961
MKIIVVFICIPFVCSGYHSLITTYTGIRGASETPIFIAVAILDGEQIDYYDSVTNKLIPK
QDWMKEYASEEIWKEDCKIRGDVHQIYRSNINVLMQRFNQSNGVHVYQRMYGCDWDEETE
ESHGFDQYGYDGEDYVMLDMREIRYITPVQQGEITVQKWNSNREQLKTLQHYYKYECVYW
LKQFLGLRKADLEIRDPEVFLLQKNPSSPVECFATGFYPSGVIITWLKNGQDHGENVELR
ELKPNEDGTFQRSSSLHVSQEDWKKSLYTCVVEHQRMTIQKSGDEIKSNSSSEVFITPER
VLAILLAALVICLCICYLVYPKKLKPYQPVNQNEEMYEYIVKNE
>DR17_LMA ENSDARG00000086127 Chr.25:11,063,589-11,066,365 Dirscherl et al.2014 XM_005174300.1
MRFLCFLCLLSLPTAVVTDSHSLWFLVTYIEGETQFPAFSVVYMLDDITVGYYNSETKTC
VPRGNTTNEDEEVDLIGYYLNEYYPPIVEKFIGLFKNKTKGPVVHQLLTVCELPDKDGVG
QMITKIAFEGSTTDEMHFIDDTLTFQGMEKVTKLHLEMFRWFHAMAYKRGKLILEKYLKK
RATQGKRRVKPRVRLIQKASDSGFCVSCLATGFYPRHINLTLLRDGQPVSDHEVTGGDLL
PNGDGTYQMRKSLEISAEERQKHKYSCSAKHLSLDNKLIATLEFDHGEPYKSVIPSVLAV
LALMLVFGAAAAVWKRRRTGTMRFKINL
>DR18_LOA ENSDARG00000087161 Chr.25:11,071,636-11,074,578 Dirscherl et al.2014 XM_005174294.1
MKWKYTESVVLALSLVFPLASYGLQSLSLLSTYIKGETQFPKLSGTAILNDFIVGYYNNT
YIPRGNTTNEDDVIDADQTKTLADYVYGSFLKRSSLLSQNNQNTSLDVYQKLVVCELLDS
DTPGKMFFRDAARGSTIDEILYTNNTFMYTVIGNVSQELIKPHLEAFKLDFSTLFYPVCI
KTLKTYLKKRKSQVNRREMPNSRLFRKASDSGGFRVSCLATGFYPRHINLTLLRDGQSVS
DHEVTGGDLLPNGDGTYQMRKSLEIKAEEREKHKYSCSVKHMGEEFHIDLADPHRIHTWI
VVAVPLVCATALVGLVVFIWKSGQKDRTAGQRERGEHDGRVL
>DR19_LHA ENSDARG00000046057 Chr.25: 11,082,589-11,085,195 Dirscherl et al.2014 XM_005174295.1
MGQILLFFVFLLPTAAPKGSHSLWMLVMYIKGQTAFPEFSYVMMLDDVSVLYYNGDTNSL
IARGNTKADDDVFNLNALPIIHDHLQSSFEDRWTLATTHLNKTDGVFALQQLVACELQDD
GEPGRMILRNAFGGSTTDQLLFVDKKFTYHDSFNVSTHVLHAHHDYNKYLCEKLLQPFCF
QTLKGYLVKRRNQINRKVKPEVRLIQKANSDSGGFRVSCLATGFYPRHINLTLLRDGQPV
SDHEVTGGDLLPNGDGTYQMRKSLEISAEEREKHKYSCSAKHLKIDNKLDIYLGIYFLSD
FDPEEPFPLVLILLSLVSVFITGVII
>DR20_LPA ENSDARG00000051710 Chr.25:11,250,338-11,263,782. Not identified in the Dirscherl et al.2014 study.
SHSIRMFATYIKGKTPFPELSGVVMLDDIRVLYYNGVTDSFLARGNTTAEDDVFDPDDLE
NIKGFIKSEFSFLNAQNTFTKTDGIFVSQILAMCELKDDGEPGQMIGQDAFERLTIFTVL
YADNKCTIDINLNISQEQKEKIIEGVKNYYRNLIQPFCYKTLKVYLKKRKDQVNRKVEPK
VRIFHKANLDFGGFRVSCLATGFYPRHINLTLLRDGQPVSDHEFTGGDLLPNGDGTYQMR
KSLEIRAEDSEKHKYTCSFKHLDKEWHIDLAEPHRNTIWIAVSVLLVCAIIVGLAMLIWK
RYQTARQRENEPNNHQTTHMN
>DR21_LNA No Ensembl gene ID Chr.25:11,276,832-11,277,543 Dirscherl et al.2014 XM_005161519.1
GQMITKSAFRGLTVDELHFLDGKFTYQGSLNYTKLEIKPYLDLAMWRFETLYYPACIE
TLKSYLKKRGTQVNRKVKPRVRLIQKASDSGWLAVSCLATGFYPRHINLTLLRDGQPVSD
HEVTGGDLLPNGDGTYQMRKSLEIRAEERQKHKYTCSAKHLGLDNKLINLNQANHINQ
>DR22_LJA ENSDARG00000096830 Chr.25: 11,280,398-11,285,009 Dirscherl et al.2014 XM_001920322.3 and XM_005161517.1
MSYVGNMIIILFLCVLLAPTTALTGSHSLWMLATYMKGEPYSEISITFMLDDITVGHYNS
KTERYIARHNTTNEDELTWNTFYVARHDLHTFFVRKLKFKNHTESPQLYQMMSHCELQGN
NKPGQMVSKIAFSGSTTDEMSIYENKFTYQSQKEMPSLALELIKWRHESVSYPTCISTLR
NYLKMRQTQVNRKVKPKVRLFQKANSDSGGFRVNCLATGFYPRHINLTLLRDGQPVSENE
VTGGDLLPNGDGTYQMRKSLEIRAEERQKHKYTCSAKHLSLDNKLIVTFDFDPGEPFKSV
IPSVLIILSLVLVFITGVVIYKCRKRRAVSSKRDYISASTSEESTGSTVTQPQQEAT

>DR23_LEA ENSDARG00000051711 Chr.25:11,300,140-11,301,469 Dirscherl et al.2014 XM_001920310.3
RGNTTAEDDVFETNVLLKISNNIQLSFMEKWVVGTNNINKTHGVLVLQRLVVCDLKDDGE
PGKMITRDAFRGSTTDELQYFDKTFTYQGTLNVSDNLLKIHLEASKRNHEYLYQPYCIKT
LKGYLKKRTNQVNRKVKPQVRLIQKASDLGWFVVSCLATGFYPRHINLTLLRDGQPVSDH
ELTGGDLLPNGDGTYQMRKSLEIRAEEREKHKYSCSVKHLSLDNKLDVDFVVLVADFDHG
EPFKSLIPSVLVVSTLMLVFGVAAAVIARKRRCSGTT
>DR24_LFA ENSDARG00000051712 Chr.25:11,314,469-11,317,472 Dirscherl et al.2014 XM_001920284.3
MDKILLLFLFLLPTSAPKGSHSLCLLATYIKGPSPFPELSGVMMLDDIPLLYYNGDTKTF
FMRGNTTAEDNVFDANAFLSIIGHIQSSFVDRWGLASRDLNKTDRIFTLQQLVLCELSED
GEHGKMISRDAVEGTTTDELQHVDHKFTYKHTLNVSAYLIDFYLELTKSLHKTLFQPTCF
KTLSGYLIQRRNQINRKVKPKVRLFKKELSSGFIVSCLATGFYPRHINLTLLRDGQPVSD
HDVTGGDLLPNGDGTYQMRKSLEIRAEERQKHKYSCSVKHLSLDNKLHVDLDFDHSKPFQ
SVIPSVLTVLALLLVFGVAAVIWKRKCRDSVKCGYSAASTSVENMETT
>DR25_LDA ENSDARG00000023203 Chr.25:11,323,244-11,327,705 Dirscherl et al.2014 XM_001920255.4
MPVILILSLRLIKTGSHSLWMHSTYIKGQTPFPEFSFVLMLDDVRVMYYNGETKNVFPRG
NATNEDDVFDLNVLRTISDKVHSSFEEKWVVATQDVNKTEVVLALQRIVGCELRHDGKPG
QMITRDAARGSTTDELLYVDKNFTYQGTLNVPAFVLNMHLKISMWNHEHLYHPFCIKTLK
GYLEKRKNQVNRKVKPKVRLLLKKLSSSFRVSCLATGFYPRHINLTLLRDGQPVSDHDVT
GGDLLPNGDGTYQMRKSLQIRAEEREKHKYTCSAKHLSLDNKLDVNLGERFEPVHSKVIV
ACVVGLVLLTIAGTIIECRKRKQSGNKCN
>DR26_LLA ENSDARG00000096977 Chr.25:11,338,870-11,351,127 Dirscherl et al.2014 XM_003201408.2
MTGKVLFVLFLLSAPTTVLTDSHSLWLLGTYIKGETQFPKLSFTVMLDDLRVGFYNSETK
EFIPRDNTTNEDEAVSDVLTVIQNTLEPFMMSTLTFKNGTESPQLFQITWHCELLDNDKP
GQIIFKIAFSGSTTDEVSFYNDNFTCQCQNNMATVPLESFQMHYETVCYPNCIATIRDYL
KKRQTQYNRKDGQPVSDHEVTGGNLLPNGDGTYQMRKSLEIRADEREKHKYTCSVSHLSL
DKILDIDFEFDPSFLIKIVIPVVVLLSLMLVLTAVLIHKCKNKRAESKETITTASTPEPT
EMSETLRK
>DR27_LKA ENSDARG00000096940 Chr.25:11,354,781-11,367,096 Dirscherl et al.2014 XM_002666795.2
MARTSENMIGKVLFVLFLLSGPTSVLTDFHSLQILGTYIKGDTLFPQISFTFMLDDLTVG
LYITERDEFIPRDNTTNEDETVSDMLDKIKNELQPLLKGTLIFQNSTESPQLFQIAWHCE
LLDNNKTGQMILKIAFSGSTTDEVSFYGNNFTCQCQNNMATVPLEYFQMQFKSIYYPVCM
TTLGGYLVKRQVQVNRKVQPKVRLIKKVHPKSGGFRLSCLASEFYPYFINLTLLRDGQPV
SDHEVTGGDLLPNGDGTYQMRKSLEIRADEREKHKYTCSAKHLDKNLDVDFEFDPSYPVK
IVIPVVVLLSLVLVLTAVLIHKCMNKQAASPPEQQEPIEMGGRLRK
>DR28_LGA ENSDARG00000051713 Chr.25:11,371,003-11,372,257 Dirscherl et al.2014 XM_001920246.4
MCKIIFLLLFLLPAAAPKGSHSLWVVATYIKGQTPFPEFSYVLMLDDITVMYYNSDTKTF
FPRGNTTAEDDLYGSKDHLIIHDFMRSTFKDKWGLATKKLNKTDGVFALQQLVVLDLRAD
SEPGQIISQNTFEGCITDEVRYVDKKLTYQGTLNVSAPVHHIHHEYVKYLCETLIHPFYF
KTLKGYLIKRRNQINRKVKPKVRLILKANSDSGGFRVSCLATGFYPRHINLTLLRDGQPV
SDHGVTGGDLLPNGDGTYQMRKSLEIRGEEREKHKYTCSATHLSLDKKLDITLGLVAEFD
PGELFKSVIPAVLIVLSLVLVFITGVVI
>DR29_LIA ENSDARG00000097766 Chr.25:11,375,070-11,380,737 Dirscherl et al.2014 XM_001920233.2
MGQILLFFVFLLPTAAPKGSHSLWMLVMYIKGQTAFPEFSYVMMLDDVSVLHYNGDTNSL
IARGNTKADDDVFNLNALPIIHDHLQSSFEDRWTLATTHLNKTDGVFALQQLVACELQDD
GEPGRMILRNAFGGSTTDQLLFVDKKFTYHDSFNVSTHVLHAHHDYNKYLCEKLLQPFCF
QTLKGYLVKSRNQINRKVKPKVRLIQKANSDSGGFRVSCLATGFYPRHINLTLLIDGQPV
SDHEVTGGDLLPNGDGTYQMRKSLEIRAEEREKHKYSCSAKHLKIDNKLDIYLDFDPEEP
FPLVLILLSLVSVFITGVIIYKCRKRRAVIIYLLSKCIKKITS
>DR30_ZKA ENSDARG00000076734 Zv9_NA257:89,256-94,080 Dirscherl and Yoder 2013 KC607870
MMSGGVASGICALLCVFLLCGDLPSAQGEKHSLYYIYTGLSKPLDLPGIYEFSAMGLLDD
RQIDSYNSEEQRKIPKQQWMKEKMQEDYWEKGTQSRKSKEQWFNVNVHILMDRMRHNKSD
LHVLQWRHGCEVEIQGSEVKFSKGVSEYGYDGENLLSFDETESQWVAPVKEALPTKRKWD
NVPILNQYTKGYLEKECVDWLNKFREYADAELRNGSPPDVYKFTKKSTKDETKLKLTCLA

TGFYPSDVMLNIRRNRVYLPEQETISTGVRPNHDQTFQLSKSVEIKEDQIDEYDCSVSHR
TFKQPIIIKWDGTDLDGKYTGYPPETVPVVIGSVLILLAILAVVVFLVMNYDGECCKGLF
IHFTLLKDCGLLLPPCGKTEYV

2b. Zebrafish (*Danio rerio*) Expressed translated matches:

>EH570470 *Danio rerio* cDNA clone 3', cDNA sequence DR1 match
FQMFMFFSGDMVVKLTVRAMMSDFLKALMSTAMMEETSWPLMMRILSGLLQWKKLFQPKR
KWDNVPILNQYTKGYLEKECVDWLNKFREYGDQELREGSPPDVHVFAKKIISGKAKLKLT
CMVTGFYPKDVILTIRKYRTALSDNEVESSGVRPNPDGTFQLRKSTNIYEKAEYDCYVAH
RTLKEPIIKKWDGECQDCSSGTPIGTIFGALIGVLLVLAVIGGAVYFLANTRMGWRNAL
>CK018140 *Danio rerio* cDNA clone 5', cDNA sequence DR4 match
LAHKFREYGDKELREGSSPEVHVFAKRIINGKIKLKLTCLATGFYPKDVILNIRKYRITL
PDNEVESTGVRPNEDGTFQLRKSINIYEDEKAEYDCYVSHTTLKEPIIKKWDGECLDGPE
SGSPIGIIAGAIIVVLVVLAAIGGAVYFLRKRSGNNNVKPSSVPTISGNKDEKCSMLPGS
DDSGQGSSDGSSKSSPTNSQEKMDIV
>EB942259 *Danio rerio* cDNA clone, cDNA sequence DR7 match
DEELRNGSSPDVYKLTKKSTKDETKLKLTCLATGFYDKDVMLNIRRNCLPEDETESTGVR
PNHDQTFQLRKSVEIKEDQIDEYDCHLTHRTLKNPIIVTQVNFTPPVVE
>EH473102 *Danio rerio* cDNA clone 3', cDNA sequence DR11 match
IPAENQWFRTWDQVMIQQKRIVHENIYYPVCIKVLRRYLNVEKNSVMRKVKPRVRLMKKK
LPDSQGLQISCLATGFYPRHINLTLFRDAEPVDDDQIIGGEILPNGDGMYQMRKSLIVSK
EELDEGHEYTCTMKHLNLDNKLDIVFDVSGTVPGCFSVSVVISVLVFMCVSVFIITKLIM
RRKRQDTGNFFSKSKRKVKLQLFYTVKKFKPVQKKKKTHNPYFLSLFLVHSIFEEISCTG
LKLFVSNTK
>EH558607 *Danio rerio* cDNA clone 3', cDNA sequence DR12 match
TMKKLVHFITWMLLVDKIWKSLFLTPKKHTIQVKMPWVITWDQLKRLHENFMYDNVYHPI
CIKTLRRYLNMEKKNVMRKVKPRVRLMQKKLSDSQGLQISCLATGFYPRHINLTLFRDAE
LVDDDQITGGEILPNGDGTYQMRKSLIVSEEELQKGHKYNCTANYLNLDNKMDIVFDVAE
SDPGSFSVSVVMGVLVFVGLSVLSITALIMRRKRRDTGSVSGTSQNQYVYAQTSVQDAT
>CN322833 *Danio rerio* cDNA clone 5', cDNA sequence DR13 match
GNLNAFRQYGYNGEDFLTLDFMEMRWITPVQQAMITTQKWNNDRGFIESDMNYFRSECIE
WLQKYLEYGKSSLMKTVSPQVSVLQKYYFSPVVCHATGFYPSGIKISWQKNGQDHDEDVE
LGELLPNADGTFQKRSTLNVKPEEWKNSKFSCVVEHQGERILTEDEIRTNNPSATIGIII
GIVLSAVLLLFIAVAGFVVYWKKKGFKPVPANPSDSVAEKPRVNICDSDMTRCGMVRRVH
QSSTYSTHLLSTRLSTHLYEPKPV
>BQ450582 *Danio rerio* cDNA clone 5' similar to TR:Q31365 Q31365 UBA*01
CLASS I MHC cDNA sequence DR14 match
KDTLERKVSPQVSLLQKSSSSPVVCHVTGFYPSGLKISWQRNGQDHDEDVELGELIPNED
GTYQRTSTLNVKPEEWKKDKFSCVVEHQSKTINSILTEDEIRTNNPTAPLGIIIGIVVAA
VLLVAIAVAGFVVYRRHKGFKPVPQNTSDGGSDNSSRT
>EH477854 *Danio rerio* cDNA clone 3', cDNA sequence DR16 match
AQTLQHYYKYDCVYWLKQFLGLRKADLEIRDPEVFLLQKNPSSPVECFATGFYPSGVIIT
WLKNGQDHGENVELRELKPNEDGTFQRSSSLHVSQEDWKKSLYTCVVEHQRMTIQKSGDE
IKSNSSSEVF*ITPERVLAILLAALVICLCICYLVYPKKLKPYQPVNQNEEMYEYIVKNE*
>DR717949 Danio rerio cDNA clone 5', cDNA sequence DR20 match
DWTDAFERLTIFTVLYADNKCTIDINLNISQEQKEKIIEGVKNYYRNLIQPFCYKTLKVY
LKKRKDQVNRKVEPKVRIFHKANLDFGGFRVSCLATGFYPRHINLTLLRDGQPVSDHEFT
GGDLLPNGDGTYQMRKSLEIRAEDSEKHKYTCSFKHLDKEWHIDLAEPHRNTIWIAVSVL
LVCAIIVGLAMLIWKRYQTARQRENEPITTNYTYECT
FECDPGFLSLSTVIGGLVLCMSVITTALIVWRKRRAA
>EH478662 *Danio rerio* cDNA clone 3, cDNA sequence DR29_LIA match
IKLTLVACELQDDGEPGRMILRNAFGGSTTDQLLFVDKKFTYHDSFNVSTHVLHAHHDYN
KYLCEKLLQPFCFQTLKGYLVKSRNQINRKVKPKVRLIQKANSDSGGFRVSCLATGFYPR
HINLTLLIDGQPVSDHEVTGGDLLPNGDGTYQMRKSLEIRAEEREKHKYSCSAKHLKIDN
KLDIYLDFDPEEPFPLVLILLSLVSVFITGVIIYKCRKRRAVIIYLLSKCIKKITS

3a. Medaka (*Oryzias latipes*) Ensembl genes:

>OL1_UHA ENSORLG00000017153 Chr.8:24,973,752-24,985,505 and BAK26822
MKIQMFLCFLGLSFHGAAAVSHSLKILFIGPFPVQNLPGFIILVLVDDVLMTYYDSNITT
MVPRQDWLKEVVDEEFWKSYTKISLYAQQTFIGDIERAKQLYNQTEGVLVVQLMCGCEWD
EETGEVKGYSQLGYDGEDFLSFDLKTESWTAANQFAEIMKHQWDNNKPFNAGWKNDLDEI
FPKWLKEFVNLGRRFLMRTVLPSVSLLQKSSSSPVSCHATGFYPDRADLFWRRDGEEIHE
GVKKGQILPNNDGTFQMSVDLQLPPEAETHRYECVFQLSGVKEDVITELEKEQINTNEGL
KPPSWKIITGVVVAVVVDVVAVVAIIVYYRKKGQSPRSRTEVELTQMSA
>OL2_UIA ENSORLG00000000970 Chr.11:1,350,825-1,351,562
ADKERLKHFKIYHISSCPELLKEHMHYGRSFLQRTERPSVSFLQKTPSSPVCCHATGFYP
DRADLFWRKDGEEIHEGVEKGQILPNHDGTFQMSSELNVSLIKHEDWRRYDCVFQLSGLT
EDIVSMLDKTQIQTNWVSPSEFPVGPVVGGAAGVVLLLVISGVLY
>OL3_UIA ENSORLG00000000983 Chr.11:1,378,045-1,380,276
NLPEYVVTALMDKVELGNFDSIKREFESKQDWIKKLFNERPKIFQYYINKCRRNQQLFKA
NMDVFKDRLNQTEGSHILQRIGGCEWDDESDEVKGFEQYSYDGEICLDLQTETWIAPKTQ
AIVIKHLWDADKERLKHIQHMLISMCPKMLQDYMHYGRSFLQRTESPSVSFLQKTPSSPV
CCHATGFYPDRADLFWRKDGEEIHEGMEKGQILPNHDGTFQMSSELNVSLIKHEDWRRYD
CVFQLSGLTEDIVSTLDKTQIQTNWVSPSEFPVGPVVGGAAGVVLLLVISGVLYCCWRNR
NDVEMSPHGHHPHQMDPQPPGSLEVYKKQAIM
>OL4_Z ENSORLG00000001056 Chr.11:1,873,262-1,877,310
MLITSVLVLLGSGLLVNCEKHSLTYIYTAFSHPVKLPGIHEFTAMGLLDNRMIDYYDSSV
QKKIPKQDWMKERLQQEYWDKGTQSRQSKQQWFKVNIDILINRMRQTSNDTHVLQWMHGC
EGVEDEHGNLQFKRGMDMYNYDGDDFLAFDDERQVWVAAADAAVPTKRKWDEVTALKDYT
KGYLEKECMEWMKTFLSYSTQQLRNASRPDVYMFFKKAKESSNVVLTCLATGFYPKDITL
NIRRDGRVLTKDDGVMSSGVRPNHDETFQRKDYVEILRSDSATYTCEIIHPASNVWVVKT
WEGSPGSDIGLVLGLVCGIGGVLAIVVIRVLYKKK
>OL5_Z ENSORLG00000001044 Chr.11:1,867,734-1,871,932
ISLCFSEKHSLTYIYTAFSHPVKLPGIHEFTAMGLLDNRMIDYYDSSVQKKIPKQDWMKE
RLQQEYWDKGTQSRQSKQQWFKVNIDILINRMRQTSNDTHVLQWMHGCEGEEDEQGNLQF
KRGMDMYNYDGDDFLAFDDKHQVWVAAADAAFPTKRKWDDVTALKDYTKGYLEKECMEWM
KTFLSYSKQQLRNASRPDVYMFFKKAKESSNVVLTCLATGFYPKDITLNIRRDGRVLTKD
DGVMSSGVRPNHDETFQRTDYVEILRSDSANYTCEIIHPASDMHVVKTWDHHLPPVDKNN
LGLYVGIVIGLLVLILGSIIVFLV
>OL6_Z ENSORLG00000001058 Chr.11:1,943,702-1,945,742
AVEHSLTYIYTAFSKPVKLPGIHEFTAMGLLDNRMIDYYDSTEQKKIPKQDWMKERLQQE
YWDKGTQSRQSKQQWFKVNIDILINRMRQTSNDTHVLQWMHGCVGKEDDNGNIQFKRGMN
MYNYDGDDFLRFDDEHQVWVAAADAAFPTKRKWDDVTALKDYTKGYLEKECM
>OL7_Z ENSORLG00000001067 Chr.11:1,971,417-1,974,810
MYPVFIFIQQTQQIKWLFLLLFAVEHSLTYIYTAFSHPVKLPGIHEFTAMGLLDNRMIDY
YDSSVQKKIPKQKWMKERLQPEYWEKGTQSRQSKQQWFKVNIDILINRMRQTSNDTHVLQ
WMHGCVGKEDDNGNIQFERGMDMYNYDGDDFLRFDDEHQVWVAAADAAFPTKRKWDDVTA
LKDYTKGYLEKECMEWMKTFLKYSTEQEKVLSTTKPPEVFMFATPAKKKSNMVLTCFATG
FLPKEITMEIKRDLEGKSRVLSADDGLVSSGVRPNEDDTFQRRDHVEILKTDPASYSCRV
IHKATNMDVEQAWDHQHPDNDATGIIIGAAAGVVVLIAVGIVAVVIVMLKKKRKPSPSSS
TSSMSSPENTELLKKNSNGRLCPFNNLTVNFH
>OL8_UEA ENSORLG00000006435 Chr.11:15,112,547-15,116,458
MKCMLLKCDFCLNYYIQKSKKQFNLMSSKHSLEYFCTAASGVKEFPEFVGVATVDKDFVG
SCSMIIHRTEPKSTVIKKFIDENSKQIWWYYTTCKEKERFYASIIDGIKKHLNQNTVKYV
FSKHVFQQHSGCEWDDETGETKSFNRYAYDGEDFLKLDLLKHEWTALTEKAYATKLTWEN
NKVSLKHSEYFYSIQCPDWLKKYVTFGREYLQRAVLPLVSLLQRSNSSLVSCHATGFFPD
IADLFWMKDGEEFHGDVEKGEILPNNDETFQMSIYLNTSSLPLTDWQKFSCVFQLFSSQK
NITVSLDKTKIRTNDATHVMCSGNTDSFTKRHWGAGTALFILVAMAVSGSNMFKRLLNLQ
YCLCSSDKENKDVNGSICPQVDVSECN
>OL9_UDA ENSORLG00000006575 Chr.11:15,263,065-15,277,733
MKILGVLSVIFLGLYSAAAVVTHSLRYLHSASSGIPGFPGFVTVVLVDGQPFSYYDSNIR
RELPRQSWMVQTEDPDFWERRTQSSFVNEQVFKRKVAMIKPRFNLTGGVHVVQLLYGCEW
DEETGDIKGQWQFGHNGEDILALDLKTNTWMESTTVASFSNKNEDWVIQMNNYLSQGCPD
QLKKYVSHGRSTLMRTESPSVSLLQKNFLSPVRCHATGFYPNRAVMFWRKDGNEIHAGVE
KGEILPNNDGTFQMSVDLKLLSDTPEKEGYECVFQFSGVNESIITKLEESNIRTNGPSRY
EVMVSAVIAILIFAAVIADWLILYKRRKAADHLNKKKQILL

>OL10_UAA ENSORLG00000006772 Chr.11:15,340,240-15,374,324 and orlaUAA*0202 AB450991
MKIQLILCFLGLSFHGAAAVSHSLKYFYTASSQVPNFPEFVSVGLVDDAQISHYDSDTRM
TVPKQDWMAMDEQYWKRQTDIGLGHQQTFKANIEILKPRFNQTGGVHVFQWMYGCEWDEE
TGAVNSFHQYGYDGEDFIALDLKTESWIAPKQQAEITKHKWDNDKALMAGRKNYLTQICP
EWLKKYVNYGSSSLMRKVLPSVSLLQKSSSSAVSCHATGFYPDRAELLWRRDGEEIHEGV
EKGQILPNNDGTFQMSVHLQPPSGEDMQRYECVFQLSGVKEDVITKLEKAKIRTNEGSSS
QMLIIIGVLAAVMAVVAVSAFIFYKKKNAKRPPSPVDNKEIQEQMLQPENPSA
>OL11_UBA ENSORLG00000006798 Chr.11:15,417,434-15,446,882 and orlaUBA*0201 BAB83850.2
MKIQMFLCFLGLSFHSAAAVTHSLKYFYTASSQVPTLPEFVAVGLVDDAQIDYYDSDIRM
VVPKQDWMKEAMDGQYWKTETENRLGDQHTFKARIEILKQSFNQTGGAHVVQNMYGCEWD
DETGEVKGYDQYGYDGEDFIALDLKSESWIAAKQQAVITKDEWDDNKAFTVGRKNYLTQI
CPEWLEKYVNYGSSSLMRKVPPSVSLLQKSSSSAVSCHATGFYPDRAELLWRRDGEEIHE
GVEKGQILPNNDGTFQMSVDLQPPSGEDMQRYECVFQLSGVKEDVITKLEKAKIRTNEGS
SSQMLIIIGVLVAVIAVVLSVSAFIFYKKMNFFNLVKPPPSPVDNKEIQQQMLQPENPSA
>OL12_UCAΨ No Ensembl ID 5,412,645-5,420,668
MRIWGVLSVIFFGMNSAAAAASEGNRQTWTVQTEDPNFWERRTKRSIVDEQVFKSKVEMI
KPSFNQTGGVHIIQLMYGCEWDDETGSIKGWWQFGYNGEDLMALDLKTNTGTAQTPAATF
HKHKKDKDKGWAIRMNTYLSQGCPDHLKKYVSYGKSTLLRTETPSVFLLQKNFLSPVRCH
ATGFYPNKAVMFWRKDGKEITEGVETGEILPNNDGTFQMSTDLKLLLDTTEKKRYECVFQ
LSGANESIITKLEQSKIKTNGANPYEVTISVVFAVIIFAAVIADWLIVYKGRKAADHLNV
KK
>OL13_UCA ENSORLG00000012414 Chr.22:5,540,863-5,547,621 and AB033381
MRILGVLSVIFFGMNSATAVTHSLRYLHTAASGIPAFPEFVTVVLVDGQPFSYYDSSIKR
ELPRQTWTVQTEDPNFWERRTKSSIVDEQVFKSKVEMIKPSFNQTGGVHIIQLMYGCEWD
DETGSIKGWWQFGYNGEDLMALDLKTNTGTAQTPAATFHKHKKDKDKGWAIRMNTYLSQG
CPDHLKKYVSYGKSTLLRTETPSVFLLQKNFLSPVRCHATGFYPNRAVMFWRKDGKEITE
GVETGEILPNNDGTFQMSTDLKLLLDTTEKKRYECVFQLSGANESIITKLEQSKIKTNGA
NPYEVTISVVIAVIIFAAVIADWLIVYKGRKDSFYIDFFAGPDGKEEEGRREREV
>OL14 ENSORLG00000019579 Scaffold 721:105,268-108,455
MMKMFITLFFLCHAASAVKHSLILIFTGISGETNLPEVAISGSVDKVEICHFDSIKREVE
PKQDWTRKLFKEKPELLQLTLKRLLFHQQLFKDDMDIFKQRLNQTEGSHILQTITGCEWD
DESNEVKGFIQFGHDGEDFISLDLQTGTWIASKTQAVVIKIYGMQTKGDLGTFNTYSCQS
VLKTSGSHALLEFLCPERPSVSFLQKTPSSLVCCHATGFYPDRADLFWRKDGEEIHEGME
KGQILPNHDGTFQMSSELNVSLIKHEDWRRYDCVFQLSGLAEDIVSTLDKISPSEFPVGP
VVGGAAGVVLLLVISGVLYCCWRNRNDFQPVDERFTRST
>OL15_UIA1 ENSORLG00000020349 Scaffold 1100:46,629-48,948 and AB604118.1
IKSEDLIIIIVSLKKSDAFFSLVKHSLKWITTGISGKTNLPEFVVTASMDNVDLWHCDST
KRELEPKQEWTRKLFKFRPELLQFHLQRCLFNQQFFKAHMDVFKQRLNQTEGSHILQRIG
GCEWDDESDEVKGFEQYGYDGEDFISLDLKTGTWVAPKTQAAVIKHSWDADKERIKYIQQ
IFMSWFPKFLKDYMPYRRSFLQRTERPSVSFLQKTPSSPVCCHATGFYPDRADLFWRKDG
EEIHEGVEKGQILPNHDGTFQMSSELNVSLIKHEDWRRYDCVFQLSGLTEDIVSMLDKTQ
IQTNWDGLEKEETSSEGSKASQEEFSLAENS
>OL16_U ENSORLG00000020236 Scaffold 1612:6,397-11,204
SSGVHAFQYMYGCEWDDETGEVKGYRQYGYDGEDFIVYDLKMESWTAPKQQAEITKHKWE
NDKDFMAFLKNYLSQECPEWLKKYVNDGRSSLMRKVLPSVSLLQKSSSSPVSCHATGFFP
HRAELLWRRDGEKIHDGVEKGWILPNNDGTFQMSVDLQLPSEEDMQRYECVFQLSGVKQD
IITKLEKAKIRTNEGIIDSNSSSQMLIITAVIAAVLAVTAFILYKKKNPKRPPARKESTN
KTQKLPLSPDSNVNLSLAFSSI
>OL17_Z ENSORLG00000020395 Scaffold 1641:9,205-13,258
LCFSPVSFPEKHSLTYIYTAFSHPVKLPGIHEFTAMGLLDNRMIDYYDSSVQKKIPKQDW
MKERLQQEYWEKGTQSRQSKQQWFKVNIDILINRMRQTSNDTHVLQWMHGCEGVEDGHGN
LQFMSGVDMYNYDGDDFLAFDDEHQIWVATLDAAVPTKRKWDEVTALKDYTKGYLEKECM
EWMNTFLSYSKQQLKSASSPDVFLFASEAKEKANVVLTCLATGFYPKDITMNIRRDGRIL
TKDDGVVSSGVRPNQDDTHQRRDHVEILRTDVASYTCEIIHPGSNMHVEKPWDHRLPTEE
NFPIAPTAAIAVILVLIGIILF
>OL18_UIA2 ENSORLG00000018729 Scaffold 3269:4,387-6,197 and orlaUIA2*2301 BAK26842.1
KSHAILSSVKHSLKWIITATSGITNLPKFVVTASVDKVEFGHFDSIKREFEPKQEWTRKL
FKEKPELFQLGLQSCLYDQHFFKAYLDVFKERLNQTEGSHILQRIGGCEWDDESDEVKGF

QQYGYDGEDFISLDLQTETWIAPKTQAVVIKHLWDADKERIKHIQHMLMSECPKSLQDHM
LYGRSFLQRTESPSVSFLQKTPSSPVCCHATGFYPDRADLFWRKDGEEIHEGVEKGQILP
NHDGTFQMSSELNVSLIKHEDWRRYDCVFQLSGLTEDIVSMLDKTQIQTNWGKIFDPCFY
SVSPSEFPVGPVVGGAAGVVLLLVISGVLCCCWRNRNVSDFQPVNGKCFLSSHLISFVFG
TIDKQKVKCERR
>OL19_UGA BAK26816.1 MHC class I molecule, alpha-chain [Oryzias latipes]
MEVQMFLCFLGLSFHGAAAVSHSLKCFYTASSQVQNFPEFVAAALVDDVQINYYDSDTRM
MVPKQAWMKEAMDEQYWKTNTEIIMGAQQTFKANIDVAKERFNQTGGVHAFQYMYGCEWD
DETGEVKGYRQYGYDGEDFIVYDLKMESWTAPKQQAEITKHKWENDKDFMAFLKNYLSQE
CPEWLKKYVNDGRSSLMRKVLPSVSLLQKSSSSPVSCHATGFFPHRAELLWRRDGEKIHD
GVEKGWILPNNDGTFQMSVDLQLPSEEDMQRYECVFQLSGVKQDIITKLEKAKIRTNEDS
SSQMLIIIAVMAAGIPAVIAAVLAVTAFILYKKKNPKHPSAPVENKEVQQQMVPENPSA

3b. Medaka (*Oryzias latipes*) Expressed translated matches and unplaced genes:

>SRX377644 Transcriptome Analysis of Japanese medaka OL5 match, assembly of single reads e.g. SRR1029922.9339434.2
DTHVLQWMHGCEGVEDEQGNLQFKRGMDMYNYDGDDFLRFDDEHQVWVAAADAAFPTKRK
WDDVTALKDYTKGYLEKECMEWMKTFLSYSTQQLRNASRPDVYMFSKKAKESSNVVLTCL
ATGFYPKDITLNIRRDGRVLTKDDGVMSSGVRPNHDETFQRTDYVEILRSDSANYTCEVS
HPASDMHVVKTWDH
>DC269633 *Oryzias latipes* cDNA 5', cDNA sequence OL7 match
KKSNMVLTCFATGFLPKEITMEIKRDLEGKSRVLSADDGLVSSGVRPNEDDTFQRRDHVE
ILKTDPASYSCRVIHKATNMDVEQAWDHQHPDNDATGIIIGAAAGVVVLIAVGIVAXVIV
MLKKKRKPSPSSSTSXMSSPENTELLKKNSR
>DC264971 Oryzias latipes regenerating fin cDNA library OL7 match
FGTRGYQHPDNDATGIIIGAAAGVVVLIAVGIVAVVIVMLKKKRKPSPSSSTSSMSSPEN
TELLKKNSNESLSSGDFCYRQHQVSRQQRTPHEQHRRCQRENLLLLLQHLP
>BJ902860 *Oryzias latipes* cDNA 5', cDNA sequence OL10 match
KQQAEITKHKWDNDKALMAGRKNYLTQICPEWLKKYVNYGSSSLMRKVLPSVSLLQKSSS
SAVSCHATGFYPDRAELLWRRDGEEIHEGVEKGQILPNNDGTFQMSVHLQPPSGEDMQRY
ECVFQLSGVKEDVITKLEKAKIRTNEGSSSQMLIIIGVLAAVMAVVAVSAFIFYKKKNAK
RPPSPVDNKEIQEQMLQPENPSA
>orla_UIA3*2201 AB604120.1 *Oryzias latipes* Orla-UIA3 gene, unknown location in the Ensembl genome
YFLTATSGTPNFPEFVGVATLDEVQIGYCDNLEGEAQLKQKWMKKLINKTPKHLKFYSEK
CIRNHLLFKANMDDFKQRLNRTEGVHVLQRMNGCEWDDESREIKGFNQCGYDGEDLIALD
LKALTWIAPKAQFVVIKNLWNANKARLEMNKNYYMSDCPKLVQEYMRYGKSFLQRTEHPS
VSLLQRTPSSPVCCHATGFYPDRADLFWRKDGEEIHEGVEKGQILPNHDGTFQMSTELNV
SLIKHEDWRRYDCVFQLSGLTEDIITMLNETHIQTNRVSPSEFPVGPVVGGAAGIVLLLV
LSGLLYCCWRRNDFQPVHGEC

4a. Platyfish (*Xiphophorus maculatus*) Ensembl genes:

>XM1_U No Ensembl gene ID. JH556745.1:1,781,732- 1,783,290
VTHSLKYFYTASSGIENFPSYVSVGLVDEVQISYCDSRTNKNIPKQDWMNEVSSEYPNYW
KEETETCLAKQQTFKISLEIAKKRFNQTEGEHIYQQMYGCEWDDETGEVKGYDQFGYDGE
DLITLDPKTQEWIAPKPEAVITKLKWDNNRAAVEHENEYLTQTCVEWLKKYVNYGRSSLM
KTDRPSVSLLQKNSSSPVSCFATGFYPNRAEMFWRKDGEEIHDGVEKGEIKPNNDGTFQM
NVNIDLSSVATGDWIKYECVFQLSGVNDITNRLEKTRILTNE
>XM2_U No Ensembl gene ID. JH556745.1:1,835,219-1,836,654
VHHSLQYIYTASSGIQNFPEFVAVGLVDGNEVMYYDSNIREAKPKQDWMKKVTEDDPQYW
DFQTRELFGTLMSFINSIEVAKKRFNQTGGVHIFQNMYGCEFDDEYKEVEGWENFGYDGE
DFIALDLKTERWSAPVYQASITKNKWDNDKRKMAETKYYITQTCPDWLEKYLEYGKNSLK
RTSDFPWLLSLLQKLSSSQVSCHATGFYPSRAEMYWRRDGEEIHEDVNKGEILPNNDGTF
QMSVDLNISSISSNDWMRYECVFQLSGLKDLVIKLEPEKIQTND
>XM3_U No Ensembl gene ID. JH556745.1:1,848,144 -1,849,911
VTHSLKYFYTASSGIENFPSYVSVGLVDEVQISYCDSRTNENIPKQDWMDEVSSEHPNYW

KEETETCLAKQRTFKINLEIAKQRYSQSGGVPIFQQMYGCEWDDETGEVKGYDQFGYDGE
DFIALDLEGQSWIAPKPQAFNTKQNWENNRVAQELEKIYLTQTCVEWLKTYVSYGRSSLM
KTDLPSVSLLQKNSSSAVCCHATGFYPNRAEMFWRKDGEKIHDGVIKVEILPNNDGTFQM
SIYVNLSFVPSDDWAKYECVFQFSGFKDNIVTRLERKKIRTNE
>XM4_U No Ensembl gene ID. JH556745.1:1,854,049-1,854,360 pseudogene, orf error
AVHHSLQYIYTASSGIKNFPEFVAVGLVDGNEVMYYDSNIREAKPKQDWMKKVTEDDPQY
WDFQTHELSRTQKSFIDGIEVAKQRFNQTGGVHIFQNMYGCEFNDEYEEVEGWENFGYDG
EDFIALDLKTER*SPPVYQASITKNKWDNDKRKMALTKNYITQICPDWLKKYLEYGKNSL
LKT
>XM5_Z ENSXMAG00000001537 JH556782.1:1,256,093-1,263,684
EKHSLSYIYTAFSNPVDLPGFHEFTAMGLLDNRMIDYYDSDXXTDTHILQWMHGCEGETQ
SDGTMQFVRGMDMYNYDGNDFLSFDDDRQVWVAPIHAAEETKRKWDEVQVLKEYTKGYLE
KECMEWMSKFRDFGKEQLQRAAPPTVHLFTRKANVETNTILTCLATGFYPKDIILHIKRN
GRILTKEDRVQTDGVLPNHDDTFQRRDYVEILKSDVAKYTCEVKHPASSMHIETSLEPKG
EGNSLVIIAAIIPILIVLGIGV
>XM6_U a1 lineage IX, ENSXMAG00000014268 JH556877.1:584,294-586,879
MNYFGLLLLFHVASSVKHSLRYFLTATYGVEDFPEFVGAATVDEFQVGYCDSNIKTAKPK
QDWMRKLIERNPEHLEWYSQKCLGSQHVFRANIDDLKQRLNLTEGPYILQVLYGC
>XM7_U ENSXMAG00000014269 JH556877.1:622,448-624,584
MIKFLLTLLFCLLSSAEKHFLKFYYSGSSGLPHSSQSEVVAEIDGSLIAYCNQTEFKIHD
WVKKGLDDDVELLTFFKMMCSHTLPNTIQARISDLKQRFKEPEGVHVFQWIDSCGWDTET
GELTGFLQYGYNGEDFIALDFKRLSWIALKQQAIPTKLAWDTDRARLDYNKIFFSQLCPG
WLKRSLDYGRNFLMRT
>XM8_U ENSXMAG00000014271 JH556877.1:626,861-631,044
MKWFVILLMFSHSSSAESHFLKIYFTGSSGFTNVPAFEILGKLDDIEGAYCNNKIVDSKI
DFVKKLFSDDPKLFEFIKSSCFITNPAFFRHMIAELMKKFNQSEGIHTFQRMGGCELNME
TKEVTGFLKYGYDGEDFLEFDLKGLKWIALRKEALFLKQLWEMDRQDLIFNRDLLTNRCY
QALNYSLVAGHNYLFRTDYPSVSLLQKTPSSPVRCHATGFYPKSFLMFWRKDGEEIHEGV
DPGDILNNEDSTFQYRVDLDISSIPYMDWARYDCVFQFTGAEDKILIKLDKEVIKTNRDQ
SSEIRIIIIVVISAVITIIIIALAFTAYKRNDSENTFL
>XM9_U (ENSXMAG00000014273) JH556877:651,665-655,118
VKDSLTFFAIISTGIKNLPNLFTTVVDDIQVDYYDSNGIRKQSSILWNNISDSNQSTIFA
KETFNSLENLTISYFNKTANLNRTEAVHVLQCMSGCELDEKSAEVVTFQKCGYNGEDFMK
LDFKNLTWVAQHTLAENFTLKLNLDQQRLNNTKVFLSLTCPKVLNEFVIRSALQREDLTS
VVLLQKTPSSLVNCFATGFYPNAARMFWMRDGMEIQNDKEPSEILPNHDNTYQMSVYLNV
SSIASEDWKRYDCVF
>XM10_U (ENSXMAG00000014273) JH556877:658,701-668,042
MGCVIVIIIVALSSTAFKKRNEKHSLTVLVTASSGLPHFPDFVTTTQVDKLPTSYCDSNK
NIRANPKYGQKLINIESQIADWYIEQCFEIMSDYLKVKMGILTDLNQSEAVHILQVIIGC
KWEEKTKETTSFLQFGYNGADFIKFDPKKLTWIPQTPQAASIKPKWEADESTYHLKRNKD
FLNQICPDWLKKYMANNEGPLQSTVFLLQKSPDSPVSCSATGFYPNKAAMFWRKDGEEIQ
DGVDKTEILCNQDNTFQMRTDINVSSVNPEDWERYECVFHLVDVKDDVVSRLEKEKIQSN
WGKTQKHNEEEKPIGMIVGITAAGVFVIIVAAVGFTVI
>XM11_U (ENSXMAG00000014273) JH556877:668,437-671,686
MIKVIALLLLLQNAASGETYYLEMDHFLYPGVPNFPEYGVMDVFEGFLASYHDDNVKHIN
QDWLKEYVKKHPEDWKLSVQACLGYKDLFRTQTKNFNTWSNQSEGKEIIQQIIRCDWDEE
TDNTFGHVKYGNNGEDFITLDTKTETWFATNSKAEVIIDEWNADKTKYEIAKYILQNGCL
PLLKVFVKYGKSYLSRKVTCHATGFFPDKGIMFWRKDGEEIHEGVEHGDLLPNDDGSFQM
SVYLEALKIPPEDWGRYECVFQLSGVQNYVVTKLDQDSIRSNNPKNEGLLTTTSIAVIAT
>XM12_U ENSXMAG00000014655 JH556877.1:860,225-876,295
QYWERQTGNLQGTQQSFKANIETAKQRFNQTGGIHVFQFMYGCEWDEETKAKKGFWQFGY
DGEDFVAFDLSSKKWTAPTPQAVITKNKWDNDRGWITQADTYLNQECPDWINTYMNYGKT
SLMRTEKPSVSFLQKSSSSPVRCHATGFYPNKAELFWRKDGEEIHE
>XM13_U a1 lineage I, ENSXMAG00000014656 JH556877.1:898,266-902,441
MTNFVLLLILGIHTTAAVTHSLKYFYTASTQVPGFPEFVVVALVDDVQIAYYDSVIKRAE
PKQDWMREITDQQYWERETGNFLGNQQIFKANIEILKQRFNQTGGVHVVQLLSGVERDV
>XM14_U ENSXMAG00000014659 JH556877.1:911,478-927,700
FNQTGGIHLFQRMYGCEWDEETGEIKGFNQFGYDGEDFVAFDLKSGSWIAPTPEAVVTKH
KWDNNKGLNANQLNYYNQECPEWIKKYVNYGRSFLMRTVPPSVSLLQKSSSSPVSCLATG
FYPNKAEILWRKDGVEIHDGVEKREILSNNDGTFQTSAELKLSTSEDWTKYDCVFQLSGF

DKDLVTPLDKANIKTNEGNSLVIILTIVAVVVFITCTAVIIIVLFKRKNAKRPPSPVEYA
EVQEIMMPK
>XM15_U ENSXMAG00000005036 JH558542.1:21-4,011
MTPLIFLILVGIQGSTAVTHSLKYFYTASSGIENFPSYVSVGLVDDVQISYCDSKTNKNI
PKQDWMDEVRSEHPDYWKEETETCLVKQQTFKISLETAKQRFNQTGGEHIYQQMYGCEWD
NETGEVKVYDQFGYDGEDLIILDSNTQEWIAPRTQAVITANKWNNDRYDLEHEKIYLTHI
CVEWLKKYVNYGRSSLMRTVPPSVSFLQRFSSSPVSCFATGFYPNKTEMFWRKDGEEIHD
DVEKGEILPNNDGTFQMSIYLDLSSVPPEDW
>XM16_U ENSXMAG00000009441 JH558870.1:2.136-4.694
MTPLIFLILVGIQGSTAVTHSLKYFYTASSGIENFPSYVSVGLVDEVQISYCDSRTNENI
PKQDWMNELSSEHPDYWKEETETCLAKQQTFKANLEIAKQRFNQTEGVHVFQQMYGCEWD
EETGEVKGYDQFGYDGEDLITLDPKTQEWIAPKPQAVITKHKWDNNRAAVEHEKI
>XM17_Z ENSXMAG00000007599 JH559400.1:2.276-4.121
EKHTLTYIYTAFSKPVNLPGLHEFTAMGLLDNSMIDYYDSENQIKVPKQEWMKEHLEQEY
WEKGTQSRKSKQQWFKVNIDILMKRMRQNDTNTHILQWMHGCEGETQPDGTMQFVRGMDM
YNYDGNDFLSFDDDRQVWVAPIHAAEETKRKWDEVQVLKEYTKGYLEKECMEWMSKFRDF
GKEQLLKA
>XM18_U ENSXMAG00000016595 JH559413.1:21-2.267
MKCLVILMFCHAISAESHFLKIYFTGSSGFTNVPAFEIVGKLDDIEGAYCNNKIVDSKID
FVKKLFSDDPKLFEFIKSSCFLTNPAFFRHMIAELMKKFNQSEGIHTFQRIGGCELNMET
IEVTGFLKYGYDGEDFLEFDLKGLKWIALRKEALFLKQLWEMDRQDLIYNRDLLTNRCYQ
ALNYSLVAGHNYLFRT
>XM19_U (ENSXMAG00000016596) JH559413.1:2.228-9.853
MTMGITECAPIRRPDDPEPIMKPVKNSLTIFAIISTGIKNLPDLFTIVIDDIQVDYYDGN
GIRKQSSNLWKNISDSYQSPIFSKEVLNSLDVGFTEYLIRLGTLNPDEAVHVLQCMIGCE
LDEKSAEVVTFQQCGYNGEDFMKLYSKNMTWTAQHPLAEKLKPLWDSAQEGLNNITLFLT
LICPQMLEKFVTLSALQREDLTSVALLQKTPSSPVNCFATGFYPNAARMFWMRDGMEIQN
DKEPSEILPNHDNTYQMSVYLNVSSIASDDWKRYDCVFRLGNKTKLTRLEKAAISTNWAS
KFPDPSSYPTYPGTIQNCQHNFEIQ
>XM20_U ENSXMAG00000000946 AGAJ01050710.1:21-4.522
MMLIFLILVGIQGSTAVTHSLKYFYTASSGIENFPSYVSVGLVDEVQISYCDSRTNENIP
KQDWMNKVNSKHPNYWEEETETCWVKQRFLKGNLEIAKHRFNQTGGEHIYQQMYGCEWDD
ETGEVKGYDEFGYDGEDLIILDSNTQQWIAPRTQAVITANRWNNDRNDLEHENEYLTQTC
VEWLRKYVNYGRRSLMRTDLPSVSLLQKSPSSPVSCFATGFYPNRAEMFWRKDGEEIHDG
VEKGEIKPNNDGTFQMSVNIDLSSVATEDWNKYECVFQLSGVNDITNRLEKTRILTNESN
IPIVIITFVLFVTVLGLIAVIGFILYNQKNNGEKWIF
>XM21_U ENSXMAG00000014517 AGAJ01052725.1:21-1.100
VTHSLKYFYTASSGIENPSYVSVGLVDDVQISYCDNNINKNIPKQDWMNKVNSEHPNYWK
EETETCLAKQRTFKFSLKIAKQRFNQTGGVHVFQQMYGCEWDDETGEVKGYDQFGSDGED
LIMLDPQTQQWIAPKPQAVITANRFNNSRIIKELEKIYLT
>XM22_Z No Ensembl gene ID. AGAJ01058203.1:1-795
HILQWMHGCEGETQSDGSLKFVRGMDMYNYDGNDFLSFDDDEQVWVAPIHAAEETKRKWD
EVQVLKEYTKGYLEKECMEWMSKFRDFGKEQLLEA

4b. Platyfish (*Xiphophorus maculatus*) Expressed translated match:

>SRR073430.265648.2 XM5 Z lineage SRA match SRX031569
TKRKWDEVQVLKEYTKGYLEKECMEWMSKFRDFGKEQLQRATPPTVHLFTRKANVETNTI
LTCLATGFYPKDIILHIKKKVGCVS

5a. Nile tilapia (*Orechromis niloticus*) Ensembl genes:

>ON1_U ENSONIG00000007074 GL831254.1:1,934,802-1,939,135
MNSLCLFLLSCHVSSAVKHSLKYFITASSGISDFPEFVGAAVVDGVLVGYCDSSIRRAEP
KLEWMKELIKKDPQHLEWYTQKCSGNQQVFRANINSLKKRLNQTGGVHIFQRMNGCEWDD
ETDTITGFNQYGYDGEDFIALDLQTLTWIAPKPQAVVTKLQWDTEKPRLEHNKNYYINRC
PDWLKKYVKYGRSFLQRTVLPSVSLLQRTPSSLVSCHATGFYPERAMMFWRRDEEEIHEG
VEHGEILPNHDGSFQMSVELNVSSIKPDDWRRYDCVFQLSDVKEDIITKLDKTAIRTNWV
SPSVFPVGPVVGGVGGVLLLLAVFGFFIWRRNSNEFQLANTGEQ

```
>ON2_U ENSONIG00000007085 GL831254.1:1,942,502-1,944,822
VNVQSYFTQVRMMKYSLKYFLTATSGLPDFPEFVASALVNGVQVGYCDSNIRTAEPTQDW
MKKLVKDDPQHLDWYSEKCFGNQQVFRANIDSLKQRLNQTRGKKKKKKRMNGCEWDDETE
EIKGFNQYGYNGEDFIALDLQTLTWIAPKPQAVITKLKWDAEKARLEHNKNYYINRCPDW
LKKYVKYGRSFLQRSVLPSVSLLQRSPSSVVSCHATGFYPHRAKMLWRKDEEEFHDGVDK
GQILPNHDGSFQTSVQLNVSSIKPEDWRRHLTNTEGLSKPLSCPISLSEFPVGPVVGAAV
GVLLLLAVFGFFIWKRNS
>ON3_U ENSONIG00000007090 GL831254.1:1,948,887-1,979,577
MAVFLLLLFCSVSSAVKHSLKYFISGSSGVPNVPEFIGVMVVDGIQTGYCDSSNKTLKPQ
LDWAKKILQTNPEQLEWYTQKCFEDQPNVFRGHIFRWKQRFNQSGGVHVIQRISGCEWDE
NTDSVTGVLKYGYNGEGFLEFDLKTLTWIALKPEADMIKQKWDADRTRTMENEDFLTQTC
AEWLKMYVDNGKRSLHRTVPPSLSLLQKTPSSPVSCHATGFHPDRVMMFWRKDGDEIHDG
VEKGDILPNNDGTFQINVYLNVSSVKPEDWRKYECVFQLLSGENVIVTKLNETMIRTNWR
ENIFGTGREKPSDMTVPIIPAVVVLVLIIVAAAAGFAVYKKKKVSERRSTSSPDNNAEQI
QLNQVK
>ON4_U ENSONIG00000007093 GL831254.1:2,157,445-2,160,180
MGRLLLLLFLCQFGSSVKHSLKFFFCQTSGVQNIPEFVVVGLVDGVQKSYYDSNTGRPEP
KTEWMKKLMKDDPQHLEWYTARSFHTQDLFKHYIENLRKRFNQTEGVHILQSMNGCEWDD
ETGQINAFNQYGYDGEDFLTFDPQRLTWIALKPKAVITKLRWDGEEDQLKCNKNFYIHEC
PEILKKYIQCGKNFSQTAVLPSVSLLQKSSSSPVSCHATGFYPDRGLMFWRKDGEELHEG
VDPGEILPNNDGTFQLSVDLKLSSVTPEDWERYDCVFQLFGANEYIITKLDKTLIRTNWG
KPAHITATVVVLAIILTAAVAAVAAVAVIAYKRKKGE
>ON5_U ENSONIG00000007094 GL831254.1:2,171,187-2,174,216
MNSVLILLLCCHVASPVKHSLKYFFTESPGAQSIPEFVAVAIVDDVQIGDSNSVREATPK
KDWIKFFEDHPQHLQWYSLQSHDSHHFFKATIETLRQRLNQTEGVHILQRMKGCEWDDET
GEINGYDQYGYDREDFLVYDLKTLTWIAPKPHAVLTKMRFDAHEHQFESNKNFLVYQCPD
FLKEYLRYGKRFLQTAVLPKVSLLQKTPSSPVSCHATGFYPDRAVMFWRKDGVQLHEGVD
PGEILPNNDGTFQMSIDLNVSSVTPEDWQRYDCVFQLAGVNEHIITKLDKTAIRTNWAEK
PADMVTFISAAVVLLTVTIITAVAFVAYKKKKGEKSECAPDDGSEQSERLNPQS
>ON6_U ENSONIG00000007098 GL831254.1:2,183,675-2,186,455
LIQSLIIVTVKHSLKFFFTQTSGVQSIPEFVVVALVDEVQGGYYDSNTGRPEPKTEWMKK
LVKDDPQHLEWYTARSLDVQHLFKHYIENLRKRFNQTGGVHILQKLDGCEWDDETGEVNG
FNQYGYDGEDFLALDLQRLTWIALKPQAVITKLRWDGEKDRLEYNKNFYVHECPEFLKKY
VQYGKDFLQTAVLPSVSLLQKSSSSPVSCHATGFYPDRAVIFWRKDGEEIPEGVNPGEIL
PNNDGTFQLSVDLKLSSVTPEDWERYDCMFQLSGVNEHIVTKLDKAAIRANWEKPADITV
SVVVLAIVLIAAVAVGAAVVFVTYKRKKGER
>ON7_U ENSONIG00000007101 GL831254.1:2,265,696-2,268,042
IKIEQKQVLNINFLLIESQTVKHSLQYFFTQTTGVQSIPEFVAVALVDGVQGGYYDSSTK
RAEPKREWIKKLMQDDPQHLQWYADQSLHTNEVFKHYIGSLRQRFNQTGGVHILQSMNGC
EWDDETGEVNGFIQYGYDGEDFIALDLQKLTWITAKPQAVIIKLRWDAEKTRLEHNKELF
IHKCPELLKNYLQYGRSFLQTAVLPSVSLLQKSSSSPVSCHATGFYPDRAVMFWRKDGEE
THEGVHHTESLPNNDGTFQMSVDLKLSVTPEDWERYDCVFQLFGVNEYIVTKPDKAAIRT
NWEKPADIRATVVVLAIVLIAAVAAGAAGMFVAYKRKKGERS
>ON8_U ENSONIG00000007104 GL831254.1:2,282,142-2,294,806
MEKKMKCVLILLLLCCHVGSPVKHSLKYFITETPGAQSIPEFVAVAFVDEVPIGDFNSVRG
AKPKKDWIKFFEDHPEHLEWYSLQSHESHHFLKATIETLRQCLNQTDGVHILQKLDGCEW
DDETGEVNGFNQYGYDGEDFLAFDLQTMTWITAKPHAVTTKMIWDADKARLESNKNFIVH
QCLEFLKKYLQKSFLQATVLPSVSLLQKSSSFLISCHATGFFPNRGMMFWRKDGEELHEG
VDPGEILPNNDGTFQMSVDLNVSSVTPEDWQRYDCVFQLSGVNEDIINKLDKAVIRTNWE
IWGNLQSENNPESVTISIIPSVVVALILILVAVGVGVYKKKKERNTEPSLGDASEIQNLR
CSYLSL
>ON9 L_ ENSONIG00000001466 GL831385.1:447,962-465,059 LG11
MFVLQLMLMSLLLHHTMSSGTGRHSLWALASYIPGSAHFPEFTVVLMLDDIQVGYYDSKV
NQVMRTSTASDHKAELNLGQEPVNVLRDIYSSMRKRLNLVKHRFNLTIDGVHVQQRVTGC
EVLEDGQPALIMFRDGSNGQDADSLLYNMTHFTYAVREGWEIQWDALKKTSFQMLYSNIY
LPFCVRTLQHFLEREKHLVMRRVKPRLRFITRQVVGGAQVTCLATDFYPRHINLSLLRDG
QPVDEGEVRVGSVLPNGNGLYQVRKTLMVGEKELQRKHNYTCEAFHLSLDNRLRINWRAE
SSYSHRVHSISPLVVLMLAAVLLLVLVLRRRRRRK
>ON10_U ENSONIG00000019989 GL831408.1:1,437-4,505
MVDGIQVGYCDVSKKILEPRQEWAKNILEKHPEQLGWYKHKCFEHQPNFFRELISSLKQQ
FNQSEGVHILQRIDGCEWDETTGEVIGIIQYHYNGEDFLE
```

>ON11_U ENSONIG00000019990 GL831408.1:25,538-26,924
SGFLAMLVWRPVSSKHSLKYLLTETPGAQSIPEFVCVGFIDDVQFGSWNSRGGEKVEKDW
IKFFEDEPQLRQDYISTCSTTHYYFKETIRTLKQHLNQNEAGVHVLQRVSGCEWDNETGE
VNGYNQYGYDGEDFIAFDLQTATWITSNRQAETTKLKWNTERARLEYNKNFVIYLCPEFL
KRHLNYGRSFLEEEILPSVSLLQKTLSSPVSCHATGFYPHRAVIFWRKDGEELHESVDPG
QILPNDDGTFQ
>ON12_U ENSONIG00000019991 GL831408.1:45,731-50,156
MNSVLILLLCCHVASPVKHSLKYFFTESPGAQSIPEFVAVAIVDDVQIGDSNSVREATPK
KDWIKFFEDHPQHLEWYSLQSHDSHHFFKATIETLRQRLNQTEGVHILQRMKGCEWDDET
GEINGYDQYGYDREDFLVYDLKTLTWIAPKPHAVLTKMRFDAHEHQFESNKNFLVYQCPD
FLKEYLRYGKRFLQTAVLPSVSLLQKTPSSPVSCHATGLYPDRAEMFWRKDGEELHEGVD
PGEILLNNDGTFQMSVDLNVSSVMPEDWQRYDCVFQLSGVNEHIITKLEKTLIKTNWAEK
PTDTVTFISAAVIVLAVTIITAVAFVTYKMKKGEKSECAPDDGSEQSERLNPQS
>ON13_U ENSONIG00000019992 GL831408.1:54,395-57,192
QCHLIQSLIIVTVKHSLKFFFTQTSGVQSIPEFVVVGLVDEVQGGYYESNTGRPEPKTEW
MKKLMKDDPQHLEWYTTQSLDLKHLFKHYIEDLRKRFNQTRGIHILQKLDGCEWDDETGE
VNGFNQYGYDGDDFLTFDPQRLIWIALKPQAVITKLRWDGEKERLQYNKNFYVHECPEFL
KKYVQYGKDFLQTPGFPSVSLLQKTPSSPVSCHATGFYPDRAEMFWRKDGEELHEGVDPG
EILPNNDDTFQLSVDLNVSSVTPEDWQRYDCVFQLSGVNEYMMTKLDKAAIRTNRGKRIV
SQVQTSIVLIATAAVGAAVVFVTYKRKK
>ON14 ENSONIG00000019994 GL831408.1:90,628-92,246
TVKHSLKYFITESPGAQSIPEFVAVAFVDEVQIGDFNVRGAKPKKDWIKFFEDNPQHLEW
YTLRCLNRHHFLKATIESLRQRLNQTEGVHILQKLDGCEWDDETGEVNGFNQYGYDGEDF
LAFDLQTMTWITAKPHAVSTKMIWDADKFLSDNVILPIVCRKLFCKYFFKNSSYYLFAVL
PSVSLLQKSSPFLISCHATGFFPNRGMMFWRKDGEELHEGVDPGEILPNNDGTFQMSVDL
KLSSVTPKDWQRYDCVFQLSGVNEDIVTKLHKTVIRTNWGKTGSCRTEKPTDAVTFISAA
VVVLTVTIITAVAFVTYK
>ON15_U ENSONIG00000019996 GL831408.1:94,716-97,113
EWYRLKSVDRHHFLKATIETLRQRLNQTEGVHILQKLDGCEWDDETGEINGFNQYGYDGE
DFLALDLQTLTWIAAKPQAVITKIKWDAQKGRVEENKNFFVDQCPEFLKEYLRYGRRFLQ
TTVLPSVSLLQKTPSSPVSCHATGFYPDRAVMFWRKDGEELHEGVDPGEILPNNDDTFQL
SVDLNVSSVTPEDWQRYDCVFQLSAVKEHIVTKLDKTMIRTNWEKPADTVTFISAAVVVL
TVTIITAVAFVTYKKNYASDNDRSHDP
>ON16_U ENSONIG00000019998 GL831408.1:147,376-148,979
CPANFTVKHSLKFFSTQTSESIPEFSAGALVDEVQIGDFNSVRGAELKKDWIKFFEDHPW
HLEWYSFQSNDSHQFFKATIETLRQRFNQTEGVHIMQRMNGCEWNDETGEINGFNLYSYD
GEDFLALDLQTLTWITPKPQAVLTKLRWDAQKDRLKLNKTFLGHLCPEFLKEYLQYGRSF
LQTAVLPSVSLLQKTPSSAISCHATGFYPDRAVMFWRKDGEEIHEGVDPGEILPNNDGTF
QLSVDLKLSSVTPEDWQRYDCVFQFSGVNEYIITKLDKTAIRTNWAEKPTDMGTFIGAAV
IVLAFTIITAVTFLTYINKK
>ON17_U ENSONIG00000019999 GL831408.1:190,452-192,562
ISEFVGFPVIDGIQMAYYCDSSNKILEARQDWAKKILDTNPQMLESLTDYCFVDRPNLFR
LWISSLKQQLNQSRAVHILQMIEGCEWDENTGEVTGLLQYGYDGEDFLKLDLKTLTWIAL
KPEADIIKQSWDADTTRMKDREKILTKICPEWLKMYVDSGNSSLQRTVLPSVSLLQKTPS
SPVSCHATGFYPDRAVMFWRKDGEELHEGV
>ON18_U ENSONIG00000020001 GL831408.1:380,634-381,888
VKHSLKLIFIESSGVQDFVGVLWADETELVHCNTNLNKAEPKPNWL
KEFMKSNQEHNDLYTQECLHSQHFFKDTLNVLRRSCSQTEGVHTLQRMYGCEWDNETEEV
KSFDRYGYDGEDLMEFNQETQTWLISTPCASIVKHKLDNQQIRIADINHYLTNVCSKWLK
KYLDYGKYFLQRTDWPSVSLLQKTPSSSVSCHATGFYPDRAVMFWRKDGKKLYQFTDREQ
IYPNQDRTFQINVVLKLSSVTSEDWSRYDCVFQLSAVKEDIIIKLDQKVIKSNRGKSEPK
IMDLVCVFRTRSWESNNFKPKK
>ON19_U ENSONIG00000020051 GL831408.1:690,605-705,735
MKVFIFFLLLGIQGAAAVTHSLKYFYTGSSQVSNFPEFVALGMVDDVQIDYFDSNTQKKV
PKQDWMNDAVDPQYWERGTASRMSKQQVFKANIEIAKQRFNQTGGVHIFQLMYGCEWDDE
TKELGGYYQFGYDGEDFISLDLPTTHWTAAKQQAVITKQKWDTNGFAVSEKNYVSTICPD
WLKKYVSYGSSSLMRTERPSVSLLQKSSSSPVTCHATGFHPNRAEMVWKKDGMELHEGVD
KGEIITNNDGTFQMSVHLDVSSIKPEDWKKYDCVFQLSGMNEDIVTKLNKSDIKTNESKT
GIRKSYLIMIIAIVVVVVVLAAVIGIIVYKKKTSRCSQPPMKEPEVVKPLNPP
>ON20_U ENSONIG00000020058 GL831408.1:746,600-760,601
MFMVTMKAFIFFLLLGIQGAAAVTHSMKYFYTASSQVPNFPEFVAVGMVDDAQMVYYDSN

TEKAVPKQDWVNDAADPQYWERNTGNFRGAQQVFKANIEILKPRFNQTGGVHLYQWMYGC
EWDDETNEVKGFEQDGYDGKDFLSFDMKTETFITPVPQAIVTKHKLESNRGFIAQKVNYL
TQICPDWLKKYVSYGRSSLMRKVLPSVSLLQKTSSSQFHCHATGFYPNRAEMFWRKDGEE
HHEGVVKGEILPNNDGTFQMSVDLDLSSVTPEDWDKYDCVFQLSGVNEDIVTRLNKVTKK
EFPVGVVVGVIAGLLLLAVIAGVYMWQKKYKGFKPTNTSDTSST
>ON21_U ENSONIG00000020061 GL831408.1:820,845-825,121
NFPEFVAVGLVDDVQMIHYDSNTQKAEFKQQWMEKAAEDDPEYLSRQTNRLMNTQQVFKG
NIDILKQRFNQTGGVHINQMMYGCEWDDKTTVVNGYHYFGYDGEDFILFDLKTDTWIAPV
MQAVITKLKWDSDGSLSGDLRNYRTKLCPEWLKKYVNYGRSSLMRTELPSVSLLQKSSSS
PVHATGFYPDRAEMVWRKDGVEIHEGVNKGEILTNNDGTFQMSVDLDVSSVKPEDWHRYR
CVFQLSGVNEDIVTKLGKAAIRTNEGKIGKRIFPAGVVIRIMGILLLLTLCFSGIFIWRR
KYKGEK
>ON22_Z ENSONIG00000018185 GL831434.1:429,549-435,583
MFVISVLVLVGTGVTVNSEKHSLHYIYTGLSKPVGLPGIHEFTAMGLLDGRMIDYYDSEN
QTKVPKQEWMREHLPADYWVKGTQSRRIKQRWFKHNIGILMERMRKNDSGSPHVLQWMVG
CEGEMQPNGALRFVRGMSKYNYDGNDFLSFDDKNRVWVAPVKEALPSKTKWDNDQVLKEY
TKGYLENECIDWLSKSVTSEQKQLKKAPPPGVYVFAKKSRVETNLTLTCLATGFYSKNII
LRIRRKGRVLTEDDGLWSSGVLPNDDETFQRRDYVEILKSDLSEFSCEVVHEATRVDVVK
TWKNGDHPEEPGSGALIGAQGGALAVIALVVGGGLGLICLHKKGR
>ON23_Z ENSONIG00000005063 GL831484.1:36,038-44,524
MLGSKMFTLALFALLCPGPTQSRDTHSLHYIYTALSKPVGLPGIHEFTAMGLLDGRMIDY
FDSEHQAKVPKQQWMRERLPADYWDKGTQSRKSKQQWFKVNIGILMERMRQNDSGTPHIL
QWMHGCEGETQPDGTLRFVRGMDMYSYDGDDFLSFDGKNEVWVAPTPQALSTKRKWDNQT
VLKEYTKGYLENECIDWLSKFVNYGKEKLKSVPEVYVFAKCSTVDTNVVLTCLATGFYPA
ELTVTIRRNGRVLTADDGLMSSGLLPNHDETFQRRDNVEVLKSDVSVFSCEVRHEATNEH
AVKDWDDRLLPDSEGSSVHILVAAVVVPFVLVGVAAVLLFLYKNLNKRCWS
>ON24_Z ENSONIG00000005069 GL831484.1:61,315-63,813
SNTHSLHYIYTALSKPVSLPGIHEFTAMGLLDDRMIDYFDSENQAKVPKQQWMRERLPAD
YWDKGTQSRKSKQQWFKVNIGILMERMRQNDSDNLHVLQWMHGCEGETNSDGTLRFVRGM
DMYSYDGGDFLSFDDKNGVWVAPTPEAEPTKRKWDDVQVLKEYTKGYLENECIDWLSKFV
NYGQQQLKKKSPPELHVFAKNSRVQTNIVLTCLATGFYPKDVIMRIRRNGRVLTADDGLK
SSGPLPNNDDTFQRREYVEVLKSDTSPYSCEVFHKATNVSLSESWDLSMLPVPDDDGSAV
IGLVAALLLVLIGVGVILLVLRKRRINGRWNEQ
>ON25_U ENSONIG00000003532 GL831521.1:20,492-21,483
LTNSVSLTAKHSWKFYVMISSEFPNTPDYLAVITIDDVMMGSYNINMKTLEAQQDWVREF
IKDDPRQWETYTKNCMNYQQILIDETRIFKQHSNEIEGAHLIQQILGCEWDDETEKINGF
NLFGYNGEDFIAFDLETGTWITSNVEAEITKQKWNGNIAKNSLLKYFLKNMCPDGMKIPL
NYAKHFLNRKGKLTFFFHFPQKTPSSPVSCHATGFYPDRAVMFWRKDGEELHEYVDHKEI
QSNHDGTFQ
>ON26_U ENSONIG00000003533 GL831521.1:29,315-34,240
MMKKPMVLLLLCHAASAAIHSFKMYFMISSGVPNFPEYLAAVNVEDVMIAYYDSNMNEPE
PRQDWAKEVKKEDLQLCTEHGQMIILYQQMLEVATASFKQHTNQTKGVHIIQELTGYEWD
DETEKVTGIKQFGYNGEDFIALDIETETWISSNPQAEITKQIWNAHTNKGFWKNVFATTF
PAWSSMYMNYAESFVHRKVPPSVSLLQKTSSSPVSCHATGFYPDRAVMFWRKDGEEIHED
VDHKEILPNHDGTFQMSVDLDISSIGPEDWRRYDCVFQLSGFEDDVVTRLNESITRTNWD
KHLIRSIPIITAMVVLVVIVTVGVGFAVYKRKKGKQLKRLPCVNSPELEKILKHEVNCVH
>ON27_U ENSONIG00000003537 GL831521.1:71,123-73,681
PNLVILLLGTNELVQSTQMKHSLKYFVTGSSGVPNIPEFMGVVVFNGIQAGYCDSNNMTL
KPKQDWAKKILQTNRQQKEWYNHMCFKEEPNFFKNMISNVKQFTKISSGVHVLQRIGGCE
RDENTGEVTGLVHFGYNAEDFLEFNLETLTWIALKPEADIIKQEWDADRVRTKHNENFLT
QICPEWLKTYVDSAKSPLQKSVLPSVSLLQKTPSSPVSCHATGFYPDRAVMFWRKDGEEL
HEGVDPGEILPNSDETFQMSVDLNVSSITPEDWRRYDCVFQLSDGEDDIITSLNKTLIRT
NWVENITKGNIIGVKTVVVSVVFVVIGFGVFKKKRGEIFSVAYKSNSNSSVSNTVFWTL
>ON28_U ENSONIG00000003541 GL831521.1:150,998-186,211
MRIICFYLCFFFFYSTSSAVKHSLQYLVTASSGVPNIPEFMGAVVLDNIHMVYCDSINKI
LEPRQNWVKEMFENDTELLEIYTRKCTVILPQNLGNRISSLKEQFQSEGVHILQMREGCE
WDEKTGEVTGFLQYGYNGEDFVEFDLKTLTWIALKPEAAITKQRWDADSVRTKLNEDFLT
NIYPQWMKMFLSYGKRSLQRTVLPSVSLLQKTPSSPIICHATGFYPDRAVMFWKKDGEEI
HEGVDPGEILPNNDGSFQVNVDLNVSSVKPKDWRRYDCVFQFSDGEHVITKLDKTVIRTN
CGKKILCNDGGERNILFADIVNIMLYLFILYWIPGGITAMLGITLDRE
>ON29_U ENSONIG00000003542 GL831521.1:195,472-207,579

MRIICFYSCCFFLYSTSSAVKHSLQLLVTASSGVPNIPEFMGAMMLDGIQMGYCDSINKI
LEPRQDWVKKMFENDTELLEMYTRYCFVNLPNNFGKRISSLEEQFQSEGVHILQMREGCE
WDEKTGNVTGFLQYGYNGEGFVEFDLKTLTWIALKPEAAITKQKWDADSVRTKHNENLLT
NIYQKWMKKFLSYGKSSLQRTVLPSVSLLQKTPSSPIICHATGFYPDRAVMFWKKDGEEI
HEGVDPGEILPNNDGTFQSNVDLNVSSVTPEDWSRYDCVFQLSNDEHVITKLDKTVISTK
CGKKIPCNGGVDTPPATMTAVIAVVVAAVVALSVAAVGFVVYKK
>ON30_U ENSONIG00000003547 GL831521.1:263,545-270,469
MMFFLFPLFCHGLFAMKHSLMHSVTGSSGDPNISEFAGVVLVDGTEAVYCDSRNKILEPR
QDWMKKIFSNDTQHLALYTQQCFEDQPRIFRFLISTLKQLLHQIEGVHILQRTGGCEWNE
NTGEVTGVLQYWYNGEGFLEFDLKTLTWIPLKPEAAIIKQQWDTDTAVIKEVESLLTKFC
PEWLKRYLNYGSSFLQRTVLPSVSLLQKTSSSPVSCHATGFYPDRAAMFWRKDGEEIHEG
VDHREILPNNDGTFQTTVNLNVSSVRPEDWKKYDCVFQLSGVENNIVTKLHKTVIRTNWD
ENNRMEFLSIIIVPVLILVAAVGFFVYKKKRGER
>ON31_U ENSONIG00000003548 GL831521.1:355,060-357,370
SKHSLKYFFTETPGAQSIPEFVGVGFIDDVQFGSWNSRRGEDIKKDWIKFFEDEPQLLQE
YISQCSTFHHYFKDTIKTLNQHLNQTEGVHILQMTSGCEWDDETGESNGYSQYGYDGEDF
IVFDLQTGTWITANQQAETTKLKWNTEHGRLQRYKNFLSNSCPEFLKKHWHYGRSFLEKE
ILPSVSLLQKTSSSPVSCHATGFYPQRAMMFWRKDGEEIHESVVHGELFSNNDGTFQLSV
DLNVSSVRSEDWKRYDCVFQISGVKMGRDDSIVTKLDEAVIWTNWEKPTNIMTFISAALV
LLAFIIIAAVAFVIYKRKKGEKSYL
>ON32_U ENSONIG00000004918 GL831531.1:134,793-150,127
MLKELMILFFCHTASAVIHSLICNFMISSGLPNAPEYLVILNLDNVLMAYYDTNMKTVEP
RQHWLKEMKKEDLDTWENYMQNAPLYQYRLAEDTKSFFQQQTGGFHVIQQITGCEWDDET
GGIHSFTIFGYNGEDFIIFDKDTKTWIASTPEAEIAKENFNGKKARTEEFKTYHKNDCPI
LLKKSFNYAKSVLHRTVLPSVSLLQKTPSSPVSCHATGFYPNRAMMFWRKDREEIHERVV
HREMLPNQDGTFQIQIDLNISSVTPEDWRRYDCVFQLSGIKEDVVIGLETSLIRTNCGTL
TTIIIVVAVIVLLVLIIIGAIGFTVYKKKKVSEQPPTSPPETDCELSERLNSETI
>ON33_Z ENSONIG00000017388 GL831555.1:88,057-89,960
TETHSLHYIYTALSKPVGLPGIHEFTAMGLLDNRMIDYFDSKNQVKVPRQEWMRKRLPAD
YWEKGTQSRKSKQQWFKVNIGILMERMRQNESDTPHVLQWMHGCEGETHPDGTLKFVRGM
DMYNYDGNDFLSFDDKNGVWVAPIDEALPTKRKWDGVQVLKEYTKGYLENECIDWLSKFV
TYGQQQLKKKSPPEVFVFAKKSKVESNLILTCLATGFYPKDIIMRIRRNGRVLTADDGLT
SSGVLPNNDETFQRRDHVEI
>ON34_Z ENSONIG00000017389 GL831555.1:218,140-223,162
NQTHSLHYIYTALSKPVGLLGIHQFTAMGLLDGRMIDYFDSENQAKVPKQEWMRERLPAD
YWERGTRSRKSKQQWFKVNIGILMERMRQNDSDIHVLQWMHGCEGEANPDGTLRFVRGMD
MYAYDGDDFLSFDDKNGVWVAPSPEAEPTKRKWDGVLVLKEYTKGYLENECIDWLSKFVT
YGQQQLQNTSPPEVSLHAKTSTVDTDVLLTCLATGFYPADIVLRMKKNESVLTADDGLMS
SGVLPNEDHTFQRRDHVEV
>ON35_Z ENSONIG00000017390 GL831555.1:264,632-277,102
MNLFAVFVLLGTVLTVNCETHSLHYIYTALSKPVGLPGIHEFTAMGLLDDRMIDYFDSEH
QAKVPKQEWMEKRLPADYWEKGTQSRKSKQQWFKVNIGILMERMRQNDSDNHVLQWMHGC
EGETNPDGTLGFVRGMDMYNYDGNDFLSFDDKNGVWVATTPEAQPTKRKWDGVQVLKEYT
KGYLENECIDWLSKFVTYGQKQLKKKSPPDVHVFTKKAKVESNLILTCLATGFYPKDIIV
KIRRNGRVLTADDGLTSSGVLPNNDETFQRRDHVEILKSDLSEFSCEVIHEATGVDVAKT
WTNKCEEVSGSGSGALIGGAVVGVVVVVAVAVGLILYKMGIIGGRRGAKDNQGGIQTIYS
PVTVGNGVVSTPLTSGGSNGNAASSNGGSNGHATVPLLNGNAH
>ON36_U ENSONIG00000008406 GL831594.1:94,645-96,811
HQYGYDGKDFIIFDLNTETWIAPKEQAVITKHKWDHDADWNTQTKHYLTQDCIEWLQKYV
YYGRHSLKRTVLPSVFLLQKSSSSPVFCHATGFHPNRAEMVWRKDGVELHEGVNKGEILP
NNDGTFQMSVDLDLSSTKPEDWRRYDCVFQISGVSKDFIIKLDKAVIKTNQKGKMGIRGN
KDQKPMFS
>ON37_U ENSONIG00000008407 GL831594.1:105,890-108,506
MRTFMMQLFYWVASVAAVKHSLKYFYTASSQVPNFPEFVVVGMVDEVQMVHYDSVTEKAV
PKQDWMNKNTDQQYWERETGNFMGTHQVFKANIEIAKQRFNQTGGVHIVQWMYGCEWDDE
TNEVDGYEQFGYDGEDFITFDLQTETWVAPKQQAVVTKHKLNSNKAFLTSVKNYYTQICP
EWVKKYLNYGRSSLMRTVLPSVSLLQKTPSSAISCHATGFYPNRVKMIWRKDGEETHEGV
EIGEILTNNDGTFQMTVNLDLTSVPAEDWRRYDCVFQLSGVNEDIVTKLDNTVIRTNDGK
SIIRN
>ON38_U ENSONIG00000008409 GL831594.1:143,390-148,282
MFMVTMKVIIFFLILGIQGAAAVTHSLKYFYTASSQVPNFPEFVTVGMVDDVQINYYDSN

TEREVPKQDWVNDAVDPQFWERNTDISRGSQQTFKANIEIAKQRFNQTGGVHIFQQMYSC
EWDDETGEVNGYEQFGYDGEDFIGFDLKTETWVAPVQQAVITKYKWDSNRFYTRQAKNYL
TQICPEWLKKYVNYGRSSLMKTVLPSVSLLQKTPSSPVTCHATGFYPDRAEMMWKKDGEE
IHEGVEIGEILTNNDGTFQMSVNLDLRSVPAEDWRRYDCVFQFSGINENVTTTLDKTAIR
TNEGK
>ON39_U ENSONIG00000008413 GL831594.1:155,357-234,792
MKAFIFFLLLGIQGAAAVTHSLKYFLTGSSQVPNFPEFVVVGMVDDVQIDYYDSNTEKAV
PKQDWIARNTDQQYWERETANCWGSQQSFKANIDTAKQRFNQTGGVHIVQRMYGCEWDDD
TGEVNGYRQDGYDGEDFISFDLKTETWVAAKQQAVITKLKWDSNKATITQYKNYLTQICP
EWLKKYVNYGRSSLMKTVLPSVSLLQKSSSSSVTCHATGFYPNRAEMVWKKDGVEHHEGV
NKGEILTNNDGTFQMSVDLDVSSVKPEDWHRYRCVFQLSGVNEDIVTRLVKSDIKTNEGS
SFSLIIPVVVAVVVLAAIAVIAFIIYKKRTDKRPPSPAENREVQEQMLPQA
>ON40_U No Ensembl gene ID. GL831856.1:8,763-10,036
VAVGLVDDVQMIHYDSNTQKAEFKQQWMEKAAEDDPEYLSRQTNRLMNTQQVFKGNIDIL
KQRFNQTGETWIAPVTQAVITKLEWDSDRSLSGDLRNYHTQLCPEWLKKYVNYGRSSLMR
TVSLLQKTSSSAISCHTTGFYPKGAEMIWRKDGEELHEGVIKGDILSNHDGTFQMSADLD
FS
>ON41_U No Ensembl gene ID. GL831856.1:10,398-11,153
VAVGLVDDVQMIHYDSNTQKAEFKQQWMEKAAEDDPEYLSRQTNRLMNTQQVFKGNIDIL
KQRFNQTGETWIAPVTQAVITKLEWDSDRSLSGDLRNYHTQLCPEWLKKYVNYGRSSLMR
>ON42_U ENSONIG00000012499 GL831856.1:43,354-51,626
MFVVTMKAIIFLLFLRIRGAVAVKHSLKYFDTASSQVPNFPEFVVVGMVDDVQIVHYDSN
TEKTVPKQDWFAKNTDQQYWKRETEIFRGSQQTSIANIETVKKRFNQTGGVHIVQRMYGC
EWDNETGKVKGYDQHGYDGEDFALDLDTEIWIAPTPQAVTTKMKWDKNRALIAQKKKYLT
QECPEWLKKYVNYGRSSLMKTVLPSVSLLQKSPSSPVTCHATGFYPNRAELVWRKDGVEL
HEGVNKGEILTNNDGNFQMSVDLDLSSVRPEDWQRYDCVFQLSGVNEDIVTKLDKAVIKT
NRGKSDEVVSSAIAILIIIATLVLAVISFIVYKKRTGERKK
>ON43_U ENSONIG00000012501 GL831856.1:66,127-71,145
MKLFIFLFLLEIHGTMGPTHSLRYFYTSSSGVSNFPEFVAVGLVDDVQMIHYDSNTQKAE
FKQQWMEKAAEDDPEYSARQTNRLMNTQQVFKGNIDTLKQRFNQTGGVHINQLMYGCDWE
DETAEVNGYHLYGYDGEDFISFDLQTETWIAPKQQAVITKLKWDSDRSLSGDLKNYHTKL
CPEWLKKYVNYGRSSLMRTELPSVSLLQKSSSSPVSCHATGFYPDRAEMVWRKDGVELHE
GVNKGEILTNHDGTFQMSVDLEVSSIKSEEWHRYRCVLQLFRVNQDIVTKLGKAAIRTNE
GKIG
>ON44_U ENSONIG00000012502 GL831856.1:76,815-85,004
MVKMKALIFVFLLGIQGAAAVTHSLKYFYTGSSQVPNFPEFVVVGMVDDVQIDYYDSNTQ
KAEPRQDWIAKNTDQQYWERETEGFKGTQQSFKANIEILKPRFNQTGGVHIVQRMYGCEW
DDDTGEVNGYRQDGYDGEDFISFDLKTETWVAPVQQAVMTKTKWDSNKATITQYKNYLTQ
ICPEWLKKYVNYGRSSLMKTVRPSVSLLQKSSSSSVTCHATGFYPNRAEMVWKKDGVEHH
EGVNKGEILTNNDGTFQMSVDLDVSSVKPEDWHRYRCVFQLSGVNEDIVTKLDKSEIRTN
EGSSFSLIIPVVVVVVVLAAIAVIAFIIYKKRTGEK
>ON45_U ENSONIG00000015360 GL831938.1:36,313-41,071
ERHTITYIETAATGWNESPELTEVIFVNRQEFVHYKSSLKKMVPKTEWIEKTVDPEYWER
ERQRNIHSEQAIKAHVVWLMERLNQTSGVHILQWMYGCEWNEDTEEVTGFEILGYDGRDY
VAFDLKTETYIASAPQAVITKHNWDRDKTRISYKKYFLTQECVDKLKNFVNSGRKSLMKT
VPPSVSLLQKSPSSPISCHATGFFPNVAEMFWRKDGEELHEDVHKGEILPNNDETFQMSV
DLKLSSVKPEDWERYDCVFQFGGVNKEIVTKLDKTKIKTNTACKNTNICSIVTQFSLVLS
EVLSVVLSEVLW
>ON46_U ENSONIG00000015362 GL831938.1:49,095-52,875
HSLKYFYTAISQVPNFPEFVVVGMVDEVQMVQFDSNTMKTVSKQDWMNRNIDEQYWERET
GGFVSAQQVFKANTEIAKQRFNQTGGVHIVQQMYGCEWDDETNEVDGYEQFGYDGEDFIT
FDPQTETWVAPKQQAVVTKHKWDSNKAFITSVKNYYTQICPEYLNYGRSSLMRTVLPSVS
LLQKSSSSAISCHVTGFYPDRAEMIWRKDGEETHEGVEIGEILTNNDGTFQMSVNLDLRS
VPAEDWRRYDCVFQFSGINENVTTTLDKTVIRTNEGKNIIRNDEDVLFVLFCV
>ON47_U No Ensembl gene ID. GL831994.1:15,609-23,582 Pseudogene, orf error
GVHINQMMYGCEWDDETAEVNGYHQYGYDGEDFISFDLQTETWIAPVTQAVITKLEWDSD
RSVSGDLRNYHTKLCPE*LKKYVNYGRSSLMRTSCHATGFYPDRAEMMWRKDGEETHEGV
EIGEILTNNDGTFQMSVNLDLRSVPAEDWRRYDCVFQFSVMNEDIVTKLDKTVIRTNEG
>ON48_U No Ensembl gene ID. GL831994.1:26,035-26,301
VKHSLKYFYTASSQVPNFPEFVVVGMVDEVQILQFDSNTMKTVSKQDWMNRNIDEQYWER
ETGGFVSAQQVFKANIEIAKQRFNQTGGL

>ON49_U No Ensembl gene ID. GL831994.1:27,876-28,142
VKHSLKYFYTASSQVPNFPEFVVVGMVDEVQIVQFDSNTMKTVSKQDWMNRNIDEQYWER
ETGGFVSAQQVFKANIEIAKQRFNQTGGL
>ON50_U ENSONIG00000012484 GL831994.1:43,947-46,959
GVHIFQTMHGCEWDDETGEVNGYMQFGYDGEDFISFDLKDQMWVAPKEQAAITKNKLDHD
WALTAQYKNYFTQICPEWLKKYVNYGRSSLMRTVRPSVSLLQKSSSSSVTCHATGFYPNR
AEMVWKKDGVELHEGVNKGEILTNNDGTFQMSVDLDVSSVKPEDWHRYRCVFQLSGVNED
IVTRLDKSEIKTNEGSSFSLIIPVVVAVVVLAAIAVIAFIIYKKRTEKRPLSPAESQEPM
LPQAET
>ON51_U ENSONIG00000011081 GL832190.1:10,155-16,215
ETGGFVSAQQVFKANIEIAKQRFNQTGGVHIVQRMYGCEWDDETNEVDGYEQFGYDGEDF
IIFDLQTETWVAPKRQAVITKHKWDSNKAFITSVKNYYTQICPEWLKKYLNYGRSSLLRT
VLPSVSLLQKSSSSAISCHAAGFYPDRAEMMWRKDGEETHEGVEIGEILTNNDGTFQMSV
NLDLRSVPAEDWRRYDCVFQFSGINENVTTTLDKTAIRTNEGKKIIRNDEDVFPISVPIS
VPIVTAALVLAVVALIGFIIYKKKTVTAKPSKPYTKTSPENDPERSPSDSKPSSDITIKT
ELMTSTYRLSVTHSLNLCVVLHVFTH
>ON52_U ENSONIG00000018290 GL832444.1:49-5,673
LTVTHSLKYFYTGSSQVPNFPEFVTVGMVDDVQIEYYDSDTEKAVPKQDWFARNTDQQYW
ESQTGLFKGTQQTFKANIEILKQRFNQTGGVHINQVMYGCEWDEETGEVNGYRQEGYDGE
DFISFDLKEETWVAAKQQAVMTKIKLDHDWALTAQRKNYLTQICPEWLKKYVNYGRSSLM
KTIVPKVSLLQKTPSSPISCHATGFFPNVAEMFWRKDGEELHEGVHKGEILPNNDETFQM
SVDLKLSSVKPEDWERHECVFQFGGVNKEIVTKLDKTKIKTNTGKTDIVVSGPSGFHVGG
VIGGLAVLLLAVCIGIYFKLRGNGNEQNIFLCFMNDIHGLIVQTVNRRAVKSRQP

5b. Nile tilapia (*Orechromis niloticus*) Expressed translated matches:

>GAID01031757.1 TSA: *Oreochromis niloticus* Unigene41013_Sample_TC
transcribed RNA sequence ON9 L lineage match
LLLHHTMSSGTGRHSLWALASYIPGSAHFPEFTVVLMLDDIQVGYYDSKVNQVMRTSTAS
DHKAELNLGQEPVNVLRDIYSSMRKRLNLVKHRFNLTIDGVHVQQRVTGCEVLEDGQPAL
IMFRDGSNGQDADSLLYNMTHFTYAVREGWEIQWDALKKTSFQMLYSNIYLPFCVRTLQH
FLEREKHLVMRRVKPRLRFITRQVVGGAQVTCLATDFYPRHINLSLLRDGQPVDEGEVRV
GSVLPNGNGLYQVRKTLMVGEKELQRKHNYTCEAFHLSLDNRLRI
>GR664657 *Oreochromis niloticus* cDNA 5', cDNA sequence ON22 Z lineage match
MLVISVLVLVGTGVTVNSEKHSLHYIYTGLSKPVGLPGIHEFTAMGLLDGRMIDYYDSEN
QTKVPKQEWMREHLPAGYWEKGTQSRRIKQRWFKHNIGILMERMRKNDSGSPHVLQWMVG
CEGETQPNDALRFVRGMNKYNYDGNDFLSFDDKSRVWVAPVKEAFPSKTKWDNDQVLKEY
TKGCLENECIDWLSKSVTSEQKQLKKAPPPGVYV

6a. Stickleback (*Gasterosteus aculeatus*) Ensembl genes:

When relevant, exon 6 and 7 borders are shown with _.

>GA1_U ENSGACG00000001837 Group X:15,422-20,706 with exon 7
MRLVGAEISVLSLLMMSLHGAAALTHSLKNFYTGSSGVPNFPEFVVVGLLDEVEISHYDS
NTRREEPRQDWMSRVTEDDPQYWKSETEILMGQQQGFKVNIETAKQRFNQTGGVHIYQNM
YGCEWDDETNEVKGYYQFGYDGEDFISFDLQTERWIAPKHQAFITKQKWDHNRALIAGKK
NYLTHVCPEWVKKYLNYGRSSLMRTERPSVSLLQKTPSSPVSCHATGFYPDRADLFWRKD
GEELHEDVDLGEILPNHDGTFQMRVDLKLSSVPAEDWRRYDCVFQLSGVDEDIVTKLDKT
RTNREKPAASTFIIIIIIAVAVLVVIIAAVVGFKVYRKRN_KRPSSSSTVVSDGSEELNP
KP_GLWVNVALDQQAMLHILLHKLSIYGSSFI
>GA2_U ENSGACG00000001910 Group X:425,779-432,445 with exon 7
AEISVLSLLMMSLHGAAALTHSLKYFLTASSGLPNFPEFVGVGLLDEVEFVHYDSDTKRA
EPRQDWMSRLTEDDPQYWKRNTELAMDTQQVYKRHIEILNQSFNQTGGVHINQKMVGCEW
DDETNEVKGYDQFGYDGEDFLSFDPETERWITAKQQAVRIKQKWDQDRARTAHRKFVLTQ
ECPEWLKKYLSYGRSSLMRTERPSVSLLQKTPSSPVSCHATGFYPDIADLFWRKDGEELH
EDVDLGEILPNHDGTFQMRVDLKLSSVPAEDWRRYDCVFQLSGVDEDIVTKLDKTRTNRE
KPAGSTFIIIIIIAVAVLVVIIAAVVGFKVYRKRN_AKRSSAKCPSSTDGSEESLRDKL

NPKP_GLWVNIALDQQAMLHILLHKLSIYGSFILHLNV
>GA3_U No Ensembl gene ID. Group X: 460,641-468,080 internal errors, pseudogene
MRLVGAEISVLSLLMMSLHGAAALTHSLKYFYTASSGLPKFPEFVAVWLLDEVEFVHYDS
NTRRLEPRQDWMIRLTEDDPQFWKRNTELAMDIQQDFKGHIEIAKQSFNQTGGVHINQKM
AGCEWDDETNEVKGYVQYGYDGEDFISFDPETEQYIAPKPQAMRVDLKLSSVPAEDWRRY
DCVFQLSGVDEDIVTKLDKTRTNTEKPAASTFIIIIIAVAVLVVIIAAVVGFKVYRKRNA
QRSSSTDGSEESLRDKLNPKP
>GA4_U ENSGACG00000001913 Group X:481,025-492,757 with exon 7
MRLVGAEISVLSLLMMSLHGAAALTHSLKYFLTASSGVPNFPEFVIVGLLDEVEFSHYDS
DTKRAEPRQDWMSRVTEDDPQFWKRNTELAMDAQQVYKGHIEIAKQRFNQTGAGVHIYQR
MVGCEWDDETNEVKGYDQFGYDGEDFISFDLQTEQYIAAKQQAVRIKQKWDHNRALKAQN
KNYLTHVCPEWLKKHLNYGRSSLMRTERPSVSLLQKTPSSPVSCHATGFYPDRAALFWRK
DGEELHEDVDLGEILPNHDGTFQMRVDLKLSSVPAEDWRRYDCVFQLSGVDEDIVTKLDK
TRTNTEKPAGSTFIIIIIIAVAVLVVIIAAVVGFKVYRKRN_KCSSASTDGSEESLRDKL
NPKP_GLWVNIALDQQAMLHILLHKLSIYGSFIFHLNV
>GA5_U ENSGACG00000001919 Group X:520,053-527,028
AEISVLSLLMMSLHGAAALTHSLKFFYTASSGVPNFPEFVAVGLLDEVEVVHYDSDTRRL
EPRQNWVSRLRGDDPQYWKRNTKNSMDAQQVFKGNIETLKQRFNQTGAGVHIVQNMYGCE
WDDVTDEVKGYDQYGYNGEDFISFDLQTEQYIAAKQEASIIKQKWNQNRALIAGKKNFLT
HVCPEGLKKFLNYGRSSLMRTERPSVSLLQKTPSSPVSCHATGFYPDRADLFWRKDGEEL
HEDVDLGEILPNHDGTFQMRVDLKLSSVPAEDWRRYDCVFQLSGVDEDIVTKLDKTRTNT
GRSETREASTFIIIIAVAVLVVIIAAVVGFKVYRNRNAKCP
>GA6_U ENSGACG00000001932 Group X:662,965-665,020
GCEWDDETNEFKGYAQYSYNGEDFISFDLQTEQWIAAKQQAVRIKQKWDQDRADIAGRKF
VLTQTCREWLKKYLSYGRSSLMRTERPSVSLLQKTPSSPVSCHATGFYPDRAALFWRKDG
EELYEDVDLGEILPNHDGTFQMRVDLNLSSVPAEDWRRYDCVFQLSGVDEDIVTKLDKTR
TNTEKPADSTFIIIIIAVAVLVVIIAAVVGFKVYRKRNVSAQRSSSSSASTDGSELSEEL
NPKP
>GA7_U No Ensembl gene ID. Group X:678,166-682,178 alpha 2 domain only
GVHIYQNMYGCEWDEETNKVKGYYQYGYDGEDFISFDLQTEQWIAAKQQAVLTKQEWDQD
RVWTAQNKNYLTYECPESLKTLLKYGRSSLMRT
>GA8_U ENSGACG00000001935 Group X:779,138-791,924
VTHSLKYFYTGSSGVPNFPEFVIVGLVDEVEVVHYDSDTWRLEPRQDWVSRVTEDDHWFW
NWQTGLAMNAQREFKGYIEIAKQRFNQTGGVHIVQNMYGCEWDDETNEVKGYDQYGYNGE
DFISFDLQTEQWIAAKQEASIIKQKWDQNRALIAGKKNFLTHVCPEGLKKFLSYGRSSLM
RTERPSVSLLQKTPSSPVSCHATGFYPDRAALFWRKDGEELHEDVDLGEILPNHDGTFQM
RVDLKLSSVPAEDWRRYDCVFQLSGVDEDIVTKLDKTRTNRGRSETRSGEGGKQTSTLFT
LCVLDLFLFVVILRLG
>GA9_U ENSGACG00000001937 Group X:802,046-805,613 with exon 7
VTHSLKYFYTGSSGLPNFPEFVIVGLVDEVEVVHYDSDTWRLEPRQDWVSRVTEDDHWFW
NWQTGLAMNAQREFKGYIKTAKQRFNQTGGVHIVQNMYGCEWDDETNEVKGYDQYGYNGE
DFISFDLQTERYIAAKQQASIIKQKWNQNRALIAGKKNFLTHECPEGLKKFLSYGRSSLM
RTERPSVSLLQKTPSSPVSCHATGFYPHRAALFWRKDGEELHEDVDLGEILPNHDGTFQM
RVDLKLSSVPAEDWRRYDCVFQLSGVDEDIVTKLDKTRTNREKPADSTFIIIIIIIAVAV
LVVIIAAVVGFKVHRKRN_AQRSSAKPSSSSSASTDGSEVTEELNPKP_GLWVNIALDQQ
AMLHILLHKLSIYGSSFI
>GA10_U No Ensembl gene ID. Group X:849,550-861,373, Lacks a1 domain
MRLVGAEISVLSLLMMSLHGAAAGVHVMQRMVTCEWDDETNEVKGYDQYGYDGEDFLSYD
LQTEQWIAQKQQAVIIKEKLDRNRDLTAGNNGYLTDFCGRYLNIYLNYGRSSLMRTERPS
VSLLQKTPSSPVSCHATGFYPDRADLFWRKDGEQLHEDVDLGEILPNHDGTFQMRVDLKL
SSVPAEDWRRYDCVFQLSGVDEDIVTKLDKTRTNTGRSETRSGEGEKPAGSTFIIIIIII
AVAVLVVIIAAVVGFKVHRKRN_GLWVNIALDQQAMLHILLHKLSIYGSFIFHLNV
>GA11_U ENSGACG00000001941 Group X:891,779-894,040
VTHSLKFFLTASSGLPNFPEFVVVGLLDEVEVVHYDSDTRRAEPRQDWMSRVTEDDPQYW
KRNTEINMDTQQDFKANIEIAKQSFNQTGAGVHIVQRMIGCEWYDETNEVNGYDQYGYDG
EDFISFDLQTEQWIAAKQEASIIKQKWNQNRALIAGKKNFLTHECPEGLKKFLSYGRSSL
MRTERPSVFLLQKTPSSPVSCHATGFYPDRAALFWRKDGEELHEDVDLGEILPNHDGTFQ
MRVDLKLSSVPAEDWRRYDCVFQLSGVDEDIVTKLDKTRTNTGRSETRSGEGGKQTSTLS
TLCVLDLGSFVFL
>GA12_U No Ensembl gene ID. Group X:916,154-926,899 with exon 7

MRLVGAEISVLSLLMMSLHGAAALTHLLKFFLTGSSGVPNFPEFVVVGLLDEVEVVHYDS
DTWRVEPRQDWMSRVRKDLPWDWLALTQNALVAQQELKAYIEILKRRFNQTGGVHVMQRM
VTCEWDDETNEVKGYDQYGYDGEDFLSYDLQTEQWIAQKQQAVIIKEKLDRNRDLTAGNN
DCLTPFCGRYLNIYLNYGRSSLMRTERPSVSLLQKTPSSPVSCHATGFYPDRADLFWRKD
GEQLHEDVDLGEILPNHNGTFQMRVDLKLSSVPAEDWRRYDCVFQLSGVDEDIVTKLDKI
RTNTEKPAASTSTSIIIIIIIIIAVAVLVAIIAAVVGFKVYRKRN_PNALLLLLLLLTAL
SSEELNPKP_GLWVNIALDQQAMLHILLHKLSIYGSFIFHLNV
>GA13_U ENSGACG00000001973 Group X:1,058,136-1,070,262
MRLVGAEISVLALLMISLHGAAALTHSLKFVLTASSGVPNFPEFVVVGLLDEVEVVHYDG
DTRRAEPRQDWMSRVREDDPQFWKSGTELAMDAQQVFKGYIETAKQSFHQTGGVHIYQRM
AGCEWDDETNEDKGYIQDGYDGEDFISFDLQTEQYIAAKQQAVITKRKWDHNRAKIAHWK
NFLTHVCPEGLKKFLSYGRSSLMRTERPSVSLLQKTPSSPVSCHATGFYPHRAALFWRKD
GEQLHEDVDLGEILPNHDGTFQMRVDLNLSSVPAEDWRRYDCVFQLSGMDEDIVTKLDKT
RTNTEKPAGSTFIIIIIAVAVLVVIIAAVVGFKVYRKRNGESSSSSSSSTDGSDVTEEL
>GA14_U ENSGACG00000001976 Group X:1,090,323-1,093,673 with exon 7
THSLKYFYTGSSGVPNFPEFVVVGLLDEVEIVHYDGNTRRTEPRQDWMSRLREDDPQFW
DIQTDIAMDAQQDFKGHTETAKQSFNQTGGVHIFQRMAGCEWDDETNEVKGYDQFGYDGE
DFISYDLQTEQWIAAKPQAVLTKQKWDQDRADIAWRKNFLTHFCPEWLKMFLSYGRSSLM
RTERPSVSLLQKTPSSPVSCHATGFYPDRANLFWRKDGEQLHEDVDLGEILPNHDGTFQM
RVDLNLSSVPAEDWRRYDCVFHLSGVDEDIVTKLDKTRTNTEKPAGSTSTSTFIIIIIII
IIAAVVVLVVIAVRRFKVHRN_AKRSSASTEVSEELNPKP_GLWVNIALDQQAMLHILLH
KLSIYGSSFI
>GA15_U No Ensembl gene ID. Group X:1,096,590-1,102,571
MRLVGAEISVLSLLMISLHGAAALTHSLKYFYTGSSGVPNFPEFVVVGLLDEVEIVHYDG
NTRRTEPRQDWMIKVIDDDPQYWKSQTDIAMDTQQAFKGYIEIAKQSFNQTGGVHIFQRM
AGCEWDDVTNEVKGYDQYGYNGEDFISFDLQTDRWIAQKQQAFITKQKWEQNSALKAHKK
NFLTHVCPESLKKYLNYGRSSLMRTERPSVSLLQKTPSSPVSCHATGFYPDRAALFWRKD
GEQLHEDVDLGEILPNHDGTFQMRVDLNLSSVPAEDWRRYDCVFQLSGVDEDIVTKLDKT
RTNR
>GA16_U ENSGACG00000001978 Group X:1,119,796-1,124,476
VTHSMKYFLTASSGVPNFPEFVVVGLLDEVEVVHYDGNTTRAEPRQDWMSRLKEDDPQYW
KSRTELAMYTQQDFKGNIETAKQRFNQTGAGVHIYQRMAGCEWDNETDEVQRYIQDGYDG
EDFISFDLQTKRWIAAKQQAVIIQQKWNQDRDKIEYWKNFLTDECAEGLKTFLNYSRTSL
LRTERPSVSLLQKTPSSPVSCHATGFYPDRAALFWRKDGEELHEDVDLGEILPNHDGTFQ
MRVDLNLSSVPAEDWRRYDCVFQLSGVDEDIVTKLDKTRTNTDKFIHPFVNSFQVRALSV
AVLVVIIAAVVGFKLYRKRK
>GA17_U ENSGACG00000001979 Group X: 1,144,164-1,151,431 with exon 7
MRLVGAEISVLSLLMMSLHGAAALTHSLKYFLTASSGLPNFPEFVIVGLLDEVELFHYDS
NTRRAEVRQDWMIRVREDDPRYLKRGTEVLMDAQQVFKVNIEIAKQRFNQTGGVHIFQRM
VGCEWDNETNEVKGYDQFGYDGEDFISYDLQTEQCIAAKQQAVITKQKWDQDRALKAHKK
NSLTHVCPESLKTLLNYGRSSLMRTERPSVSLLQKTPSSPVSCHATGFYPDRAALFWRKD
GEELHEDVDLGEILPNHDGTFQMRVDLKLSSVPAEDWRRYDCVFQLSGVDEDIVTKLNKT
RTNTEKPAGSTFIIIIIIIAVAVLVVIIVAVVGFKVYRTVRE_AQRSSSSSSSTDGSEV
SEEVNPNP_GLWVNIALDQQAMLHILLHKLSIYGSFIFYLNV
>GA18_Z ENSGACG00000009731 Group X:14,983,618-14,987,412
MSPLAVLVLLQVTAAVFGVKHSLTYTYTAFAHKPVGLPGIHEFTAMGHLDTRMIDYFDSD
QQLKVPKQPWMGERLDKDYWVKGTQSRQSKQQWFKVNIGILMNRLRQNKTSGQHVLQWQH
GCEGEMQLDGTLKFSTGVDMYSYDGYDFLSFDDSNSAWVAGAPAAQQTKRTWDGVDVLKE
YTKGYLEKECMEWMEKFLKYQGSVLENATKPEVYLFASKAKKEANVILTCMATGFYPKEI
QLWIKRNGRVLRREDGVMSSGSRPNGDETFQRKDWVEILKTDQSQYTCEVIHKATQVNIE
KEWDHKLPENGSPIGVAVGVPVLVLVLAVVAGVLIFCYRKASSTSSTSSTSSTSSTSSTS
SSNTDSIS
>GA19_U ENSGACG00000000116 Scaffold_58:785,580-791,571
MRLVGAEISVLALLMMSLHGAAALTHSLKFFYTGSSGLPNFPEFVAVGLLDEVEIDHYDS
DTRRAEPRQDWMSRVIEDDPQYWKSQTEIAMAIQQVYKGNIETLKQRFNQTGGVHIYQNM
YGCEWDNETNKVKGYIQFGYDGEDFISFDLQTEQWIAAKQKAVITKQKWDQNRADKAHWK
NYLTHVCPEWLKKYLNYGRSSLMRTERPSVSLLQKTPSSPVSCHATGFYPDRASLFWRKD
EEELHEDVDLGEILPNHDGTFQMRVDLKLSSVPAEDWRRYDCVFQLSGVDEDIVTKLDKT
RTNTEKPAGSTSTFIIIIAVAVLVVIMAAVVGFKVYRKRNAQRSSAKWPFDKSEEERLSW
TN
>GA20_U (ENSGACG00000000122) Scaffold_58:823,228-835,972 with exon 7

```
MRLVGAEISVLSLLMMSLHGAAALTHSLKNFDTASSGVPNFPEFVNVGLLDEVEMFHYDS
NTTRAEPKQDWMSRVIEDDPQYWKRQTEKSMNAQQVFKVDIGTAKRRFNQTGGVHIVQLM
IGCEWDDVTNEVKGYNQYGYDGEDFISFDLQTEQWIAPKQQAVLTKQKWDHNRALKAHDK
NYLTHVCPEWLKKYLNYGRSSLMRTERPSVSLLQKTPSSPVSCHATGFYPHRAALFWRKD
GEQLHEDVDLGEILPNHDGTFQMRVDLKLSSVPAEDWRRYDCVFQLSGVDEDIVTKLDKT
RTNTEKPAGSTFIIIIIIAVAVLVVIIAAVVGFKVYRKRN_AKYSSAKCPSDKSEEESLS
GTN_GLWVNVALDQQAMLHILLHKLSIYGSSFI
>GA21_U (ENSGACG00000000122) Scaffold_58:865,091-877,650
MRLVGAEISVLSLLMMSLHGAAALTHSMKYFYTGSSGVPNFPEFVIVGLLDEVEMFHYDS
NTRRAEPRQDWMSRVTEDDPQYWKRQTEKSMDTQQVYKVDIEILKQRFNQTGGVHIVQLM
IGCEWDDVTNEVKGYNQYGYDGEDFISFDLQTEQWIAPKQQAVLTKQKWDHDRALKAHDK
NYLTHVCPEWLKKFLNYGRSSLMRTERPSVSLLQKTPSSPVSCHATGFYPHRAALFWRKD
GEQLHEDVDLGEILPNHDGTFQMRVDLKLSSVPAEDWRRYDCVFQLSGVDEDIVTKLDKT
RTNAEKPAGSTFIIIIIIIIAVVVLVVIIAAVVGFMVHRKRNGER
>GA22_U (ENSGACG00000000122) Scaffold_58:892,818-902,815, with exon 7
THSMKYFYTGSSGVPNFPEFVSVGLVDEVEFVHYDSNTRRAEPRQDWMSRVTEDDPQFWK
RETENFMGHQQVFKANIETAKQRFNQTGGVHINQWMVGCEWDNETNEVKGYYQFGYDGED
FISFDLQTERWIAPKQQAVITKRKLDQNRAKIAEDKNYLTHVCPEGLKKYLNYGRSSLMR
TERPSVSLLQKTPSSPVSCHATGFYPDRADLFWRKDGEQLHEDVDLGEILPNHDGTFQMR
VDLKLSSVPAEDWRRYDCVFQLSGVDEDIVTKLDKTRTNREKPAGSTFIIIIIIAVAVPA
AIIAAVVGFKVHRNRN_KRPSSASSSSSASTDGSELSEELNPKP_GSWVNIALDQQAMLH
ILLHKLSIYGSSFI
>GA23_U (ENSGACG00000000122) Scaffold_58:949,528-954,420
MRLVGAEISVLSLLMMSLHGAAALTHSMKYFLTASSGVPNFPEFVIVGLLDEVEMFHYDS
NTRRAEPRQDWMSRVTEDDPQYWKSETETFMGTQQVYKVDIETVKRRFNQTGGVHIVQFM
YGCEWDDETNEVKGYAQDGYDGEDFISFDLQTEQYIAPKQQAVITKQKWDQNRAWIAGKK
NYLTHVCPEWVKKHLSYGRSSLMRTERPSVSLLQKTPSSPVSCHATGFYPDRADLFWRKD
GEELHEDVDLGEILPNHDGTFQMRVDLKLSSVPAEDWRRYDCVFQLSGVDEDIVTKLDKT
RTNREKPAASTFIIIIAVAVLVVIIAAVVGFKVHRNRNGESSSSSSASTDGSELSEEL
>GA24_U ENSGACG00000000141 Scaffold_58:996,326-1,001,749 with exon 7
MRLVGAEISVLSLLMMSLHGAAALTHSMKFFYTASSGLPNFPEFVIVGLLDEVEMFHYDS
NTRRTEPRQDWMSRVTEDDPQYWKSQTEIAMGQQQVFKGHIEIAKQRFNQTGAGVHIYQN
MYGCEWDDETNEVKGYNQDGYDGEDFISFDLQTERWIAPKHQAFITKQKWDHNRALIAGR
KNYLTHVCPEWVKKYLNYGRSSLMRTERPSVSLLQKTPSSPVSCHATGFYPDRAALFWRK
DGEELHEDVDLGEILPNHDGTFQMRVDLELSSVPAEDWRRYDCVFQLSGVDEDIVTKLDK
TRTNREKPAASTFIIIIAVAVLVAIIAAVVGFKLYRNRN_AKPSSSSSASTEGSEVTEEL
NPKP_GFWVNVALDQQAMPHILLHKLSIYGSSFI
>GA25_U No Ensembl gene ID. Scaffold_58:1,034,716-1,038,858
MRLVGAEISVLALLMMSLHGAAALTHSMKYFYTASSGVPNFPEFVAVGLLDEVEFSHYDS
NTRRTEPRQDWMIRLTEDDPQYWKRSTEIFMGNQQVFKGTLKQQNNASTKLEVFTG
>GA26_U ENSGACG00000000151 Scaffold_58:1,078,466-1,084,416
VTHSMKYFYTASSGVPNFPEFVAVGLLDEVEFSHYDSNTRRLEPRQDWMSRVTEDDPQYW
KRYTENFMGAQQVYKGNIETLKQRFNQTGAGVHINQRMVGCEWDDETNEVKGYDQDGYDG
EDFISFDLQTEQWIAPKQQAVITKRKWDHNRAQIAQNKNYLTHECPEGLKKYLNYGRSSL
MRTERPSMSLLQKTPSSPVSCHATGFYPHRAALFWRKDGEELHEDVDLGEILPNHDGTFQ
MRVDLNLSSVPAEDWRRYDCVFQLSGVDEDIVTKLDKTRTNREKPAGSTFIIIIIAVAVL
VVIIAAVVGFKVHRKRNAKPSSSSSATTDGSDVTEELNPKP
>GA27_U ENSGACG00000001288 Scaffold_452:3,492-8,260 (x=NNN region)
MRLVGAEISVLSLLMMSLHGAAALTHSLKYFYTASSGVPNFPEFVIVGLLDEVEMFHYDS
NTRRAEPRQDWMSRVTEDDPQYWKRYTEILMGSQQVYKGNIETAKQRFNQTGxxxxxxxx
xxxxxxxxERPSVSLLQKTPSSPVSCHATGFYPDRAALFWRKDGEELHEDVDLGEILPNH
DGTFQMRVDLKLSSVPAEDWRRYDCVFQLSGVDEDIVTKLDKTRTNTEKPAGPPPPSTFI
IIFIAVAVLVAIIALFISAKRSAAKSPFSSASTDGSDVTEELNPK
>GA28_U No Ensembl gene ID. Scaffold_653:7,716-9,769
MRLVGAEISVLSLLMMSLHGAAALTHSLKYFFTASSGVPNFPEFVAVWLLDEVEFVHYDG
DTRRLEPRQDWMIRVIDDDPQFWKRNTELYMGCQQDFKGYIEIAKQSFNQTGGLFM
>GA29_U ENSGACG00000000124 Scaffold_854:596-5,501 pseudogene, orf error
MRLVGAEISVLSLLMMSLHGAAALTHTMKYFLTASSGLPNFPDSVALWLLDEVEIEH*NS
NTRRAEPRQDWMSRVTEDDPQYWKSESETFMGTQQVYKVDIETVKRRFNQTGGVHIYQRM
VSCEWDDETNEVKGYDQDGYDGEDFISFDPETEQYIAPKQQAVRIKQKWDQDRARIAHNK
NFLTHVFPEWVKKYLSYGRSSLMRTERPSVSLLQKTPSSPVSCHATGFYPDRAALFWRKD
```

GEELHEDVDLGEILPNHNGTFQMRVDLKLSSVPAEDWRRYDCVFQLSGVDEDIVTKLDKT
RTNTEKPAASTFIIIAVAVLVAIIAAVVGFKVYRKRNGERDASSSASAVGSEVTEEL
>GA30_U ENSGACG00000002179 Scaffold_1223:3,225-5,871
WKWYTEILMGNQQVFKANIETLKQRFNQTGAGVHIFQRMYGCEWDDETNEVKGYYQFGYD
GEDFISFDLQTEQWIAAKQQAVITKQKWNQNRAEILYNKNYLTHECPEWLKKFLNYGRSS
LMRTERPSVSLLQKTPSSPVSCHATGFYPHRAALFWRKDGEQLHEDVDLGEILPNHDGTF
QMRVDLKLSSVPAEDWRRYDCVFQLSGVDEDIVTKLDKTRTNTGRSETRSGEGEKPAGST
FIIIIIAVAVLVVIIAAVVGFKVYRKRNGE

6b. Stickleback (*Gasterosteus aculeatus*) expressed translated matches and BAC genes:

>DN656534 *Gasterosteus aculeatus* cDNA 5', GA1+5+23 match
QTGGVHIVQLMFGCEWDDVTNEVKGYYQFGYDGEDFISFDLQTEQWIAPKQQAVITKQKW
EQNRALIAGEKNSLTHVCPQWLKKYLSYGRSSLMRTERPSVSLLQKTPSSPVSCHATGFY
PDRADLFWRKDGEELHEDVDLGEILPNHDGTFQMRVDLKLSSVPAEDWRRYDCVFQLSGV
DEDIVTKLDKTRTNTEKPAGSTSTSTFIIIIIIIAVAVLVVIIAAVVGFKVYRKKNAKCS
SAKPSSSSASTDDSEVTEELNPYPSDKFEEERLSGTN
>DN658147 *Gasterosteus aculeatus* cDNA 5', GA4+8+17+27 match
RKEPRQDWMSRVIEDDPQYWKRYTEILMGTQQVFKAGIETLKQRFNQTGGVHIVQRMYGC
EWDDETNEVKGYYQDGYDGEDFISFDLQTEQYIAPKQQAVITKQKWNQNRALIAQNKNFL
THVCPGWLKKYLSYGRSSLMRTERPSVSLLQKTPSSPVSCHATGFYPDRAALFWRKDGEE
LHEDVDLGEILPNHDGTFQMRVDLKLSSVPAEDWRRYDCVFQLSGVDEDIVTKLDKTRNN
TEKPAGSTFIIIIFIAVAVLVAIIAAVVGIKVYRNRNAKYSSVQCSSSSSSSGSNGGSDV
TEGTES
>DW035296 *Gasterosteus aculeatus* cDNA sequence GA9 match
RQDWMSRLTEDDPQYWKRNTENAMGHQQDFKGYIETVKQRFNQTGGVHIYQWMVGCEWDD
ETGEVKGYDQDGYDGEDFISYDLQTEQWIAAKQQAVITKRKLDHNRARIAGKKNYLTHVC
PEWVKKYLNYGRSSLMRTERPSVSLLQKTPSSPVSCHATGFYPHRAALFWRKDGEELHED
VDLGEILPNHDGTFQMRVDLKLSSVPAEDWRRYDCVFQLSGVDEDIVTKLDKTRTNTEKP
ADSTFIIIIIAVAVLVVIIAAVVGFKLYRKRNAKPSSSSSASTDGSEVTQELNPKP
>DW036315 *Gasterosteus aculeatus* cDNA sequence GA9 match,lacks TM
RLVGAEISVLSLLMISLHGAAALTHSLKFFYTASSGVPNFPEFVAVWLLDEVEFVHYDGD
TRRLEPRQDWMIRVTEDDPQFWKRNTELYMGCQQDFKGYIEIAKQSFNQTGGVHIYQKMA
GCEWDDDTNEVKGYVQYSYNGEDFISFDLQTDRWIAAKQQAVRIQQMLDQDRALTAHKKN
FLTHECPEWVKKYLSYGRSSLMRTERPSVSLLQKTPSSPVSCHATGFYPHRAALFWRKDG
EELHEDVDLGEILPNHDGTFQMRVDLKLSSVPAEDWRRYDCVFQLSGVDEDIVTKLGQTX
THTEKPAGSTSPSSSSSSSSSLWLFLSSSSLLWLNSRLQKENAPGSSF
>DN677624.1 *Gasterosteus aculeatus* cDNA clone 3', GA13 match
GQRLYSGCYDGEDFISFDWQTEQYIAAKQQAVITKRKWDHNRVKIAPWKNFLTHVCPEGL
KKFLKYGRSSLMRTERPSVSLLQKTPSSPVSCHATGFYPHRAALFWRKDGEQLHEDVDLG
EILPNHDGTFQMRVDLNLSSVPAEDWRRYDCVFQLSGMDEDIVTKLDKTRTNTGRPETRS
SEGEKPAGSTFIIIIIAVAVLVVIIAAVVGFKVYRKRNAKCSSAKCPSSSSSSSTDGSDV
TEELNPKPLKSASCTQRVAPRGHELFTAAVPQSCPQYEHKELLM
>DW605527.1 *Gasterosteus aculeatus* cDNA sequence GA15 match, lacks TM
MRLVGAEISVLSLLMMSLHGAAALTHSMKFFLTASSGVPNFPEFVIVGRL
DEVELFHYDSDTRRLEPRQDWVSRLTEDDPQYWKSGTELAMDAQQLFKVNIEIAKQRFNQ
TGGVHIYQRMAGCEWDNETNEVQGYIQDGYDGEDFISFDLQTEQYIAAKQQAVITKQKWD
QDRALIAQNKNFLTHVCPQWLKKYLSYGRSSLMRTERPSVSLLQKTPSSPVSCHATGFYP
DRAALFWRKDGEQLHEDVDLGEILPNHDGTFQMRVDLNLSSVPAEDWRRYDCVFQLSGVD
EDIVTKLXQTRTXREEPAXLTSSSSSLLWLVLSSSSLLGLEFRFTERRN
>DW039424.1 *Gasterosteus aculeatus* cDNA sequence GA15 match, lacks TM
VLSLLMMSLHGAAALTHSLKYFYTGSSGVPNFPEFVSVGLLDEVEFVHYDSNTRRAEPRQ
DWMSRVTEDDPQFWKSQTELAMDTQQDFKGHIETAKQSFHQTGGVHIYQRMYGCEWDDET
NEVKGYDQYGYDGEDFISFDLQTKRWIPAKQQAVITKQKLDHDSADKAYRKNFLTHVCPE
WLKKYLNYGRSSLMRTERPSVSLLQKTPSSPVSCHATGFYPDRAALFWRKDGEQLHEDVD
LGEILPNHDGTFQMRVDLNLSSVPAEDWRRYDCVFQLSGVDEDIVTKLDKTRDHREKPAG
STSSSSSSSLWLVLSSSSLLWVGSRVTERRTPNSLSGNALLLLLLSALSSLKD
>DT950082 Gasterosteus aculeatus 5', cDNA sequence GA18_Z lineage match

MSPLAVLVLLQVTAVVFGGKHSLTYTYTAFAHKPVGLPGIHEFTAMGHLDTHMIDYFDSD
RQEKVPKQPWMGERLDKDYWVKGTQSRQSKQQWFKVNIGILMDRLKQNKTSGQHVLQWQH
GCEGETQPDGTLKFSTGVDMYSYDGYDFLSFDDSNSAWVAGAQAAQQTKRTWDGVDVLKE
YTKGYLEKECMEWMGKFLTYQXRVLXSAKKPEVHLFASKAKKEAHVILTCMATXFLPKDI
ELWIQRNGRVLRREDGVMSSGSRPNGDETFQKRDWVEILKTDQSQYTCEVIHEGSGVNIQ
KEWDGKLLKTXNPXIGWGWSWAPCPPVGGWMLIFLTGKEYSPLTSKKGVNDP
>DW600052.1 *Gasterosteus aculeatus* cDNA clone 5', GA20+21+30 match
MRLVGAEISVLALLMMSLHGAAALTHSLKFFYTGSSGLPNFPEFVNVGLLDEVEMFHY
DSNTRRSEPRQDWMSRVTEDDPQYWKRQTEKSKDTQQVFKVDIETAKQRFNQTGGVHIVQ
LMIGCEWDDVTNEVKGYNQYGYDGEDFISFDLQTEQWIAPKQQAVLTKQKWDHNRALKAH
DKNYLTHECPEWLKKFLNYGRSSLMRTERPSVSLLQKTPSSPVSCHATGFYPHRAALFWR
KDGEQLHEDVDLGEILPNHDGTFQMRVDLKLSSVPAEDWRRYDCVFQLSGVDEDIVTKLG
QNRDHRGEPAGSTFIIIIIIAVAVLVVIIAAVVGIQVYGKRTANALRPTLFLFFCFTDG
SDVPEELDPKP
>DW026036.1 *Gasterosteus aculeatus* cDNA clone 5', GA20+21+30 match
DIGTAKRRFNQTGGVHIVQLMIGCEWDDVTNEVKGYNQYGYDGEDFISFDLQTEQWIAPK
QQAVLTKQKWDHDRALKAHDKNYLTHVCPEWLKKFLNYGRSSLMRTERPSVSLLQKTPSS
PVSCHATGFYPHRAALFWRKDGEQLHEDVDLGEILPNHDGTFQMRVDLKLSSVPAEDWRR
YDCVFQLSGVDEDIVTKLDKTRTNTEKPAASTFIIIIIIXVAVLVVIIVAVVGFKVHRK
RNAKCPSSTDGSEKSLRDQLNPKPLKSASCTXFMXEGCSGSQXIAPDLLHKLSFLAHLSF
>DW676054.1 EST with cytoplasmic exon 6 and 7 sequences
RRYDCVFQLSGVDEDIVTKLDKTRTNTEKPADSTFIIIIIAVAVLVVIIAAVVGFKLYRK
RNAKPSSSSSASTDGSEVTQELNPKPRSRRPAHSLWVNIALDQQAMPHILLHKLSIYGSS
FI
>DW676150.1 EST with stop codon prior to exon 7
GCEWDDETNEVKGYDQYGYDGEDFISFDLQTKRWIPAKQQAVLTKQKWDQDRAEIAHRKN
FLTHVCPEWLKKFLSYGRSSLMRTERPSVSLLQKTPSSPVSCHATGFYPDRAALFWRKDG
EQLHEDVDLGEILPNHDGTFQMRVDLKLSSVPAEDWRRYDCVFQLSGVDEDIVTKLDKTR
TNTEKPAGSTFIIIIIAVAVLVVIIAAVVGFKVYRKRNAKRSPSSSTVGSELSEELNPNP
_SRRPAHSLWVNIALDQQAMLHILLHKLSIYGSSFIQTSESGLVNIWKL
>DN715043.1 EST with exon 6 and 7, but with frame shift in exon 7
TSGVKFPFGGEYWVFFPRRSLQGVKAGSPGEDFLRGSLGSQISKIWNFWLRNKLKVILLA
GPQERRTQPGLDEQGLRGRSSILGRFRLSSVWDSSRFLKGPHETAKQSFHQTGGVHIYQR
MAGCEWDDVTDEVKGYNQDGYDGEDFISFDLETGQLIAAKQQAVRIKQKWDQNRAVKAGY
NNYLTRVCAEGLKKFLNYGRSSLMRTERPSVSLLQKTPSSPVSCHATGFYPDRAALFWRK
DGERLHEDVDLEEILPNHDGTFQMRVDLKLSSVPAEDWRRYDCVFQLSGVDEDIVTKLDK
TRTNTEKPAGSTFIIIIIAVAVLVVIIAAVVGFKVYRKRNAQRSSSSSSSAVGSDVTEELN
PKPLKLASCTQFMGECCSGSTSDAPHSSP
>DT959454.1 EST with exon 6 only
QLHQDVDLGEILPNHDGTFQMRVDLKLSSVPAEDWRRYDCVFQLSGVHKDIVTKLDKTRT
NTEKPAASTFIIIIIAVAVLVVIIAAVVGFKVYRKRNAKCSSAKPSSSSSASTEGSDVTE
ELNPKP
>DW664617.1 EST with exon 6 only
AESQRLIFSIEGGTLRSIWETSRSSKATIETAKQRFNQTGGVHMXQYMYGCEWDDETDEV
KGYHQYGYDGEDFISSDLQTEQYTAAKQQAVITKQKWDQDRAVTAHWKNYLTHVCPEGLK
KLLSYGRSSLMRTERPSVSLLQKTPSSPVSCHATGFYPDRASLFWRKDGEQLHEDVDLGE
ILPNHDGTFQMRVDLKLSSVPAEDWRRYDCVFQLSGVDEDIVTKLDKTRTNREKPADSTF
IIIIIIAVAVLVVIIAAVVGFKVYRKRNAKYSSAKCPSDKSEEESLSGTN
>GA_UAA ABN14358.1 *Gasterosteus aculeatus*, from BAC sequence, Schaschl & Wegner, Immunogenetics59;295-304, 2007
MRLVGAEISVLSLLMMSLHGAAALTHSLKYFLTASSGLPNFPEFVIVGLLDEVELFHYDG
DTRRAEVRQDWMIRVRGDDPRYLKRGTEVLMDAQQVFKVNIEIAKQRFNQTGGVHIFQRM
VGCEWDNETNEFKGYDQFGYDGEDFISYDLQTEQCIAAKQQAVITKQKWDQDRALKAHKK
NSLTHVCPESLKTLLNYGRSSLMRTERPSVSLLQKTPSSPVSCHATGFYPDRADLFWRKD
GEELHEDVDLGEILPNHDGTFQMRVDLNLSSVPAEDWRRYDCVFQLSGVDEDIVTKLDKT
RTNREKPAGSTFIIIIIIAVAVLVVIIVAVVGFKVYRERN_AKHSSSSSSSAVGSEL_GL
WVNIALDQQAMLHILLHKLSIYGSFIFHLNV
>GA_UBA ABN14357.1 *Gasterosteus aculeatus*, from BAC sequence, Schaschl & Wegner 2007
MRLVGAEISVLSLLMMSLHGAAALTHSMKYFFTGSSGVPNFPEFVAVGRLDEVEVVHYDG
NTRRAEPRQDWMIKVIDDDPQYWKSRTELAMDTQQAFKGYIEIAKQSFNQTGGVHIYQRM

AGCEWDNETNEVKGYIQDGYNGEDFISFDLQTDRWIAAKQQAVRIQQMLDHNRAFKAQNK
NFLTHVCPEGLKKFLSYGRSSLMRTERPSVSLLQKTPSSPVSCHATGFYPDRADLFWRKD
GEELHEDVDLGEILPNHDGTFQMRVDLKLSSVPAKDWRRYDCVFQLSGVDEDIVTKLDKT
RTNTGRSETRSGEGEKPAASTFIIIIIAVAVLVIIAAVVGFKVHRKRNDDYLCSTPPPTV
QESLTVRSCAIKAAHFLIDCTTAPVEALVSWISPLLAEEDHRSRARAR
>GA_UAC ABN14356.1 *Gasterosteus aculeatus*, lacks TM, from BAC sequence, Schaschl & Wegner 2007
MRLVGAEISVLSLLMMSLHGAAALTHSLKYFLTASSGLPNFPEFVAVGLLDEVEIVHYDS
NTRRGEPRQDWMIRVTEDDPQYWKSQTEILMDDQQEFKASIEIAKQRFNQTGGVHIIQNM
YGCEWDDETRAVKGYDQYGYDGEDFIAFDLQTEQYIAPKQQAVRIKQKWDQDRAWIAYNK
NYLTYLCPEWVKKYLIYGRSSLMRTERPSVSLLQKTPSSPVSCHATGFYPHRAALVWRKD
GEELHEDVDLGEILPNHDGTFQMRVDLKLSSVPAEDWRRYDCVFQLPGVHEDIVTKLDKT
RTNTGRSETRSGEGGKQTSTLVHFFVKKFSHPFVNSFQVRALFESMLNYCYV

7a. Tetraodon (*Tetraodon nigroviridis*) Ensembl genes:

>TN1_U ENSTNIG00000009992 Chr.7:4,474,277-4,475,964
MFYTCIFICLFSVTDHLFAEKHYLKYYLTASSGVTNQPEFVAQLVVDNLPGGYCDSNGKI
PQPRDDWAIKMIHEDPKQLELYENECVRYQHTYRGHIKNIKQQFNQSGGIHIFQRIHGCE
WDDETGEVTAFNQFGYDGEDFISLDPVTMTWVAAKPQALITKYKWDSEIIYLQIKKMFNS
HLCPDRLKMYLQFLKRFQQKKVQPSVFLLQKTPSSPVSCHVTGFHPENGVVFWRKDGQKL
QEEVEYTEILPNHDGTFQTRVDLELSSVSPEEWSRYECVFHPPGDQEDITLKLDKDVIRT
NWGKTGSACCVICQSETSLLKCV
>TN2_U No Ensembl gene ID, Chr.7:4.476.384-4.479.173
MHWQIVLLLLFYSGSAVKHSLRYFITSTSGVTEFPDFVAQTVVDNFQGGWCENNQAQPRN
EWAKKIAEENPKEVKYHLDSCVYLTQLYRAYVEDLKQQFNQSEAQPSVFLLQKTPSSAVS
CHVTGFHPENGVVFWRKDGQKLQEEVEYTEILPNHDGTFQTRADLELSSVSPEEWSRYEC
VFHPPGDQEDITLKLGSSVQTPLTLHPS
>TN3_P No Ensembl gene ID, UN_RANDOM: 6,397,631-6,399,093
GSHSLGFLSTGRFHPGTGLYFEQVTVFDGVLISHCDSRTPLEQFKAVLESHNLIRTCGNA
QNDVFDALTEVSKFIKSPLYVQRRRGCTRSAEGLNSAFEIWAVNGEDFIQFDADHQEWEA
LADTALPIRDSWNNRETRNHVFGSFLKDQCPEMIQRIKLREVEQRTDLRVFATPIDRSQT
LLKCHVTSTDKSLRSLSLTEDGASRALWVAVRGPLPSVDGSVVLRLTAEVPLGYSNIYGC
VVQTGGGTITVMWDGNTLDGRDIIYISDNWISLYILGSLAFLGALLVCVIHRWCSRLVSL
HLL
>TN4_P No Ensembl gene ID, UN_RANDOM:6,406,602- 6,407,214
GFHSLGFLSTGRFPPGTCPDFEQVTVFDGVLISHCDSRTQQEQFKAVLESHNLTRTCEEA
QYDVYDALKEISKFINDVQRRRGCTRSAEGLNSAFEIWAVNGEDFIQFDADHQEWEALAD
TALTIRDSWNNQEIRNLVSRSFLKDQCPEMIQRIKLREVEQKT
>TN5_P No Ensembl gene ID, UN_RANDOM: 6,412,622- 6,415,839
MGSREKTHTRLLVLWAFFLSAAECGSHSLDFLSTGRFHPGTGLYFEQVTVFDGVLISHCD
SRTQQEQFKAVLESHNLIRTCENAQYDVSDALREISKFINSTVDVQRRRGCTRSAEGLNS
AFEIWAVNGEDFIQFDADHQEWEALADTALTVRDSWNNLETRNQVFGSFLKDQCPEMIQR
INLTEVEQRTDLRVFATPIDRFQTLLKCHVTSTDKSLGSLSLTEDGASRALWVAVRGPLP
SVDGSVVLRLTAEVPLGYSNIYGCVVPTGGGTITVMWDGKTLDGRDIFYASIHWISLCIL
VFIVFALVLLVCAIQRWCCFKVSL
>TN6_P No Ensembl gene ID, UN_RANDOM: 6,420,283-6,420,651
QFDADHQEWEALADTALTIRDSWNNLETRNVFGSFLKDQCPQMIKKIKLREVEQRTDLRV
FATPIDRFQTLLKCHVTSTDKSLRSLSL
>TN7_P No Ensembl gene ID, Un_random 6,421,741- 6,422,362
GSHSLGFLFTGQFPPGTGPDFEQVTVFDGVLISHCDSRTQQEQFKAVLESHNLIRTCEEA
QYDVSDALREVSKFINSSLDVQRRRGCTRSAEGLTSAFEIWAVNGEDFIQFDADHQEWEA
LADTALTTRDSWNNQETRNQVFGSFLKDQCPQMIQRTSLTEVEQRT
>TN8_U No Ensembl gene ID, UnR:16,359,958-16,359,902 (NNN MHCI region)
CSDPPAEGHPEVKEPLKPA
>TN9_U ENSTNIG00000003024 Un_random:41,327,814-41,329,045
SLPVTHTLKYFYYRSSGVPNFPEFLAVVMVDDVQISHCDSNTRRAEPRQEWMEKVTADDP
QYWERETAKFLDAQQTYNARIEILKPSFNQTGAGVHVYQSTYGCEWDDETKKINGFSQGG
YDGEDLLRFKLKEAICAAAKPEAEILKRSGDEKKAEMESLKYYLTHECVYWLKKYLDYGR
SSLMRTELPSVSLLQKEPSSPVTCMATGFYPGGATLSWRRGEEELHEEVEPGEILPNPDG

TFQMSAALDLS
>TN10_U No Ensembl gene ID, Un_random:41.339.884-41.345.128
KSYYNQNCIKWLKKYVIYRRSSLRGTELPSVSLLQKSPSLISCHATGFYPGGATLSWRRG
EEELHEEVEPGEILPNPDGTFQMSAALDLSSVPPEDWSSYKCVFQLSGGQEIPTSLHRKR
IRTNWEENPSITSNTTAIIAVAVAVAVAVAVLVLVLAIAGLIVYRQKKVRDLQEVLEGHK
>TN11_U No Ensembl gene ID, Un_random:42.551.598-42.552.372
SLPVIHTLKYFYYRSSGVPNFPEFSGAALLDGAEIVHYDSDTRRAEPRQEWMENLSAGDP
QYWERETAKFLGSHQTYKARTEILKPRFNQTGSGVHIFQNMYGCEWDDETDERNGFDQWG
YDGEDLISFNLTEEIWVAAKPEAEIITHTWNQSRAEMEHWKNYLTR
>TN12_U ENSTNIG00000001973 Un_random:46,457,180-46,457,895
GVHIFQWMYGCEWDDEIDERNWFCQWGYDGEDFISFKLKEEIYVAAKREAEIITHRWNQN
RALIEQQKYYYQICIKWLQKFVDYGRSSLMRTELPSVSLLQKEPSSPVTCMATGFYPGGA
TLSWRRGEEELHEEVEPGEILPNPDGTFQMSAALDLSSVPPEDWSSYKCVFQLSGGQEVP
TSLDRKRITTNWEG
>TN13_Z ENSTNIG00000003449 Un_random:59,040,025-59,042,948
MVLLVLLGSGLVVDGGLSEIHSLHYIYTALSRPVNRPGIHEFTAMGMLDDHMIDYFDSDK
QVKVPKQTWMKTELEENYWEKGTQSRKSKQQWFKVNIKILKDRFRQNDSDIHVLQWVHGC
NIDTSGNETEFLHGIDMYSYDGESFLTFDEANENWVAPNDAALQTKRKWDDLQVLKEYTK
VYLKKECVTWLERFLKYQKENTAPVKPAVYALSREANVQANIVLTCMATGFSSINTIVQI
KRDGLVLTKDDGDVRPNGDGTYQKRDQVEIPKSDKSKYTCEVIHESSGLHEIRVWDTDEG
NTAVVVGAVVGVVVVVVLVIGIGLYMAVKK
>TN14_U ENSTNIG00000004145 Un_random:66,895,563-66,896,295
GVHTFQWMVGCEWDDETDEINGFDQYGYDGDDFLSFKLKDGTWVAAKREAEITKRKWDYD
GAGIDYWKYYHNQNCIKWLKKYLDYGRSSLMRTELPHSVSLLQKEPSSPVICMATVFYPD
GATLSWRRGEEELHEEVEPGEILPNPDGTFQMSAALDLSSVPPEDWSSYKCVFQLSGGQE
VHTSLDRKQIRTNWGKTGGGGDDGE
>TN15_U ENSTNIG00000004925 Un_random: 74,651,122-74,651,415
THTLKYFHTVSSGVPNFPEFVAVVMVDDVHISHYDSDTRRAQPRQEWMENVSAEHPQYWE
RETGNFMGAHQAYKAWIEILKPSFNQTGG
>TN16_U ENSTNIG00000001016 Un_random:91,605,519-91,607,947
VTVSSGIPNLPEYVVVGMLDDVQTDHYDSITGRAEPRQEWRKKVKAEHPEDWEGQTQIAA
GIHQNFKATIEILKQRFNQTGAGVHIFQLMYGCEWDDETEERNGFHQWGYDGEDFISFKL
KEEIYVAAKREAEVSARRWNQDRAEIEQQKNYYQTCIKWLKKYLDYGRSSLMRTEPPPLS
VPSSRKEPSSPVTCVATGFYPGGATLSWRRGEEELHEEVEPGEILPNPDGTFQMSAALDL
SSVPPEDWSSYKCVFQLSGGQEVPTSLDKNPSITTAIVGVGVAVLVLVLGAIAGFIMYRQ
KKGESWR
>TN17_U No Ensembl gene ID, Un_random:91,609,131- 91,610,586
SVNHTLKILFTASSGIPNLPEYVIVGLMGDVQMFHYDSDTGRAEPRQEWVEKVKADDPDY
WEEQTRIAVGEQQNFKASIENIKPRFNQTGSGVHVLQRTFGCEWADETDERNWFDQYGYD
GEDFISFKLTEEIYVAARREAEIITHTWNQNRAAIEHQKSYNQNCIEWLKK
>TN18_U No Ensembl gene ID, UnRandom:91,614,129-91,615,547
SGVYTVQFIYGCEWDDETGKSGGIYKFGYGGEDLLSFNLKEETLVAAKPEAEIITQELNQ
DKRWIERLTRYFTYYCAKWLKKYVDYGRCSLMRTELPSVSLLQKEPSSPVTCVATGFYPG
GATLSWRRGEEELHEEVEPGEILPNPDGTFQMSAALDLSSVPPEDWSSYKCVFQLSGGQE
VPTSLDRKRIRTNVWVVFSPPENPSNTSITTAIVAVAVLVLVLGAIAGFLVYRQKGE
>TN19_U No Ensembl gene ID, UnRandom:91,620,258-91,620,889
FPLSYSSPAPTTPEFSSVSLLQKEPSSPVTCMATGFYPGGATLSWRRGEEELHEEVEPGE
ILPNPDGTFQMSAALDLSSVPPEDWSSYKCVFQLCGGQEVPTSLDRKWIRTNWVWVVFSP
PENPSNTSITTAIVGVAVAVLVLVLVLGTIAGFIVYRQKKGE
>TN20_U No Ensembl gene ID, Un_random 91,622,837- 91,625,870 pseudogene,
Orf error
SVNHTLKYLYTGSSGIPNLPEYVIVGLVDDVQTDHYDSITGRAEPRQEWRKKVKAEHPED
WEGQTQIAAGIHQNFKATIEILKQRFNQTGSGVHVLQRTYGCEWDDETDERNGFDQYGGY
DGEDFISFKLTEEIWVAARREAEIIKHKWDQERAWIEQLKTYFTQICIDWLKPFSPSLLP
QSPTQCPSSRRSPPLPSPAWLQASTLTGHTVLEERGGGAP*GGGARRVLPNPDGTFQMSA
ALDLSSVPPEDWSSYKCVFQLSGGQEVPTSLDRKRNRTNWEVWVVFSPPENPSITTAIVG
VAVAVLVLVLGAIAGFIVYRQKKGECSDPPAEGHPEVKEPLKPAA
>TN21_U No Ensembl gene ID, Un_random:91,632,905-91,635,261
VTHTLNYFHTVSSGVPNFPEFVAVVMVDDVHISHYDSNTRRTQPRQEWMENVNADDPQYW
EGETGNFMGAHQAYKARIEILKPRFNQTGSGVHTYQLMYGCEWDDETDEINGFNQYGYDA
EDFISFKLKEEIWVAAKREAEITKRKCDQNRANTDYLKSYYTQICIKWLQKFVDYGRSSL

MRTELPSVSLLQKEPSSPVTCMATGFYPDGATLSWRRGEEELHEEVEPGEILPNPDGTFQ
MSAALDLSSVPPKDWSSYKCVFQLSGGQEVPTSLDRKRIRTNWE
>TN22_U No Ensembl gene ID, Un_random:91,638,301-91,640,135
SLPVIHTLKYFYYRSSGVPNFPEFSGAALLDGAEIVHYDSDTRESEPRQEWMENLSAGDP
QYWERETAKFLGAQQSYKARTEILKPRFNQTGSGVHIFQNMYGCEWDDETDERNGFDQWG
YDGEDLISFNLTEEIWVAAKREAEITHTWNQSRAEMEHWKNYLTRECVYWLKEYVTYGRS
SLMRTLPSVSLLQKEPSSPVTCMATGFYPGGATLSWRRGEEELHEEVEPGEILPNPDGTF
QMSAALDLSSVPPEDWSSYNCVFQLSGGQEVPTSLDRKQIRTNWEVWVVFSPPENPSNTS
TIVRVVISVLVLVLGTIAGFLVYRQKKGECSDPTAEGHPEVREPLRP
>TN23_U ENSTNIG00000001085 Un_random:91,636,602-91,644,322
MKIAKFVVVLAALAVYRSRAVIHTLKYFYYRSSGVPNFPEFSGAALLDGAEIVHYDSDTR
RAEPRQEWMENLSAGDPQYWERETAKFLGAQQTYNARIEILKPRFNQTGAGVHIFQNMYG
CEWDDETDERNGFDQWGYDGEDLMSFNLTEEIWVAAKREAEITTHTWNQDRAEMEYWKNY
LTRECVSSLKTYLDYGRSSLMRTELPSVSLLQKEPSSPVTCMATGFYPGGATLSWRRGEE
ELHEEVEPGEILPNPDGTFQMSAALDLSSVPPEDWSSYKCVFQLSGGQEVPTSLDRKRIR
TNWENPSITSITSAIVGVAVLVLVLGAIAGFLVSRQRKGE
>TN24_U ENSTNIG00000000474 Un_random:91,647,017-91,647,905
GVHVLQRMYGCEWDDETNESLSGFYQYGYDGEDFISFNLTEEAWVAARREAEVTKRRWEK
HEGVKEHWKTYLTHRCISWVQEYVAYRQSSLVRTELPPSVSLLQKEPSSPVTCMATGFYP
GATLSWRRGEEELHEEVEPGEILPNPDGTFQMSAALDLSSVPPEDWSSYKCVFQLSGGQE
VPTSLDRKRIRTNWGKTGGGGDDGEKNGLVLLWEQRLLLFSS
>TN25_U No Ensembl gene ID, Un_random:91,656,239-91,657,762
NFLGAHQTYKAWIEILKPHFNQTGSGVHVYQSTYGCEWDDETDERNGFSQDGYDGEDLLR
FKLKEEIFVAAKPEAEILKRSGDEKKAEMESLKYYLTHECVYWLKKFVTYGRSSLMRTEL
PSVSLLQKEPSSSSPVTCMATGFYPGGATLSWRRGEEELHEEVEPGEILPNPDGTFQMSA
ALDLSSVPPEDWSSYKCVFQLSGGQEVPTSLDRKRIRTNWEVWVVFSPPENPSNTSAIVG
VVAAVLVLVLGAIAGFLVYRQKKGECSDPPAEGHPEVTEPLRPAA

7b. Tetraodon (*Tetraodon nigroviridis*) Expressed translated matches:

>TN_P lineage sequence from Brain SRX191169 Pufferfish brain; *Tetraodon nigroviridis* RNA-Seq, assembly of individual 76 bp 100% sequence ID reads TN3-7 P lineage match
STGRFPPGTGPDFEQVTVFDGVLISHCDSRTQQEQFKAVLESHNLIRTCEEAQYDVSDAL
TEISKFINSPLYVQRRRGCTRSAEGLNSAFEIWAVNGEDFIQFDADHQEWEALADTALTI
RDSWNNLETRNHVFGSFLKVQCPEMIQRIKLREVEQKTDLRVFATPIDRFQTLLKCHVTS
TDKSLRSLSLTEDGASRALWVAVRGPLPSVDGSVVLRLTAEVPLGYSNIYGCVVQTGGGT
ITVMWDGKTLDGRDIFYASNNWISLYILVFIVFLL
>CR726246 *Tetraodon nigroviridis* full-length cDNA TN13 Z lineage match
METTKSFMVLLVLLGSGLVVDGEKHSLHYIYTALSRPINRPGIHEFTAMGMLDDHMIDYF
DSDKQVKVPKQTWMKTELEENYWEKGTQSRKSKQQWFKVNIKILKDRFHQNDSDIHVLQW
VHGCNIDTSDNEIKFLHGIDMYSYDGESFLTFDEANENWVAPNNAALQTKRKWDDLQVLK
EYTKVYLKKECVTWLERFLKYQKENTAPVKPAVYTFSREAKVQTNIVLTCMATGFSSINT
IVQIKRDGVVLTNGDGGQSSGVRPNGDGTYQKRDQVEIPKSDKSKYTCEVIHESSGLHET
RVWDTDEGNTAVAMGAVVGVVVVVALLIGISLFVVVKK

8a. Pufferfish (*Takifugu rubripes*) Ensembl genes (Fugu4 genome assembly):

>TR1_U ENSTRUG00000017062 Scaffold_6:3,198,531-3,200,246
MLIITIMTQNKVKLFAAAEKHYLRYFLTASTGATNFQDFLAQVVADDLQGGYCDSNGAKP
RDEWAERIVEDDPEELKYYTDACVHYVHTYRAQVKNFNQQFNQSEGIHTFQRIHGCEWDD
KTGKVTAFNQFGYDGEDFIALDPETMTWVAAKPQALITKRKWDNEITYLEIKKIFNTETC
PKRLKKFLQYIQKFHQRDVQPSVFLLQKTPSSPVSCHATGFYPKVAVMFWRKDGEKLHEE
VEYMEILPNHDGTFQRSVDLKLSSVSPEEWSRYECVFQLPGVVEDIAIVLDTSVIRTNWG
KMAPKVSYWVVAGVVVVVLLLLASIMGYFIWKR
>TR2_U ENSTRUG00000017063 Scaffold_6:3,205,239-3,210,655

QGKKKHSLRYFLTESSGVTNLPEFVAQLMVDDLLGGYCDSNRKIPKPRDWAVKMILEDPK
QFEIYENECDRYQHKYRTQIKNLKEQFNQTEGIHIFQRIHGCEWDDETDKKTAFNQFGYD
GEDFIALDPETMTWVAAKPQALITKRNWDNEIIYLEIKKLFNAEICPKRLRKYLQYIQKF
HQRDVQPSVFLLQKTPSSPVSCHATGFYPKEAVMFWRKDGEKLHEEVEYMEILPNHDGTF
QRSVDLKLSSVSPEEWSRYECVFQLPGVVEDIAIVLDTSVIRTNWVPPAEFPTGVVAGVV
VVVLLLLASIMGYFIWKRPNK
>TR3_U ENSTRUG00000002296 Scaffold_61:4,244-8,738
MRVVLSLTVVFHLTSAVTHTLKYFYTGSFGVPNFPEFVAVGLVDDVPIIRYDSNTRRAQP
KQEWMKEVTADDPQYWSKSTQTLLFTQQTLKINMEIAEQRFNQTRGVHIIQKMCGCEWDD
ETGEKNGFDQFGYDGADFISLKLKEGIWVAGKREAEITKHKWDQDEAQIEQQKFYYTQVC
IHWLQKYVNYGKSSLMRTELPSVSLLQKTPSSPVSCHATGFYPDRATLSWRKGEEELHED
VDHGEILLNPDGTFQTSVDLKVSSVPPEDWSSYKCLFQLSGVKEITTTLDKNQIRTNWGK
SGAEDPSNTTVIAAVAVVVLALVLAAVVGFLLYRKKKVIVWN
>TR4_P No Ensembl gene ID, Scaffold_209:508,917- 510,010
HFKPVLVSHNLPGSCRPACYDVFEALKASSKFINHTRDVQRRRVCITSDERLVSAFDNWA
VNGEGFIQFDAGAQKWKALSPSAEMIKDSWNNREARNHFFGHFINEECQIKLREADKNTD
LRVFANPVDRTKALLKCHVTSTDKSVRSVSLTEDGAPKANWITVTGPLPSGDGSVILILT
AEVPLIHTNIYGCVVQTEDRNITVMW
>TR5_P No Ensembl gene ID, Scaffold_209:516,289-517,881
GSHSLEFLSTGRVQPGSGAHFEQVTEFDGVVISHCDSGTQQEHFKPVLESHNLPGTCRAA
CYDVFVALKEISQFINHTRVDRTKALLKCHVTSTDKSVRSVSLTEDGAPKANWITVTGPL
PSGDGSVILILTAEVPLIHTNIYGCVVQTEDRTITVMWDGNTLDGRHILYIHMTFWRIIG
IIFTVCCLISVMTLLCKS
>TR6_P No Ensembl gene ID, Scaffold_497:44,782-49,340
MNIGLLFWILLPAAECGSHSLEFLSTGRVQPGSGPHFEQVTEFDGVVISHCDSGTQQEHF
KPVLESHNLPGTCRPACYDVFDALKEISKFINHTRNVQRRRGCITSDERLESAFDNWAVN
GEGFIQFDAGAQKWKALSPSAEMIKDSWNGYEAQNHVFGQFIRRYCPEMIHQIKLRETEK
RTDLRVFANPVDLTKALLKCHVTSTDKSVRSVSLTEDGATKANWITVTGPLPSGDGSVIL
ILTAEVPLIHTNIYGCVVQTEDRNITVMWDGNTLDGRHILYAGVPMKFWIIIGIIFVCCL
ISVMTLLCKSHFFDCNFQVSL
>TR7_U ENSTRUG00000003539 Scaffold_585:69,748-72,643
MRKLLFMLLLSCKSASAVQPSLTIYYSLSSGLPNIPEFFASVEVSGLEAGYCDTTKKKVE
PKTNWAKTFLDHHQEQLDWYTAECVERFPAYMKYWMYNVKEIYNQTGAGVHVIQRLDHCE
LDSETGEISAFSKFGYDGEDLLELDLKTLHWTALTPKALAVKPRWDSDEHRTLWISLYIT
KTQQDQMNHHETNDGFLSVPPELPSVSLLQKTPSSPVSCHATGFYPDRATLSWRKGEEEL
HEDVDHGEMLLNPDGTFQMSVDLNVSSVPPEDWSSYKCVFQLSGAKEITTTLDKNQIRTN
WGETVSSSNHPGAAVIIVILVVLLLPVGVAIRFCIRTRNN
>TR8_Z ENSTRUG00000004298 Scaffold_2080:23,720- 25,204
WMHGCDADIDHGEPTFLHGIEKYSYDGANFLAFNENQEIWDARDDAARETKTRWDKVQVL
REYTMAYLQKECVTWLTRFLKYQNENEVPAKPDLYVFITGAKDPTNMVLKCMATGFTPTN
TVLQIKLGGRVLTREDGLHSTDILPNGDGTFQKTEYVEIPKSDNSDYYCELSHTPSSLRV
VKFWDKKAPDNTTTILLAVGIPVVVVILIGGLLILVIKK
>TR9_P No Ensembl gene ID, Scaffold_2108:8,529-12,205
MNICLLFWILLPAAECGSHSLEFLSTGRVQPGSGPHFEQVTEFDGVVISHCDSGTQQEHF
KPVLESHNLPGTCEVALNDAFGALKAISKFINHTRNVQRRRGCITSDERLESAFDNWAVN
GEGFIQFDAGAQKWKALSPSAEMIKDSWNSRESRNDFFGQFINEECPEMIHQIKLREAEK
RTDLRVFANPVDRTKALLKCHVTSTDKSVRSVSLTEDGAPKANWITVTGPLPSGDGSVIL
ILTAEVPLIHTNIYGCVVQTEDRNITVMWDGKTLAGVNGTFLAPILVSCVLLICATARML
KCKSHYFC
>TR10_P No Ensembl gene ID, Scf.2108:15,381-16,811 pseudogene, ORF error indicated by star
GSHSLEFLSTGRVQPGSGPHFEQVTEFDGVVISHCDSGTQQEHFKPVLESHNLPGTCRPA
CYDVFDALKAISKFTNHTRDVQRRRGCITSDERLESAFDNWAVNGEGFIQFDAGAQKWKA
LSPSAEMIKDS*NGYEARNHLFGQFIRRYCLEMIHQIKLREIEKRTDLRVFANPVDRTKA
LLKCHVTSTDKSVRSVSLTEDGAPKANWITVTGPLPSGDGSVILILTAEVPLIHTNIYGC
VVQTEDRTITVMWDGNTLDGREILSTSVSWTQILGCVLFLCVLFLICTTALVLK
>TR11_P No Ensembl gene ID, Scaffold_2108:22,382-22,702 Pseudogene
DLRVFANPVDRTKALLKCHVTSTDKSVRSVSLTEDGAPKANWITVTGPLPSGDGSVILIL
TAEVPLIHTNIYGCVVQTEERNITVMW
>TR12_U ENSTRUG00000001978 Scaffold_2168:9,134-10,367
MLLKPDGTFQMSVDLKVSSVPPEDWSSYKCVFQLSGGKEITTTLDKNQIRTNWTEDPSNT

AVIAAVAVVVLVLVLAAVVGFLLYRKKKVSGKCTKTEGHSEVKVPLKPNPN
>TR13_U ENSTRUG00000002529 Scaffold_2183:6,228-13,427
MKSLDFLLLLALLSLPDSSAVTHTLKYFYTASSGVPNFPEFVAFGMVDDVQMVRYDSNTG
RLQFKQEWMKEATADDPQFWDKKTGILMGAQQWFKVNIEILKQRFNQTGAVGGDKHQHMY
GCEWDDETREKNGFFQDGYDGADFISLKLKEGIWVAAKREAEISKHKWDQDEALIEQLMD
YYTQICIKWLQKFVNYGKSSLMRTELPSVSLLQKTPSSPVRCHATGFYPDTAVMSWRKGE
EELHEDVDHGEMLLNPDGTFQASVDLNVSSVPPEDWSSYKCVFQLSGGKEINTTLDKNQI
RTNWTEDPSNTAVIAAVAVVVLALVLIGVVGFLLYRKKKVAGKCTKPEGHSEVKVPLKPN
PI
>TR14_P No Ensembl gene ID, Scaffold_2190:6,635-8,502
GSHSLEFLSTGRVQPGSGPHFEQVTEFDGVVISHCDSGTQQEHFKPVLESHNLPGTCRPA
CYDVFDALKEISKFINHTRNVQRRRGCITSDERLESAFDNWAVNGEGFIQFDAGAQKWKA
LSPSAEMIKDSWNGYEVQNYLFGQFIKEQCPEMIHQIKLREVEKRTDLRVFANPVDLTRA
LLKCHVTSTDKSVRSVSLTEDGATKANWITVTGPLPSGDGSVILILTAEVPLIHTNIYGC
VVQTEDRDITVMWDGNTLDGREILYATVDWPFWKILTIVTFSCVFLLICTTAVVLKHNEK
KIKRRMKKRIPGSKRETEKENLRMSLVMKPLQDQKIN
>TR15_P No Ensembl gene ID, Scaffold_2190:12,606-15,159
MNICLLFWILLPAAECGSHSLEFLSTGRVQPGSGPHFEQVTEFDGVVISHCDSGTQQEHF
KPVLESHNLPGTCEVALNDAFGAIKEISKLINHTLYVQRRRGCITSDERLESAFDNWAVN
GEGFIQFDAGAQKWKALSPSAEMIKDSWNGYEARNHLFGQFIRRYCLEMIHHIKLREIEK
RTDLRVFANPVNRTKALLKCHVTSTDKSVRSVSLTEDGAPKANWITVTGPLPSGDGSVIL
ILTAEVPLIHTNIYGCVV
>TR16_P No Ensembl gene ID, Scaffold_2440:1,665-4,373
MNICLLFWILLPAAECGSHSLEFLSTGRVQPGSRLHFEQVTEFDGVVISHCDSGTQQEHF
KPVLESHNLPGTCRPACYDVFDALKAISKFTNHTRNVQRRRGCITSDERLESAFDNWAVN
GEGFIQFDAGAQKWKVLSPSAEMIKDSWNNREARNHFFGQFIRRYCLEMIHQIKLKETEK
RTDLRVFANPVDRTKALLKCHVTSTDKSVRSVSLTEDGAPKANWITVTGPLPSGDGSVIL
ILTAEVPLIHTNIYGCVVQTEDRTSLSCG
>TR17_P No Ensembl gene ID, Scaffold_2627:1,321-3,402
MNICLLFWILLPAAECGSHSLEFLSTGRVQPGSGLHFEQVTEFDGVVISHCDSGTQQEHF
KPVLESHNLPGTCTAAFYDVFDALKEISKFINHTRNVQRRRGCITSDERLESAFDNWAVN
GEGFIQFDAGAQKWKALSPSAEMIKDSWNGYEARNHFFGHFINEECPKMIHQIKLREVEK
RT
>TR18_P No Ensembl gene ID, Scaffold_2628:2,060-8,810
MNICLLFWILLPAAECGSHSLEFLSTGRFQPGSGPHFEQVTEFDGVVISHCDSGTQQEHF
KPVLESHNLPGTCRPACYDVFDALKEISKFINHTRNVQRRRGCITSDERLESAFDNWAVN
GEGFIQFDAGAQKWKALSPSAEMIKDSWNGYEPRKHVFGQFIRRYCLEMIHQIKLREIEK
RTDLRVFAHPVDRTKALLKCHVTSTDKSVRSVSLTEDGAPKANWITVTGPLPSGDGSVIL
ILTAEVPLIHTNIYGCVVQTEDRNITVMWDGKTLDGRHIPHASVDWTFLKVFTSVLLPFY
FLSCITSLLLKSK
>TR19_U ENSTRUG00000002033 Scaffold_2693:10,282-11,984
CELDSETGEISAFSKFGYDGEDLLELDLKTLHWTALTPKALAVKPRWDSDEHRTLRISLY
ITKVPERMKQYSAIMKEHLERTELPSVSLLQKTPSSPVSCHATGFYPDRATLFWRKGEEE
LHEDVDHGEMLLNPDGTFQMSVDLKVSSVPPEDWSSYKCVFQLSGAKEITTTLDKNQIRT
NWGETGEPPGILISYFVIGILAA
>TR20_U ENSTRUG00000000486 Scaffold_2708:7,649-9,458
GVHIAQRMYGCEWDDETGEKNGFRQDGYDGADFISLKLKEGIWVAGKREAEITKHKWDQD
EAQIEQQKFYYTQVCIHWLQKYVNYGKSSLMRTELPSVSLLQKTPSSPVSCHATGFYPDR
ATLSWRKGEEELHEDVDHGEMLLNPDGTFQMSVDLKVSSVPPEDWSSYKCVFQLSGGKEI
TTTLDKNQIRTNWRKPWVRGDGAEDPSNTAVIAAVAVVVLVLVLAAVVGFLLYRKKKVIV
CNCGFCSKNLCSDLTAKGHSEVKEPLKPNP
>TR21_Z ENSTRUG00000003955 Scaffold_3060:3,567-5,990
EKHSLHYIYTATSQPISRQGIHEFTAMGVLDGNVIDYFDSDAQKKVPRQKWMEKELDKSY
WEKGTESRKSKQQWFKVNINILKTRYRQNDSDIHILQWMHGCDADIDHGEPRFLHGIDKY
SYDGANFLAFNENQEIWDARDDAARETKTRWDKVQVLREYTMAYLQKECVTWLKRFLDYQ
NKNEIPAKPDLYVFITGAKDPTNMVLKCMATGFTPTNTVLQIKLGGRVLTREDGVNSTDI
LPNGDGTFQKTDYVEIPRSDNSDYYCELSHTPSSLRVVKFWDKKAPDDTTKILLAVRISL
GAVILIGGLLILVIKKFCIG
>TR22_P No Ensembl gene ID, Scaffold_3394:4,970-7,539
MKFCLLLYWVLLPAAECGSHSLEFLSTGRVQPGSGPHFEQVTEFDGVVISHCDSGTQQEH
FKPVLESHNLTGTCRPACYDVFDALKAISKFINHTRNVQRRRGCITSDERLESAFDNWAV

NGEGFIQFDAGAQKWKVLSPSAEMIKDSWNGYEARNHLFGQFIRRHCLEMIHHIKLREIE
KRTDLCVFANPVDRTKALLKCHVTSTDKSVRSVSLTEDGAPKANWITVTGPLPSGDGSVI
LILTAEVPLIHTNIYGCVVQTEDRTITVMWDGNTLDGREILSTSVSWTIILGCVLFLFVL
FLICTTALVLKCTSKYHYSKCNI
>TR23_P No Ensembl gene ID, Scaffold_3485:6,558-6,833
LRVFANPVNRTKALLKCHVTSTDKSVRSVSLTEDGAPKANWITVTGPLPSGDGSVILILT
AEVPLIHTNIYGCVVQTEDRNITVMW
>TR24_P No Ensembl gene ID, Scaffold_3928:258-976
GSHSLEFLSTGRVQPGSGAHFEQVTEFDGVVISHCDSGTQQEHFKPVLESHNLIGTCTAA
LNDGFDALKAISKFINHTRNVQRRRGCITSDERLESAFDNWAVNGEGFIQFDAGAQKWKA
LSPSAEMIKESWNGYEAQNHFFGQFINEECPEMIHHIKLREAEKRT
>TR25_P No Ensembl gene ID, Scaffold_3975:600-7,309
VQRRRGCITSDERLESAFDNWAVNGEGFIQFDAGAQKWKALSPSAEMIKDSWNGYEPRKH
VFGQFIRRYCLEMIHQIKLREIEKRTDLRVFANPVDRTKALLKCHVTSTDKSVRSVSLTE
DGASKANWITVTGPLPSGDGSVILILTAEVP
>TR26_P No Ensembl gene ID, Scaffold_4784:5,138-6,243
GSHSLEFLSTGRVQPGSGPHFEQVTEFDGVVISHCDSGTQQEHFKPVLESHNLPGTCEVA
LNDAFGALKEISKLINHTLYVQRRRGCITSDERLESAFDNWAVNGEGFIQFDAGAQKWKA
LSPSAEMIKDSWNNREARNHFFGHFINEECPEMIHQIKLREVGKKNDLRVFANPVDRTKA
LLKCHVTSTDKSVRSVSLTEDGAPKANWITVTGPLPSGDGSVILILTAEVPLIHTNIYGC
VVQTEDRNITVMW
>TR27_P No Ensembl gene ID, Scaffold_5016:1,270-3,336
GSHSLEFLSTGRVQPGSGPHFEQVTEFDGVVISHCDSGTQQEHFKPVLESHNLPGTCEVA
LNDAFGALKAISKFINHTRDVQRRRGCITSDERLESAFDNWAVNGEGFIQFDAGAQKWKA
LSPSAEMIKDSWNSRESRNDFFGQFINEECPEMIHQIKLREAEKRT
>TR28_P No Ensembl gene ID, Scaffold_7638:1,280-3,354
MNICLLFWILLPAAECGSHSLEFLSTGRVQPGSGLHFEQVTEFDGVVISHCDSGTQQEHF
KPVLESHNLPGTCTAAFYDVFDALKAISKFINHTRNVQRRRGCITSDERLESAFDNWAVN
GEGFIQFDAGAQKWKALSPSAEMIKDSWNSYETRNHLFGQFIRRYCLEMIHHIKLREIEQ
RT
>TR29_P No Ensembl gene ID, Scaffold_7656:92-2,346
GSHSLEFLSTGRVQPGSGPHFEQVTEFDGVVISHCDSGTQQEHFKPVLESHNLTGTCRPA
CYDVFDALKAISKFINHTRNVQRRRGCITSDERLESAFDNWAVNGEGFIQFDAGAQKWKV
LSPSAEMIKDSWNSYEARNHLFGQFIRRYCLEMIHQIKLKETEKRTDLRVFANPVNRTKA
LLKCHVTSTDKSVRSVSLTEDGAPKANWITVTGPLPSGDGSVILILTAEVPLIHTNIYGC
VVQTEDRNITVMWDGKTLDGREILYATVVWPLWKILTIVTFSCVCLLICTTALVLKHNEK
KIKRRMKKRIPGSMTETEKENLRMSLVMKPLQDPKIN
>TR30_P No Ensembl gene ID, Scaffold_8439:1,979-3,842
MNICLLFWILLPAAECGSHSLEFLSTGRVQPGSGPHFEQVTEFDGVVISHCDSGTQQEHF
KPVLESHNLPGSCRPACYDVFDALKAISKFINHTRDVQRRRGCITSDERLESAFDNWAVN
GEGFIQFDAGAQKWKALSPSAEMIKDSWNGYEARNHLF
>TR31_P No Ensembl gene ID, Scaffold_8559:3-1,449
NGEGFIQFDAGAQKWKVLSPSAEMIKDSWNNREARNHFYGHFISKQCPEMIHQIKLKEIE
KRTDLRVFANPVDRTKALLKCHVTSTDKSVRSVSLTEDGAPKANWITVTGPLPSGDGSVI
LILTAEVPLIHTNIYGCVVQTEDRTITVMWDGNTLDGRHILYIHMTFWRIIGIIFTVCCL
ISVMTLLCKSHFFDCNFQVSL
>TR32_P No Ensembl gene ID, Scaffold_9266:50-2,297
MNICLLFWILLPAAECGSHSLEFLSTGRVQPGSGPHFEQVTEFDGVVISHCDSGTQQEHF
KPVLESHNLPGTCEVALNDAFDALKEISKLINHTLYVQRRRGCITSDERLESAFDNWAVN
GEGFIQFDAGAQKWKALSPSAEMIKDSWNGCEARNHFFGQFINEECPEMIHQIKLREVEK
RT
>TR33_P No Ensembl gene ID, Scaffold_9833:1,141-2,380
DLRVFANPVNRTKALLKCHVTSTDKSVRSVSLTEDGAPKANWITVTGPLPSGDGSVILIL
TAEVPLIHTNIYGCVVQTEDRNITVMWDGNTLDGRHILYIQMTFWRIIGNIFTVCCLISV
MTLLCKSHFFDCNFQLSL
>TR34_P No Ensembl gene ID, Scaffold_9991:1-1,073
RTCQPGSRPHFEQVTEFDGVVISHCDSGTQQEHFKPVLESHNLPGTCRPACYDVFDALKE
ISKFINHTRNVQRRRGCITSDERIESAFDNWAVNGEGFIQFDAGAQKWKALSPSAEMIKD
SWNGYETRNHLFGQFIRRYCPEMIHQIKLRETEKRTDLRVFANPVDRTKALLKCHVTSTD
KSVRSVSLTEDGAPKANWITVTGPLPSGDGSVILILTAEVPLIHTNIYGCVVQTEDRNIT
VMW

8b. Pufferfish (*Takifugu rubripes*) Expressed translated matches:

```
>Fugu_SRA, assembly of sequence reads from Gill shotgun transcriptome
library SRX363279. TR14 P lineage match
FWILLPAAECGSHSLEFLSTGRVQPGSGPHFEQVTEFDGVVISHCDSGTQQEHFKPVLES
HNLPGTCRPACYDVFDALKEISKFINHTRNVQRRRGCITSDERLESAFDNWAVNGEGFIQ
FDAGAQKWKALSPSAEMIKDSWNGYEARKHVFGQFIRRYCPEMIHQIKLKETEKRTDLRV
FANPVDLTRALLKCHVTSTDKSVRSVSLTEDGATKANWITVTGPLPSGDGSVILILTAEV
PLIHTNIYGCVVQTEDRNITVMWDGNTLDGREILYATVDWPFWKTLTIVTFSCVFLLICT
TAVVLKYNEKKIKRRMKKRIPGSKT
>CK829467 fast skeletal muscle Takifugu rubripes cDNA clone, TR20 U lineage
match
LKEGIFVAAKREAEITKHKWDQNEALIERQKSYYTQICIEWLQKYVNYGKSSLMRTELPS
VSLLQKTPSSPVSCHATGFYPDRATLSWRKGEEELHEDVDHGEMLLNPDGTFQMSVDLKV
SSVPPEDWSSYKCVFQLSGGKEITTTLDKNQIRTNWGKPGVRGDGEDPSNTAVIAAVAVV
VLVLVLAVVGFLLYKKKK
>CA590706.1 adult ovary Takifugu rubripes cDNA clone, TR21 Z lineage match
MELTPFFILVFGILGLGPTVNPEKHSLHYIYTATSQPISRQGIHEFTAMGVLDGNVIDYF
DSDAQKKVPRQKWMEKELDKSYWEKGTESRKSKQQWFKVNINILKTRYRQNDSDIHILQW
MHGCDADIDHGEPRFLHGIDKYSYDGANFLAFNENQEIWDARDDAARETKTRWDKVQVLR
EYTMAYLQKECVTWXKRFLDYQN
```

9. Spotted gar (*Lepisosteus oculatus)* Ensembl Genes:

```
>LO1_n.d. No Ensembl gene ID, LG14:17,405,421-17,409,911,
JH591341:1,423,681-1,425,174 unknown lineage
MRPFFIFIFFQTLTLPVTAGTFTLRYLYTGVARDVDRFSFSAVLTLNHVPILFYDSRTGN
IVPRQEWLREAFDSAFWDTERMRNREQLIVFESTMLLMMQSFNHTTAEPHTYQRLRECKL
EANGDVRVSDRFGYDGTDYLSLDQGTGTWIPAVPQAQSVKQKWDADTARTQECRVYLEEE
CIQVLKTLIRNGTGALERPVYVEVFQVEREPWEFCCLVTGISSSGAEVQWVVDQQGVLAE
GQSREELLPNGDGSFQVRQVLRVSQQERERHSYACQVNGTLQEPSFPKLRSKFGEYIIAH
HLSVLVCAGLYYLSVVKTRTQGE
>LO2_P No Ensembl gene ID, JH591468:12.330-13,136
PPHTESASASHWLVYTSTAVSEAGLLSQLLISRVLDQQTVSHWDSASWRETHTSHWPSRA
AGLRDVNYEHEEMENRERLQGILRTRSNMGKGQSALRSVLGCRLGDDGSVALFDHYGFNG
QDFLSFEVDTMTWRASSLQAEDTQRDWNQNRVKNQYIRAFLEIDCMETLKRFLEFRKIDK
NHTGETEKTPSAVL
>LO3_P No Ensembl gene ID, JH591468:29,332-29,660
WSLSASQLTPFAPPLSESASASHWLVYSSTAVSEAGLLSQLLISRVLDQQTVSHWDSASR
RETRTRHWPSGAAGLRDINYEHEETENRERLQGILRKRSNMGKGQSAFRSVL
>LO4_P No Ensembl gene ID, JH591468:44,285-45,296
ASHSLVYTSTAVSEAGLLSQLLISRVLDQQTVSHWDSTSQRETRTRHWPSGAAGLEDIDY
ELEETENRERLQGILRKRSNMGKDVLQRCRGCRLEDDGSVALFDHYGFNGKYFLSFEVDN
MIWRASSLQAKDTQRDWNQNRVKNQYTRFFLEIDCMETLKRFLEFREIDKNHT
>LO5_U No Ensembl gene ID, JH591501:9,921-24,254
MLPLLLCALVLGCREQTPAVTHSLRYFYTGVTGVRGFPEFTIVGLVDGQEFVHYDSDIKR
MIPKTEWIERNEGKDYWDRQTQILIGASQVFKTDLVNLPQRFNQSAGVHTSQSMYGCEWD
DEDGTTRGFQQEGYDGEDYLVFDLKTLTWVAPTQRAFLTKQNWDADRAFNEGKKNYLTQI
CIEWLKKYVNYGRETLNRRERPQVSVFHKDSGSGSTELTCLATGFFPRDILVSWWRDGQE
LHEDVDSGEVVPNGDGSFQVRKRLRVRAGEEHKYSCRVDHTSLEKTIVQHWEPPSLVPII
AAVCAVVALAVIAVVAVVLVRRRKSSGETS
>LO6_P No Ensembl gene ID, JH591523.1:90,771-91,013
SPFTPPHTESASASHWLVYSSTAVSEAGLLSQLLISRVLDQQTVSHWDSASWRLRWPSGA
AGLRDVDYEHEETESRERLRK
>LO7_n.d. No Ensembl gene ID, JH591545:5,102-9,058
MRRWFRVLVPALLLCGAQLVPAVVRSLYYYGCGRPGGLEQPDVTVVGRLDGEEFSYYDST
QRNVTPRQGWVRQLEDPEGWKTNTRVLEAFLQIYRTAVRERQAPGSHTVLLLCGCELEED
GGGTSRGRCALGHEGKVYLRLDTRTRTWTQVSRAPGAEQGWEPDRSFNRTVLTFLEQYCS
```

QWLKIFIKHTRKTLCRAPAPEAPGLQRGREPNSPVTCSVVSLFPGDVAVSWQRDGQELTE
GVDPAVVLPNGDGSFQVRRSLRVSEEELERHSFTCFVNGTSLGEELVIPWEPSSQNVWMA
DKNTIFVICTTAFYVLIAALVICCVTC
>LO8_U No Ensembl gene ID, JH591545:10,915-12,600
ERPLVSLSHKDSGPGGTELTCLATGFFPRDIVLSWWRDGQELQEDVESGGVVPNGDGSFQ
VRKSLRVRAGEEDKYQYSCRVDHTSLEEEIIRIWADEDR
>LO9_U No Ensembl gene ID, JH591545:58,488-73,129
GTHSYQYIYTATVGMSEFPEFVVVGMVDGEQVNYYDSSIKEMIPRREWVKGAVDPDFWNR
GTQILLGWQQTFKAGIEILRQRFNQTGGVHTWQLMYGCELDSDGTTRGYFQFGYDGADYI
SLDKSTLTWTAANQRAVITKNKWDATGADARFQTNYLENTCIEWVKKYVEYGRETLLRTE
RPQVSVYSRVSGSQGPEVTCLATGFFPKDIVVHLQRDGQDLQEDVHSGEVLPNGDGSFQV
RKSLRVSEEELKRHQYSCRVDH
>LO10_U No Ensembl gene ID, JH591750:302-10,078
MMWQLLVVSLCGVQLVSAVTHSLRYFHTGFTGVPGFPEFVAVGMVDDEQIDYYDSVTQKD
VPKQQWMKDHMDASYWERQTQKRFGWQQGSNKYALEELPKRFNQTDKRPQVSVFHKDSRS
GGTELTCLATGFFPRDILVSWLRDGQELQEDVDSGEVLPNGDGSFQVRKSLRVRAGEEDK
YSCRVDHKSLSPGDILMPWEPPSSSLVPIIAAVCAVVALAVIAVVAVVLVRRRKSSDRGE
NVLILCLIHPQEVRSPSTDRQQERNQVIPLRTPAPE
>LO11_L No Ensembl gene ID, JH591577:7,422-11,660
MEDWRSIHKRSSISHIRDISDLEYFHWRGKSDTPEFSSCEGTHSMSWSFTLTQEAQWIPK
FAVVGYLDGLPMEYYDSTHERVVSRRHWRPDPAPAENEEEKKGAAEHTYISMFEKLQLAK
KHFNHSGGLHTYQRQAGCELSDDGTQLFHARDAYDGQDLLQYNMDHLRWDPLVPELKNDE
ALMHSDDIEQNNTYQPLCIQVLKSYLEQEKDRPVMRVKPRVQVFQKTSALSGGTEVTCLA
TGFYPRALELTLLRDRRPVPEQELTGGEVLPNGDGTYQLRKSLALSKEEEERRERHRYTC
RVQHSGLDNMLEVAREPEPDLDTGLIAGVVIGVLIVALVLPVAACVLWRKKGRGACRRSD
VKYTEAQGRDQSGPSSNSSGP
>LO12_L No Ensembl gene ID, JH591577:52,184-56,541
MKPSALFLLCCGELAWADSHSLWYFMTLTTGPSQFPEFVVVGMVDDVQVEYYDSDVGKVI
SRRHWRPDAEVEEADSKVTAAKHYHNSMRNKLQLLMSHLNHTGGHRTYQRIAGCELDDDG
SARFGRWDAYDGRDALVYNTQSYSWSLLIPQVVIDKALFQVNKIRADTFYQPLCVRVLKS
YLQQERTRLMRRVKPRVRVFQKTSAFSGGTEVTCLATGFYPRALELTLLRDGRPVPEQEL
TGGEVLPNGDGTYQLRKSLALSEEEEERRGRHRYTCRVQHSGLDNGLEVDWEPEPDLDTG
LIVGVVIVVLIVVLVLPVAACVLWRKK
>LO13_L No Ensembl gene ID, AHAT01044524_1:117-1,888
DSHSLWYFLTLTTGPSQFPEFMVVGMVDDVQVEYYDSDIGKMISRRHWCPDAEVKEADNK
EFAVKDFYFGMRDKLQVLMSHLNHTGGGHRTYQVIAGCELDDDGSARFGRWDAYNGQDAM
NYNMMSYGYTVLIPGVVMDKARLQVTKFMMDTFYQPLCVRVLKSYLQQERSRLMRVKPRV
RVFQKTSALSGGTEVTCLATGF
>LO14_Z Spotted gar Z lineage sequence. Initially identified as alpha 1 and alpha 2 domains assembled from individual genomic SRA reads but later supported by individual assembled reads (97-100% identity) from a brain transcriptome (SRX543528)
GPALLLVLALCWPASGERHSLHYIYTALSKPVDGIPEFTAMGVLNDRQIDYYDNWIMQKI
PKQSWMKTNMGQEYWEKGTQSRKSKEQWFKVNVGILMDRMRQNNSDLHILQWMHGCEIEV
QPGSKPQFLRGYDQYSYDGRDFLSFDESKMQWVAPVWPAEPTKHKWDNEQILNQYTKGYL
ERECVDWLTKFLGFGEQQIKKSVPPDVHM

10. Atlantic cod (*Gadus morhua*) P and Z lineage sequences identified in this study:

>GM_PAA *Gadus morhua* Genomic contig CAEA01150108.1:156-2079
MLISIPHWIIFNICNYAYFVQSTECDPHSLEFTSMFGPESKFRPTFQQLVRFDGVPLSHC
ESWSEKALPELRPSLQDVQISLASCRQAHIDLKESHYEFPYVTNSTVDLIQRRRGCSRSE
NGALSAFETWAVNGADFVTFDPKTLQWKAMSQAAREIASRWNTRKTRNLVFRDFVNIHCP
KMIKSLELKYVDQKTDLHISAQVLPNESKFKLRCQVTSTDRSVKLLTLIGEGASYARQVT
VDGPLPFGDDYVTLRLTAVIPSSEHQYGCRVQTEKHSFSAFW
>GM_PAA EST GW844691 *Gadus morhua* cDNA, cDNA sequence.
MLISIPHWIIFNICNYAYFVQSTECDPHSLEFTSMFGPESKFRPTFQQLVRFDGVPLSHC

ESWSEKALPELRPSLQDVQISLASCRQAHIDLKESHLKFQSVTNSTVDLIQRRRGCSRSE
NGALSAFETWAVNGADFVTFDPKTLQWKAMSQAAREIASRWNTRKTRNLVFRDFVNIHCP
KMIKSLELKYVDQKTDLHISAQVLPNESKFKLRCQVTSTDRSVKLLTLIGEGASYARQVT
VDGPLPFGDDYVILRLTAVIPSSEHQYGCRVQTEKHSFSAFW
>JW206372.1 TSA:SRR499431.27147.2 *Gadus morhua* Brain tissue library SRX148752, P lineage match
MLISIPHWIIFNICNYAYFVQSTECDPHSLEFTSMFGPESKFRPTFQQLVRFDGVPLSHC
ESWSEKALPELRPSLQDVQISLASCRQAHIDLKESHLKFQSVTNSTVDLIQRRRGCSRSE
NGALSAFETWAVNGADFVTFDPKTLQWKAMSQAAREIASRWNTRKTRNLVFRDFVNIHCP
KMIKSLELKYVDQKTDLHISAQVLPNESKFKLRCQVTSTDRSVKLLTLIGEGASYARQVT
VDGPLPFGD
>GM_ZEA ENSGMOG00000000827 GeneScaffold_1860: 3-2,446 (Defined as ZEA in Ensembl, but should have been ZAA as first Z lineage gene to be identified)
SLTYIYTAFTRPVGLPGIHEFSAIGLLDGRPIDYFDSDTQVKVPREDWMKERLDKEYWDK
GTSSRQTKQQWFKVSLKVLNDRLRKNVNHYLQWRHGCEVNIQSERKLSGIDQYGYDGDDF
LSFDSHHKQWVAWTEPLTKRKWDGIQQLNDYTDGYLHNECVQWLDTFLKYGAVLENDTPP
EVYISAAIENNLKLSCLATGFHPKEITMNIRRDGHLVDRDDGLQSTGIRPNGDGTHQIKK
WVMIPRGD
>TSA_brain SRA|SRR499431.25737.2 Z lineage match
MKERLDKEYWDKGTSSRQTKQQWFKVNLKILTDRLRKNDTDNHVLQWRHGCEVNTEGSER
KLSGIDQYSYDGEDFLSFDSHHKQWVAPMDGAMLTKRKW
>GW854499 *Gadus morhua* cDNA, cDNA sequence, Z lineage match
MLSVYTLVLLCGVWTVNAEIHSLTYIYTAFTRPVGLPGIHEFSAMGLLDGRPIDYFDSDT
QVKVPREDWMKEGLDKEYWDKGTSSRQTKQQWFKVNLKILTDRLRKNDTDNHVLQWRHGC
EVNTEGPQRKLSGIDQYSYDGDDFLSFDSDHKQWVAPMNEAMPTKRKWDGIQQLNDYTDG
YLHNECVQWLDNFLKYRKEADIKASPPKVYMSARPFKNNLKLSCLATGFHPKEITMNIRR
DGHLVDRLDGLQSTGIRPYGDGTHQIKMWVMIPG
>GW857106 *Gadus morhua* cDNA, cDNA sequence, Z lineage match
MWSVSTLVLLCGVWTVDAEINSLTYIYTAFTRPVGLPGIHEFSAIGLLDGRPIDYFDSDT
PVKVPKEDWMKERLDKEYWDKGTSSRQTKQQWFKVNLKILTDRLRKNDTDNHVLQWRHGC
EVNTEGTKRKLSGIDQYSYDGDDFLSFDSDNKQWVAPMFEAMPTKRKWDGIQQLNDYTDG
YLHNECVQWLATFLKYRKEANIKASRPEVYMSARPFKNNLKLSCLATGFHPKEITMNIRR
DGHLVDRDDGLQSTGIPPNGDG
>GW848072 *Gadus morhua* cDNA, cDNA sequence, Z lineage match
MLSVYTLVLLCGVWTVNAEINSLTYIYTAFTRPVGLPGIHEFSAIGLLDGRPIDYFDSDT
QVKVPKEDWMKERLDKEYWDKGTSSRQTKQQWFKVNLKILTDRLRKNDTDNHVLQWRHGC
EVNTEGSERKLSGIDQYSYDGEDFLSFDSHHKQWVAPMDGAMLTKRKWDGIQQLNDYTDG
YLHNECVQWLDTFLXYRKEANIKASRPEVYMSARPVKNNLKLSCLATGFHPQEITMNIRR
DGHLVDRLDGLQSTGGPS
>GW862369 *Gadus morhua* cDNA, cDNA sequence, Z lineage match
MLSVYTLVLLCGVWTVNAEINSLTYIYTAFTRPVGLPGIHEFSAIGLLDGRPIDYFDSDT
QVKVPKEDWMKERQDKEYWDKGTSSRQTKQQWFKVNLKILTDRLRKNDTDNHVLQWRHGC
EVNTEVPEGKPSGIDQYSYDGDDFLSFDSVSKQWVAPMFEAMPTKRKWDGIQQLNDYTDG
YLHNECVQWLKTFLKYRKEANIKASPPKVYMSACPFKNHLKLSCLATGFPPQGDHYELQ
>EY976927 spleen SSH library enriched for genes down-regulated by formalin-killed atypical *Aeromonas salmonicida Gadus morhua* cDNA, Z lineage match
ARESISVIAAEVVPREDWMKERLDKEYWVKGTSSRQTKQQWFKVNLKILTDRLRKNDTDN
HVLQWRHGCEVNTEGPQRKLSGIDQYSYDGDDFLSFDSDNKQWVAPMNEAMPTKRKWDGI
QQLNDYTDGYLHNECVQWLDDFLKYRKEVDIKASPPKVYMSARPFKNNLKLSCLATGFHP
KEITMNIRRDGHLVDRLDGLQSTGIRPNGDGTHQIKMWVMIPGGDTAKYLP

11. Sablefish (*Anoplopoma fimbria*) MHC class I P lineage sequences:

>Sablefish_EST P lineage *Anoplopoma fimbria* GO625557 and GO625558, stop codon both 5’ and 3’ reads
GGSEACYDILESLYNIPPFVNGTVSVVQRRRGCIQADNGMVSAFEAWAVNGMDFMTFDPE
SQE*TSKSPSALTVKHRWNNNRQRNHAFRHFIIEQCPLLIQKIKLRSMHQKTELRIFAKP
EKNTARFLLRCHVTSTDTSLSSVHLIGDGASRASWITVDGPMPSEDGSVILRLTAEISQS
RNTNTYGCRVQTGGHNTTVFWDG
>JO689867.1_P lineage TSA: *Anoplopoma fimbria* cDNA sequence, orf error

GGSEACYDILESLYNIPPFVNGTVSVVQRRRGCIQADNGMVSAFEAWAVNGMDFMTFDPE
SQE*TSKSPSALTVKHRWNNNRQRNHAFRHFIIEQCPLLIQKIKLRSMHQKTELRIFAKP
EKNTARFLLRCHVTSTDTSLSSVHLIGDGASRASWITVDGPMPSEDGSVILRLTAEISQS
RNTNTYGCRVQTGGHNTTVFWDGNTLDGRYLLYQSSVLWEMGIIVLGVFCIVLIITVISC
ATICLLKCEKKKSRSPSRVDPALIEQFIKISESVASPDLQNVIVSFIRGTERDTESQDQW
DVRIMIRDLNYYDPDYFAHRVDGAPTANGRIEQVI

12. MHCI sequences from other teleost and ray-finned fishes identified in this study:

Sockeye salmon (*Oncorhynchus nerka,* unpublished data):

>Onne-UBA*0101 (*Oncorhynchus nerka*) (partial sequence) KM085986
GLLHIASAVTHSLRYFYTATTGIPDFPEFVDVGVVNGKVISYYDSITKRKVPKQSWMEEN
FNQQYWNQGTDLLKGTEQTFKANIQVAQTRFNQTGGVHIVQVMYGCTWDDDSGVIEGVQQ
HGYDGEDFLVYDMKTFTWIAPKQQAEITQRKWNNNPAGMAQRKSYITQECVEWLKKYVDF
GKNTLMRTVPPSVSLLQKTPSSPVTCHATGFYPSGVMVSWHKDGQDHHHHVEHGEILQND
DGTFQKSSHLTVTPEEWKNNKYQCVVQVTGIKEDFIKVLTESEIQTNWDDPAPNIVLIIG
VVVALLLVVFAVVGVVIWKKKSKKGFVPASTSDTDSDNCEGVKTI
>Onne-UBA*0201 (*Oncorhynchus nerka*) (partial sequence) KM085987
CVLHTTSAATHSLKYFYTTVSGDIDFPEFTIVGLVNNGQFVYYDSNIKRMVPKTEWMKQS
AGADYWDTESEKQVGTHQVFKNNIQVAKDRFNQSMSTGVHTFQLMYGCELGDDGITRGDF
QYGYDGADFLSLDKSTLTWTAANQKAVITKLKWDATGAYANNQKNYLENTCIEWLKKYVN
YGKDTLERTVRPSVSLLQKTPSSKVTCHATGFYPSGVMVFWQKDGQEQHGDVEHGEILQN
DDGTFQKSTHLTVTPEEWKNNNYQCVVQLAGIEVDITTVLIESEIQTNFGKTNRGSNDRN
PIGLIIGGVIALLVIIAVFGVFIWKKKNKKGFVPASTSDTDSENSGKGIQKI
>Onne-UBA*0310 (*Oncorhynchus nerka*) (partial sequence) KM085988
LTATTGLSDFPEFVALNLVDDELMGYFDTKTNRFEGKQSWVEEKLGQQYMEREENILRST
SQSFKVNVGIVMERFNQTKGVHTFQLMYGCELGDDGITRGDFQYGYDGADFLSLDKSTLT
WTAANQKAVITKLKWDATGAYANNQKNYLENTCIEWLKKYVNYGKDTLERTVRPSVSLLQ
KTPSSKVTCHATGFYPSGVMVFWQKDGQEQHGDVEHGEILQNDDGTFQKSTHLTVTPEEW
KNNNYQCVVQLAGIEVDITTVLIESEIQTNFGKTNRGSNDRNPIGLIIGGVIALLVIIAV
FGVFIWKKKNKKGFVPASTSDTDSENSGKGIQKI
>Onne-UBA*0401 (*Oncorhynchus nerka*) (partial sequence) KM085989
GLLHTASAATHSLKYFYTAVSGDIDYPEFTAVGLVDNGQFMYFDSSTKTAVPKTEWMKRE
GADYWDSQTQGLIGEHQTFKVNIQTLKDRFNQSKSTGVHVFQNMYGCEWDDEAGATEGFY
QFGYDGEDFVALDLKTTKWIAPTPQAVITKLKWDSNTANNEYLKNYFTQECIDWLKKYVD
YGKSTLMRTVPPSVSLLQKTPSVTCHATGFYPSDVMVSWQKDGQDHHEDVEYGETLPNDD
GTFQKSIHLTMTPEDRKNN

Channel catfish (*Ictalurus punctatus*):

>icpuJT320438_S lineage, Channel catfish TSA sequence
MRHCININKATIIHTLVLFLHLQQSRCDVQSFSLYLLRSEGLSLPRYTQSVTVNDVTLYS
FDSNMKSTGPCPEWLNTTAGQQLWKEASFLFHRNMANMDLALQTAKSQFNLTGSHADINV
YQAYSRCDLYPDGTTKSSLTHAFNGKDFLSLDMDSKTYIASVPQALIYKSIRDKDIIWLE
IVVSYYKKRCFDHLRMFLEHAPGVRNKKVPEVRLFERQGAGSTLLTCHVTGFYPRAVQVK
WIGADLQLVEDEMNHVLPNGDGTFQTRSSVIRPEENTGDQRYSCVVHHSSLEGNITVTWG
KEE
>icpuJT245151_S lineage, Channel catfish TSA sequence
MKERKTLFILLFFLHPVQLFGDDVHSISGFFISSQGLSLPNYMERITVNDVTIFYHDSGK
NIIAPCPKWLNTTTGQQHWENIRVISQHNKQMIATAIESAIQQFNLTGSHADINIYQGYS
RCDLYPNGTMKASLAHAFNGKDFLSLDFDSKTYITSVPQALKYKRLREQNSVLFGITVSF
YQKTCFDRLKMFLKHAPGVKMQKEPKVRLFERQRAGSTLLTCHVTGFYPRAVQ
>IcpuFD361597_S lineage Channel catfish 5' cDNA sequence
MRHRLNINKATIIHTLVLFLHLQQSRCDVHSISCYFLGSEGLGLPHYSESVTVNDVTLFY
YNSNMKSTGPCPKWLNTTAGQQLWKETSFLSHHNMANMDLALQTAKSQFNLTGSHVDINV
YQGYSRCDLYPDGTTKSSLTHAFNGNDFLSLDIDSKTYIASVPQALIYKRAREKNIIWLE
KLVSFYKKTRFDRLRMFLEYAPGVRNKKVPEVRLFER

>icpuFD354405_S lineage, Channel catfish 5' cDNA sequence
QSVTVNDVTLYSFDSNMKSTGPCPEWLNTTAGQQLWKEASFLFHRNMANMDLALQTAISQ
FNLTGSHADINVYQAYSRCDLYPDGTTKSSLTHAFNGKDFLSLDMDSKTYIASVPQALIY
KSIRDEDIIWLEIVVSYYKKRCFDHLRMFLEHAPGVRNKKVPEVRLFERQGAGSTLLTCH
VTGFYPRAVQVKWIGADLQLVEDEMNHVLPNGDGTFQTRSSVIRP
>icpuFD018561_S lineage, Channel catfish 5' cDNA sequence
ERITVNDVTIFYHDSGKNIIAPCPKWLNTTTGQQYWENIRVISQHNKQMIATAIESAIQQ
FNLTGSHADINIYQGYSRCDLYPNGTMKASLAHAFNGKDFLSLDFDGKTYITSVPQALKY
KRLREQNSVLFGITVSFYQKTCFDRLKMFLKHAPGVKMQK
>icpuCK423282_S lineage, Channel catfish 5' cDNA sequence
GPVRIPGSTTRPTKSSLIHAFNGKDFLSLDIDSKMFIASVPQALIYKSIREKDKIWLETV
VSFYKKTCFDDLRMFLELAPGVRNKKAPQVRLFERQGAGSTILTCHVTGFYPRAVQVKWI
GADLQLVKDEINDVLPNGDGTFQTRSSVIRPEENTGDQRYSCVVHHSSLEGNITVTWGKE
EKPFRLYVRITLGCVFIVTVVGLVIRGFFYNKGQFIVVS
>icpuFD042554.1_S lineage, Channel catfish 5' cDNA sequence
NKQMIATAIESAIQQFNLTGSHADINIYQGYSRCDLYPNGTMKASLAHAFNGKDFLSLDF
DSKTYITSVPQALKYKRLREQNSVLFGITVSFYQKTCFDRLKMFLKHAPGVKMQKEPKVR
LFERQRAGSTLLTCHVTGFYPRAVQVKWIGADLQLVDDEMNDVLPNGDGTFQTRSSVIRP
EENTGDQRYSCVVHHSSLEGNITVTWGKEEKPFRLYVWITLGCVFIVTVVGLVIRCILKS
KDAGI*

Other species:

>Rainbow trout P lineage genomic CCAF010115454.1 and TSA:GBTD01292480.1 Oncorhynchus mykiss transcribed RNA sequence
VLQRRRGCTYITNESSAGFDQWGLIGEN*LTFDPTSHTWMSESLQANPIEQSWNSNKVRSHLFKEFL
QDDLPHKLDVSLRCHVTSTDLSGLKVHLTRDRDVMTDRVRVIGPLPNVDGSVLLRLSVEIPTGLTKT
*RYRCKVQTSTSNTAA
>Ayu_S (*Plecoglossus altivelis)* S lineage TSA accession JP747954
MFLSATTCTARMISFFFVFVFTYSNAEPHSLHRHCIATQGISYHKHIQFVMIDDHIIYYY
NSSSDLGSPMPEWLDHSEGSEFWKEFTRNLKYNRHVMEKAVQLTSEHFNHSHDHVYQAQG
HCGWNTDGTRVAYMSHAYDGKDFISFDVQNKQWTAVVPEAHFYKTSRQQHSEDLDRITNH
YESECILWLKKLLQFSSKLLKPKAPDVSLFERPSSSEVLVTCHVTGFYPKEVQVEWLGEE
GHPLVQEVRRGEVLPNEDGTYQLRTILAVPLGSQHSLSYSCLVVHSSVQGNITKIWEPKH
SRFLKYWSILFICLLFLFFLLAAVGCYVRKHYYTAVDNH
>Striped Seabass (*Morone saxatilis*) P lineage TSA report GBAA01146398
MQMWIKLLIQWFLMSTAECGSHSLEFLSTGRVQPEDQPQFEQLTVFDGVPISYCNSWKKR
EELKPTLESNNLPKHCDEANNIIIDSVHVIPALINSTVYVIQRRCGCVRSADGIVSAFDA
WAVNGMDFISFDPESQRWTSQSPLAIPVRHWWKNNNGRNFAFKHFLRERCPLLIQGMKLR
STHQNTELHIFAKPIADTDQALLRCHVTSTDKSVSSVHLIGDGAFKANWISVTGPMPSED
GSVILRLTAEISLSQSTNMYGCRVQTGGHNITIFWDGNTLDGRNLLNMLTVHWKILTAIL
GFVCIIFVITAISCGTIFLLKCVKKKSRP
>Amazon molly_Za (*Poecilia formosa)*_Z lineage ENSPFOG00000004058 KI519845.1:763.515-830.994
YYKCNLHLHFSVFCLTEKHSLTYIYTAFSKPVNLPGLHEFTAMGLLDNNMIDYYDSEHQV
KVPKQDWMEKHLEPEYWEKGTQSRKSKQQWFKVNIDILMKRMRQNDNDTHILQWMHGCEG
ETQPDGSLKFVRGMDMYNYDGSDFLSFDDNHQVWVAPIHAAEETKRKWDEVQVLKEYTKG
YLEKECMEWMSKFRGFGKEKIEKASPPEVYLFTRKVNIEQNIILTCLATGFYPGEIILNI
KRNGRVLTKEDGLVTSGYRPNHDHTFQRRDHVEILKSDVSAYICEVEHLASKRHVNQTWE
NPFVEDKGNTNVIIAASASIVIVVVAVAVTLVCLKLRTSRL
>Amazon molly_Zb (*Poecilia formosa)* Z lineage ENSPFOG00000004282 KI519845.1:838.386-844.199
FVADVQVVDKMSLLAVFVLLGTGMMVNCEKHSLTYIYTAFSKPVNLPGLHEFTAMGLLDN
NMIDYYDSEHQVKVPKQDWMAKHLEPEYWDKGTQSRKSKQQWFKVNIDILMKRMRQNNTD
THILQWMHGCEGETQPDGTMKFVRGMDMYNYDGSDFLSFDDNHQVWVAPIHAAEETKRKW
DEVQVLKEYTKGYLEKECMEWMAKFRDFGKDQLLKASPPTVHLFTRKAKSETNTILTCLA
TGFLPKDITMNIKRNGRVLTLEDGLVTTGVRPNNDNTFQKRDHVEILKSDVSVYTCEVNH
RASKMHVEEKWDHIIVEDNGNMPIIIGGAVAILIVIAAIGGTLIFLKKKTNQLGFHKESS
GSQQVIFIHSILFSFEDYNN
>Amazon molly_Zc (*Poecilia formosa)*_Z lineage ENSPFOG00000004327 KI519845.1:852.537-856.320

YSSAHKAAAFAVLSFYKLFPTYLRTEKHSLTYIYTAFSKPVNLPGLHEFTAMGLLDNRMI
DYYDSENQVKVPKEDWMEKHLEPEYWEKGTQSRKSKQQWFKVNIDILMKRMRQNNNDTHI
LQWMHGCEGETQPDGSLKFVRGMDMYNYDGSDFLSFDDNHQVWVAPIHAAEETKRKWDEV
QVLKEYTKGYLEKECMEWMTKFRGFGEEQLLNAKRPEVHLFTRKAKADTNTILTCLATGF
YPKDIILQIKRNGRILTKEDGIQTSGVLPNEDDTFQRKDHVEILKSDAAAYTCEVIHPAS
HMKVEKKWDHNCNEDEQRPNIIIFVAVSMLLFVVGGVLVFLFGYKKMRC
>Eel_U lineage_VII, GAGT01004863.1 Japanese eel (*Anguilla japonica)* transcribed TSA contig04994, alpha 1 lineage VII match
MSGLVLLSIVFCGLRGAFAVMHSWKAFYTGSTGLSESPEFVAVNIVDDEPVGYFDSRTNS
FQYRQKWMEEHLGPEYLKQQTDILKGAIPRFKANVGIAMKRFNQSGGVHTFQNMYGCEWD
DETGATGGFDQYGYDGEDLVNYEMKNQRFIAPVQQAFFSVQKWNNDPAGLEYEMQYLTLE
CVAWL
>Eel_U_lineage VIII, GAGT01017750.1 Japanese eel (*Anguilla japonica)* transcribed TSA contig20909, alpha 1 lineage VIII match
IHGASAASHSLKYFYTGVTAGIDFPEFTAVGLVDDEEFSYFDSNIPKIIPKTEWFEKAVD
EQYWHRNTQNFIGAHQTFKAGVGILMQRFNQTQGVHTYQNVYGCEWDDETGATGGFLQYG
YDGEDLVNYEMKNQRFIAPAQQGFIT
>Eel_Z lineage, Japanese eel (*Anguilla japonica*) gill transcriptome TSA Accession SRX247092. Assembly of individual SRA reads.
MKLKIILALFCVFGTVQGEIHNLHYIYTALSKPITQPGVYQFTALGILNDRPIDVYNSKD
KVKKPLQKWMEEHNEKDYWEKGTQSRRSKEQWFDVNVKILMERMRQNQSDPDVHILQWMH
GCQAEKTPDGKLKFHNGFDQYGYDGKDFLNFDSENMAWVAPVPAAESTKTKWDNVPILSQ
YTKGYLQTDCVHWLEKFLKYQELDFQDSSPPDVYIFSKKSTSPDYRTLVCLATGFYPKDV
AVDILKDGAPLAESDGVESSGVRTNGEPKETFQLRKWLEIKTSDTSKYSCRVKHQTLRSP
IVKFWDGKCHNCDGDNTGAIVGAVVGVLLIVLVAVLVGLYFLRKKMNEKEKAAQAAANGL
IQPLTGVSTEGNGSNSSVQSGDSGRGSTDSGEPKEVTKPFIVNGAIA
>Sturgeon Z lineage alpha 1 fragment, assembly of SRA genomic reads, mostly from *Acipenser persicus*, a little bit from nearly identical *Acipenser baerii*
CREGFPEFTAMGLLDDMQIDYYDSVNQKKESKQTWMKENMAPDYWEKGTKSRKSKEQWFK
VNVRILEKRF
>Sturgeon Z lineage alpha 2 domain fragment, assembly of SRA genomic reads, mostly from *Acipenser persicus*, a little bit from nearly identical *Acipenser gueldenstaedtii*
CRLDEDNNEEVQGYDQHSYDGNDFLSFDKNHLQWVAPMQPAVATKLKWDGEKILNQYTKG
YLENECINWLKMFLGYGERDIYRS
>Lungfish (*Protopterus annectens*) classical MHCI, assembled individual SRA reads from SRX152529
MNFLPAFLVVAAVFTGFSQCRSHSLRYFYTGVSGISEFPEFVLLGYVDDQTIGKFESTDQ
VYKPQVQWMEENEDQGYWTRETQNIQSWQQVFKANVKTLMDRTNQTTGVHTNQVMYGCSL
NDDGTTTGEEQHAFDGKDFISFDKDSLTWNAAALYGVITVNRWNSDANFNMQTKGYLEQT
CIEWLKKYTQYGQSSLEQKVKPVVKVTDKTAGDRTKLKCTVTGFHPRDIDVHFYKDDKDQ
MQATEFAGVLPNSDGTYYTETWIEIAPQDKDRYSCHVEHASLTPTLIVKPDPESNNVLII
VIAVVGGVVFWKRRRTGGQKYAAANKTEHGESSSNSSANA

Relevant previously published sequences:

13. Previously published zebrafish (*Danio rerio*) sequences:

Sequences denoted dz are from McConnell et al.2013 (main text reference 23). Dijkstra et al.2007 is main text reference 26.

.

>XM_001340377.4, Chr3:24,523,355 Dijkstra et al.2007, DR10 match
MMYLVLAVCILSASVFSQSGSHSLWVFATFLTGDISVQFPEFSAVVMLDDIVIGHYNADD
RSFVPSTVQESVNITEQITISTVCKGIHEGMKTKAYYLIDYLNHTRGLHVQQRLVGCELL
QNEPGQMMTLEAFNGESGFERRYDIQGDQQTHWKWPVIKSRAQLEYDAWLYAHFYRPLCI
NQLRKYLKKEKKRVMARVKPRVRVIQRTCSKTGKIQMTCLATGFYPRHINLTLLQDGQPV
NEERVMGGELLPNADGTYQMRKSVELSAEEQRERRTYTCTVNHLSLDNKLDISIAEPGLD
PVIIFPSVLLLLCVFGVLAGFLMWRKKY

>XM_691881 Dijkstra et al.2007, DR11 match
MLFLLYLLSCLTFTDAGFHSLLVLATYVDGQTPFPELSVVVMLDDVQIIYYDSDTWRVFH
RSPSDSKYYDEDQSDADAVFHDTYDEMKYRVLHLKNQLNHTDGITVLQRIVGCELFNDKP
GIYHLWDAHDGKTIEKFTFNIYNHEFQLKNQWFRTWDQVMIQQKRIVHENIYYPVCIKVL
RRYLNVEKNSVMRKVKPRVRLMKKKLPDSQGLQISCLATGFYPRHINLTLFRDAEPVDDD
QIIGGEILPNGDGMYQMRKSLIVSKEELDEGHEYTCTMKHLNLDNKLDIVFDVSGTVPGC
FSVSVVISVLVFMCVSVFIITKLIMRRKRQDTE
>NM_001017904 Dijkstra et al.2007, DR12 match
MALATYIVGQTPFPEFSVVVMLDDLQLAYYDSIGWKTIYRSGSDSKYYDEEQSDAGIVFR
DMFYDMKDRAFYLKDHQNHTDGVHVHQRLVGCELLNNEKTGPLHYWDAFGGQNMEEFIFD
TEKHTIQVKMPWVITWDQLKRLHENFMYDNVYHPICIKTLRRYLNMEKKNVMRKVKPRVR
LMQKKLSNSQGLQISCLATGFYPRHINLTLFRDAVLVDDDQITGGEILPNGDGTYQMRKS
LIVSEEELQKGHKYNCTANYLNLDNKMDIVFDVAESDPGFFSVSVVMGVLVFVGLSVLSI
TALIMRRKRRDTGSVSGTSQNQYVYAQTSVQDAT
>NW_645013 Dijkstra et al.2007, DR24 match
SHSLWVVATYIKGQTPFPEFSYVLMLDDITVMYYNSDTKTFFPRGNTTAEDDLYGVFAVQ
QLVVLDLRADSEPGQIISQNTFEGCITDEVRYVDKKLTYQGTLNVSAPVHHIHHEYVKYL
CETLIHPFYFKTLKGYLIKRRNQINRKVKPKVRLILKANSDSGGFRVSCLATGFYPRHIN
LTLLRDGQPVSDHGVTGGDLLPNGDGTYQMRKSLEIRGEEREKHKYTCSATHLSLDKKLD
ITLGN
>CAD56801.1 novel protein similar to MHC class I heavy chain [Danio rerio], Dijkstra et al.2007
SHSLMVFGTYIAGQTQFPEFSAVVMLDDLQVLYYDSITQKPVPHSQSNSEYKDTNYTLIF
RDMHDLLKYRTFYVKDNWNLTDGLHVYQKHAGCELLDNDEPGLFLSWDAFNGKNTEEFTF
DVATRTVQNTLPWMRIKNLSHLEWLQVTFLYKNVYHPICIKTLRRFLGKEKKIVMRKVKP
RVRLIKKRLPDCQGLQISCLATGFYPRHINLTLFRDTEPVDDDQITGGEILPNGDGTYQM
RKTLIISKEELHERHQYNCTMKHLDLDNKLDLKFGNEKQAIMKTKHILILLGFIALYSDE
>dzUDA NP_571779.1 major histocompatibility complex class I UDA precursor [Danio rerio], McConnell et al.2013
MQQIVLLLIGAHLSYAGIHSLKYFFTGVSGDIDFPEFTAIGIVDGEQFYYYDSNKMKSVP
KTEWIRQNEGADYWDRQTQLAIGYHQVYKVDFQTLKERFNQSQGIHTIQEMYGCEWDDET
RATNGFYQDSYDGEDFVYLDLKEMRYISPVPQALLTLQKWNDDKAFLAQQINYLSIECIE
WLQKYMQYGKSSLEKTVSPQVSLLQKSSSSPVVCHATGFYPSGIKISWQKNGQDHDEDVE
LEELLPNEDGTFQKSSTLTVTPEEWKNNKFRCVVEHQGKTKTEKEIRTNEVSVPFGIIIA
VIAVVLLIAIGITGYMVWKKKQGFKPVSGSDDGSNSSTHAVPQA
>dzUEA NP_571780.1 major histocompatibility complex class I UEA precursor [Danio rerio] McConnell et al.2013
MQQVLLMLLGAHLAYAGTHSLRYFYTGVSGDIDFPEFTVVSLVDGEQFVYFDSNTMKTVP
KTEWMRQNVGEDYWERETQIFTGAHPVFKNNIQVIKERFNQTQGVHTFQQMYGCEWDDQT
EAKNGFDQIGYDGEDFLSLDLKEIRWISPVQQGLITTQNWNNNRGFLQSYGGYYSTVCIE
WLQKYIQYGKSSLKKTVSPQVSLLQKSSSSPVVCHATGFNPSGIKISWQKNGQDHDEDVE
LGELLPNADGTFQKTSTLNVKPEEWKNNKFRCVVEHQGETRAVDDPILTNEDSLPIGLIV
GVVAALVLLVVIAVAGYMVYQKKKGFKPVSGSDDGSNSSAHAVPQA
>dzUFA CAD58763.1 major histocompatibility complex class I UFA gene [Danio rerio] McConnell et al.2013
MRQLVLLLLGVHLAYAGKHSHTVIYTATKGLPDFPEFVAATMVDGMQVNYFDSEIKEVIP
RQEWVRGAVDEQFWQRNTQIRSNMHQLFKNNINIAMERFNQTQGVHTFQFMVGCELEDDG
TTRGHWQYGYDGEDFISFDKNTLTNVVANPQAVITKNKWDANKAQNEYRKQYLENQCIEW
LRKYVGYGKDSLGRKDAPEVFMLQKDPSSPIVCQATGFYPSNIMMTWQKNKEEHFEDVDV
GATLTNADGTFQKTVTLSVKPEEWKNNKEAYRCVVQHVGAKDDVIVTVKDIRSNGGSDNT
IAIVVGCVAAVAVLAVIAGLIYWKKSNGYGRASSKDTDSEQSDPPTVQVK
>dzUGA NP_956879.1 major histocompatibility complex class I UXA2 precursor [Danio rerio] McConnell et al.2013
MQQVLLFLLGAHLAYAGTHSLRYFYTAVSGDIDFPEFTMVGLVDGGQFIYFDSKKMEAVP
KTEWIRQNEGADYWDINTQRLIATHQAFKNNIQVAKERFNQSQGVHTFQVMYGCELEDDG
STRGYWQYGYDGEDFLSLDKSTLTWTATKPQAVITKNKWDADNADRQYTKSYLENECIEW
VKKYVDYGKDTLERKDAPEVFMLQKDPSSPVVCQATGFYPSNIMMTWQKNKEEHFEDVDV
GATLTNADGTFQKTVTLSVKPEEWKNNKEAYRCVVQHVGAKNDVIVTVKDIRSNEGSDNT
IAIIVGCVAAVALLAVIAGLIYWRRSNGYGKASSKDTDSEQSDPRAVEVK
>dzUHA NP_001070109.1 uncharacterized protein LOC767703 precursor [Danio rerio] McConnell et al.2013

MQSLTGLLLVVCLQYASGATHSWKAYYTATTGLTEFPEFVALNVIDDQLMGYFDSKTNRF
KSQFQWMEDNLGKEYDEQETNILQGHTASFKNNVKVAMERFNQTQGVHTNQRMYGCEWDD
ETGAINGFFQDGYDGEDFLSLDLKELRWISPVQQGLMTTQKLNNDRANTEYYKNYFTTEC
IEWLQKYLQYGKSSLQKTVSPHVSLLQKSSPVVCHATGFYPSGIKISWQKNGQDHDEDVD
LGELLPNEDGTFQKASTLKVNSDELKNNKFTCVVEHQGEIETRAIEDSVSVGLIAGVAVA
VLLVIVIAVAGFMMYRKKKGYKPANSSDDGSRSSAQDGPRA
>dzUIA AGL92229.1 MHC class I antigen UIA [Danio rerio] McConnell et al.2013
MQTIILLLLGAHLANAVTHSMKYIITGVSELDSFPEYTSVGTVDDIPFVYFDSNIKRMIP
KTEWIKNEGADYWDRESQRESGIVPVFKNNIQIAMQRFNQTAGVHTVQNMYGCEWDDQTG
AKNGFDQYGYDGEDWLTLELKELRWISPVQQAMITTQNWNKNRAQLEYYRNYYSTECIEW
LQKYLQYGKSSLQKTASPQVSLLQKSSSSPVVCHATGFYPSAIKISWQKNGQDHDEDVDL
GELLPNADGTFQRTSTLNVKPEEWKNNKFSCVVEHQGETRAADDKILINNDSAPIGLIAG
VVAALVLLVVIAVAGYMVYQKKKGFKPVSGSDDGSNSSAHAVPQA
>dzUJA NP_956700.1 uncharacterized protein LOC393377 precursor [Danio rerio] McConnell et al.2013
MRAILIFLIGLNLTNAVIHSHEYIYTATSGIEGFPEFVIVGMVDGQQINYFDTHSMKMVP
RTDWVSGAVDPDFWNRNGQIASGTHLNFKNNIEVAKQRFNQTGGVHTVQAMYGCEWDDQT
GAKSAFKAFGYDGEDFLSVDFKELRYISPVQQGIPTVQKWNNDRGMIESDRNYYGTVCIE
WLQKYLQYGKSSLEKTVSPQVLLLQKSSSSPVVCHATGFYPRGIIISWQKNGQDYDEDVE
LGELLPNADGTFQKTSTLNVKPEEWKNNKFSCVVEHQGKTRAADETILTNEESAPIALIA
GVVAALLLLVVIVVAGYMVYQKKKGFKPVSGSDDGSNSSAHAVPQA
>dzUKA NP_001038925.1 uncharacterized protein LOC751750 precursor [Danio rerio] McConnell et al.2013
MEYILLLVVCLPYVDGATHTWNAYYTATTGLSQFPEFVALNLIDGQLMGYFDSKTNRFKS
QFQWMEDKLGTAYDEQQTNILQRHTATFKNNIKVAIERLNQTQGVHTFQEMYGCEWDDQT
GNINAFRQYGYNGEDFLILDFKVWRWITPVQQAMITTQKWNNDKGFIESDRNYFRSECIE
WLKKYLMYGKSSLEKTVSPQVFVLQKSSSSPVVCHATGFYPSGIKISWQKNGQDHDEDVE
LSELLPNADGTFQKASILTVTPEEWEKNKFNCVVEHQGVTINSILTEVEIRTNNSSAPIG
IIIGIVYAVVLLLFIAVAWFVVYRKKKGIKHVAANAVNSDKNSGCGSGSDSVSHKS
>ZE*0101 AJ420953.1 Danio rerio cDNA for MHC class I antigen (dare-ZE*0101 gene), Stet et al.2002
MAVFAVLFSAVMLLAIVPAWTEKHSLYYIYTALSRPVNLPGIHEFTAMGLLDDRQIDYYN
SQEQKKIPKQPWMKEKMQEDYWEKGTQSRKSKEQWFNVNVNILMERMRHNKSDVHVLQWR
HGCEIESQDNNVRFSKGIDEYSYDGENFLSFDDADSQWVAPVDAALPTKRKWDNVPILNQ
YTKGYLEKECVDWLNKFREYGDEELKQGSAPKVHVFAKRYVNGKAKLKLTCLATGFYPKD
VYLTIRKYRTALSDSEVESSGVRPNHDGTFQLRKSTYILEEEKAEYDCYVAHRTLNAPVV
TTWDGKCSDCSKESAIGLIVGAIIGAVVVAAIVVVAFILKKNNKFCFRTTQEPSEENGRV
LMKDPVFKEENGAGKDPSVVPLTNGNH
>ZE*0401 AJ420956.1 Danio rerio partial cDNA for MHC class I antigen (dare-ZE*0401 gene), Stet et al.2002
MTSFDRSFTLLYLCLFHVILSSFRAEKHSLYFIYTGLSRPLDLPDIYEFSAMGLLDDRQI
DSYNSEEQRNIPKQQWMKEKMQEDYWETRTQSRKEKQLWFYDNVHLLIDRNRQSTSVLHV
LQWRHGCEVEKRGNEHSFNKSIDEYGYDGEDFLYYDDAESRWVAPVEEALPTKRKWDNLP
ILNQYTKDYLEKECV
>dyZAA GenBank KC607829, Dirscherl & Yoder 2013
MAVFAVLFSAVMLLAIVPAWTEKHSLYYIYTALSRPVNLPGIHEFTAMGLLDDRQIDYYN
SQEQKKIPKQPWMKEKMQEDYWEKGTQSRKSKEQWFNVNVNILMERMRHNKSDVHVLQWR
HGCEIESDNNVRFSKGIDEYSYDGENFLSFDDADSQWVAPVDAALPTKRKWDNVPILNQY
TKGYLEKECVDWLNKFREYGDEELKQGSAPKVHVFAKRYVNGKAKLKLTCLATGFYPKDV
YLTIRKYRTALSDSVESSGVRPNHDGTFQLRKSTYILEEEKAEYDCYVAHRTLNAPVVTT
WDGKCSDCSKESAIGLIVGAIIGAVVVAAIVVVAFILKKNNKFCFRTTQEPSEENGRVLM
KDPVFKEENGAGKDPSVVPLTGNH
>dyZLA GenBank KC607872, Dirscherl & Yoder 2013
MAVLAVLFSAVMLLSVVPAWTDHVLLSFNTEKHSLFYIYTALSRPVDLPGIYEFTAMGLL
DDRQIDYYNSIDQKKIPKQPWMKEKMQEDYWEKGTQSRKSKEQWFNVNVNILMDRMRHNK
SDVHVLQRHGCEIDSQGNDVRFSKGIDEYSYDGENFLAFDDAESRWVAPVEEALPTKRKW
DNVPILNQYTKGYLEKECVDWLKKFREYGDQELRKVSPRDVHVFAKRYINGKDKLKLTCL
ATGFYPKDVYLTIRYRTALSDNDLESSGVRPNHDGTFQLRKSTYINEDEKAEYDCYVNHR
TLKEPVIIRWNGECLSEPPIAMIAGIIGVLILLGAIGVTVWILKKKNIIGNGDEKHFVVS
TVSGNKDENGFVVSTVSGNKDKRSMPDGSADSGKGSSA

14. Selected Atlantic cod (*Gadus morhua*) MHCI sequences from two U lineage clades:

10 sequences were selected from each of the two cod U lineages distinguished by Malmstrøm et al.2013, main text reference 20.

```
>GM1_AGV52778.1 MHC class I antigen, partial [Gadus morhua]
VLHSLHYFYTGSSGLSTFPEFVAVGMVDGVQIDYYDSNIQRAVLKQDWMEQVIREDRDYL
ERNAGILQGAQQTYKAGVGILKQRFNQTGGTHLYQRMYGCEWDGEDDSTDGYNQYGYDGE
DFIAFDPKTLTWVAPVRQAVPTKQKWDGLRAYNEYWKNYQTKECVDWLKKYLAYGKSTLQ
RTERPRVSLLQRSPSSPVVCHATGFYPDRVVVFWRRDGQELHEQVDPGEVLPNHDGTFQV
SVDFNLKAVPQEDWGRYECVVQLKGIEDISTRLDPALIRTNGGKSGLTIPIIIGLLVLLL
VAAAAVVVGVLLYKKRNASDKQQKPGGSDTSSEGTEGQNPPPESQPLTT
>GM10_AGV52769.1 MHC class I antigen, partial [Gadus morhua]
VLHSMRIFYTASSGLTAFPEFVAVGMVDGVQFNYYDSNTQRTVPKQDWMELVIREDRDYL
ERETGKRKGAQQVFKANIGIAKQRFNQTGGAHMFQNMYGCEWDDEDDSTDGYNQYGYDGE
DFISLDLEHLTWVAPVQQACTTKRKLDENKAQLQYFKNYYTKECVDWLKKYLVYGKSTLQ
RTDRPRVSLLQRSPSSPVVCHATGFYPDRVVVFWTRDGQELHEQVDPGEVLPNHDGTFQV
SVDLNLKAVPQEDWGRYECVVQLKGIEDISTPLDPALIRTNSGKSGLTIPIIIGLLVLLL
AAAAAVVGVLLYKKRNDKRHKPVGSDTSSENTEGQNPAPEAQPLTK
>GM11_AGV52768.1 MHC class I antigen, partial [Gadus morhua]
VLHSLHYFYTGSSGLTAFPEFVAVGMVDGVQMLHYDSVSKRAVAKQDWMERYTRDHPEVL
ERDNGNLQGAQQTFKAGVGILKQRFNQTGGAHMVQNMYGCEWDDEDGAIDGYDQYGYDGE
DFLAWNMKTMTWVAPVPQAFSTKQRWNQDRADLQYNKNYQTKECVDWLKKYLAYGKSTLQ
RTERPRVSLLQRSPSSPVVCHATGFYPDRVVVFWRRDGQELHEQVDPGEVLPNHDGTFQV
SVDLDLKAVPQEDWGRYECVVQLRGIEDISTPLDPALIRTNEGKSGLTIPIIIGVLVLLL
AAAAIVGVILYKKRNASDKGHKPVASSDTSSENTEGQNPAPEAQPLTT
>GM12_AGV52767.1 truncated MHC class I antigen, partial [Gadus morhua]
VLHSLHYFYTASSGLTTFPEFVVVGMVDGVQMVHYDSVSKRAVAKQDWMEQVTKEDRDYL
DRETGKFQGSQQSFKVSIGTAKQRFNQTGGAHLYQNMYGCEWDDEDGTTDGYHQFGFDGE
DFIAFDLKTLTWVAPVQQAFPTKRRWDENRADNQYIRNYFTKECVDWLKKYLTYGKSTLQ
RTERPRVSLLQRRPSSPVVCHATGFYPDRVVVFWRRDGQELHEQVDLGEVLPNHDGTFQV
SVDLNLTAVPQEDWGRYECVVQLKSIEDISTPLDPALIRTNGGKSGLTIPIIIGLLVLLL
AAATIVGVLLYKKRNASDQRHKPVATSDTSSEDA
>GM24_AGV52755.1 MHC class I antigen, partial [Gadus morhua]
VLHSLHYFYTASSGLSTFPEFVAVGMVDGVQIDYYDSITQRTVLKQDWMKQATSGDGDYL
ERETGKAQGAQQTFKANIGTAKQRFNQTGGAHIVQKMYGCEWDDEDGSTDGYRQFGYDGE
DFIAWDMKTMTWVAPVRQAVITKQRWNEERAQLQYLKNYITEDCVDWLKKYLANGKSTLQ
RTERPRVSLLQRSPSSPVVCHATGFYPDRVVVFWRRDGQELHEQVDPGEVLPNHDGTFQV
SVDLNLKAVPQEDWGRYECVVQLKGIEDISTPLDPALIRCNSGKSGLTIPIIIGLLVLLL
AAAAAAVVGVLLYKKRNASDKRHKPVGSDTSSENTEGQNPAPEAQPLTK
>GM26_AGV52753.1 MHC class I antigen, partial [Gadus morhua]
VLHSLHYFYTASSGLSAFPEFVAVGMVDGVQMVHYDSNTQRTVLKQDWMEQVTSGDGDYL
VRNTGKFQGAQQVFKANIGIAKQRFNQTGGAHMVQWMYGCEWDDEDNSTDGYNQYGYDGE
DFISLDLKTLTWVAPVRQAFSTKQRWDGLRAQTVRYKYYYTKECVDWLKKYLAYGKSTLQ
RTERPRVSLLQRSPSSPVVCHATGFYPDRVVVFWRRDGQELHEQVDPGEVLPNHDGTFQV
SVDLNLKAVPQEDWGRYECVVQLKGIEDISTPLDPALIRTNWEDKSGLTIPIIIGLLVLL
LAAAAAVVGVLLYKKRNASDQRHKPVGSDTSSENTEGQNPAPEAQPLTT
>GM35_AGV52744.1 MHC class I antigen, partial [Gadus morhua]
VIHSLQVFHTASSGLSTFPGYVMVVMVDEVQVEYYDSNTQRIITKQDWIDQFYRDPPGEL
EIATERRKGNQQTFKANIGTAKQRFNQTGGAHIVQKMYGCEWDDEDGSTDGYRQFGYDGE
DFIAWDMKTMTWVAPVRQTVITKQRWNEERAQLQYLKNYITEDCVDWLKKYLANGKSTLQ
RTERPRVSLLQRSPSSPVVCHATGFYPDRVVVFWRRDGQELHEQVDPGEVLPNHDGTFQV
SVDLNLKAVPQEDWGRYECVVQLKGIEDISTPLDPALIRCNSGKSGLTIPIIIGLLVLLL
AAAAAAVVGVLLYKKRNASDKRHKPVGSDTSSENTEGQNPAPEAQPLTK
>GM41_AGV52738.1 MHC class I antigen, partial [Gadus morhua]
VLHSLHYFYTASSGLKAFPEFVAVGMVDGVQMLHFHSNTQRVVLKQDWMEQLTREYPDYL
ERSTGLSLGSQQSFKYNIGILMKRFNRTGGAHVYQEMYGCEWDDEDGSTDGYDQFGYDGE
```

DFLSLDLKSLTWVAPVPQAFSTKLRWDQKRGYNEQQKNYYTKECVHWLKKYLVYGKSALQ
RTERPRVSLLQRSPSSPVVCHATGFYPDRVVVFWRRHGQELHEQVDPGEVLPNHDGTFQV
SVDLNLTAVPQEDWGRYECVVQLKGIEDISTPLDPALIRTNWEDKSGLTIPIIIGLLVLL
LAAAAAVVGVLLYKKRNASDQRHKPVGSDTSSENTEGQNPAPEAQPLTT
>GM49_AGV52730.1 truncated MHC class I antigen, partial [*Gadus morhua*]
VLHSLHYFYTGSSGLSTFPEFVAVGMVDGVQIDYYDSITQKKVLKQEWMKRYTIREDPDY
LERLTGILQGHQQTFKASIVNVKQRFNQTGGTHLYQFMCGCEWDDEDDSTDGYNQYGYDG
EDFIAFDLKTLTWVAPVRQAVPTKQKWDGLRAYNEYWKNYQTKECVEWLKKYLSYGRSTL
QRTERPRVSLLQRSPSSPVVCHATGFYPDRVVVFWRRDGQELHEQVDPGEVLPNHDGTFQ
VSVDLNLTAVPQDWRRYECVVQLRGIEDISTSLDPALIRTNSGKSGLTIPIIIGLLVLLL
AAAAIVGVLLYKKRNASDQRHKPVATSDTSSEDA
>GM54_AGV52725.1 MHC class I antigen, partial [*Gadus morhua*]
VIHSQHFFFTASSGLSTFPEYVDVQMVDEVQIGYYDSNTQRSIPKQDWADQANRDKDPDY
LEKDTENRKGLQQAFKANMGILKQRFNQTGGAHIIQRMDGCEWDDEDGTTEGYDQHGYDG
EDFISLDLKTLTWVAPVRQAFSTKQRWDGLRAQTVRYKYYYTKECVDWLKKYLVYGKSTL
QRTDRPRVSLLQRSPSSPVVCHATGFYPDRVVVFWTRDGQELHEQVDPGEVLPNHDGTFQ
VSVDLNLKAVPQEDWGRYECVVQLKGIEDISTPLDPALIRTNSGKSGLTIPIIIGLLVLL
LAAAAAVVVGVLLYKKRNASDKRHKPVGSDTSSENTEGQNPAPEAQPLTK
>GM58_AGV52721.1 truncated MHC class I antigen, partial [*Gadus morhua*]
VIHSLQFFYTASSGLPTFPEYVMVVMVDEVQVEYYDSNTQRIITKQDWVDQANEDKVPDY
LERETERRKGNQQVLKGNIGTLKKRFNQTGGAHVYQQMYGCEWDDEDGTTEGYEQHGYDG
EDFLSLDLKTLTWVAPVHQAFPSKLRLDHNTAYNQYVKNYYTKECVDWVKKLLGYGKSTL
QRTERPQVSLLQRSPSSPVVCHATGFYPNRVVVFWRRDGQELHEQVDPGEVLPNHDGTFQ
VSVDLDLKAVPQEDWGRYECVVQLKGIEDISTPLDPALIRTNGGKSGLTIPIIIGLLVLL
LAAATIVGVLLYKKRNASDQRHKPVATSDTSSEDA
>GM62_AGV52717.1 MHC class I antigen, partial [*Gadus morhua*]
VLHSLHYFYTASSGLTTFPEFVAVGMVDGVQINYYDSNTQRVVLKQDWMERFASGDADYL
ERSTGIIQGTQQVFKANIGIAKQRFNQTGGAHMFQNMYGCEWDDEDDSTDGYHQYGYDGE
DFISLDLKTLTWVAPVQQACTTKRKLDENKAQLQYFKNYYTKECVDWLKKYLVYGKSTLQ
RTDRPRVSLLQRSPSSPVVCHATGFYPDRVVVFWTRDGQELHEQVDPGEVLPNHDGTFQV
SVDLNLKAVPQEDWGRYECVVQLKGIEDISTPLDPALIRTNSGKSGLTIPIIIGLLVLLL
AAAAAVVGVLLYKKRNASDKRHKPVGSDTSSENTEGQNPAPEAQPLTK
>GM83_AGV52696.1 MHC class I antigen, partial [*Gadus morhua*]
VIHSLQFFSTASSGLSTFPEFVMVEMVDEVQVEYYDSNTQRSIPKQDWVEQANRDKVPDY
LERETENRKGIQQGFKAGMGILKQRFNQTGGAHIIQKMNGCEWDDEDGSTDGYRQFGYDG
EDFIAWDMKTMTWVAPVRQAVITKQRWNEERAQLQYLKNYITEDCVDWLKKYLANGKSTL
QRTERPRVSLLQRSPSSPVVCHATGFYPDRVVVFWRRDGQELHEQVDPGEVLPNHDGTFQ
VSVDLNLKAVPQEDWGRYECVVQLKGIEDISTPLDPALIRCNSGKSGLTIPIIIGLLVLL
LAAAAAAVVGVLLYKKRNASDKRHKPVGSDTSSENTEGQNPAPEAQPLTK
>GM90_AGV52689.1 MHC class I antigen, partial [*Gadus morhua*]
VIHSLHYFYTASSGLTAFPEFVAVGMVDGVQMLHFDSFSKKTVLKQDWMEQATRGYPSYL
ERSNRLSLGSQQAFKADIGIAKQRFNQTGGAHIYQEMYGCEWDDEDDSTDGYDQYGYDGE
DFIAFDLKTLTWVAPVRQAYPTKQRWDGLRGYDQQQKNYLTKECVYWLKNYLVYGKSTLQ
RTERPRVSLLQKSPSSPVVCHATGFYPDRVVVFWRRDGQELHEQVDPGEVLPNHDGTFQV
SVDLNLKAVPQEDWGRYECVVQLKGIEDISTPLGRALIRTNSGKSGLTIPIIIGLLVLLL
AAAAAVVGVLLYKKRNASDKGHKLVGSSDTSSEKTEGKNPAPEAQPL
>GM93_AGV52686.1 MHC class I antigen, partial [*Gadus morhua*]
VIHSLQSFDTASSGLPTFPEYVMVLMVDEVQVEYYDSNTQRIITKQDWVDQANRDKDPDY
LERETERRKGNQQVFKGNIGTLKKRFNQTGGAHIVQRMYGCEWDDEDGTTEGYEQYGYDG
EDFISLDLKTLTWVAPVHQAFPSKLRLDHDTAQNQYKKNYFNKECVDWLKVYLVYGKSTL
QRTERPRVSLLRRSPSSPVVCHATGFYPDRVVVFWRRDGQELHEQVDPGEVLPNHDGTFQ
VSVDLYLTAVPQEDWGRYECVVQLKGIEDISTPLDPAHIMTNREGSHILAFILTGVAVLA
VVVAAVVGVFLYKKRNDSDKRHKPVGSDTSSENTEGQNPSPEAQPLTT
>GM98_AGV52681.1 MHC class I antigen, partial [*Gadus morhua*]
VIHSLHFFDTASSGLSTFPEYVGVVMVDEVQVEYYDSNTQRIIPKQDWADQANREESDSL
ERDTEIRKGNQQAFKAGMGILKRRFNQTGGAHIIQQMYGCEWDDEDGTTEGYNQLGYDGE
DFLSLDLKTLTWVAPVHQAFPSKLRLDHNTAYNQYVKNYYTKECVDWVKKLLGYGKSTLQ
RTERPQVSLLQRSPSSPVVCHATGFYPNRVVVFWRRDGQELHEQVDPGEVLPNHDGTFQV
SVDLYLTAVPQEDWGRYECVVQLKGIEDISTTLDPAHIRTNGGDNHILAFILTGVAVVAA
FAVVVGVFLCRKRNDSEKCHKPVDSDTSSENTEGQKPSPEAQPLTT
>GM100_AGV52657.1 MHC class I antigen, partial [*Gadus morhua*]

VIHSLQFFYTGSSGLSTFPEYVMVLMVDEVQVEYYDSNTQRIITKQDWADQATRDKDPDY
LERETENRKGNQQVEKGNIGTLKKRFNQTGGAHILQRMYGCEWDDEDGTTEGYQQYGYDG
EDFLSLDLKTLIWVAPVHQAFATKLRWDHDTAYNQYLKNYYTKECVDWLKKLLAYGKSTL
QRTERPRVSLLQRSPSSPVVCHATGFYPNRVVVFWRRDGQELHEQVDPGEVLPNHDGTFQ
VSVDLDLTAVPQEDWGRYECVVQLKGIEDISTTLDPAHIRTNGGDNHILAFILTGVAVVA
VVAAVAVVGVFLYRKKNDSEKCHKPVDSDTSSENTEGQKLAPEYQPLPT
>GM104_AGV52653.1 MHC class I antigen, partial [*Gadus morhua*]
VIHSLQLFYTASSGLSTFPEFVTVQMMDEVQVEYYDSNTQRIIPKQDWVDQANRDKVPDY
LERATERRKGIQQVFKADMGTLKRRFNQTGGAHIIQTMSGCEWDDEDGTTEGYDQYGYDG
EDFISLDLKTLTWVAPVHQALTTKHKWEQNRAYMEQQKHYYTKVCVDWLKKYLAYGKSTL
QKTERPRVSLLQRSPSSPVVCHATGFYPNRVVVFWRRDGQELHEQVDPGEVLPNHDGTFQ
VSVDLDLTAVPQEDWGRYECVVQLKGIEDISTPLDPAHIRTNREGSHILAFILTGVAVLA
VVVAAVVGVVLYQKRNDSDKRHKPVDSDTSSENTEGQKLAPEYQPLST
>GM120_AGV52672.1 MHC class I antigen, partial [*Gadus morhua*]
VIHSLQFFYTASSGLSTFPEYVIVQMVDEVQIEYYDSNTQRSIPKQDWAEQANRDTDPDY
LERDAENRKGTQQVVKANMGILKQRFNQTGGAHIIQRMDGCEWDDEDGTTEGYDQHGYDG
EDFISLDLKTMTWVAPVRQAVTTKHRLEQNRAYMEELKHYYTKECVDWLKKYLVYGKSTL
QRTERPRVSLLQRSPSSPVVCHATGFFPNRVVVFWRRDGQELHEQVDPGEVLPNHDGTFQ
VSVDLDLTAVPQEDWGRYECVVQLKGIEDISTPLDPALIRTNREGSHILAFILTGVAVLA
VVVAAVVGVFLYQKRNDSDKRHKPVGSDTSSENTEGQKLAPEAQPLTT
>GM125_AGV52667.1 MHC class I antigen, partial [*Gadus morhua*]
VIHSLQFFYTGSSGLSTFPEYVMVLMVDEVQVECYDSNTQRIITKQDWADQATRDKDPDY
LERETENRKGNQQVEKGNIGTLKKRFNQTGGAHILQRMYGCEWDDEDGTTEGYQQYGYDG
EDFLSLDLKTLIWVAPVHQAFATKLRWDHDTAYNQYLKNYYTKECVDWLKKLLAYGKSTL
QRTERPRVSLLQRSPSSPVVCHATGFYPNRVVVFWRRDGQELHEQVDPGEVLPNHDGTFQ
VSVDLDLTAVPQEDWGRYECVVRLKGIEDISTTLDPAHIRTNGGDNHILAFILTGVAVVA
VVAAVAVVGVFLYRKKNDSEKCHKPVDSDTSSENTEGQKLAPEYQPLST
>AAL14530.1 MHC class Ia antigen [*Gadus morhua*]
MKLLTGLLLLVFGHGGSSVLHSLQFFYTASSGLTAFPEFVAVGMVDGVQFYYYDSNTQRA
VLKQDWMEQVTSAHDYLKRNTENSQGSQQAFKANIGIAKQRFNQTGGAHMAQLMCGCEWD
DEDGTTDGYNQQAYDGEDFLSLDLKTMTWVAAVRQAFSTKQRWDGLKAFNEQQKHYYTVE
CVDWLKKYLAYGKSTLQRTERPRVSLLQRSPSSPVVCHATGFYPDRVVVFWRRDGQELHE
LVDPGEVLPNHDGTFQVSVDLNLKAVPQEDWGRYECVVQLRGIEDISTPLDPALIRTNGG
RTRLRVAFTIPIIIGFVVLLPAAAAAVVGVLLYKKRKPSDQRHKPVATSDTSSEDA

15. Previously published tilapia (*Oreochromis niloticus*) MHCI sequences

>orniUBA1 AB270897 Sato et al.2006, main text ref.31
KHSLKYFFTETPGAQSIPEFVGVGFIDEVQFGGWNSRRGEEVKK
DWIKLFEDDPQHLHQYIFECSASHHYFKDTIKTLKQRLNQTEDVHILQRMSGCEWDDE
TGEVNGYNQYGYDGEDFIAFDLQTVTWITPKPQAVTTKLRWNMEHARLKFNKNFFSNQ
CPEFLKRYLHYGRSFLETAVLPSVSLLQKTPSSPVSCHATGFYPQRAMMFWRKDGEEI
HEGVDYGEILPNNDGTFQMSVDLKLSSVRPEEWDRYDCVFEISGVKMRQDEDIVTKLDKAVIWTNW
>orniUBA2 AB270897 Sato et al.2006
KHSLKFFFCQTSGVQNIPEFVVVGLVDGVQKSYYDSNTGRPEPK
TEWMKKLMKDDPQHLEWYTARSFHTQDLFKHYTENLRKRFNQTEGVHILQSMNGCEWD
DETGQINAFNQYGYDGEDFLTFDPQRLTWIALKLQAVITKLRWDGEEDQLKCNKNFYI
HECPEILKKYIQCGKNFSQTAVLPSVSLLQKSSSSPVSCHATGFYPDRGLMFWRIDGE
ELHEGLDPGEILPNNDGTFQLSVDLKLSSVTPEDWQRYDCVFQLSGGEDNIVTKLNKT
LIRTNW
>orniUAA1 AB270897 Sato et al.2006
KHSLKYFVTGSSGAPNIPELFGALMVDGIQVGYCDVSKKILEPR
QEWAKNILEKHPEQLDWYQHKCFEDQPNFFRELISSLKQQFNQSEGVHILQRIDGCEW
DETTGEVIGIIQYHYNGEDFLEFDLKKRTWIALKPEADVTKQKWATDQPGIKHKENDL
TKICPDYLKLYVKHVKSSPQKKVLPSVSLLQKTPSSPVSCHATGFYPDRAVMFWRKDG
EELHEGVEIGEILPNNDGTFQLSVNLNVSSVTPEDWRRYECVFQLSDVENNSVNTLNK
TVIRTNESKRNS

16. Relevant published rainbow trout (*Oncorhynchus mykiss*) MHC class I sequences:

>onmyUAA0101 CCE21324.1 AF091779.1 MHC class I alpha chain, partial [*Oncorhynchus mykiss*] Shum et al.1999 (main text reference 27)
MITTILISFMQFSIVAPHSLHRHCIATQGTLYPKNIQLVMIDDVIVYYYNSSAEQEAVVP
EWLNHPEGIEFWQEVHRNLKFNRYVMDTAVRVTSEHYNHSHDHFYQAHGRCGWKSDGTTE
AFMSHAYDGKDFVSFDVSTRTWTAAVSHAVFYKRKRETDLEDLVRLVIHYESGCIRWLEK
LLEFSVTVREPKVPAVSLFERPPHGNSEVEVTCHVTGFYPRAVQVEWLGAEELPMVDGVN
SGEVLPNGDGSYQLRKSLTVPQEAQDTQSYSCLVLHSSIAGNITVTWAPKKNLANVLMAI
VIIVSVVLILTVLFKYLVWRRAVAQNPRG
>onmyUEA AAS93784.1| MHC class Ib antigen [Oncorhynchus mykiss]
MISFCLLFLWIPSSSADPHSLKYLYTAVSGDTDFPEFTVVGLLDDQQFVHFGSNTKTLVN
DAEWMNKTGKYYNDLYNEPLINQYEGFKNLITFAKKQFNQTQSKGVHTIQNLYGCEWNDE
TELQDYFHHYGYDGEDFISLDMKTVRWITSVQQADTIKQKWDDNSKDLHYLKWYFTKECI
DTLKKYVDFASSVLKKMVPPSVSLLQKTPSSPVTCHATGFYPSGVMVSWQKDGQEQHEDV
LHGEILLNGDGTFQKSAHLTIDSEKWENNNYTCVVEHKENIIAIRLNQSVIKTNSVKPSE
YVPIIIGLGVVAVILLVLVPIITFIMWNRKSTASDDGSNPSMTQQNQIQLEVSLSLIKQE
EAEKRGPLLKDSEAS
>onmyLAA_ABI21843.1 nonclassical MHC class I antigen [*Oncorhynchus mykiss*], Dijkstra et al.2007
MNFYTLFLIYLPIVNAGKSSHSLWGLATHVLGETEFPEFCVLWMLDDVQVGYYDSNSWRF
ISRTDENIDEEYSKTVQGASWDVYLSMRKRSSLLQHRFNSTTGIFVHQRLIGCELEDNEK
QGQLMIKEAFNGIDGGVLNFNKLQYNYHPKWPELEFNQQRTQYIQMGLDKVYLPICIKSL
KDYLKKEEKLVMRKVRPRVRLISKESTDTEGAKITCLAFGFYPRHINLTLLRDGQPVAEH
ELKGGQLLPNGDWTYQLRKSLTITVQELRERPNYTCTANHISMDNKLDVSWVPDTGPDSA
SIIPVVLVMAVVLILIGILVVIGMWKWKHAGVPTFSGHIYSAAKDTETEQSNSSLETEST
DS
>onmyLBA_ABI21844.1 nonclassical MHC class I antigen [*Oncorhynchus mykiss*], Dijkstra et al.2007
MGKLSIFLFALSFCTIVNSGSGSHSLWALATYISGETPFPEFTVVVMLDDIQVTYYDSNM
KHFIYKGHNTSDKIHDDEAKNGDFVFGVMYHHMKERYFHLKHHFNLTEGVQVQQKLTGCE
MFDNGEPALVMFKDAFNAIYTDRTLYYNMTHFTYDAGKLLLGWDGMRQAYERTLYENVYL
PICIKSLKKILKREKNVVMRKVPPRLRLIKKEVSGGFQVSCLAFGFYPRHINLTLLRDGQ
PVAEQELTGGEVLPSGDGTYQLRKSLEVSTEELKKRHNYTCTASHLSLDNKLDVSWESGA
ERVHLSTLSALLVMLLVVILFSIFICVKRRWSNTASQSKLANVDATVSEEINLSSDSET
>onmyLCA_ABI21845.1 nonclassical MHC class I antigen [*Oncorhynchus mykiss*], Dijkstra et al.2007
MGKLSVFLFVLSFCTIVNSGSGSHSLWALATYISGETPFPEFTVVVMLDDVQVTYYDSNM
KHFIYKGHNTSDKIHDDEAKNGDFVFGVMYHHMKERYFHLKHHLNLTEGVQVQQRMAGCE
MFDNGEPALIMTKNTFNAVFVDHAIYYNMTHFTYDAGKLLQGWDGMRQLQEKILYENVLL
TLCIRTLKTILKREKNVMMRKVPPRLRLIKKEVSGGFQVSCLAFGFYPRHINLTLLRDGQ
PVAEQELTGGEVLPSGDRTYQLRKRLEVSTEELKKRHNYTCTASHLSLDNKLDVSWESGA
ERVHLSTLSALLVMLLVVILFSIFICVKRRWSNTASQSKLVNVDATVSEEMNLSSDSEN
>onmyLDA_BAF37937.1 MHC class I antigen [*Oncorhynchus mykiss*], Dijkstra et al.2007
MGKLSIFLFVLSFYTIVNAGSGSHSLWALATYIIGETPFPEFTVVLMLDDVQIGYYDSNI
KLSVYRGYHITDKINDEAQDGSDVLGTMYHHMKERSFRLKHHLNLTEGVHVQQRIGGCEI
LHNGEPALIMTKNSFNGIFADYAVYYNMTHFTYDSGQLLLGYNWMRQATERTLYANVWLP
ICINTLKKCLNREKNFVMRRVPPRLRLLIKKEVSGDLQVICLAFGFYPRHINLTLLRDGH
PVAEQELTGGEVLPSGDGTYQLRKSIYVSTEELREKHNYTCTASHLSLNNKLDVSWESGA
ERVHLFILSAPLVMALIVILFCILICLARRIRAASQNLQLASVDALEADERNLSSDSEI
>onmyLEA ABI21847.1 nonclassical MHC class I antigen, partial [*Oncorhynchus mykiss*], Dijkstra et al.2007
KMHDNKAQDGAYVFGMMFNNMKERSFNLKHHFNLTEGVQVQQRMSGCEMLDNGEPVMFVV
KDTFNAIYTDQMVCYNMTHFIYDAGKLLRGWDGMRQAFERVLFENVHLPICIRTLKTILK
REKNFAMRKVPPRLRLIKKEVSGGLKVSCLAFGFYPRHINLTLLRDGQPVAEQDLTGGEV
LPSGDGTYQLRKSLEVSTEELKKRHNYTCTASHLSLDNKLDVSWESGAERVHLSTFSALL
VMLLIVILLGIFICVRRWRYTASQSKIANVDAKVSEEMNLSSDSET

17. Relevant published Cyprinid MHC class I sequences:

>caauZA1 AAA72345.1 unnamed protein product [*Carassius auratus*], Hashimoto et al.1990 (main text reference 6)
MFTVMIKADTLPVFSAVCESDHIQISHYSTEEQIWMRENLTEHDWDRAPVGPPETTDWYL
DLIRILSNRTESSDLLVLQRVIGCELEKLPDGAVNLTPVDEYGFGGEDCVDFSSDTSQCS
DKSLNVKETKIKRDHRVKLQEFFKNCLDWISTFNNTKKNSPDVGVFARKAPDDHSKLVLI
CLVTGFYPRDIEMNIRLNRINIQNQISSGVRPNDDETFQMRSSVKIDRNHRGSYDCHVIH
SSLTE
>caauZD1 AAA49185.1 MHC antigen, partial [*Carassius auratus*],Okamura et al.1993 (main text reference 32)
PSDVHVFARKSPDDHTKLNLSCLATGFYPRDIEMYIRLNRIKLEDQISSGIRPNDDETFQ
MRSSVEIDINSEGSYDCFVIHSSLTEPVSVKW
>cycaZB1 L10420 CYIMHC1C_2 *Cyprinus carpio* MHC gene, Okamura et al.1993
KFTVLSKADIFPEFSAVAVADDIQIKHYSNEDWIRSILTEDDWINAPEAPPDSRDWFLHQ
IRILSNCTDSLCSELHVLQRVIDCEREKLSDGSVNLRAFDEYGFDGEDFMAFDSETLQWI
DKHPKAKETKIKWDQQTERIQFLKQYLKNCMNWISTFNNTKKHSPDVCVFARRAPDDHSK
LVLICLVTGFYPRDVEMNIRLNRINIQNQISSGIRPNDDESFQMRSSVKIDRNHRGSYDC
HVIHSSLTE
>cycaZC1 L10421 CYIMHC1D_2 *Cyprinus carpio* MHC gene, Okamura et al.1993
KYTVLTKAGPFPVFSAVGESDHIQIARYRSEERVWMRENLTVDAWNEAPGAPAETNDGYL
DLIRILSNCTECSELHVLQRISGCELEKLPDGAVTSLKVFDEFVYDGKDFISCKYDFFPW
MDKVIETEMNHQTGQNPFLKDFLINCTKWISTFNNTYKNSPDVHVFARKAPDDHSKLVLI
CLATGFYPRDIEMNIRLDRTALGNQIFSEIRPNADGSFQLRSSVKIDRNHKGSFDCFVNH
SSLTK
>cyca-ZE*0101 Q8MGT8 MHC class I antigen *Cyprinus carpio*
MGTSVVTASFALLCVFLLCEPSLQTEKHSLYYIYTALSKPVDQPGIYQFTAMGLLDDREI
DYYNSKEQRKIPRQHWMKEKMQEDYWEKGTQSRKSKEQWFNVNVDILMKRMRHNESDVHV
FQWRHGCEIEKNGDEVKFSKGIDEYSYDGANFLSFDDKEFQWVAPVAAAVPTKRKWDNVT
ILNQYTKGYLEKECVDWLNKFREYGDEVIRKGSPPEVHVFAKRCTRDKTKLELTCFATGF
YPKDVILSIRKYRSPLPEKEIESSGVRPNHDGTYQLKKTVIIQEDEKADYDCVVSHSTLN
ETIITKWDGTCPDCGSSLVIGIVIGAVLVIVVVAAVCLYLFMTKRLTCRQQSATNGQVQG
AYEVNENLLPGYNSNGHAPTAVNGGPNGVIIIAN

18. Relevant published Paddlefish (*Polyodon spatula*) and Sturgeon (Acipenser sinensis) MHC class I sequences:

>Paddlefish UBA*01 ACV87421.1 MHC class Ia chain [*Polyodon spathula*], Wang et al.2010 (main text reference 60)
MLRAVVLAILCCFHAASAGSHSLRYFYTGTSGVTEFPEFVIVGMVDDVQISHCDSKSKQT
VPKQQWMKDNVEPGYWERNTQICLGNQQIFKVAVIDLPKRFNQTEGVHTVQRMVGCELDD
DGTKRGFEQHGYDGEDYIMFDKDTLTWTAASQRGFTTKVKWDPLTASNQQRKAYLEGTCI
EWLKKYVQYGRETLERRVPPEVTLLQRKARGSADMEVLCHVTGFFPRAVEVTWVRDGQDQ
LEEGVQNGEVLLNQDGTYQLRKILTVSPEEQGRHRYSCQVDHISFKEKQIYIWDPNMRSS
SDGGPPIGIIAGVIVGVLLLVAAVIGVVIWKRRQGGAQKSDYSKAPSKEGSDTSSDTAP
>Paddlefish UBA*03 ACV87423.1 MHC class Ia chain [*Polyodon spathula*], Wang et al.2010 (main text reference 60)
MLRAVVLAFLCCVHAASGAGTHSLRYFFTGVTAGTGLPEFVTVGLVDDEQHVHYDSVSKK
AVARQDWMAKSEGPEYWESETQNFAGEEQVFKVNIGTLMQRFNQTGGVHTVQRMYGCELD
DDGTKRGFDQFGFDGKDFIIFDKDSLSWTAPVMQAVITKNKLDADRALNQQQKAYLEQIC
IEWLQKYVQYGKETLERRVPPAVTLRHKKARGSADTEVVCHVTGFYPRAVEVTWVRDGQV
QLEDRVQSGEVLPNQDGTYQLRKILTVSPEEQGRHSYSCQVDHVSFTERQNYIWDPNMRS
SSDGGSPIGLIIGVIVGVLALVGVIVGVVAWKKKQGAPKNYNPARTNDGSDTSSDTAP
>Sturgeon U lineage ACV87437.1 MHC class Ia chain [*Acipenser sinensis*] Wang et al.2010 (main text reference 60)
MLRAVVLAILCCVHAESGTRTHSLRYFYTGTSGMTEFPEFVAVGMVDDVQIDYYDSKSKK

DISKQQWMKDNMEPAYWEGNTQKCLGHQQNFKANIGIAMQRFNQTGGVHTAQTMLGCELD
EDGTKRGFWQEGYDGEDYIIFDKDTLTWTAANQRGFTTKVKWDPNTARNQYLKGYLEGTC
IEWLQKYVQYGRETLERRVPPAVTLLQRKARGSADTEVLCHVTGFFPRAVEVTWVRDGRD
QLEEGVQSGEVLPNQDGTYQLRKILTLSPEEQGRHSYSCQVDHISLDQKIVKEWDPNMRS
SSDVPESSSPIGLIAGVIVGVLAVAAVIIGVVIWKKRQGGAQKNYTPAQTNEGSDTSSDT
AP

19. Relevant published Shark, lungfish, chicken, Human, MHC class I sequences:

>Spiny dogfish AAN77874.1 MHC class I antigen [*Squalus acanthias*]
MIRLIVLALLCGGVSAGTHSLRYFYNSMTPIPGVPEFVAVGYVDDALFVHYDSDRKQMIP
RQRWIEESEDKQYWERETQKQLGWEQIGKVDIQTLITRTNLTGGIHTLQVMYGCELRDDG
STAGFFQYGWDGKDLISFDKEHLVWNTPVTWQVVTKNKWEQDRGLGQQRKGYLEQECIEW
LKKYLTAGERELKPVAPRVFPSVNKASNIRPTELSCLVTGFYPRDIEVTLLRNGQPITDT
ESTGILPNHDGTYQLTRWAQITLDEGATYSCQYDQGDKVGVEIRHWDGTFPGSPEGTNLG
LIVGIVIGAVALIALVIGAVVWKRRGEKKSGYNPANPAERGESSSNSSAQA
>Elephant shark AFM85876.1 MHC class I antigen, partial [Callorhinchus milii]
MLQKLLLLTALCGAALAGSHSLRYFYTAVHGAPGVPEFSIVGYVDDQQFVRYQSNSESRR
MEPRQRWIQEREDAGYWDGQTRTAQGWEQTYKVNVKTVMGRYNQTGGIHTVQQMYGCELK
GDGSVGGFIQYGYDGNDFIAFDKDRKVWTAPTAAAVVTKNKWENTPGLSEQEKDYLEQIC
IEWLRKYVEYGHESLRPVRPEVTLSSPRGSRLSCLAAGFYPRAIEVKLLKNGQILSDEES
PGTSPNQDGTFQLRKSVEINPTATETYSCQVEHSSLTTPFTVVYTGPEVGGGPPTLIIVV
VVIVVLLIVAAAIGGFYL
>Lungfish AAF15304.1 Z-like MHCI sequence [*Protopterus aethiopicus*]
MPGESLKVPQALLVCWFLSVQFVDSSFHSLRYTYSAYASNDKLVEFVAQGLLDDVQIDYY
DNHIRREVPKQQWMNESMEAGYWERGTQSRNSKEHWFTVNTQIVMQRRNDTSGYHTLQWV
HGCSLTDGIKIGIDQYAYDGEDFLSFDKEKLSWIAVNKAAQQTREKWDEEKNLNQYTKRY
LEQECIEWLQNFLRFSNGKLEKKVGPETKIFRKRRHDGKYTFSCHASEFFPSALSIKWIN
IKTNEKKEETAEALPNNDGTFQAWVDIDSDLECARDLVCMITHGNSSEEHRIPAEPLRSR
KVFLYIIIIAVVLLLLSAAVLLLLFLKKKKKCAYANEVIAENYLEEAGLPAVAKDAVKDE
TADLLIKDEKLLLLLLTKLQIKMKQVTH
>Chicken Q31400 (*Gallus gallus*) MHC class I alpha chain BF2
MGPCGALGLGLLLAAVCGAAAELHTLRYIQTAMTDPGPGQPWFVTVGYVDGELFVHYNST
ARRYVPRTEWIAAKADQQYWDGQTQIGQGNEQIDRENLGILQRRYNQTGGSHTVQWMYGC
DILEGGPIRGYYQMAYDGRDFTAFDKGTMTFTAAVPEAVPTKRKWEEESEPERWKNYLEE
TCVEWLRRYVEYGKAELGRRERPEVRVWGKEADGILTLSCRAHGFYPRPIVVSWLKDGAV
RGQDAHSGGIVPNGDGTYHTWVTIDAQPGDGDKYQCRVEHASLPQPGLYSW EPPQPNLVP
IVAGVAVAIVAIAIMVGVGFIIYRRHAGKKGKGYNIAPDREGGSSSSSTGSNPAI
>HLA-A2 AAA76608.2 HLA-A2 [*Homo sapiens*]
MAVMAPRTLVLLLSGALALTQTWAGSHSMRYFFTSVSRPGRGEPRFIAVGYVDDTQFVRF
DSDAASQRMEPRAPWIEQEGPEYWDGETRKVKAHSQTHRVDLGTLRGYYNQSEAGSHTVQ
RMYGCDVGSDWRFLRGYHQYAYDGKDYIALKEDLRSWTAADMAAQTTKHKWEAAHVAEQL
RAYLEGTCVEWLRRYLENGKETLQRTDAPKTHMTHHAVSDHEATLRCWALSFYPAEITLT
WQRDGEDQTQDTELVETRPAGDGTFQKWAAVVVPSGQEQRYTCHVQHEGLPKPLTLRWEP
SSQPTIPIVGIIAGLVLFGAVITGAVVAAVMWRRKSSDRKGGSYSQAASSDSAQGSDVSL
TACKV
>HLA-B27 CAA83876.1 human lymphocyte antigen[*Homo sapiens*]
MRVTAPRTLLLLLWGAVALTETWAGSHSMRYFHTSVSRPGRGEPRFITVGYVDDTLFVRF
DSDAASPREEPRAPWIEQEGPEYWDRETQICKAKAQTDREDLRTLLRYYNQSEAGSHTLQ
NMYGCDVGPDGRLLRGYHQHAYDGKDYIALNEDLSSWTAADTAAQITQRKWEAARVAEQL
RAYLEGECVEWLRRYLENGKETLQRADPPKTHVTHHPISDHEATLRCWALGFYPAEITLT
WQRDGEDQTQDTELVETRPAGDRTFQKWAAVVVPSGEEQRYTCHVQHEGLPKPLTLRWEP
SSQSTVPIVGIVAGLAVLAVVVIGAVVAAVMCRRKSSGGKGGSYSQAACSDSAQGSDVSL
TA
>HLA-Cw6 CAA80437.1| [*Homo sapiens*]
MRVMAPRTLILLLSGALALTETWACSHSMRYFDTAVSRPGRGEPRFISVGYVDDTQFVRF
DSDAASPRGEPRAPWVEQEGPEYWDRETQKYKRQAQADRVNLRKLRGYYNQSEDGSHTLQ
WMYGCDLGPDGRLLRGYDQSAYDGKDYIALNEDLRSWTAADTAAQITQRKWEAAREAEQW

RAYLEGTCVEWLRRYLENGKETLQRAEHPKTHVTHHPVSDHEATLRCWALGFYPAEITLT
WQRDGEDQTQDTELVETRPAGDGTFQKWAAVVVPSGEEQRYTCHVQHEGLPEPLTLRWEP
SSQPTIPIVGIVAGLAVLAVLAVLGAVMAVVMCRRKSSGGKGGSCSQAASSNSAQGSDES
LIACKA
>HLA-G EAX03236.1 [*Homo sapiens*]
MKTPRMVVMAPRTLFLLLSGALTLTETWAGSHSMRYFSAAVSRPGRGEPRFIAMGYVDDT
QFVRFDSDSACPRMEPRAPWVEQEGPEYWEEETRNTKAHAQTDRMNLQTLRGYYNQSEAS
SHTLQWMIGCDLGSDGRLLRGYEQYAYDGKDYLALNEDLRSWTAADTAAQISKRKCEAAN
VAEQRRAYLEGTCVEWLHRYLENGKEMLQRADPPKTHVTHHPVFDYEATLRCWALGFYPA
EIILTWQRDGEDQTQDVELVETRPAGDGTFQKWAAVVVPSGEEQRYTCHVQHEGLPEPLM
LRWKQSSLPTIPIMGIVAGLVVLAAVVTGAAVAAVLWRKKSSD
