# Supplementary material for: A comprehensive analysis of teleost MHC class I sequences
Source: BMC Evol Biol. 2015 Mar 6;15:32. doi: 10.1186/s12862-015-0309-1 (PMC4364491; doi:10.1186/s12862-015-0309-1)
Supplement: Additional file 5: Table S2. — Regional syntenies in selected MHCI regions. [file 12862_2015_309_MOESM5_ESM.pdf]

Additional file 5: Table S2. Regional syntenies in selected MHC class I regions

Table of Contents

	Page
Cavefish MHC class I regions vs. Medaka, Stickleback, Tetraodon, Zebrafish, Fugu and Tilapia	2
Zebrafish MHC class I regions vs.Tetraodon, Stickleback, Tilapia, Medaka, Fugu and Cavefish	3
Medaka MHC class I regions vs. Cavefish, Stickleback, Tetraodon, Zebrafish, Fugu and Tilapia	4
Tilapia MHC class I regions vs. Cavefish, Stickleback, Medaka, Zebrafish, Fugu and Tetraodon	6
Tetraodon MHC class I regions vs. Zebrafish, Stickleback, Tilapia, Medaka, Fugu and Cavefish	6
Fugu MHC class I regions vs. Cavefish, Stickleback, Medaka, Zebrafish, Tilapia and Tetraodon	8
Atlantic salmon MHC class I regions vs Cavefish, Stickleback, Medaka, Zebrafish, Tilapia and Tetraodon	8

Legend to Table S2. The table shows a summary of synteny searches using Ensembl Biomart with Ensembl gene IDs from the selected species against other teleosts with Ensembl genomes. Regions with MHC genes only, with genes on one side of the MHC class I gene only or highly abundant genes were omitted from the analysis or not shown. Although seemingly omitted, stickleback is represented through analysis of regions in other species. In gene abundant regions only a few genes on each side of the MHCI gene were chosen for synteny analysis, but a complete gene organization of the regions can be found in Additional file 1: Figure 1. Regional synteny is shown with pink shading and represents one syntenic gene on both sides of the MHC class I gene within a 2 Mb region. Grey shading shows genes syntenic on one side of the MHCI gene only. MHC class I genes are shown with red font and synteny groups S1-S11 (see also Additional file 1: Figure S1) are shown with green font. Abbreviations are as follows: n.m.= no match, Scf.=scaffold, Chr.=chromosome, UnR= Unrandom, UC= ultracontig, R= random.

Cavefish		Medaka	Stickleback	Tetraodon	Zebrafish	Fugu	Tilapia
PPFIBP1_L	KB871893: 38 kb	Chr.23:20,051,197	Gr.IV:31,951,426	Chr.19:1,276,671	Chr.4:9,877,785	Scf.21:1,208,924	GL831243.1:195,805
CYB5R3	KB871893: 68 kb	Chr.23:20,049,871	Gr.IV:31,945,908	Chr.19:1,273,521	Chr.4:10,869,199	Scf.21:1,205,622	GL831243.1:216,720
ENDO-U_L	KB871893: 81 kb	Chr.17:4,352,014	Gr.III:10,946,082	15_R:1,629,193	Chr.4:11,098,397	Scf.68:586,341	GL831198.1:809,382
AM5+6 (P)	KB871893: 114 kb	n..m	n.m.	n.m.	n..m	n..m	n.m.
CRY1a	KB871893: 140 kb	Chr.23:20,205,991	Gr.IV:32,004,462	n.m.	Chr.4:11,077,567	Scf.21:1,258,914	GL831243.1:32,386
BTBD11a	KB871893: 201 kb	Chr.23:20,352,410	Gr.IV:32,012,155	Chr.19:1,359,678	Chr.4:10,878,302	Scf.21:1,316,032	GL831195.1:130,614
AP3S2	KB872367.1: 1 kb	Chr.3:31,732,658	Gr.II:4,577,683	UnR:93,586,915	Chr.25:10,975,095	Scf.91:199,262	n.m.
ANPEPB	KB872367.1: 12 kb	Chr.6:22,383,739	GR.XIX:20,215,845	UnR:89,033,143	Chr.25:11,024,630	Scf.727:1,102	n.m.
AM12 (L ψ) S5	KB872367.1: 53 kb	n.m.	n.m.	n.m.	DR17=Chr.25:11.063 kb	n.m.	n.m.
SV2BB	KB872367.1: 86 kb	Chr.6:22,425,407	Gr.XIV:6,533,608	Chr.4:1,973,708	Chr.25:11,109,520	Scf.84:324,086	n.m.
ITF20	KB882095: 2,979 kb	Chr.13:17,426,676	Gr.I:1,185,206	Chr.16:8,301,165	Chr.15:23,138,390	Scf.300:33,201	GL831270:1,511,708
RAD1	KB882095: 2,984 kb	Chr.12:9,107,064	Gr.XIV:4,020,462	Chr.4:6,416,908	Chr.15:23,134,045	Scf.44:1,100,560	GL831236:2,163,659
BBC3	KB882095:3,002 kb	n.m.	Gr.I:15,767,604	n.m.	Chr.15:23,098,765	n.m.	n.m.
AM32 (Lψ)	KB882095: 3,016 kb	n.m.	n.m.	n.m.	n.m.	n.m.	n.m.
SAE1	KB882095:3,025 kb	Chr.13:3,198,680	Gr.I:15,780,868	Chr.16:6,635,208	Chr.15:23,076,168	Scf.181:160,670	GL831340:733,560
ZC3H4	KB882095:3,045 kb	n.m.	Gr.I:15,798,118	Chr.16:6,624,795	Chr.15:23,062,877	Scf.181:150,812	GL831340:716,603
CDC42EP3_L	KB882095:3,073 kb	Chr.13:3,255,047	Gr.I:15,826,511	Chr.16:6,609,798	Chr.15:23,047,062	Scf.181:135,622	GL831340:689,085
Eya3	KB882192: 1,697 kb	Chr.11:19,186,115	Gr.X:2,013,153	21_R:68,229	Chr.19:25,377,412	Scf.69:955,934	GL831186.1:2,498,290
SGK1_L	KB882192: 1,713 kb	Chr.11:19,179,849	Gr.X:2,003,674	21_R:63,142	Chr.19:25,368,510	Scf.69:949,168	GL831186.1:2,489,950
AM33+34 (S)	KB882192: 1,855 kb	n..m	GA17= Gr.X:1,144 kb	n.m.	n.m.	n.m.	n.m.
MYO1G_L	KB882192: 1,895 kb	Chr.11:19,070,125	Gr.X:1,935,507	21_R:7,683	Chr.19:25,126,132	Scf.69:906,463	GL831186.1:2,336,517
CDK5R1_L	KB882192: 2,046 kb	Chr.11:19,056,775	Gr.X:1,920,938	UnR:102,975,220	Chr.19:25,081,989	Scf.69:899,997	GL831186.1:2,320,452
DLB	KB882234: 114 kb	Chr.14:681,908	Gr.VII:12,730,248	UnR:88,691,267	Chr.5:38,294,022	Scf.99:593,698	GL831214.1:2,082,367
NAIF1_L	KB882234: 140 kb	Chr.14:665,964	Gr.VII:12,746,132	Chr.7:1,127,030	Chr.5:38,266,878	Scf.99:585,052	GL831214.1:2,062,584
ALKBH6	KB882234: 174 kb	Chr.21:18,527,000	Gr.XVI:6,372,279	Chr.2:20,688,290	Chr.5:38,250,227	Scf.38:748,717	GL831428.1:495,118
NOVA1	KB882234: 202 kb	Chr.14:595,896	Gr.VII:12,768,515	Chr.7:1,145,713	Chr.5:38,211,291	Scf.99:551,438	GL831214.1:2,008,471
MICU2	KB882234: 255 kb	Chr.14:909,921	Gr.VII:12,597,726	UnR:84,864,439	Chr.5:38,199,270	Scf.576:509	GL831214.1:2,247,525
AM35-37 (U)	KB882234: 282 kb	n.m.	n.m.	n.m.	n.m.	n.m.	n.m.
SPTBN4_L	KB882234: 341 kb	Chr.14:922,987	Gr.VII:12,566,414	UnR:59,916,599	Chr.5:24,248,414	Scf.642:20,538	n.m.
RPL36a	KB882234: 433 kb	Chr.10:3,723,391	Gr.IV:7,480,346	Chr.1:4,940,118	Chr.5:24,293,967	Scf.5:1,337,548	GL831169.1:1,723,199
GLRA4b	KB882234: 447 kb	Chr.14:17,423,078	Gr.VII:17,036,480	Chr.7:6,512,293	Chr.5:24,298,675	Scf.6:1,195,576	GL831331.1:1,216,094
AIFM1	KB882234: 496 kb	Chr.14:17,407,312	Gr.VII:17,045,611	Chr.7:6,504,162	Chr.5:24,333,942	Scf.6:1,202,682	GL831331.1:1,198,802
TNFSF1014	KB882234: 550 kb	n.m.	n.m.	n.m.	Chr.5:24,363,303	n.m.	n.m.

ATG4Da	KB882301: 1,045 kb	Chr.1:14,295,975	Gr.IX:906,876	Chr.18:4,537,422	Chr.1:51,895,489	Scf.207:13,240	n.m.
ACTR2	KB882301: 1,071 kb	n.m.	Scf.216:55,071	n.m.	n.m.	n.m.	n.m.
SPRED2a	KB882301: 1,103 kb	n.m.	Gr.IX:18,695,601	UnR:70,161,706	Chr.1:51,953,389	Scf.102:24,266	GL831344.1:811,687
MEIS4.1a_L	KB882301: 1,146 kb	n.m.	Gr.IX:18,708,797	UnR:70,156,493	Chr.1:51,983,934	Scf.102:32,929	GL831344.1:783,640
CNRIP1a	KB882301: 1.309 kb	n.m.	Gr.IX:18,766,878	n.m.	Chr.1:52,102,724	Scf.102:50,925	GL831344.1:609,901
**AM38-42 (S)**	KB882301: 1.321 kb	n..m	n.m.	n.m.	n.m.	n.m.	n.m.
RNF11a	KB882301: 1.433 kb	Chr.1:12,283,773	Gr.IX:17,759,743	Chr.18:4,656,119	Chr.1:52,625,090	Scf.:207:127,474	n.m.
RAD23aa	KB882301: 1.445 kb	Chr.8:22,965,145	Gr.XI:2,238,081	UnR:61,172,274	Chr.1:52,605,851	Scf.41:1,403,726	GL831179.1:4,115,841
NFIXA	KB882301: 1,478 kb	Chr.1:12,200,983	Gr.IX:17,689,001	Chr.18:4,667,055	Chr.1:52,397,836	Scf.207:137,227	GL831193.1:938,608
**Zebrafish**		**Tetraodon**	**Stickleback**	**Tilapia**	**Medaka**	**Fugu**	**Cavefish**
AGPAT3	Chr. 1: 47,334 kb	Chr.2:16,781,289	Gr.XVI:13,923,149	GL831170:3,176,631	Ultra2:1,206,836	Scf.83:1,068,504	KB882144.1:409,938
PDXKB	Chr.1: 47,385 kb	Chr.17:8,698,907	Gr.VI:15,490,151	GL831135:11,336,650	n.m.	Scf.113:708,940	KB882144.1:458,867
CRYZL1	Chr.1: 47,586 kb	Chr.17:8,735,021	Gr.VI:14,567,755	GL831135:11,266,859	Scf.1229:21,394	Scf.113:747,571	KB882144.1:656,763
SETD4	Chr.1: 47,603 kb	Chr.17:8,738,071	Gr.VI:14,572,247	GL831135:11,259,492	Chr.15:298,831	Scf.113:749,101	KB882144.1:662,310
**DR1-4 (Z)** S2	Chr.1: 47,613 kb	n.m.	n.m.	n.m.	n.m.	n.m.	n.m.
CBR1	Chr.1: 47,662 kb	Chr.17:8,741,087	Gr.VI:14,576,350	GL831135:11,250,427	Chr.15:306,701	Scf.113:754,656	KB882144.1:682,954
MORC3B	Chr.1: 47,675 kb	n.m.	n.m.	n.m.	n.m.	n.m.	KB882144.1:694,048
BCL9	Chr.1: 47,828 kb	Chr.2:12,224,593	Gr.XVI:8,409,957	GL831311:417,460	Chr.21:27,067,144	Scf.103:940,004	KB882144.1:833,740
GCDHL	Chr.3:467 kb	Chr.18:5,322,352	m.m.	GL831274:1582315	Chr.8:22,151,606	Scf.62:261,860	KB880151.1:21
**DR5 (Z)**	Chr.3:502 kb	n.m.		n.m.	OL1=Chr.8: 24.973 kb	n.m.	n.m.
KCNJ4	Chr.3:531 kb	UnR:5,685,253	GrXI:4,200,864	GL831154:2,297,712	Chr.8:19,756,912	Scf.41:400,490	KB872106.1:113,413
KDELR3	Chr.3:716 kb	UnR:5,691,317	GrXI:4,190,072	GL831154:2,283,059	Chr.8:19,783,365	Scf.41:390,686	KB872106.1:663,013
DDX47	Chr.3:988 kb	n.m.	n.m.	GL831154:1,658,172	Chr.8:20,404,649	n.m.	n.m.
**DR6-9 (Z)**	Chr.3:1,072 kb	TN3= Un_R: 6.398 kb	n.m.	n.m.	n.m.	n.m.	n.m.
CCDC134	Chr.3:1,157 kb	n.m.	n.m.	GL831154:2,190,101	Chr.8:19,897,755	Scf.2134:7,970	KB872394.1:72,214
RAPGEFL1	Chr.3:23,621 kb	Chr.3:15,206,192	Scf.622:696	GL831151:988,576	n.m.	Scf.307:149,858	KB882082.1:2,573,801
CASC3	Chr.3:23,720 kb	Chr.3:15,195,552	GrXI:16,703,692	GL831151:1,295,705	n.m.	Scf.307:138,561	KB871704.1:341,106
MSL1	Chr.3:23,756 kb	n.m.	n.m.	n.m.	n.m.	n.m.	KB871704.1:409,671
**DR10 (L)**	Chr.3:23,,762 kb	TN3= UnR: 6.398 kb	n.m.	n.m.	n.m.	n.m.	n.m.
NR1D1	Chr.3:23,779 kb	n.m.	n.m.	GL831151:920,526	n.m.	n.m.	KB871704.1:426,429
IGF2BP1	Chr.3:23,815 kb	UnR:5,320,549	GrXI:1,217,111	GL831179:604,597	n.m.	Scf.2384:12,373	KB871704.1:456,317
UBE2Z	Chr.3:23,875 kb	UnR:5,626,841	GrXI:1,438,972	GL831733:68,023	Chr.8:24,226,472	Scf.41:461,228	KB871704.1:517,780
ATP5G1	Chr.3:23,890 kb	UnR:5,621,614	GrXI:1,445,791	GL831733:32,940	n.m.	Scf.41:467,476	KB871704.1:534,325
LSM1	Chr.8:46,922 kb	UnR:65,942,413	GrXIII:13,170,146	GL831172:1,115,727	Chr.9:18,440,734	Scf.442:41,505	KB882105.1:492,854
BAG4	Chr.8:46,929 kb	UnR:65,944,659	GrXIII:13,167,467	GL831172:1,111,326	Chr.9:18,438,330	Scf.442:38,830	KB882105.1:496,313
RTDR1	Chr.8:46,954 kb	n.m.	Scf.150:112,809	GL831312:66,763	n.m.	Scf.114:604,297	KB882105.1:515,648
MHCII	Chr.8:46,979 kb	n.m.	n.m.	GL831312:112,497	n.m.	n.m.	n.m.
**DR11+12 (L)**	Chr.8:47,093 kb	TN14= UnR: 66.895 kb	n.m.	n.m.	n.m.	n.m.	n.m.
PABPC1	Chr.8: 47,170 kb	Chr.9:6,330,537	GrXII:650,653	GL831137:7,862,234	Chr.7:16,844,411	Scf.75:1,035,824	KB872505.1:14,525
UBAP2	Chr.8: 47,194 kb	Chr.12:7,056,141	GrXIII:8,149,971	GL831238:210,678	Chr.9:11,395,215	Scf.50:58,566	KB882289.1:1,364,878
ADAMTS13	Chr.8: 47,245 kb	UnR:31,470,879	GrXIII:13,880,401	GL831172:2,043,825	Chr.9:19,453,416	Scf.18:1,089,653	KB882289.1:1,387,404

ZNF384	Chr.19:7,588 kb	UnR:16,437,671	Gr.X:59,283	GL831289:88,647	Chr.11:15,481,577	Scf.61:31,673	KB882167.1:1,111,939
KIFC1	Chr.19:7,613 kb	UnR:77,013,140	Gr.X:49,650	GL831289:79,719	Chr.11:15,474,058	Scf.61:24,111	KB882167.1:1,097,257
ZBTB22B	Chr.19:7,623 kb	UnR:16,427,766	Gr.X:44,121	GL831289:73,631	Chr.11:15,468,153	Scf.61:19,638	KB882167.1:1,084,975
DAXX	Chr.19:7,636 kb	UnR:77,028,826	Gr.X:28,778	GL831594:63,643	Chr.11:15,459,697	Scf.61:13,157	KB882167.1:1,063,507
TAPBP	Chr.19:7,661 kb	UnR:51,472,461	Gr.X:24,836 (m.m.)	GL831594:79,893	Chr.11:15,453,862	Scf.61:9,142	KB882167.1:1,052,277
**DR13+14 (U)**	Chr.19:7,667 kb	TN8= UnR:16,359 kb	GA1 =Gr.X:15 kb	(ON10-21/ ON36-39)	OL10 =Chr.11:15.359 kb	TR3= Scf.61:4 kb	n.m.
BRD2	Chr.19:7,775 kb	UnR:16,332,513	Scf.58:706,703	GL831408:575,195	Chr.11:15,241,334	Scf.523:14,385	KB882167.1:971,140
HSD17B8	Chr.19:7,796 kb	UnR:16,327,246	Gr.X:37,874	GL831408:567,313	Chr.11:15,231,491	Scf.523:21,963	KB882167.1:961,915
COL11A2	Chr.19:7,802 kb	UnR:16,312,942	Scf.58:671,489	GL831408:524,645	Chr.11:15,190,534	Scf.523:29,998	KB882167.1:897,732
RXRB	Chr.19:7,871 kb	UnR:16,279,716	Scf.58:629,374	GL831408:481,721	Chr.11:15,140,156	Scf.523:75,325	KB882167.1:848,860
NCL1	Chr.22:4,937 kb	Chr.1:16,040,725	Gr.VIII:13,335,579	GL831140:1,212,314	Chr.4:34,577,946	Scf.122:497,210	KB882097.1:3,583,760
S1PR4	Chr.22:5,024 kb	Chr.1:16,066,110	Gr.VIII:13,303,558	n.m.	Chr.4:34,628,852	Scf.122:526,251	KB882097.1:3,631,106
MYO9B	Chr.22:5,036 kb	Chr.1:16,080,985	Gr.VIII:13,255,573	GL831140:1,069,595	n.m.	Scf.122:542,595	n.m.
**DR15+16 (U)**	Chr.22:5,239 kb	n.m.	n.m.	n.m.	n.m.	n.m.	n.m.
GGH	Chr.22:5,532 kb	n.m.	n.m.	n.m.	n.m.	n.m.	KB882145.1:7,405,524
CCDC51	Chr.22:5,580 kb	UnR:16,763,866	Gr.XVII:837,614	GL831197:2,751,123	Scf.3784:5205	Scf.538:39,791	KB882145.1:7,463,652
AP3S2	Chr.25:10,975 kb	UnR:93,586,915	Gr.II:4,577,683	GL831133:5,867,342	Chr.3:31,732,658	Scf.91:199,262	KB872367.1:1,164
C25H11orf24	Chr.25:10,991 kb	n.m.	n.m.	GL831206:2,897,685	Ultra72:544,503	n.m.	n.m.
ANPEP	Chr.25:11,045 kb	UnR:89,025,077	n.m.	GL831206:3,145,127	Chr.6:22,407,366	Scf.727:8,588	KB882132.1:3,144,499
**DR17-29 (L)** **S5**	Chr.25:11,063 kb	n.m.	n.m.	n.m.	n.m.	n.m.	n.m.
MESP_L	Chr.25:11,382 kb	UnR:93,568,469	Gr.II:4,606,219	n.m.	Chr.3:31,681,338	Scf.91:174,282	n.m.
SV2BB	Chr.25:11,405 kb	n.m.	n.m.	GL831206:3,102,546	Chr.6:22,425,407	n.m.	KB872367.1:86,595
**Medaka**		**Cavefish**	**Stickleback**	**Tetraodon**	**Zebrafish**	**Fugu**	**Tilapia**
RAB11FIP4A	Chr.8: 24,838 kb	KB871771.1:612,223	Gr.XI:1,268,646	UnR:5,287,742	Chr.3:44,274,208	Scf.41:869,108	GL831179.1:670,563
AP2A1	Chr.8: 24,853 kb	KB882142.1:1,515,996	Gr.XI:457,068	Chr.3:7,991,927	Chr.3:32,503,428	Scf.438:44,023	GL831357.1:331,345
ABCC1_L	Chr.8: 24,878 kb	KB871842.1:209,544	Scf.80:184,463	Chr.3:8,026,093	Chr.3:36,447,000	Scf.438:98,093	GL831357.1:452,754
**OL1 (U)**	Chr.8: 24,973 kb	n.m.	n.m.	n.m.	n.m.	n..m	n.m.
SNRNP70	Chr.8: 25,027 kb	KB882142.1:2,282,853	Scf.80:215,762	Chr.3:7,940,106	Chr.3:32,062,043	Scf.151:446,650	GL831357.1:430,018
LIN7B	Chr.8: 25,039 kb	KB882142.1:2,271,646	Scf.80:222,212	Chr.3:7,949,364	Chr.3:32,070,869	Scf.151:457,543	GL831357.1:413,103
PPP1R35_L	Chr.8: 25,046 kb	KB871790.1:519,564	n.m.	n.m.	Chr.20:54,835,862	n.m.	GL831357.1:381,851
PRMT1	Chr.8: 25,049 kb	KB882142.1:1,550,847	Gr.XI:482,609	Chr.3:8,013,599	Chr.3:32,493,809	Scf.438:84,179	GL831357.1:373,322

CD209_L	Chr.11: 1,361 kb	n.m.	Gr.IX:12,189,816	n.m.	n.m.	n.m.	GL831254.1:2,100,415
**OL3 (U)** **S1**	Chr.11: 1,378 kb	n.m.	n.m.	n.m.	n.m.	n.m.	n.m.
ZHX2 (1 of 2)	Chr.11: 1,722 kb	KB871714.1:141,946	Scf.1093:2,876	Chr.8:3,206,556	n.m.	Scf.291:319,207	GL831550.1:158,298
ZHX1	Chr.11: 1,820 kb	KB872819.1:802,920	Scf.472:6,946	UnR:15,907,803	n.m.	Scf.2087:4,843	GL831550.1:266,213
**OL4-7 (Z)**	Chr.11: 1,867 kb	n.m.	n.m.	n.m.	n.m.	n.m.	n.m.
TNXB (1 of 2)	Chr.11: 1,990 kb	KB871620.1:1,045,175	Gr.X:14,996,770	Chr.2:2,757,268	Chr.1:59,843,267	Scf.59:93,065	GL831484.1:87,754
ATF6B	Chr.11: 2,063 kb	n.m.	Gr.X:15,029,689	n.m.	n.m.	n.m.	n.m.
VPS52	Chr.11: 2,196 kb	KB872097.1:117,050	Gr.X:15,053,871	UnR:55,652,980	Chr.19:18,319,012	Scf.69:1,076,609	GL831741.1:71,313
RPS18	Chr.11: 2,209 kb	KB872097.1:147,565	Gr.X:15,061,664	UnR:55,650,281	Chr.19:18,309,520	Scf.69:1,073,156	GL831741.1:85,329
RING1	Chr.11: 2,213 kb	KB872097.1:155,811	Gr.X:15,063,627	n.m.	n.m.	n.m.	GL831741.1:91,019
COL11A2	Chr.11: 15,190 kb	KB882167.1:897,732	Scf.58:671,489	UnR:16,312,942	Chr.19:7,802,938	Scf.523:29,998	GL831408.1:524,645
HSD17B8	Chr.11: 15,231 kb	KB882167.1:961,915	Gr.X:37,874	UnR:16,327,246	Chr.19:7,796,149	Scf.523:21,963	GL831408.1:567,313
RING3 (BRD2)	Chr.11: 15,241 kb	KB882167.1:971,140	Scf.58:706,703	UnR:16,332,513	Chr.19:7,775,502	Scf.523:14,385	GL831408.1:575,195
**OL9 (U)**	Chr.11: 15,263 kb	n.m.	GA20=Scf.58:800 kb	n.m.	n.m.	n.m.	ON10=GL831408.1:690 kb
PSMB9	Chr.11: 15,190 kb	KB882167.1:1,028,343	Scf.58:980,864 (++)	UnR:16,351,553	Chr.19:7,742,916	n.m.	GL831408.1:632,588
PSMB9-LIKE	Chr.11: 15,312 kb	KB882167.1:1,034,044	Scf.58:934,200 (++)	UnR:16,353,072	Chr.19:7,737,759	Scf.2168:16,602	GL831408.1:639,745
**OL10-11 (U)**	Chr.11: 15,417 kb	n.m.	GA1 =Gr.X:15,422 kb	TN8 =UnR:16,359 kb	UBA=Chr.19:7,670 kb	TR3=Scf.61:4 kb	ON36=GL831594.1:49 kb
DAXX	Chr.11: 15,459 kb	KB882167.1:1,063,507	Gr.X:28,778	UnR:16,421,643	Chr.19:7,636,988	Scf.61:13,157	GL831594.1:63,643
KNSL2 (KIFC1)	Chr.11: 15,474 kb	KB882167.1:1,097,257	Gr.X:49,650	UnR:16,431,265	Chr.19: 7,613,506	Scf.61:24,111	GL831594.1:79,719
FLOT1a	Chr.11: 15,494 kb	KB882167.1:1,134,654	Gr.X:73,294	UnR:16,446,022	Chr.19:7,560,892	Scf.61:41,300	GL831594.1:104,075
TUBB	Chr.11: 15,501 kb	KB882167.1:620,818	Gr.X:14,690,304	UnR:9,774,724	Chr.1:56,764,159	Scf.3284:1,316	GL831594.1:115,831
SLC35G2 a	Chr.22: 5,251 kb	KB882088.1:4,215,392	Gr.XV:9,862,819	Chr.10:9,302,854	Chr.2:24,759,987	Scf.158:635,886	GL831153.1:1,492,218
PLS1	Chr.22: 5,296 kb	KB882137.1:1,842,668	Gr.XV:9,826,568	Chr.10:9,276,349	Chr.2:7,845,116	Scf.158:660,269	GL831153.1:1,385,529
GYG1A	Chr.22: 5,307 kb	KB882088.1:4,171,977	Gr.XV:9,818,878	Chr.10:9,269,791	Chr.2:24,706,355	Scf.158:668,094	GL831153.1:1,371,706
HLTF	Chr.22: 5,316 kb	KB882088.1:4,192,543	Gr.XV:9,809,272	Chr.10:9,262,921	Chr.2:24,713,629	Scf.158:675,420	GL831153.1:1,354,293
PAK2	Chr.22: 5,338 kb	KB871801.1:481,580	Gr.XV:9,799,234	Chr.10:9,254,613	Chr.2:36,337,663	Scf.158:685,453	GL831153.1:1,338,789
OL12 (U)	Chr.22: 5,412 kb	n.m.	n.m.	n.m.	n..m	n.m.	n.m.
ELAVL1	Chr.22: 5,564 kb	KB871801.1:214,837	Gr.XV:9,709,554	Chr.10:9,237,747	Chr.2:36,844,171	Scf.2115:10,625	GL831153.1:3,469,526
SLC1A8B	Chr.22: 5,577 kb	KB871801.1:227,761	Gr.XV:9,718,255	Chr.10:9,242,160	Chr.2:36,831,337	Scf.2115:885	GL831153.1:3,477,339
REEP6	Chr.22: 5,594 kb	KB871801.1:3,867,245	Gr.XV:9,729,005	Chr.10:9,196,706	Chr.11:7,408,056	Scf.10:2,763,344	GL831153.1:3,493,059
DIRAS1A	Chr.22: 5,613 kb	KB871801.1:3,940,995	Gr.XV:9,744,821	Chr.10:9,182,921	Chr.11:7,470,877	Scf.10:2,753,701	GL831153.1:3,512,268
VGLL1	Chr.22: 5,645 kb	KB872151.1:132,432	Gr.XV:7,594,721	Chr.10:7,534,109	Chr.17:27,293,547	Scf.10:1,002,339	GL831263.1:105,731

Tilapia		Cavefish	Stickleback	Medaka	Zebrafish	Fugu	Tetraodon
MS4A_L	GL831385.1: 234 kb	n.m.	n.m.	n.m.	n.m.	n.m.	n.m.
IQGAP3	GL831385.1: 377 kb	KB872011.1:169,421	Gr.XX:395,788	Scf.1687:3,380	Chr.16:58,627,752	Scf.270:69,544	UnR:51,175,235
TTC24	GL831385.1: 402 kb	KB872011.1:198,112	n..m	n.m.	n.m.	n.m.	n.m.
IGDCC3_L	GL831385.1: 427 kb	KB872011.1:255,693	n.m.	n.m.	Chr.16:58,132,553	Scf.270:80,180	UnR:36,799,788
POLR3GLB	GL831385.1: 443 kb	KB872011.1:57,761	n.m.	n.m.	Chr.16:45,667,787	n.m.	UnR:36,807,295
ON9 (L)	GL831385.1: 447 kb	n.m.	n.m.	n.m.	n.m.	n.m.	n.m.
TXNIPB	GL831385.1: 488 kb	KB872011.1:35,284	Gr.XIII:9,941,933	Scf.1409:13,291	Chr.16:45,707,109	Scf.4:580,338	Chr.12:2,657,610
BNIPL	GL831385.1: 497 kb	n.m.	n.m.	n.m.	Chr.16:40,942,507	n.m.	UnR:36,809,629
PRUNE	GL831385.1: 510 kb	KB871885.1:64,311	Gr.XX:568,369	Scf.7900:60	Chr.16:40,905,195	Scf.270:94,186	UnR:36,812,558
MYO1EB	GL831385.1: 526 kb	KB871885.1:31,531	Gr.XX:559,381	Ultra188:307,254	Chr.16:40,867,008	Scf.270:101,404	UnR:36,816,353
PI4KB_L	GL831385.1: 556 kb	KB871885.1:1,293	Gr.XX:548,331	Ultra188:292,345	Chr.16:40,803,414	Scf.270:112,495	UnR:36,831,654

Tetraodon		Zebrafish	Stickleback	Tilapia	Medaka	Fugu	Cavefish
BIRC2	Chr.7:4,453 kb	Chr.21:21,935,952	Gr.VII:21,556,358	GL831183:1,820,653	Chr.14:13,155,142	Scf.6:3,223,470	KB882129.1:1,721,322
DDX52	Chr.7:4,457 kb	n.m.	Gr.VII:21,547,796	GL831183:1,810,500	Chr.14:12,747,632	Scf.6:3,216,749	KB882275.1:1,390,053
HEATR6	Chr.7:4,464 kb	Chr.21:21,948,887	Gr.VII:21,539,332	GL831183:1,801,048	Chr.14:12,755,753	Scf.6:3,210,114	KB882275.1:1,344,201
TN1+2 (U) S8	Chr.7:4,474 kb	n.m.	n.m.	n.m.	n.m.	TR1=Scf.6:3,198 kb	
SRSF1a	Chr.7:4,480 kb	Chr.15:15,091,095	Gr.VII:21,531,881	GL831183:1,787,791	Chr.14:12,769,932	Scf.6:3,194,947	KB882118.1:2,728,532
PIGS	Chr.7:4,482 kb	Chr.21:38,418,319	Gr.VII:21,526,716	GL831183:1,780,862	Chr.14:12,776,106	Scf.6:3,191,056	KB882275.1:1,312,326
DIXDC1b	Chr.7:4,504 kb	Chr.21:24,346,357	n.m.	GL831183:2,070,154	Scf.2107:9,179	Scf.6:3,167,615	KB882184.1:1,917,207
IL2RB	UnR:6,376 kb	Chr.3:25,297,233	Gr.XI:4,997,878	GL831154:3,358,590	Chr.8:18,842,323	Scf.209:238,404	n.m.
UBE2L	UnR:6,381 kb	n.m.	Gr.XI:5,009,101	GL831154:3,375,712	Chr.8:18,822,424	Scf.3:1,365,240	KB872120.1:28,191
MPST_L	UnR:6,384 kb	Chr.3:25,268,072	Gr.XI:5,013,296	GL831154:3,383,860	Chr.8:18,800,980	Scf.209:229,825	KB872120.1:38,426
TN3-7 (P) S10	UnR:6,397 kb	n.m.	n.m.	n.m.	n.m.	TR4=Scf.209:508 kb	n.m.
unknown	UnR:6,426 kb	Chr.3:25,260,060	Gr.XI:5,057,906	n.m.	n.m.	Scf.209:218,267	KB872120.1:48,616
MIEF1	UnR:6,433 kb	Chr.3:25,237,420	Gr.XI:5,062,437	GL831154:3,431,969	Chr.8:18,750,956	Scf.209:213,625	KB872120.1:61,063
CTSD	UnR:6,437 kb	Chr.3:25,222,423	Gr.XI:5,068,126	GL831154:3,437,651	Chr.8:18,742,987	n.m.	KB872120.1:79,204
ATF4B2	UnR:6,452 kb	Chr.3:24,493,668	Gr.XI:5,079,354	GL831154:3,457,312	Chr.8:18,713,130	Scf.209:199,035	n.m.
RXRB	UnR:16,279 kb	Chr.19:7,871,577	Scf.58:629,374	GL831408:481,721	Chr.11:15,140,156	Scf.523:75,325	KB882167.1:848,860
COL11A2	UnR:16,312 kb	Chr.19:7,802,938	Scf.58:671,489	GL831408:524,645	Chr.11:15,190,534	Scf.523:29,998	KB882167.1:897,732
BRD2	UnR:16,332 kb	Chr.19:7,775,502	Scf.58:706,703	GL831408:575,195	Chr.11:15,241,334	Scf.523:14,385	KB882167.1:971,140
TAP2	UnR:16,346 kb	Chr.19:7,746,873	Scf.58:975,270	GL831408:623,238	Chr.11:15,298,761	Scf.523:289	KB882167.1:1,008,064
TN8 (U frg)	UnR:16,359 kb	DR13=Chr.19:7,667 kb	GA24=Scf.58/ GA1=Gr.X	ON19=GL831408:690 kb	OL10=Chr.11:15,359 kb	TR3= Scf.61:4 kb	n.m.
DAXX	UnR:16,421 kb	Chr.19:7,636,988	Gr.X:28,778	GL831594:63,643	Chr.11:15,459,697	Scf.61:13,157	KB882167.1:1,063,507
KIFC1	UnR:16,431 kb	Chr.19:7,613,506	Gr.X:49,650	GL831289:79,719	Chr.11:15,474,058	Scf.61:24,111	KB882167.1:1,097,257
FLOT1	UnR:16,446 kb	Chr.19:7,560,892	Gr.X:73,294	GL831289:104,075	Chr.11:15,494,061	Scf.62:41300	KB882167.1:1,134,654

HIST1H3	UnR:41,065 kb	n..m	Gr.XIX:2,304,804	GL831136:2,259,111	Chr.1:2,513,712	n.m.	n.m.
WNT11	UnR:41,166 kb	Chr.10:33,059,096	Gr.VII:13,318,639	GL831477:212,823	Chr.14:23,692,408	Scf.3079:5685	KB882272.1:1,256,317
COL21A1	UnR:41,179 kb	Chr.2:50,557,326	Scf.37:508,232	GL831314:389,795	Chr.15:23,644,417	Scf.24:1140942	n.m.
**TN9-11 (U)**	UnR:41,327 kb	n.m.	n.m.	n.m.	n.m.	n.m.	n.m.
DTD2	UnR:41,700 kb	Chr.17:31,776,302	Scf.984:3,034	GL831232:1,095,922	Chr.22:23,689,838	Scf.419:161371	KB882197.1:1,636,528
GCC1	UnR:46,396 kb	Chr.4:18,997,697	Scf.90:405,703	GL831201:1,470,641	n.m.	Scf.2243:14,867	KB882087.1:1,595,735
MCAM	UnR:46,410 kb	Chr.5:60,319,303	Gr.VII:24,162,733	GL831273:97,857	Chr.14:28,939,089	Scf.51:1,324,826	KB882089.1:2,181,105
PAQR6	UnR:46,425 kb	Chr.16:48,820,172	Gr.XX:15,642,253	GL831154:4,515,506	Chr.16:26,655,244	Scf.35:1,599,745	KB882167.1:457,298
KLHL	UnR:46,439 kb	n.m.	n.m.	n.m.	n.m.	n.m.	KB882088.1:1,213,617
**TN12 (U)**	UnR:46,457 kb	n.m.	n.m.	n.m.	n.m.	n.m.	n.m.
MAP2K1	UnR:46,504 kb	Chr.18:19,199,831	Gr.XIX:6,544,984	GL831142:8,041,452	Chr.6:11,421,673	Scf.2:1,217,717	KB871930.1:118,225
MLPH_L	UnR:46,515 kb	n.m.	n.m.	n.m.	Ultra36:67,947	n.m.	n.m.
NRXN1	UnR:46,518 kb	n.m.	n.m.	n.m.	n.m.	n.m.	KB882130.1:189,117
DNAJB12	UnR:46,532 kb	n..m	n.m.	n.m.	n.m.	n.m.	KB882130.1:1,109,048
JPH1B	UnR:59,004 kb	Chr.2:30,005,551	Gr.III:5,492,873	GL831354:455,011	n.m.	Scf.74:849,557	KB882166.1:1,172,202
BLVRA	UnR:59,015 kb	Chr.2:50,414,868	Gr.III:5,088,854	GL831354:506,112	Chr.17:5,261,686	Scf.74:840,046	KB882137.1:2,842,204
PAXIP1	UnR:59,024 kb	Chr.2:29,617,508	Gr.III:4,287,012	GL831501:191,946	Chr.17:13,348,920	Scf.74:818,166	KB882166.1:656,138
SSR2	UnR:59,037 kb	Chr.19:11,721,333	Scf.130:196,782	GL831658:84,003	Chr.11:17,243,255	Scf.69:1,221,062	KB871927.1:230,296
**TN13 (Z)** **S9**	UnR:59,040 kb	n.m.	n.m.	n.m.	n.m.	n.m.	n.m.
SACM1LA	UnR:59,045 kb	n.m.	n.m.	n.m.	n.m.	n.m.	KB871582.1:1,047,640
PCMTD2	UnR:59,059 kb	Chr.23:30,994,383	Gr.XII:18,192,150	GL831399:438,752	Chr.7:4,893,171	n.m.	KB882138.1:2,088,561
CBLC	UnR:59,077 kb	Chr.16:28,000,382	Gr.XX:662,725	GL831458:59,862	Chr.16:18,765,322	n.m.	KB882126.1:234,497
ORC4	UnR:59,149 kb	Chr.6:1,422,821	Scf.74:535,114	GL831181:2,670,810	Chr.2:6,259,653	Scf.110:100,311	KB882235.1:176,036
NEU1	UnR:66,822 kb	Chr.19:27,295,873	Gr.X:3,280,327	GL831186:78,973	Chr.11:4,453,691	Scf.435:39,107	KB871582.1:949,242
PDK2	UnR:66,875 kb	Chr.19:6,297,800	Gr.XI:2,022,609	n.m.	n.m.	Scf.253:296,663	KB872038.1:397,440
MICALL1x2	UnR:66,886 kb	n.m.	Gr.XIII:16,314,591	n.m.	n.m.	n.m.	KB882236.1:427,302
GGA1	UnR:66,888 kb	n.m.	Gr.XI:4,234,770	GL831154:2,349,927	Chr.8:19,705,962	Scf.253:309,946	n.m.
**TN14 (U)**	UnR:66,895 kb	n.m.	n.m.	n.m.	n.m.	n.m.	n.m.
PLXNA3	UnR:66,904 kb	Chr.8:9,764,702	Scf.114:235,561	GL831175:468,927	Ultra90:355,648	Scf.54:994,853	KB882094.1:3,219,986
BCAP31	UnR:66,942 kb	Chr.8:9,518,258	Scf.114:277,266	GL831175:404,906	Ultra90:548,075	Scf.54:967,902	KB882094.1:3,014,918
PRDM15	UnR:66,965 kb	Chr.10:34,249,730	Gr.VII:13,527,772	GL831477:493,540	Chr.14:23,377,076	Scf.572:5,753	KB882272.1:425,658
MRPL44	UnR:66,990 kb	Chr.15:34,510,801	Gr.I:5,544,654	GL831147:6,497,363	Chr.13:22,450,832	Scf.144:253,959	KB872350.1:20,597

TLN1_L	UnR:74,568 kb	Chr.10:8,107,187	n.m.	GL831133:670,419	n.m.	Scf.106:721,682	KB882201.1:901,742
TPM2	UnR: 74,584 kb	Chr.25:33,891,094	Gr.XIX:12,127,399	GL831142:6,919,089	Chr.6:12,491,143	Scf.106:750,168	n.m.
CRFA26	UnR: 74,588 kb	Chr.10:7,783,983	Gr.XIV:14,819,163	GL831139:9,313,843	n.m.	Scf.106:760,170	KB882201.1:702,872
MPDZ_L	UnR: 74,639 kb	n.m.	Gr.IX:218,653	GL831245:372,147	Chr.1:15,408,903	n.m.	KB871630.1:4,163,296
**TN15 (U)**	UnR: 74,651 kb	n..m	n.m.	n.m.	n.m.	n.m.	n.m.
PMPCB	UnR: 74,656 kb	Chr.4:1,620,084	Gr.IV:24,705,906	GL831452:59,274	Scf.860:66,616	Scf.390:27,432	KB871768.1:101,407
DNAJC2	UnR: 74,659 kb	Chr.4:1,630,121	Gr.IV:24,699,090	GL831452:50,819	Scf.860:59,050	Scf.390:33,500	KB871768.1:109,311
PSMC2	UnR: 74,664 kb	Chr.4:13,992,340	Gr.IV:24,694,746	GL831452:46,245	Scf.860:44,642	Scf.390:40,795	KB871646.1:601,347
SLC26A5	UnR: 74,671 kb	Chr.4:13,997,929	Gr.IV:24,682,335	GL831452:33,507	Scf.860: 29,407	Scf.390:44,283	KB871646.1:604,862
MECP2	UnR: 74,709 kb	Chr.8:815,205	Scf.141:50,816	GL83387:568,861	Ultra90:2,330,995	Scf.215:89,033	KB882094.1:2,466,717
RPL15	UnR:91,568 kb	Chr.19:19,373,861	Gr.X:12,194,476	GL831306:505,679	Chr.11:17,344,655	Scf.61:420,784	KB872083.1:189,842
NKIRAS1	UnR:91,570 kb	Chr.19:19,365,001	Gr.X:12,192,349	GL831306:501,668	n.m.	Scf.61:423,342	KB872083.1:185,257
UBE2E1x2	UnR:91,572 kb	Chr.19:19,345,469	n.m.	GL831306:483,844	Chr.11:17,349,746	n.m.	KB872083.1:116,846
SF3A3	UnR:91,599 kb	Chr.19:16,724,548	Gr.X:12,147,135	GL831306:385,243	Chr.11:17,359,826	Scf.61:463,934	KB872083.1:104,949
**TN16-25 (U) S11**	UnR:91,605 kb	n.m.	n.m.	n.m.	OL11= Chr.11:15,417 kb	TR3= Scf.61:4 kb	n.m.
MANEAL	UnR:91,649 kb	Chr.19:16,691,946	Gr.X:12,138,487	GL831306:357,580	Chr.11:17,366,902	Scf.61:467,693	KB872083.1:91,312
LIN28A	UnR:91,661 kb	Chr.19:14,873,056	Gr.X:12,126,255	GL831224:695,464	Chr.16:20,005,996	Scf.61:474,885	KB872083.1:72,571
PDIK1L	UnR:91,666 kb	Chr.19:14,917,590	Gr.X:12,115,441	GL831306:333,138	Chr.11:17,387,411	Scf.61:481,795	KB872083.1:50,157
**Fugu**		**Cavefish**	**Stickleback**	**Medaka**	**Zebrafish**	**Tilapia**	**Tetraodon**
ZC3H7B	Scf.497:3 kb	KB871619.1:696,570	Gr.XI:4,670,284	Chr.8:19,340,406	Chr.3:4,774,863	GL831154.1:2,913,191	UnR:6,131,681
TEFB	Scf.497:15 kb	KB871619.1:736,912	Gr.XI:4,665,256	Chr.8:19,365,755	Chr.3:5,651,586	GL831154.1:2,938,053	Chr.8:8,586,708
TOB2	Scf.497:27 kb	KB871619.1:768,740	Gr.XI:4,653,805	Chr.8:19,374,271	Chr.3:5,677,205	GL831154.1:2,950,297	UnR:47,391,436
POLR3H	Scf.497:34 kb	KB871619.1:800,887	Gr.XI:4,637,899	Chr.8:19,390,254	Chr.3:5,689,082	GL831154.1:2,755,237	UnR:73,290,998
CSDC2B	Scf.497:39 kb	KB871619.1:811,647	Gr.XI:4,634,138	Chr.8:19,399,583	Chr.3:5,706,665	GL831154.1:2,749,478	UnR:73,287,739
**TR6 (P)**	Scf.497: 46 kb	n.m.	n.m.	n.m.	n.m.	n.m.	n.m.
PMM1	Scf.497:55 kb	KB871619.1:821,622	Gr.XI:4,625,556	Chr.8:19,408,153	Chr.3:4,345,950	GL831154.1:2,735,399	UnR:73,284,730
GUCY2C	Scf.497:64 kb	KB871619.1:857,065	Gr.XI:4,609,050	Chr.8:19,435,999	Chr.3:4,299,002	GL831154.1:2,708,773	UnR:57,302,493
PLBD1	Scf.497:77 kb	KB872476.1:92,845	Gr.XI:4,560,383	Chr.8:19,471,884	Chr.3:4,175,405	GL831154.1:2,692,760	UnR:57,296,236
PICK1	Scf.497:82 kb	KB872476.1:109,866	Gr.XI:4,554,292	Chr.8:19,492,314	Chr.3:4,143,611	GL831154.1:2,591,295	UnR:57,291,346
**Atlantic Salmon**		**Cavefish**	**Stickleback**	**Medaka**	**Zebrafish**	**Tilapia**	**Tetraodon**
ARNT2		KB882249:1,450,404	Gr.II:160762	Scf.3434:959	Chr.7:11,383,168	GL831223:2,640,968	UnR:68,492,792
LCA S4		n.m.	n.m.	n.m.	n.m.	n.m.	n.m.
FAH	ssLCA	KB882249:1,683,761	Gr.II:197,614	Scf.1769:4,707	Chr.7:11,512,739	GL832094:17,428	UnR:68,490,023
MTHFS		KB882249:1,742,615	Gr.II:208404	n.m.	Chr.7:11,467,668	GL831275:211147	n.m.
ASB14		KB882145:4,610,238	Gr.XVII:1,367,496	Chr.5:33,665,135	Chr.11:43,664,191	GL831197:2,220,938	UnR:14,233,722
APPL1		KB882145:4,621,488	Gr.XVII:1,272,439	Chr.5:33,590,427	Chr.11:43,680,161	GL831197:2,406,622	UnR:13,898,257
LDA	Chr.12						
IL17RD		KB882145:4,655,437	Gr.XVII:1,290,873	Chr.5:33,618,533	Chr.11:43,722,036	GL831197:2,360,929	UnR:14,306,043
Fam208A		KB882145:4,862,896	n.m.	Chr.5:33,723,393	Chr.22:42,053,115	GL831197:2,290,945	n.m.

CCDC80		KB871578:4.763.670	GrXVI:17.945.381	Chr.21:13.604.386	Chr.9:35.060.460	GL831210.1:2.701.351	Chr.2:8.879.151
SLC35A5		KB875989:23.366	GrXVI:17.940.494	Chr.21:13.593.109	Chr.9:35.083.371	GL831210.1:2.720.549	Chr.2:8.884.387
F5		KB875989:2.588	n.m.	Chr.21:13.563.787	Chr.9:35.098.310	GL831210.1:2.728.799	n.m.
LIA S3	Chr.21	n.m.	n.m.	n.m.	n.m.	n.m.	n.m.
RGCC		KB882147:1.553.243	GrXVI:17.924.003	Chr.21:13.555.339	Chr.9:17.954.983	GL831210.1:2.747.305	n.m.
VWA8		KB882147:1.596.597	GrXVI:17.875.706	Chr.21:13.467.815	Chr.9:18.056.837	GL831210.1:2.777.465	Chr.2:8.901.100
DGKH		KB882147:1.739.612	GrXVI:17.838.915	Chr.21:13.369.186	Chr.9:18.302.283	GL831210.1:2.870.934	UnR:92.265.639
