# Supplementary material for: A comprehensive analysis of teleost MHC class I sequences
Source: BMC Evol Biol. 2015 Mar 6;15:32. doi: 10.1186/s12862-015-0309-1 (PMC4364491; doi:10.1186/s12862-015-0309-1)
Supplement: Additional file 6: — Text S3. Additional U lineage data. [file 12862_2015_309_MOESM6_ESM.pdf]

Additional file 6: Text S3. Additional U lineage data

Table of Contents

		Page
	Definition of U lineage	1
Text S3a	1. Alignment of deduced U lineage alpha 1 domain amino acid (aa) sequences	2
	2. Alignment of deduced U lineage alpha 2 domain aa sequences	4
	3. Alignment of deduced U lineage alpha 3 domain aa sequences	6
Text S3b	1a. Phylogenetic tree including all deduced stickleback and more cod U lineage alpha 1 domain aa sequences	8
	1b. Phylogenetic tree including more selected teleost U lineage alpha 1 domain deduced aa sequences	9
	1c. Phylogenetic distribution of U lineage alpha 1 domains	10
	2. Phylogenetic tree of deduced U lineage alpha 2 domain aa sequences	11
	3. Phylogenetic tree of deduced U lineage alpha 3 domain aa sequences	12
Text S3c	1. Lineage distribution of salmonid UBA alpha 1 alleles	13
	2. Sequence identity within and between salmonid lineages	15
Text S3d	Exon intron structure of the stickleback gene GA20	16
Text S3e	Alignment of deduced stickleback U lineage aa sequences	21
Text S3f	Schematic exon intron structure of stickleback seven exon genes	26
Text S3g	Alignment of deduced Atlantic cod MHC class I aa sequences	27

Definition of U lineage

In the current paper we define the U lineage as the classical MHC class I sequences in bony fishes, plus the bony fish non-classical MHC class I sequences which cluster together with them upon phylogenetic tree analysis. At least two rather characteristic (although not absolutely characteristic) residues can be distinguished for the U lineage which set them apart from classical sequences in other classes of species. At position 27, shark and tetrapod classical MHC class I have a rather well conserved tyrosine which in human interacts with β2m Y63 [Saper et al. 1991, main text reference 3], but bony fish classical MHC class I molecules tend to have methionine, valine, leucine or isoleucine at this position. Bony fishes tend to have a histidine at β2m position 63 which is uncommon in cartilaginous fish and the tetrapod line (not shown), but it is unclear how far that affected the different evolution of the MHC class I position 27 residue. Also for unknown reason, at position 85 the bony fish classical MHC class I sequences have a highly conserved phenylalanine, which appears quite rare in other classes of vertebrates. In human and chicken classical MHC class I the side chain of residue Y85 connects the end of the α1 domain, underneath the closed end of the peptide binding groove, with several residues of the α2 domain (GenBank PDB accessions 3HLA and 4G42). In summary, the distribution of characteristic motifs is consistent with single lineage identity of the bony fish U lineage sequences versus classical sequences in other species, although for the individual motif residues an effect of convergent evolution forced by interactions with co-factors cannot be excluded. When discussing various MHCI at the level of primitive bony fish, our use of “U lineage” terminology is solely based on phylogenetic tree analysis and possibly does not refer to an actual pure lineage that is fully separate from some of the nonclassical S, L and P lineages.

Text S3a. Alignment of deduced U lineage amino acid sequences

Text S3a.1 Amino acid alignment of deduced U lineage alpha 1 domain sequences

```
              1        *         20        *         40        *             60        *         80        *
                    A B                                                         A  A   B  C
                  A B C            C B         B          B                 A   B  BB  C  FC  F  FF  F
HLA-A2       :GSHSMRYFFTSVSRPGRGEPRFIAVGYVDDTQFVRFDSDAASQRMEPRAPWIEQ----EGPEYWDGETRKVKAHSQTHRVDLGTLRGYYNQSEA-
Shark        : THSLRYFYNSMT-PIPGVPEFVAVGYVDDALFVHYDSD--RKQMIPRQRWIEE---SEDKQYWERETQKQLGWEQIGKVDIQTLITRTNLTG--
Sturgeon     : THSLRYFYTGTS-GMTEFPEFVAVGMVDDVQIDYYDSK--SKKDISKQQWMKD---NMEPAYWEGNTQKCLGHQQNFKANIGIAMQRFNQTG--
Paddlefish*01: SHSLRYFYTGTS-GVTEFPEFVIVGMVDDVQISHCDSK--SKQTVPKQQWMKD---NVEPGYWERNTQICLGNQQIFKVAVIDLPKRFNQTE--
Paddlefish*03: THSLRYFFTGVT-AGTGLPEFVTVGLVDDEQHVHYDSV--SKKAVARQDWMAK---SEGPEYWESETQNFAGEEQVFKVNIGTLMQRFNQTG--
sasa*0101    : THALKYFYTASS-EVPNFPEFVVVGVVDGVQMVHYDSN--SQRAVPKQDWVNK---AADPQYWERNTGIFKGSQQTFKANIDIAKQRFNQSG--  I
onmy*0301    : THSLKYFYTASS-EVPNFPEFVVVGMVDGVQMVHYDSN--SQRMVPRQDWMNKAA-ETLPQYWESQTGILKGTQQTYKASIDIVKQRFNQSG--  I
satr*0101    : THSLKYFYTASS-EVANFPEFVAVGMVDGVQMFHYDSN--SQRAVPKQDWMNKAT-ETLPQYWERETGNCKGSQQNFKANIDIVQQRFNQSG--  I
satr*0901    : THSLKYFYTASS-EVPNFPEFVAVGMVDGVQMFHYDSN--SQRAVPKQDWVNK---AADPQYWERNTGNCKGSQQSFKANIDIAKQRFNQSG--  I
poreZ54085   : THSLKYFYTGSS-QVPNFPEFVVVAMVGDVQMVHYDSN--SGKAVPKQDWMNA---AADPQYWERNTGNFLGAQQTFKANIEIAKQRFNQTG--  I
orlaUAA*0202 : THSLKYFYTASS-QVPNFPEFVSVGLVDDAPISHYDSD--TRMTIIKQDWMKD---AMDEQYLERNTANFLGSQQVYKANIEVAKQRFNQTG--  I
orlaUBA*0201 : THSLKYFYTASS-QVPTLPEFVAVGLVDDAQIDYYDSD--IRMVVPKQDWMKE---AMDGQYWKTETENRLGDQHTFKARIEILKQSFNQTG--  I
GA1          : THSLKNFYTGSS-GVPNFPEFVVVGLLDEVEISHYDSN--TRREEPRQDWMSRVTED-DPQYWKSETEILMGQQQGFKVNIETAKQRFNQTG--  I
GA20         : THSLKNFDTASS-GVPNFPEFVNVGLLDEVEMFHYDSN--TTRAEPKQDWMSRVIED-DPQYWKRQTEKSMNAQQVFKVDIGTAKRRFNQTG--  I
GA12         : THLLKFFLTGSS-GVPNFPEFVVVGLLDEVEVVHYDSD--TWRVEPRQDWMSRVRKD-LPWDWLALTQNALVAQQELKAYIEILKRRFNQTG--  I
GA17         : THSLKYFLTASS-GLPNFPEFVIVGLLDEVELFHYDSN--TRRAEVRQDWMIRVRED-DPRYLKRGTEVLMDAQQVFKVNIEIAKQRFNQTG--  I
GM1          : LHSLHYFYTGSS-GLSTFPEFVAVGMVDGVQIDYYDSN--IQRAVLKQDWMEQVIRE-DRDYLERNAGILQGAQQTYKAGVGILKQRFNQTG--  I
GM26         : LHSLHYFYTASS-GLSAFPEFVAVGMVDGVQMVHYDSN--TQRTVLKQDWMEQVTSG-DGDYLVRNTGKFQGAQQVFKANIGIAKQRFNQTG--  I
GM35         : IHSLQVFHTASS-GLSTFPGYVMVVMVDEVQVEYYDSN--TQRIITKQDWIDQFYRD-PPGELEIATERRKGNQQTFKANIGTAKQRFNQTG--  I
GM54         : IHSQHFFFTASS-GLSTFPEYVDVQMVDEVQIGYYDSN--TQRSIPKQDWADQANRDKDPDYLEKDTENRKGLQQAFKANMGILKQRFNQTG--  I
sasa*0901    : THSLRYFYTATT-GIPDFPEFVDVGVVNGKVISYYDSI--IKRKVPKQSWMEE---NLNQQYWNQGTDQLKGTEQSFKANIQVAQTRFNQTG--  II
sasaUGA      : IHSLRYFYTSSS-GISDFPEFVDMGMVNDQVISHYDSI--TKRKVPKQSWMGK---VFDQQYWDSTTEDLRGAEKVFKNNLQTAQKRFNQTG--  II
onmy*0901    : THSLRYFYTATT-GIPDFPEFVDVGVVNGKVISYYDSI--TKRKVPKQSWMEE---NLNQQYWNQGTDQLKGAEQTFKANIQIVQTRFNQTG--  II
satr*2601    : THSLRYFYTATT-GIPDFPEFVDVGVVNGKVISYYDSI--TKRKVPKQSWMEE---NLDQQYWNQGTEILKGTEQSFKANIQVAQTRFNQTG--  II
onne*0101    : THSLRYFYTATT-GIPDFPEFVDVGVVNGKVISYYDSI--TKRKVPKQSWMEE---NFNQQYWNQGTDLLKGTEQTFKANIQVAQTRFNQTG--  II
cycaX91022_I : THTLQYYFTATT-GISNFPRLVDVGMLNGEPISMYDST--SQKKVPKQKWMAE---NLDYEYWNSATEMRKARDQVFFSNIQVLMDRFNQTA--  II
dareCN019589 : THRLQYFYTATT-GIKEFPRFVDVGMLNGEVISMYDSN--LQRKVPKQRWMAE---NLDQQYWDRYTEIVKATEQAFFNNIQVVMSRFNQTA--  II
dareEB993072 : THSLQYFYTATT-GIKEFPAFVDVGMLNGEVISMYDSN--SQRKVPKQRWIAE---NLDQQYWDSSTEGMKGVEQSFFNNIQVAMSRFNQTS--  II
```

```
                       *         20        *         40        *             60        *         80        *
                    A B                                                         A  A   B  C
                  A B C            C B         B          B                 A   B  BB  C  FC  F  FF  F
HLA-A2       :GSHSMRYFFTSVSRPGRGEPRFIAVGYVDDTQFVRFDSDAASQRMEPRAPWIEQ----EGPEYWDGETRKVKAHSQTHRVDLGTLRGYYNQSEA-   III
sasa*0801    : THSLRYVYTATS-GIPDFPEFVNLGIVDGMQIDYYDSN--TKRAVPKQDWMAK---TEGSDYWDRQTQVSIGSEQTFKANIDIVKQRFNQTG--   III
sasa*4001    : THSLRYVYTATS-GIPDFPEFVTVGLVNGEPISYHDSI--IRRETPRQDWMKE---AVDPDYWNRNTQTSIGDEQTFKANIDVAKQRFNQTG--   III
onmy*0501    : THSLRYVYTATS-GVPDFPEFVSLGIVDGMQIDYYDSN--TKRVVPKQDWMAK---TEGSDYWERQTQNSIGDEQTFKANIDVAKQRFNQTG--   III
satr*1301    : THSLRYVYTATS-GIPAFPEFVTVGLVNGEPISYYDSV--MRRETPRQDWMKE---AVDPDYWERETQISIGSEQTFKADIENIKPRFNQTG--   III
meamJF921097 : KHSLQYFYTATS-GLPNFPQFVTVGIVDGEPFSYYDSN--IRRETPRQEWMAK---SVGEDYWERNTQISIGAEQSFINNINVAKDRFNQTG--   III
orniACQ83468 : RHSLQYFYTASS-GIPSFPEFLTVGMVDGEPFSYYDCK--IGKEIPKQDWMAK---AEGPEYWDRQTQISIGAQQSFKNNIDVAKQRFNQTG--   III
--------------------------------------------------------------------------------------------------------------
sasa*3301    : THSLKYFYTGST-GIEGFPQFVAVGIVDGMHIDYFDSV--SEKNVLKQSWM------EGARDEKSITNIRKGHQQSFKANVEIVMQRFNQTT--   IV
onmy*0201    : THSLKYFYTGST-GIEGFPQFVAVGIVNGMQIDYFDGV--SEKNVLKQSWM------EGVRDEKMITNTRKGHQQTFKASVEIVMQRFNQTT--   IV
onmy*1701    : THSLKYFYTGST-GIEGFPQFVAVGIVDGMQIDYFDSV--SEKNVLKQSWM------EGVRDEKMITNTRKGNQQTFKANVEIGMQRFNQTT--   IV
satr*1001    : THSLKYFYTGST-GIEVFPQFVAVGIVDGMHIDYFDSV--SEKNVLKQSWM------EGARDEKSITNTRKGIQQSFKANVEIAMQRFNQTT--   IV
--------------------------------------------------------------------------------------------------------------
sasa*0701    : THSLKYFYTAVS-GDIDFPEFTIVGLVNNGQFVYYDSN--IKRMVPKTEWMKQ---SAGADYWDTESEKQVGQNQGFKNNIQVLKDRFNQSMST   V
sasa*1401    : THSLKYFYTAVS-GDIDFPEFTVVGLVDEGQFMYFDSN--TKTAVPKTEWMKK---SVGADYWDRQTQIGIGAHQNFKANIQVAKDRFNQSKST   V
onmy*4801    : THSLKYFYTAVS-GDIDFPEFTAVGLVDKGQFMYFDSS--TKTAVPKTEWMKR----EGADYWDRQTQGLIGAHQTFKVNIQTLKDRFNQSKST   V
satr*0801    : IHTLKNFYTAAS-GDISFPEFIIVGLVDNTPYLYFDSN--TKTAVPKTEWMKK---SVGADYWDSMTQIGIGAHQTFKANIQVAKDRFNESKST   V
onne*0201    : THSLKYFYTTVS-GDIDFPEFTIVGLVNNGQFVYYDSN--IKRMVPKTEWMKQ---SAGADYWDTESEKQVGTHQVFKNNIQVAKDRFNQSMST   V
onne*0401    : THSLKYFYTAVS-GDIDYPEFTAVGLVDNGQFMYFDSS--TKTAVPKTEWMKR----EGADYWDSQTQGLIGEHQTFKVNIQTLKDRFNQSKST   V
dareZ46776   : THSLRYFYTGVS-GDIDFPEFTVVSLVDGEQFVYFDSN--TMKTVPKTEWMRQ---NVGEDYWERETQIFTGAHPVFKNNIQVIKERFNQTQ--   V
cycaAB018581 : THSLRYFYTAVS-GISDFPEFTAVGLVDDQQFDYFDSK--TMKDVPKTEWIRQ---NVDAGYWDRQTQTLIGTHQSFKNNIQVAKERFNQTT--   V
dareUGA      : THSLRYFYTAVS-GDIDFPEFTMVGLVDGGQFIYFDSK--KMEAVPKTEWIRQ---NEGADYWDINTQRLIATHQAFKNNIQVAKERFNQSQ--   V
cycaAJ007901 : THSLRYFYTGVS-GVSGLPELTVVGLVDDQQFMYFDSN--TKKAVPKTEWMRQ---SEGADYWDEETQNDNNYHESFKKNIQKAKERFNQST--   V
--------------------------------------------------------------------------------------------------------------
sasa*0201    : TNTLQYFYTATS-GIDNFPEFVTMGIVNGHQIDHYDSI--TKRAIQKAEWISG---AVDPDYWKTNTQIYAGTETVFVNNINVAKSRFNQTG--   VI
onmy*4901    : TNTLQYFYTATS-GIDNFPEFVTMGIVNGHQIDHYDSI--TKRAIQKAEWISG---AVDPDYWKRNTQTYAAEEPVFLNNIKIAKSRFNQTG--   VI
satr*0701    : TNTLQYFYTATS-GIDNFPEFVTVGIVNGHQIDHYDSI--TKRAIQKAEWISG---AVDPDYWKRNTQIYAGAETVFVNNINVAKSRFNQTG--   VI
dareCO814745 : THTLRYFYTATS-GIDNFPEFMTVGLVDGQQIDYYDSN--IRKAVQKAEWISG---AVDPDYWNRNTQIYSGTEAVYKNNINVARSRFNQTG---  VI
cycaX91015   : THTLQYFYTATS-GIENFPEFMTAGVVDGQQIDYYDSI--IRKAVQKAEWISG---AVDPDYWNRNTQIYAGNEPSFKENINIVKSRFNQTG--   VI
--------------------------------------------------------------------------------------------------------------
sasa*1001    : IHSWKAFLTAST-GLSDFPEFVALNLVDDELMGYFDTK--TNRFEGKQSWVEE---KLGQQYLEQQENILRSTSQSFKVNVGIAMERFNQTK--   VII
onmy*4501    : IHSWKAFLTAST-GLSDFPEFVALNLVDDELMGYFDTK--TNRFEGKQSWVEE---KLGQQYMERQENILRGTSQSFKVNVGIVMERFNQTK--   VII
onne*0301    : IHSWKAFLTATT-GLSDFPEFVALNLVDDELMGYFDTK--TNRFEGKQSWVEE---KLGQQYMEREENILRSTSQSFKVNVGIVMERFNQTK--   VII
dareZ46777   : THSWKAYYTGTT-GLTEFPEFVALNLIDDQLMGYFDSK--TNRFKSQFQWMED---NLGKEYDEQQTNILLGYPEVFKNNIKVVMERFNQTQ--   VII
Eel(4863)    : MHSWKAFYTGST-GLSESPEFVAVNIVDDEPVGYFDSR--TNSFQYRQKWMEE---HLGPEYLKQQTDILKGAIPRFKANVGIAMKRFNQSG--   VII
--------------------------------------------------------------------------------------------------------------
onmy*4701    : THSMQYIVTAVS-GLDEIPEHTEVGMVDGQQFVYYDSV--LKKIIPKTDWIEK---NVDASYWKRETDRNIATEQTFKSNVAIAMTRFNQTR--   VIII
chstJK546338 : THSMKYIVTTVS-GLDGFPEHNEVGLVDGQEFVHYDSH--LKKIIPKTDWIEK---NEGADYWERETQRNINTEQTFKANVAIAMGRFNQTG--   VIII
AM27         : THSMQYFYTGVT-PGINFPEFTSVGLVDGEPFIYYDSD--IREDIPKTDWIKK-VLDYEAGFFDRQTQIHQGSQESFKVNVQTAMQRFNQTK--   VIII
icpuAY008848 : THSLQYVYTAVT-PGINFPEFIVVGQVDGQQFGYYDSK--IRKMIPKTEWIQK-INADDADYWNTQTQILQGAQETFKVGVDTLMQRFNQTA--   VIII
icpuAF053546 : THSLQYVYTAVT-PGVNFPEFTSLGQLDGQQFTYYDSK--IKKAIPKTEWIQK-VNADDPHYWNRGTQTLQGAQETFKVDVDTLMQRFNQTA--   VIII
Eel (17750)  : SHSLKYFYTGVT-AGIDFPEFTAVGLVDDEEFSYFDSN--IPKIIPKTEWFEK---AVDEQYWHRNTQNFIGAHQTFKAGVGILMQRFNQTQ--   VIII
--------------------------------------------------------------------------------------------------------------
orniUBA1     : KHSLKYFFTETP-GAQSIPEFVGVGFIDEVQFGGWNSR--RGEEVKK-DWIKL--FEDDPQHLHQYIFECSASHHYFKDTIKTLKQRLNQTE---  IX
orniUBA2     : KHSLKFFFCQTS-GVQNIPEFVVVGLVDGVQKSYYDSN--TGRPEPKTEWMKK-LMKDDPQHLEWYTARSFHTQDLFKHYTENLRKRFNQTE---  IX
orniUAA1     : KHSLKYFVTGSS-GAPNIPELFGALMVDGIQVGYCDVS--KKILEPRQEWAKNILEKHPEQLDWYQHKCFEDQPNFFRELISSLKQQFNQSE---  IX
```

Text S3a.2 Alignment of deduced U lineage alpha 2 domain amino acid sequences

```
                        100        *         120       *         140       *          160       *         180
                       A
                       B               C C
                     C C               D E                              E        DD  A
                   F E D               E F F    FF                  F  FF     E  EE  DD  A   A   A
HLA-A2       : GSHTVQRMYGCDVGSD-WRFLRGYHQYAYDGKDYIALKEDLRSWTAADMAAQTTKHKWEAA-HVAEQLRAYLEGTCVEWLRRYLENGKETLQRT
Shark        : GIHTLQVMYGCELRDD-GS-TAGFFQYGWDGKDLISFDKEHLVWNTPVTWQVVTKNKWEQDRGLGQQRKGYLEQECIEWLKKYLTAGEREL-KP
Paddlefish*01: GVHTVQRMVGCELDDD-GT-KRGFEQHGYDGEDYIMFDKDTLTWTAASQRGFTTKVKWDPLTASNQQRKAYLEGTCIEWLKKYVQYGRETLERR
Paddlefish*03: GVHTVQRMYGCELDDD-GT-KRGFDQFGFDGKDFIIFDKDSLSWTAPVMQAVITKNKLDADRALNQQQKAYLEQICIEWLQKYVQYGKETLERR
Sturgeon     : GVHTAQTMLGCELDED-GT-KRGFWQEGYDGEDYIIFDKDTLTWTAANQRGFTTKVKWDPNTARNQYLKGYLEGTCIEWLQKYVQYGRETLERR
sasa*0101    : GVHVNQWMYGCEWDDEAGV-TEGFEQWGYDGEDFIAFDLKTKSWIAPTPQSVITKLKWDSDTAQNEHDKHYLTQTCIEWLKKYLDYGKSTLMRT  I
sasa*0201    : GVHVNQKMYGCEWDDETGV-TEGFDQDGYDGEDFLAFDLKTLTWIAPTPQAVITKLKWDSNTAQNEYRKNYLTQTCIEWLKKYLDYGKSTLMRT  I
sasa*0701    : GVHVFQVMYGCEWDDEAGA-TEGFDQYGYDGEDFLAFDLKTLKWIAPTPQAVITKLKWDSDTAQNEYRKNYYTQTCIEWLKKYVDYGKSTLMRT  I
sasa*0801    : GVHVNQNMYGCEWDDEAGV-TEGFDQYGYDGEDFLAFDLKTLKWIAPTPQSLITKLKWDNNMAQIQQDKHYLTQTCIEWLKKYLDYGKSTLMRT  I
sasa*1001    : GVHVFQNMYGCEWDDETGV-TEGFDQDGYDGEDFLAFDLKTLTWIAPTPQAVNTKHKWDSNTAYNEQEKNYLTQICIEWLKKYVDYGKSTLMRT  I
sasa*1401    : GVHVFQKMYGCEWDDEAGA-TEGLTQYGYDGEDFIAFDLKTKSWIAPTPQSLITKLKWDSDTAYNEQEKNYLTQICIEWLKKYVDYGKSTLMRT  I
onmy*101     : GVHTFQNMYGCEWDDDTGA-TEGFFQYGYDGEDFLALDLKTKKWIAPTPQAVITKHKWDSNTANEERRKHYLTQECIEWLKKYLDYGKSTLMRT  I
onmy*401     : GVHIVQRMYGCEWDDEAGV-TEGFNQYGYDGEDFIAFDLKTTKWIAPTPQAVITKLKWDSNTAYTENWNNYLTQTCIEWLKKYVDYGKSTLMRT  I
onmy*0901    : GVHTFQNMYGCEWDDDTGA-TEGFFQYGYDGEDFLALDLKTKKWIAPTPQAVITKHKWDSNTANEERRKHYLTQECIEWLKKYLDYGKSTLMRT  I
onmy*4501    : GVHIVQRMYGCEWDDETGV-TEGFNQHGYDGEDFIAFDLKTTKWIAPTPQAVITKHKWDSNTVRNEQKKNYLTQTCIEWLKKYVDYGKSTLMRT  I
onmy*4901    : GVHTNQRMYGCEWNDETGA-TGGYFQDGYDGEDFIAFDLKTKTWIAPKPQAVITKLKWDSDIAMTEQKKHYLTQECIEWLKKYLDYGKSTLMTT  I
satr*0101    : GVHVFQNMYGCEWDDEAGV-TEGFYQFGYDGKDFIAFDLKTLKWTAPTPQAVITKLKWDSNMAFNEQKKNYLTQICIEWLKKYVDYGKSTLMRT  I
satr*0701    : GVHAFQMMCGCEWDDEAGA-TGGFYQFGYDGEDFLAFDLKTLKWTAPRPQAVITKLKWDSNTANNEYRKNYLTQECIEWLKKYVDYGKSTLMRT  I
satr*0801    : GVHVNQMMCGCEWDDETGA-TGGFEQYGYDGEDFIAFHLKTLTWIAPTPQAVITKNKWDSDTAQNEYWKNYLTQECIEWLKKYVDYGKNTLMRT  I
satr*1001    : GVHVNQVMYGCEWDDEAGV-TEGFNQHGYDGEDFLAFDLKTLTWIAPKPQAVITKNKLDSDTAQNEHWKNYLTQECIEWLKKYVDYGKSTLMRT  I
GA1          : GVHIYQNMYGCEWDDETNE-VKGYYQFGYDGEDFISFDLQTERWIAPKHQAFITKQKWDHNRALIAGKKNYLTHVCPEWVKKYLNYGRSSLMRT
GA12         : GVHVMQRMVTCEWDDETNE-VKGYDQYGYDGEDFLSYDLQTEQWIAQKQQAVIIKEKLDRNRDLTAGNNDCLTPFCGRYLNIYLNYGRSSLMRT
GA17         : GVHIFQRMVGCEWDNETNE-VKGYDQFGYDGEDFISYDLQTEQCIAAKQQAVITKQKWDQDRALKAHKKNSLTHVCPESLKTLLNYGRSSLMRT
GA20         : GVHIVQLMIGCEWDDVTNE-VKGYNQYGYDGEDFISFDLQTEQWIAPKQQAVLTKQKWDHNRALKAHDKNYLTHVCPEWLKKYLNYGRSSLMRT
GM1          : GTHLYQRMYGCEWDGEDDS-TDGYNQYGYDGEDFIAFDPKTLTWVAPVRQAVPTKQKWDGLRAYNEYWKNYQTKECVDWLKKYLAYGKSTLQRT
GM26         : GAHMVQWMYGCEWDDEDNS-TDGYNQYGYDGEDFISLDLKTLTWVAPVRQAFSTKQRWDGLRAQTVRYKYYYTKECVDWLKKYLAYGKSTLQRT
GM35         : GAHIVQKMYGCEWDDEDGS-TDGYRQFGYDGEDFIAWDMKTMTWVAPVRQTVITKQRWNEERAQLQYLKNYITEDCVDWLKKYLANGKSTLQRT
GM54         : GAHIIQRMDGCEWDDEDGT-TEGYDQHGYDGEDFISLDLKTLTWVAPVRQAFSTKQRWDGLRAQTVRYKYYYTKECVDWLKKYLVYGKSTLQRT
```

```
                       100         *         120       *         140       *          160       *         180
                      A
                      B               C C
                    C C               D E                               E        DD  A
                  F E D               E F      FF                  F  FF     E  EE  DD  A   A   A
HLA-A2      : GSHTVQRMYGCDVGSD-WRFLRGYHQYAYDGKDYIALKEDLRSWTAADMAAQTTKHKWEAA-HVAEQLRAYLEGTCVEWLRRYLENGKETLQRT
Shark       : GIHTLQVMYGCELRDD-GS-TAGFFQYGWDGKDLISFDKEHLVWNTPVTWQVVTKNKWEQDRGLGQQRKGYLEQECIEWLKKYLTAGEREL-KP
sasa*4001   : GVHTVQLMYGCELGDD-GI-TRGDYQFGYDGADFLSLDKSTLTWTAANQKAVITKLKWDATGAEANFQKDYLENTCIEWLKKYVNYGKDTLERK  II
onmy*0501   : GVHTFQLMYGCELGDD-GI-TRGDFQLGYDGADFLSLDKSTLTWTAANQKAVITKLKWDATGAEANFQKNYLENTCIEWLKKYVNYGKDTLERK  II
onmy*4801   : GVHTFQLMYGCELGDD-GI-TRGDFQYGYDGADFLSLDKSTLTWTAANQKAVITKLKWDATGADANFQKNYLENTCIEWLKKYVNYGKDTLERK  II
satr*0901   : GVHTFQLMYGCELGDD-GI-TRGDFQCGYDGADFLSLDKSTLTWTAANQKAVITKLKWDATGAEANFQKNYLENTCIEWLKKYVNYGKDTLERK  II
cycaX91022  : GVHSFQLMYGCELDTD-GT-KRGYMQYGYDGQDFISLDKNTLTFTAANPQAMITKNKWEANRAEAEQWKGYLENMCIEWLQKYVGYGKDTLERK  II
dareCN019589: GVHTFQEMYGCELEDD-GS-TRGYWQYGYDGEDFLSLDKSTLTWTATKPQAIITKNKWDADNADKQFTKSYLENQCIEWLKKYVGYGKDTLERK  II
meam*0201   : GVHSFQFMYGCELDD--GT-KRGYMQYGYDGEGFLILDKSTLTWTAPNDQALITKKKWDATGVEANRAKAYLESECIEWLNKYVDYGKDTLKRK  II
dareZ46776  : GVHTFQWMYGCELYDD-GT-KRGYMQYGYDGEDFLSLDKKTLTWTASNPQAVITKVKWDSTGADANFQNNYLDNTCIEWLKKYVDYGKDTLERK  II
dareUGA     : GVHTFQVMYGCELEDD-GS-TRGYWQYGYDGEDFLSLDKSTLTWTATKPQAVITKNKWDADNADRQYTKSYLENECIEWVKKYVDYGKDTLERK  II
cycaAB018581: GVHSFQFMYGCELDTD-GT-TRGYMQYGYDGEDFLSLDKSSLTYTAANPQAVITKVKWDSTRAQANSAKAYLENTCIEWLNKYVAYGKDTLERK  II
cycaX91015  : GVHSVQQMYGCELHDD-GT-KGGYMQYGYDGEDFLSLDKSSLTWTAANPQAVITKVKWDSTRAETKSETNYLENICIEWLQKYVRYGKDTLERK  II
dareZ46777  : GVHTFQFMYGCEMDDD-GN-KQVHWQIGYDGEDFISLDKKTLTWTAANSQAMTTKVKWDSTGAEANYWKGYLENECIEWVQKYVGYGKDTLERK  II
icpuAY008848: GVHTWQMMCGCKRDDN-AT-TRGYSQYGYDGEDFISLDLKTLTYTAAKSQALITKNKWDNDPDMTVSRKNYLEKECIEWLQKYVGYGRETLERK  II
icpuAF053546: GVHTVQMMCGCERDDD-GT-TRGFLQHGYDGEDFVSLDLKTKTWIAPTPQAVITKNKWDATGA-AANQQNYLENICIEWLQKYVAYGRETLERK  II
sasa*0901   : GVHIFQYMYGCTWDDDSGV-TDGLRQYGYDGEDFLVYDMKAFTWIAPKLQAEITTRKWNNEPAQMEYLKSYITQECVEWLKKYVDYGKNTLMRT  III
sasaUGA     : GMHISQDMYGCEWDDETGL-TEGFHHIGYDGQDLLVFDLKRATWIASVPQALHSKMKWEGDPSSIESEKRYLTQDCIVWLKKYLEYGKTTLQRT  III
onmy*4701   : GVHIYQYMYGCMWDDDSGV-TEGHRQYGYDGEDFLVYNMKTFTWIAPKQQSEITQRKWNNEPAQMEYLKSYITQECIEWLKKYVDYGKSTLMRT  III
dareEB993072: GVHTYQNMYGCEWDDQTEA-TNGFFQFGYDGEDLLSLDYKEMRYISPVQQGHISAQKWNKDKGLIENDRNYLSTICIEWLQKYLQYGKSNLERT
dareAF182155: GIHTIQEMYGCEWDDETRA-TNGFYQDSYDGEDFVYLDLKEMRYISPVPQALLTLQKWNDDKAFLAQQINYLSIECIEWLQKYMQYGKSSLEKT
poreZ54085  : GIHLLQEMYGCEWDDETGE-IKGYTQFGYDGDDFSVFDLKTESWTAPVTEAVVTTHKWDNDKGLNAGWVNYLTQNCPEWLKKYVNYGRSSLMRT
orlaUBA*0201: GAHVVQNMYGCEWDDETGE-VKGYDQYGYDGEDFIALDLKSESWIAAKQQAVITKDEWDDNKAFTVGRKNYLTQICPEWLEKYVNYGSSSLMRK
orlaAB450991: GLHVYQNMYGCEWDEETGE-VNSFRQFGYDGEDFIALDVKTESYTAAKQQAEITKHKWENDKTGMSYWKNYLTQICPEWLKKYVNYGSSSLMRK
orniACQ83468: GVHIFQWMYGCEWDEETGE-GNGYQQYGYDGKDFIILDLQKETWVAPVQQAAITKNKWDSNKVEFTRYKSYVTQDCREWLKKYVNYGKSSLMRT
```

Text S3a.3 Alignment of deduced U lineage alpha 3 domain amino acid sequences

```
                      *          200       *         220        *         240       *                260       *
HLA-A2       : DAPKTHMTHHAVS-DHEATLRCWALSFYPAEITLTWQRDGEDQTQDT-ELVETRPAGDGTFQKWAAVVVP--SGQEQ-----RYTCHVQHEGLPKPLTLRW
Shark        : VAPRVFPSVNKASNIRPTELSCLVTGFYPRDIEVTLLRNGQPIT-DT-ESTGILPNHDGTYQLTRWAQIT--LDEGA-----TYSCQYDQGDKVGVEIRHW
Paddlefish*01: VPPEVTLLQRKARGSADMEVLCHVTGFFPRAVEVTWVRDGQDQLEEGVQNGEVLLNQDGTYQLRKILTVS---PEEQGRH--RYSCQVDHISFKEKQIYIW
Paddlefish*03: VPPAVTLRHKKARGSADTEVVCHVTGFYPRAVEVTWVRDGQVQLEDRVQSGEVLPNQDGTYQLRKILTVS---PEEQGRH--SYSCQVDHVSFTERQNYIW
Sturgeon     : VPPAVTLLQRKARGSADTEVLCHVTGFFPRAVEVTWVRDGRDQLEEGVQSGEVLPNQDGTYQLRKILTLS---PEEQGRH--SYSCQVDHISLDQKIVKEW
sasa*0101    : VPPSVSLLQKTPS----SPVTCHATGFYPSGVMVSWQKDGQDHHED-VEYGETLQNDDGTFQKSSHLTVT---PEEWKNN--KYQCVVQVTGVKEDFIKVL
sasa*1401    : VPPSVSLLQKTPS----SPVTCHATGFYPSGVMVSWQKDGQDHHED-VEYGETLQNDDGTFQKSSHLTVT---PEEWKNN--KYQCVVQVTGVKEDFIKVL
sasa*0801    : VPPSVSLLQKTPS----SPVTCHATGFYPSGVMVSWQKDGQDHHED-VEHGETLQNDDGTFQKSSHLTVT---PEEWKNN--KYQCVVQVTGLQEDFIKVL
sasa*0201    : VPPSVSLLQKTPS----SPVTCHATGFYPSGVMVSWQKDGQDHHED-VEHGETLQNDDGTFQKSSHLTVT---PEEWKNN--KYQCVVQVTGLQEDFIKVL
sasa*1001    : VPPSVSLLQKTPS----SPVTCHATGFYPSGVMVSWQKDGQDHHED-VEYGETLQNDDGTFQKSSHLTVT---PEEWKNN--KYQCVVQVTGLQEDFIKVL
sasa*0701    : VPPSVSLLQKTPS----SPVTCHATGFYPSGVMVSWQKDGQDHHED-VEHGETLQNDDGTFQKSSHLTVT---PEEWKNN--KYQCVVQVTGIKDDIIKVL
sasa*0901    : VPPSVSLLQKSPS----SPVTCHATGFYPSGVMVFWQKDGQDQHED-VEYGETLPNHDGTFQKSSHLTVT---PEDRKNN--KYQCVVQVTGIKDDFIKVL
sasaUGA      : VPPSVSLLQKTPS----SPVTCHATGFYPSGVMVFWQKDGQDHHED-VENGETLHNDDGTFQKRTHLKVT---SEEWKNN--KYQCVVQVTGIKEDFIKVL
onmy*401     : VPPSVSLLQKAPS----SPVTCHATGFYPRDVMVSWQKDGQDHHED-VEYGETLPNDDGTFQKSSHLTVT---PEDRKNS--KYQCVVQVKGIKKDFIEVL
onmy*4701    : VPPSVSLFQKTPS----SPVTCHATGFYPSDVMVSWQKDGQDHHED-VEYGETLPNNDGTFQKSIHLTVT---PDDRKNN--KYQCVVQVKGIMEDFIKVL
onmy*4501    : VPPLVSLFQKTSS----SLVTCHATGFYPSDVMLSWQKDGQDHHED-VEYGETLPNDDGTFQKSIHLTVT---PEDRKNS--KYQCVVQVKGIKEDFIKVL
onmy*0901    : VPPSVFLLQKTPS----SPVTCHATGFYPSDVMVSWQKDGQDHHED-VEYGETLPNDDGTFQKSIHLTMT---PEDRKNN--KYQCVVQVKGIKEDFIGVP
onmy*4901    : VPPSVTLLQKTPS----SPVTCHATGFYPSGVMVFWQKDGQDQHGD-VEHGETLPNDDGTFQKSTHLTLT---PEEWKKK--QYQCVVQVTGIKEDFIKVL
onmy*0501    : VRPSVSLLQKTPS----SPVTCHATGFYPSGVMVFWQKDGQEQHGD-VEHGEILQNDDGTFQKSTHLTVT---PEEWKNN--KYQCVVQLAGIEDDITKVL
onmy*4801    : VRPSVSLLQKTPS----SPVTCHATGFYPSGVMVFWQKDGQEQHGD-VEHGEILQNDDGTFQKSTHLTVT---PEEWKNN--KYQCVVQLAGIEDDITKVL
onmy*101     : VPPSVFLLQKTPT----SPVTCHATGFYPSDVMVSWQKDGQDHHED-VEYGETLPNDDGTFQKSIHLTMT---PEDRKNN--KYQCVVQVKGIKEDFIGVP
satr*0101    : VPPSVSLLQKTPS----SPVTCHATGFYPSDVMVSWQKDGQDHHED-VEYGETLPNDDGTFQKSIRLTVT---PEEWKNS--KYQCVVQVKGLKEDFIKVL
satr*0801    : VPPSVSLLQKTPS----SPVTCHATGFYPSDVMVSWQKDGQDHHED-VEHGETLQNDDGTFQKSSHLTVT---PEEWKNN--KYQCVVQVKGLKEDFIKVL
satr*1001    : VPPSVSLLQKTPS----SPVTCHATGFYPSDVMVSWQKDGQDHHED-VEHGETLQNDDGTFQKSSHLTVT---PEEWKNS--KYQCVVQVKGLKEDFIKVL
satr*0701    : VPPSMSLLQKNPS----SPVTCHATGFYPSDVMVSWQKDGQDHHED-VEYGETLPNDDGTFQKSSHLTVT---PEDRKNS--KYQCVVQVKGLKEDFIKVL
```

```
                       *          200        *         220        *         240        *                260       *
HLA-A2        : DAPKTHMTHHAVS-DHEATLRCWALSFYPAEITLTWQRDGEDQTQDT-ELVETRPAGDGTFQKWAAVVVP--SGQEQ-----RYTCHVQHEGLPKPLTLRW
poreZ54085    : VPPSVSLLQMTSS----SPVSCYATGFYPNRAEMLWRKDGVETHDG-VEKGEILPNNDGTFQMSVELTLS--ASEDWT----KYDCVFQLSGVDKDLVIPL
orlaUBA*0201: VPPSVSLLQKSSS----SAVSCHATGFYPDRAELLWRRDGEEIHEG-VEKGQILPNNDGTFQMSVDLQPP--SGEDMQ----RYECVFQLSGVKEDVITKL
orlaAB450991: VLPSVSLLQKSSS----SAVSCHATGFYPDRAELLWRRDGEEIHEG-VEKGQILPNNDGTFQMSVDLQPP--SGEDMQ----RYECVFQLSGVKEDVITKL
icpuAY008848: VRPEVSLFQEEES---SPEVVCHATGFFPKTVMITWQKDGEDVHED-VELRETLPNQDGSFQKRSILTVS---AEDLQKH--TYTCVIQHSSLEKEIVLNQ
icpuAF053546: VPPTASVFQEEES---SPEVVCHATGFFPKTVMITWQKDGEDVHED-VELRETLPNQDGSFQKRSILTVS---AEDLQKH--TYTCVIQHSSLGKEIVLPV
orniACQ83468: DVPKVSLLQKTSS----SPVSCHATGFYPNRAEMVWKKDGVEFHDG-VHKGEILTNNDGTFQMTVNLDVSSVKPEDWD----RYTCVFQLSGVNEDIVTRL
cycaX91022    : VSPQVSLLQKDPS----SPVMCHTTSFYPSGVTITWQKNGQDHDED-VALGELIINEDGTFQRASTLNVK---PEEWKNN--KFSCVVEHQG--KTIREIL
cycaX91015    : VSPQVSLLQKDPL----SPVTCHTTGFYPSGVTITWQKNGQDHDED-VDLGELIINEDGTFQRASTLNVK---PEEWKNN--KFSCVVEHQG--KTIREIL
cycaAB018581: VSPQVSLLQKSSS----SSVTCHATGFYPKEVTVSWQKNGQDHDED-VYLGELLPNEDGTFQKTSTITVT---PEELKKN--EFSCVVEHQG--KTIREIL
dareZ46777    : VSPQVSLLQKSSS----SPVVCHVTGFYPSGLKISWQRNGQDHDED-VELGELIPNEDGTYQRTSTLNVK---PEEWKKD--KFSCVVEHQS--KTINSIL
dareUGA       : DAPEVFMLQKDPS----SPVVCQATGFYPSNIMMTWQKNKEEHFED-VDVGATLTNADGTFQKTVTLSVK---PEEWKNNKEAYRCVVQHVGAKNDVIVTV
GA1           : ERPSVSLLQKTPS----SPVSCHATGFYPDRADLFWRKDGEELHED-VDLGEILPNHDGTFQMRVDLKLSSVPAEDWR----RYDCVFQLSGVDEDIVTKL
GA12          : ERPSVSLLQKTPS----SPVSCHATGFYPDRADLFWRKDGEQLHED-VDLGEILPNHNGTFQMRVDLKLSSVPAEDWR----RYDCVFQLSGVDEDIVTKL
GA17          : ERPSVSLLQKTPS----SPVSCHATGFYPDRAALFWRKDGEELHED-VDLGEILPNHDGTFQMRVDLKLSSVPAEDWR----RYDCVFQLSGVDEDIVTKL
GA20          : ERPSVSLLQKTPS----SPVSCHATGFYPHRAALFWRKDGEQLHED-VDLGEILPNHDGTFQMRVDLKLSSVPAEDWR----RYDCVFQLSGVDEDIVTKL
GM1           : ERPRVSLLQRSPS----SPVVCHATGFYPDRVVVFWRRDGQELHEQ-VDPGEVLPNHDGTFQVSVDFNLKAVPQEDWG----RYECVVQLKGI-EDISTRL
GM26          : ERPRVSLLQRSPS----SPVVCHATGFYPDRVVVFWRRDGQELHEQ-VDPGEVLPNHDGTFQVSVDLNLKAVPQEDWG----RYECVVQLKGI-EDISTPL
GM35          : ERPRVSLLQRSPS----SPVVCHATGFYPDRVVVFWRRDGQELHEQ-VDPGEVLPNHDGTFQVSVDLNLKAVPQEDWG----RYECVVQLKGI-EDISTPL
GM54          : DRPRVSLLQRSPS----SPVVCHATGFYPDRVVVFWTRDGQELHEQ-VDPGEVLPNHDGTFQVSVDLNLKAVPQEDWG----RYECVVQLKGI-EDISTPL
```

Manually curated amino acid alignment of selected U lineage alpha 1 domain sequences (defined through phylogenetic clustering) used to produce phylogenetic tree shown in main text Figure 3 and Text S3b and c. Numbering above the alignment refers to mature HLA-A2. Amino acids are colored according to physiochemical properties. Gaps shown with a dash are introduced to optimize the alignment using the human sequence HLA-A2 as a reference. Alpha 1 and alpha 2 domain lineages as defined by Kiryu et al.[main text reference 57] and expanded upon by Nonaka et al. [main text reference 13] are shown on the right hand side of each sequence. Conserved N-linked glycosylation sites are underlined. Strands (S) and helixes (H) for the HLA-A2 sequence are underlined in red. HLA-A2 positions known to be involved in peptide anchoring are shaded red and residues contributing to the six pockets are labelled A through F [main text references 1 and 3] are indicated above the alignment. As the leader sequence is missing in many sequences, we cannot predict the first amino acid of the alpha 1 domain and this residue is therefore only shown for HLA-A2. Species are reflected in sequence names as follows: sasa is *Salmo salar* (Atlantic salmon), onmy is *Oncorhynchus mykiss* (rainbow trout), satr is *Salmo trutta* (brown trout), pore is *Poecilia reticulata* (guppy), orla is *Oryzias latipes* (medaka), GA is *Gasterosteus aculeatus* (stickleback), GM is *Gadus morhua* (cod), onne is *Oncorhynchus nerka* (sockeye salmon), cyca is *Cyprinus carpio* (common carp), dare is *Danio rerio* (zebrafish), meam is *Megalobrama amblycephala* (Wuchang bream), chst is *Channa striata* (snakehead), icpu is *Ictalurus punctatus* (catfish), orni is *Oreochromis niloticus* (tilapia), Eel is *Anguilla japonica*, sturgeon is *Acipencer sinesis*, paddlefish is *Polyodon spatula*, and shark is *Squalus acanthias* (spiny dogfish). An asterisk in the sequence name refers to an UBA allele. Lineage specific residues are shaded yellow and unusual cysteines are shaded cyan. Sequence references are shown either in the alignment, in S3b or in legend to main text Figure 2. A solid line is used to separate sequence groups and/or species.

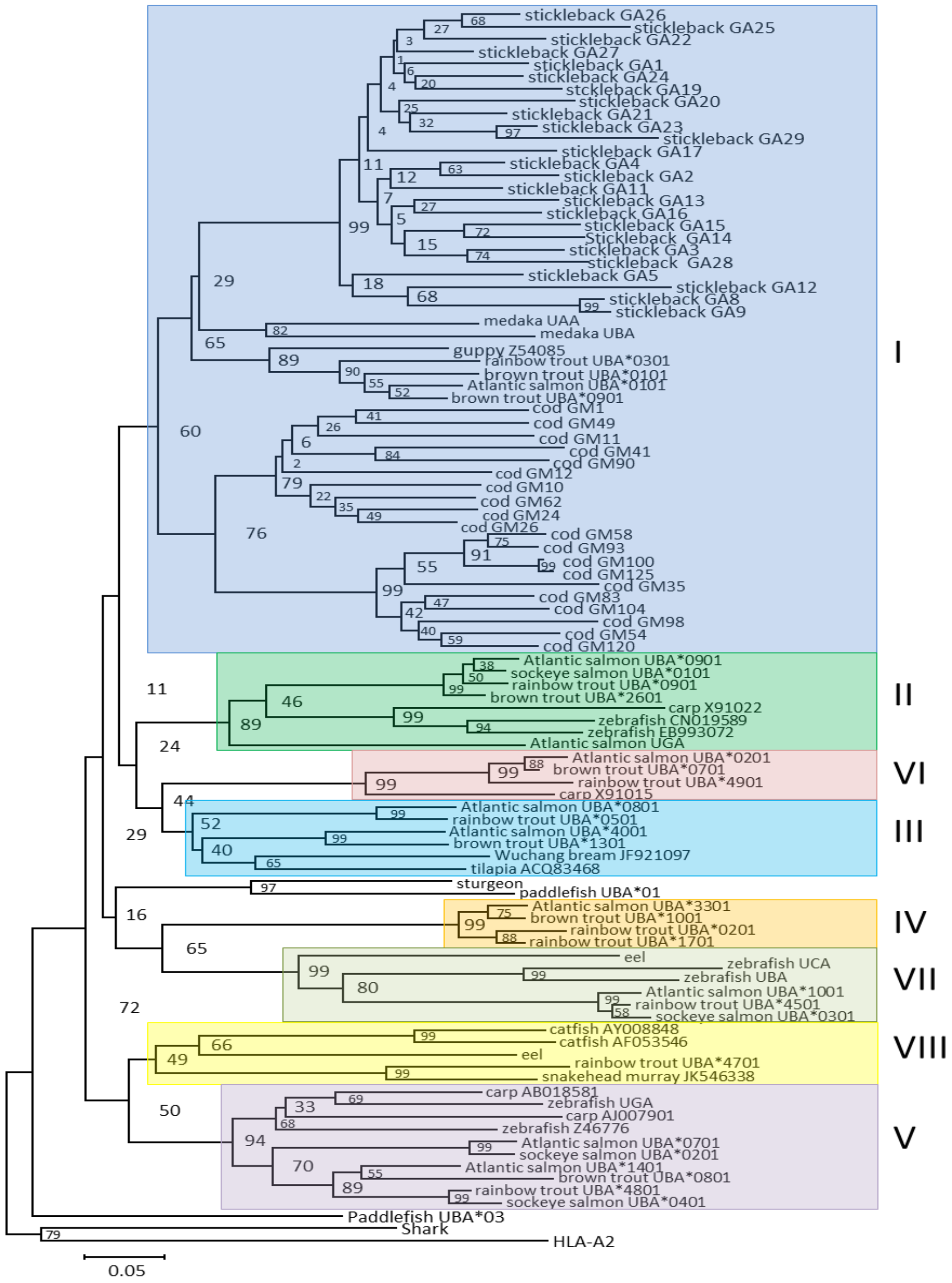

Text S3b1a. Phylogenetic tree of all deduced stickleback and more cod U lineage alpha 1 domain amino acid sequences

The tree of deduced sequences from the nine U alpha 1 domain lineages is based on hand-made alignments shown in S3a and produced using MEGA5 with Neighbor-joining P-distance and pairwise deletions (See S3b1b for details). The tree is an expansion of the tree show in main text Fig.3 including more stickleback and Atlantic cod sequences. Bootstrap values (in percentage) from 1000 trials are shown. Alpha 1 domain lineages as defined by Nonaka et al. and Kiryu et al. [main text reference 13 and 57] are shown using colored shading. The tree is rooted using the human HLA-A2 and shark sequences. Sequence names mostly reflect Latin species names where stickleback GA is *Gasterosteus aculeatus*, cod GM is *Gadus morhua*, rainbow trout is *Oncorhynchus mykiss* , Atlantic salmon is *Salmo salar*, brown trout is *Salmo trutta*, sockeye salmon is *Oncorhynchus nerka* , catfish is *Ictalurus punctatus*, tilapia is *Oreochromis niloticus*, zebrafish is *Danio rerio*, carp is *Cyprinus carpio*, guppy is *Poecilia reticulata*, Wuchang bream is *Megalobrama amblycephala*, medaka is *Oryzias latipes*, snakehead murray is *Channa striata*, eel is *Anguilla japonica* , paddlefish is *Polyodon spatula*, sturgeon is *Acipenser sinensis* and shark is *Squalus acanthias* (spiny dogfish). Sequence GenBank references are as follows: Atlantic salmon: *UBA*0101* AAN75113, *UBA*0201* AF504023, *UBA*0701* AAN75109, *UBA*0801* AAN75115, *UBA*0901* AAN75119, *UBA*1001* AAN75118, *UBA*1401* AAN75110, *UBA*4001* AEW27162, *UGA* ACX35601. Rainbow trout: *UBA*101* AF287483, *UBA*401* AF287487, *UBA*0501* AAG02508, *UBA*0901* AAG02512, *UBA*4501* AY278451, *UBA*4701* AY278449, *UBA*4801* AF318188, *UBA*4901* AF318190. Brown trout: *UBA*0101* AF296374, *UBA*0701* AF296380, *UBA*0801* AF296381, *UBA*0901* AAG02528, *UBA*1001* AF296383 and Sockeye salmon: *UBA*0101* KM085986, *UBA*0201* KM085987, *UBA*0310* KM085988 and *UBA*0401* KM085989. Medaka: *UAA*0202* AB450991, *UBA*0201* BAB83850.2. Zebrafish: *UGA* NM_200585, Paddlefish (*Polyodon spatula*): UBA*01 ACV87421 and UBA*03 ACV87423 and Atlantic cod: GM1 AGV52778.1 , GM10 AGV52769.1 , GM11 AGV52768.1 , GM12 AGV52767.1, GM24 AGV52755.1 , GM26 AGV52753.1 , GM35 AGV52744.1, GM41 AGV52738.1, GM49 AGV52730.1, GM54 AGV52725.1 , GM58 AGV52721.1 , GM62 AGV52717.1, GM83 AGV52696.1, GM90 AGV52689.1 , GM93 AGV52686.1, GM98 AGV52681.1, GM100 AGV52657.1, GM104 AGV52653.1, GM120 AGV52672.1, GM125 AGV52667.1. Stickleback: GA1-GA30 references are shown in text S2 while the Human HLA-A2 reference is AAA76608.2.

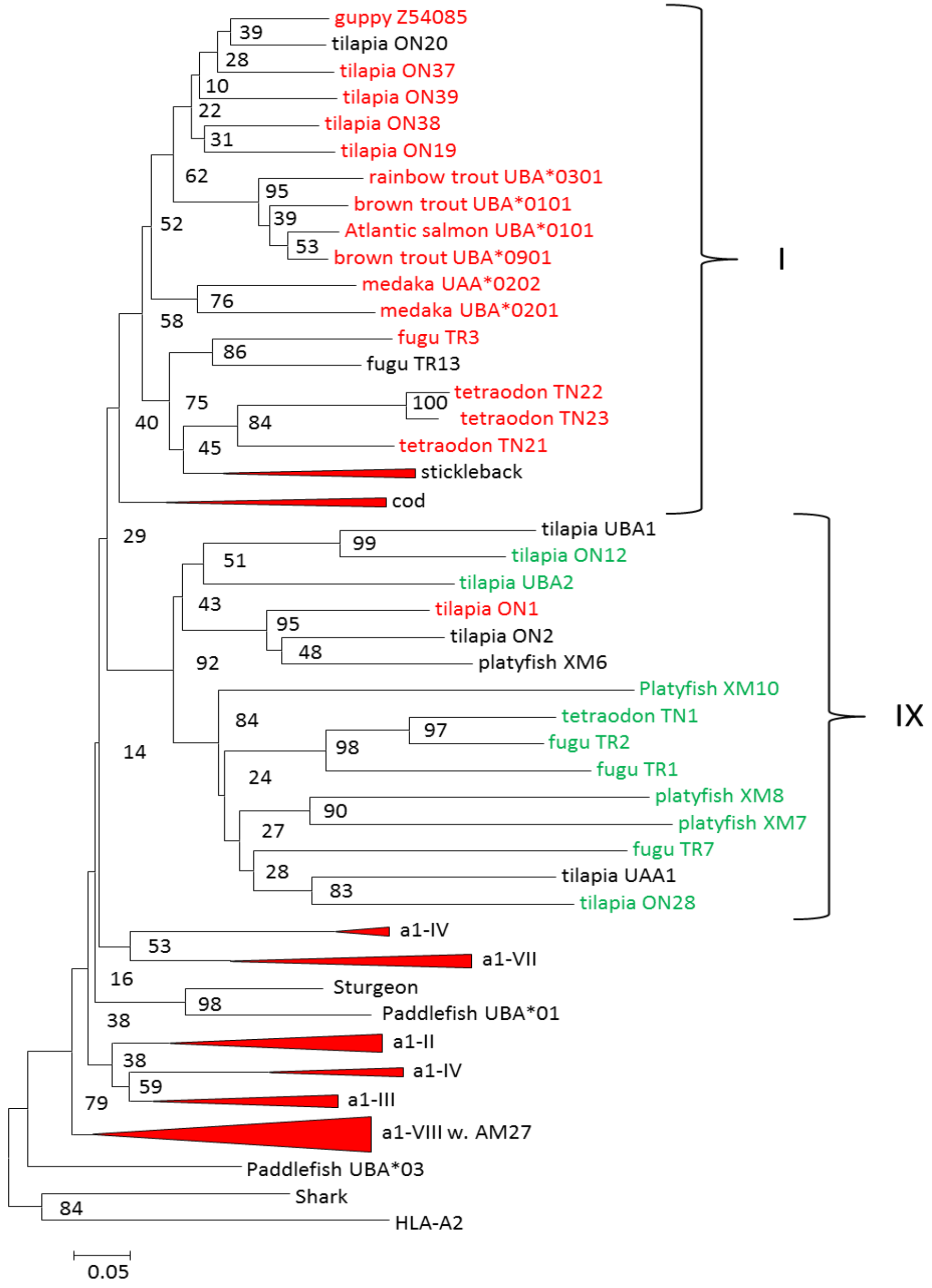

Text S3b1b. Phylogenetic tree including more selected teleost U lineage alpha 1 domain amino acid sequences

Phylogenetic tree of selected U lineage alpha 1 domain sequences identified in this study in addition to representative sequences from the eight defined lineages (see main text and figure S3b1a). The a1-II through a1-VIII clades and the stickleback and cod clades are collapsed and thus shown using a red arrow. Although crude, sequences are here defined as classical, nonclassical or unknown based on identity to the HLA-A2 peptide anchoring residue motif of YYRTKWYY as follows: Sequences complying with 6 or more of these eight residues are considered potentially classical and shown using red font, sequences with 4 or less are considered nonclassical and shown using green font. Sequences with 5 of these residues, in addition to those with incomplete alpha 2 domains are shown using black font. The majority of sequences here defined as non-classical cluster with the tilapia UBA1/2 and UAA1 sequences (Genbank AB270897) defined as a separate alpha 1 domain lineage by Nonaka et al., 2011 [main text reference 13] here denoted lineage IX.

Sequences originate from the following species: Atlantic salmon is *Salmo salar*, rainbow trout is *Oncorhynchus mykiss*, brown trout is *Salmo trutta*, guppy is *Poecilia reticulata*, tilapia is *Oreochromis niloticus*, medaka is *Oryzias latipes*, tetraodon is *Tetraodon nigroviridis*, fugu is *Takifugu rubripes*, stickleback is *Gasterosteus aculeatus*, paddlefish is *Polyodon spatula*, sturgeon is *Acipenser sinensis*, platyfish is *Xiphophorus maculatus* and shark is spiny dogfish (*Squalus acanthias*). AM27 is a cavefish (*Astyanax mexicanus*) sequence.

Sequence references are shown in main text Fig.3, in legend to S3b1a, in the figure or in Additional file 4: Text S2. The tree is based on hand-made alignments of alpha 1 domain sequences as shown in S3a and the evolutionary history was inferred using the Neighbor-Joining method [main text reference 95]. The percentages of replicate trees in which the associated taxa clustered together in the bootstrap test (1000 replicates) are shown next to the branches [96]. The tree is drawn to scale, with branch lengths in the same units as those of the evolutionary distances used to infer the phylogenetic tree. The evolutionary distances were computed using the p-distance method [97] and are in the units of the number of amino acid differences per site. All ambiguous positions were removed for each sequence pair. Evolutionary analyses were conducted in MEGA5 [98].

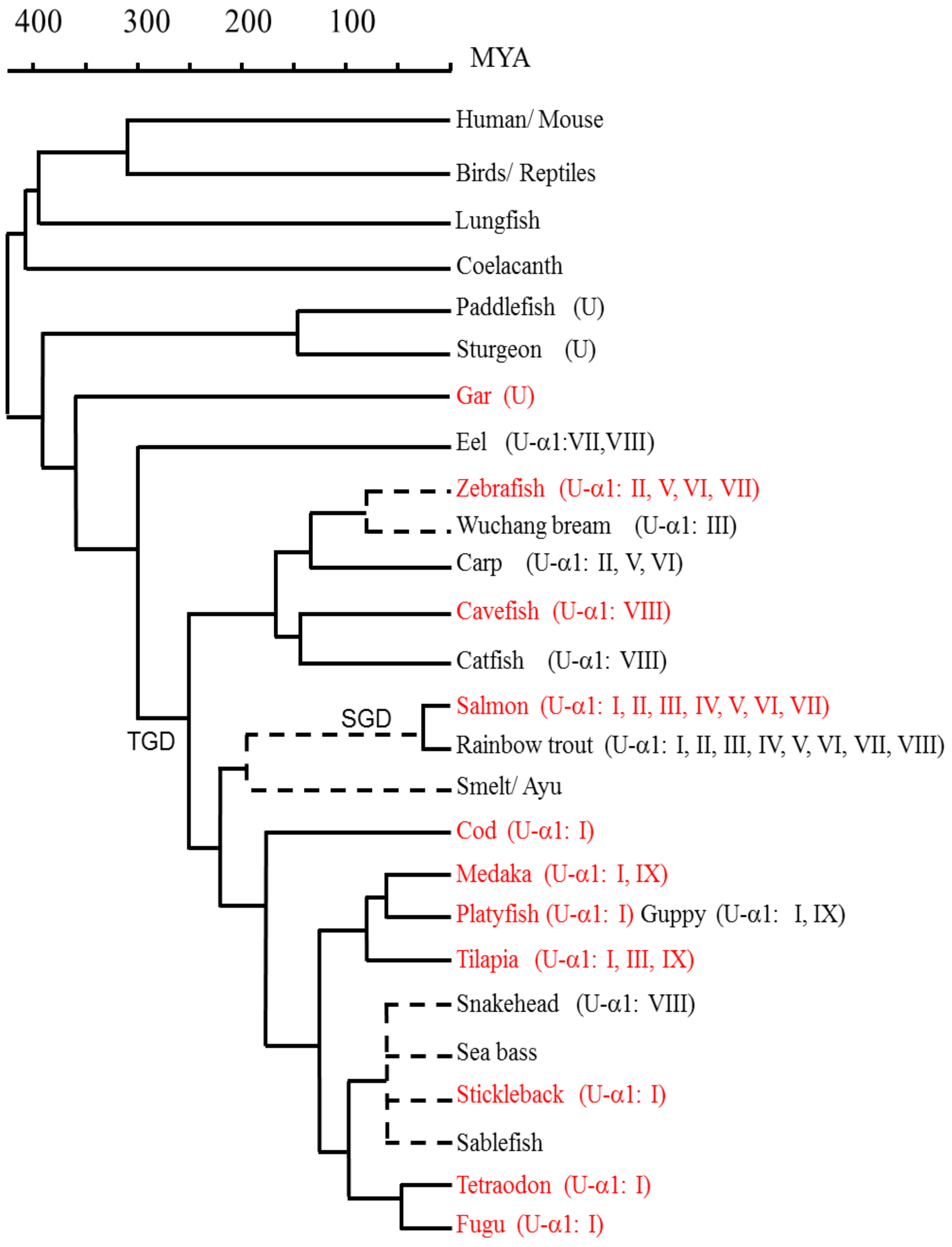

Legend SF3b.1c Phylogenetic distribution of U lineage a1 domains

Species with sequenced genomes where we have analysed presence of U lineage a1 (U-a1) domains in available genomes and transcriptomes are shown with red font. Species with limited genome and/or transcriptome resources where search and analyses have been restricted to certain lineages are shown with black font.

Lineages of alpha 1 domains as defined by Kiruy et al., 2005 [main text reference 57] and Nonaka et al., 2011[main text reference 13] are shown in parenthesis after the species name. Here we have defined the additional lineage from Nonaka et al., 2011 [main text reference 13] as lineage IX. Sequences can be found in additional file 4:Text S2.

Dotted lines relate to phylogenetic branch knots where the referenced literature was not informative on the absolute time of the event. References to phylogenies can be found in legend to main text Figure 1. In primitive bony fish our use of “U lineage” terminology does not refer to an actual pure lineage that is fully separate from some of the nonclassical lineages.

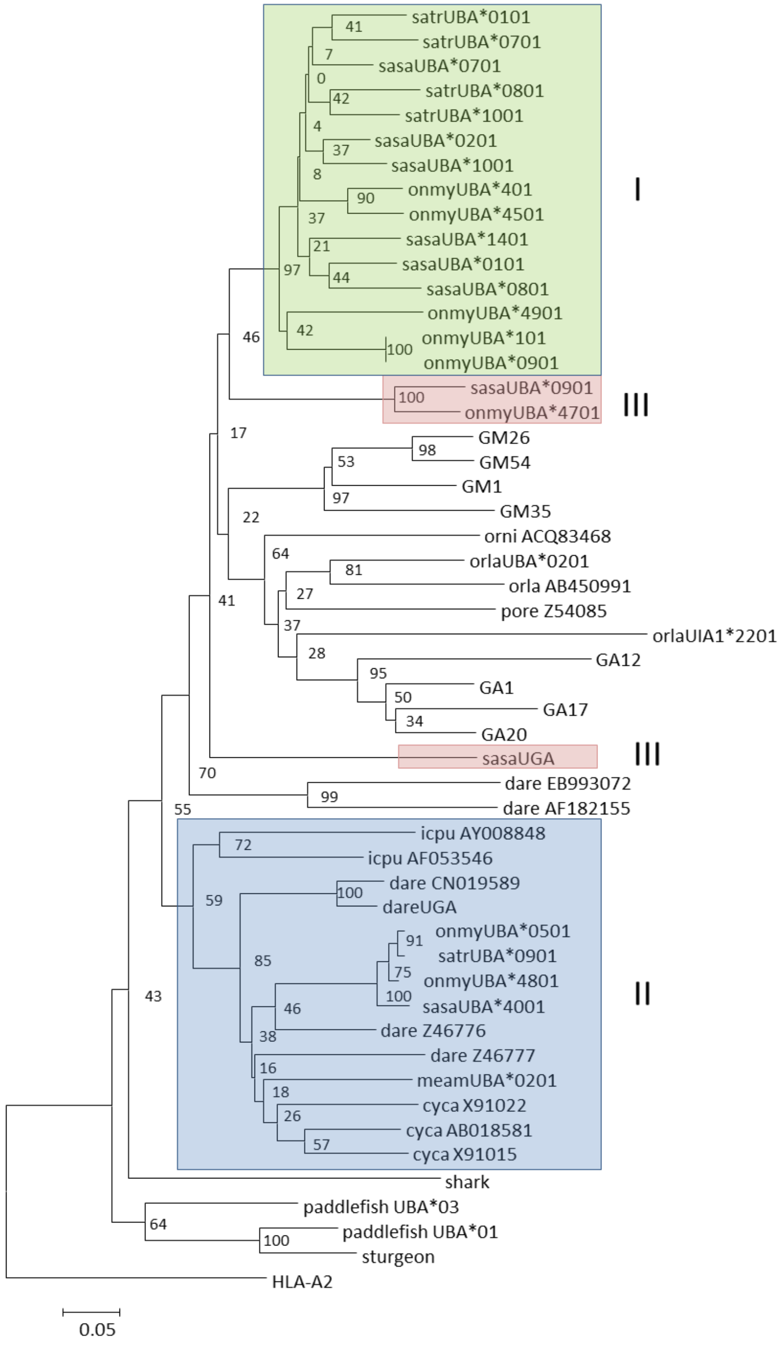

Text S3b2. Phylogenetic tree of deduced U lineage alpha 2 domain amino acid sequences

Sequence references are shown in main text Fig.3 or in legend to S3b1a. The tree is based on hand-made alignments of alpha 2 domain sequences shown in S3a and the evolutionary history was inferred using the Neighbor-Joining method [main text reference 95]. The percentages of replicate trees in which the associated taxa clustered together in the bootstrap test (1000 replicates) are shown next to the branches [96]. The tree is drawn to scale, with branch lengths in the same units as those of the evolutionary distances used to infer the phylogenetic tree. The evolutionary distances were computed using the p-distance method [97] and are in the units of the number of amino acid differences per site. All ambiguous positions were removed for each sequence pair. Evolutionary analyses were conducted in MEGA5 [98].

Alpha 2 domain lineages are shown using green, red and blue colored boxes. The tree is rooted using the human HLA-A2 sequence. Here we use proper MHC nomenclature for the sequences as follows: Satr is *Salmo trutta* (brown trout), sasa is *Salmo salar* (Atlantic salmon), onmy is *Oncorhynchus mykiss* (rainbow trout), orla is *Oryzias latipes* (medaka), orni is *Oreochromis niloticus* (Nile tilapia), pore is *Poecilia reticulata* (guppy), dare is *Danio rerio* (zebrafish), icpu is *Ictalurus punctatus* (catfish), cyca is *Cyprinus carpio* (common carp) and meam is *Megalobrama amblycephala* (Wuchang bream). Additionally GM is *Gadus morhua* (Atlantic cod) and GA is *Gasterosteus aculeatus* (stickleback).

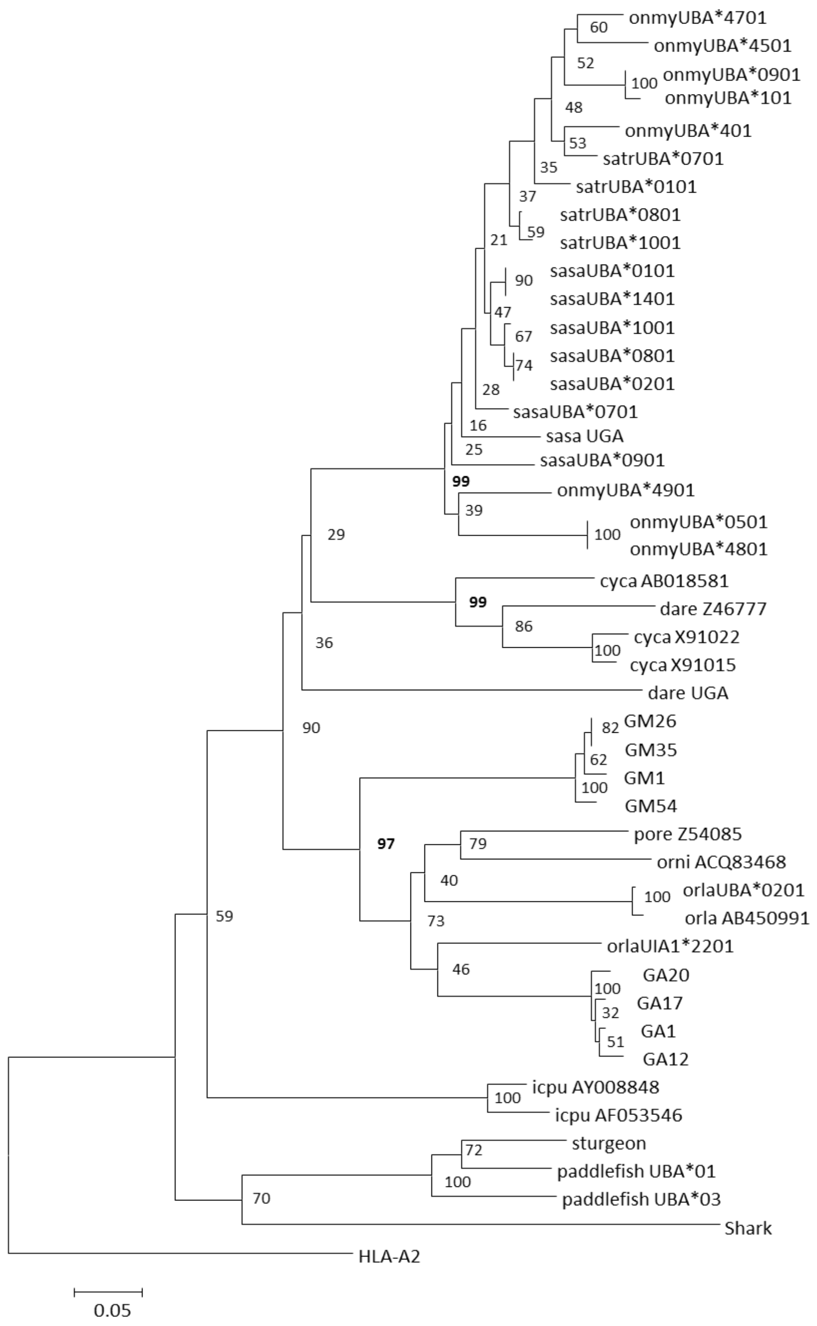

Text S3b3. Phylogenetic tree of deduced U lineage alpha 3 domain amino acid sequences

The tree is based on hand-made alignments of alpha 3 domain sequences shown in S3a and produced using MEGA5 with Neighbor Joining P-distance and pairwise deletions (see legend to S3b1a for details). Bootstrap values in percentage from 1000 trials are shown. The tree is rooted using the human HLA-A2 sequence. Bootstrap values showing phylogenetic clustering of sequences is highlighted in bold. Sequence Genbank accession numbers not shown in the figure, can be found in main text Fig.2, legend to Text S3b1 and in additional file 4: Text S2.

Satr is *Salmo trutta* (brown trout), sasa is *Salmo salar* (Atlantic salmon), onmy is *Oncorhynchus mykiss* (rainbow trout), orla is *Oryzias latipes* (medaka), orni is *Oreochromis niloticus* (Nile tilapia), pore is *Poecilia reticulata* (guppy), dare is *Danio rerio* (zebrafish), icpu is *Ictalurus punctatus* (catfish), cyca is *Cyprinus carpio* (common carp) and meam is *Megalobrama amblycephala* (Wuchang bream). Additionally GM is *Gadus morhua* (Atlantic cod) and GA is *Gasterosteus aculeatus* (stickleback).

Text S3c1. Lineage distribution of Salmonid alpha 1 domain alleles

	I (50)	II (8)	III (15)	IV (10)	V (27)	VI (12)	VII (5)	VIII (5)
1	sasa*0101	sasa*0901	sasa*0301	sasa*3301	sasa*0601	sasa*0201	sasa*1001	onmy*2301
2	sasa*0102	sasa*0903	sasa*0302	onmy*0201	sasa*0602	sasa*2001	onmy*2101	onmy*2501
3	sasa*0501	onmy*0901	sasa*1201	onmy*0202	sasa*0603	sasa*2002	onmy*2401	onmy*2601
4	sasa*1801	onmy*0902	sasa*3801	onmy*1701	sasa*0701	sasa*2301	onmy*4501	onmy*2602
5	sasa*1802	onmy*2901	sasa*3901	onmy*1702	sasa*0702	sasa*2401	onne*0301	onmy*4701
6	sasa*0401	satr*2601	sasa*0801	onmy*2801	sasa*1401	sasa*2402		
7	sasa*0402	satr*2801	sasa*2501	onmy*3001	sasa*1501	sasa*2403		
8	sasa*1101	onne*0101	sasa*3001	satr*1001	sasa*1601	onmy*0701		
9	sasa*1301		sasa*3101	satr*1101	sasa*1701	onmy*1901		
10	sasa*2101		sasa*3201	satr*2001	sasa*2601	onmy*4901		
11	sasa*2801		sasa*4001		sasa*2602	satr*0501		
12	sasa*3601		onmy*0501		onmy*0801	satr*0701		
13	sasa*3701		onmy*0502		onmy*1801			
14	sasa*2701		onmy*2201		onmy*1802			
15	sasa*2901		satr*1301		onmy*4801			
16	sasa*1901				onmy*3101			
17	sasa*2201				onmy*3201			
18	sasa*3501				satr*0801			
19	onmy*0101				satr*2201			
20	onmy*0102				satr*2901			
21	onmy*0103				satr*2401			
22	onmy*0104				satr*3001			
23	onmy*1101				satr*1701			
24	onmy*0301				satr*2701			
25	onmy*2701				satr*3101			
26	onmy*0401				onne*0201			
27	onmy*0402				onne*0401			
28	onmy*0601							
29	onmy*1501							
30	onmy*1502							
31	onmy*1001							
32	onmy*2001							
33	onmy*1201							
34	onmy*1301							
35	onmy*1401							
36	onmy*1601							

37	satr*0101							
38	satr*1901							
39	satr*2301							
40	satr*0201							
41	satr*0301							
42	satr*1201							
43	satr*2501							
44	satr*0401							
45	satr*0601							
46	satr*0901							
47	satr*1401							
48	satr*1601							
49	satr*1801							
50	satr*2101							

Summary of salmonid alpha 1 domain lineage distribution among 132 *UBA* alleles based upon phylogenetic analyses (data not shown). Within lineage sequences with 98% amino acid identity or higher are boxed and color shaded. Atlantic salmon and rainbow trout sequences are taken from the IPD-MHC database (http://www.ebi.ac.uk/ipd/mhc/fish/index.html) with the exception of the rainbow trout sequences: onmy*UBA*4501* AY278451, onmy*UBA*4701* AY278449, onmy*UBA*4801* AF318188, onmy*UBA*4901* AF318190 and the Atlantic salmon sequences sasa*UBA*3501* AEQ27776.1, sasa*UBA*3601* AEQ27777.1, sasa*UBA*3701* AEQ27778.1, sasa*UBA*3801* AEQ27779.1, sasa*UBA*3901* AEQ27780.1 and sasa*UBA*4001* AEW27162.1. Brown trout (satr, *Salmo trutta*) sequences are taken from O'Farrell et al. [O'Farrell B, Benzie JAH, McGinnity P, de Eyto E, Dillane E, et al. (2013) Selection and Phylogenetics of Salmonid MHC Class I: Wild Brown Trout (*Salmo trutta*) Differ from a Non-Native Introduced Strain. PLoS ONE 8(5): e63035. doi:10.1371/journal.pone.0063035] and the GenBank sequence accession numbers are: satr*UBA*0101* AAG02520.1, satr*UBA*0201* AAG02521.1, satr*UBA*0301* AAG02522.1, satr*UBA*0401* AAG02523.1, satr*UBA*0501* AAG02524.1, satr*UBA*0601* AAG02525.1, satr*UBA*0701* AAG02526.1, satr*UBA*0801* AAG02527.1, satr*UBA*0901* AAG02528.1, satr*UBA*1001* AAG02529.1, satr*UBA*1101* CAK18614.1, satr*UBA*1201* CAK18615.1, satr*UBA*1301* CAK18616.1, satr*UBA*1401* CAK18617.1, satr*UBA*1501* CAK18618.1, satr*UBA*1601* CAK18619.1, satr*UBA*1701* CAK18620.1, satr*UBA*1801* CAK18621.1, satr*UBA*1901* CAK18622.1, satr*UBA*2001* CAK18623.1, satr*UBA*2101* CAK18624.1, satr*UBA*2201* CAK18625.1, satr*UBA*2301* CAK18626.1, satr*UBA*2401* CAK18627.1, satr*UBA*2501* CAK18628.1, satr*UBA*2601* CAK18629.1, satr*UBA*2701* CAK18630.1, satr*UBA*2801* CAK18631.1, satr*UBA*2901* CAK18632.1, satr*UBA*3001* CAK18633.1, satr*UBA*3101* CAK18634.1. The Sockeye salmon (onne, *Oncorhynchus nerka*) sequences onne*UBA*0101* KM085986, onne*UBA*0201* KM085987, onne*UBA*0301* KM085988 and onne*UBA*0401* KM085989 are from an ongoing work (unpublished data).

Text S3c2. UBA alpha 1 domain lineages and sequence identity within and between salmonid species

	Total # alleles	# Salmon	# Trout	# Brown trout	Salmon-Trout	Trout-BrownT	Salmon-BrownT	Overall % ID
I	50	18<br>(6; 80-97%)	18<br>(9; 82-95 %)	14<br>(10; 75-93%)	79-97 %	70-94 %	70-94 %	70-97 %
II	7	2<br>(1)	3<br>(1)	2<br>(2, 95%)	92-94 %	90-95 %	95-97 %	90-97 %
III	15	11<br>(3; 69-86 %)	3<br>(1)	1	72-91 %	67 %	67-80 %	67-91 %
IV	10	1	6<br>(3; 93-100%)	3<br>(1)	90-93 %	88-92 %	95 %	88-95 %
V	25	11<br>(2; 73-100 %)	6<br>(3; 75-100 %)	8<br>(5; 69-100 %)	67-88 %	71-94 %	65-97 %	65-97 %
VI	12	7<br>(1)	3<br>(2; 93 %)	2<br>(2; 82 %)	89-91 %	83-93 %	78-97 %	78-97 %
VII	4	1	3<br>(1)	0	95-97 %	0	0	95-97 %
VIII	5	0	5<br>(1)	0	0	0	0	99-100 %
GA	25							56-97 %
Cod	20 selected							53-99 %

Number of alleles within each alpha 1 domain lineage for each of the three salmonid species Atlantic salmon (Salmon, *Salmo salar*), Rainbow trout (Trout, *Oncorhynchus mykiss*) and Brown trout (BrownT., *Salmo trutta*). Treating sequences with % identity of ≥98% as identical reduces the number of unique sequences here shown in parenthesis with sequence identity range for each species and each lineage. The stickleback (GA) and cod sequences are not allelic and thus shaded grey. Sequence references can be found in Text S3c.1.

Text S3d. Exon intron structure of Stickleback gene GA20 (scaffold_58:823.000-837.000)

Many of the stickleback U-lineage ESTs and predicted genes show differences in the transmembrane and cytoplasmic domains. We thus investigated the exon intron structure of stickleback genes and found that some ESTs have sequences either lacking a transmembrane domain or using one or both of the two different exons encoding the cytoplasmic domain (exons 6 and 7). Both exon 6 and exon 7 sequences are found in most ESTs, but with the exception of one EST (DW676054.1), exon 7 is not part of the translated protein due to a stop codon in exon 6. The exon 7 sequence contains a potential endosomal sorting motif similar to that found in Atlantic cod sequences, YGSS/F (see alignment text S3e + g). Adding to the complexity, exon 7 is also used in another reading frame in one EST (DN715043.1 in S3e and additional file 4: Text S2). The gene structure of GA20 is shown below including the exon 6 and exon 7 sequences and their potential translations.

Genomic DNA scaffold_58:823.000:837.000 (reverse complement) vs GA20 mRNA.

Exon 1: 961-1030 (genomic); 1-70 (mRNA)

```
961         GGACGTTAAAATGCGACTTGTCGGGGCGGAGATCTCGGTTCTGTCTCTCC
                      ||||||||||||||||||||||||||||||||||||||||
1                     ATGCGACTTGTCGGGGCGGAGATCTCGGTTCTGTCTCTCC
                       M  R  L  V  G  A  E  I  S  V  L  S  L

1001        TGATGATGAGCCTTCACGGCGCTGCAGCACGTGAGTTGAC
            ||||||||||||||||||||||||||||||
41          TGATGATGAGCCTTCACGGCGCTGCAGCAC
            L  M  M  S  L  H  G  A  A  A
```

Exon 2: 2623-2889 (genomic); 71-337 (mRNA)

```
2623        TTGGTTCCAGTGACTCACTCGCTGAAGAATTTCGACACTGCGTCCTCTGG
                      ||||||||||||||||||||||||||||||||||||||||
71                    TGACTCACTCGCTGAAGAATTTCGACACTGCGTCCTCTGG
                      L  T  H  S  L  K  N  F  D  T  A  S  S  G

2663        AGTCCCAAACTTCCCAGAGTTTGTGAATGTTGGGCTGCTGGATGAAGTTG
            ||||||||||||||||||||||||||||||||||||||||||||||||||
111         AGTCCCAAACTTCCCAGAGTTTGTGAATGTTGGGCTGCTGGATGAAGTTG
              V  P  N  F  P  E  F  V  N  V  G  L  L  D  E  V

2713        AGATGTTTCACTATGACAGTAACACCACGAGAGCAGAACCCAAACAGGAC
            ||||||||||||||||||||||||||||||||||||||||||||||||||
161         AGATGTTTCACTATGACAGTAACACCACGAGAGCAGAACCCAAACAGGAC
            E  M  F  H  Y  D  S  N  T  T  R  A  E  P  K  Q  D
```

```
2763      TGGATGAGCAGAGTCATAGAGGACGATCCTCAGTACTGGAAGAGGCAGAC
          ||||||||||||||||||||||||||||||||||||||||||||||||||
211       TGGATGAGCAGAGTCATAGAGGACGATCCTCAGTACTGGAAGAGGCAGAC
           W  M  S  R  V  I  E  D  D  P  Q  Y  W  K  R  Q  T

2813      TGAGAAGTCTATGAACGCCCAGCAGGTCTTCAAAGTCGACATTGGAACAG
          ||||||||||||||||||||||||||||||||||||||||||||||||||
261       TGAGAAGTCTATGAACGCCCAGCAGGTCTTCAAAGTCGACATTGGAACAG
            E  K  S  M  N  A  Q  Q  V  F  K  V  D  I  G  T

2863      CAAAACGACGCTTCAACCAAACTGGAGGTTTGTTTAT
          |||||||||||||||||||||||||||
311       CAAAACGACGCTTCAACCAAACTGGAG
          A  K  R  R  F  N  Q  T  G
```

Exon 3: 12023-12300 (genomic); 338-615 (mRNA)

```
12023     TCTCTCTCAGGTGTCCACATTGTCCAGTTGATGATCGGATGTGAATGGGA
                    ||||||||||||||||||||||||||||||||||||||||
338                 GTGTCCACATTGTCCAGTTGATGATCGGATGTGAATGGGA
                    G  V  H  I  V  Q  L  M  I  G  C  E  W  D

12063     TGATGTGACCAATGAGGTCAAAGGTTATAATCAGTATGGTTATGATGGAG
          ||||||||||||||||||||||||||||||||||||||||||||||||||
378       TGATGTGACCAATGAGGTCAAAGGTTATAATCAGTATGGTTATGATGGAG
            D  V  T  N  E  V  K  G  Y  N  Q  Y  G  Y  D  G

12113     AAGACTTCATATCATTTGACCTGCAGACAGAGCAATGGATCGCTCCAAAA
          ||||||||||||||||||||||||||||||||||||||||||||||||||
428       AAGACTTCATATCATTTGACCTGCAGACAGAGCAATGGATCGCTCCAAAA
          E  D  F  I  S  F  D  L  Q  T  E  Q  W  I  A  P  K

12163     CAGCAGGCTGTCCTCACCAAACAGAAGTGGGATCATAACAGAGCTCTGAA
          ||||||||||||||||||||||||||||||||||||||||||||||||||
478       CAGCAGGCTGTCCTCACCAAACAGAAGTGGGATCATAACAGAGCTCTGAA
           Q  Q  A  V  L  T  K  Q  K  W  D  H  N  R  A  L  K

12213     AGCACACGACAAGAACTACCTGACTCATGTGTGTCCTGAGTGGCTGAAGA
          ||||||||||||||||||||||||||||||||||||||||||||||||||
528       AGCACACGACAAGAACTACCTGACTCATGTGTGTCCTGAGTGGCTGAAGA
            A  H  D  K  N  Y  L  T  H  V  C  P  E  W  L  K
```

```
12263         AGTACTTGAACTACGGGAGGAGCTCTCTGATGAGAACCAGTAGGATCA
              ||||||||||||||||||||||||||||||||||||||
578           AGTACTTGAACTACGGGAGGAGCTCTCTGATGAGAACC
              K  Y  L  N  Y  G  R  S  S  L  M  R  T
```

Exon 4: 12776-13073 (genomic); 616-913 (mRNA)

```
12776         TCTGTCTCCAGAGCGTCCCTCGGTGTCTCTCCTCCAGAAGACTCCCTCCT
                        ||||||||||||||||||||||||||||||||||||||||
616                     GAGCGTCCCTCGGTGTCTCTCCTCCAGAAGACTCCCTCCT
                         E  R  P  S  V  S  L  L  Q  K  T  P  S

12816         CTCCAGTCAGCTGCCACGCTACAGGTTTCTACCCCCACAGAGCCGCCCTC
              ||||||||||||||||||||||||||||||||||||||||||||||||||
656           CTCCAGTCAGCTGCCACGCTACAGGTTTCTACCCCCACAGAGCCGCCCTC
              S  P  V  S  C  H  A  T  G  F  Y  P  H  R  A  A  L

12866         TTCTGGAGGAAAGATGGAGAGCAGCTCCATGAGGACGTGGACCTCGGAGA
              ||||||||||||||||||||||||||||||||||||||||||||||||||
706           TTCTGGAGGAAAGATGGAGAGCAGCTCCATGAGGACGTGGACCTCGGAGA
               F  W  R  K  D  G  E  Q  L  H  E  D  V  D  L  G  E

12916         GATCCTCCCCAACCACGACGGGACCTTCCAGATGAGGGTTGACCTGAAAC
              ||||||||||||||||||||||||||||||||||||||||||||||||||
756           GATCCTCCCCAACCACGACGGGACCTTCCAGATGAGGGTTGACCTGAAAC
                I  L  P  N  H  D  G  T  F  Q  M  R  V  D  L  K

12966         TGTCCTCCGTCCCTGCTGAAGACTGGAGGAGGTACGACTGTGTGTTCCAG
              ||||||||||||||||||||||||||||||||||||||||||||||||||
806           TGTCCTCCGTCCCTGCTGAAGACTGGAGGAGGTACGACTGTGTGTTCCAG
              L  S  S  V  P  A  E  D  W  R  R  Y  D  C  V  F  Q

13016         CTGTCTGGTGTGGACGAGGACATCGTCACCAAACTGGACAAGACCAGGAC
              ||||||||||||||||||||||||||||||||||||||||||||||||||
856           CTGTCTGGTGTGGACGAGGACATCGTCACCAAACTGGACAAGACCAGGAC
               L  S  G  V  D  E  D  I  V  T  K  L  D  K  T  R  T

13066         CAACACGGGTAGGTCTGA
              ||||||||
906           CAACACGG
                N  T
```

Exon 5: 13667-13774 (genomic); 914-1021 (mRNA)

```
13667      TGCTCCACAGAGAAGCCTGCTGGCTCCACCTTCATCATCATCATCATCAT
                     ||||||||||||||||||||||||||||||||||||||||
914                  AGAAGCCTGCTGGCTCCACCTTCATCATCATCATCATCAT
                     E  K  P  A  G  S  T  F  I  I  I  I  I  I

13707      CGCTGTGGCTGTTCTTGTCGTCATCATCGCTGCTGTGGTTGGATTCAAGG
           ||||||||||||||||||||||||||||||||||||||||||||||||||
954        CGCTGTGGCTGTTCTTGTCGTCATCATCGCTGCTGTGGTTGGATTCAAGG
             A  V  A  V  L  V  V  I  I  A  A  V  V  G  F  K

13757      TTTACAGAAAGAGGAAC G gtgagagaga
           ||||||||||||||||| |
1004       TTTACAGAAAGAGGAAC G
           V  Y  R  K  R  N
```

Exon 6: 14118-14185 (genomic); 1022-1089 (mRNA)

```
14118      tttatttcag CCAAATACTCTTCAGCCAAATGTCCTTCTGACAAATCCGA
                      ||||||||||||||||||||||||||||||||||||||||
1022                  CCAAATACTCTTCAGCCAAATGTCCTTCTGACAAATCCGA
                      A  K  Y  S  S  A  K  C  P  S  D  K  S  E

14158      AGAAGAGAGTCTCTCAGGGACAAACTGAACCCTAAACC
           ||||||||||||||||||||||||||||
1062       AGAAGAGAGTCTCTCAGGGACAAACTGA
             E  E  S  L  S  G  T  N  *
```

Exon 7: 14539-14627 (genomic); 1022-1110 (mRNA)

```
14539       ctctctttag GTTTATGGGTGAATGTTGCTCTGGATCAACAAGCGATGCT
                       ||||||||||||||||||||||||||||||||||||||||
1022                   GTTTATGGGTGAATGTTGCTCTGGATCAACAAGCGATGCT
                       G  L  W  V  N  V  A  L  D  Q  Q  A  M  L
                         F  M  G  E  C  C  S  G  S  T  S  D  A

14579       CCACATTCTTCTCCACAAACTGTCAATTTATGGCTCATCTTTCATTTAAACGTCTGAAT
            ||||||||||||||||||||||||||||||||||||||||||||||||||
1062        CCACATTCTTCTCCACAAACTGTCAATTTATGGCTCATCTTTCATTTAA
              H  I  L  L  H  K  L  S  I  Y  G  S  S  F  I  *   (DW676054.1, see Text S3e)
             P  H  S  S                                   (e.g. DN715043.1, see Text S3e)
```

Text S3e. Alignment of deduced stickleback U lineage amino acid sequences

Stickleback U lineage sequences with ORF error or one domain only are not included i.e. GA3, GA7 and GA29. Dots indicate identity while dashes indicate gaps apart from the 3' region where all residues are shown. Numbering according to the mature HLA-A2 sequence is shown below the alignment while amino acids in HLA-A2 peptide anchoring positions are indicated above the alignment and with red shading in the HLA-A2 sequence. Star defines stop codon. Potential endosomal sorting motif in stickleback exon 7 is shaded yellow. Abbreviations are as follows: CP is connecting peptide, TM is transmembrane region and CYT is cytoplasmic domain. The GenBank sequence references for human HLA-A2 is AAA76608.2 while sequences with references for the remaining sequences can be found in Additional file 4: Text S2.

```
             Exon 1 (Leader sequence) Exon 2 (Alpha 1 domain)
                                            Y7                                                     Y59
GA1        : MRLVGAEISVLSLLMMSLHGAAAL--THSLKNFYTGSS-GVPNFPEFVVVGLLDEVEISHYDSN--TRREEPRQDWMSRVTEDDPQYWKS :   85
GA2        : -----...................PV.....Y.L.A..-.L.......G........FV....D--.K.A.........L.........R :   82
GA4        : ........................--.....Y.L.A..-.........I........F.....D--.K.A................F..R :   85
GA9        : -----------------------V--.....Y......-.L.......I...V....VV....D--.W.L......V.......HWF.NW :   62
GA12       : ........................--..L..F.L....-..................VV....D--.W.V..........RK.L.WD.LA :   85
GA14       : --------------------------.....Y......-...................V...G.--...T.........LR.....F.DI :   61
GA17       : ........................--.....Y.L.A..-.L.......I........LF.....--...A.V.....I..R....R.L.R :   85
GA20       : ........................--.......D.A..-.........N........MF.....--.T.A..K.......I........R :   85
GA22       : --------------------------...M.Y......-.........S...V....FV.....--...A................F..R :   61
GA24       : ........................--...M.F...A..-.L.......I........MF.....--...T.................... :   85
GA_UAA     : ........................--.....Y.L.A..-.L.......I........LF...GD--...A.V.....I..RG...R.L.R :   85
GA10       : .......................------------------------------------------------------------------- :   23
GA13       : ...........A...I........--.....FVL.A..-..................VV...GD--...A..........R.....F... :   85
GA23       : ........................--...M.Y.L.A..-.........I........MF.....--...A.................... :   85
GA26       : -----------------------V--...M.Y...A..-.........A........F......--...L...................R :   62
GA_DW035296: ------------------------------------------------------------------------.......L.........R :   18
GA_DN658147: --------------------------------------------------------------------.K..........I........R :   22
GA27       : ........................--.....Y...A..-.........I........MF.....--...A...................R :   85
GA_DW039424: ---------...............--.....Y......-.........S........FV.....--...A................F... :   76
GA8        : -----------------------V--.....Y......-.........I...V....VV....D--.W.L......V.......HWF.NW :   62
GA11       : -------------------------V.....F.L.A..-.L................VV....D--...A...................R :   62
GA_UBA     : ........................--...M.Y.F....-.........A..R.....VV...G.--...A.......IK.ID........ :   85
GA_UAC     : ........................--.....Y.L.A..-.L.......A.........V.....--...G.......I............ :   85
GA5        : -----...................--.....F...A..-.........A........VV....D--...L....N.V..LRG.......R :   80
GA30       : ---------------------------------------------------------------------------------------..W :    3
GA16       : -----------------------V--...M.Y.L.A..-..................VV...G.--.T.A.........LK......... :   62
GA19       : ...........A............--.....F......-.L.......A.........D....D--...A..........I......... :   85
GA15       : ...............I........--.....Y......-...................V...G.--...T.......IK.ID........ :   85
GA21       : ........................--...M.Y......-.........I........MF.....--...A...................R :   85
GA25       : ...........A............--...M.Y...A..-.........A........F......--...T.......I.L.........R :   85
GA28       : ........................--.....Y.F.A..-.........A.W......FV...GD--...L.......I..ID....F..R :   85
HLA-A2     : -------------------------GS..MRY.F.SV.RPGRGE.R.IA..YV.DTQFVRF..DAASQ.M...AP.IEQ---EG.E..DG :   62
                                      1       *         20        *          40        *            60
```

```
                                   Exon 3 (Alpha 2 domain)
                          Y/R84                                                       T  KW147
GA1        : ETEILMGQQQGFKVNIETAKQRFNQTG-GVHIYQNMYGCEWDDETNE-VKGYYQFGYDGEDFISFDLQTERWIAPKHQAFITKQKWDHNR : 173
GA2        : N..LA.DT..VY.RH..ILN.S.....-....N.K.V..........-....D.........L...PE.....TA.Q..VRI.....QD. : 170
GA4        : N..LA.DA..VY.GH..I.........A......R.V..........-....D.................QY..A.Q..VRI........ : 174
GA9        : Q.GLA.NA.RE..GY.K..........-....V..............-....D.Y..N.............Y..A.Q..S.I....NQ.. : 150
GA12       : L.QNALVA..EL.AY..IL.R......-...VM.R.VT.........-....D.Y.......L.Y.....Q...Q.Q..V.I.E.L.R.. : 173
GA14       : Q.D.A.DA..D..GHT.....S.....-....F.R.A..........-....D...........Y.....Q...A.P..VL......QD. : 149
GA17       : G..V..DA..V......I.........-....F.R.V.....N....-....D...........Y.....QC..A.Q..V.......QD. : 173
GA6        : -------------------------------------..........-F...A.YS.N............Q...A.Q..VRI.....QD. :  52
GA20       : Q..KS.NA..V...D.G...R......-....V.L.I......V...-....N.Y...............Q.....Q..VL......... : 173
GA_DW664617: ----------------...........-...MX.Y..........D.-....H.Y.........S.....QYT.A.Q..V.......QD. :  72
GA22       : ...NF..H..V..A.............-....N.W.V.....N....-............................Q..V...R.L.Q.. : 149
GA24       : Q...A.....V..GH..I.........A...................-....N.D................................... : 174
GA_UAA     : G..V..DA..V......I.........-....F.R.V.....N....-F...D...........Y.....QC..A.Q..V.......QD. : 173
GA_DW676150: -------------------------------------..........-....D.Y..............K...PA.Q..VL......QD. :  52
GA10       : ----------------------------...VM.R.VT.........-....D.Y.......L.Y.....Q...Q.Q..V.I.E.L.R.. :  84
GA13       : G..LA.DA..V..GY......S.H...-......R.A..........-D...I.D...............QY..A.Q..V...R...... : 173
GA23       : ...TF..T..VY..D...V.R......-....V.F............-....A.D...............QY....Q..V.......Q.. : 173
GA26       : Y..NF..A..VY.G...L.........A....N.R.V..........-....D.D...............Q.....Q..V...R...... : 151
GA_DW035296: N..NA..H..D..GY...V........-......W.V........G.-....D.D.........Y.....Q...A.Q..V...R.L.... : 106
GA_DN656534: ------------------------...-....V.L.F......V...-......................Q.....Q..V......EQ.. :  64
GA_DN658147: Y......T..V..AG...L........-....V.R............-......D...............QY....Q..V......NQ.. : 110
GA_DN715043: ----------------.....S.H...-......R.A......V.D.-....N.D............E.GQL..A.Q..VRI.....Q.. :  72
GA27       : Y......S..VY.G.............--------------------------------------------------------------- : 112
GA_DW039424: Q..LA.DT..D..GH......S.H...-......R............-....D.Y..............K...PA.Q..V.....L..DS : 164
GA8        : Q.GLA.NA.RE..GY..I.........-....V..............-....D.Y..N............Q...A.QE.S.I.....Q.. : 150
GA11       : N...N.DT..D..A...I...S.....A....V.R.I....Y.....-.N..D.Y...............Q...A.QE.S.I....NQ.. : 151
GA_UBA     : R..LA.DT..A..GY..I...S.....-......R.A.....N....-....I.D..N...........D....A.Q..VRIQ.ML.... : 173
GA_UAC     : Q.....DD..E..AS..I.........-....I............RA-....D.Y........A......QY....Q..VRI.....QD. : 173
GA5        : N.KNS.DA..V..G...L.........A....V..........V.D.-....D.Y..N............QY..A.QE.S.I....NQ.. : 169
GA30       : Y......N..V..A...L.........A....F.R............-......................Q...A.Q..V......NQ.. :  92
GA16       : R..LA.YT..D..G.............A......R.A.....N..D.-.QR.I.D..............K....A.Q..V.IQ...NQD. : 151
GA19       : Q...A.AI..VY.G...L.........-..............N...K-....I.................Q...A.QK.V.......Q.. : 173
GA15       : Q.D.A.DT..A..GY..I...S.....-....F.R.A......V...-....D.Y..N...........D....Q.Q.........EQ.S : 173
GA21       : Q..KS.DT..VY..D..IL........-....V.L.I......V...-....N.Y...............Q.....Q..VL.......D. : 173
GA25       : S...F..N..V..GTLKQQNNASTKLE--------------------------------------------------------------- : 112
GA28       : N..LY..C..D..GY..I...S.....GLFM----------------------------------------------------------- : 116
GA7        : ----------------------------..............E...K-......Y...............Q...A.Q..VL...E..QD. :  61
HLA-A2     : ..RKVKAHS.THR.DLG.LRGYY..SEA.S.TV.R....DVGSD-WRFLR..H.YA...K.Y.ALKEDLRS.T.ADMA.QT..H..EAA- : 150
                         *       80        *       100       *       120       *       140      *
```

```
                                            Exon 4 (Alpha 3 domain)
                     Y159        Y171
GA1        : ALIAGKKNYLTHVCPEWVKKYLNYGRSSLMRTERPSVSLLQKTPS---SPVSCHATGFYPDRADLFWRKDGEELHEDVDLGEILPNHDGT : 260
GA2        : .RT.HR.FV..QE....L....S......................---.............I............................ : 257
GA4        : ..K.QN...........L..H........................---...............A.......................... : 261
GA9        : ........F...E...GL..F.S......................---............H..A.......................... : 237
GA12       : D.T..NNDC..PF.GRYLNI.........................---........................Q..............N.. : 260
GA14       : .D..WR..F...F....L.MF.S......................---...............N........Q................. : 236
GA17       : ..K.H...S.......SL.TL........................---...............A.......................... : 260
GA6        : .D...R.FV..QT.R..L....S......................---...............A..........Y............... : 139
GA20       : ..K.HD...........L...........................---............H..A........Q................. : 260
GA_DW664617: .VT.HW..........GL..L.S......................---...............S........Q................. : 159
GA22       : .K..ED..........GL...........................---........................Q................. : 236
GA24       : .....R.......................................---...............A.......................... : 261
GA_UAA     : ..K.H...S.......SL.TL........................---.......................................... : 260
GA_DW676150: .E..HR..F........L..F.S......................---...............A........Q................. : 139
GA10       : D.T..NNG...DF.GRYLNI.........................---........................Q................. : 171
GA13       : .K..HW..F.......GL..F.S......................---............H..A........Q................. : 260
GA23       : .W..................H.S......................---.......................................... : 260
GA26       : .Q..QN......E...GL..................M........---............H..A.......................... : 238
GA_DW035296: .R...........................................---............H..A.......................... : 193
GA_DN656534: .....E..S......Q.L....S......................---.......................................... : 151
GA_DN658147: ....QN..F......G.L....S......................---...............A.......................... : 197
GA_DN715043: .VK..YN....R..A.GL..F........................---...............A........R.......E......... : 159
GA27       : --------------------------------.............---...............A.......................... : 167
GA_DW039424: .DK.YR..F........L...........................---...............A........Q................. : 251
GA8        : ........F.......GL..F.S......................---...............A.......................... : 237
GA11       : ........F...E...GL..F.S..............F.......---...............A.......................... : 238
GA_UBA     : .FK.QN..F.......GL..F.S......................---.......................................... : 260
GA_UAC     : .W..YN.....YL.........I......................---............H..A.V........................ : 260
GA5        : ........F.......GL..F........................---.......................................... : 256
GA30       : .E.LYN......E....L..F........................---............H..A........Q................. : 179
GA16       : DK.EYW..F..DE.A.GL.TF...S.T..L...............---...............A.......................... : 238
GA19       : .DK.HW...........L...........................---...............S......E................... : 260
GA15       : ..K.H...F.......SL...........................---...............A........Q................. : 260
GA21       : ..K.HD...........L..F........................---............H..A........Q................. : 260
GA25       : ------------------------------------------------------------------------------------------ :   -
GA28       : ------------------------------------------------------------------------------------------ :   -
GA7        : VWT.QN.....YE...SL.TL.K.........---------------------------------------------------------- :  93
HLA-A2     : HVAEQLRA..EGT.V..LRR..EN.KET.Q..DA.KTHMTHHAV.DHEATLR.W.LS...AEIT.T.QR...DQTQ.TE.V.TR.AG... : 240
                      160       *         180       *         200       *         220       *         240
```

```
                                                                Exon 5 (CP and TM)
GA1        : FQMRVDLKLSSVPAEDWRRYDCVFQLSGVDEDIVTKLDKTRTNR----------EKPAAST-----F--IIIIIIAVAVLVVIIAAVVGF : 333
GA2        : FQMRVDLKLSSVPAEDWRRYDCVFQLSGVDEDIVTKLDKTRTNR----------EKPAGST---------------VAVLVVIIAAVVGF : 322
GA4        : FQMRVDLKLSSVPAEDWRRYDCVFQLSGVDEDIVTKLDKTRTNT----------EKPAGST-----F--IIIIIIAVAVLVVIIAAVVGF : 334
GA9        : FQMRVDLKLSSVPAEDWRRYDCVFQLSGVDEDIVTKLDKTRTNR----------EKPADST-----F-IIIIIIIAVAVLVVIIAAVVGF : 311
GA12       : FQMRVDLKLSSVPAEDWRRYDCVFQLSGVDEDIVTKLDKIRTNT----------EKPAASTSTS-II-IIIIIIIAVAVLVAIIAAVVGF : 338
GA14       : FQMRVDLNLSSVPAEDWRRYDCVFHLSGVDEDIVTKLDKTRTNT----------EKPAGSTSTS-TFIIIIIIIIIAAVVVLVVIAVRRF : 315
GA17       : FQMRVDLKLSSVPAEDWRRYDCVFQLSGVDEDIVTKLNKTRTNT----------EKPAGST-----FIIIIIIIIAVAVLVVIIVAVVGF : 335
GA6        : FQMRVDLNLSSVPAEDWRRYDCVFQLSGVDEDIVTKLDKTRTNT----------EKPADST-----F---IIIIIAVAVLVVIIAAVVGF : 211
GA20       : FQMRVDLKLSSVPAEDWRRYDCVFQLSGVDEDIVTKLDKTRTNT----------EKPAGST-----F--IIIIIIAVAVLVVIIAAVVGF : 333
GA_DW664617: FQMRVDLKLSSVPAEDWRRYDCVFQLSGVDEDIVTKLDKTRTNR----------EKPADST-----F--IIIIIIAVAVLVVIIAAVVGF : 232
GA22       : FQMRVDLKLSSVPAEDWRRYDCVFQLSGVDEDIVTKLDKTRTNR----------EKPAGST-----F--IIIIIIAVAVPAAIIAAVVGF : 309
GA24       : FQMRVDLELSSVPAEDWRRYDCVFQLSGVDEDIVTKLDKTRTNR----------EKPAAST-----F----IIIIAVAVLVAIIAAVVGF : 332
GA_UAA     : FQMRVDLNLSSVPAEDWRRYDCVFQLSGVDEDIVTKLDKTRTNR----------EKPAGST-----F--IIIIIIAVAVLVVIIVAVVGF : 333
GA_DW676150: FQMRVDLKLSSVPAEDWRRYDCVFQLSGVDEDIVTKLDKTRTNT----------EKPAGST-----F---IIIIIAVAVLVVIIAAVVGF : 211
GA_DW676054: -----------------RRYDCVFQLSGVDEDIVTKLDKTRTNT----------EKPADST-----F---IIIIIAVAVLVVIIAAVVGF :  55
GA10       : FQMRVDLKLSSVPAEDWRRYDCVFQLSGVDEDIVTKLDKTRTNTGRSETRSGEGEKPAGST-----F-IIIIIIIAVAVLVVIIAAVVGF : 255
GA13       : FQMRVDLNLSSVPAEDWRRYDCVFQLSGMDEDIVTKLDKTRTNT----------EKPAGST-----F---IIIIIAVAVLVVIIAAVVGF : 332
GA23       : FQMRVDLKLSSVPAEDWRRYDCVFQLSGVDEDIVTKLDKTRTNR----------EKPAAST-----F----IIIIAVAVLVVIIAAVVGF : 331
GA26       : FQMRVDLNLSSVPAEDWRRYDCVFQLSGVDEDIVTKLDKTRTNR----------EKPAGST-----F---IIIIIAVAVLVVIIAAVVGF : 310
GA_DW035296: FQMRVDLKLSSVPAEDWRRYDCVFQLSGVDEDIVTKLDKTRTNT----------EKPADST-----F---IIIIIAVAVLVVIIAAVVGF : 265
GA_DN656534: FQMRVDLKLSSVPAEDWRRYDCVFQLSGVDEDIVTKLDKTRTNT----------EKPAGSTSTS-TF-IIIIIIIAVAVLVVIIAAVVGF : 229
GA_DN658147: FQMRVDLKLSSVPAEDWRRYDCVFQLSGVDEDIVTKLDKTRNNT----------EKPAGST-----F--IIIIFIAVAVLVAIIAAVVGI : 270
GA_DN715043: FQMRVDLKLSSVPAEDWRRYDCVFQLSGVDEDIVTKLDKTRTNT----------EKPAGST-----F---IIIIIAVAVL-VIIAAVVGF : 230
GA27       : FQMRVDLKLSSVPAEDWRRYDCVFQLSGVDEDIVTKLDKTRTNT----------EKPAGPPPPST-F---IIIFIAVAVLVAIIAL---F : 240
GA_DW039424: FQMRVDLNLSSVPAEDWRRYDCVFQLSGVDEDIVTKLDKTRDHR----------EKPAGST----------------------------- : 302
GA8        : FQMRVDLKLSSVPAEDWRRYDCVFQLSGVDEDIVTKLDKTRTNRGRSETRSGEGGKQTSTL-----F---TLCVLDLFLFVVIL--RLG- : 316
GA11       : FQMRVDLKLSSVPAEDWRRYDCVFQLSGVDEDIVTKLDKTRTNTGRSETRSGEGGKQTSTLSTLCVLDLGSFVFL-------------- : 313
GA_UBA     : FQMRVDLKLSSVPAKDWRRYDCVFQLSGVDEDIVTKLDKTRTNTGRSETRSGEGEKPAAST-----F---IIIIIAVAVL-VIIAAVVGF : 341
GA_UAC     : FQMRVDLKLSSVPAEDWRRYDCVFQLPGVHEDIVTKLDKTRTNTGRSETRSGEGGKQTSTL----------------------------- : 321
GA5        : FQMRVDLKLSSVPAEDWRRYDCVFQLSGVDEDIVTKLDKTRTNTGRSETR----E--A-ST-----F----IIIIAVAVLVVIIAAVVGF : 330
GA30       : FQMRVDLKLSSVPAEDWRRYDCVFQLSGVDEDIVTKLDKTRTNTGRSETRSGEGEKPAGST-----F---IIIIIAVAVLVVIIAAVVGF : 261
GA16       : FQMRVDLNLSSVPAEDWRRYDCVFQLSGVDEDIVTKLDKTRTNT-------DKFIHPFVNS-----F---QVRALSVAVLVVIIAAVVGF : 313
GA19       : FQMRVDLKLSSVPAEDWRRYDCVFQLSGVDEDIVTKLDKTRTNT----------EKPAGSTST---F----IIIIAVAVLVVIMAAVVGF : 333
GA15       : FQMRVDLNLSSVPAEDWRRYDCVFQLSGVDEDIVTKLDKTRTNR---------------------------------------------- : 304
GA21       : FQMRVDLKLSSVPAEDWRRYDCVFQLSGVDEDIVTKLDKTRTNA----------EKPAGST-----FIIIIIIIIAVVVLVVIIAAVVGF : 335
HLA-A2     : FQKWAAVVVP--SGQEQ-RYTCHVQHEGLPKPLTLRWEPSSQPTIP-------------------IVGIIAGLVLFGAVITGAVVAAVMW : 308
                      *             260       *         280                           *         300
```

```
                      Exon 6  (CYT 1)                          Exon 7  (CYT 2)
GA1         : KVYRK-RN--KRPSSSS---------TVVSDGSEELNPKP--------GLWVNVALDQQAMLHILLHKLSIYGSSFI------------- : 390
GA2         : KVYRK-RN-AKRSSAKCPSS----TDGSEESLRDKLNPKP--------GLWVNIALDQQAMLHILLHKLSIYGS-FILHLNV-------- : 389
GA4         : KVYRK-RN--KCSS--------ASTDGSEESLRDKLNPKP--------GLWVNIALDQQAMLHILLHKLSIYGS-FIFHLNV-------- : 396
GA9         : KVHRK-RN-AQRSSAKPSSSSSASTDGSE--VTEELNPKP--------GLWVNIALDQQAMLHILLHKLSIYGSSFI------------- : 376
GA12        : KVYRK-RN------PNALLLLLLLLTALS---SEELNPKP--------GLWVNIALDQQAMLHILLHKLSIYGS-FIFHLNV-------- : 401
GA14        : KVH---RN-AKRSS--------AST-----EVSEELNPKP--------GLWVNIALDQQAMLHILLHKLSIYGSSFI------------- : 367
GA17        : KVYRTVRE-AQRSSSSSSS-----TDGSE--VSEEVNPNP--------GLWVNIALDQQAMLHILLHKLSIYGS-FIFYLNV-------- : 400
GA6         : KVYRKRNVSAQRSSSSS-----ASTDGSE--LSEELNPKP-------------------------------------------------- : 244
GA20        : KVYRK-RN-AKYSSAKCPS------DKSEEESLSGTN-----------GLWVNVALDQQAMLHILLHKLSIYGSSFI------------- : 391
GA_DW664617 : KVYRK-RN-AKYSSAKCPS------DKSEEESLSGTN----------------------------------------------------- : 261
GA22        : KVHRN-RN--KRPSSASSSSS-ASTDGSE--LSEELNPKP--------GSWVNIALDQQAMLHILLHKLSIYGSSFI------------- : 372
GA24        : KLYRN-RN-AKPSSSSS-----ASTEGSE--VTEELNPKP--------GFWVNVALDQQAMPHILLHKLSIYGSSFI------------- : 392
GA_UAA      : KVYRE-RN-AKHSSSSSSS---AVGSEL--------------------GLWVNIALDQQAMLHILLHKLSIYGS-FIFHLNV-------- : 389
GA_DW676150 : KVYRK-RN-AKRSPSSS-------TVGSE--LSEELNPNP*-SRRPAHSLWVNIALDQQAMLHILLHKLSIYGSSFIQTSESGLVNIWKL : 288
GA_DW676054 : KLYRK-RN-AKPSSSSS-----ASTDGSE--VTQELNPKP-RSRRPAHSLWVNIALDQQAMPHILLHKLSIYGSSFI------------- : 122
GA10        : KVHRK-RN----------------------------------------GLWVNIALDQQAMLHILLHKLSIYGS-FIFHLNV-------- : 295
GA13        : KVYRK-RN-GESSSSSSSS-----TDGS--DVTEEL------------------------------------------------------ : 359
GA23        : KVHRN-RN-GESSSSSS-----ASTDGS--ELSEEL------------------------------------------------------ : 358
GA26        : KVHRK-RN-AKPSSSSS-----ATTDGS--DVTEELNPKP-------------------------------------------------- : 341
GA_DW035296 : KLYRK-RN-AKPSSSSS-----ASTDGS--EVTQELNPKP-------------------------------------------------- : 296
GA_DN656534 : KVYRK-KN-AKCSSAKPSSSS-ASTDDS--EVTEELNPYPSDKFEEERLSGTN------------------------------------- : 277
GA_DN658147 : KVYRN-RN-AKYSSVQCSSSSSSSGSNGGSDVTEGTES---------------------------------------------------- : 306
GA_DN715043 : KVYRK-RN-AQRSSSSSSS-----AVGS--DVTEELNPKPLKLASCTQFMGECCSGSTSDAPHSSP------------------------ : 287
GA27        : ISAK--RSAAKSPFSSAS------TDGS--DVTEELNPK--------------------------------------------------- : 269
GA_DW039424 : ---------SSSSSSSLWLVLSSSSLLWVGSRVTERRTPNSLSGNALLLLLLSALSSLKD------------------------------ : 353
GA_UBA      : KVHRK-RN---DDYLCSTPPPTVQESLTVRSCAIKAAHFLIDCTTAPVEALVSWISPLLAEEDHRSRARAR------------------- : 408
GA5         : KVYRNRNAKCP------------------------------------------------------------------------------- : 341
GA30        : KVYRKRNGE--------------------------------------------------------------------------------- : 270
GA16        : KLYRK-RK---------------------------------------------------------------------------------- : 320
GA19        : KVYRK-RN-AQRSSAKWPFDKSEEERLSWTN----------------------------------------------------------- : 362
GA21        : MVHRKRNGER-------------------------------------------------------------------------------- : 345
HLA-A2      : RRKSSDRKGGSYSQAASSDSAQGSDVSLTACKV--------------------------------------------------------- : 341
               *         320       *         340
```

Text S3f. Stickleback seven exon genes

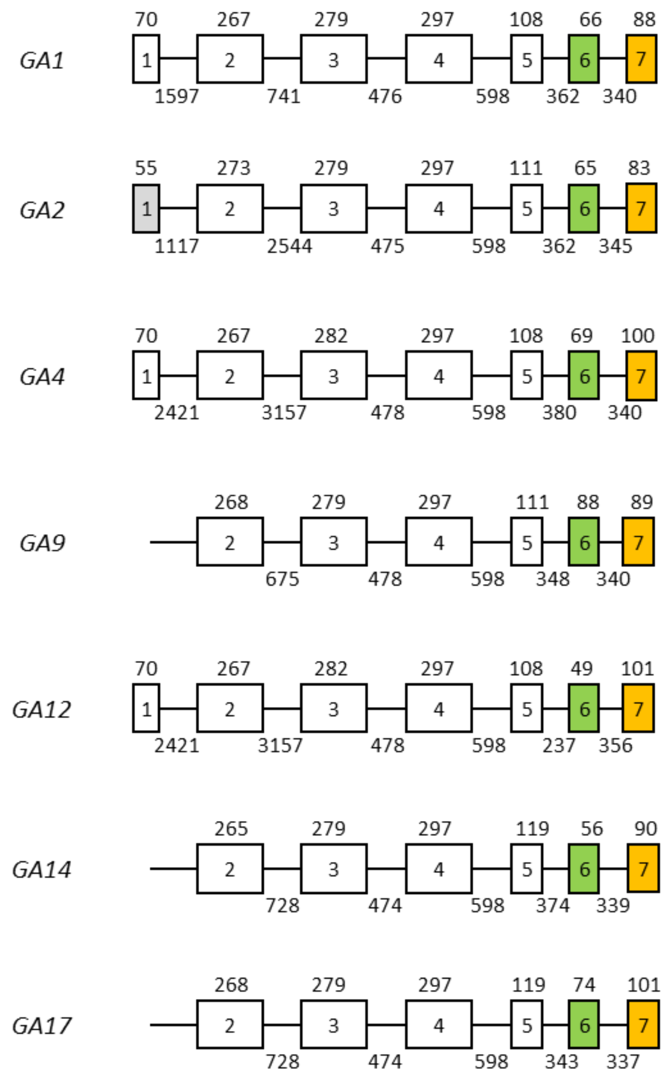

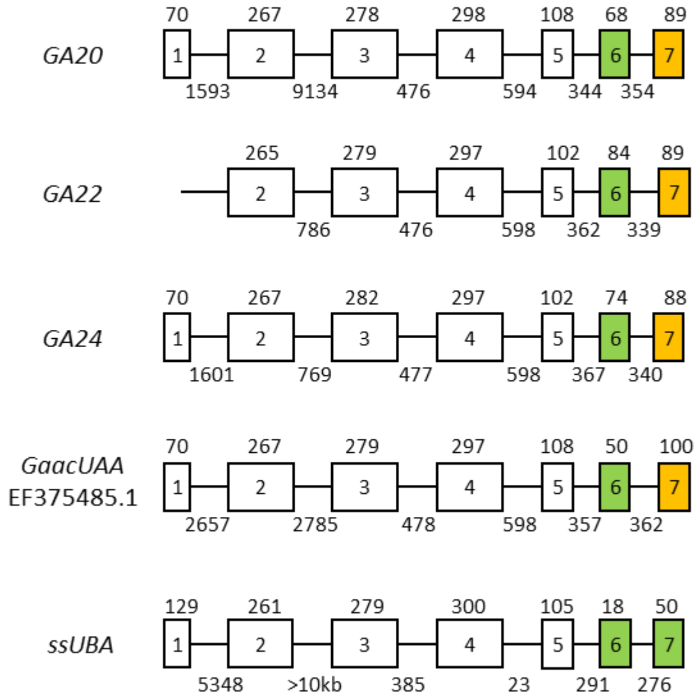

Overview of stickleback MHC I gene organization. Exons are numbered and shown as boxes with exon size above and intron size below the gene. Exon 1 encodes the leader sequence, exon 2-4 encode alpha 1-3 domains, exon 5 transmembrane and exons 6 and 7 cytoplasmic domains. The green (exon 6) and orange (exon 7) boxes are cytoplasmic domains without and with endosomal sorting motifs respectively. Grey box is an incomplete leader sequence. The GaacUAA gene information originates from a BAC sequence [Schaschl H. & Wegner K.M. Contrasting modes of evolution between the MHC class I genomic region and class II region in the three-spined stickleback (*Gasterosteus aculeatus*). Immunogenetics 59:295-304, 2007]. The GA10 gene lacks an alpha 1 domain and is not shown. Gene organization of the salmon UBA locus is shown for comparison [Lukacs et al. Genomic organization of duplicated MHC class I regions in Atlantic salmon. BMC Genomics 8: 251, 2007].

Text S3g. Alignment of deduced Atlantic cod MHC class I amino acid sequences

Numbering below the alignment relates to mature human HLA-A2 residues. Regions are depicted on top, where the cod exon intron structure is currently unknown, so predictions were made using human and salmon sequences. Dots indicate identity and dashes are gaps introduced to maximize the alignment. Ten sequences were randomly selected for each of the two cod clades defined by Malmstrøm et al.[main text reference 20] and shown separated by a solid line in the alignment. HLA-A2 peptide anchoring positions i.e. Y7, Y59, Y84, T143, K146, W147, Y159, Y171 are shown below the alignment, and as a red shaded residue in the HLA-A2 sequence, where the Y84 is consistently R in teleosts and thus shown as Y/R84. Residues in HLA-A2 known to contribute to the six pockets A through F [main text reference 1 and 3] are indicated above the alignment. Yellow shading shows endosomal sorting motifs in the cytoplasmic domain as defined by Malmstrøm et al.[20]. GenBank accession numbers for 20 cod sequences are GM1 is AGV52778.1 , GM10 is AGV52769.1 , GM11 is AGV52768.1 , GM12 is AGV52767.1, GM24 is AGV52755.1 , GM26 is AGV52753.1 , GM35 is AGV52744.1, GM41 is AGV52738.1, GM49 is AGV52730.1, GM54 is AGV52725.1 , GM58 is AGV52721.1 , GM62 is AGV52717.1, GM83 is AGV52696.1, GM90 is AGV52689.1 , GM93 is AGV52686.1, GM98 is AGV52681.1,GM100 is AGV52657.1, GM104 is AGV52653.1, GM120 is AGV52672.1, GM125 is AGV52667.1 while accession numbers for the salmon sasa*UBA*(**0301* allele) and HLA-A2 sequences are AAN75116.1 and AAA76608.2 respectively.

```
           Alpha 1 domain

                A B                                                           A  A   B  C
              A B C             C B         B          B                  A   B  BB  C  FC  F  FF  F
GM100    : VIHSLQFFYTGSS-GLSTFPEYVMVLMVDEVQVEYYDSN--TQRIITKQDWADQAT-RDKDPDYLERETENRKGNQQVEKGNIGTLKKRFNQ : 88
GM125    : .............-....................C....--...............-................................... : 88
GM104    : ......L...A..-.......F.T.Q.M...........--.....P....V...N-...V......A..R...I...F.ADM....R.... : 88
GM120    : ..........A..-.........I.Q......I......--...S.P.....E..N-..T.......DA.....T...V.A.M.I..Q.... : 88
GM93     : ......S.D.A..-..P......................--..........V...N-.............R.......F............. : 88
GM98     : .....H..D.A..-.........G.V.............--.....P........N-.E-ES.S...D..I......AF.AGM.I..R.... : 87   Clade 1
GM35     : ......V.H.A..-......G....V.............--..........I..FY-..-P.GE..IA..R......TF.A....A.Q.... : 87
GM54     : ....QH..F.A..-.........D.Q......IG.....--...S.P........N-.........KD......L..AF.A.M.I..Q.... : 88
GM58     : ..........A..-..P........V.............--..........V...N-E..V.........R.......L............. : 88
GM83     : ........S.A..-.......F...E.............--...S.P....VE..N-...V.............I..GF.AGM.I..Q.... : 88
GM90     : .....HY...A..-..TA...F.A.G...G..MLHF..F--SKKTVL....ME...-.G-Y.S....SNRLSL.S..AF.AD..IA.Q.... : 87
GM1      : .L...HY......-.......F.A.G...G..ID.....--I..AVL....ME.VI-.E-.R.....NAGILQ.A..TY.AGV.I..Q.... : 87
GM10     : .L..MRI...A..-..TA...F.A.G...G..FN.....--...TVP....MELVI-.E-.R.......GK...A...F.A...IA.Q.... : 87
GM11     : .L...HY......-..TA...F.A.G...G..MLH...V--SK.AVA....MERY.-..-H.EV...DNG.LQ.A..TF.AGV.I..Q.... : 87
GM12     : .L...HY...A..-..T....F.V.G...G..MVH...V--SK.AVA....ME.V.-KE-.R...D...GKFQ.S..SF.VS...A.Q.... : 87   Clade 2
GM24     : .L...HY...A..-.......F.A.G...G..ID....I--...TVL....MK...-SG-.G.......GKAQ.A..TF.A....A.Q.... : 87
GM26     : .L...HY...A..-...A...F.A.G...G..MVH....--...TVL....ME.V.-SG-.G...V.N.GKFQ.A...F.A...IA.Q.... : 87
GM41     : .L...HY...A..-..KA...F.A.G...G..MLHFH..--...VVL....ME.L.-.E-Y......S.GLSL.S..SF.Y...I.M....R : 87
GM49     : .L...HY......-.......F.A.G...G..ID....I--..KKVL..E.MKRY.I.E-.......L.GILQ.H..TF.AS.VNV.Q.... : 88
GM62     : .L...HY...A..-..T....F.A.G...G..IN.....--...VVL....MERFA-SG-.A.....S.GIIQ.T...F.A...IA.Q.... : 87
sasaUBA  : AT...RYV..AT.-.IPD...F.T.GL.NGEPIS....I--IR.ETPR...MAK---TE-GS..W.SQ.QVSI.SE.TF.A..DVA.Q.... : 85
HLA-A2   : GS..MRY.F.SV.RPGRGE.RFIA.GY..DT.FVRF..DAAS..MEPRAP.IE.-----EG.E.WDG..RKV.AHS.THRVDL...RGYY.. : 87
                 Y7                                                       Y59                    Y/R84
           1        *         20        *         40        *              60        *         80
```

```
             Alpha 2 domain
                     A
                     B               C C
                   C C               D E                              E        DD  A
                 F E D               E F      FF                  F  FF     E  EE  DD  A   A   A
GM100   : TG-GAHILQRMYGCEWDDEDGT-TEGYQQYGYDGEDFLSLDLKTLIWVAPVHQAFATKLRWDHDTAYNQYLKNYYTKECVDWLKKLLAYGKS : 178
GM125   : ..-...................-..................................................................... : 178
GM104   : ..-....I.T.S..........-....D.........I.......T........LT..HK.EQNR..MEQQ.H....V.......Y...... : 178
GM120   : ..-....I...D..........-....D.H.......I......MT.....R..VT..H.LEQNR..MEE..H............Y.V.... : 178
GM93    : ..-....V..............-....E.........I.......T.........PS...L.....Q...K...FN........VY.V.... : 178
GM98    : ..-....I.Q............-....N.L...............T.........PS...L..N......V...........V....G.... : 177
GM35    : ..-....V.K...........S-.D..R.F.......IAW.M..MT.....R.TVI..Q..NEER.QL......I.ED.......Y..N... : 177
GM54    : ..-....I...D..........-....D.H.......I.......T.....R...S..Q...GLR.QTVRY.Y............Y.V.... : 178
GM58    : ..-...VY.Q............-....E.H...............T.........PS...L..N......V...........V....G.... : 178
GM83    : ..-....I.K.N.........S-.D..R.F.......IAW.M..MT.....R..VI..Q..NEER.QL......I.ED.......Y..N... : 178
GM90    : ..-....Y.E..........DS-.D..D.........IAF.....T.....R..YP..Q...GLRG.D.QQ...L.....Y...NY.V.... : 177
GM1     : ..-.T.LY.........G..DS-.D..N.........IAF.P...T.....R..VP..QK..GLR...E.W...Q..........Y...... : 177
GM10    : ..-...MF.N..........DS-.D..N.........I....EH.T.....Q..CT..RKL.ENK.QL..F..............Y.V.... : 177
GM11    : ..-...MV.N...........A-ID..D..........AWNM..MT.....P...S..Q..NQ.R.DL..N...Q..........Y...... : 177
GM12    : ..-...LY.N............-.D..H.F.F.....IAF.....T.....Q...P..R...ENR.D...IR..F..........Y.T.... : 177
GM24    : ..-....V.K...........S-.D..R.F.......IAW.M..MT.....R..VI..Q..NEER.QL......I.ED.......Y..N... : 177
GM26    : ..-...MV.W..........NS-.D..N.........I.......T.....R...S..Q...GLR.QTVRY.Y............Y...... : 177
GM41    : ..-...VY.E...........S-.D..D.F.............S.T.....P...S......QKRG..EQQ.........H....Y.V.... : 177
GM49    : ..-.T.LY.F.C........DS-.D..N.........IAF.....T.....R..VP..QK..GLR...E.W...Q.....E....Y.S..R. : 178
GM62    : ..-...MF.N..........DS-.D..H.........I.......T.....Q..CT..RKL.ENK.QL..F..............Y.V.... : 177
sasaUBA : ..-.V.VN.K.........T.V-...FD.D........AF.....T.I..TP..VI...K..SN..Q.E.R...L.QT.IE....Y.D.... : 175
HLA-A2  : SEA.S.TV......DVGSD-WRFLR..H..A...K.YIA.KEDLRS.T.ADMA.QT..HK.EAA-HVAEQ.RA.LEGT..E..RRY.EN..E : 177
                                                                  T  KW147         Y159        Y171
            *         100        *         120       *         140       *          160       *
```

```
                Alpha 3 domain
GM100    : TLQRTERPRVSLLQRSPS---SPVVCHATGFYPNRVVVFWRRDGQELHEQVDPGEVLPNHDGTFQVSVDLDLTAVPQEDW--GRYECVVQLK : 265
GM125    : ..................---...........................................................--.......R.. : 265
GM104    : ...K..............---...........................................................--.......... : 265
GM120    : ..................---..........F................................................--.......... : 265
GM93     : .............R....---............D....................................Y.........--.......... : 265
GM98     : ........Q.........---.................................................Y.........--.......... : 264
GM35     : ..................---............D....................................N.K.......--.......... : 264
GM54     : .....D............---............D......T.............................N.K.......--.......... : 265
GM58     : ........Q.........---...................................................K.......--.......... : 265
GM83     : ..................---............D....................................N.K.......--.......... : 265
GM90     : ..............K...---............D....................................N.K.......--.......... : 264
GM1      : ..................---............D...................................FN.K.......--.......... : 264
GM10     : .....D............---............D......T.............................N.K.......--.......... : 264
GM11     : ..................---............D......................................K.......--.........R : 264
GM12     : ...............R..---............D..................L.................N.........--.......... : 264
GM24     : ..................---............D....................................N.K.......--.......... : 264
GM26     : ..................---............D....................................N.K.......--.......... : 264
GM41     : A.................---............D........H...........................N.........--.......... : 264
GM49     : ..................---............D....................................N......-..--R........R : 264
GM62     : .....D............---............D......T.............................N.K.......--.......... : 264
sasaUBA  : ..M..VP.S.....KT..---...T........SG.M.S.QK...DH..D.EH..T.Q.D.....K.SH.TV.---P.E.KNNK.Q....VT : 261
HLA-A2   : .....DA.KTHMTHHAV.DHEATLR.W.LS...AEITLT.Q...EDQTQDTELV.TR.AG.....KWAAVVVP--SGQE---Q..T.H..HE : 264
              180       *         200      *          220      *          240      *               260
```

```
                     CP            TM                        CYT
GM100    : GI-EDISTTLDPAHIRTN-GGDNHILAFILTGVAVVAV-VAAVAVVGVFLYRKKNDSEKCHKPVD-SDTSSENTEGQKLAPEYQPLPT-- : 349
GM125    : ..-...............-...................-..........................-....................S.-- : 349
GM104    : ..-.....P.........-REGS............L..--VVA.....V..Q.R...D.R.....-....................S.-- : 348
GM120    : ..-.....P....L....-REGS............L..--VVA........Q.R...D.R....G-................A...T.-- : 348
GM93     : ..-.....P......M..-REGS............L..--VVA........K.R...D.R....G-...........NPS..A...T.-- : 348
GM98     : ..-...............-.................---A.FAV......C..R...........-............PS..A...T.-- : 346
GM35     : ..-.....P....L..C.-S.KSGLTIP.II.LL.LLLAA..A.....L..K.R.A.D.R....G-...........NP...A...TK-- : 349
GM54     : ..-.....P....L....-S.KSGLTIP.II.LL.LLLAA..AV....L..K.R.A.D.R....G-...........NP...A...TK-- : 350
GM58     : ..-.....P....L....-..KSGLTIP.II.LL.LLL--..ATI...L..K.R.A.DQR....AT......DA---------------- : 335
GM83     : ..-.....P....L..C.-S.KSGLTIP.II.LL.LLLAA..A.....L..K.R.A.D.R....G-...........NP...A...TK-- : 350
GM90     : ..-.....P.GR.L....-S.KSGLTIP.II.LL.LLL-A..A.....L..K.R.A.D.G..L.GS......K....KNP...A...---- : 347
GM1      : ..-.....R....L....-..KSGLTIP.II.LL.LLLVA..AV....L..K.R.A.D.QQ..GG-......G....NPP..S...T.-- : 349
GM10     : ..-.....P....L....-S.KSGLTIP.II.LL.LLL-A..A.....L..K.R.--D.R....G-...........NP...A...TK-- : 346
GM11     : ..-.....P....L....-E.KSGLTIP.II..L.LLL--..A.I...I..K.R.A.D.G....AS...........NP...A...T.-- : 348
GM12     : S.-.....P....L....-..KSGLTIP.II.LL.LLL--..ATI...L..K.R.A.DQR....AT......DA---------------- : 334
GM24     : ..-.....P....L..C.-S.KSGLTIP.II.LL.LLLAA..A.....L..K.R.A.D.R....G-...........NP...A...TK-- : 349
GM26     : ..-.....P....L....WEDKSGLTIP.II.LL.LLL-A..A.....L..K.R.A.DQR....G-...........NP...A...T.-- : 349
GM41     : ..-.....P....L....WEDKSGLTIP.II.LL.LLL-A..A.....L..K.R.A.DQR....G-...........NP...A...T.-- : 349
GM49     : ..-.....S....L....-S.KSGLTIP.II.LL.LLL--..A.I...L..K.R.A.DQR....AT......DA---------------- : 334
GM62     : ..-.....P....L....-S.KSGLTIP.II.LL.LLL-A..A.....L..K.R.A.D.R....G-...........NP...A...TK-- : 348
sasaUBA  : .LQ..FIKV.TESE.K..-WN.PN--IVLII..V.ALLL.VVAV....VIWK..S---.KGFVPA-.TSDTDSDNSGRA.QMT------- : 337
HLA-A2   : .LPKPLTLRWE.SSQP.IP-------IVGIIAGL.LFGA.ITG...AAVMW.R.SSDR.GGSYS-QAAS.DSAQGSDVSLTACKV----- : 341
                *         280              *         300       *         320        *         340
```
