# Supplementary material for: A comprehensive analysis of teleost MHC class I sequences
Source: BMC Evol Biol. 2015 Mar 6;15:32. doi: 10.1186/s12862-015-0309-1 (PMC4364491; doi:10.1186/s12862-015-0309-1)

Additional file 7: Text S4. Additional Z lineage data

Table of Contents

		Page
Text S4a	Alignment of deduced Z lineage amino acid sequences	2
Text S4b	Residue conservation of tetrapod MHCI a1a2 domain sequences	5
Text S4c	Percent identity per domain between deduced Z lineage amino acid sequences	7
Text S4d.1	Phylogenetic tree of deduced Z lineage alpha 1 domain amino acid sequences	8
Text S4d.2	Phylogenetic tree of deduced Z lineage alpha 2 domain amino acid sequences	9
Text S4d.3	Phylogenetic tree of deduced Z lineage alpha 3 domain amino acid sequences	9

Text S4a. Alignment of deduced Z lineage amino acid sequences

Alignment of deduced teleost Z lineage sequences with complete a1-a3 domains. Dashes are introduced to maximize alignment and horizontal lines are used to separate sequence groups and/or species. Human HLA-A2 residues known to anchor peptide termini i.e. Y7, Y59, Y84, T143, K146, W147, Y159, Y171 are shown as shaded red residues. HLA-A2 residue positions known to contribute to the six pockets A through F [main text reference 1 and 3] are indicated above the alignment and shaded yellow when conserved among typical Z lineage sequences. The AM21 sequence, with most pocket residues complying with the conserved teleost Z lineage motif, is an incomplete gene fragment due to a short contig. Numbering above the alignment relates to the mature human HLA-A2 sequence while residue numbers of each sequence are shown to the right. Residues are colored according to physiochemical properties. Conserved N-linked glycosylation motifs are underlined. Sequence names reflect species Latin names where sasa is *Salmo salar* (Atlantic salmon), DR is *Danio rerio* (zebrafish), AM is *Astyanax mexicanus* (blind cavefish), OL is *Oryzias latipes* (medaka), GA is *Gasterosteus aculeatus* (stickleback), ON is *Oreochromis niloticus* (Nile tilapia), TN is *Tetraodon nigroviridis* (green spotted pufferfish), TR is *Takefugu rubripes* (Japanese pufferfish), caau is *Carassius auratus* (goldfish), cyca is *Cyprinus carpio* (common carp), LO is *Lepisosteus oculatus* (spotted gar), eel is *Anguilla japonica* and sturgeon is *Acipencer sinensis*. Eel and sturgeon Z lineage sequences derive from assembly of transcriptome and genomic SRA reads (additional file 4: Text S2). From the recently available genome of Amazon molly (*Poecilia formosa*) only alpha 1 and alpha 2 domain sequences from the three identified Z lineage sequences are shown (additional file 4: Text S2). Zebrafish locus designations shown in parenthesis originate from Dirscherl & Yoder, 2013 [main text reference 34], where the ZAA and ZLA sequences not identified in the Ensembl genome are not shown. Sequence reference not found in additional files 3: Text S1 or 4: Text S2: Lungfish (*Protopterus aethiopicus*) is GenBank accession # AAF15304.1. Sub-lineages for the atypical Z lineage sequences from cavefish and cyprinids are also shown in parenthesis where the typical cavefish Z lineage sequences were defined as the Z1 lineage.Abbreviations are: CP is connecting peptide, TM is transmembrane region and CYT is cytoplasmic domain.

```
                   Leader sequence              Alpha 1 domain
                                                1     *        20        *        40        *        60    *
                                                      A B                                             A  A  B  C
                                                    A B C                C B        B        B        A  B BB C FC
HLA-A2      : -----------------------------GSHSMRYFFTSVSRPGRG-EP---RFIAVGYVDDTQFVRFDSDAASQRMEPRAPWIEQ-EGPEYWDGETRKVKAHSQTHR :  75
sasaUBA     : ---------MKCFILLLLG-IALHSSSA-ATHSLRYVYTATS-GIPD-FP---EFVTVGLVNGEPISYYDSI--IRRETPRQDWMAKTEGSDYWESQTQVSIGSEQTFK :  91
sasaZAAa    : -----MNISHLTVFVLYFSLECICQ--S-DTYSLSYIYTALS-KPVD-LPGIHEFTAMGLMNNQQIDYYDSV--SKKKIPKQDWMREKLPADYWEKGTQSRKSKEQWFK :  97
sasaZBAa    : ------------------------------IYSLNYIYTALS-KPVD-LPGIHEFNAMGLMNNKQIDYYDSV--SKKKIPKQNWMREKLPADYWEKGTQSRKSKEQWFK :  75
sasaZCAa    : --MSAFKMYVVALLLLFATLSTEDT--V-ETWSLNYIYTALS-KPVE-LPGIHEFTAMGLMNDKQIDYYDSV--AKKKIPKQDWMREKLPADYWEKGTQSRKSKEQWFK : 100
sasaZDAa    : ------------------------------IYSLNYIYTALS-KPVD-LPGIHEFTAMGLMNNQQIDYYDSV--SKKKIPKQDWMREKLPADYWEKGTQSRKSKEQWFK :  75
sasaZBAb    : -------MYTFMLFVFYFSTECIVQSQS-EIYSLNYIYTALS-KPVE-LPGIHEFTAMGLMNDIQIDYYDSV--DKKKIPKQDWMREKLPADYWEKGTQSRKRKEQWFK :  97
sasaZCAb    : -------MYTFMLFVFYFSTECIVQSQS-EIYSLNYIYTALS-KPVE-LPGIHEFTAMGLMNNRQIDYYDSV--DKKKIPKQDWMRDKLPADYWEKGTQSRKSKEQWFK :  97
DR4   (ZBA) : -------MGSFAVLFSAVMLVAVVPAWT-EKHSLYYIYTALS-RPVN-LPGIYEFTAMGLLDDRQIDYYNSE--QQKKIPKQHWMKEKMQEDYWEKGTQSRKSKEQWFN :  97
DR3   (ZCA) : -------MAVLAVLFSAVMLLSVVSVRT-EKHSLYYIYTALS-RPVD-QSGIYQFTAMGLLDDRQIDYYNNI--DQKKIPKQHWMKEKMQEDYWEKGTQSRKSKEQWFN :  97
DR2   (ZDA) : -------MAVFVVLLLAVMPLDVEPATT-EKHSLYYIYTALS-RPVN-LPGIHEFTAMGLLDDRQIDYYNSQ--DQKKIPKQHWMKEKMQEDYWEKGTQSRKSKEQWFN :  97
DR1   (ZEA) : -------MAVFAALFSVVMLFTVVPAWT-EKHSLYYIYTALS-RPVH-LPGIYEFTAMGLLDDRQIDYYNSQ--EQKKIPKQHWMKEKMQEDYWEKGTQSRKSKEQWFN :  97
DR5   (ZFA) : ---MIMDVKVLTLLCVFLLYGALPLCRA-EKHSLYYIYTGLS-RPVD-LPGIYEFSAMGLLDDRQIDSYNSR--EQRKIPKQQWMKEKMQEDYWEKGTQSRKSKEQWFN : 101
DR6   (ZGA) : ------------------------------KHSLYYIYTGLS-KPLD-LPGIYEFSAMGLLDDRQIDSYNSR--DQRKIPKQQWMKEKMQEDYWEKGTQSRKSKEQWFN :  75
DR7   (ZHA) : ------------------------------KHSLYYIYTGLS-KPLD-LPGIYEFSAMGLLDDRQIDSYNSE--EQRKIPKQQWMKEKMQEDYWEKGTQSRKSKEQWFN :  75
DR8   (ZIA) : MMSGGVASGICALLCVFLLCGDLPSAQG-EKHSLYYIYTSLS-KPLD-LPGIYEFSAMGLLDDRQIDSYNSE--EQRKIPKQQWMKEKMQEDYWEKGTWSRKSKEQWFN : 104
DR9   (ZJA) : MTS---FDRSFTLLYLCLFHVIMSSFRA-EKHSLYFIYTGLS-RPVD-LPGIYEFSAMGLLDDRQIDSYNSE--EQRNIPKQQWMKEKMQEDYWENRTQSRKEKQLWFY : 101
DR30  (ZKA) : MMSGGVASGICALLCVFLLCGDLPSAQG-EKHSLYYIYTGLS-KPLD-LPGIYEFSAMGLLDDRQIDSYNSE--EQRKIPKQQWMKEKMQEDYWEKGTQSRKSKEQWFN : 104
AM8   (Z1)  : -------MGVSTAVCVLLLNAVFISTLA-ERHSLYYIYSALN-KDVS-LPGIYEFTALGLLDGREIDYYNSK--EQKKIPKQSWMMEKMQEDYWEKGTQSRKSKEQWFK :  97
AM18  (Z1)  : ------------------------------NHFLYYIYIGLS-KDLN-VKGLYEFTALGLLDDREIDYYNSK--EQKKIPKQSWMMEKMQEDYWEKGTQSRKSKEQWLK :  75
AM21  (Z1)  : ------------------------------KHSLFYIYSALN-KDVS-LPGIYEFTALGLLDDREIDYYNSK--EQKKIHKQNWMIEKMQKDYWEKGTLSRKSKERWFK :  75
AM23  (Z1)  : ------------------------------NHFLYYIYTGLS-KDLN-LKGVYEFTALGLLDDREISFFNGVQQLQDDIKKRTFFNEKQRRTRLKKGTQSRKSKEQWLK :  77
OL4         : ----------MLITSVLVLLGSGLLVNC-EKHSLTYIYTAFS-HPVK-LPGIHEFTAMGLLDNRMIDYYDSS--VQKKIPKQDWMKERLQQEYWDKGTQSRQSKQQWFK :  94
OL5         : ------------------------------KHSLTYIYTAFS-HPVK-LPGIHEFTAMGLLDNRMIDYYDSS--VQKKIPKQDWMKERLQQEYWDKGTQSRQSKQQWFK :  75
OL16        : ------------------------------KHSLTYIYTAFS-HPVK-LPGIHEFTAMGLLDNRMIDYYDSS--VQKKIPKQDWMKERLQQEYWEKGTQSRQSKQQWFK :  75
GA18        : ----------MSPLAVLVLLQVTAAVFG-VKHSLTYTYTAFAHKPVG-LPGIHEFTAMGHLDTRMIDYFDSD--QQLKVPKQPWMGERLDKDYWVKGTQSRQSKQQWFK :  95
ON22        : ----------MFVISVLVLVGTGVTVNS-EKHSLHYIYTGLS-KPVG-LPGIHEFTAMGLLDGRMIDYYDSE--NQTKVPKQEWMREHLPADYWVKGTQSRRIKQRWFK :  94
ON23        : -----MLGSKMFTLALFALLCPGPTQSR-DTHSLHYIYTALS-KPVG-LPGIHEFTAMGLLDGRMIDYFDSE--HQAKVPKQQWMRERLPADYWDKGTQSRKSKQQWFK :  99
ON24        : ------------------------------THSLHYIYTALS-KPVS-LPGIHEFTAMGLLDDRMIDYFDSE--NQAKVPKQQWMRERLPADYWDKGTQSRKSKQQWFK :  75
ON33        : ------------------------------THSLHYIYTALS-KPVG-LPGIHEFTAMGLLDNRMIDYFDSK--NQVKVPRQEWMRKRLPADYWEKGTQSRKSKQQWFK :  75
ON34        : ------------------------------THSLHYIYTALS-KPVG-LLGIHQFTAMGLLDGRMIDYFDSE--NQAKVPKQEWMRERLPADYWERGTRSRKSKQQWFK :  75
ON35        : ----------MNLFAVFVLLGTVLTVNC-ETHSLHYIYTALS-KPVG-LPGIHEFTAMGLLDDRMIDYFDSE--HQAKVPKQEWMEKRLPADYWEKGTQSRKSKQQWFK :  94
TN13        : ----------MVLLVLLGSGLVVDGGLS-EIHSLHYIYTALS-RPVN-RPGIHEFTAMGMLDDHMIDYFDSD--KQVKVPKQTWMKTELEENYWEKGTQSRKSKQQWFK :  94
TR21        : ------MELTPFFILVFGILGLGPTVNP-EKHSLHYIYTATS-QPIS-RQGIHEFTAMGVLDGNVIDYFDSD--AQKKVPRQKWMEKELDKSYWEKGTESRKSKQQWFK :  98
Amazon_Za   : ---------------------------- EKHSLTYIYTAFS-KPVN-LPGLHEFTAMGLLDNNMIDYYDSE--HQVKVPKQDWMEKHLEPEYWEKGTQSRKSKQQWFK :  76
Amazon_Zb   : -----------------------------EKHSLTYIYTAFS-KPVN-LPGLHEFTAMGLLDNNMIDYYDSE--HQVKVPKQDWMAKHLEPEYWDKGTQSRKSKQQWFK :  76
Amazon_Zc   : -----------------------------EKHSLTYIYTAFS-KPVN-LPGLHEFTAMGLLDNRMIDYYDSE--NQVKVPKEDWMEKHLEPEYWEKGTQSRKSKQQWFK :  76
Gar LO14    : -----------------------------ERHSLHYIYTALS-KPVDGIP---EFTAMGVLNDRRIDYYDNW--IMQKIPKQSWMKTNMPQEYWEKGTQSRKSKEQWFK :  74
Eel         : -----------MKLKIILALFCVFGTVQGEIHNLHYIYTALS-KPIT-QPGVYQFTALGILNDRPIDVYNSK--DKVKKPLQKWMEEHNEKDYWEKGTQSRRSKEQWFD :  94
Sturgeon    : -------------------------------------------CREG-FP---EFTAMGLLDDMQIDYYDSV--NQKKESKQTWMKENMAPDYWEKGTKSRKSKEQWFK :  60
Lungfish    : ----MPGESLKVPQALLVCWFLSVQFVDSSFHSLRYTYSAYA-SNDK-LV---EFVAQGLLDDVQIDYYDNH--IRREVPKQQWMNESMEAGYWERGTQSRNSKEHWFT :  98
AM3   (Z2)  : ---------MMRSITALLLFSFLRIVIG-EKLSLNYCYTMQY-TNS--ESNLYDCTVVTLLNDTQIYFYNSA--DPNKTPKPHWLK-ISETD-WIKSTTWSDGDRQWMN :  92
AM4   (Z2)  : ------MKCYIFKISIRMLKGVFAAMAA-DNHSLLYLYTMQS-KNS--YSNIYDCTAVTLLNDRQIDFYSSSS-DKPRTAKQNWLKNISESD-WIISTEKLQYDTQLLK :  97
AM10  (Z2)  : --------MGESEVWVLLVFGVFLPSYG-DEHSLYYLHTSQFKHN----DSIHEFTALTLLDDVQIDFYNST--DGVRTPKQDWLKEINGSD-WERGSAELRDNGQYLN :  93
AM22  (Z2)  : ---MGTNYPNPTILEKCIIVLGEHHFFT-GKHSLLYLYTMES-KNS--KSNIYDCTAVTLLNYRQIDFYNST--DESRTAKQNWLKNISESD-WKTSTEKLQNDRQLLN :  99
AM25  (Z2)  : ------------------------------EHLLEYLYTMQS-KN----CTIYDCTAVTLLNDRQIDFYNSSS-DKPRTAKQNWMKNISESD-WKTITEKLQSDRSLLN :  72
caauZA1(Z3) : --------------------------------------MFTVM--IK-ADTLPVFSAVCESDHIQISHYS--------TEEQIWMRENLTEHDWDRAPVGPPETTDWYL :  60
cycaZB1(Z3) : --------------------------------------KFTVL--SK-ADIFPEFSAVAVADDIQIKHYS--------NED--WIRSILTEDDWINAPEAPPDSRDWFL :  58
cycaZC1(Z3) : --------------------------------------KYTVL--TK-AGPFPVFSAVGESDHIQIARYR--------SEERVWMRENLTVDAWNEAPGAPAETNDGYL :  60
```

```
                               Alpha 2 domain
                 80           *         100           *         120       *         140       *          160       *
                                       A
                                       B                  C C
                                     C C                  D E                              E        DD  A
              F  FF  F             F E D                  E F F    FF                  F  FF     E  EE  DD  A   A   A
HLA-A2     : VDLGTLRGYY---NQSEAGSHTVQRMYGCDVGSD--WRF--LRGYHQYAYDGKDYIALKEDLRSWTAADMAAQTTKHKWEAA-HVAEQLRAYLEGTCVEWLRRYLENGK : 176
sasaUBA    : ANIDVAKQRF---NQTG-GVHVNQKMYGCEWDDE-TGV---TEGFDQDGYDGEDFLAFDLKTLTWIAPTPQAVITKLKWDSNTAQNEYRKNYLTQTCIEWLKKYLDYGK : 192
sasaZAAa   : VNVDILMKRMRH-NNT--DVHVLQWKVGCEIDQQSDGTLKFIKGIDQYSYDGDDFLAFDDVTMQWVAPVDQALPTKRKLDDVQILNTYTKGYLEKECVDWLSKFMEYED : 203
sasaZBAa   : VNVNILMDRMRH-NNT--NVHILQWKHGCEIDQQRDGTVKFIKGIDQYSYDGDDFLAFDDVTMQWVAPVDQALPTKRKWDGVQILNQYTKGYLEKECVDWLSKFMEYGD : 181
sasaZCAa   : VNVNILMDRMRH-NNT--DVHILQWKHGCEINQQSDGTLKFIKGTDQYSYDGDDFLAFDDVTMQWVAPVDQALPTKRKWDGVQILNQYTKGYLEKECVDWLSTFMEYGK : 206
sasaZDAa   : VNVNILMDRMRH-NNT--DVHILQWKHGCEIDQQSDGTLKFIKGTDQYSYDGDDFLAFDDVTMQWVAPVDQALPTKRKWDGVQILNQYTKGYLEKECVDWLSKFMEYGE : 181
sasaZBAb   : VNVNILMERMRH-NNT--DVHVLQWRHGCEVDKQPDGTLKFMKGIDQYSYDGDDFLAFDDVTMQWVAPVDQALPTKRKWDGVQILNQYTKGYLEKECVDWLSKFMEYGE : 203
sasaZCAb   : VNVNILMERMRH-NNT--GVRILQWKHGCEVDKQPDGTLKFIKGTDQYSYDGDDFLAFDDVTMQWVAPVDQALPTKRKWDGVQILNQYTKGYLEKECVDWLSKFMEYGE : 203
DR4  (ZBA) : VNVNILMERMRH-NKS--DVHVLQWRHGCEIESQ-GNDVRFSRGIDEYSYDGRNFLSFDDAESQWVAPVEEALPTKRKWDNVPILNQYTKGYLEKECVDWLNKFREYGD : 202
DR3  (ZCA) : VNVNILMDRMRH-NNS--DLHVLQWRHGCEIEIQ-GNEHRFSKGIDEYSYDGRNFLSFDDAESQWVAPVDAALPTKRKWDNVPILNQYTKGYLEKECVDWLNKFREYGD : 202
DR2  (ZDA) : VNVNILMDRMRH-NKS--DLHVLQWRHGCEIDSQ-DDDVRFSKGIDEYSYDGENFLSFDDADSQWVAPVDAALPTKRKWDNVPILNQYTKGYLEKECVDWLNKFREYGN : 202
DR1  (ZEA) : VNLKILMERMRHNNNS--DVHVLQWRHGCEIDSQ-GNDVRFSKGIDEYSYDGRNFLAFDDADSQWVAPVEEALPTKRKWDNVPILNQYTKGYLEKECVDWLNKFREYGD : 203
DR5  (ZFA) : VNVHILMDRMRH-NRL--DLHVLQWRHGCEVEIK-GSEVKFSKGIDEYGYDGENFLAFDEAESQWVAPVEEALPTKRKWDSVPILNQYTKGYLEKECVDWLNKFREYAN : 206
DR6  (ZGA) : VNVNILMDRMRY-NKS--DLHILQWRHGCEVEIQ-GSEVKFSKGIDEYGYDGENFLAFDEAESQWVAPVEEALPTKRKWNNIPILNQYTKGYLEKECVDWLNKFREYAD : 180
DR7  (ZHA) : VNVNILMDRMRH-NMS--DLHILQWRTGCEVEIQ-GSEVKFSKGIDEYGYDGENFLSFDEAESQWVAPVKEALPTKRKWDNVPILNQYTKGYLEKECVDWLNKFREYAD : 180
DR8  (ZIA) : VNVHILMERMRH-NMS--DLHILQWRHGCEVEIQ-GSEVKFSKGVSEYGYDGENLLYFDDAESRWVAPVEEALPTKRKWDNIPILNQYTKGYLEKECVDWLNKFREYAD : 209
DR9  (ZJA) : DNVHLLIDRNRQ-STS--VLHVLQWRHGCEVEKQ-GNEHSFNKSIDEYGYDGEDFLYYDDAESRWVAPVEEALPTKRKWDNLPILNQYTKDYLEKECVDWLNKFREYAD : 206
DR30 (ZKA) : VNVHILMDRMRH-NKS--DLHVLQWRHGCEVEIQ-GSEVKFSKGVSEYGYDGENLLSFDETESQWVAPVKEALPTKRKWDNVPILNQYTKGYLEKECVDWLNKFREYAD : 209
AM8  (Z1)  : VSLETVMQRMNH-SNT--DLHVLQWRHGCEIDES-NGEVKFLRGISEYGYDGSDFLSFDRQTMTWIAQVPAAVITKMKWDNVAILTQYTKGYLEKECVDWLTKFLKFRK : 202
AM18 (Z1)  : DFLDTIIQQMNH-SNQ--DLHVLQWRTGCEIEEF-NGTVKFLRGISEYGYDGSDFLSFDIENMRWIAAVPQAEPIMRQWDNETIVNQDAKDSLEKECVDWLTKFLDDRK : 180
AM21 (Z1)  : VNVDILMKRMNH-NST--DLHVLQWRTGCEVEES-NGEVKFLNGIDQYSYDGSDFLSFDNSNSVWIAPVQAAEETKRKWDGVAILNQYMQGYLEKECVSWLKKFMEYGK : 180
AM23 (Z1)  : DFLDTIIQQMNH-SNQ--DLHVLQWRHGCEIDES-SGEVKFLRGISEYSYDGSDFLYFDYENMRWIAAVPQAEPTKRKWDNETIVNQDAKRYLEKECVDWLIKFMEYGK : 182
OL4        : VNIDILINRMRQ-TSN--DTHVLQWMHGCEGVEDEHGNLQFKRGMDMYNYDGDDFLAFDDERQVWVAAADAAVPTKRKWDEVTALKDYTKGYLEKECMEWMKTFLSYST : 200
OL5        : VNIDILINRMRQ-TSN--DTHVLQWMHGCEGEEDEQGNLQFKRGMDMYNYDGDDFLAFDDKHQVWVAAADAAFPTKRKWDDVTALKDYTKGYLEKECMEWMKTFLSYSK : 181
OL17       : VNIDILINRMRQ-TSN--DTHVLQWMHGCEGVEDGHGNLQFMSGVDMYNYDGDDFLAFDDEHQIWVATLDAAVPTKRKWDEVTALKDYTKGYLEKECMEWMNTFLSYSK : 181
GA18       : VNIGILMNRLRQ-NKTS-GQHVLQWQHGCEGEMQLDGTLKFSTGVDMYSYDGYDFLSFDDSNSAWVAGAPAAQQTKRTWDGVDVLKEYTKGYLEKECMEWMEKFLKYQG : 202
ON22       : HNIGILMERMRK-NDSG-SPHVLQWMVGCEGEMQPNGALRFVRGMSKYNYDGNDFLSFDDKNRVWVAPVKEALPSKTKWDNDQVLKEYTKGYLENECIDWLSKSVTSEQ : 201
ON23       : VNIGILMERMRQ-NDSG-TPHILQWMHGCEGETQPDGTLRFVRGMDMYSYDGDDFLSFDGKNEVWVAPTPQALSTKRKWDNQTVLKEYTKGYLENECIDWLSKFVNYGK : 206
ON24       : VNIGILMERMRQ-NDSD-NLHVLQWMHGCEGETNSDGTLRFVRGMDMYSYDGGDFLSFDDKNGVWVAPTPEAEPTKRKWDDVQVLKEYTKGYLENECIDWLSKFVNYGQ : 182
ON33       : VNIGILMERMRQ-NESD-TPHVLQWMHGCEGETHPDGTLKFVRGMDMYNYDGNDFLSFDDKNGVWVAPIDEALPTKRKWDGVQVLKEYTKGYLENECIDWLSKFVTYGQ : 182
ON34       : VNIGILMERMRQ-NDS--DIHVLQWMHGCEGEANPDGTLRFVRGMDMYAYDGDDFLSFDDKNGVWVAPSPEAEPTKRKWDGVLVLKEYTKGYLENECIDWLSKFVTYGQ : 181
ON35       : VNIGILMERMRQ-NDS--DNHVLQWMHGCEGETNPDGTLGFVRGMDMYNYDGNDFLSFDDKNGVWVATTPEAQPTKRKWDGVQVLKEYTKGYLENECIDWLSKFVTYGQ : 200
TN13       : VNIKILKDRFRQ-NDS--DIHVLQWVHGCNIDTS-GNETEFLHGIDMYSYDGESFLTFDEANENWVAPNDAALQTKRKWDDLQVLKEYTKVYLKKECVTWLERFLKYQK : 199
TR21       : VNINILKTRYRQ-NDS--DIHILQWMHGCDADID-HGEPRFLHGIDKYSYDGANFLAFNENQEIWDARDDAARETKTRWDKVQVLREYTMAYLQKECVTWLKRFLDYQN : 203
Amazon_Za  : VNIDILMKRMRQ-NDN--DTHILQWMHGCEGETQPDGSLKFVRGMDMYNYDGSDFLSFDDNHQVWVAPIHAAEETKRKWDEVQVLKEYTKGYLEKECMEWMSKFRGFGK : 182
Amazon_Zb  : VNIDILMKRMRQ-NNT--DTHILQWMHGCEGETQPDGTMKFVRGMDMYNYDGSDFLSFDDNHQVWVAPIHAAEETKRKWDEVQVLKEYTKGYLEKECMEWMAKFRDFGK : 182
Amazon_Zc  : VNIDILMKRMRQ-NNN--DTHILQWMHGCEGETQPDGSLKFVRGMDMYNYDGSDFLSFDDNHQVWVAPIHAAEETKRKWDEVQVLKEYTKGYLEKECMEWMTKFRGFGE : 182
Gar LO14   : VNVGILMDRMRQ-NNS---LHILQWMHGCEIEVQPGSKPQFLRGYDQYSYDGRDFLSFDESKMQWVAPVWPAEPTKHKWDNEQILNQYTKGYLERECVDWLTKFLAFGE : 179
Eel        : VNVKILMERMRQ-NQSDPDVHILQWMHGCQAEKTPDGKLKFHNGFDQYGYDGKDFLNFDSENMAWVAPVPAAESTKTKWDNVPILSQYTKGYLQTDCVHWLEKFLKYQE : 202
Sturgeon   : VNVRILEKRF------------------CRLDED--NNEE-VQGYDQHSYDGNDFLSFDKNHLQWVAPMQPAVATKLKWDGEKILNQYTKGYLENECINWLKMFLGYGE : 148
Lungfish   : VNTQIVMQRR---NDTS-GYHTLQWVHGCSL-TD--GI---KIGIDQYAYDGEDFLSFDKEKLSWIAVNKAAQQTREKWDEEKNLNQYTKRYLEQECIEWLQNFLRFSN : 197
AM3  (Z2)  : KVINIQSS---V-NKS--VGHVLQWNLSCEGER-----HAPVFSSNEFGFDGENLIYFNCTSKTWLTPENKKNPEMEQLWNQQ--YRQLAVEKCVQCEEMLKMYLNYNT : 188
AM4  (Z2)  : SLIDMQLNEFSP-NQS--DGHVLQWRHGCEGEQSSDGSLTVLNSINEFGYDGEDLIQFNCTSKTWITPEENREREREEKWNKD--HQYRADKKCEQCLEMLKMYLKYNT : 201
AM10 (Z2)  : FLLNKQMMVFGH-IES--GDHILHWKYGCEAVEHQNGSMTVFSFTNDYSYDGENFISYDWNLQLWSASVKQAYETEKKWNTEH--HQN---RVLQTCVDWLKIYLKKSP : 194
AM22 (Z2)  : RLLDIQMSEFRH-NQS--DGHVLQWRQGCEVDR----SLTVVKSISELAYDGIELIYFNCTSEMWLTSGKPTDRMLEKWNKDY--RRT-AIKKCEECVRVLEMYLQYRT : 198
AM25 (Z2)  : KLLDIQMDESGH-SNS--DGHVLQWRHRCAGERQSDGSVTVSRSINEFAYDEEELVNYNCTSNTWYNSD-NQKKEEIEKLSAV------LMKKCSQCEEMLKIFLQHST : 171
caauZA1(Z3): DLIRILSNRTES---S--DLLVLQRVIGCELEKLPDGAVN-LTPVDEYGFGGEDCVDFSSDTSQCSDKSLNVKETKIKRDHRVK-----LQEFFKNCLDWISTFNNTKK : 158
cycaZB1(Z3): HQIRILSNCTDS-LCS--ELHVLQRVIDCEREKLSDGSVN-LRAFDEYGFDGEDFMAFDSETLQWIDKHPKAKETKIKWDQQTERIQ-FLKQYLKNCMNWISTFNNTKK : 162
cycaZC1(Z3): DLIRILSNCTEC---S--ELHVLQRISGCELEKLPDGAVTSLKVFDEFVYDGKDFISCKYDFFPWMDK---VIETEMN--HQTGQNP-FLKDFLINCTKWISTFNNTYK : 158
```

```
                      Alpha 3 domain                                                                                 CP
                   180       *         200       *         220       *         240       *         260       *
HLA-A2       : ETLQRT---DAPKTHMTHHAVS--DHEATLRCWALSFYPAEITLTWQRDG-EDQTQDT-ELVETRPAGDGTFQKWAAVVVPSGQEQ---RYTCHVQHEGLPKPLTLRWE : 275
sasaUBA      : STLMRT---VPPSVSLLQKTPS-----SPVTCHATGFYPSGVMVSWQKDG-QDHHEDV-EHGETLQNDDGTFQKSSHLTVT-PEEWKNNKYQCVVQVTGLQEDFIKVLT : 290
sasaZAAa     : KEFSWAD--SAPKVYAFAKKAK-TAGHVRLTCMATGFYPKDVVMHIKKNGVPLTDRDGVQSAGLLPNDDETYQIRMSVQIP---EADKETYECYVNHRALKEPIVVKWD : 306
sasaZBAa     : KEFSRAD--SAPKVYAFAKKAK-TAGHVRLTCMATGFYPKDVEMNIKKNGVPLTKHDGVQSAGVLPNDDETYQIRMSVQIP---EADKETYECYVHHRTLEEPIVIKWD : 284
sasaZCAa     : KHLRMDD--SAPKVYAFAKKAK-TAGHVRLTCMATGFYPKDVVMHIKKNGVPLTKHDGVQSAGVLPNDDESYQIRMSVQIP---EADKETYECYVYHRTLEEPIVEKWD : 309
sasaZDAa     : KEFSRPD--SAPKVYAFAKKAK-TAGHVRLTCMATGFYPKDVVMHIKKNGVPLTDRDGVQSAGVLPNDDETYQIRMSVQIP---EADKETYECYVHHRTLEKPIVIKWD : 284
sasaZBAb     : KHFSSTD--SPPNIYVFTKKAK-PAGNVHLTCMVTGFYPKDVIIHFKKNGVQLTEDDGVLSTGARPNNDDTYQIRISVQIP---EADKDMYECSVSHAMLKEPIVEKW- : 305
sasaZCAb     : KHFSSAD--SPPDINVFANKAK-TAGNVHLTCMATGFYPKDVIIHFKKNGVQLTEDDGVLSTGARPNNDDTYQIRISVQIP---EADKQTYECSVSHITLVQPIVVKW- : 305
DR4  (ZBA)   : KELREG---SSPEVHVFAKRIINGKIKLKLTCLATGFYPKDVILNIRKYRITLPDNE-VESTGVRPNEDGTFQLRKSINIY---EDEKAEYDCYVSHTTLKEPIIKKWD : 304
DR3  (ZCA)   : QELRDG---SPPDVYVFARRIISGKIKLKLTCMATGFYPKDMILTIRKYRTTLPDND-LDSSGVRPNQDGTFQLRKSTNIY---EDEKAEYDCYVNHRTLKEPIIVRWN : 304
DR2  (ZDA)   : EELREG---SPPDVHVFARSIINGKAKLKLTCLATGFYPKDVYLTIRKYRMALLDKD-LESSGVRPNHDGTFQLRMSTYIY---EDEKAEYDCYVKHRTLGAPIIKKWD : 304
DR1  (ZEA)   : QELREG---SPPDVHVFAKKIISGKAKLKLTCMVTGFYPKDVILTIRKYRTALSDNE-VESSGVRPNPDGTFQLRKSTNIY---E--KAEYDCYVAHRTLKEPIIKKWD : 303
DR5  (ZFA)   : EELRNG---SPPEVHVFAVKSISDKTKLKLTCLATGFYSKDTMLVIRRN--RLPEKK-TESTGVRPNHDQTFQLRKSVEIE---QDETDEYDCYMTHRTLKGPVIARWD : 306
DR6  (ZGA)   : EELRKA---SSPDVYKLTKKSTKDETKLKLTCLATGFYDKDVMLNIRRN--CLPEDE-TESTGVRPNHDQTFQLRKSVEIK---EDQIDEYDCHLTHRTLKNPVTATR- : 279
DR7  (ZHA)   : EELRNG---SSPDVYKLTKKSTKDETKLKLTCLATGFYDKDVMLNIRRN--CLPEDE-TESTGVRPNHDQTFQLRKSVEIK---EDQIDEYDCHLTHRTLKNPIIVTQ- : 279
DR8  (ZIA)   : EELRKA---SPPDVYKLTKKSTKDETKLKLTCLATGFYPNDVMLNIRRN--CLPEDE-TESTGVRPNHDQTFQLRKSVEIK---EDQIDEYDCHLTHRTLKNPVTATR- : 308
DR9  (ZJA)   : EELRKA---SPPDVFKLTRKSTKDETKLKLTCLATGFYPNDVMLNIRRYRVYLPEQE-TISTGVRPNHDQTFQLSKSVEIK---EDQIDNYDCSVSHRTFKQPIIIKWD : 308
DR30(ZKA)    : AELRNG---SPPDVYKFTKKSTKDETKLKLTCLATGFYPSDVMLNIRRNRVYLPEQE-TISTGVRPNHDQTFQLSKSVEIK---EDQIDEYDCSVSHRTFKQPIIIKWD : 311
AM8  (Z1)    : ESQQKD---APLDVQVFAKPSVSDSSKLTLTCLATGFYPKDATVIWRRSSSPLSEDL-ITSSAVRPNDDGTYQLRKSVEIL---GAEKDQYECYVSHRTLKEPVIKKLG : 304
AM18 (Z1)    : ETLRNH---SSPAVHGFVKKSVRDPKKLTLTCLITGFYPKDVKMSLRKFTTEIPEHL-ITSSGVRPNDDGTYQLRKSVEIS---GDDPTDYDCYLPHSSFKKPVMKKWV : 282
AM21 (Z1)    : ETLRK-------------------------------------------------------------------------------------------------------- : 185
AM23 (Z1)    : ETLRKH---SPPAVYGFAKKSVSDSKKVSLTCLATGFYPKDVDLSVRKFGTSIPDHL-ITTSGVRPNEDGTYQLRKSAEIS----DDPTDYDCNVHHSTIEEPAIKKWE : 283
OL4          : QQLRNA---SRPDVYMFFKKAK-ESSNVVLTCLATGFYPKDITLNIRRDGRVLTKDDGVMSSGVRPNHDETFQRKDYVEIL---RSDSATYTCEIIHPASNVWVVKTWE : 302
OL5          : QQLRNA---SRPDVYMFFKKAK-ESSNVVLTCLATGFYPKDITLNIRRDGRVLTKDDGVMSSGVRPNHDETFQRTDYVEIL---RSDSANYTCEIIHPASDMHVVKTWD : 283
OL17         : QQLKSA---SSPDVFLFASEAK-EKANVVLTCLATGFYPKDITMNIRRDGRILTKDDGVVSSGVRPNQDDTHQRRDHVEIL---RTDVASYTCEIIHPGSNMHVEKPWD : 283
GA18         : SVLENA---TKPEVYLFASKAK-KEANVILTCMATGFYPKEIQLWIKRNGRVLRREDGVMSSGSRPNGDETFQRKDWVEIL---KTDQSQYTCEVIHKATQVNIEKEWD : 304
ON22         : KQLKKA---PPPGVYVFAKKSR-VETNLTLTCLATGFYSKNIILRIRRKGRVLTEDDGLWSSGVLPNDDETFQRRDYVEIL---KSDLSEFSCEVVHEATRVDVVKTWK : 303
ON23         : EKLK-----SVPEVYVFAKCST-VDTNVVLTCLATGFYPAELTVTIRRNGRVLTADDGLMSSGLLPNHDETFQRRDNVEVL---KSDVSVFSCEVRHEATNEHAVKDWD : 306
ON24         : QQLKKK---SPPELHVFAKNSR-VQTNIVLTCLATGFYPKDVIMRIRRNGRVLTADDGLKSSGPLPNNDDTFQRREYVEVL---KSDTSPYSCEVFHKATNVSLSESWD : 284
ON33         : QQLKKK---SPPEVFVFAKKSK-VESNLILTCLATGFYPKDIIMRIRRNGRVLTADDGLTSSGVLPNNDETFQRRDHVEI----------------------------- : 258
ON34         : QQLQNT---SPPEVSLHAKTST-VDTDVLLTCLATGFYPADIVLRMKKNESVLTADDGLMSSGVLPNEDHTFQRRDHVEV----------------------------- : 257
ON35         : KQLKKK---SPPDVHVFTKKAK-VESNLILTCLATGFYPKDIIVKIRRNGRVLTADDGLTSSGVLPNNDETFQRRDHVEIL---KSDLSEFSCEVIHEATGVDVAKTWT : 302
TN13         : ENTAP----VKPAVYALSREAN-VQANIVLTCMATGFSSINTIVQIKRDGLVLTKDDG----DVRPNGDGTYQKRDQVEIP---KSDKSKYTCEVIHESSGLHEIRVWD : 296
TR21         : KNEIP----AKPDLYVFITGAK-DPTNMVLKCMATGFTPTNTVLQIKLGGRVLTREDGVNSTDILPNGDGTFQKTDYVEIP---RSDNSDYYCELSHTPSSLRVVKFWD : 304
Gar LO14     : QQIKKS------------------------------------------------------------------------------------------------------- : 185
Eel          : LDFQDS---SPPDVYIFSKKST-SPDYRTLVCLATGFYPKDVAVDILKDGAPLAESDGVESSGVRTNGEPKETFQLRKWLE-IKTSDTSKYSCRVKHQTLRSPIVKFWD : 306
Sturgeon     : RDIYRS------------------------------------------------------------------------------------------------------- : 154
Lungfish     : GKLEKK---VGPETKIFRKRRH-D-GKYTFSCHASEFFPSALSIKWINIKTNEKKEE---TAEALPNNDGTFQAWVDIDSD---LECARDLVCMITHGNSSEE-HRIPA : 294
AM3  (Z2)    : TDLTPSH--SPT-VYIFQNKSVSDSSELILTCMATGFYPKDVKMSLMKSGTEIPDHL-ITSSGVRPNHNGTYQLRKSVEIQ---EDEKDQYECYVSHSSLTEPKIEKWE : 290
AM4  (Z2)    : TDITPKH--TPPAVFIFAKKSKNDLKKLNLTCLITGFYNRDVKMSLRKLTTEIPDHL-ITSSGFRLNDDETYQLRKSVEIQ---EDDSVDYSCYVSHSSLTEPVIKQWV : 304
AM10 (Z2)    : EIEK-----KPPVVSMFITSSPPGPNLDSLTCVATGFYPKYLKMILRISGVPISEDQ-IKSTGVRPSGDGTYNLSKTVEVL---GHEKFEYDCYVTHSSLEEPIIKNV- : 293
AM22 (Z2)    : ANTTDIN-TTPPAVHVFANKSVRDSRKLTLTCLITGFYPKDVKMSLRKSGTSLPDHL-ITSSGIRPNDDGTYQLRESVEIQ---EDDKAQYDCYVSHSSLQTPVIKKWD : 302
AM25 (Z2)    : FNITPSQ-SAPPAVHVFSKKSVHDSSKQILTCLATGFYPKDVKMSLRKSGTSLPEHL-ITSSAVRPNDDETYQLRKSVEIQ---EDDPADYDCYVNHSSLQTPVIKQWD : 275
caauZA1(Z3)  : N--------SP-DVGVFARKAPDDHSKLVLICLVTGFYPRDIEMNIRLNR-INIQN--QISSGVRPNDDETFQMRSSVKID---RNHRGSYDCHVIHSSLTE------- : 245
cycaZB1(Z3)  : H--------SP-DVCVFARRAPDDHSKLVLICLVTGFYPRDVEMNIRLNR-INIQN--QISSGIRPNDDESFQMRSSVKID---RNHRGSYDCHVIHSSLTE------- : 249
cycaZC1(Z3)  : N--------SP-DVHVFARKAPDDHSKLVLICLATGFYPRDIEMNIRLDR-TALGN--QIFSEIRPNADGSFQLRSSVKID---RNHKGSFDCFVNHSSLTK------- : 245
```

```
                                 TM                   CYT
                    280                 *         300       *         320       *         340
HLA-A2      : PSSQPTIP---------IVGIIAGLVLFGAVITGAVVAAVMWRRKSSDRKGGSYSQAASSDSAQGSDVSLTACKV---------------------------------- : 341
sasaUBA     : ESEIKTNWNDPN-----IVLIIGVVVALLLVVVAVVVGVVIWKKKS--KKGFVPASTSDTDSDNSGRAAQMT------------------------------------- : 355
sasaZAAa    : G-KCCDCSSGG--------AVVIGAVVIAFIVVLILVGLFVLHRRGTIGRS---------------------------------------------------------- : 348
sasaZCAa    : G-KFYDCNQ--------VTGVIIGVVAVVLLFIVVTPLLVLWKKGK--------------------------------------------------------------- : 346
sasaZDAa    : G-ICCDCSS--------FNAVVIGAVVITFIVVLILVVLFVLHRRGTIVIPGLRTTATGNGVAFSGVNTS--------------------------------------- : 345
DR4  (ZBA)  : G-ECLDGPESGSP----IGIIAGAIIVVLVVLAAIGGAVYFLRKRSGNNNVKPSSVPTISGNKDEKCSMLPGSDDSGQGSSDGSSKSSPTNSQEKMDIV---------- : 398
DR3  (ZCA)  : GEYLSEPP---------IAIIA-AIIGVLILLVAIGVTVWILKKKNII------------GNKDEKRSMPNGSANYGRGSSA--------------------------- : 364
DR2  (ZDA)  : G-KCSDCSK----VTFLGMIVGAIIGAVLVLTVIGLVILVLTRKKAPKKPFYKNGIGDNDPSAIPLNNNQHIEPSVKESKNKPTAEGQSTSGDLKS------------- : 395
DR1  (ZEA)  : G-ECQDCSSGTP--IGTIFGALIGVLLVLAVIGGAVYFLANTRMGWRNAL----------------------------------------------------------- : 350
DR5  (ZFA)  : G-KCKDCLPN--------LNWIWVVAGAVLMLGVVALLLVLLKKKIIDLRQRLSGSQSPLYQIQADSESEDYNH----------------------------------- : 371
DR9  (ZJA)  : GTDLDGIYTGHPPET----VPVVIGSVLIFLAILGVVVFLVMKCGEQDSSSTDTTECTSILLGFIADHLNKELTEGENW------------------------------ : 383
DR30(ZKA)   : GTDLDGKYTGYPPET--VPVVIGSVLILLAILAVVVFLVMNYDGECCKGLFIHFTLLKDCGLLLPPCGKTEYV------------------------------------ : 382
AM8  (Z1)   : GAP-INTDLPEELGMLPPF------------------------------------------------------------------------------------------ : 322
AM18 (Z1)   : NTKNSAGT-----------ALIGGAAVGVMVILLGVVLTVLIKKRKNDTDRQNLHKGCRNFVYSREETSRSENIEH--------------------------------- : 347
AM23 (Z1)   : Q-KC-LTNPKTGSG--LVLIGGVAGGVIVILLIVLAVLFFVLKKKRMNGEYK--------------------------------------------------------- : 331
OL4         : GSPGSD----------------IGLVLGLVCGIGGVLAIVVIRVLYKKK------------------------------------------------------------ : 335
OL5         : H-HLPPVDKNN-------LGLYVGIVIGLLVLILGSIIVFLV------------------------------------------------------------------- : 317
OL17        : HRLPTEEN-------------FPIAPTAAIAVILVLIGIILF------------------------------------------------------------------- : 312
GA18        : HKLPENGSPIG--------VAVGVPVLVLVLAVVAGVLIFCYRKASSTSSTSSTSSTSSTSSTSSSNTDSIS------------------------------------- : 368
ON22        : NGDHPEEPGSG---ALIG-AQGGALAVIALVVG-GGLGLICLHKKGR-------------------------------------------------------------- : 345
ON23        : DRLLPDSEGSSVH------ILVAAVVVPFVLVGVAAVLLFLYKNLNKRCWS---------------------------------------------------------- : 351
ON24        : LSMLPVPDDDGS-------AVIGLVAALLLVLIGVGVILLVLRKRRINGRWNEQ------------------------------------------------------- : 331
ON35        : NKCEEVSGSGSGALIGGAVVGVVVVVAVAVGLILYKMGIIGGRRGAKDNQGGIQTIYS--------------------------------------------------- : 360
TN13        : TDEG------NT----AVVVGAVVGVVVVVVLVIGIGLYMAVKK----------------------------------------------------------------- : 330
TR21        : KKAPDDTTK----------ILLAVRISLGAVILIGGLLILVIKKFCIG------------------------------------------------------------- : 342
Eel         : GKCHNCDGDNTG------AIVGAVVGVLLIVLVAVLVGLYFLRKKMNEKEKAAQAAANGLIQPLTGVSTEGNGSNSSVQSGDSGRGSTDSGEPKEVTKPFIVNGAIA-- : 407
Lungfish    : EPLRSRK----------VFLYIIIIAVVLLLLSAAVLLLLFLKKKKKCAYANEVIAENYLEEAGLPAVAKDAVKDETADLLIKDEKLLLLLLTKLQIKMKQVTH----- : 388
AM3  (Z2)   : D-KV----------SVGFIVGVAVAVVAVVILFCGLVLFCALKNRKKKRE----------------------------------------------------------- : 329
AM4  (Z2)   : G-RCTDCPDNAYQS--LIIGVVVLGGLAAIYFLILGVLMMIIKQKSGEQSAC--------------------------------------------------------- : 353
AM22 (Z2)   : G-KCRNCTSGRSWTG---FIGVAVLVVAGILFICVLIC----------------------------------------------------------------------- : 336
AM25 (Z2)   : K-KCSNWTDGRSWTG---------LIIVLILVVLVFICVLIYKKPQCVKYSSAPSDPGSPDELPSNSNGGVPNGK---------------------------------- : 340
```

Text S4b. Residue conservation of the α1 and α2 domains among MHCI molecules

Three-dimensional structure of the α1 and α2 domains of HLA-A2 (Protein Data Bank ID: 3hla) is shown by the main chain trace. The positions of conserved and divergent amino acid residues among three sequences are indicated by red and yellow circles, respectively. The positions of identical residues between two of three sequences are indicated by orange circles. The residues of HLA-A2 interacting with peptide ligand [main text reference 1, 3] are shown by larger circles. Ellipses cover those HLA-A2 residues that, according to our sequence alignments, have no matching residues in the three sequences whose information is depicted in the respective figures. Individual figures show the following: (A) Comparison among typical Z lineage sequences of zebrafish (*Danio rerio* DR1, Text S2), gar (*Lepisosteus oculatus* LO14, Text S2), and lungfish (*Protopterus aethiopicus*, AAF15304.1). (B) Comparison among teleost classical U lineage sequences of salmon (*Salmo salar* UBA*0301, AF504022), medaka (*Oryzias latipes* BAD93265), and sturgeon (*Acipenser sinensis* ACV87428). (C) Comparison among the tetrapod classical MHCI sequences of human HLA-A2 (AAA76608.2), chicken (ADZ31379), and frog (*Xenopus laevis*, AF185583). (D) Comparison among HLA-A allelic sequences of A*0201 (IMTG/MHC Acc No. HLA00005), A*0101 (HLA00001), and A*2301 (HLA00048).

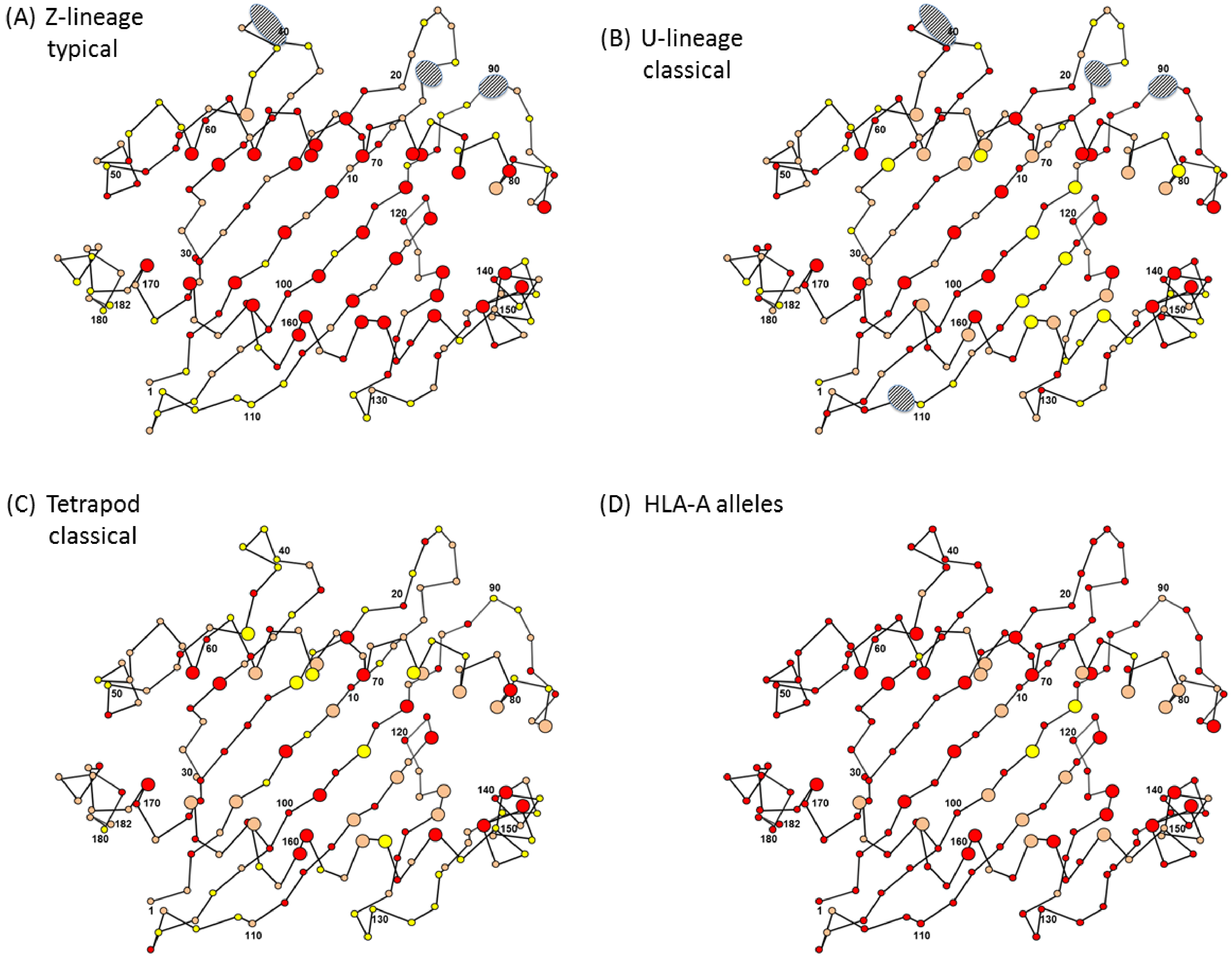
(A) Z-lineage typical
(B) U-lineage classical
(C) Tetrapod classical
(D) HLA-A alleles

Text S4c. Percent identity per domain between deduced Z lineage amino acid sequences

Alpha 1 domain

	1	2	3	4	5	6	7	8	9	10	11	12	13	14	15	16	17	18	19	20	21	22	23	24	25	26	27	28	29	30	31	32	33	34	35	36	37
1: AM2 (Z2)	100	81	78	80	54	49	31	26	31	30	30	30	31	31	32	30	31	31	31	30	28	30	30	31	36	36	39	35	38	38	38	38	35	33	24	18	25
2: AM4 (Z2)	81	100	73	76	54	47	31	26	28	30	30	31	31	31	33	30	34	34	34	34	31	31	31	30	34	34	35	33	34	34	34	35	31	29	25	18	26
3: AM25 (Z2)	78	73	100	76	49	51	37	31	34	34	34	34	34	33	36	34	32	32	32	33	31	31	31	30	39	40	41	40	40	39	40	40	38	33	25	19	24
4: AM22 (Z2)	80	76	76	100	56	50	31	27	31	32	32	33	34	33	34	31	34	34	34	33	31	31	31	33	40	40	42	40	40	40	40	40	38	31	21	17	22
5: AM3 (Z2)	54	54	49	56	100	37	28	22	27	30	29	27	33	34	34	33	29	29	29	27	27	28	29	29	41	41	41	38	38	36	38	40	35	28	22	16	25
6: AM10 (Z2)	49	47	51	50	37	100	35	28	36	34	32	34	34	36	38	35	32	32	33	33	33	33	33	33	41	43	40	40	41	41	40	41	38	32	24	17	22
7: AM18 (Z1)	31	31	37	31	28	35	100	74	79	54	53	55	55	55	57	56	54	54	55	54	51	52	53	51	66	65	64	68	66	64	66	64	64	57	22	22	21
8: AM23 (Z1)	26	26	31	27	22	28	74	100	57	41	40	42	42	42	44	43	42	42	42	41	42	43	43	40	49	49	47	52	51	48	51	49	48	43	15	18	15
9: AM8 (Z1)	31	28	34	31	27	36	79	57	100	66	64	65	66	65	67	66	60	60	62	60	62	62	62	58	74	73	70	77	70	69	70	68	70	59	21	21	19
10: sasaZDAa	30	30	34	32	30	34	54	41	66	100	97	96	94	93	93	93	68	68	69	68	72	72	71	68	74	77	73	73	72	69	71	71	71	57	25	26	26
11: sasaZBAa	30	30	34	32	29	32	53	40	64	97	100	92	92	91	92	92	66	66	67	67	71	71	70	67	73	76	72	72	72	69	71	71	71	57	25	26	26
12: sasaZAAa	30	31	34	33	27	34	55	42	65	96	92	100	90	89	89	90	68	68	69	68	74	73	71	67	73	74	70	73	69	68	69	68	69	55	25	26	26
13: sasaZCAb	31	31	34	34	33	34	55	42	66	94	92	90	100	96	96	92	68	68	69	67	73	73	74	69	76	77	73	75	70	69	69	70	69	55	23	25	26
14: sasaZBAb	31	31	33	33	34	36	55	42	65	93	91	89	96	100	97	93	66	66	67	65	71	73	73	69	76	77	73	75	70	69	69	70	69	56	26	27	29
15: sasaZDAb	32	33	36	34	34	38	57	44	67	93	92	89	96	97	100	96	68	68	69	67	74	75	75	70	78	79	75	77	73	71	71	73	71	57	25	26	27
16: sasaZCAa	30	30	34	31	33	35	56	43	66	93	92	90	92	93	96	100	67	67	68	66	73	74	74	68	76	78	74	75	73	69	71	71	71	57	25	26	27
17: OL4	31	34	32	34	29	32	54	42	60	68	66	68	68	66	68	67	100	100	99	95	74	74	73	69	70	71	66	71	65	64	65	65	66	59	26	23	23
18: OL5	31	34	32	34	29	32	54	42	60	68	66	68	68	66	68	67	100	100	99	95	74	74	73	69	70	71	66	71	65	64	65	65	66	59	26	23	23
19: OL17	31	34	32	34	29	33	55	42	62	69	67	69	69	67	69	68	99	99	100	96	73	73	74	69	71	73	67	73	66	65	66	66	67	60	25	23	23
20: OL7	30	34	33	33	27	33	54	41	60	68	67	68	67	65	67	66	95	95	96	100	73	73	73	67	69	70	65	70	64	63	64	64	65	58	25	23	23
21: ON23	28	31	31	31	27	33	51	42	62	72	71	74	73	71	74	73	74	74	73	73	100	96	93	85	71	70	65	69	68	68	68	67	66	58	26	26	27
22: ON24	30	31	31	31	28	33	52	43	62	72	71	73	73	73	75	74	74	74	73	73	96	100	92	84	73	71	66	70	69	69	69	68	67	59	26	26	29
23: ON35	30	31	31	31	29	33	53	43	62	71	70	71	74	73	75	74	73	73	74	73	93	92	100	84	74	73	67	71	69	69	69	68	67	59	22	23	27
24: ON22	31	30	30	33	29	33	51	40	58	68	67	67	69	69	70	68	69	69	69	67	85	84	84	100	69	68	63	67	67	66	67	66	65	59	26	27	30
25: DR4 (ZBA)	36	34	39	40	41	41	66	49	74	74	73	73	76	76	78	76	70	70	71	69	71	73	74	69	100	96	90	92	88	87	88	87	87	74	23	23	27
26: DR2 (ZDA)	36	34	40	40	41	43	65	49	73	77	76	74	77	77	79	78	71	71	73	70	70	71	73	68	96	100	91	91	87	84	87	88	87	73	23	23	27
27: DR3 (ZCA)	39	35	41	42	41	40	64	47	70	73	72	70	73	73	75	74	66	66	67	65	65	66	67	63	90	91	100	88	85	81	84	85	85	70	23	23	26
28: DR1 (ZEA)	35	33	40	40	38	40	68	52	77	73	72	73	75	75	77	75	71	71	73	70	69	70	71	67	92	91	88	100	85	85	85	82	87	71	23	23	26
29: DR7 (ZHA)	38	34	40	40	38	41	66	51	70	72	72	69	70	70	73	73	65	65	66	64	68	69	69	67	88	87	85	85	100	96	98	96	93	80	26	26	30
30: DR8 (ZIA)	38	34	39	40	36	41	64	48	69	69	69	68	69	69	71	69	64	64	65	63	68	69	69	66	87	84	81	85	96	100	96	91	91	78	26	26	30
31: DR30 (ZKA)	38	34	40	40	38	40	66	51	70	71	71	69	69	69	71	71	65	65	66	64	68	69	69	67	88	87	84	85	98	96	100	96	95	81	26	26	30
32: DR6 (ZGA)	38	35	40	40	40	41	64	49	68	71	71	68	70	70	73	71	65	65	66	64	67	68	68	66	87	88	85	82	96	91	96	100	92	78	26	26	30
33: DR5 (ZFA)	35	31	38	38	35	38	64	48	70	71	71	69	69	69	71	71	66	66	67	65	66	67	67	65	87	87	85	87	93	91	95	92	100	81	25	25	27
34: DR9 (ZJA)	33	29	33	31	28	32	57	43	59	57	57	55	55	56	57	57	59	59	60	58	58	59	59	59	74	73	70	71	80	78	81	78	81	100	27	27	29
35: caauZA1 (Z3)	24	25	25	21	22	24	22	15	21	25	25	25	23	26	25	25	26	26	25	25	26	26	22	26	23	23	23	23	26	26	26	26	25	27	100	64	58
36: cycaZC1 (Z3)	18	18	19	17	16	17	22	18	21	26	26	26	25	27	26	26	23	23	23	23	26	26	23	27	23	23	23	23	26	26	26	26	25	27	64	100	54
37: cycaZB1 (Z3)	25	26	24	22	25	22	21	15	19	26	26	26	26	29	27	27	23	23	23	23	27	29	27	30	27	27	26	26	30	30	30	30	27	29	58	54	100

Alpha 2 domains

	1	2	3	4	5	6	7	8	9	10	11	12	13	14	15	16	17	18	19	20	21	22	23	24	25	26	27	28	29	30	31	32	33	34	35	36	37
1: OL4	100	93	85	88	61	61	58	54	47	51	51	55	54	55	58	54	56	55	54	53	54	55	50	50	53	50	49	49	25	38	27	30	27	21	22	28	21
2: OL5	93	100	86	85	64	64	61	56	48	51	51	55	54	56	59	54	56	55	54	54	54	56	51	51	54	51	50	50	27	39	29	29	28	22	21	29	21
3: OL7	85	86	100	78	59	59	57	52	46	50	52	53	52	53	54	51	55	51	50	51	53	52	48	47	50	47	47	47	24	36	26	31	25	19	22	25	20
4: OL17	88	85	78	100	59	60	57	52	44	49	51	55	54	55	58	55	58	56	54	52	54	55	49	50	52	50	50	49	27	38	28	30	26	21	21	26	21
5: ON24	61	64	59	59	100	91	81	72	48	54	52	61	60	60	61	59	61	60	58	60	59	60	54	54	55	53	53	54	31	42	33	30	29	22	21	29	25
6: ON35	61	64	59	60	91	100	79	76	46	52	52	60	60	57	60	60	63	61	55	59	56	56	53	53	54	52	52	53	30	39	32	29	27	20	22	27	24
7: ON23	58	61	57	57	81	79	100	72	50	55	52	62	61	58	63	62	62	63	56	60	60	59	56	53	53	55	54	53	31	42	35	33	27	21	22	29	24
8: ON22	54	56	52	52	72	76	72	100	46	47	50	53	53	56	53	54	55	55	52	56	52	53	52	53	49	51	53	52	26	33	28	31	24	20	22	27	22
9: AM18 (Z1)	47	48	46	44	48	46	50	46	100	80	68	50	51	50	52	47	52	50	52	55	55	54	58	56	56	54	55	54	29	41	35	33	31	21	27	27	28
10: AM23 (Z1)	51	51	50	49	54	52	55	47	80	100	71	60	61	58	61	57	63	57	63	63	65	63	60	60	61	61	64	61	30	43	33	34	30	20	30	29	27
11: AM8 (Z1)	51	51	52	51	52	52	52	50	68	71	100	57	58	56	57	54	59	57	58	59	63	60	60	60	60	59	59	56	32	43	32	36	34	20	31	29	28
12: sasaZDAa	55	55	53	55	61	60	62	53	50	60	57	100	94	90	92	92	90	89	71	69	71	69	65	63	65	65	63	59	28	38	28	33	28	16	24	28	22
13: sasaZBAa	54	54	52	54	60	60	61	53	51	61	58	94	100	90	88	90	88	89	73	71	72	70	67	64	66	68	65	63	28	38	29	32	26	16	24	27	22
14: sasaZAAa	55	56	53	55	60	57	58	56	50	58	56	90	90	100	85	85	86	84	70	68	69	68	64	61	64	64	60	60	29	37	29	30	28	17	27	29	23
15: sasaZCAa	58	59	54	58	61	60	63	53	52	61	57	92	88	85	100	88	86	84	70	69	70	69	65	63	65	65	63	59	31	40	31	33	28	17	22	28	22
16: sasaZCAb	54	54	51	55	59	60	62	54	47	57	54	92	90	85	88	100	93	90	67	65	67	65	63	60	63	64	61	60	29	37	29	33	24	15	22	26	19
17: sasaZBAb	56	56	55	58	61	63	62	55	52	63	59	90	88	86	86	93	100	89	72	70	72	70	66	65	68	66	63	64	32	39	32	30	29	17	28	29	24
18: sasaZDAb	55	55	51	56	60	61	63	55	50	57	57	89	89	84	84	90	89	100	69	67	68	67	66	64	65	67	65	63	28	37	29	33	26	16	26	28	21
19: DR1 (ZEA)	54	54	50	54	58	55	56	52	52	63	58	71	73	70	70	67	72	69	100	95	91	90	81	79	82	81	78	76	27	35	27	29	27	21	28	31	27
20: DR4 (ZBA)	53	54	51	52	60	59	60	56	55	63	59	69	71	68	69	65	70	67	95	100	89	91	83	81	82	81	79	77	29	36	29	30	29	22	26	32	29
21: DR2 (ZDA)	54	54	53	54	59	56	60	52	55	65	63	71	72	69	70	67	72	68	91	89	100	89	82	79	82	79	77	74	31	38	31	33	29	22	28	29	29
22: DR3 (ZCA)	55	56	52	55	60	56	59	53	54	63	60	69	70	68	69	65	70	67	90	91	89	100	85	83	83	82	80	79	29	35	30	30	27	22	27	30	26
23: DR7 (ZHA)	50	51	48	49	54	53	56	52	58	60	60	65	67	64	65	63	66	66	81	83	82	85	100	93	93	93	89	80	31	38	30	34	27	23	28	27	26
24: DR30(ZKA)	50	51	47	50	54	53	53	53	56	60	60	63	64	61	63	60	65	64	79	81	79	83	93	100	90	88	90	77	32	36	29	30	31	27	33	29	29
25: DR5 (ZFA)	53	54	50	52	55	54	53	49	56	61	60	65	66	64	65	63	68	65	82	82	82	83	93	90	100	92	86	80	32	40	30	30	31	24	28	30	28
26: DR6 (ZGA)	50	51	47	50	53	52	55	51	54	61	59	65	68	64	65	64	66	67	81	81	79	82	93	88	92	100	93	83	30	38	29	34	30	23	27	30	26
27: DR8 (ZIA)	49	50	47	50	53	52	54	53	55	64	59	63	65	60	63	61	63	65	78	79	77	80	89	90	86	93	100	84	30	34	26	31	30	26	30	29	26
28: DR9 (ZJA)	49	50	47	49	54	53	53	52	54	61	56	59	63	60	59	60	64	63	76	77	74	79	80	77	80	83	84	100	30	38	32	31	32	26	28	31	26
29: caauZA1 (Z3)	25	27	24	27	31	30	31	26	29	30	32	28	28	29	31	29	32	28	27	29	31	29	31	32	32	30	30	30	100	61	53	20	26	20	15	19	16
30: cycaZB1 (Z3)	38	39	36	38	42	39	42	33	41	43	43	38	38	37	40	37	39	37	35	36	38	35	38	36	40	38	34	38	61	100	54	24	27	24	15	22	21
31: cycaZC1 (Z3)	27	29	26	28	33	32	35	28	35	33	32	28	29	29	31	29	32	29	27	29	31	30	30	29	30	29	26	32	53	54	100	24	27	22	19	23	21
32: AM10 (Z2)	30	29	31	30	30	29	33	31	33	34	36	33	32	30	33	33	30	33	29	30	33	30	34	30	30	34	31	31	20	24	24	100	28	26	24	29	21
33: AM4 (Z2)	27	28	25	26	29	27	27	24	31	30	34	28	26	28	28	24	29	26	27	29	29	27	27	31	31	30	30	32	26	27	27	28	100	61	57	52	38
34: AM3 (Z2)	21	22	19	21	22	20	21	20	21	20	20	16	16	17	17	15	17	16	21	22	22	22	23	27	24	23	26	26	20	24	22	26	61	100	49	37	33
35: AM22 (Z2)	22	21	22	21	21	22	22	22	27	30	31	24	24	27	22	22	28	26	28	26	28	27	28	33	28	27	30	28	15	15	19	24	57	49	100	34	31
36: AM2 (Z2)	28	29	25	26	29	27	29	27	27	29	29	28	27	29	28	26	29	28	31	32	29	30	27	29	30	30	29	31	19	22	23	29	52	37	34	100	26
37: AM25 (Z2)	21	21	20	21	25	24	24	22	28	27	28	22	22	23	22	19	24	21	27	29	29	26	26	29	28	26	26	26	16	21	21	21	38	33	31	26	100

Alpha 3 domains

	1	2	3	4	5	6	7	8	9	10	11	12	13	14	15	16	17	18	19	20	21	22	23	24	25	26	27	28	29	30	31	32	33	34	35	36	37
1: sasaZAAa	100	94	91	92	88	69	69	51	51	48	49	44	44	44	44	40	43	49	49	47	47	43	46	49	47	36	42	41	37	48	47	45	41	45	47	48	46
2: sasaZDAa	94	100	94	94	88	70	66	51	50	50	49	45	45	45	45	41	44	49	49	45	45	44	49	49	47	38	42	40	37	48	47	46	41	45	47	45	45
3: sasaZBAa	91	94	100	96	89	69	66	51	50	49	50	47	47	47	47	42	44	49	50	47	47	44	51	50	48	39	45	43	38	51	49	48	41	45	47	46	45
4: sasaZCAa	92	94	96	100	91	69	66	50	49	48	48	44	44	44	44	40	43	48	49	45	45	43	48	49	47	38	42	42	37	48	47	46	40	44	46	45	44
5: sasaZDAb	88	88	89	91	100	71	70	52	51	49	50	45	45	47	49	44	45	49	49	47	47	43	46	49	49	36	43	43	38	47	46	45	41	45	47	44	46
6: sasaZCAb	69	70	69	69	71	100	87	49	48	47	48	44	44	45	48	45	44	49	45	41	41	39	44	45	47	36	38	37	36	46	45	47	42	43	48	41	48
7: sasaZBAb	69	66	66	66	70	87	100	50	47	42	48	47	47	48	49	44	43	47	45	42	42	41	44	47	47	38	38	37	33	45	45	44	40	43	48	41	47
8: DR1 (ZEA)	51	51	51	50	52	49	50	100	85	80	82	58	58	59	57	55	65	56	53	53	48	52	55	52	57	43	49	47	49	45	45	45	41	45	48	39	44
9: DR3 (ZCA)	51	50	50	49	51	48	47	85	100	80	81	56	56	57	57	56	63	55	53	51	47	48	56	54	55	42	47	46	48	44	44	45	42	46	47	41	43
10: DR2 (ZDA)	48	50	49	48	49	47	42	80	80	100	75	56	56	57	56	56	64	56	52	49	46	47	51	48	52	40	47	46	49	45	45	46	42	45	49	41	45
11: DR4 (ZBA)	49	49	50	48	50	48	48	82	81	75	100	62	62	61	61	58	66	56	53	53	49	52	55	52	53	42	48	48	47	44	44	45	40	43	45	39	44
12: DR6 (ZGA)	44	45	47	44	45	44	47	58	56	56	62	100	100	97	85	82	74	48	52	47	46	54	51	49	53	39	47	45	42	50	50	45	37	49	50	42	43
13: DR7 (ZHA)	44	45	47	44	45	44	47	58	56	56	62	100	100	97	85	82	74	48	52	47	46	54	51	49	53	39	47	45	42	50	50	45	37	49	50	42	43
14: DR8 (ZIA)	44	45	47	44	47	45	48	59	57	57	61	97	97	100	87	85	74	49	53	48	47	53	52	48	54	40	47	45	42	50	50	44	38	49	51	43	44
15: DR30 (ZKA)	44	45	47	44	49	48	49	57	57	56	61	85	85	87	100	93	70	49	52	49	47	52	52	48	53	40	48	46	45	49	49	43	38	48	51	43	44
16: DR9 (ZJA)	40	41	42	40	44	45	44	55	56	56	58	82	82	85	93	100	67	48	49	47	46	49	49	46	51	38	47	45	45	46	47	43	38	44	46	38	40
17: DR5 (ZFA)	43	44	44	43	45	44	43	65	63	64	66	74	74	74	70	67	100	53	54	49	47	53	52	49	57	43	46	42	44	44	44	43	41	50	48	43	48
18: AM22 (Z2)	49	49	49	48	49	49	47	56	55	56	56	48	48	49	49	48	53	100	82	82	73	73	67	73	62	47	48	48	51	40	40	41	39	45	44	37	46
19: AM25 (Z2)	49	49	50	49	49	45	45	53	53	52	53	52	52	53	52	49	54	82	100	76	70	73	69	70	63	48	48	46	48	45	45	41	41	45	48	38	44
20: AM2 (Z2)	47	45	47	45	47	41	42	53	51	49	53	47	47	48	49	47	49	82	76	100	80	75	67	72	55	47	49	49	45	41	40	38	38	43	43	37	41
21: AM4 (Z2)	47	45	47	45	47	41	42	48	47	46	49	46	46	47	47	46	47	73	70	80	100	71	64	67	52	42	49	48	43	37	37	37	35	41	40	37	39
22: AM18 (Z1)	43	44	44	43	43	39	41	52	48	47	52	54	54	53	52	49	53	73	73	75	71	100	73	65	56	47	47	45	45	40	40	41	35	43	43	34	41
23: AM23 (Z1)	46	49	51	48	46	44	44	55	56	51	55	51	51	52	52	49	52	67	69	67	64	73	100	64	56	44	43	41	41	44	44	41	38	42	42	40	41
24: AM3 (Z2)	49	49	50	49	49	45	47	52	54	48	52	49	49	48	48	46	49	73	70	72	67	65	64	100	61	49	46	45	44	39	39	37	35	36	39	33	34
25: AM8 (Z1)	47	47	48	47	49	47	47	57	55	52	53	53	53	54	53	51	57	62	63	55	52	56	56	61	100	51	45	43	44	45	45	44	43	47	48	43	43
26: AM10 (Z2)	36	38	39	38	36	36	38	43	42	40	42	39	39	40	40	38	43	47	48	47	42	47	44	49	51	100	36	33	32	33	33	33	38	34	33	33	36
27: caauZA1 (Z3)	42	42	45	42	43	38	38	49	47	47	48	47	47	47	48	47	46	48	48	49	49	47	43	46	45	36	100	93	78	47	47	48	42	41	47	39	39
28: cycaZB1 (Z3)	41	40	43	42	43	37	37	47	46	46	48	45	45	45	46	45	42	48	46	49	48	45	41	45	43	33	93	100	77	42	42	45	38	36	42	38	39
29: cycaZC1 (Z3)	37	37	38	37	38	36	33	49	48	49	47	42	42	42	45	45	44	51	48	45	43	45	41	44	44	32	78	77	100	42	42	45	38	38	44	35	36
30: OL4	48	48	51	48	47	46	45	45	44	45	44	50	50	50	49	46	44	40	45	41	37	40	44	39	45	33	47	42	42	100	96	76	61	62	67	62	58
31: OL5	47	47	49	47	46	45	45	45	44	45	44	50	50	50	49	47	44	40	45	40	37	40	44	39	45	33	47	42	42	96	100	76	61	61	66	61	56
32: OL17	45	46	48	46	45	47	44	45	45	46	45	45	45	44	43	43	43	41	41	38	37	41	41	37	44	33	48	45	45	76	76	100	67	52	60	55	55
33: OL7	41	41	41	40	41	42	40	41	42	42	40	37	37	38	38	38	41	39	41	38	35	35	38	35	43	38	42	38	38	61	61	67	100	55	62	57	60
34: ON22	45	45	45	44	45	43	43	45	46	45	43	49	49	49	48	44	50	45	45	43	41	43	42	36	47	34	41	36	38	62	61	52	55	100	79	70	71
35: ON35	47	47	47	46	47	48	48	48	47	49	45	50	50	51	51	46	48	44	48	43	40	43	42	39	48	33	47	42	44	67	66	60	62	79	100	71	72
36: ON23	48	45	46	45	44	41	41	39	41	41	39	42	42	43	43	38	43	37	38	37	37	34	40	33	43	33	39	38	35	62	61	55	57	70	71	100	72
37: ON24	46	45	45	44	46	48	47	44	43	45	44	43	43	44	44	40	48	46	44	41	39	41	41	34	43	36	39	39	36	58	56	55	60	71	72	72	100

Percent amino acid Identity Matrix created by ClustalX 2.0.11. See legend to Text S4a and additional file 4: Text S1 for sequence details.

S4d.1 Alpha 1 domain

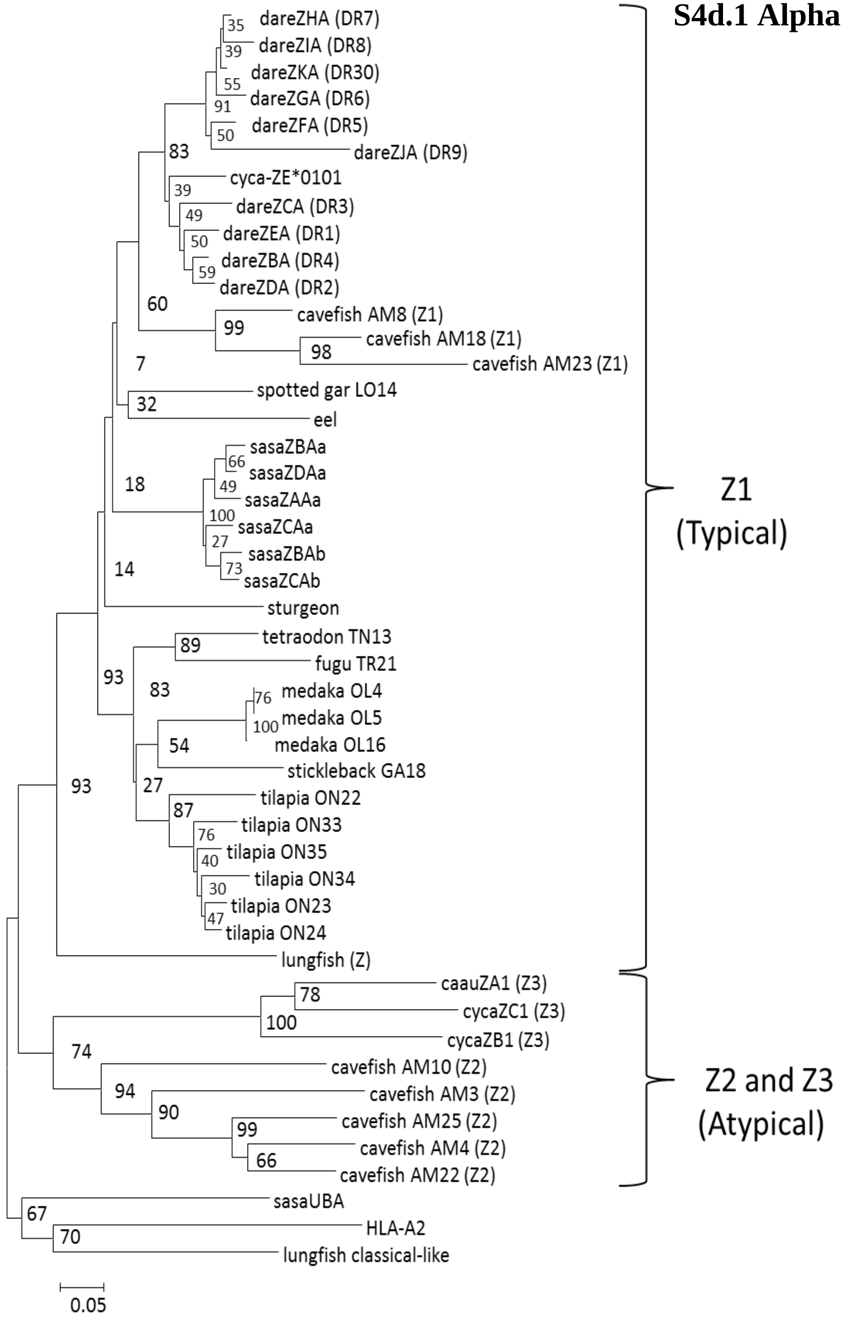

Text S4d.1-3. Phylogenetic tree of deduced Z lineage amino acid alpha 1 (S4d.1), alpha 2 (S4d.2) and alpha 3 (S4d.3) domain sequences

Sequence names reflect species as follows: sasa is *Salmo salar*, dare is *Danio rerio*, cavefish AM is *Astyanax mexicanus*, medaka OL is *Oryzias latipes*, stickleback GA is *Gasterosteus aculeatus*, tilapia ON is *Oreochromis niloticus*, tetraodon TN is *Tetraodon nigroviridis*, fugu TR is *Takefugu rubripes*, cyca is *Cyprinus carpio*, caau is *Carassius auratus*, eel is Japanese Eel (*Anguilla japonica*), sturgeon is (*Acipenser sinensis*) and lungfish is (*Protopterus aethiopicus*). Locus designations for zebrafish sequences comes from Dirscherl & Yoder [main text reference 34] and the Z2 and Z3 sublineages are shown in parenthesis. The trees are rooted using the human HLA-A2, salmon UBA and classical-like lungfish sequences apart from the un-rooted alpa3 domain tree. Sequence references can be found in additional files 3: Text S1 and 4: Text S2 apart from carp ZE*0101 which is GenBank accession # Q8MGT8 and lungfish (Z) which has GenBank accession # AAF15304.1.

As shown in the figures, Z1 lineage alpha 1 and alpha 2 domain sequences cluster with high bootstrap support as opposed to the alpha 3 domain that clusters in a phylogenetic manner.

The evolutionary history was inferred using the Neighbor-Joining method [main text reference 95]. The percentage of replicate trees in which the associated taxa clustered together in the bootstrap test (1000 replicates) are shown next to the branches [main text reference 96]. The tree is drawn to scale, with branch lengths in the same units as those of the evolutionary distances used to infer the phylogenetic tree. The evolutionary distances were computed using the p-distance method [97] and are in the units of the number of amino acid differences per site. All ambiguous positions were removed for each sequence pair. Evolutionary analyses were conducted in MEGA5 [98].

S4d.2 Alpha 2 domain

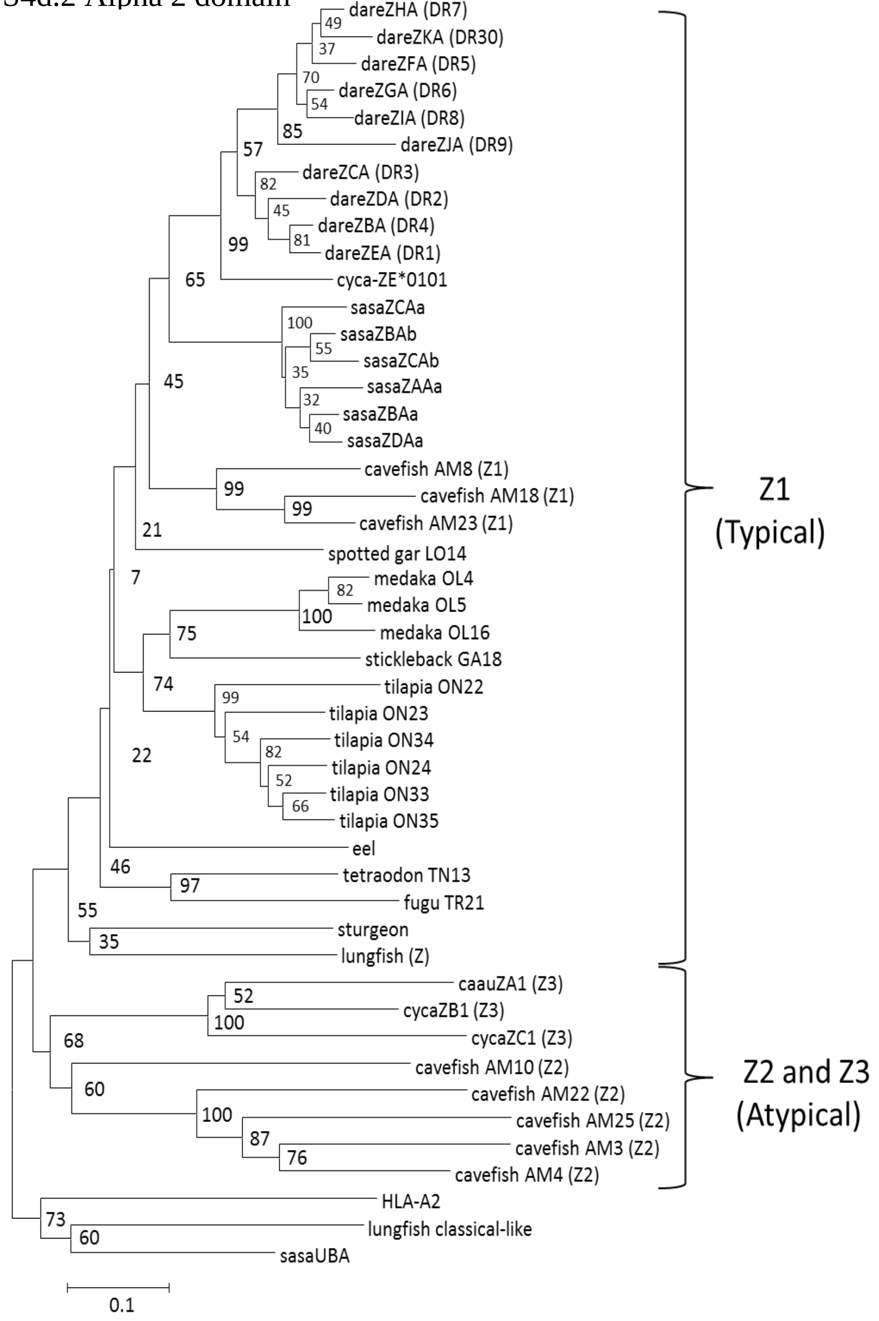

S4d.3 Alpha 3 domain

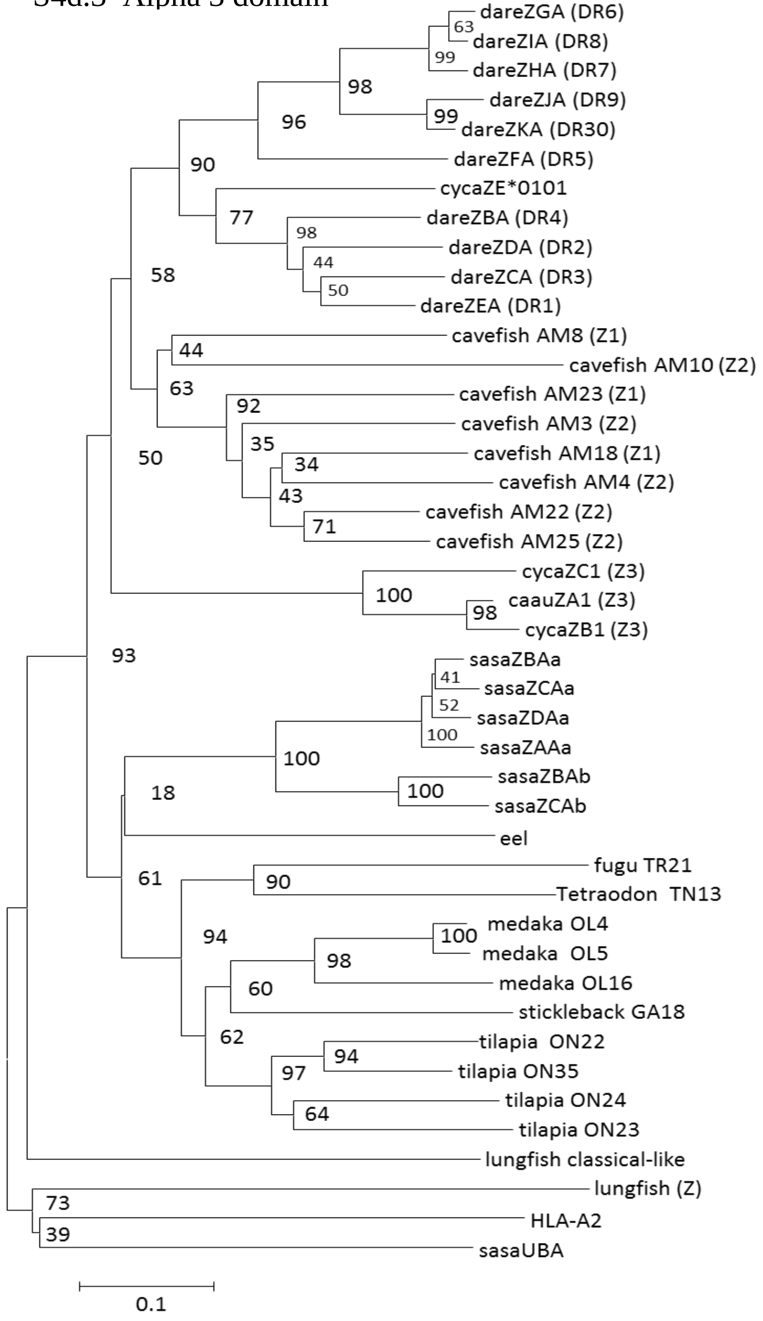

Supplement: Additional file 7: — Text S4. Additional Z lineage data. [file 12862_2015_309_MOESM7_ESM.pdf]
