# Supplementary material for: A comprehensive analysis of teleost MHC class I sequences
Source: BMC Evol Biol. 2015 Mar 6;15:32. doi: 10.1186/s12862-015-0309-1 (PMC4364491; doi:10.1186/s12862-015-0309-1)
Supplement: Additional file 8: — Text S5. Additional L lineage data. [file 12862_2015_309_MOESM8_ESM.pdf]

Additional file 8: Text S5. Additional L lineage data

Table of contents

		Page
Text S5a	Alignment of deduced L lineage amino acid sequences	2
Text S5b	Percent identity per domain between deduced L lineage amino acid sequences	8
Text S5c	Phylogenetic tree of deduced L lineage domain amino acid sequences	9
Text S5d	Exon intron organization of selected teleost L lineage genes	10
Text S5e	Hydrophobicity scores for teleost MHC class I α1α2 domains	11
Text S5f	Sequence and hydrophobicity of selected vertebrate MHC I α1α2 domains	12
Text S5g	Hydrophobicity distribution for selected α1α2 domains	13

Text S5a. Alignment of deduced L lineage amino acid sequences

```
           Leader sequence                      Alpha 1 domain
                                                   1        *          20        *         40
                                                         A B
                                                       A B C             C B         B
HLA-A2   : ----------------------------------------GSHSMRYFFTSVSRPGR-GEPRFIAVGYVDDTQFVRFDSDAA : 41
sasaUBA  : ----------------------MKCFILLLLGIALHSSSAATHSLRYVYTATSGIP--DFPEFVTVGLVNGEPISYYDSI-- : 56
sasaLCA  : -------------------MGKLSVFLFVLSFYTIVNSGSGSHSLWALATYISGET--PFPEFTVVVMLDDVQVAYYDSN-- : 59
sasaLDA  : -------------------MGKLSIFLFVLSFYTIVNAGSGSHSLWALATYIIGET--PFPEFTVVLMLDDVQIGYYDSN-- : 59
sasaLFA  : -------------------MGKLSVLFFIL-FYTIGNAGSGSHSLWALATYINGET--PFPEFTVVVMLDDVQVGYYDSN-- : 58
sasaLGA  : -------------------MGKLSVFLFVFSFYTIVSPGSGSHSLWALATYIVGET--PFPEFTVVVMLDDVQVAYYDSN-- : 59
sasaLHA  : -------------------MGKLSVFLFVLSFYTIANAGSGSHSLWALATHIIGET--PFPEFTVVVMLDDVQVGYYDSN-- : 59
sasaLIA  : -------------------MAKLCFFLILLSLYTIVNA--GSHSLWAFATCISGEA--PFPECSVVLMQDDIQVGYFDSN-- : 57
onmyLAA  : ----------------------MNFYTLFLIYLPIVNAGKSSHSLWGLATHVLGET--EFPEFCVLWMLDDVQVGYYDSN-- : 56
onmyLBA  : -------------------MGKLSIFLFALSFCTIVNSGSGSHSLWALATYISGET--PFPEFTVVVMLDDIQVTYYDSN-- : 59
onmyLCA  : -------------------MGKLSVFLFVLSFCTIVNSGSGSHSLWALATYISGET--PFPEFTVVVMLDDVQVTYYDSN-- : 59
onmyLDA  : -------------------MGKLSIFLFVLSFYTIVNAGSGSHSLWALATYIIGET--PFPEFTVVLMLDDVQIGYYDSN-- : 59
onmyLEA  : ---------------------------------------------------------------------------------- : -
ON9      : -------------------MFVLQLMLMSLLLHHTMSSGTGRHSLWALASYIPGSA--HFPEFTVVLMLDDIQVGYYDSK-- : 59
DR10_LCA : -------------------MMYLVLAVCILSASVFSQS--GSHSLWVFATFLTGDISVQFPEFSAVVMLDDIVIGHYNAD-- : 59
DR11_LBA : -------------------MKTMLFLLYLLSCLTFTDA--GFHSLLVLATYVDGQT--PFPELSVVVMLDDVQIIYYDSD-- : 57
DR12_LAA : ------------------MFLRMFCFVCLLPFIGVVNA--GSHSLMALATYIVGQT--PFPEFSVVVMLDDLQLAYYDSI-- : 58
DR17_LMA : --------------------MRFLCFLCLLSLPTAVVT--DSHSLWFLVTYIEGET--QFPAFSVVYMLDDITVGYYNSE-- : 56
DR18_LOA : ----------------MKWKYTESVVLALSLVFPLASY--GLQSLSLLSTYIKGET--QFPKLSGTAILNDFIVGYYN---- : 58
DR19_LHA : --------------------MGQILLFFVFLLPTAAPK--GSHSLWMLVMYIKGQT--AFPEFSYVMMLDDVSVLYYNGD-- : 56
DR20_LPA : -----------------------------------------SHSIRMFATYIKGKT--PFPELSGVVMLDDIRVLYYNGV-- : 37
DR21_LNA : ---------------------------------------------------------------------------------- : -
DR22_LJA : --------------MSYVGNMIIILFLCVLLAPTTALT--GSHSLWMLATYMKGE---PYSEISITFMLDDITVGHYNSK-- : 61
DR23_LEA : ----------------------------------------GPHSLMLLATYIKGQT--PFPEFSYVLMLDDVSTLYYNGE-- : 38
DR24_LFA : --------------------MDKILLLFLFLLPTSAPK--GSHSLCLLATYIKGPS--PFPELSGVMMLDDIPLLYYNGD-- : 56
DR25_LDA : ----------------------MPVILILSLRLIKT----GSHSLWMHSTYIKGQT--PFPEFSFVLMLDDVRVMYYNGE-- : 52
DR26_LLA : ----------------MTGKVLFVLFL--LSAPTTVLT--DSHSLWLLGTYIKGET--QFPKLSFTVMLDDLRVGFYNSE-- : 58
DR27_LKA : ---------MARTSENMIGKVLFVLFL--LSGPTSVLT--DFHSLQILGTYIKGDT--LFPQISFTFMLDDLTVGLYITE-- : 65
DR28_LGA : -------------------MCKIIFLLLFLLPAAAPK---GSHSLWVVATYIKGQT--PFPEFSYVLMLDDITVMYYNSD-- : 56
DR29_LIA : --------------------MGQILLFFVFLLPTAAPK--GSHSLWMLVMYIKGQT--AFPEFSYVMMLDDVSVLHYNGD-- : 56
AM12_L   : --------------------MFVLYSNICFSLFLCSVS--GSHSLGLIIAYIKGET--PFSEFSFTVLLDDITVGHFDSE-- : 56
AM32_L   : ----------------------------------------GSHSLAMLATYIKGNT--PFPEYSFTMVLDDITVGYYSSE-- : 38
LO11_L   : MEDWRSIHKRSSISHIRDISDLEYFHWRGKSDTPEFSSCEGTHSMSWSFTLTQEAQ--WIPKFAVVGYLDGLPMEYYDST-- : 78
```

```
                                                             Alpha 2 domain
                    *          60        *         80        *         100        *         120
                                                                      A
                                                                      B               C C
            A  A   B  C                       C C               D E
               B              A   B  BB  C  FC  F  FF  F          F E D               E F F
HLA-A2    : SQRMEPRAPWIEQ-EGPEYWDGETRKVKAHSQTHRVDLGTLRGYYNQSEAGSHTVQRMYGCDVGSD-WRFLRGYHQYAYDG- : 120
sasaUBA   : IRRETPRQDWMAKTEGSDYWESQTQVSIGSEQTFKANIDVAKQRFNQTG-GVHVNQKMYGCEWDDETGV-TEGFDQDGYDG- : 135
sasaLCA   : MKHFIYRGHNTPNKIHDDEAKNGDFVFGVMYHHMKERYFHLKHHLNLTE-GVQVQQRMAGCEMFDN-GEPALIMTKNTFNAV : 139
sasaLDA   : IKQSVYRGYHITDKMND-EAQDGTYVLGTMYDHMKERSFRLKHHLNLTE-GVHVQQRIGGCEILHN-GEPALIMTKNSFNAI : 138
sasaLFA   : MKDFIYRGHNPTDKIHDDVAQDGAYVFGIIYQSIKERSFHLKHQLNLTE-GVQVQQRMSGCEMLDN-GEPALIMFKETFNGI : 138
sasaLGA   : DKQSVYRGQHIT-KTKDDEAQDGAHVFRVIYQSMKDRSFELKHRFNLTE-GVQVQQKITGCEMLNN-GESALVMYKDVFNAI : 138
sasaLHA   : MKHFIYSGHNPTDKIHDDVAQDGAYVFGTMYQSIKERSFHLKYHLNLTR-GVQVQQRMAGCEMLNN-GEPALIMSKNTFNAI : 139
sasaLIA   : KEQFIHKGPYAPDETEVEEAQDAAYVFGHMFLSMKRRLSDLRYRFNSTG-NIDVQQRMAGCEMLDT-GEPGLILSTDAFNAI : 137
onmyLAA   : SWRFISR---TDENIDEEYSKTVQGASWDVYLSMRKRSSLLQHRFNSTT-GIFVHQRLIGCELEDN-EKQGQLMIKEAFNGI : 133
onmyLBA   : MKHFIYKGHNTSDKIHDDEAKNGDFVFGVMYHHMKERYFHLKHHFNLTE-GVQVQQKLTGCEMFDN-GEPALVMFKDAFNAI : 139
onmyLCA   : MKHFIYKGHNTSDKIHDDEAKNGDFVFGVMYHHMKERYFHLKHHLNLTE-GVQVQQRMAGCEMFDN-GEPALIMTKNTFNAV : 139
onmyLDA   : IKLSVYRGYHITDKIND-EAQDGSDVLGTMYHHMKERSFRLKHHLNLTE-GVHVQQRIGGCEILHN-GEPALIMTKNSFNGI : 138
onmyLEA   : -------------KMHDNKAQDGAYVFGMMFNNMKERSFNLKHHFNLTE-GVQVQQRMSGCEMLDN-GEPVMFVVKDTFNAI :  67
ON9       : VNQVMRTSTASDHKAELNLGQEPVNVLRDIYSSMRKRLNLVKHRFNLTIDGVHVQQRVTGCEVLED-GQPALIMFRDGSNGQ : 140
DR10_LCA  : DRSFVPSTVQESVNITEQITISTVCKGIHEGMKTKA--YYLIDYLNHTR-GLHVQQRLVGCELLQN--EPGQMMTLEAFNGE : 136
DR11_LBA  : TWRVFHRSPSDSKYYDEDQSDADAVFHDTYDEMKYR-VLHLKNQLNHTD-GITVLQRIVGCELFND--KPGIYHLWDAHDGK : 135
DR12_LAA  : GWKTIYRSGSDSKYYDEEQSDAGIVFRDMFYDMKDR-AFYLKDHQNHTD-GVHVHQRLVGCELLNN-EKTGPLHYWDAFGGQ : 137
DR17_LMA  : TKTCVPRGNTTNEDEEV--DLIGYYLNEYYPPIVE---KFIGLFKNKTK-GPVVHQLLTVCELPDK-DGVGQMITKIAFEGS : 131
DR18_LOA  : -NTYIPRGNTTNEDDVIDADQTKTLADYVYGSFLKRSSLLSQNNQNTSL-D--VYQKLVVCELLDS-DTPGKMFFRDAARGS : 135
DR19_LHA  : TNSLIARGNTKADDDVFNLNALPIIHDHLQSSFEDR-WTLATTHLNKTD-GVFALQQLVACELQDD-GEPGRMILRNAFGGS : 135
DR20_LPA  : TDSFLARGNTTAEDDVFDPDDLENIKGFIKSEFS---FLNAQNTFTKTD-GIFVSQILAMCELKDD-GEPGQMIGQDAFERL : 114
DR21_LNA  : ----------------------------------------------------------------------GQMITKSAFRGL :  12
DR22_LJA  : TERYIARHNTTNEDELTW-NTFYVARHDLHTFFVR---KLK--FKNHTE-SPQLYQMMSHCELQGN-NKPGQMVSKIAFSGS : 135
DR23_LEA  : TKTYVPRGNTTAEDDVFETNVLLKISNNIQLSFMEK-WVVGTNNINKTH-GVLVLQRLVVCDLKDD-GEPGKMITRDAFRGS : 117
DR24_LFA  : TKTFFMRGNTTAEDNVFDANAFLSIIGHIQSSFVDR-WGLASRDLNKTD-RIFTLQQLVLCELSED-GEHGKMISRDAVEGT : 135
DR25_LDA  : TKNVFPRGNATNEDDVFDLNVLRTISDKVHSSFEEK-WVVATQDVNKTE-VVLALQRIVGCELRHD-GKPGQMITRDAARGS : 131
DR26_LLA  : TKEFIPRDNTTNEDEAVS-DVLTVIQNTLEPFMMS---TLT--FKNGTE-SPQLFQITWHCELLDN-DKPGQIIFKIAFSGS : 132
DR27_LKA  : RDEFIPRDNTTNEDETVS-DMLDKIKNELQPLLKG---TLI--FQNSTE-SPQLFQIAWHCELLDN-NKTGQMILKIAFSGS : 139
DR28_LGA  : TKTFFPRGNTTAEDDLYGSKDHLIIHDFMRSTFKDK-WGLATKKLNKTD-GVFALQQLVVLDLRAD-SEPGQIISQNTFEGC : 135
DR29_LIA  : TNSLIARGNTKADDDVFNLNALPIIHDHLQSSFEDR-WTLATTHLNKTD-GVFALQQLVACELQDD-GEPGRMILRNAFGGS : 135
AM12_L    : KKMYFSRGLSDVEEDDGV---------------------------------------------------------------- :  74
AM32_L    : TKTFVS---------------------------------------------------------------------------- :  44
LO11_L    : HERVVSRRHWRPDPAPAENEEEKKGAAEHTYISMFEKLQLAKKHFNHSG-GLHTYQRQAGCELSDD-GT-QLFHARDAYDG- : 156
```

```
                                                                                   Alpha 3 domain
                       *         140         *         160         *         180         *
                                                E          DD  A
                 FF                     F   FF     E       EE  DD   A   A   A
HLA-A2    : --KDYIALKEDLRSWTAADMAAQTTKHKWEAA-HVAE--QLRAYLEGTCVEWLRRYLENGKETLQRTDAPKTHMTHHAVS-- : 195
sasaUBA   : --EDFLAFDLKTLTWIAPTPQAVITKLKWDSNTAQNE--YRKNYLTQTCIEWLKKYLDYGKSTLMRTVPPSVSLLQKTPS-- : 211
sasaLCA   : FADHAIYYNITHFTYDAGKLLQG-----WDGMRQAQEKILYENVLLTLCIRTLKTILKREKNIVMRKVPPRLR-LIKKEVSG : 215
sasaLDA   : FEDYAVYYNMTHFTYDSGKLLLG-----YNWIRQATERTLYANVWLPICINTLKKCLKRE-NFVMRRVPPRLR-LIKKAVSG : 213
sasaLFA   : FVDHAIYNSMTHFTYDSGTLLLG-----YYGIRQAYEKALFENVLLPICIKNLKTILKREKNVVMRKVPPRLR-LIKKEVSG : 214
sasaLGA   : YTDRTLYYNMTHFTYDAGKLLLG-----WDGIRQAYERTLYENVYLPICIKSLKRLLKREKNIVMRKVPPRLR-LIKKEVSG : 214
sasaLHA   : YTDRTLYYNMTHFTYDAGKLLPG-----WDAMRREYLRILFGNVFLPICIKTMKTFLKMEKNVVMRKVPPRLR-LIKKEVSG : 215
sasaLIA   : LAD-LIYYNMTHYSYNSGNLLSP-----WSEVHQTYTKWHYQTIYLPVCIKTLKRFLERLKNFVMRKVRPRVR-LIQKAMSG : 212
onmyLAA   : DGGVLNFNKLQYNYHPKWPELEF------NQQRTQYIQMGLDKVYLPICIKSLKDYLKKEEKLVMRKVRPRVR-LISKESTD : 208
onmyLBA   : YTDRTLYYNMTHFTYDAGKLLLG-----WDGMRQAYERTLYENVYLPICIKSLKKILKREKNVVMRKVPPRLR-LIKKEVSG : 215
onmyLCA   : FVDHAIYYNMTHFTYDAGKLLQG-----WDGMRQLQEKILYENVLLTLCIRTLKTILKREKNVMMRKVPPRLR-LIKKEVSG : 215
onmyLDA   : FADYAVYYNMTHFTYDSGQLLLG-----YNWMRQATERTLYANVWLPICINTLKKCLNREKNFVMRRVPPRLRLLIKKEVSG : 215
onmyLEA   : YTDQMVCYNMTHFIYDAGKLLRG-----WDGMRQAFERVLFENVHLPICIRTLKTILKREKNFAMRKVPPRLR-LIKKEVSG : 143
ON9       : DADSLLYNMTHFTYAVREGWEIQ-----WDALKKTSFQMLYSNIYLPFCVRTLQHFLEREKHLVMRRVKPRLR-FITRQVVG : 216
DR10_LCA  : SGFERR-YDIQGDQQTHWKWPVIK-----SRAQLEYDAWLYAHFYRPLCISQLRKYLKKEKKRVMARVKPRVR-VIQRTCSK : 211
DR11_LBA  : TIEKFTFNIYNHEFQLKNQWFRT-----WDQVMIQQKRIVHENIYYPVCIKVLRRYLNVEKNSVMRKVKPRVR-LMKKKLPD : 211
DR12_LAA  : N-MEEFIFDTEKHTIQVKMPWVIT----WDQLKRLHENFMYDNVYHPICIKTLRRYLNMEKKNVMRKVKPRVR-LMQKKLSD : 213
DR17_LMA  : TTDEMHFIDDT-LTFQGMEKVT--------KLHLEMFRW-FHAMAYKRGKLILEKYLKKRATQGKRRVKPRVR-LIQKAS-D : 201
DR18_LOA  : TIDEILYTNNT-FMYTVIGNVSQE----LIKPHLEAFKLDFSTLFYPVCIKTLKTYLKKRKSQVNRREMPNSR-LFRKAS-D : 210
DR19_LHA  : TTDQLLFVDKK-FTYHDSFNVSTH----VLHAHHDYNKYLCEKLLQPFCFQTLKGYLVKRRNQINRKVKPEVR-LIQKANSD : 211
DR20_LPA  : TIFTVLYADNK-CTIDINLNISQE----QKEKIIEGVKNYYRNLIQPFCYKTLKVYLKKRKDQVNRKVEPKVR-IFHKANLD : 190
DR21_LNA  : TVDELHFLDGK-FTYQGSLNYTKL----EIKPYLDLAMWRFETLYYPACIETLKSYLKKRGTQVNRKVKPRVR-LIQKAS-D :  87
DR22_LJA  : TTDEMSIYENK-FTYQSQKEMPS---------LALELIKWRHESVSYPTCISTLRNYLKMRQTQVNRKVKPKVR-LFQKANSD : 207
DR23_LEA  : TTDELQYFDKT-FTYQGTLNVSDN----LLKIHLEASKRNHEYLYQPYCIKTLKGYLKKRTNQVNRKVKPQVR-LIQKAS-D : 192
DR24_LFA  : TTDELQHVDHK-FTYKHTLNVSAY----LIDFYLELTKSLHKTLFQPTCFKTLSGYLIQRRNQINRKVKPKVR-LFKKELSS : 211
DR25_LDA  : TTDELLYVDKN-FTYQGTLNVPAF----VLNMHLKISMWNHEHLYHPFCIKTLKGYLEKRKNQVNRKVKPKVR-LLLKKLSS : 207
DR26_LLA  : TTDEVSFYNDN-FTCQCQNNMATV--------PLESFQMHYETVCYPNCIATIRDYLKKRQTQYNRK--------------- : 190
DR27_LKA  : TTDEVSFYGNN-FTCQCQNNMATV--------PLEYFQMQFKSIYYPVCMTTLGGYLVKRQVQVNRKVQPKVR-LIKKVHPK : 211
DR28_LGA  : ITDEVRYVDKK-LTYQGTLNVSAP----VHHIHHEYVKYLCETLIHPFYFKTLKGYLIKRRNQINRKVKPKVR-LILKANSD : 211
DR29_LIA  : TTDQLLFVDKK-FTYHDSFNVSTH----VLHAHHDYNKYLCEKLLQPFCFQTLKGYLVKSRNQINRKVKPKVR-LIQKANSD : 211
AM12_L    : -------VDPN-QARSMSSLMHSD------LD-ERWLYVKHVLNQTEQCIKTLRGFLKKRSNQVKRKVKPRVR-IIQRRRSV : 140
AM32_L    : ------------------------------------------------------------------AVKPRVR-LIQRRCSV :  59
LO11_L    : --QDLLQYNMDHLRWDPLVPELKN-----DEALMHSDDIEQNNTYQPLCIQVLKSYLEQEKDRPVMRVKPRVQVFQKTSALS : 231
```

```
                 200       *         220        *         240       *              260       *
HLA-A2     : DHEATLRCWALSFYPAEITLTWQRDG-EDQTQDTELVETRPAGDGTFQKWAAVVVP--SGQEQ---RYTCHVQHEGLPKPLT : 271
sasaUBA    : ---SPVTCHATGFYPSGVMVSWQKDG-QDHHEDVEHGETLQNDDGTFQKSSHLTVT---PEEWKNNKYQCVVQVTGLQEDFI : 286
sasaLCA    : --GFQVSCLVFGFYPRHINLTLLRDGQPVAEQELTGGEVLPSGDGTYQLRKSLEVS--TEELKKRHNYTCTASHLSLDNKLD : 293
sasaLDA    : --DIQVICLAFGFYPRHINLTLLRDGHPVAEQELTGGEVLPSGDGTYQLRKSIYVS--TEELRERHNYTCTASHLSLNNKLD : 291
sasaLFA    : --GLQVSCLAFGFYPRHINLTLLRDGQPVAEQELTGGEVLPSGDGTYQLRKSLEVS--TEELKKRHNYTCTASHLSLDNKLD : 292
sasaLGA    : --GFQVSCLAFGFYPRHINLTLLRDGQPVAEQELTGGEVLPSGDGTYQLRKSLEVS--TEELKKRHNYTCTASHLSLDNKLD : 292
sasaLHA    : --GLQVSCLAFGFYPRHINLTLLRDGQPVAEQELTGGEVLPSGDGTYQLRKSLEVS--TEELKKRHSYTCTASHLSLDNKLD : 293
sasaLIA    : --GACVSCLAFGFYPRHINLTLLRDGQPIVEQEMTGGQLLPNGDGTYQMRKSLEVN--TEELRERHNYTCTTSHLSLDNKLD : 290
onmyLAA    : TEGAKITCLAFGFYPRHINLTLLRDGQPVAEHELKGGQLLPNGDWTYQLRKSLTIT--VQELRERPNYTCTANHISMDNKLD : 288
onmyLBA    : --GFQVSCLAFGFYPRHINLTLLRDGQPVAEQELTGGEVLPSGDGTYQLRKSLEVS--TEELKKRHNYTCTASHLSLDNKLD : 293
onmyLCA    : --GFQVSCLAFGFYPRHINLTLLRDGQPVAEQELTGGEVLPSGDRTYQLRKRLEVS--TEELKKRHNYTCTASHLSLDNKLD : 293
onmyLDA    : --DLQVICLAFGFYPRHINLTLLRDGHPVAEQELTGGEVLPSGDGTYQLRKSIYVS--TEELREKHNYTCTASHLSLNNKLD : 293
onmyLEA    : --GLKVSCLAFGFYPRHINLTLLRDGQPVAEQDLTGGEVLPSGDGTYQLRKSLEVS--TEELKKRHNYTCTASHLSLDNKLD : 221
ON9        : --GAQVTCLATDFYPRHINLSLLRDGQPVDEGEVRVGSVLPNGNGLYQVRKTLMVG--EKELQRKHNYTCEAFHLSLDNRLR : 294
DR10_LCA   : TGKIQMTCLATGFYPRHINLTLLQDGQPVNEERVMGGELLPNADGTYQMRKSVELS--AEEQRERRTYTCTVNHLSLDNKLD : 291
DR11_LBA   : SQGLQISCLATGFYPRHINLTLFRDAEPVDDDQIIGGEILPNGDGMYQMRKSLIVS--KEELDEGHEYTCTMKHLNLDNKLD : 291
DR12_LAA   : SQGLQISCLATGFYPRHINLTLFRDAELVDDDQITGGEILPNGDGTYQMRKSLIVS--EEELQKGHKYNCTANYLNLDNKMD : 293
DR17_LMA   : S-GFCVSCLATGFYPRHINLTLLRDGQPVSDHEVTGGDLLPNGDGTYQMRKSLEIS--AEE-RQKHKYSCSAKHLSLDNKLI : 279
DR18_LOA   : SGGFRVSCLATGFYPRHINLTLLRDGQSVSDHEVTGGDLLPNGDGTYQMRKSLEIK--AEE-REKHKYSCSVKHMG--EEFH : 287
DR19_LHA   : SGGFRVSCLATGFYPRHINLTLLRDGQPVSDHEVTGGDLLPNGDGTYQMRKSLEIS--AEE-REKHKYSCSAKHLKIDNKLD : 290
DR20_LPA   : FGGFRVSCLATGFYPRHINLTLLRDGQPVSDHEFTGGDLLPNGDGTYQMRKSLEIR--AED-SEKHKYTCSFKHLD--KEWH : 267
DR21_LNA   : SGWLAVSCLATGFYPRHINLTLLRDGQPVSDHEVTGGDLLPNGDGTYQMRKSLEIR--AEE-RQKHKYTCSAKHLGL-DNKL : 165
DR22_LJA   : SGGFRVNCLATGFYPRHINLTLLRDGQPVSENEVTGGDLLPNGDGTYQMRKSLEIR--AEE-RQKHKYTCSAKHLSLDNKLI : 286
DR23_LEA   : LGWFVVSCLATGFYPRHINLTLLRDGQPVSDHELTGGDLLPNGDGTYQMRKSLEIR--AEE-REKHKYSCSVKHLSLDNKLD : 271
DR24_LFA   : --GFIVSCLATGFYPRHINLTLLRDGQPVSDHDVTGGDLLPNGDGTYQMRKSLEIR--AEE-RQKHKYSCSVKHLSLDNKLH : 288
DR25_LDA   : --SFRVSCLATGFYPRHINLTLLRDGQPVSDHDVTGGDLLPNGDGTYQMRKSLQIR--AEE-REKHKYTCSAKHLSLDNKLD : 284
DR26_LLA   : ------------------------DGQPVSDHEVTGGNLLPNGDGTYQMRKSLEIR--ADE-REKHKYTCSVSHLSLDKILD : 245
DR27_LKA   : SGGFRLSCLASEFYPYFINLTLLRDGQPVSDHEVTGGDLLPNGDGTYQMRKSLEIR--ADE-REKHKYTCSAKHL--DKNLD : 288
DR28_LGA   : SGGFRVSCLATGFYPRHINLTLLRDGQPVSDHGVTGGDLLPNGDGTYQMRKSLEIR--GEE-REKHKYTCSATHLSLDKKLD : 290
DR29_LIA   : SGGFRVSCLATGFYPRHINLTLLIDGQPVSDHEVTGGDLLPNGDGTYQMRKSLEIR--AEE-REKHKYSCSAKHLKIDNKLD : 290
AM12_L     : SGWDGVTCLATGFYPRHINLTILRDGQPVPDHLITGVSCCPMETGRIR---------------------------------- : 188
AM32_L     : SGWDGVTCLATGFYPRHINLTVHRNGLPVPDHLITRGG-------------------------------------------- :  97
LO11_L     : -GGTEVTCLATGFYPRALELTLLRDRRPVPEQELTGGEVLPNGDGTYQLRKSLALSKEEEERRERHRYTCRVQHSGLDNMLE : 312
```

```
                             280           *           300         *         320         *         340
                    CP              TM                     CYT
HLA-A2   : LRWEPSSQPTIP-----IVGIIAGLVLFGAVITGAVVAAVMWRRKSSDRKGGSYSQAASSDSAQGSDVSLTACKV------ : 341
sasaUBA  : KVLTESEIKTNWNDPN-IVLIIGVVVALLLVVVAVVVGVVIWKKKSKKGFVPASTSDTDSDNSGRAAQMT----------- : 355
sasaLCA  : VSWESGAERVHLST------LSVLLVMLLILILLVTFICV--KRRWSNTASQ--SELANV-DAKVSEEMNLSSDSEN---- : 359
sasaLDA  : VSWESGAERVHLFI------LSAPLVMALIVILFCIFICLV-RRIRAASQNL--LQLASV-DALEADEMNLSSDSEKT--- : 359
sasaLFA  : VSWESEAERVHLST------LSVLLVML-ILILLGIFICV--KRRWRCTASH--LKLVNV-DAKA---------------- : 345
sasaLGA  : VSWESGAERVHLST------LSVLLMMLLILILLGIFICV--KRRWSNTASQ--SELANV-DAKVSEEINLSSDSET---- : 358
sasaLHA  : VSWEPGAERVHLFT------ISILLMMLLIVILLGIFICV--KR-RRCTASQ--AFVTSC-QHGGH--------------- : 347
sasaLIA  : VSWIPESGMDR----VGLYVKSAPLATVAIIILLSIFVCV--RRRNTAGSQTL-SQLSNANDAQVAEQISLSSHSET---- : 360
onmyLAA  : VSWVPDTGPDS-----ASIIPVVLVMAVVLILIGILVVIGMWKWKHAGVPTFSGHIYSAAKDTET-EQSN-SSLETESTDS : 362
onmyLBA  : VSWESGAERVHLST------LSALLVMLLVVILFSIFICV--KRRWSNTASQ--SKLANV-DATVSEEINLSSDSET---- : 359
onmyLCA  : VSWESGAERVHLST------LSALLVMLLVVILFSIFICV--KRRWSNTASQ--SKLVNV-DATVSEEMNLSSDSEN---- : 359
onmyLDA  : VSWESGAERVHLFI------LSAPLVMALIVILFCILICLA-RRIRAASQN---LQLASV-DALEADERNLSSDSEI---- : 359
onmyLEA  : VSWESGAERVHLST------FSALLVMLLIVILLGIFICV--RR-WRYTASQ--SKIANV-DAKVSEEMNLSSDSET---- : 286
ON9      : INWRAESSYSHRVHS--------ISPLVVLMLAAVLLLVLVLRRRRRRRK------------------------------ : 336
DR10_LCA : ISIEPGLDP----------VIIFPSVLLLLCVFGVLAGFLMWRKKYKQPEQVTYTPTSAT-DQTTEQSQL----------- : 350
DR11_LBA : IVFDVSGTVPGC-----FSVSVVISVLVFMCVSVFIITKLIMRRKRQDTG--RGSEKCDY-SPTISSSQDEI--------- : 355
DR12_LAA : IVFDVAESDP-----GSFSVSVVMGVLVFVGLSVLSITALIMRRKRRDTGSVSGTSQNQYVYA-QTSVQDAT--------- : 359
DR17_LMA : ATLEFDHGEPYKS-------VIPSVLAVLALMLVFGAAAAVWKRRRTGTMRFKINL------------------------- : 328
DR18_LOA : IDLADPHRIHTW---------IVVAVPLVCATALVGLVVFIWKSGQKDRTAGQRERGEHDGRVL---------------- : 342
DR19_LHA : IYLGIYFLSDFDPEEPFP---LVLILLSLVSVFITGVII------------------------------------------ : 326
DR20_LPA : IDLAEPHRNTIW---------IAVSVLLVCA-IIVGLAMLIWKRYQTARQR-ENEPNNHQTTHMN---------------- : 321
DR21_LNA : INLNQANHINQ---------------------------------------------------------------------- : 176
DR22_LJA : VTF------DFDPGEPFKSVIPSVLIILSLVLVFITGVVIY-KCRKRRAVSSKRDYISASTSEESTGSTVTQPQQEAT--- : 357
DR23_LEA : VDFVV-LVADFDHGEPFKSLIPSVLVVSTLMLVFGVAAAVIARKRRCSGTT------------------------------ : 321
DR24_LFA : VDL------DFDHSKPFQSVIPSVLTVLALLLVFGVAAVI-WKRKCRDSVKCGYSAASTSVENMETT-------------- : 348
DR25_LDA : VNLGERF--EPVHSK----VIVACVVG-LVLLTIAGTII-ECRKRKQSGNKCN---------------------------- : 329
DR26_LLA : I--------DFEFDPSFLIKIVIPVVVLLSLMLVLTAVLI--HKCKNKRAESKETITTASTPEPTEMSETLRK-------- : 308
DR27_LKA : V--------DFEFDPSYPVKIVIPVVVLLSLVLVLTAVLI--HKCMNKQAAS-----PPEQQEPIEMGGRLRK-------- : 346
DR28_LGA : ITLGLVAEFDPGELFKS--VIPAVLIVLSLVLVFITGVVI----------------------------------------- : 328
DR29_LIA : IYL------DFDPEEPFP----LVLILLSLVSVFITGVIIY-KCRKRRAVIIYLLSKCIKKITS----------------- : 343
AM12_L   : --------------------------------------------------------------------------------- :   -
AM32_L   : --------------------------------------------------------------------------------- :   -
LO11_L   : VAREPEPDLDTG------LIAGVVIGVLIVALVLPVAACVLWRKKGRGACRRSDVKYTEAQGRDQSGPSSNSSGP------ : 381
```

Alignment of deduced L lineage amino acid sequences compared with salmon *UBA*0301* and Human HLA-A2 sequences. Dashes indicate missing sequence. Individual domains are shown on top. Numbering above the alignment is based on mature HLA-A2 while number of amino acids for each sequence is shown on the right hand side. Human HLA-A2 residues known to anchor peptides i.e. Y7, Y59, Y84, T143, K146, W147, Y159, Y171 are shaded red. HLA-A2 residue positions known to contribute to the six pockets A through F [main text reference 1 and 3] are indicated above the alignment. Residues are colored according to physiochemical properties and conserved or semi-conserved N-linked glycosylation sites are underlined. Species names generally reflect species Latin name where sasa is *Salmo Salar* (Atlantic salmon), onmy is *Oncorhynchus mykiss* (rainbow trout), DR is *Danio rerio* (zebrafish), ON is *Oreochromis niloticus* (nile tilapia), AM is *Astyanax mexicanus* (blind cavefish) and LO is *Lepisosteus oculatus* (spotted gar). Rainbow trout (onmy) L lineage sequences are from Dijkstra et al., 2007 [main text reference 26] and zebrafish sequences from Dirscherl et al, 2014 [main text reference 25]. GenBank sequence references not shown in additional files 3: Text S1 and 4: Text S2 are sasa*UBA* allele**0301* AAN75116.1 and human HLA-A2 AAA76608.2. Abbreviations are as follows: CP for connecting peptide, TM for transmembrane region and CYT for cytoplasmic domain.

Text S5b. Percent identity per domain between deduced L lineage amino acid sequences

Alpha 1 domain

	1	2	3	4	5	6	7	8	9	10	11	12	13	14	15	16	17	18	19	20	21	22	23	24	25	26	27	28
1: DR11_LBA	100	64	49	46	48	46	49	48	46	48	38	43	39	35	33	32	33	33	39	36	36	26	28	25	28	30	25	20
2: DR12_LAA	64	100	52	52	54	54	56	52	52	58	45	41	41	37	36	35	34	31	40	34	31	25	30	26	27	32	25	19
3: sasaLFA	49	52	100	90	79	77	79	74	74	76	52	51	47	33	36	35	38	39	41	40	36	35	36	30	33	43	26	20
4: sasaLHA	46	52	90	100	79	77	78	74	74	71	55	49	43	35	35	34	34	35	40	36	33	32	35	28	30	41	24	18
5: onmyLCA	48	54	79	79	100	98	95	72	74	69	51	46	43	33	36	35	35	35	41	36	33	30	35	29	30	38	24	19
6: onmyLBA	46	54	77	77	98	100	93	69	72	69	53	46	45	33	34	33	34	34	41	36	35	30	35	29	32	40	26	19
7: sasaLCA	49	56	79	78	95	93	100	72	74	72	50	46	43	33	38	36	36	38	42	38	34	32	38	31	33	41	26	20
8: sasaLDA	48	52	74	74	72	69	72	100	94	76	52	48	48	36	33	32	38	37	40	37	30	30	34	25	28	43	28	21
9: onmyLDA	46	52	74	74	74	72	74	94	100	75	49	49	47	37	33	32	38	37	40	37	30	30	33	25	28	41	27	20
10: sasaLGA	48	58	76	71	69	69	72	76	75	100	53	50	52	35	35	33	38	37	39	37	34	31	33	25	28	39	28	21
11: sasaLIA	38	45	52	55	51	53	50	52	49	53	100	48	43	32	28	27	29	31	35	34	33	27	30	25	32	32	23	24
12: onmyLAA	43	41	51	49	46	46	46	48	49	50	48	100	45	30	33	32	30	32	33	30	29	36	34	27	32	35	27	23
13: ON9	39	41	47	43	43	45	43	48	47	52	43	45	100	27	32	31	28	29	33	31	28	26	26	23	29	32	22	15
14: DR10_LCA	35	37	33	35	33	33	33	36	37	35	32	30	27	100	27	29	30	30	36	31	29	21	28	23	29	33	23	20
15: DR19_LHA	33	36	36	35	36	34	38	33	33	35	28	33	32	27	100	99	58	60	60	61	53	40	39	34	37	38	19	13
16: DR29_LIA	32	35	35	34	35	33	36	32	32	33	27	32	31	29	99	100	56	59	59	60	52	38	39	34	38	37	18	13
17: DR23_LEA	33	34	38	34	35	34	36	38	38	38	29	30	28	30	58	56	100	66	58	59	52	42	45	40	38	48	22	13
18: DR25_LDA	33	31	39	35	35	34	38	37	37	37	31	32	29	30	60	59	66	100	60	54	53	44	45	38	39	46	25	15
19: DR28_LGA	39	40	41	40	41	41	42	40	40	39	35	33	33	36	60	59	58	60	100	61	54	38	43	36	43	49	26	19
20: DR24_LFA	36	34	40	36	36	36	38	37	37	37	34	30	31	31	61	60	59	54	61	100	59	44	39	33	39	41	20	15
21: DR20_LPA	36	31	36	33	33	35	34	30	30	34	33	29	28	29	53	52	52	53	54	59	100	39	44	38	39	42	19	15
22: DR18_LOA	26	25	35	32	30	30	32	30	30	31	27	36	26	21	40	38	42	44	38	44	39	100	45	39	36	44	17	10
23: DR26_LLA	28	30	36	35	35	35	38	34	33	33	30	34	26	28	39	39	45	45	43	39	44	45	100	65	52	57	18	16
24: DR27_LKA	25	26	30	28	29	29	31	25	25	25	25	27	23	23	34	34	40	38	36	33	38	39	65	100	44	49	16	13
25: DR22_LJA	28	27	33	30	30	32	33	28	28	28	32	32	29	29	37	38	38	39	43	39	39	36	52	44	100	47	14	13
26: DR17_LMA	30	32	43	41	38	40	41	43	41	39	32	35	32	33	38	37	48	46	49	41	42	44	57	49	47	100	17	18
27: sasaUBA0301	25	25	26	24	24	26	26	28	27	28	23	27	22	23	19	18	22	25	26	20	19	17	18	16	14	17	100	31
28: HLA-A2	20	19	20	18	19	19	20	21	20	21	24	23	15	20	13	13	13	15	19	15	15	10	16	13	13	18	31	100

Alpha 2 domain

	1	2	3	4	5	6	7	8	9	10	11	12	13	14	15	16	17	18	19	20	21	22	23	24	25	26	27	28	29
1: DR10_LCA	100	43	29	40	28	28	31	29	23	23	23	22	32	32	33	29	31	34	32	28	32	30	31	36	36	29	32	20	19
2: onmyLAA	43	100	33	40	31	31	28	25	27	27	21	22	29	27	25	29	40	43	36	33	32	31	38	36	34	32	29	21	15
3: DR11_LBA	29	33	100	50	30	31	27	23	25	25	20	24	32	36	28	27	33	33	30	30	31	29	26	24	27	34	28	21	19
4: DR12_LAA	40	40	50	100	29	29	31	24	26	26	24	24	31	32	27	29	38	36	35	32	30	30	32	31	34	29	37	24	20
5: DR26_LLA	28	31	30	29	100	74	58	41	36	35	33	31	39	39	42	34	25	28	27	27	26	26	28	25	25	26	24	12	13
6: DR27_LKA	28	31	31	29	74	100	57	40	40	39	31	35	40	42	40	35	24	25	25	24	20	19	25	21	22	26	22	13	13
7: DR22_LJA	31	28	27	31	58	57	100	39	36	35	31	38	42	43	39	33	27	30	32	31	30	28	32	26	25	27	19	13	16
8: DR17_LMA	29	25	23	24	41	40	39	100	33	32	33	30	42	35	39	31	18	23	18	18	17	17	18	20	20	16	18	18	15
9: DR19_LHA	23	27	25	26	36	40	36	33	100	99	62	55	58	56	40	44	27	32	28	27	29	29	30	25	26	26	26	17	20
10: DR29_LIA	23	27	25	26	35	39	35	32	99	100	61	54	57	55	39	43	27	32	28	27	29	29	30	25	26	26	26	17	20
11: DR28_LGA	23	21	20	24	33	31	31	33	62	61	100	54	56	54	34	44	24	25	28	24	25	25	27	24	24	26	19	12	13
12: DR24_LFA	22	22	24	24	31	35	38	30	55	54	54	100	57	51	43	42	23	25	25	21	20	20	22	19	20	26	23	15	16
13: DR23_LEA	32	29	32	31	39	40	42	42	58	57	56	57	100	70	56	48	34	37	33	32	33	32	34	31	29	33	28	20	16
14: DR25_LDA	32	27	36	32	39	42	43	35	56	55	54	51	70	100	46	41	32	34	30	29	29	28	28	29	32	33	32	17	17
15: DR18_LOA	33	25	28	27	42	40	39	39	40	39	34	43	56	46	100	43	24	28	27	26	26	25	29	23	24	33	29	24	11
16: DR20_LPA	29	29	27	29	34	35	33	31	44	43	44	42	48	41	43	100	28	33	29	27	30	29	27	26	26	32	25	19	15
17: sasaLGA	31	40	33	38	25	24	27	18	27	27	24	23	34	32	24	28	100	89	74	70	69	67	69	70	66	52	47	20	14
18: onmyLBA	34	43	33	36	28	25	30	23	32	32	25	25	37	34	28	33	89	100	75	73	73	73	72	68	67	53	48	22	14
19: sasaLHA	32	36	30	35	27	25	32	18	28	28	28	25	33	30	27	29	74	75	100	70	71	72	68	64	62	53	47	17	14
20: onmyLEA	28	33	30	32	27	24	31	18	27	27	24	21	32	29	26	27	70	73	70	100	71	71	68	63	61	50	41	22	13
21: sasaLCA	32	32	31	30	26	20	30	17	29	29	25	20	33	29	26	30	69	73	71	71	100	95	74	67	67	53	46	22	16
22: onmyLCA	30	31	29	30	26	19	28	17	29	29	25	20	32	28	25	29	67	73	72	71	95	100	75	66	65	52	45	22	16
23: sasaLFA	31	38	26	32	28	25	32	18	30	30	27	22	34	28	29	27	69	72	68	68	74	75	100	68	68	52	42	23	18
24: sasaLDA	36	36	24	31	25	21	26	20	25	25	24	19	31	29	23	26	70	68	64	63	67	66	68	100	95	49	46	21	16
25: onmyLDA	36	34	27	34	25	22	25	20	26	26	24	20	29	32	24	26	66	67	62	61	67	65	68	95	100	50	49	24	18
26: sasaLIA	29	32	34	29	26	26	27	16	26	26	26	26	33	33	33	32	52	53	53	50	53	52	52	49	50	100	43	18	14
27: ON9	32	29	28	37	24	22	19	18	26	26	19	23	28	32	29	25	47	48	47	41	46	45	42	46	49	43	100	22	23
28: sasaUBA0301	20	21	21	24	12	13	13	18	17	17	12	15	20	17	24	19	20	22	17	22	22	22	23	21	24	18	22	100	40
29: HLA-A2	19	15	19	20	13	13	16	15	20	20	13	16	16	17	11	15	14	14	14	13	16	16	18	16	18	14	23	40	100

Alpha 3 domain

	1	2	3	4	5	6	7	8	9	10	11	12	13	14	15	16	17	18	19	20	21	22	23	24	25	26	27	28
1: sasaLFA	100	99	98	98	99	99	97	89	91	81	72	63	57	58	59	67	65	67	68	68	68	67	66	64	64	63	34	31
2: sasaLHA	99	100	97	97	98	98	96	88	89	80	71	63	57	58	59	67	65	67	68	68	68	67	66	64	64	62	34	31
3: onmyLEA	98	97	100	96	97	97	95	87	88	80	72	62	55	57	58	66	64	67	67	67	67	68	67	63	63	61	35	32
4: sasaLCA	98	97	96	100	99	99	97	88	88	80	71	62	57	58	59	67	65	67	68	68	68	67	66	62	62	62	33	30
5: onmyLBA	99	98	97	99	100	100	98	89	89	81	72	63	58	59	60	68	66	68	69	70	69	68	67	63	63	63	34	31
6: sasaLGA	99	98	97	99	100	100	98	89	89	81	72	63	58	59	60	68	66	68	69	70	69	68	67	63	63	63	34	31
7: onmyLCA	97	96	95	97	98	98	100	87	87	79	71	61	55	57	58	66	64	66	67	67	67	66	65	61	61	62	33	30
8: sasaLDA	89	88	87	88	89	89	87	100	97	78	68	63	51	53	53	61	59	61	63	63	62	59	61	60	58	59	31	31
9: onmyLDA	91	89	88	88	89	89	87	97	100	76	68	61	50	52	53	60	57	60	62	62	61	60	61	61	59	60	31	31
10: sasaLIA	81	80	80	80	81	81	79	78	76	100	74	66	59	61	59	69	68	69	71	72	71	67	67	62	61	59	34	31
11: onmyLAA	72	71	72	71	72	72	71	68	68	74	100	61	53	54	54	60	59	60	61	63	63	60	61	56	54	55	30	26
12: DR10_LCA	63	63	62	62	63	63	61	63	61	66	61	100	51	53	52	64	63	63	64	65	63	61	61	58	57	54	36	27
13: DR18_LOA	57	57	55	57	58	58	55	51	50	59	53	51	100	80	71	80	79	76	74	77	78	75	73	53	55	45	34	27
14: DR20_LPA	58	58	57	58	59	59	57	53	52	61	54	53	80	100	74	78	79	78	77	74	77	74	75	55	55	46	30	26
15: DR27_LKA	59	59	58	59	60	60	58	53	53	59	54	52	71	74	100	77	78	77	73	74	73	74	76	54	53	47	30	27
16: DR19_LHA	67	67	66	67	68	68	66	61	60	69	60	64	80	78	77	100	97	89	85	90	86	83	84	64	67	54	36	24
17: DR29_LIA	65	65	64	65	66	66	64	59	57	68	59	63	79	79	78	97	100	90	86	88	86	84	85	61	65	53	36	24
18: DR28_LGA	67	67	67	67	68	68	66	61	60	69	60	63	76	78	77	89	90	100	86	86	83	83	87	63	65	55	36	28
19: DR22_LJA	68	68	67	68	69	69	67	63	62	71	61	64	74	77	73	85	86	86	100	88	84	85	84	61	65	57	37	28
20: DR17_LMA	68	68	67	68	70	70	67	63	62	72	63	65	77	74	74	90	88	86	88	100	88	86	84	64	67	58	38	27
21: DR23_LEA	68	68	67	68	69	69	67	62	61	71	63	63	78	77	73	86	86	83	84	88	100	85	83	62	63	53	36	26
22: DR24_LFA	67	67	68	67	68	68	66	59	60	67	60	61	75	74	74	83	84	83	85	86	85	100	88	64	64	54	38	30
23: DR25_LDA	66	66	67	66	67	67	65	61	61	67	61	61	73	75	76	84	85	87	84	84	83	88	100	65	65	55	38	29
24: DR11_LBA	64	64	63	62	63	63	61	60	61	62	56	58	53	55	54	64	61	63	61	64	62	64	65	100	86	53	31	28
25: DR12_LAA	64	64	63	62	63	63	61	58	59	61	54	57	55	55	53	67	65	65	65	67	63	64	65	86	100	52	35	27
26: ON9	63	62	61	62	63	63	62	59	60	59	55	54	45	46	47	54	53	55	57	58	53	54	55	53	52	100	29	31
27: sasaUBA0301	34	34	35	33	34	34	33	31	31	34	30	36	34	30	30	36	36	36	37	38	36	38	38	31	35	29	100	33
28: HLA-A2	31	31	32	30	31	31	30	31	31	31	26	27	27	26	27	24	24	28	28	27	26	30	29	28	27	31	33	100

Percentage sequence identity plot for individual domains created in ClustalX 2.0.11 using amino acids sequences. Information on individual sequences can be found in legend to Text S5a.

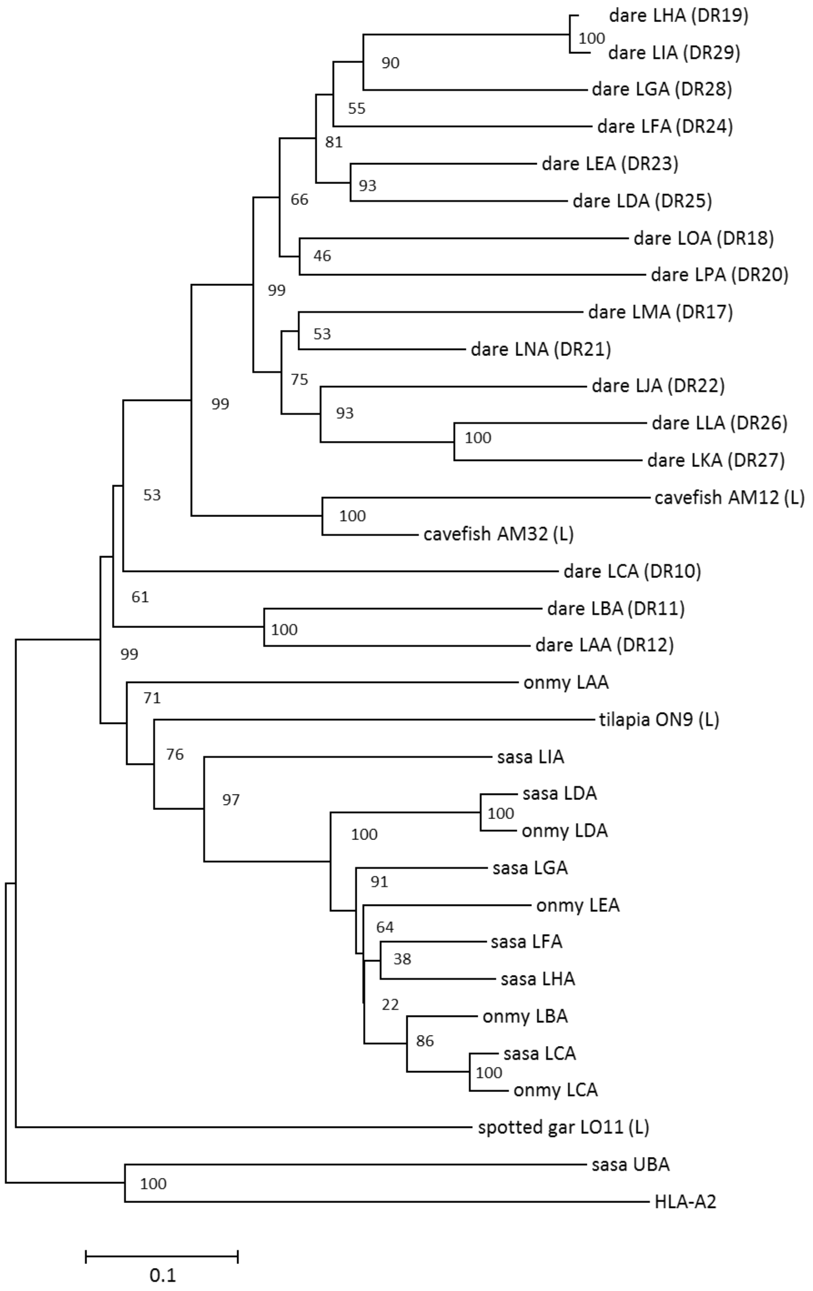

Text S5c. Phylogenetic tree of deduced L lineage amino acid sequences

Phylogenetic tree of deduced alpha 1 through alpha 3 domain MHCI L lineage amino acid sequences. The evolutionary history was inferred using the Neighbor-Joining method [main text reference 95]. The percentage of replicate trees in which the associated taxa clustered together in the bootstrap test (1000 replicates) are shown next to the branches [96]. The tree is drawn to scale, with branch lengths in the same units as those of the evolutionary distances used to infer the phylogenetic tree. The evolutionary distances were computed using the p-distance method [97] and are in the units of the number of amino acid differences per site. All ambiguous positions were removed for each sequence pair. Evolutionary analyses were conducted in MEGA5 [98]. Sequence names reflect species Latin names where sasa is *Salmo salar* (Atlantic salmon), onmy is *Oncorhynchus mykiss* (rainbow trout), dare is *Danio rerio* (zebrafish), tilapia is *Oreochromis niloticus*, cavefish is *Astyanax mexicanus*, and spotted gar is *Lepisosteus oculatus*. Zebrafish L lineage genes have locus designation from Dirscherl et al. [main text reference 38] and rainbow trout (onmy) sequences are from Dijkstra et al.2007 where where GenBank accession numbers are shown in additional file 4: Text S2. Sequence references not found in additional files 3: Text S1 and 4: Text S2 are as follows: salmon sasa*UBA* (**0301* allele) is AAN75116.1 and human HLA-A2 is AAA76608.2. Note that ON9, which lacks the intron between the alpha 1 and 2 domains (see S5d), maps with the salmonid L group that lost further introns, and apart from trout LAA which does have all MHCI consensus introns.

Text S5d. Exon intron organization of selected teleost L lineage genes

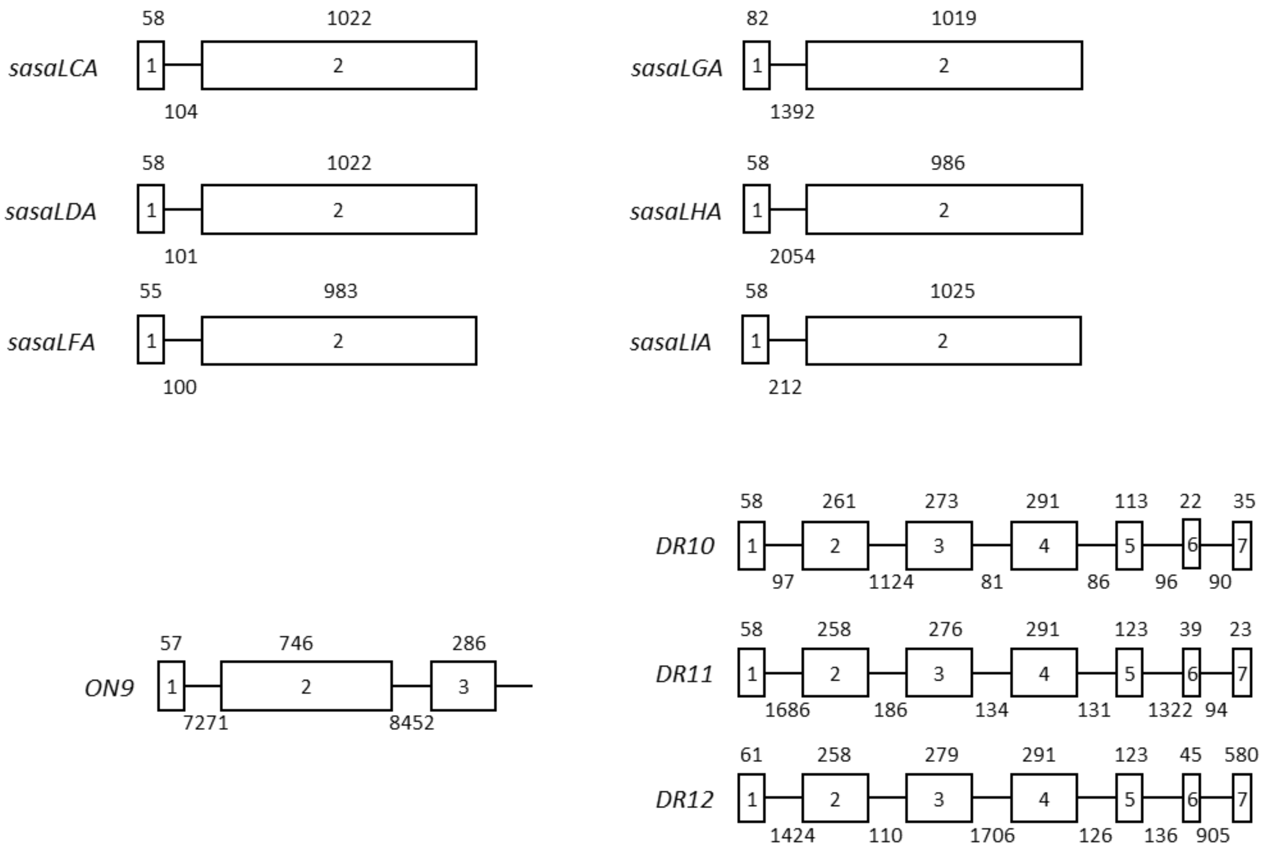

Exon intron organization of salmon, tilapia and selected zebrafish L lineage genes. Exon sizes are shown as boxes with size in base pairs shown above the box while intron sizes are shown below. sasa is *Salmo salar* (Atlantic salmon), DR is *Danio rerio* (zebrafish) and ON is *Oreochromis niloticus* (tilapia).

Text S5e. Hydrophobicity scores for teleost MHC class I α1α2 domains

Gene	U lineage	Z lineage	L lineage	S lineage	P lineage
1	sasaUBA0301 = -0,691	sasaZAAa = -0,739	sasaLCA =-0,341	sasaSAA = -0,528	TN3 = -0,472
2	sasaUHA1 = -0,685	sasaZBAa = -0,807	sasaLDA = -0,322	AM33 = -0,241	TN5 = -0,511
3	sasaUDA = -0,641	sasaZCAa = -0,821	sasaLFA = -0,200	AM34 = -0,406	TR6 = -0,661
4	sasaUGA = -0,682	sasaZDAa = -0,819	sasaLGA =-0,413	AM38 = -0,381	TR9 = -0,630
5	sasaULA = -0,716	sasaZBAb = -0,797	sasaLHA = -0,237	AM39 = -0,437	TR10 = -0,604
6	DR13 = -0,709	sasaZCAb = -0,812	sasaLIA = -0,309	AM40 = -0,461	TR14 = -0,612
7	DR14 = -0,763	sasaZDAb = -0,798	onmyLAA = -0,565	AM41 = -0,609	TR18 = -0,635
8	DR16 = -0,704	DR1 ZEA = -1,072	onmyLBA = -0,419	AM42 = -0,549	TR22 = -0,584
9	OL10 = -0,696	DR2 ZAA = -1,067	onmyLCA = -0,352		TR26 = -0,569
10	OL11 = -0,683	DR3 ZCA = -1,061	onmyLDA = -0,291		TR29 = -0,584
11	OL13 = -0,595	DR4 ZBA = -1,078	DR10 LCA = -0,352		CodPAA = -0,522
12	OL18 = -0,567	DR5 ZFA = -0, 981	DR11 LBA = -0,502		
13	OL19 = -0,764	DR8 ZIA = - 0,957	DR12 LAA = -0,541		
14	ON3 = -0,632	DR9 ZJA = -1,110	DR17 LMA = -0,374		
15	ON27 = -0,569	DR25 ZKA = -0,991	DR18 LOA = -0,319		
16	ON39 = -0,772	OL4 = -0,866	DR19 LHA = -0,321		
17	ON43 = -0,787	OL5 = -0,929	DR20 LPA = -0,365		
18	GA4 = -0,826	OL7 = -0,846	DR22 LJA = -0,708		
19	GA13 = -0,673	OL17 = -0,779	DR23 LEA = -0,479		
20	GA17 = -0,607	ON22 = -0,847	DR24 LFA = -0,263		
21	GA20 = -0,812	ON23 = -0,815	DR25 LDA = -0,466		
22	GA21 = -0,805	ON24 = -0,864	DR27 LKA = -0,258		
23	GA23 = -0,767	ON35 = -0,861	DR28 LGA = -0,384		
24	GA26 = -0,986	GA18 = -0,703	DR29 LIA = -0,311		
25	TR7 = -0,531	TN13 = -0,851	ON9 = -0,320		
26	TR13 = -0,642	TR21 = -0,988			
Average Hydrophobicity	-0,704	-0,895	-0,377	-0,452	-0,580

Hydrophobicity scores are given as GRAVY scores (grand average of hydropathy) as follows: Hydrophobicity was calculated at the website hydrophobicity calculator "Protein GRAVY" (http://www.bioinformatics.org/sms2/protein_gravy.html)[Stothard P. The sequence manipulation suite: JavaScript programs for analyzing and formatting protein and DNA sequences, Biotechniques 28 (6);1102-1104, 2000]. The grand average of hydropathy (GRAVY) value for MHCI α1α2 domain protein sequences was calculated by adding the hydropathy value for each residue and dividing by the length of the sequence [Kyte J. and Doolittle R.F. A simple method for displaying the hydropathic character of a protein, J.Mol.Biol.157 (1);105-132, 1982]. Sequence names reflect Latin species names and are as follows: sasa is *Salmo salar* (Atlantic salmon), onmy is *Oncorhynchus mykiss* (Rainbow trout), DR is *Danio rerio* (zebrafish), ON is *Oreochromis niloticus* (tilapia), GA is *Gasterosteus aculatus* (stickleback), TR is *Takifugu rubripes* (fugu), TN is *Tetraodon nigroviridis* (tetraodon), OL is *Oryzias latipes* (medaka) and AM is *Astyanax mexicanus* (cavefish). Sequences with gravy values below -0,5 are shaded blue while those with values above -0,5 are shaded pink.

Text S5f. Sequence and hydrophobicity of selected vertebrate MHC I α1α2 domains

```
>human HLA-A2 AAA76608.2 Gravy: -0,902
GSHSMRYFFTSVSRPGRGEPRFIAVGYVDDTQFVRFDSDAASQRMEPRAPWIEQEGPEYW
DGETRKVKAHSQTHRVDLGTLRGYYNQSEAGSHTVQRMYGCDVGSDWRFLRGYHQYAYDG
KDYIALKEDLRSWTAADMAAQTTKHKWEAAHVAEQLRAYLEGTCVEWLRRYLENGKETLQ
RT
>human HLA-E BAB63328.1 Gravy: -0,911
GSHSLKYFHTSVSRPGRGEPRFISVGYVDDTQFVRFDNDAASPRMVPRAPWMEQEGSEYW
DRETRSARDTAQIFRVNLRTLRGYYNQSEAGSHTLQWMHGCELGPDRRFLRGYEQFAYDG
KDYLTLNEDLRSWTAVDTAAQISEQKSNDASEAEHQRAYLEDTCVEWLHKYLEKGKETLL
HL
>human CD1a NP_001754.2 Gravy: -0,408
EPLSFHVTWIASFYNHSWKQNLVSGWLSDLQTHTWDSNSSTIVFLCPWSRGNFSNEEWKE
LETLFRIRTIRSFEGIRRYAHELQFEYPFEIQVTGGCELHSGKVSGSFLQLAYQGSDFVS
FQNNSWLPYPVAGNMAKHFCKVLNQNQHENDITHNLLSDTCPRFILGLLDAGKAHLQRQ
>human CD1b NP_001755.1 Gravy: -0,056
GPTSFHVIQTSSFTNSTWAQTQGSGWLDDLQIHGWDSDSGTAIFLKPWSKGNFSDKEVAE
LEEIFRVYIFGFAREVQDFAGDFQMKYPFEIQGIAGCELHSGGAIVSFLRGALGGLDFLS
VKNASCVPSPEGGSRAQKFCALIIQYQGIMETVRILLYETCPRYLLGVLNAGKADLQRQ
>human CD1c AAA51941.1 Gravy: -0,242
EHVSFHVIQIFSFVNQSWARGQGSGWLDELQTHGWDSESGTIIFLHNWSKGNFSNEELSD
LELLFRFYLFGLTREIQDHASQDYSKYPFEVQVKAGCELHSGKSPEGFFQVAFNGLDLLS
FQNTTWVPSPGCGSLAQSVCHLLNHQYEGVTETVYNLIRSTCPRFLLGLLDAGKMYVHRQ
>human CD1d NP_001757.1 Gravy: -0,449
RLFPLRCLQISSFANSSWTRTDGLAWLGELQTHSWSNDSDTVRSLKPWSQGTFSDQQWET
LQHIFRVYRSSFTRDVKEFAKMLRLSYPLELQVSAGCEVHPGNASNNFFHVAFQGKDILS
FQGTSWEPTQEAPLWVNLAIQVLNQDKWTRETVQWLLNGTCPQFVSGLLESGKSELKKQ
>human CD1e P15812.2 Gravy: -0,187
EQLSFRMLQTSSFANHSWAHSEGSGWLGDLQTHGWDTVLGTIRFLKPWSHGNFSKQELKN
LQSLFQLYFHSFIQIVQASAGQFQLEYPFEIQILAGCRMNAPQIFLNMAYQGSDFLSFQG
ISWEPSPGAGIRAQNICKVLNRYLDIKEILQSLLGHTCPRFLAGLMEAGESELKRK
>chicken CD1a BAF63013.1 Gravy: -0,138
GSHMLKLLHFATFQNSTSVLVGGLGLLGDVKMGSLDSRTGNIRYYRPWLRPSLPKGDWDV
IESSIKSYVRDFSRLVQMYTTVPYPFVFQSSIGCELQSNGTIRTFFDIAYEGQNFLRFNL
DAGTWDQMQHNQLSAKAEHLMANASTLNEVIQVLLNDTCVDILRLFIQAGKADLERQ
>chicken CD1b NP_001019753.1 Gravy: -0,078
ESQFFQLFYTLLLGNVSSTELTGMALLADVPIMVLDPHTWNLNICRPWVQEITAETEVKK
ILSFSMVGIRNTIRFMHEMTAKAGLDYPRVFQIHTGCKLYTNGTRWSFVNIGEGGRDLVT
YELSRERWVPQRSTLLAKVMSNTLTDLRAVSGFLEHVFSSSFPNYILMLHEEGRTDLERR
```

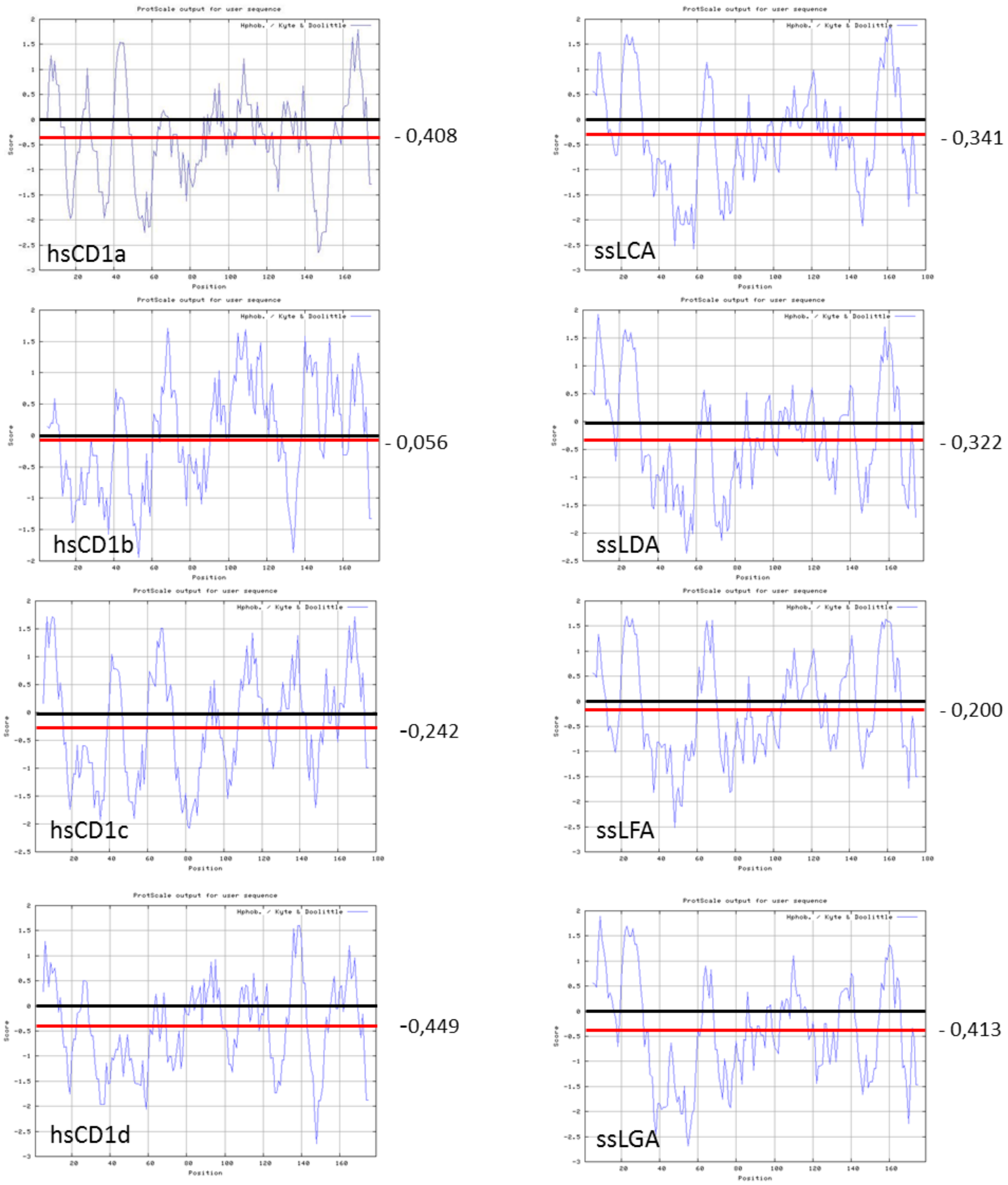

Text S5g. Hydrophobicity distribution for selected α1α2 domains

Kyte and Doolittle hydrophobicity plots of L lineage and other relevant MHC class I sequences. The black line shows the baseline of null charge (Gravy=0) while the red line defines the average hydrophobicity of the respective gene. Numerical average is shown on the right hand side of each plot and also listed in Table 1. CD1b is by far the most hydrophobic molecule while HLA-A2 defines the least hydrophobic molecule. Note that the scale varies between images providing a visual imbalance. Hydrophobicity plots were made using ProtScale (http://web.expasy.org/cgi-bin/protscale/protscale.pl). The sequence names reflect species Latin names i.e. hs is *Homo sapiens*, ss is *Salmo salar*, gg is *Gallus gallus* and ON is *Oreochromis niloticus*.

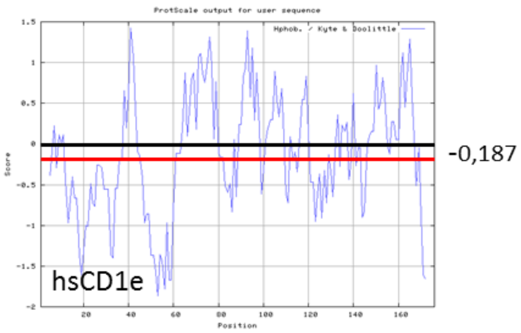
ProtScale output for user sequence
Hphob. / Kyte & Doolittle
Score
Position
hsCD1e
-0,187

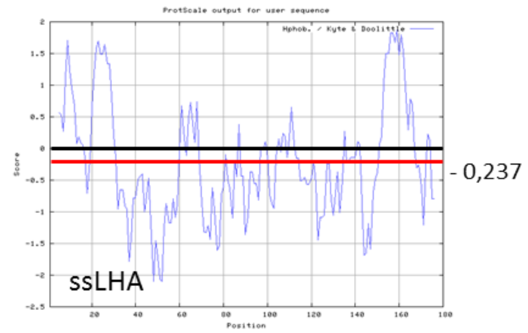
ProtScale output for user sequence
Hphob. / Kyte & Doolittle
Score
Position
ssLHA
- 0,237

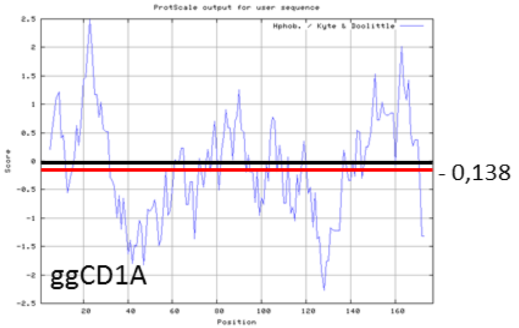
ProtScale output for user sequence
Hphob. / Kyte & Doolittle
Score
Position
ggCD1A
- 0,138

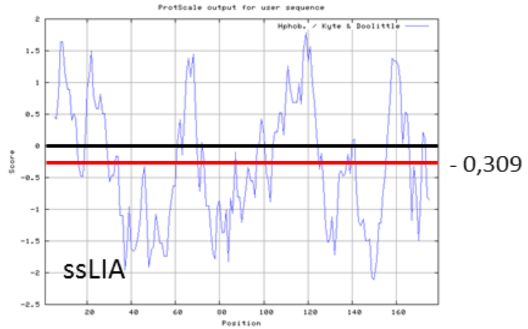
ProtScale output for user sequence
Hphob. / Kyte & Doolittle
Score
Position
ssLIA
- 0,309

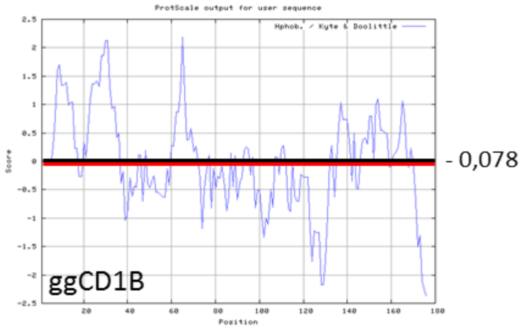
ProtScale output for user sequence
Hphob. / Kyte & Doolittle
Score
Position
ggCD1B
- 0,078

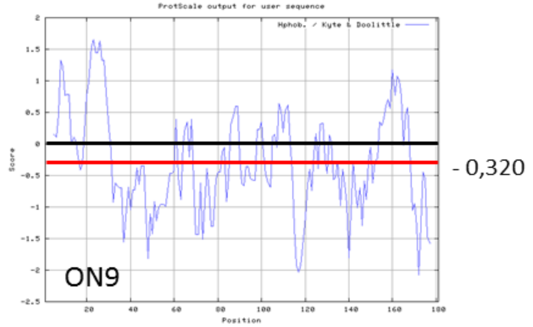
ProtScale output for user sequence
Hphob. / Kyte & Doolittle
Score
Position
ON9
- 0,320

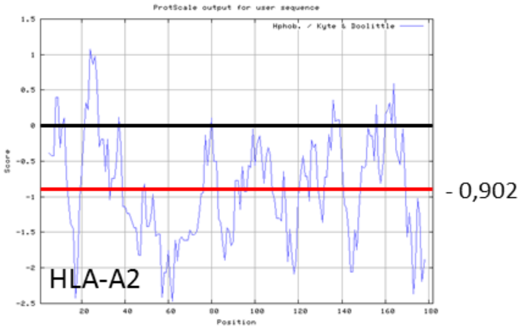
ProtScale output for user sequence
Hphob. / Kyte & Doolittle
Score
Position
HLA-A2
- 0,902

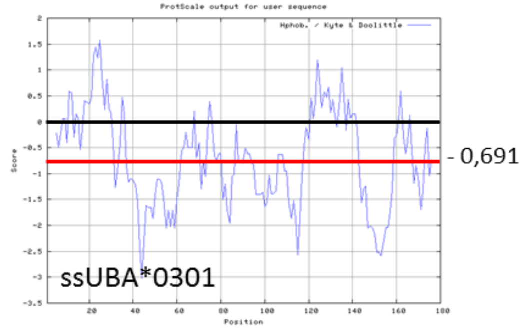
ProtScale output for user sequence
Hphob. / Kyte & Doolittle
Score
Position
ssUBA*0301
- 0,691
