# Supplementary material for: A comprehensive analysis of teleost MHC class I sequences
Source: BMC Evol Biol. 2015 Mar 6;15:32. doi: 10.1186/s12862-015-0309-1 (PMC4364491; doi:10.1186/s12862-015-0309-1)
Supplement: Additional file 9: — Text S6. Additional S lineage data. [file 12862_2015_309_MOESM9_ESM.pdf]

Additional file 9: Text S6. Additional S lineage data

Table of contents		Page
Text S6a	Alignment of deduced S lineage amino acid sequences	1
Text S6b	Percent identity per domain between deduced S lineage amino acid sequences	4
Text S6c	Phylogenetic tree of deduced S lineage domain amino acid sequences	5

Text S6a. Alignment of deduced teleost S lineage amino acid sequences

Amino acid sequence alignment of S lineage sequences, including the first trout S lineage sequence initially denoted *UAA* by Shum et al. [main text reference 27], compared to salmon *UBA* and human HLA-A2 sequences. Individual domains are shown above and bordered with a gap. Human HLA-A2 residues known to anchor peptides i.e. Y7, Y59, Y84, T143, K146, W147, Y159, Y171 are shaded red. HLA-A2 residue positions known to contribute to the six pockets A through F [main text reference 1 and 3] are indicated above the alignment. Numbering above the alignment relates to the mature HLA-A2 sequence. The number of amino acid residues is shown on the right hand side of each sequence. A line is introduced to separate the S lineage sequences from the rest. Residues are colored according to their physiochemical properties while unusual cysteines are highlighted with cyan shading. Also note the added residues in the beginning of the alpha 2 domain found in some cavefish and channel catfish sequences and the short cytoplasmic domains. Conserved glycosylation sites are underlined. Sequence names mostly reflect species Latin name where sasa is *Salmo Salar* (Atlantic salmon), onmy is *Oncorhynchus mykiss* (rainbow trout), ip and icpu is *Ictalurus punctatus* (channel catfish), Ayu is *Plecoglossus altivelis* and AM is *Astyanax mexicanus* (blind cavefish). Apart from onmy*UAA* (allele *0101, GenBank accession CCE21324.1), sasa*UBA* (allele*0301, GenBank accession AAN75116.1) and HLA-A2 (human, GenBank accession AAA76608.2) sequence references not shown in the alignment can be found in additional files 3: Text S1 and 4: Text S2. Abbreviations are as follows: CP is connecting peptide, TM is transmembrane region and CYT is cytoplasmic domain.

```
              Leader sequence              Alpha 1 domain
                                            1        *         20        *         40        *         60
                                                  A B                                                      A  A
                                                A B C            C B         B          B              A   B  BB
HLA-A2     : ----------------------------- GSHSMRYFFTSVSRPGRGEPRFIAVGYVDDTQFVRFDSDAASQRMEPRAPWIEQ-EGPEYWDGETRKVK : 68
sasaUBA    : ----------MKCFILLLLGIALHSS-SA ATHSLRYVYTATS-GIPDFPEFVTVGLVNGEPISYYDSI--IRRETPRQDWMAKTEGSDYWESQTQVSI : 84
sasaSAA    : -------------MITTILISFMQFS-IV APHSLHRHCIATQ-GTL-YPKNIQLVMIDDVTVYYYNSS--AEQEAVVPEWLNHLEGIEFWQEVNRNLK : 80
onmyUAA    : -------------MITTILISFMQFS-IV APHSLHRHCIATQ-GTL-YPKNIQLVMIDDVIVYYYNSS--AEQEAVVPEWLNHPEGIEFWQEVHRNLK : 80
AyuJP74795 : --MFLSATTCTARMISFFFVFVFTYS-NA EPHSLHRHCIATQ-GIS-YHKHIQFVMIDDHIIYYYNSS--SDLGSPMPEWLDHSEGSEFWKEFTRNLK : 91
AM33 (S)   : --MEKMKESYIHCVLLLLLLHLLQLS-GG EVNSITAYFIGIQ-GLN-LPDYMERIAVNDVTMFYYDSS--MKDEVSCPDWLNTSSGKQHWMDINLISL : 91
AM34 (S)   : ---------MKKLYCLFFCFHLLQISTGG EFHSFSFFFIATQ-GLN-FPSYIERVTVDDVTMFYYDSS--MTVEPLCPQWLNTTEGLQQWKNMNDRAK : 85
AM38 (S)   : ---MCAEAMKKSNLLCLLLVYLQQDS--- GSLNLCISVVKTS-GFNTIPEYIQKNTLNDVTIFHYDSD--MDSKMPCPDWINSTAARREWNYMQFQTD : 89
AM39 (S)   : -----------MKYICILLLLLQQVKTDD LRYDSCAYYMVSK-GLG-LPEFSERRVLNDVTVYHHDSS--LDSKMPCPDWINTTAGKEHWKIIYHWTD : 83
AM40 (S)   : ---------MKNANLLFLLLYLQQVTT-D LRYDNCCYWMVSK-GYD-LPEFTERRVLNEVTVYYHDSS--LKSKMPCPDWINTTAGIYEWGQIQYWSN : 84
AM41 (S)   : ----------------------------- IRYDSCAHWTYSK-GYG-LPEYTERRVLNDVTVYYHVSR--QKSKMPCPDWINTTAEKELWGTIHDWTD : 65
AM42 (S)   : ----------------------------- IRYDNCAYWTFSK-GFG-LPEFTERLALNDVTVYYHDSS--LKSKMPRPDWINTTAGKDMWRIIHDWTE : 65
ipJT320438 : MRHCININKATIIHTLVLFLHL-QQS-RC DVQSFSLYLLRSE-GLS-LPRYTQSVTVNDVTLYSFDSN--MKSTGPCPEWLNTTAGQQLWKEASFLFH : 92
ipFD361597 : MRHRLNINKATIIHTLVLFLHL-QQS-RC DVHSISCYFLGSE-GLG-LPHYSESVTVNDVTLFYYNSN--MKSTGPCPKWLNTTAGQQLWKETSFLSH : 92
ipFD354405 : ----------------------------- -----------------------QSVTVNDVTLYSFDSN--MKSTGPCPEWLNTTAGQQLWKEASFLFH : 44
ipJT245151 : ------MKERKTLFILLFFLHPVQLF-GD DVHSISGFFISSQ-GLS-LPNYMERITVNDVTIFYHDSG--KNIIAPCPKWLNTTTGQQHWENIRVISQ : 87
ipFD018561 : ----------------------------- -----------------------ERITVNDVTIFYHDSG--KNIIAPCPKWLNTTTGQQYWENIRVISQ : 44
```

```
                                   Alpha 2 domain
              *         80        *             100        *         120       *         140       *          160
                                               A
                                               B               C C
              B  C                           C C               D E                              E        DD  A
              C  FC  F  FF  F              F E D               E F F    FF                  F  FF     E  EE  DD
HLA-A2     : AHSQTHRVDLGTLRGYYNQSEA ---GSHTVQRMYGCDVGSD-WRFLRGYHQYAYDGKDYIALKEDLRSWTAADMAAQTTKHKWEAA-HVAEQLRAYL : 160
sasaUBA    : GSEQTFKANIDVAKQRFNQTG ----GVHVNQKMYGCEWDDETGV-TEGFDQDGYDGEDFLAFDLKTLTWIAPTPQAVITKLKWDSNTAQNEYRKNYL : 176
sasaSAA    : FSRFVMDTAVRVTSEHYNHS- ----HDHFYQAHGRCGWKSD-GT-TEAFMSHAYDGKDFVSFDVSTRTWTAAVSHAVFYKRKRETDLEDLVRLVIHY : 170
onmyUAA    : FNRYVMDTAVRVTSEHYNHS- ----HDHFYQAHGRCGWKSD-GT-TEAFMSHAYDGKDFVSFDVSTRTWTAAVSHAVFYKRKRETDLEDLVRLVIHY : 170
AyuJP74795 : YNRHVMEKAVQLTSEHFNHS- ----HDHVYQAQGHCGWNTD-GT-RVAYMSHAYDGKDFISFDVQNKQWTAVVPEAHFYKTSRQQHSEDLDRITNHY : 181
AM33 (S)   : HNKHSMATALKSAILQFNQT- ASSSDVNIYQGYGRCSVYPN-GT-LKALLTHAFNGKDFLTFDVDRKSYIASVPQAVIYKRQREANPVWLEIMASFY : 185
AM34 (S)   : YNNHYLSSALESIVKQFNQT- ----ETNIYQGYSHCNIFPN-GT-RKAAFTQAFRGKDFFSLDIDRKTYVASVPQAVVYKRQREANTVLLETVVSFY : 175
AM38 (S)   : RISYHITQGFESAVQQFNRT- GSLADRNIYQARGCCVLYPN-GT-SASSLTHTFNGKDFIGFDFDRKTFVAPVHEAVVYKHQRETRSSLFN-VLHFF : 182
AM39 (S)   : YNKYVGTLGLQSATEQFNQT- GSLTDGNIYQAFGCCSLYPN-GT-YRTLLAHAFNGKDFASFDFQTKTFVAAVPQAVLYKTLREKDPANMEEIATLY : 177
AM40 (S)   : HNRFVGTLGLQSAINQFNLT- GTLTDRNIYQGFGCCSLYPN-GT-YSAFLNHAFNGKDFTSFDINSKTFVAAVPQAVVYKTLRESDQTTINYVASYY : 178
AM41 (S)   : HNIEITTLGFQTATQQFNLT- GSLSDRNIYQVTGCCSLYPN-GT-YTAFMNHAFNGKDFTSFDINTKTFVAAVPQAVRYKTLRESQPLTIDEVAEFY : 159
AM42 (S)   : NNRYGGTLQFHTATQQFNLT- GLLTDQNIYQASGCCSVYPN-GT-YTAFLTYAFNEKDFASFDINTKTFVPAVPQAVFYKNLRERDSLRVDMAVKYY : 159
ipJT320438 : RNMANMDLALQTAKSQFNLT- GSHADINVYQAYSRCDLYPD-GT-TKSSLTHAFNGKDFLSLDMDSKTYIASVPQALIYKSIRDKDIIWLEIVVSYY : 186
ipFD361597 : HNMANMDLALQTAKSQFNLT- GSHVDINVYQGYSRCDLYPD-GT-TKSSLTHAFNGNDFLSLDIDSKTYIASVPQALIYKRAREKNIIWLEKLVSFY : 186
ipFD354405 : RNMANMDLALQTAISQFNLT- GSHADINVYQAYSRCDLYPD-GT-TKSSLTHAFNGKDFLSLDMDSKTYIASVPQALIYKSIRDEDIIWLEIVVSYY : 138
ipJT245151 : HNKQMIATAIESAIQQFNLT- GSHADINIYQGYSRCDLYPN-GT-MKASLAHAFNGKDFLSLDFDSKTYITSVPQALKYKRLREQNSVLFGITVSFY : 181
ipFD018561 : HNKQMIATAIESAIQQFNLT- GSHADINIYQGYSRCDLYPN-GT-MKASLAHAFNGKDFLSLDFDGKTYITSVPQALKYKRLREQNSVLFGITVSFY : 138
ipCK423282 : --------------------- ------------------------TKSSLIHAFNGKDFLSLDIDSKMFIASVPQALIYKSIREKDKIWLETVVSFY :  52
ipFD042554 : -NKQMIATAIESAIQQFNLT- GSHADINIYQGYSRCDLYPN-GT-MKASLAHAFNGKDFLSLDFDSKTYITSVPQALKYKRLREQNSVLFGITVSFY :  93
```

```
                                   Alpha 3 domain
                  *         180         *         200         *         220         *         240         *
              A   A   A
HLA-A2      : EGTCVEWLRRYLENGKETLQRT DAPKTHMT--HHAVS-DHEATLRCWALSFYPAEITLTWQRDG-EDQTQDT-ELVETRPAGDGTFQKWAAVVVPSG : 252
sasaUBA     : TQTCIEWLKKYLDYGKSTLMRT VPPSVSLL--QKTPS----SPVTCHATGFYPSGVMVSWQKDG-QDHHEDV-EHGETLQNDDGTFQKSSHLTVT-P : 264
----------------------------------------------------------------------------------------------------------------
sasaSAA     : ESGCIRWLKKLLQFSVTFREPK V-PAVSLF--ERPPHGNSEVEVTCHVTGFYPRAVQVEWLGAEGLPMV-DGVSSGEVLPNGDGSYQLRKSLTVP-Q : 262
onmyUAA     : ESGCIRWLEKLLEFSVTVREPK V-PAVSLF--ERPPHGNSEVEVTCHVTGFYPRAVQVEWLGAEELPMV-DGVNSGEVLPNGDGSYQLRKSLTVP-Q : 262
AyuJP74795  : ESECILWLKKLLQFSSKLLKPK A-PDVSLF--ERP--SSSEVLVTCHVTGFYPKEVQVEWLGE-EGHPLVQEVRRGEVLPNEDGTYQLRTILAVP-L : 271
AM33 (S)    : KKTCFERLKMFLQHASVHITKK V-PEVHLF--KSF--KSGSSVLACHVTGFYPKEVQVEWIGA-GLQPV-DG-EVIEVLPNGDGTYQTRRSVIRP-E : 273
AM34 (S)    : RTTCFERLKMFLEHAPEVRVKK G----WIF--ERA--ESSSSVLTCHVTGFKPKQVQVEWIGA-GLQPV-DG-EITDMLPNGDGTYQTRRSVIRPRE : 261
AM38 (S)    : GTQCVQNIQ-LFKTAPAILERK V-PEVRIF-QQKK--RAGSVTVTCHVTGFYPREVQVFWLGS-DLQPV-DE-GVTEILPNGDGTYQTRKSVIVP-E : 270
AM39 (S)    : KKTCLERLQ-ILKEAPRVNIKK V-PEVRII--EKK--RTGSVTVTCHVTGFYPREVQVIWLGS-DLQPVVKG--VTEALPNDDGTYQTRKSVIVP-E : 264
AM40 (S)    : QKTCLDRLK-ILKEAPRVRDKR ---------------------------------------------------------------------------- : 199
AM41 (S)    : KKTCLERLK-MFKQAPEIHRRK D-PNVWIL-QQKK--RAGSVTVTCHVTGFYPREVQVFWLGP-DLQPV-DE-GVTEILPNGDGTYQTRKSVIVP-E : 247
AM42 (S)    : KKTCLERLK-ILQQDPRVLIRK V-PEVQILEQQKK--KSGSVTVTCHVTGFYPREVQVFWLGS-DLQPV-DE-GVTEILPNGDGTYQTRKSVIVP-E : 248
ipJT320438  : KKRCFDHLRMFLEHAPGVRNKK V-PEVRLF--ERQ--GAGSTLLTCHVTGFYPRAVQVKWIGA-DLQLVEDE--MNHVLPNGDGTFQTRSSVIRP-E : 274
ipFD361597  : KKTRFDRLRMFLEYAPGVRNKK V-PEVRLF--ER------------------------------------------------------------------ : 217
ipFD354405  : KKRCFDHLRMFLEHAPGVRNKK V-PEVRLF--ERQ--GAGSTLLTCHVTGFYPRAVQVKWIGA-DLQLVEDE--MNHVLPNGDGTFQTRSSVIRP-- : 225
ipJT245151  : QKTCFDRLKMFLKHAPGVKMQK E-PKVRLF--ERQ--RAGSTLLTCHVTGFYPRAVQ------------------------------------------ : 233
ipFD018561  : QKTCFDRLKMFLKHAPGVKMQK ---------------------------------------------------------------------------- : 160
ipCK423282  : KKTCFDDLRMFLELAPGVRNKK A-PQVRLF--ERQ--GAGSTILTCHVTGFYPRAVQVKWIGA-DLQLVKDE--INDVLPNGDGTFQTRSSVIRP-E : 140
ipFD042554  : QKTCFDRLKMFLKHAPGVKMQK E-PKVRLF--ERQ--RAGSTLLTCHVTGFYPRAVQVKWIGA-DLQLVDDE--MNDVLPNGDGTFQTRSSVIRP-E : 181
```

```
                                        CP          TM                         CYT
                      260         *         280         *         300         *         320         *         340
HLA-A2      : QEQ---RYTCHVQHEGLPKPLTLRW EPSSQPT-----IPIVGIIAGLVLFGAVITGAVVAAVMWRRKSSDRKGGSYSQAASSDSAQGSDVSLTACKV- : 341
sasaUBA     : EEWKNNKYQCVVQVTGLQEDFIKVL TESEIKTNWNDPNIVLIIGVVVALLLVVVAVVVGVVI-WKKKSKKGFVPASTSDTDS-DNSGRAAQMT----- : 355
-------------------------------------------------------------------------------------------------------------------
ssSAA       : EAQDTQSYSCLVLHSSIAGNITVTW APKKNLA-----NVLMAIVIIVSVVLILTVLFKYLV--RRRAVGKSQS------------------------- : 328
onmyUAA     : EAQDTQSYSCLVLHSSIAGNITVTW APKKNLA-----NVLMAIVIIVSVVLILTVLFKYLV--WRRAVAQNPRG------------------------ : 329
AyuJP74795  : GSQHSLSYSCLVVHSSVQGNITKIW EPKHSRFLK---YWSI-LFICLLFLFFLLAAVGCYV--RKHYYTAVDNH------------------------ : 339
AM33 (S)    : ENPEKHSYSCVVQHSSIAGNITKTW VAEEHSL-----LAVWISLVCILVIIGTGLVL------RKFCRCGQRDTGI---------------------- : 338
AM34 (S)    : ENPEKHSYSCVVQHSSIGGNITKTW VTETRIR-----MGVLASLVCIVLAVIGCGLVF-----RQFCRTK---------------------------- : 321
AM38 (S)    : EDVGKQNYSCVVLHISIPNNITTVW VGQKCGG-----FALWIPLVCISLLACGTGFGV-----WWRCQTRDAVI------------------------ : 334
AM39 (S)    : EDVGKHTYSCVVLHISVPNNITTVW EVKAGG------VAVWIPLLCISLLASVIGIGV-----WWRCKTRDAVI------------------------ : 327
AM41 (S)    : EDVGKQNYSCVVLHISVPNNITTVW DVNMTD------SED------------------------------------------------------------ : 281
AM42 (S)    : EDVGKQNYSCVVLHISVPNNITTVW ERACGG------VWIA-ILGCILLSAAAYISL------KNFKHHCLVI------------------------- : 308
ipJT320438  : ENTGDQRYSCVVHHSSLEGNITVTW GKEE------------------------------------------------------------------------ : 303
ipCK423282  : ENTGDQRYSCVVHHSSLEGNITVTW GKEEKPFR----LYVRITLGCVFIVTVVGLVI------RGFFYNKGQFIVVS--------------------- : 207
ipFD042554  : ENTGDQRYSCVVHHSSLEGNITVTW GKEEKPFR----LYVWITLGCVFIVTVVGLVI------RCILKSKDAGI------------------------ : 245
```

Text S6b. Percent identity per domain between deduced S lineage amino acid sequences

Alpha 1 domain

	1	2	3	4	5	6	7	8	9	10	11	12	13	14	15	16	17	18
1: sasaSAA	100	96	66	28	28	28	29	27	22	24	20	21	19	26	29	36	21	23
2: onmyUAA0101	96	100	67	27	27	27	28	26	22	21	20	22	19	25	27	34	21	23
3: Ayu	66	67	100	27	27	27	28	27	24	19	18	18	19	24	26	35	24	21
4: icpuFD358144	28	27	27	100	100	100	98	80	41	42	42	39	32	54	51	45	24	20
5: icpuFD324696	28	27	27	100	100	100	98	80	41	42	42	39	32	54	51	45	24	20
6: icpuFD361597	28	27	27	100	100	100	98	80	41	42	42	39	32	54	51	45	24	20
7: icpuFD363057	29	28	28	98	98	98	100	80	40	41	41	38	31	53	51	45	24	20
8: icpuJT320438	27	26	27	80	80	80	80	100	40	40	40	39	33	46	41	40	20	20
9: AM39	22	22	24	41	41	41	40	40	100	72	67	68	44	39	42	34	22	20
10: AM40	24	21	19	42	42	42	41	40	72	100	66	67	39	40	40	29	22	15
11: AM41	20	20	18	42	42	42	41	40	67	66	100	72	41	38	35	27	20	17
12: AM42	21	22	18	39	39	39	38	39	68	67	72	100	40	36	38	28	26	20
13: AM38	19	19	19	32	32	32	31	33	44	39	41	40	100	33	29	34	24	20
14: icpuJT245151	26	25	24	54	54	54	53	46	39	40	38	36	33	100	59	48	28	20
15: AM33	29	27	26	51	51	51	51	41	42	40	35	38	29	59	100	53	24	17
16: AM34	36	34	35	45	45	45	45	40	34	29	27	28	34	48	53	100	26	24
17: sasaUBA0301	21	21	24	24	24	24	24	20	22	22	20	26	24	28	24	26	100	31
18: HLA-A2	23	23	21	20	20	20	20	20	20	15	17	20	20	20	17	24	31	100

Alpha 2 domain

	1	2	3	4	5	6	7	8	9	10	11	12	13	14	15	16	17	18	19	20	21	22	23
1: sasaSAA	100	97	63	38	40	37	36	30	37	35	33	33	33	33	36	36	36	36	35	35	35	33	28
2: onmyUAA0101	97	100	61	36	38	38	38	30	35	36	33	33	33	33	38	38	38	38	37	37	37	32	29
3: Ayu	63	61	100	36	36	33	35	30	36	33	32	32	32	32	33	33	33	32	29	29	29	28	25
4: AM41	38	36	36	100	66	73	67	52	57	52	52	52	52	52	45	45	45	48	47	47	47	27	21
5: AM42	40	38	36	66	100	65	66	45	48	46	47	47	47	47	43	43	43	45	42	41	42	27	23
6: AM40	37	38	33	73	65	100	71	49	51	53	54	54	54	53	51	51	51	54	51	51	51	26	20
7: AM39	36	38	35	67	66	71	100	50	54	47	51	51	51	51	51	49	51	54	49	49	49	25	22
8: AM38	30	30	30	52	45	49	50	100	44	44	47	47	47	47	44	44	44	46	40	40	41	20	20
9: AM33	37	35	36	57	48	51	54	44	100	65	64	64	64	64	59	59	58	60	63	61	63	27	24
10: AM34	35	36	33	52	46	53	47	44	65	100	63	63	63	63	58	58	57	61	62	61	62	26	24
11: icpuJT245151	33	33	32	52	47	54	51	47	64	63	100	100	100	99	70	70	69	70	72	73	73	25	22
12: icpuFD039731	33	33	32	52	47	54	51	47	64	63	100	100	100	99	70	70	69	70	72	73	73	25	22
13: IcpuFD042554	33	33	32	52	47	54	51	47	64	63	100	100	100	99	70	70	69	70	72	73	73	25	22
14: icpuFD018561	33	33	32	52	47	53	51	47	64	63	99	99	99	100	69	69	68	69	71	72	72	25	22
15: icpuJT320438	36	38	33	45	43	51	51	44	59	58	70	70	70	69	100	99	99	90	84	84	85	27	24
16: icpuFD354405	36	38	33	45	43	51	49	44	59	58	70	70	70	69	99	100	98	89	83	83	84	27	24
17: icpuFD054080	36	38	33	45	43	51	51	44	58	57	69	69	69	68	99	98	100	89	84	84	85	27	24
18: icpuFD046697	36	38	32	48	45	54	54	46	60	61	70	70	70	69	90	89	89	100	85	85	86	26	25
19: icpuFD361597	35	37	29	47	42	51	49	40	63	62	72	72	72	71	84	83	84	85	100	96	96	28	23
20: icpuFD363057	35	37	29	47	41	51	49	40	61	61	73	73	73	72	84	83	84	85	96	100	96	27	24
21: icpuFD358144	35	37	29	47	42	51	49	41	63	62	73	73	73	72	85	84	85	86	96	96	100	28	24
22: sasaUBA0301	33	32	28	27	27	26	25	20	27	26	25	25	25	25	27	27	27	26	28	27	28	100	40
23: HLA-A2	28	29	25	21	23	20	22	20	24	24	22	22	22	22	24	24	24	25	23	24	24	40	100

Alpha 3 domain

	1	2	3	4	5	6	7	8	9	10	11	12	13	14	15	16
1: sasaSAA	100	96	69	57	57	55	56	56	50	54	54	53	53	55	36	26
2: onmyUAA0101	96	100	67	58	56	54	55	57	51	56	56	55	55	57	34	24
3: Ayu	69	67	100	50	52	50	52	56	51	47	47	49	47	47	37	30
4: AM38	57	58	50	100	95	93	88	64	59	60	62	62	63	63	38	30
5: AM42	57	56	52	95	100	93	87	64	58	57	58	58	58	59	40	30
6: AM41	55	54	50	93	93	100	85	59	58	58	58	59	60	58	39	30
7: AM39	56	55	52	88	87	85	100	60	57	57	58	57	58	59	39	28
8: AM33	56	57	56	64	64	59	60	100	82	65	66	66	66	67	36	28
9: AM34	50	51	51	59	58	58	57	82	100	66	66	65	66	64	31	24
10: icpuCK423282	54	56	47	60	57	58	57	65	66	100	99	95	93	93	36	31
11: icpuGH649617	54	56	47	62	58	58	58	66	66	99	100	93	93	95	36	31
12: icpuFD346410	53	55	49	62	58	59	57	66	65	95	93	100	96	96	36	32
13: icpuFD042554	53	55	47	63	58	60	58	66	66	93	93	96	100	95	36	31
14: icpuJT320438	55	57	47	63	59	58	59	67	64	93	95	96	95	100	36	30
15: sasaUBA0301	36	34	37	38	40	39	39	36	31	36	36	36	36	36	100	31
16: HLA-A2	26	24	30	30	30	30	28	28	24	31	31	32	31	30	31	100

Percentage amino acid sequence identity plot created using ClustalX 2.0.11. Sequence information can be found in legend to text S6a.

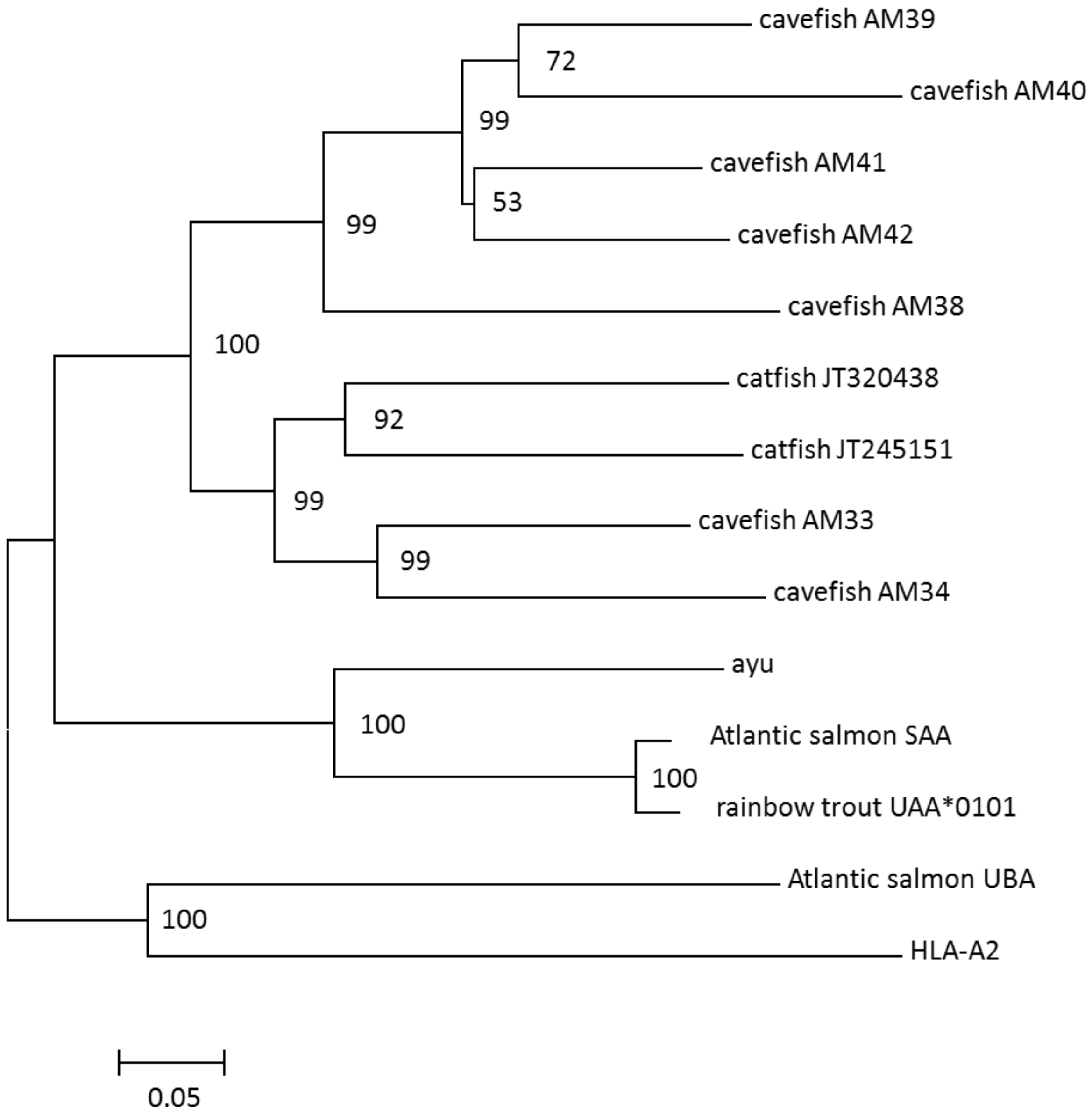

Text S6c. Phylogenetic tree of deduced S lineage domain amino acid sequences

Phylogenetic tree of S lineage alpha 1 through alpha 3 domain amino acid sequences. The evolutionary history was inferred using the Neighbor-Joining method [main text reference 95]. The percentage of replicate trees in which the associated taxa clustered together in the bootstrap test (1000 replicates) are shown next to the branches [96]. The tree is drawn to scale, with branch lengths in the same units as those of the evolutionary distances used to infer the phylogenetic tree. The evolutionary distances were computed using the p-distance method [97] and are in the units of the number of amino acid differences per site. All ambiguous positions were removed for each sequence pair. Evolutionary analyses were conducted in MEGA5 [98]. The trout onmyUAA0101 sequence (GenBank accession CCE21324.1) is the first S lineage sequence published by Shum et al. [main text reference 27] but was renamed in Lukacs et al., [main text reference 29]. See legend to S6a or additional file 4: Text S2 for GenBank sequence accession numbers not shown in the figure. Channel catfish is *Ictalurus punctatus*, cavefish is *Astyanax mexicanus*, Atlantic salmon is *Salmo salar*, rainbow trout is *Oncorhynchus mykiss* and ayu is *Plecoglossus altivelis*. Atlantic salmon UBA is the UBA*0301 allele. See legend to Text S6a for sequence references not provided in the figure. Note that the channel catfish sequences cluster with the AM33-34 clade.
