# Supplementary material for: A comprehensive analysis of teleost MHC class I sequences
Source: BMC Evol Biol. 2015 Mar 6;15:32. doi: 10.1186/s12862-015-0309-1 (PMC4364491; doi:10.1186/s12862-015-0309-1)
Supplement: Additional file 10: — Text S7. Additional P lineage data. [file 12862_2015_309_MOESM10_ESM.pdf]

Additional file 10: Text S7. Additional P lineage data

Table of Contents		Page
Text S7a	Alignment of deduced P lineage amino acid sequences	2
Text S7b	Percent identity per domain between deduced P lineage amino acid sequences	7
Text S7c	Exon intron structure of P lineage genes	8
Text S7d	Phylogenetic tree of deduced P lineage domain amino acid sequences	9

Text S7a. Alignment of deduced P lineage amino acid sequences

Amino acid sequence alignment of selected P lineage sequences from cavefish, fugu, tetraodon, salmon, sablefish, cod and seabass aligned with human HLA-A2 and salmon *UBA* sequences. Dashes indicate missing sequence and unusual cysteines in the alpha 1 domain are shaded cyan. Individual domains, numbering according to mature HLA-A2, and HLA-A2 residues known to constitute the six A through F pockets [main text reference 1 and 3] are shown above the alignment. HLA-A2 residues known to anchor peptide N- and C-terminal ends are shaded red while the remaining residues are colored according to physiochemical properties. Conserved N-linked glycosylation motifs are underlined. Sequence names mostly reflect species Latin names where AM is *Astyanax mexicanus* (cavefish), TR is *Takifugu rubripes* (fugu), TN is *Tetraodon nigroviridis* (tetraodon), LO is *Lepisosteus oculatus* (spotted gar), sasa is *Salmo salar* (Atlantic salmon), cod is *Gadus morhua*, sablefish is *Anoplopoma fimbria* and seabass is *Dicentrarus labrax*. The short sequences TN6, TR23 and LO6 were omitted and internal stop codon in TR10 is shown with a star. Only one P gene was found in cod and thus defined as *PAA* according to MHC nomenclature in teleosts. The seabass sequence reference is GBAA01146398. The remaining GenBank or Ensembl sequence references can be found in Additional files 3: Text S1 and 4: Text S2 including the fugu and tetraodon sequences assembled from individual SRA reads from fugu gills (SRX363279) and tetraodon brain (SRX191169). Numbering above the alignment is according to mature HLA-A2. Abbreviations are: CP for connecting peptide, TM for transmembrane region and CYT for cytoplasmic region.

```
                                                   1        *         20         *         40         *          60
             Leader sequence          Alpha 1 domain
                                           A B
                                         A B C            C B         B          B              A
HLA-A2      : --------------------------GSHSMRYFFTSVSRPGRGEPRFIAVGYVDDTQFVRFDSDAASQRMEPRAPWIEQ-EGPEYWD : 61
sasaUBA0301: --------MKCFILLLLGIALHSSSAATHSLRYVYTATS-GIPDFPEFVTVGLVNGEPISYYDSI--IRRETPRQDWMAKTEGSDYWE : 77
AM5         : --------MRVLLLCVFCFTGETASAEKHSLQYLYTLRS-APEDDTEFEITTVFDGLIISHCKSP--RFRDHSREDWISQTFTNAEWK : 77
AM6         : --------MIFILLSVYCSVLETTSAEKHTLQYLYTLRS-APEDDTEFEITTVFDGLIISHCKSP--RFRDQSRYDWISQTFTDAEWK : 77
TR4         : -----------------------------------------------------------------------HFKPVLVSHNL------ : 11
TR5         : --------------------------GSHSLEFLSTGRV-QPGSGAHFEQVTEFDGVVISHCDSG--TQQEHFKPVLESHNL------ : 53
TR6         : ---------MNIG-LLFWILLPAAECGSHSLEFLSTGRV-QPGSGPHFEQVTEFDGVVISHCDSG--TQQEHFKPVLESHNL------ : 69
TR9         : ---------MNIC-LLFWILLPAAECGSHSLEFLSTGRV-QPGSGPHFEQVTEFDGVVISHCDSG--TQQEHFKPVLESHNL------ : 69
TR10        : --------------------------GSHSLEFLSTGRV-QPGSGPHFEQVTEFDGVVISHCDSG--TQQEHFKPVLESHNL------ : 53
TR14        : --------------------------GSHSLEFLSTGRV-QPGSGPHFEQVTEFDGVVISHCDSG--TQQEHFKPVLESHNL------ : 53
TR15        : ---------MNIC-LLFWILLPAAECGSHSLEFLSTGRV-QPGSGPHFEQVTEFDGVVISHCDSG--TQQEHFKPVLESHNL------ : 69
TR16        : ---------MNIC-LLFWILLPAAECGSHSLEFLSTGRV-QPGSRLHFEQVTEFDGVVISHCDSG--TQQEHFKPVLESHNL------ : 69
TR17        : ---------MNIC-LLFWILLPAAECGSHSLEFLSTGRV-QPGSGLHFEQVTEFDGVVISHCDSG--TQQEHFKPVLESHNL------ : 69
TR18        : ---------MNIC-LLFWILLPAAECGSHSLEFLSTGRF-QPGSGPHFEQVTEFDGVVISHCDSG--TQQEHFKPVLESHNL------ : 69
TR22        : ---------MKFCLLLYWVLLPAAECGSHSLEFLSTGRV-QPGSGPHFEQVTEFDGVVISHCDSG--TQQEHFKPVLESHNL------ : 70
TR24        : --------------------------GSHSLEFLSTGRV-QPGSGAHFEQVTEFDGVVISHCDSG--TQQEHFKPVLESHNL------ : 53
TR26        : --------------------------GSHSLEFLSTGRV-QPGSGPHFEQVTEFDGVVISHCDSG--TQQEHFKPVLESHNL------ : 53
TR27        : --------------------------GSHSLEFLSTGRV-QPGSGPHFEQVTEFDGVVISHCDSG--TQQEHFKPVLESHNL------ : 53
TR28        : ---------MNIC-LLFWILLPAAECGSHSLEFLSTGRV-QPGSGLHFEQVTEFDGVVISHCDSG--TQQEHFKPVLESHNL------ : 69
TR26        : --------------------------GSHSLEFLSTGRV-QPGSGPHFEQVTEFDGVVISHCDSG--TQQEHFKPVLESHNL------ : 53
TR29        : --------------------------GSHSLEFLSTGRV-QPGSGPHFEQVTEFDGVVISHCDSG--TQQEHFKPVLESHNL------ : 53
TR30        : ---------MNIC-LLFWILLPAAECGSHSLEFLSTGRV-QPGSGPHFEQVTEFDGVVISHCDSG--TQQEHFKPVLESHNL------ : 69
TR32        : ---------MNIC-LLFWILLPAAECGSHSLEFLSTGRV-QPGSGPHFEQVTEFDGVVISHCDSG--TQQEHFKPVLESHNL------ : 69
TR34        : -------------------------------------RTCQPGSRPHFEQVTEFDGVVISHCDSG--TQQEHFKPVLESHNL------ : 43
TR_SRA      : --------------------------GSHSLEFLSTGRV-QPGSGPHFEQVTEFDGVVISHCDSG--TQQEHFKPVLESHNL------ : 53
TN3         : --------------------------GSHSLGFLSTGRF-HPGTGLYFEQVTVFDGVLISHCDSR--TPLEQFKAVLESHNL------ : 53
TN4         : --------------------------GFHSLGFLSTGRF-PPGTCPDFEQVTVFDGVLISHCDSR--TQQEQFKAVLESHNL------ : 53
TN5         : --------------------------GSHSLDFLSTGRF-HPGTGLYFEQVTVFDGVLISHCDSR--TQQEQFKAVLESHNL------ : 53
TN7         : --------------------------GSHSLGFLFTGQF-PPGTGPDFEQVTVFDGVLISHCDSR--TQQEQFKAVLESHNL------ : 53
TN_SRA      : ----------------------------------STGRF-PPGTGPDFEQVTVFDGVLISHCDSR--TQQEQFKAVLESHNL------ : 45
Cod_PAA     : MLISIPHWIIFNICNYAYFVQSTEC-DPHSLEFTSMFGP-ESKFRPTFQQLVRFDGVPLSHCESW--SEKALPELRPSLQDV-----Q : 79
Seabass     : ------MQMWIKLLIQWFLMSTAEC-GSHSLEFLSTGRV-QPEDQPQFEQLTVFDGVPISYCNSW--KKREELKPTLESNNL------ : 72
LO2         : --------------------------ASHWLVYTSTAVS-EAGLLSQLLISRVLDQQTVSHWDSA--SWRETHTSHWPSRAAGLRDVN : 67
LO3         : --------------------------ASHWLVYSSTAVS-EAGLLSQLLISRVLDQQTVSHWDSA--SRRETRTRHWPSGAAGLRDIN : 79
LO4         : --------------------------ASHSLVYTSTAVS-EAGLLSQLLISRVLDQQTVSHWDST--SQRETRTRHWPSGAAGLEDID : 59
```

```
                     *         80        *          100         *         120       *         140
                                          Alpha 2 domain
                                                  A
                                                  B
                                                  C                C C
              A  A   B  C                       C C                D E
              B  BB  C  FC  F  FF  F          F E D                E F F    FF                  F  F
HLA-A2     : GETRKVKAHSQTHRVDLGTLRGYYNQSEAGSHTVQRMYGCDVGSD--WRFLRGYHQYAYDGKDYIALKEDLRSWTAADMAAQTTKHK : 146
sasaUBA0301: SQTQVSIGSEQTFKANIDVAKQRFNQTG-GVHVNQKMYGCEWDDE-TGV-TEGFDQDGYDGEDFLAFDLKTLTWIAPTPQAVITKLK : 161
AM5        : NRDLFCEGEYYLHNTLKKKIEDVINTTN---GIIQREISC-TEDDSVVRM---SDRWGVNGEDFLTLDPKTLKWSSDSPLAAPVQSD : 157
AM6        : NMDLFCESEYYFHKTLKKKIEGVYNTTN---GIIQRERSC-TEDDSVVLM---SDSWGVNGEDFLTLDPKTLKWSSDTPLATPVQSG : 157
TR4        : --PGSCRPACYDVFEALKASSKFINHT----RDVQRRRVCITSDERLVSA---FDNWAVNGEGFIQFDAGAQKWKALSPSAEMIKDS :  89
TR5        : --PGTCRAACYDVFVALKEISQFINHT------------------------------------------------------------- :  78
TR6        : --PGTCRPACYDVFDALKEISKFINHT----RNVQRRRGCITSDERLESA---FDNWAVNGEGFIQFDAGAQKWKALSPSAEMIKDS : 147
TR9        : --PGTCEVALNDAFGALKAISKFINHT----RDVQRRRGCITSDERLESA---FDNWAVNGEGFIQFDAGAQKWKALSPSAEMIKDS : 147
TR10       : --PGTCRPACYDVFDALKAISKFTNHT----RDVQRRRGCITSDERLESA---FDNWAVNGEGFIQFDAGAQKWKALSPSAEMIKDS : 131
TR14       : --PGTCRPACYDVFDALKEISKFINHT----RNVQRRRGCITSDERLESA---FDNWAVNGEGFIQFDAGAQKWKALSPSAEMIKDS : 131
TR15       : --PGTCEVALNDAFGAIKEISKLINHT----LYVQRRRGCITSDERLESA---FDNWAVNGEGFIQFDAGAQKWKALSPSAEMIKDS : 147
TR16       : --PGTCRPACYDVFDALKAISKFTNHT----RNVQRRRGCITSDERLESA---FDNWAVNGEGFIQFDAGAQKWKVLSPSAEMIKDS : 147
TR17       : --PGTCTAAFYDVFDALKEISKFINHT----RNVQRRRGCITSDERLESA---FDNWAVNGEGFIQFDAGAQKWKALSPSAEMIKDS : 147
TR18       : --PGTCRPACYDVFDALKEISKFINHT----RNVQRRRGCITSDERLESA---FDNWAVNGEGFIQFDAGAQKWKALSPSAEMIKDS : 147
TR22       : --PGTCRPACYDVFDALKAISKFINHT----RDVQRRRGCITSDERLESA---FDNWAVNGEGFIQFDAGAQKWKALSPSAEMIKDS : 148
TR24       : --IGTCTAALNDGFDALKAISKFINHT----RNVQRRRGCITSDERLESA---FDNWAVNGEGFIQFDAGAQKWKALSPSAEMIKES : 131
TR25       : ---------------------------------VQRRRGCITSDERLESA---FDNWAVNGEGFIQFDAGAQKWKALSPSAEMIKDS :  51
TR26       : --PGTCEVALNDAFGALKEISKLINHT----LYVQRRRGCITSDERLESA---FDNWAVNGEGFIQFDAGAQKWKALSPSAEMIKDS : 131
TR27       : --PGTCEVALNDAFGALKAISKFINHT----RDVQRRRGCITSDERLESA---FDNWAVNGEGFIQFDAGAQKWKALSPSAEMIKDS : 131
TR28       : --PGTCTAAFYDVFDALKAISKFINHT----RNVQRRRGCITSDERLESA---FDNWAVNGEGFIQFDAGAQKWKALSPSAEMIKDS : 147
TR29       : --TGTCRPACYDVFDALKAISKFINHT----RNVQRRRGCITSDERLESA---FDNWAVNGEGFIQFDAGAQKWKVLSPSAEMIKDS : 131
TR30       : --PGSCRPACYDVFDALKAISKFINHT----RDVQRRRGCITSDERLESA---FDNWAVNGEGFIQFDAGAQKWKALSPSAEMIKDS : 147
TR31       : ------------------------------------------------------------NGEGFIQFDAGAQKWKVLSPSAEMIKDS :  28
TR32       : --PGTCEVALNDAFDALKEISKLINHT----LYVQRRRGCITSDERLESA---FDNWAVNGEGFIQFDAGAQKWKALSPSAEMIKDS : 147
TR34       : --PGTCRPACYDVFDALKEISKFINHT----RNVQRRRGCITSDERIESA---FDNWAVNGEGFIQFDAGAQKWKALSPSAEMIKDS : 121
TR_SRA     : --PGTCRPACYDVFDALKEISKFINHT----RNVQRRRGCITSDERLESA---FDNWAVNGEGFIQFDAGAQKWKALSPSAEMIKDS : 131
TN3        : --IRTCGNAQNDVFDALTEVSKFI--------DVQRRRGCTRSAEGLNSA---FEIWAVNGEDFIQFDADHQEWEALADTALPIRDS : 127
TN4        : --TRTCEEAQYDVYDALKEISKFIND-------VQRRRGCTRSAEGLNSA---FEIWAVNGEDFIQFDADHQEWEALADTALTIRDS : 128
TN7        : --IRTCEEAQYDVSDALREVSKFINSS----LDVQRRRGCTRSAEGLTSA---FEIWAVNGEDFIQFDADHQEWEALADTALTTRDS : 131
TN5        : --IRTCENAQYDVSDALREISKFINST----VDVQRRRGCTRSAEGLNSA---FEIWAVNGEDFIQFDADHQEWEALADTALTVRDS : 131
TN_SRA     : --IRTCEEAQYDVSDALTEISKFINSP----LYVQRRRGCTRSAEGLNSA---FEIWAVNGEDFIQFDADHQEWEALADTALTIRDS : 123
Cod_PAA    : ISLASCRQAHIDLKESHLKFQSVTNST---VDLIQRRRGCSRSENGALSA---FETWAVNGADFVTFDPKTLQWKAMSQAAREIASR : 160
Seabass    : --PKHCDEANNIIIDSVHVIPALINST---VYVIQRRCGCVRSADGIVSA---FDAWAVNGMDFISFDPESQRWTSQSPLAIPVRHW : 151
Sablefish  : ----GGSEACYDILESLYNIPPFVNGT---VSVVQRRRGCIQADNGMVSA---FEAWAVNGMDFMTFDPESQE-TSKSPSALTVKHR :  76
sasaPψ     : --------------------------------VLQRRRGC-TYITN-SST---GFGQGLNGENFLTFDPTSHTWMSESLQANPIEQS :  50
LO2        : YEHEEMENRERLQGILRTRSNMGKGQS-----ALRSVLGCRLGDDGSVAL---FDHYGFNGQDFLSFEVDTMTWRASSLQAEDTQRD : 146
LO3        : YEHEETENRERLQGILRKRSNMGKGQS-----AFRSVL-------------------------------------------------- : 112
LO4        : YELEETENRERLQGILRKRSNMGK-------DVLQRCRGCRLEDDGSVAL---FDHYGFNGKYFLSFEVDNMIWRASSLQAKDTQRD : 136
```

```
                *          160       *         180        *          200       *         220
                                                    Alpha 3 domain
             E        DD  A
             F     E  EE  DD  A   A   A
HLA-A2     : WEAA-HVAEQLRAYLEGTCVEWLRRYLENGKETLQRT-DAPKTHMTHHAVS-DHEATLRCWALSFYPAEITLTWQRDGEDQTQDT-E : 229
sasaUBA0301: WDSNTAQNEYRKNYLTQTCIEWLKKYLDYGKSTLMRT-VPPSVSLLQKTPS----SPVTCHATGFYPSGVMVSWQKDGQDHHEDV-E : 242
AM5        : WNQMKFMTPSLKDFKLNQCKPSLMKLKMKKEEYLQENMYPKMYIFGKSSHDG-DAVSMRCYISHKYLSGVRVRLTLDG--VAVDNVN : 241
AM6        : WNQMKFMTPSLKDFKMYQCKPSLMKLKKKKEEYLKGNLRPKIYIFGKPSHDG-DAVSLRCYVSHKYLSGVRVRLTLDG--VVVDNIN : 241
TR4        : WNNREARNHFFGHFINEEC-----QIKLREAD--KNT----DLRVFANPVDR-TKALLKCHVTSTDKSVRSVSLTEDG-APKANWIT : 163
TR5        : ------------------------------------------------RVDR-TKALLKCHVTSTDKSVRSVSLTEDG-APKANWIT : 115
TR6        : WNGYEAQNHVFGQFIRRYCPEMIHQIKLRETE--KRT----DLRVFANPVDL-TKALLKCHVTSTDKSVRSVSLTEDG-ATKANWIT : 226
TR9        : WNSRESRNDFFGQFINEECPEMIHQIKLREAE--KRT----DLRVFANPVDR-TKALLKCHVTSTDKSVRSVSLTEDG-APKANWIT : 226
TR10       : *NGYEARNHLFGQFIRRYCLEMIHQIKLREIE--KRT----DLRVFANPVDR-TKALLKCHVTSTDKSVRSVSLTEDG-APKANWIT : 209
TR11       : -----------------------------------------DLRVFANPVDR-TKALLKCHVTSTDKSVRSVSLTEDG-APKANWIT :  44
TR14       : WNGYEVQNYLFGQFIKEQCPEMIHQIKLREVE--KRT----DLRVFANPVDL-TRALLKCHVTSTDKSVRSVSLTEDG-ATKANWIT : 210
TR15       : WNGYEARNHLFGQFIRRYCLEMIHHIKLREIE--KRT----DLRVFANPVNR-TKALLKCHVTSTDKSVRSVSLTEDG-APKANWIT : 226
TR16       : WNNREARNHFFGQFIRRYCLEMIHQIKLKETE--KRT----DLRVFANPVDR-TKALLKCHVTSTDKSVRSVSLTEDG-APKANWIT : 226
TR17       : WNGYEARNHFFGHFINEECPKMIHQIKLREVE--KRT-------------------------------------------------- : 182
TR18       : WNGYEPRKHVFGQFIRRYCLEMIHQIKLREIE--KRT----DLRVFAHPVDR-TKALLKCHVTSTDKSVRSVSLTEDG-APKANWIT : 226
TR22       : WNGYEARNHLFGQFIRRHCLEMIHHIKLREIE--KRT----DLCVFANPVDR-TKALLKCHVTSTDKSVRSVSLTEDG-APKANWIT : 227
TR24       : WNGYEAQNHFFGQFINEECPEMIHHIKLREAE--KRT-------------------------------------------------- : 166
TR25       : WNGYEPRKHVFGQFIRRYCLEMIHQIKLREIE--KRT----DLRVFANPVDR-TKALLKCHVTSTDKSVRSVSLTEDG-ASKANWIT : 130
TR26       : WNNREARNHFFGHFINEECPEMIHQIKLREVG--KKN----DLRVFANPVDR-TKALLKCHVTSTDKSVRSVSLTEDG-APKANWIT : 210
TR27       : WNSRESRNDFFGQFINEECPEMIHQIKLREAE--KRT-------------------------------------------------- : 166
TR28       : WNSYETRNHLFGQFIRRYCLEMIHHIKLREIE--QRT-------------------------------------------------- : 182
TR29       : WNSYEARNHLFGQFIRRYCLEMIHQIKLKETE--KRT----DLRVFANPVNR-TKALLKCHVTSTDKSVRSVSLTEDG-APKANWIT : 210
TR30       : WNGYEARNHLF---------------------------------------------------------------------------- : 158
TR31       : WNNREARNHFYGHFISKQCPEMIHQIKLKEIE--KRT----DLRVFANPVDR-TKALLKCHVTSTDKSVRSVSLTEDG-APKANWIT : 107
TR32       : WNGCEARNHFFGQFINEECPEMIHQIKLREVE--KRT-------------------------------------------------- : 182
TR33       : -----------------------------------------DLRVFANPVNR-TKALLKCHVTSTDKSVRSVSLTEDG-APKANWIT :  44
TR34       : WNGYETRNHLFGQFIRRYCPEMIHQIKLRETE--KRT----DLRVFANPVDR-TKALLKCHVTSTDKSVRSVSLTEDG-APKANWIT : 200
TR_SRA     : WNGYEARKHVFGQFIRRYCPEMIHQIKLKETE--KRT----DLRVFANPVDL-TRALLKCHVTSTDKSVRSVSLTEDG-ATKANWIT : 210
TN3        : WNNRETRNHVFGSFLKDQCPEMIQRIKLREVE--QRT----DLRVFATPIDR-SQTLLKCHVTSTDKSLRSLSLTEDG-ASRALWVA : 206
TN4        : WNNQEIRNLVSRSFLKDQCPEMIQRIKLREVE--QKT-------------------------------------------------- : 163
TN5        : WNNLETRNQVFGSFLKDQCPEMIQRINLTEVE--QRT----DLRVFATPIDR-FQTLLKCHVTSTDKSLGSLSLTEDG-ASRALWVA : 210
TN7        : WNNQETRNQVFGSFLKDQCPQMIQRTSLTEVE--QRT-------------------------------------------------- : 166
TN_SRA     : WNNLETRNHVFGSFLKVQCPEMIQRIKLREVE--QKT----DLRVFATPIDR-FQTLLKCHVTSTDKSLRSLSLTEDG-ASRALWVA : 202
Cod_PAA    : WNTRKTRNLVFRDFVNIHCPKMIKSLELKYVD--QKT----DLHISAQVLPNESKFKLRCQVTSTDRSVKLLTLIGEG-ASYARQVT : 240
Seabass    : WKNNNGRNFAFKHFLRERCPLLIQGMKLRSTH--QNT----ELHIFAKPIADTDQALLRCHVTSTDKSVSSVHLIGDG-AFKANWIS : 231
Sablefish  : WNNNRQRNHAFRHFIIEQCPLLIQKIKLRSMH--QKT----ELRIFAKPEKNTARFLLRCHVTSTDTSLSSVHLIGDG-ASRASWIT : 156
sasaPψ     : WNSNKVRSHMFKEFLQHDCPH--------------------------------EDVSLRCHVTSTDLSGLKVHLTRDR-GVMTDRAR : 104
LO2        : WNQNRVKNQYIRAFLEIDCMETLKRFLEFRKIDKNHTG--ETEKTPSAVL------------------------------------- : 194
LO4        : WNQNRVKNQYTRFFLEIDCMETLKRFLEFREIDKNHT-------------------------------------------------- : 173
```

```
             *         240       *            260       *         280                  *         300
                                                            CP                TM
HLA-A2     : LVETRPAGDGTFQKWAAVVVPSGQEQ---RYTCHVQHEGLPKPLTLRWEPSSQPT-----------IPIVGIIAGLVLFGAVITGAV : 302
sasaUBA0301: HGETLQNDDGTFQKSSHLTVT-PEEWKNNKYQCVVQVTGLQEDFIKVLTESEIKTNWNDPN-------IVLIIGVVVALLLVVVAVV : 321
-----------------------------------------------------------------------------------------------------
AM5        : ISSPAPNMDGSVQIRLETKTNI--KEPNR-YHCVVDTD--YLHIFTAW--------------------------------------- : 284
AM6        : ISSPAPNMDGSVQIRL----------------------------------------------------------------------- : 257
TR4        : VTGPLPSGDGSVILILTAEVP---LIHTNIYGCVVQTE--DRNITVMW--------------------------------------- : 206
TR5        : VTGPLPSGDGSVILILTAEVP---LIHTNIYGCVVQTE--DRTITVMWDGNTLDGRH-------ILYIHMTFWRIIGIIFTVCCLIS : 190
TR6        : VTGPLPSGDGSVILILTAEVP---LIHTNIYGCVVQTE--DRNITVMWDGNTLDGRH------ILYAGVPMKFWIIIGIIFVCCLIS : 302
TR9        : VTGPLPSGDGSVILILTAEVP---LIHTNIYGCVVQTE--DRNITVMW--------------------------------------- : 269
TR10       : VTGPLPSGDGSVILILTAEVP---LIHTNIYGCVVQTE--DRTITVMWDGNTLDGRE-----ILSTSVSWTQILGCVLFLCVLFLIC : 286
TR11       : VTGPLPSGDGSVILILTAEVP---LIHTNIYGCVVQTE--ERNITVMW--------------------------------------- :  87
TR14       : VTGPLPSGDGSVILILTAEVP---LIHTNIYGCVVQTE--DRDITVMWDGNTLDGR---EILYATVDWPFWKILTIVTFSCVFLLIC : 289
TR15       : VTGPLPSGDGSVILILTAEVP---LIHTNIYGCVV---------------------------------------------------- : 258
TR16       : VTGPLPSGDGSVILILTAEVP---LIHTNIYGCVVQTE--DRT-------------------------------------------- : 264
TR18       : VTGPLPSGDGSVILILTAEVP---LIHTNIYGCVVQTE--DRNITVMW--------------------------------------- : 269
TR22       : VTGPLPSGDGSVILILTAEVP---LIHTNIYGCVVQTE--DRTITVMW--------------------------------------- : 270
TR25       : VTGPLPSGDGSVILILTAEVP------------------------------------------------------------------ : 151
TR26       : VTGPLPSGDGSVILILTAEVP---LIHTNIYGCVVQTE--DRNITVMW--------------------------------------- : 253
TR29       : VTGPLPSGDGSVILILTAEVP---LIHTNIYGCVVQTE--DRNITVMWDGKTLDGRE---ILYATVVWPLWKILTIVTFSCVCLLIC : 289
TR31       : VTGPLPSGDGSVILILTAEVP---LIHTNIYGCVVQTE--DRTITVMWDGNTLDGRH-------ILYIHMTFWRIIGIIFTVCCLIS : 182
TR33       : VTGPLPSGDGSVILILTAEVP---LIHTNIYGCVVQTE--DRNITVMWDGNTLDGRH-------ILYIQMTFWRIIGNIFTVCCLIS : 119
TR34       : VTGPLPSGDGSVILILTAEVP---LIHTNIYGCVVQTE--DRNITVMW--------------------------------------- : 243
TR_SRA     : VTGPLPSGDGSVILILTAEVP---LIHTNIYGCVVQTE--DRNITVMWDGNTLDGRE---ILYATVDWPFWKTLTIVTFSCVFLLIC : 289
TN3        : VRGPLPSVDGSVVLRLTAEVP---LGYSNIYGCVVQTG--GGTITVMW--------------------------------------- : 249
TN5        : VRGPLPSVDGSVVLRLTAEVP---LGYSNIYGCVVPTG--GGTITVMWDGKTLDGRD--IFYASIHWISLCILVFIVFALVLLVCAI : 290
TN_SRA     : VRGPLPSVDGSVVLRLTAEVP---LGYSNIYGCVVQT-------------------------------------------------- : 236
Cod_PAA    : VDGPLPFGDDYVILRLTAVIP---SSEHQ-YGCRVQTE--KHSFSAFW--------------------------------------- : 282
Seabass    : VTGPMPSEDGSVILRLTAEIS--LSQSTNMYGCRVQTG--GHNITIFWDGNTLDGRNLLNMLTVHWKILTAILGFVCIIFVITAISC : 314
Sablefish  : VDGPMPSEDGSVILRLTAEIS--QSRNTNTYGCRVQTG--GHNTTVFWDG--------------------------------------- : 202
sasaPψ     : VIGPLPNVDGSVLLRLSVEIP--TGHTKSRYHCKVQTS--TSNTAA------------------------------------------- : 146
```

```
                     *          320         *          340
                    CYT
HLA-A2      : VAAVMWRRKSSDRKGGSYSQAASSDSAQGSDVSLTACKV- : 341
sasaUBA0301: VGVVIWKKKSKKGFVPASTSDTDSDNSGRAAQMT------ : 355
TR5         : VMTLLCKS-------------------------------- : 198
TR6         : VMTLLCKS-------------------------------- : 310
TR10        : TTALVLK--------------------------------- : 293
TR14        : TTAVVLK--------------------------------- : 296
TR29        : TTALVLKHNEKKIKRRMKKR-------------------- : 309
TR31        : VMTLLCKSHFFDCNFQVSL--------------------- : 201
TR33        : VMTLLCKSHFFDCNFQLSL--------------------- : 138
TR_SRA      : TTAVVLKYNEKKIKRRMKKRIPGSKT-------------- : 315
TN5         : QRWCCFKVSL------------------------------ : 300
Seabass     : GTIFLLKCVKKKSRPP------------------------: 330
```

Text S7b. Percent identity per domain between deduced P lineage amino acid sequences

Alpha 1 domain

	1	2	3	4	5	6	7	8	9	10	11	12	13	14	15	16	17	18	19
1: TN3 (P)	100	87	69	69	71	68	68	67	67	67	49	26	28	26	17	13	18	18	13
2: TN5 (P)	87	100	76	76	77	74	74	73	72	72	51	28	35	33	19	13	19	19	16
3: TR6 (P)	69	76	100	100	99	99	97	95	91	91	56	32	32	31	19	15	18	21	16
4: TR14 (P)	69	76	100	100	99	99	97	95	91	91	56	32	32	31	19	15	18	21	16
5: TR18 (P)	71	77	99	99	100	97	96	94	90	90	55	32	32	31	19	15	18	21	16
6: TR22 (P)	68	74	99	99	97	100	99	96	92	90	56	32	32	31	19	15	18	21	16
7: TR29 (P)	68	74	97	97	96	99	100	95	91	88	55	32	32	31	21	17	19	21	16
8: TR16 (P)	67	73	95	95	94	96	95	100	88	86	54	33	31	31	18	14	17	19	14
9: TR9 (P)	67	72	91	91	90	92	91	88	100	97	56	31	32	31	19	15	18	22	16
10: TR26 (P)	67	72	91	91	90	90	88	86	97	100	58	31	32	31	19	15	18	22	16
11: Seabass (P)	49	51	56	56	55	56	55	54	56	58	100	37	33	31	23	17	14	19	20
12: codPAA	26	28	32	32	32	32	32	33	31	31	37	100	20	21	15	12	11	17	16
13: AM5 (P)	28	35	32	32	32	32	32	31	32	32	33	20	100	88	27	18	20	21	11
14: AM6 (P)	26	33	31	31	31	31	31	31	31	31	31	21	88	100	26	19	21	22	11
15: sasaUBA0301	17	19	19	19	19	19	21	18	19	19	23	15	27	26	100	44	29	26	20
16: sasaZAAa	13	13	15	15	15	15	17	14	15	15	17	12	18	19	44	100	25	23	14
17: HLA-A2	18	19	18	18	18	18	19	17	18	18	14	11	20	21	29	25	100	21	19
18: sasaLCA	18	19	21	21	21	21	21	19	22	22	19	17	21	22	26	23	21	100	20
19: sasaSAA	13	16	16	16	16	16	16	14	16	16	20	16	11	11	20	14	19	20	100

Alpha 2 domain

	1	2	3	4	5	6	7	8	9	10	11	12	13	14	15	16	17	18	19	20
1: sasaUBA0301	100	43	40	23	24	23	25	24	25	26	25	25	25	22	22	22	27	28	33	26
2: sasaZAAa	43	100	29	18	20	16	17	16	16	17	17	18	18	21	19	18	19	19	26	16
3: HLA-A2	40	29	100	17	18	18	20	17	18	18	21	18	17	22	24	16	18	16	28	20
4: Sablefish(P)	23	18	17	100	65	46	47	47	46	47	48	48	52	51	49	47	30	30	20	19
5: Seabass (P)	24	20	18	65	100	40	41	42	41	42	41	42	43	49	47	42	32	31	20	17
6: TR18 (P)	23	16	18	46	40	100	93	91	92	94	89	86	82	62	57	40	26	26	17	19
7: TR22 (P)	25	17	20	47	41	93	100	90	92	92	89	88	83	63	58	41	26	26	17	19
8: TR16 (P)	24	16	17	47	42	91	90	100	97	92	85	88	85	62	57	41	28	28	16	16
9: TR29 (P)	25	16	18	46	41	92	92	97	100	93	88	86	82	60	56	40	28	28	16	17
10: TR6 (P)	26	17	18	47	42	94	92	92	93	100	92	88	84	63	58	41	26	26	17	18
11: TR14 (P)	25	17	21	48	41	89	89	85	88	92	100	89	84	64	60	41	28	28	18	19
12: TR9 (P)	25	18	18	48	42	86	88	88	86	88	89	100	89	64	59	43	26	26	17	18
13: TR26 (P)	25	18	17	52	43	82	83	85	82	84	84	89	100	63	57	44	25	25	17	18
14: TN3 (P)	22	21	22	51	49	62	63	62	60	63	64	64	63	100	93	49	26	27	20	21
15: TN5 (P)	22	19	24	49	47	57	58	57	56	58	60	59	57	93	100	47	24	25	19	19
16: codPAA	22	18	16	47	42	40	41	41	40	41	41	43	44	49	47	100	32	32	24	17
17: AM5 (P)	27	19	18	30	32	26	26	28	28	26	28	26	25	26	24	32	100	87	17	11
18: AM6 (P)	28	19	16	30	31	26	26	28	28	26	28	26	25	27	25	32	87	100	18	12
19: sasaSAA	33	26	28	20	20	17	17	16	16	17	18	17	17	20	19	24	17	18	100	14
20: sasaLCA	26	16	20	19	17	19	19	16	17	18	19	18	18	21	19	17	11	12	14	100

Alpha 3 domain

	1	2	3	4	5	6	7	8	9	10	11	12	13	14	15	16	17	18	19
1: Sablefish(P)	100	78	60	60	61	60	59	57	60	60	60	59	54	28	19	25	23	23	19
2: Seabass (P)	78	100	67	67	66	67	66	66	67	66	60	59	51	32	19	21	21	24	23
3: TR9 (P)	60	67	100	100	99	99	99	98	98	95	74	71	47	29	22	26	20	26	24
4: TR26 (P)	60	67	100	100	99	99	99	98	98	95	74	71	47	29	22	26	20	26	24
5: TR29 (P)	61	66	99	99	100	98	98	97	97	94	72	70	48	28	22	26	20	26	23
6: TR18 (P)	60	67	99	99	98	100	98	97	97	94	74	71	47	29	22	26	20	26	25
7: TR16 (P)	59	66	99	99	98	98	100	99	96	95	73	71	48	28	23	25	18	24	23
8: TR22 (P)	57	66	98	98	97	97	99	100	95	94	74	71	47	29	22	25	20	26	24
9: TR6 (P)	60	67	98	98	97	97	96	95	100	98	72	70	47	29	22	25	20	26	24
10: TR14 (P)	60	66	95	95	94	94	95	94	98	100	72	70	45	29	22	26	20	26	24
11: TN3 (P)	60	60	74	74	72	74	73	74	72	72	100	97	45	29	23	25	21	27	24
12: TN5 (P)	59	59	71	71	70	71	71	71	70	70	97	100	43	29	22	25	21	26	23
13: codPAA	54	51	47	47	48	47	48	47	47	45	45	43	100	22	16	22	21	20	24
14: AM5 (P)	28	32	29	29	28	29	28	29	29	29	29	29	22	100	26	21	28	24	20
15: sasaUBA0301	19	19	22	22	22	22	23	22	22	22	23	22	16	26	100	33	31	36	33
16: sasaLCA	25	21	26	26	26	26	25	25	25	26	25	25	22	21	33	100	25	38	32
17: sasaZAAa	23	21	20	20	20	20	18	20	20	20	21	21	21	28	31	25	100	38	25
18: sasaSAA	23	24	26	26	26	26	24	26	26	26	27	26	20	24	36	38	38	100	24
19: HLA-A2	19	23	24	24	23	25	23	24	24	24	24	23	24	20	33	32	25	24	100

Percentage identity between domains based on amino acids and calculated using ClustalX2.0.11. See legend to Text S7a for sequence details.

Text S7c. Exon intron structure of P lineage genes

Schematic exon intron structure of P lineage genes. Exon sizes in base pairs are shown above the gene while intron sizes are shown below.

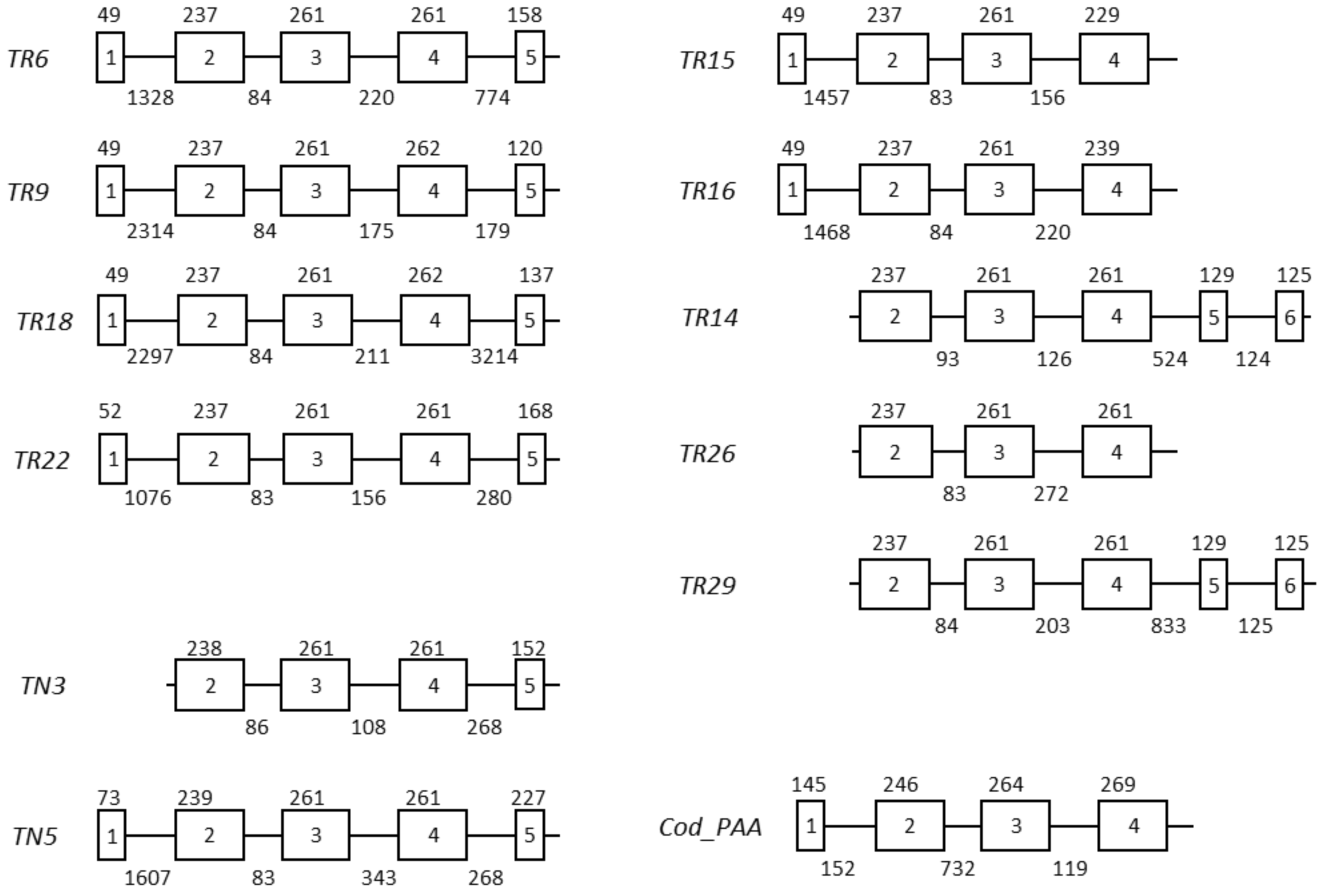

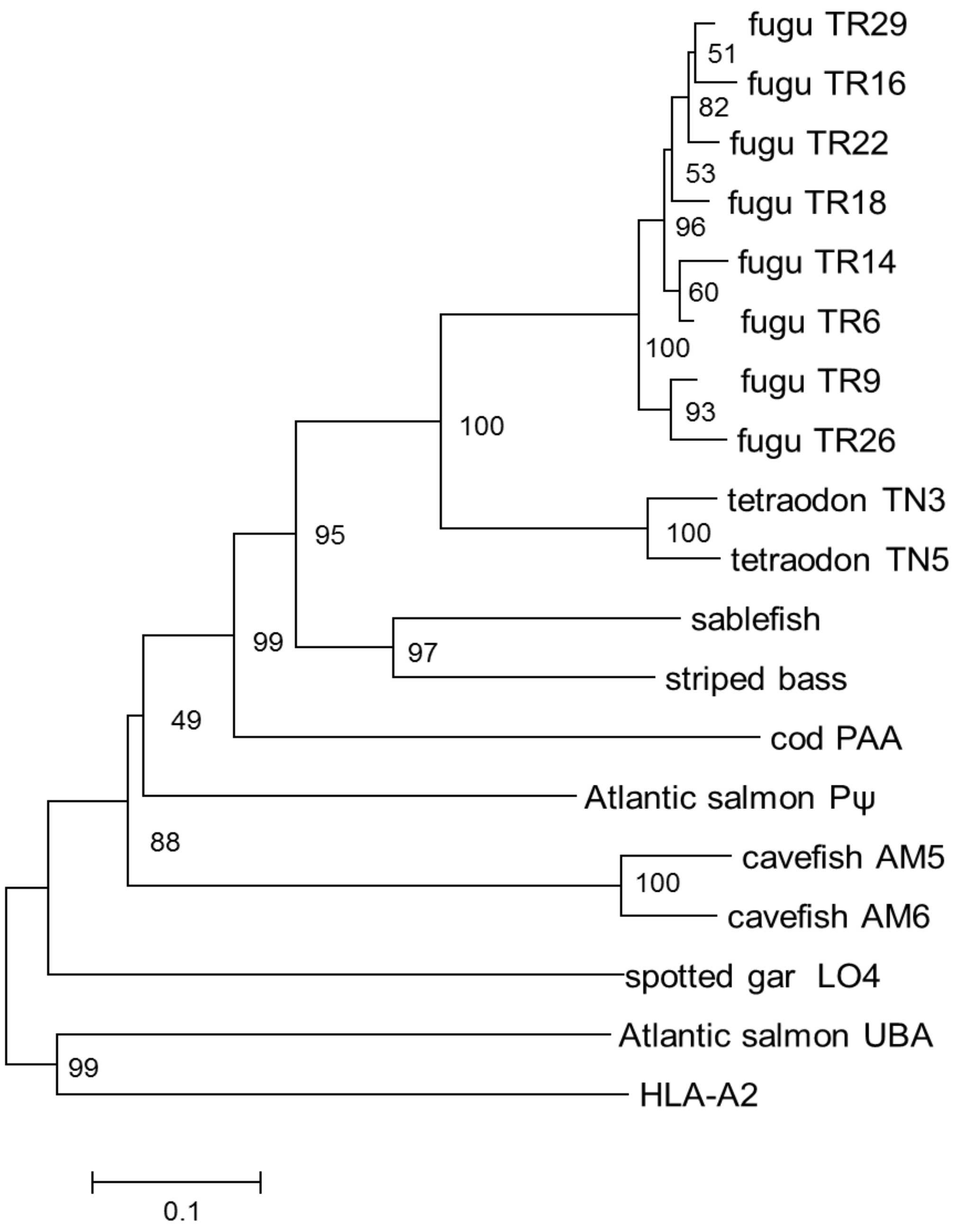

Text S7d. Phylogenetic tree of deduced P lineage amino acid sequences

Phylogenetic tree based on alpha 1 through alpha 3 domain amino acid sequences. The evolutionary history was inferred using the Neighbor-Joining method [main text reference 95]. The percentages of replicate trees in which the associated taxa clustered together in the bootstrap test (1000 replicates) are shown next to the branches [96]. The tree is drawn to scale, with branch lengths in the same units as those of the evolutionary distances used to infer the phylogenetic tree. The evolutionary distances were computed using the p-distance method [main text reference 97] and are in the units of the number of amino acid differences per site. All ambiguous positions were removed for each sequence pair. Evolutionary analyses were conducted in MEGA5 [main text reference 98].

Sequence references can be found in either additional files 3: Text S1 or additional text 4: Text S2. Cavefish is *Astyanax mexicanus*, tetraodon is *Tetraodon nigriviridis*, fugu is *Takifugu rubripes*,spotted gar is *Lepisosteus oculatus*, Atlantic salmon is *Salmo salar*,sablefish is *Anoplopoma fimbria* and striped bass is *Dicentrarchus labrax*. The tree is rooted using the salmon *UBA*0301* allele (GenBank accession AAN75116.1) and the human HLA-A2 sequences (GenBank accession AAA76608.2).
