# Supplementary material for: A comprehensive analysis of teleost MHC class I sequences
Source: BMC Evol Biol. 2015 Mar 6;15:32. doi: 10.1186/s12862-015-0309-1 (PMC4364491; doi:10.1186/s12862-015-0309-1)
Supplement: Additional file 11: — Text S8. Comparison of all lineages. [file 12862_2015_309_MOESM11_ESM.pdf]

Additional file 11: Text S8. Comparison of all lineages

Table of contents		Page
Text S8a	Alignment of representative deduced MHC class I amino acid sequences from all five lineages	2
Text S8b	Phylogenetic tree of deduced alpha 2 amino acid domain sequences	6
Text S8c	Phylogenetic tree of deduced alpha 3 amino acid domain sequences	7
Text S8d	Phylogenetic distribution of MHCI lineages	8

Text S8a. Alignment of representative MHC class I amino acid sequences from all five lineages

Alignment of selected MHC class I amino acid sequences from ray-finned fishes aligned against sequences from chondrichthyes (elephant and dogfish shark) and human using the human HLA-A2 sequence as reference. The leader sequences are not shown. Numbering on top is according to the mature HLA-A2 sequence. Letters A through F above the alignment represent HLA-A2 residues known to constitute the peptide binding groove pockets A through F [main text references 1 and 3] while T are HLA-A2 residues known to interact with CD8 [Gao et al., Crystal structure of the complex between human CD8alpha(alpha) and HLA-A2. Nature 1997, 387(6633):630-634]. Residues conserved between depicted human, chondrichthian and some ray-finned sequences are shaded grey, while semi-conserved residues are shaded green. Cysteines are shaded cyan blue, unique lineage motifs are shaded purple and potential tyrosine-based endosomal sorting motifs in the cytoplasmic domain are shaded yellow. N-linked glycosylation motifs are underlined. Lineages are separated by a black line and the lineage representatives used for aligning the connecting peptide (CP), transmembrane (TM) and cytoplasmic (CYT) domains are selected based on expressed support. Where there is no expressed support, the cytoplasmic domain is shown with a question mark. Transmembrane regions are predicted using RHYTM (http://proteinformatics.charite.de/rhythm/index.php?site=home). Sequence names mostly reflect the species Latin name where sasa is Atlantic salmon (*Salmo salar*), AM is cavefish (*Astyanax mexicanus*), DR is zebrafish (*Danio rerio*), ON is nile tilapia (*Oreochromis niloticus*), GA is stickleback (*Gasterosteus aculeatus*), TN is tetraodon (*Tetraodon nigroviridis*), TR is fugu (*Takifugu rubripes*), OL is medaka (*Oryzias latipes*), LO is spotted gar (*Lepisosteus oculatus*), eel is *Anguilla japonica*, ayu is *Plecoglossus altivelis* and seabass is *Dicentrarcus labrax*. GenBank accession numbers not provided in additional files 3: Text S1 and 4: Text S2 are: catfish (*Ictalurus punctatus*) S lineage sequence is JT320438, Ayu S lineage is JP747954, cod PAA (*Gadus morhua*) P lineage is GW844691, paddlefish (*Polyodon spatula*) UBA*01 is ACV87421.1 and UBA*03 is ACV87423.1, sturgeon (*Acipenser sinensis*) U lineage is ACV87437.1, lungfish (*Protopterus aethiopicus*) is AAF15304.1, the shark spiny dogfish (*Squalus acanthias*) is AAN77874.1, the chimaera elephant shark (*Callorhinchus milii*) *is* AFM85876.1, and the human sequence HLA-G is EAX03236.1, HLA-A2 is AAA76608.2 and HLA-B27 is CAA83876.1.

Alpha 1 domain

```
                      1        *          20           *         40         *           60         *         80            *
                            A B                                                                    A  A   B  C
                          A B C                 C B         B          B                 A   B  BB  C  FC  F  FF  F
HLA-A2              --GSHSMRYFFTSVSRPGRG-EP---RFIAVGYVDDTQFVRFDSDAASQRMEPRAPWIEQ---EGPEYWDGETRKVKAHSQTHRVDLGTLRGYY---NQSEA-
HLA-G               --GSHSMRYFSAAVSRPGRG-EP---RFIAMGYVDDTQFVRFDSDSACPRMEPRAPWVEQ---EGPEYWEEETRNTKAHAQTDRMNLQTLRGYY---NQSEA-
HLA-B27             --GSHSMRYFHTSVSRPGRG-EP---RFITVGYVDDTLFVRFDSDAASPREEPRAPWIEQ---EGPEYWDRETQICKAKAQTDREDLRTLLRYY---NQSEA-
Elephant shark      --GSHSLRYFYTAVH-GAPG-VP---EFSIVGYVDDQQFVRYQSNSESRRMEPRQRWIQE--REDAGYWDGQTRTAQGWEQTYKVNVKTVMGRY---NQTG--
Spiny Dogfish       --GTHSLRYFYNSMT-PIPG-VP---EFVAVGYVDDALFVHYDSD--RKQMIPRQRWIEE--SEDKQYWERETQKQLGWEQIGKVDIQTLITRT---NLTG--
------------------------------------------------------------------------------------------------------------------------------
sasaUBA (U)         --ATHSLRYVYTATS-GIPD-FP---EFVTVGLVNGEPISYYDSI--IRRETPRQDWMAK--TEGSDYWESQTQVSIGSEQTFKANIDVAKQRF---NQTG--
DR14 (UBA) (U)      --ATHSWKAYYTGTT-GLTE-FP---EFVALNLIDDQLMGYFDSK--TNRFKSQFQWMED--NLGKEYDEQQTNILLGYPEVFKNNIKVVMERF---NQTQ--
OL10 (UAA) (U)      --VTHSLKYFYTASS-QVPN-FP---EFVSVGLVDDAPISHYDSD--TRMTIIKQDWMKD--AMDEQYLERNTANFLGSQQVYKANIEVAKQRF---NQTG--
OL11 (UBA) (U)      --VTHSLKYFYTASS-QVPT-LP---EFVAVGLVDDAQIDYYDSD--IRMVVPKQDWMKE--AMDGQYWKTETENRLGDQHTFKARIEILKQSF---NQTG--
------------------------------------------------------------------------------------------------------------------------------
ON39 (U)            --VTHSLKYFLTGSS-QVPN-FP---EFVVVGMVDDVQIDYYDSN--TEKAVPKQDWIAR--NTDQQYWERETANCWGSQQSFKANIDTAKQRF---NQTG--
GA9  (U)            --VTHSLKYFYTGSS-GLPN-FP---EFVIVGLVDEVEVVHYDSD--TWRLEPRQDWVSRVTEDDHWFWNWQTGLAMNAQREFKGYIKTAKQRF---NQTGA-
TR13 (U)            --VTHTLKYFYTASS-GVPN-FP---EFVAFGMVDDVQMVRYDSN--TGRLQFKQEWMKEATADDPQFWDKKTGILMGAQQWFKVNIEILKQRF---NQTGAV
LO5  (U)            --VTHSLRYFYTGVT-GVRG-FP---EFTIVGLVDGQEFVHYDSD--IKRMIPKTEWIER--NEGKDYWDRQTQILIGASQVFKTDLVNLPQRF---NQSA--
Paddlefish*01 (U)   --GSHSLRYFYTGTS-GVTE-FP---EFVIVGMVDDVQISHCDSK--SKQTVPKQQWMKD--NVEPGYWERNTQICLGNQQIFKVAVIDLPKRF---NQTE--
Paddlefish*03 (U)   --GTHSLRYFFTGVT-AGTG-LP---EFVTVGLVDDEQHVHYDSV--SKKAVARQDWMAK--SEGPEYWESETQNFAGEEQVFKVNIGTLMQRF---NQTG--
Sturgeon (U)        GTRTHSLRYFYTGTS-GMTE-FP---EFVAVGMVDDVQIDYYDSK--SKKDISKQQWMKD--NMEPAYWEGNTQKCLGHQQNFKANIGIAMQRF---NQTG--
------------------------------------------------------------------------------------------------------------------------------
sasaZAAa (Z)        --DTYSLSYIYTALS-KPVD-LPGIHEFTAMGLMNNQQIDYYDSV--SKKKIPKQDWMRE--KLPADYWEKGTQSRKSKEQWFKVNVDILMKRMRH-NNT---
AM8 (Z1)            --ERHSLYYIYSALN-KDVS-LPGIYEFTALGLLDGREIDYYNSK--EQKKIPKQSWMME--KMQEDYWEKGTQSRKSKEQWFKVSLETVMQRM---NHSNT-
AM3 (Z2)            --EKLSLNYCYTMQY-T-NS-ESNLYDCTVVTLLNDTQIYFYNSA--DPNKTPKPHWLK---ISETD-WIKSTTWSDGDRQWMNKVINIQSSV----NKS---
AM25 (Z2)           ---EHLLEYLYTMQS-K-N---CTIYDCTAVTLLNDRQIDFYNSSS-DKPRTAKQNWMKN--ISESD-WKTITEKLQSDRSLLNKLLDIQMDESGH-SNS---
DR1 (ZEA)           --EKHSLYYIYTALS-RPVH-LPGIYEFTAMGLLDDRQIDYYNSQ--EQKKIPKQHWMKE--KMQEDYWEKGTQSRKSKEQWFNVNLKILMERMRHNNNS---
OL4  (Z)            --EKHSLTYIYTAFS-HPVK-LPGIHEFTAMGLLDNRMIDYYDSS--VQKKIPKQDWMKE--RLQQEYWDKGTQSRQSKQQWFKVNIDILINRMRQ-TSN---
GA18 (Z)            --VKHSLTYTYTAFAHKPVG-LPGIHEFTAMGHLDTRMIDYFDSD--QQLKVPKQPWMGE--RLDKDYWVKGTQSRQSKQQWFKVNIGILMNRLRQ-NKTS--
ON22 (Z)            --EKHSLHYIYTGLS-KPVG-LPGIHEFTAMGLLDGRMIDYYDSE--NQTKVPKQEWMRE--HLPADYWVKGTQSRRIKQRWFKHNIGILMERMRK-NDSG--
TN13 (Z)            --EIHSLHYIYTALS-RPVN-RPGIHEFTAMGMLDDHMIDYFDSD--KQVKVPKQTWMKT--ELEENYWEKGTQSRKSKQQWFKVNIKILKDRFRQ-NDS---
TR21 (Z)            --EKHSLHYIYTATS-QPIS-RQGIHEFTAMGVLDGNVIDYFDSD--AQKKVPRQKWMEK--ELDKSYWEKGTESRKSKQQWFKVNINILKTRYRQ-NDS---
Eel  (Z)            --EIHNLHYIYTALS-KPIT-QPGVYQFTALGILNDRPIDVYNSK--DKVKKPLQKWMEE--HNEKDYWEKGTQSRRSKEQWFDVNVKILMERMRQ-NQSDP-
Gar LO14            --ERHSLHYIYTALS-KPVDGIP---EFTAMGVLNDRRIDYYDNW--IMQKIPKQSWMKT--NMPQEYWEKGTQSRKSKEQWFKVNVGILMDRMRQ-NNS---
Sturgeon (Z)        ----------------CREG-FP---EFTAMGLLDDMQIDYYDSV--NQKKESKQTWMKE--NMAPDYWEKGTKSRKSKEQWFKVNVRILEKRF---------
Lungfish (Z)        --SFHSLRYTYSAYA-SNDK-LV---EFVAQGLLDDVQIDYYDNH--IRREVPKQQWMNE--SMEAGYWERGTQSRNSKEHWFTVNTQIVMQRR---NDTS--
------------------------------------------------------------------------------------------------------------------------------
AM33 (S)            --EVNSITAYFIGIQ-GLN--LP---DYMERIAVNDVTMFYYDSS--MKDEVSCPDWLNT--SSGKQHWMDINLISLHNKHSMATALKSAILQF---NQT---
AM39 (S)            --LRYDSCAYYMVSK-GLG--LP---EFSERRVLNDVTVYHHDSS--LDSKMPCPDWINT--TAGKEHWKIIYHWTDYNKYVGTLGLQSATEQF---NQT---
sasaSAA (S)         --APHSLHRHCIATQ-GTL--YP---KNIQLVMIDDVTVYYYNSS--AEQEAVVPEWLNH--PEGIEFWQEVNRNLKFNRFVMDTAVRVTSEHY---NHS---
Ayu  (S)            --EPHSLHRHCIATQ-GIS--YH---KHIQFVMIDDHIIYYYNSS--SDLGSPMPEWLDH--SEGSEFWKEFTRNLKYNRHVMEKAVQLTSEHF---NHS---
Catfish (S)         --DVQSFSLYLLRSE-GLS--LP---RYTQSVTVNDVTLYSFDSN--MKSTGPCPEWLNT--TAGQQLWKEASFLFHRNMANMDLALQTAKSQF---NLT---
------------------------------------------------------------------------------------------------------------------------------
sasaLCA (L)         GSGSHSLWALATYIS-GETP-FP---EFTVVVMLDDVQVAYYDSN--MKHFIYRGHNTPN--KIHDDEAKNGDFVFGVMYHHMKERYFHLKHHL---NLTE--
sasaLGA (L)         GSGSHSLWALATYIV-GETP-FP---EFTVVVMLDDVQVAYYDSN--DKQSVYRGQHIT---KTKDDEAQDGAHVFRVIYQSMKDRSFELKHRF---NLTE--
DR12 (L)            --GSHSLMALATYIV-GQTP-FP---EFSVVVMLDDLQLAYYDSI--GWKTIYRSGSDS---KYYDEEQSDAGIVFRDMFYDMKDRAFYLKDHQ---NHTD--
ON9  (L)            GTGRHSLWALASYIP-GSAH-FP---EFTVVLMLDDIQVGYYDSK--VNQVMRTSTASDH--KAELNLGQEPVNVLRDIYSSMRKRLNLVKHRF---NLTID-
LO11 (L)            --GTHSMSWSFTLTQ-EAQW-IP---KFAVVGYLDGLPMEYYDST--HERVVSRRHWRPD--PAPAENEEEKKGAAEHTYISMFEKLQLAKKHF---NHSG--
------------------------------------------------------------------------------------------------------------------------------
AM5  (P)            --EKHSLQYLYTLRS-APED-DT---EFEITTVFDGLIISHCKSP--RFRDHSREDWISQ--TFTNAEWKNRDLFCEGEYYLHNTLKKKIEDVI---NTTN--
TN5  (P)            --GSHSLDFLSTGRF-HPGT-GL---YFEQVTVFDGVLISHCDSR--TQQEQFKAVLESH--NL--------IRTCENAQYDVSDALREISKFI---NST---
TR9  (P)            --GSHSLEFLSTGRV-QPGS-GP---HFEQVTEFDGVVISHCDSG--TQQEHFKPVLESH--NL--------PGTCEVALNDAFGALKAISKFI---NHT---
TR14 (P)            --GSHSLEFLSTGRV-QPGS-GP---HFEQVTEFDGVVISHCDSG--TQQEHFKPVLESH--NL--------PGTCRPACYDVFDALKEISKFI---NHT---
CodPAA (P)          --DPHSLEFTSMFGP-ESKF-RP---TFQQLVRFDGVPLSHCESW--SEKALPELRPSLQ--DV-----QISLASCRQAHIDLKESHLKFQSVT---NST---
Seabass(P)          --GSHSLEFLSTGRV-QPED-QP---QFEQLTVFDGVPISYCNSW--KKREELKPTLESN--NL--------PKHCDEANNIIIDSVHVIPALI---NST---
LO4  (P)            --ASHSLVYTSTAVS-EAGL-LS---QLLISRVLDQQTVSHWDST--SQRETRTRHWPSG--AAGLEDIDYELEETENRERLQGILRKRSNMGK--------
------------------------------------------------------------------------------------------------------------------------------
```

Alpha 2 domain

```
                            100           *         120          *         140       *            160       *         180
                           A
                           B                  C C
                          C C                 D E                                  E          DD  A
                        F E D                 E F F       FF                  F  FF     E     EE  DD  A   A   A
HLA-A2         ----GSHTVQRMYGCDVGSD--WRF--LRGYHQYAYDG---KDYIALKEDLRSWTAADMAAQTTKHKWEAA-HVAE--QLRAYLEGTCVEWLRRYLENGKETLQRT-
HLA-B27        ----GSHTLQNMYGCDVGPD--GRL--LRGYHQHAYDG---KDYIALNEDLSSWTAADTAAQITQRKWEAA-RVAE--QLRAYLEGECVEWLRRYLENGKETLQRA-
HLA-G          ----SSHTLQWMIGCDLGSD--GRL--LRGYEQYAYDG---KDYLALNEDLRSWTAADTAAQISKRKCEAA-NVAE--QRRAYLEGTCVEWLHRYLENGKEMLQRA-
Elephant shark ----GIHTVQQMYGCELKGD--GS---VGGFIQYGYDG---NDFIAFDKDRKVWTAPTAAAVVTKNKWENTPGLSE--QEKDYLEQICIEWLRKYVEYGHESL-RP-
Spiny dogfish  ----GIHTLQVMYGCELRDD--GS---TAGFFQYGWDG---KDLISFDKEHLVWNTPVTWQVVTKNKWEQDRGLGQ--QRKGYLEQECIEWLKKYLTAGEREL-KP-
sasaUBA (U)    ----GVHVNQKMYGCEWDDE-TGV---TEGFDQDGYDG---EDFLAFDLKTLTWIAPTPQAVITKLKWDSNTAQNE--YRKNYLTQTCIEWLKKYLDYGKSTLMRT-
DR14 (UBA)     ----GVHTFQFMYGCEMDDD--GN---KQVHWQIGYDG---EDFISLDKKTLTWTAANSQAMTTKVKWDSTGAEAN--YWKGYLENECIEWVQKYVGYGKDTLERK-
OL10 (UAA)     ----GLHVYQNMYGCEWDEE-TGE---VNSFRQFGYDG---EDFIALDVKTESYTAAKQQAEITKHKWENDKTGMS--YWKNYLTQICPEWLKKYVNYGSSSLMRK-
OL11 (UBA)     ----GAHVVQNMYGCEWDDE-TGE---VKGYDQYGYDG---EDFIALDLKSESWIAAKQQAVITKDEWDDNKAFTV--GRKNYLTQICPEWLEKYVNYGSSSLMRK-
ON39 (U)       ----GVHIVQRMYGCEWDDD-TGE---VNGYRQDGYDG---EDFISFDLKTETWVAAKQQAVITKLKWDSNKATIT--QYKNYLTQICPEWLKKYVNYGRSSLMKT-
GA9 (U)        ----GVHIVQNMYGCEWDDE-TNE---VKGYDQYGYNG---EDFISFDLQTERYIAAKQQASIIKQKWNQNRALIA--GKKNFLTHECPEGLKKFLSYGRSSLMRT-
TR13 (U)       ----GGDKHQHMYGCEWDDE-TRE---KNGFFQDGYDG---ADFISLKLKEGIWVAAKREAEISKHKWDQDEALIE--QLMDYYTQICIKWLQKFVNYGKSSLMRT-
ON3 (U)        ----GVHVIQRISGCEWDEN-TDS---VTGVLKYGYNG---EGFLEFDLKTLTWIALKPEADMIKQKWDADRTRTM--ENEDFLTQTCAEWLKMYVDNGKRSLHRT-
LO5 (U)        ----GVHTSQSMYGCEWDDE-DGT---TRGFQQEGYDG---EDYLVFDLKTLTWVAPTQRAFLTKQNWDADRAFNE--GKKNYLTQICIEWLKKYVNYGRETLNRR-
Paddlefish01(U)----GVHTVQRMVGCELDDD--GT---KRGFEQHGYDG---EDYIMFDKDTLTWTAASQRGFTTKVKWDPLTASNQ--QRKAYLEGTCIEWLKKYVQYGRETLERR-
Paddlefish03(U)----GVHTVQRMYGCELDDD--GT---KRGFDQFGFDG---KDFIIFDKDSLSWTAPVMQAVITKNKLDADRALNQ--QQKAYLEQICIEWLQKYVQYGKETLERR-
Sturgeon (U)   ----GVHTAQTMLGCELDED--GT---KRGFWQEGYDG---EDYIIFDKDTLTWTAANQRGFTTKVKWDPNTARNQ--YLKGYLEGTCIEWLQKYVQYGRETLERR-
sasaZAAa (Z)   ----DVHVLQWKVGCEIDQQSDGTLKFIKGIDQYSYDG---DDFLAFDDVTMQWVAPVDQALPTKRKLDDVQILNT--YTKGYLEKECVDWLSKFMEYEDKEFSWAD
AM8 (Z1)       ----DLHVLQWRHGCEIDES-NGEVKFLRGISEYGYDG---SDFLSFDRQTMTWIAQVPAAVITKMKWDNVAILTQ--YTKGYLEKECVDWLTKFLKFRKES-QQKD
AM3 (Z2)       ----VGHVLQWNLSCEGER-----HAPVFSSNEFGFDG---ENLIYFNCTSKTWLTPENKKNPEMEQLWNQQ--YR--QLAVEKCVQCEEMLKMYLNYNTTDLTPSH
AM25 (Z2)      ----DGHVLQWRHRCAGERQSDGSVTVSRSINEFAYDE---EELVNYNCTSNTWYNSD-NQKKEEIEKLSAVL--------MKKCSQCEEMLKIFLQHSTFNITPSQ
DR1 (ZEA)      ----DVHVLQWRHGCEIDSQ-GNDVRFSKGIDEYSYDG---RNFLAFDDADSQWVAPVEEALPTKRKWDNVPILNQ--YTKGYLEKECVDWLNKFREYGDQELREG-
OL4 (Z)        ----DTHVLQWMHGCEGVEDEHGNLQFKRGMDMYNYDG---DDFLAFDDERQVWVAAADAAVPTKRKWDEVTALKD--YTKGYLEKECMEWMKTFLSYSTQQLRNA-
GA18 (Z)       ----GQHVLQWQHGCEGEMQLDGTLKFSTGVDMYSYDG---YDFLSFDDSNSAWVAGAPAAQQTKRTWDGVDVLKE--YTKGYLEKECMEWMEKFLKYQGSVLENA-
ON22 (Z)       ----SPHVLQWMVGCEGEMQPNGALRFVRGMSKYNYDG---NDFLSFDDKNRVWVAPVKEALPSKTKWDNDQVLKE--YTKGYLENECIDWLSKSVTSEQKQLKKA-
TN13 (Z)       ----DIHVLQWVHGCNIDTSGNET-EFLHGIDMYSYDG---ESFLTFDEANENWVAPNDAALQTKRKWDDLQVLKE--YTKVYLKKECVTWLERFLKYQKENTAP--
TR21 (Z)       ----DIHILQWMHGCDADIDHGEP-RFLHGIDKYSYDG---ANFLAFNENQEIWDARDDARETKTRWDKVQVLRE--YTMAYLQKECVTWLKRFLDYQNKNEIP--
Eel (Z)        ----DVHILQWMHGCQAEKTPDGKLKFHNGFDQYGYDG---KDFLNFDSENMAWVAPVPAAESTKTKWDNVPILSQ--YTKGYLQTDCVHWLEKFLKYQELDFQDS-
Gar LO14       -----LHILQWMHGCEIEVQPGSKPQFLRGYDQYSYDG---RDFLSFDESKMQWVAPVWPAEPTKHKWDNEQILNQ--YTKGYLERECVDWLTKFLAFGEQQIKKS-
Sturgeon (Z)   -------------CRLDED--NNEE-VQGYDQHSYDG---NDFLSFDKNHLQWVAPMQPAVATKLKWDGEKILNQ--YTKGYLENECINWLKMFLGYGERDIYRS-
Lungfish (Z)   ----GYHTLQWVHGCSL-TD--GI---KIGIDQYAYDG---EDFLSFDKEKLSWIAVNKAAQQTREKWDEEKNLNQ--YTKRYLEQECIEWLQNFLRFSNGKLEKK-
sasaSAA (S)    ----HDHFYQAHGRCGWKSD--GT---TEAFMSHAYDG---KDFVSFDVSTRTWTAAVSHAVFYKRKRETDLEDLV--RLVIHYESGCIRWLKKLLQ-FSVTFREP-
AM33 (S)       ASSSDVNIYQGYGRCSVYPN--GT---LKALLTHAFNG---KDFLTFDVDRKSYIASVPQAVIYKRQREANPVWLE--IMASFYKKTCFERLKMFLQHASVHITKK-
AM39 (S)       GSLTDGNIYQAFGCCSLYPN--GT---YRTLLAHAFNG---KDFASFDFQTKTFVAAVPQAVLYKTLREKDPANME--EIATLYKKTCLERLQ-ILKEAPRVNIKK-
Ayu (S)        ----HDHVYQAQGHCGWNTD--GT---RVAYMSHAYDG---KDFISFDVQNKQWTAVVPEAHFYKTSRQQHSEDLD--RITNHYESECILWLKKLLQFSSKLLKPK-
Catfish (S)    GSHADINVYQAYSRCDLYPD--GT---TKSSLTHAFNG---KDFLSLDMDSKTYIASVPQALIYKSIRDKDIIWLE--IVVSYYKKRCFDHLRMFLEHAPGVRNKK-
sasaLCA (L)    ----GVQVQQRMAGCEMFDN--GEP---ALIMTKNTFNAVFADHAIYYNITHFTYDAGKLLQG----WDGMRQAQEKILYENVLLTLCIRTLKTILKREKNIVMRK-
sasaLGA (L)    ----GVQVQQKITGCEMLNN--GES---ALVMYKDVFNAIYTDRTLYYNMTHFTYDAGKLLLG----WDGIRQAYERTLYENVYLPICIKSLKRLLKREKNIVMRK-
DR12 (LAA)     ----GVHVHQRLVGCELLNN--EKT---GPLHYWDAFGGQNMEEFI-FDTEKHTIQVKMPWVIT---WDQLKRLHENFMYDNVYHPICIKTLRRYLNMEKKNVMRK-
ON9 (L)        ----GVHVQQRVTGCEVLED--GQP---ALIMFRDGSNGQDADSLL-YNMTHFTYAVREGWEIQ---WDALKKTSFQMLYSNIYLPFCVRTLQHFLEREKHLVMRR-
LO11 (L)       ----GLHTYQRQAGCELSDD--GT---QLFHARDAYDG---QDLLQYNMDHLRWDPLVPELKN-----DEALMHSDDIEQNNTYQPLCIQVLKSYLEQEKDRPVMR-
AM5 (P)        ------GIIQREISCTEDDSVVRM------SDRWGVNG---EDFLTLDPKTLKWSSDSPLAAPVQSDWNQMKFMTP--SLKDFKLNQCKPSLMKLKMKKEEYLQENM
TN5 (P)        ------VDVQRRRGCTRSAEGLNS-----AFEIWAVNG---EDFIQFDADHQEWEALADTALTVRDSWNNLETRNQ--VFGSFLKDQCPEMIQRINLTEVE--QRT-
TR9 (P)        ------RNVQRRRGCITSDERLES-----AFDNWAVNG---EGFIQFDAGAQKWKALSPSAEMIKDSWNSRESRND--FFGQFINEECPEMIHQIKLREAE--KRT-
TR14 (P)       ------RNVQRRRGCITSDERLES-----AFDNWAVNG---EGFIQFDAGAQKWKALSPSAEMIKDSWNGYEVQNY--LFGQFIKEQCPEMIHQIKLREVE--KRT-
CodPAA (P)     -----VDLIQRRRGCSRSENGALS-----AFETWAVNG---ADFVTFDPKTLQWKAMSQAAREIASRWNTRKTRNL--VFRDFVNIHCPKMIKSLELKYVD--QKT-
Seabass (P)    -----VYVIQRRCGCVRSADGIVS-----AFDAWAVNG---MDFISFDPESQRWTSQSPLAIPVRHWWKNNNGRNF--AFKHFLRERCPLLIQGMKLRSTH--QNT-
LO4 (P)        ----VLDVLQRCRGCRLEDD--GS---VALFDHYGFNG---KYFLSFEVDNMIWRASSLQAKDTQRDWNQNRVKNQ--YTRFFLEIDCMETLKRFLEFREIDKNHT-
```

Alpha 3 domain

```
                                *         200         *         220         *         240         *         260         *
                                                                      TTT TTTTT
HLA-A2           -DAPKTHMTHHAVS--DHEATLRCWALSFYPAEITLTWQRDG-EDQTQD-TELVETRPAGDGTFQKWAAVVVP--SGQEQ---RYTCHVQHEGLPKPLTLRW
HLA-B27          -DPPKTHVTHHPIS--DHEATLRCWALGFYPAEITLTWQRDG-EDQTQD-TELVETRPAGDRTFQKWAAVVVP--SGEEQ---RYTCHVQHEGLPKPLTLRW
HLA-G            -DPPKTHVTHHPVF--DYEATLRCWALGFYPAEIILTWQRDG-EDQTQD-VELVETRPAGDGTFQKWAAVVVP--SGEEQ---RYTCHVQHEGLPEPLMLRW
Elephant shark   -VRPEVTLSSPRGS------RLSCLAAGFYPRAIEVKLLKNG-QILSDE-E-SPGTSPNQDGTFQLRKSVEIN--PTATE---TYSCQVEHSSLTTPFTVVY
Spiny dogfish    -VAPRVFPSVNKAS-NIRPTELSCLVTGFYPRDIEVTLLRNG-QPIT-D-TESTGILPNHDGTYQLTRWAQIT--LDEGA---TYSCQYDQGDKVGVEIRHW
--------------------------------------------------------------------------------------------------------------------------
sasaUBA (U)      -VPPSVSLLQKTPS-----SPVTCHATGFYPSGVMVSWQKDG-QDHHED-VEHGETLQNDDGTFQKSSHLTVT---PEEWKNNKYQCVVQVTGLQEDFIKVL
DR14 (UBA)       -VSPQVSLLQKSSS-----SPVVCHVTGFYPSGLKISWQRNG-QDHDED-VELGELIPNEDGTYQRTSTLNVK---PEEWKKDKFSCVVEHQSKTINSILTE
OL10 (UAA)       -VLPSVSLLQKSSS-----SAVSCHATGFYPDRAELLWRRDG-EEIHEG-VEKGQILPNNDGTFQMSVDLQPP--SGEDM--QRYECVFQLSGVKEDVITKL
OL11_(UBA)       -VPPSVSLLQKSSS-----SAVSCHATGFYPDRAELLWRRDG-EEIHEG-VEKGQILPNNDGTFQMSVDLQPP--SGEDM--QRYECVFQLSGVKEDVITKL
--------------------------------------------------------------------------------------------------------------------------
TN1  (U)         -VQPSVFLLQKTPS-----SPVSCHVTGFHPENGVVFWRKDG-QKLQEE-VEYTEILPNHDGTFQTRVDLELSSVSPEEW--SRYECVFHPPGDQEDITLKL
ON39 (U)         -VLPSVSLLQKSSS-----SSVTCHATGFYPNRAEMVWKKDG-VEHHEG-VNKGEILTNNDGTFQMSVDLDVSSVKPEDW--HRYRCVFQLSGVNEDIVTRL
GA9  (U)         -ERPSVSLLQKTPS-----SPVSCHATGFYPHRAALFWRKDG-EELHED-VDLGEILPNHDGTFQMRVDLKLSSVPAEDW--RRYDCVFQLSGVDEDIVTKL
TN23 (U)         -ELPSVSLLQKEPS-----SPVTCMATGFYPGGATLSWRRGE-EELHEE-VEPGEILPNPDGTFQMSAALDLSSVPPEDW--SSYKCVFQLSGGQEVPTSLD
TR13 (U)         -ELPSVSLLQKTPS-----SPVRCHATGFYPDTAVMSWRKGE-EELHED-VDHGEMLLNPDGTFQASVDLNVSSVPPEDW--SSYKCVFQLSGGKEINTTLD
ON3  (U)         -VPPSLSLLQKTPS-----SPVSCHATGFHPDRVMMFWRKDG-DEIH-DGVEKGDILPNNDGTFQINVYLNVSSVKPEDW--RKYECVFQLLSGENVIVTKL
LO5  (U)         -ERPQVSVFHKD-S-GSGSTELTCLATGFFPRDILVSWWRDG-QELHED-VDSGEVVPNGDGSFQVRKRLRVR-----AGEEHKYSCRVDHTSLEKTIVQHW
Paddlefish01(U)  -VPPEVTLLQRKAR-GSADMEVLCHVTGFFPRAVEVTWVRDG-QDQLEEGVQNGEVLLNQDGTYQLRKILTVS---PEEQGRHRYSCQVDHISFKEKQIYIW
Paddlefish03(U)  -VPPAVTLRHKKAR-GSADTEVVCHVTGFYPRAVEVTWVRDG-QVQLEDRVQSGEVLPNQDGTYQLRKILTVS---PEEQGRHSYSCQVDHVSFTERQNYIW
Sturgeon (U)     -VPPAVTLLQRKAR-GSADTEVLCHVTGFFPRAVEVTWVRDG-RDQLEEGVQSGEVLPNQDGTYQLRKILTLS---PEEQGRHSYSCQVDHISLDQKIVKEW
--------------------------------------------------------------------------------------------------------------------------
sasaZAAa (Z)     -SAPKVYAFAKKAK-TAGHVRLTCMATGFYPKDVVMHIKKNGVPLTDRDGVQSAGLLPNDDETYQIRMSVQIP-----EADKETYECYVNHRALKEPIVVKW
AM8  (Z1)        -APLDVQVFAKPSVSDSSKLTLTCLATGFYPKDATVIWRRSSSPLSEDL-ITSSAVRPNDDGTYQLRKSVEIL-----GAEKDQYECYVSHRTLKEPVIKKL
AM3  (Z2)        -SPTVYIFQN-KSVSDSSELILTCMATGFYPKDVKMSLMKSGTEIPDHL-ITSSGVRPNHNGTYQLRKSVEIQ-----EDEKDQYECYVSHSSLTEPKIEKW
AM25 (Z2)        SAPPAVHVFSKKSVHDSSKQILTCLATGFYPKDVKMSLRKSGTSLPEHL-ITSSAVRPNDDETYQLRKSVEIQ-----EDDPADYDCYVNHSSLQTPVIKQW
DR1  (ZEA)       -SPPDVHVFAKKIISGKAKLKLTCMVTGFYPKDVILTIRKYRTALSDNE-VESSGVRPNPDGTFQLRKSTNIY-----E--KAEYDCYVAHRTLKEPIIKKW
OL4  (Z)         -SRPDVYMFFKKAK-ESSNVVLTCLATGFYPKDITLNIRRDGRVLTKDDGVMSSGVRPNHDETFQRKDYVEIL-----RSDSATYTCEIIHPASNVWVVKTW
GA18 (Z)         -TKPEVYLFASKAK-KEANVILTCMATGFYPKEIQLWIKRNGRVLRREDGVMSSGSRPNGDETFQRKDWVEIL-----KTDQSQYTCEVIHKATQVNIEKEW
ON22 (Z)         -PPPGVYVFAKKSR-VETNLTLTCLATGFYSKNIILRIRRKGRVLTEDDGLWSSGVLPNDDETFQRRDYVEIL-----KSDLSEFSCEVVHEATRVDVVKTW
TR21 (Z)         -AKPDLYVFITGAK-DPTNMVLKCMATGFTPTNTVLQIKLGGRVLTREDGVNSTDILPNGDGTFQKTDYVEIP-----RSDNSDYYCELSHTPSSLRVVKFW
TN13 (Z)         -VKPAVYALSREAN-VQANIVLTCMATGFSSINTIVQIKRDGLVLTKDD----GDVRPNGDGTYQKRDQVEIP-----KSDKSKYTCEVIHESSGLHEIRVW
Eel  (Z)         -SPPDVYIFSKKST-SPDYRTLVCLATGFYPKDVAVDILKDGAPLAESDGVESSGVRTNGEPKETFQLRKWLE---IKTSDTSKYSCRVKHQTLRSPIVKFW
Lungfish (Z)     -VGPETKIFRKRRH-D-GKYTFSCHASEFFPSALSIKWINIKTNEKKEE---TAEALPNNDGTFQAWVDIDSD-----LECARDLVCMITHGNSSEE-HRIP
--------------------------------------------------------------------------------------------------------------------------
sasaSAA  (S)     -V-PAVSLFERPPH-GNSEVEVTCHVTGFYPRAVQVEWLGAEGLPMV-DGVSSGEVLPNGDGSYQLRKSLTVP---QEAQDTQSYSCLVLHSSVAGNITLTW
AM33 (S)         -V-PEVHLFKSF-K--SGSSVLACHVTGFYPKEVQVEWIGA-GLQPV-DG-EVIEVLPNGDGTYQTRRSVIRP---EENPEKHSYSCVVQHSSIAGNITKTW
AM39 (S)         -V-PEVRIIEKK-R--TGSVTVTCHVTGFYPREVQVIWLGS-DLQPVVKG--VTEALPNDDGTYQTRKSVIVP---EEDVGKHTYSCVVLHISVPNNITTVW
Ayu  (S)         -A-PDVSLFERP-S--SSEVLVTCHVTGFYPKEVQVEWLGE-EGHPLVQEVRRGEVLPNEDGTYQLRTILAVP---LGSQHSLSYSCLVVHSSVQGNITKIW
Catfish (S)      -V-PEVRLFERQ-G--AGSTLLTCHVTGFYPRAVQVKWIGA-DLQLVEDE--MNHVLPNGDGTFQTRSSVIRP---EENTGDQRYSCVVHHSSLEGNITVTW
--------------------------------------------------------------------------------------------------------------------------
sasaLCA (L)      -VPPRLR-LIKK-EVS-GGFQVSCLVFGFYPRHINLTLLRDGQPVAEQE-LTGGEVLPSGDGTYQLRKSLEVS--TEELKKRHNYTCTASHLSLDNKLDVSW
sasaLGA (L)      -VPPRLR-LIKK-EVS-GGFQVSCLAFGFYPRHINLTLLRDGQPVAEQE-LTGGEVLPSGDGTYQLRKSLEVS--TEELKKRHNYTCTASHLSLDNKLDVSW
DR12 (LAA)       -VKPRVRLMQKKLSDS-QGLQISCLATGFYPRHINLTLFRDAELVDDDQ-ITGGEILPNGDGTYQMRKSLIVS--EEELQKGHKYNCTANYLNLDNKMDIVF
ON9  (L)         -VKPRLRFITRQ-VV--GGAQVTCLATDFYPRHINLSLLRDGQPVDEGE-VRVGSVLPNGNGLYQVRKTLMVG--EKELQRKHNYTCEAFHLSLDNRLRINW
LO11 (L)         -VKPRVQVFQKTSALS-GGTEVTCLATGFYPRALELTLLRDRRPVPEQE-LTGGEVLPNGDGTYQLRKSLALSKEEEERRERHRYTCRVQHSGLDNMLEVAR
--------------------------------------------------------------------------------------------------------------------------
AM5  (P)         -YPKMYIFGKSSHD-G-DAVSMRCYISHKYLSGVRVRLTLDG---VAVDNVNISSPAPNMDGSVQIRLETKTNI----KEPNR-YHCVVDTD--YLHIFTAW
TN5  (P)         ----DLRVFATPID-R-FQTLLKCHVTSTDKSLGSLSLTEDG--ASRALWVAVRGPLPSVDGSVVLRLTAEVP-----LGYSNIYGCVVPTG--GGTITVMW
TR9  (P)         ----DLRVFANPVD-R-TKALLKCHVTSTDKSVRSVSLTEDG--APKANWITVTGPLPSGDGSVILILTAEVP-----LIHTNIYGCVVQTE--DRNITVMW
TR14 (P)         ----DLRVFANPVD-L-TRALLKCHVTSTDKSVRSVSLTEDG--ATKANWITVTGPLPSGDGSVILILTAEVP-----LIHTNIYGCVVQTE--DRDITVMW
CodPAA (P)       ----DLHISAQVLP-NESKFKLRCQVTSTDRSVKLLTLIGEG--ASYARQVTVDGPLPFGDDYVILRLTAVIP-----SSEHQ-YGCRVQTE--KHSFSAFW
Seabass (P)      ----ELHIFAKPIA-DTDQALLRCHVTSTDKSVSSVHLIGDG--AFKANWISVTGPMPSEDGSVILRLTAEISL----SQSTNMYGCRVQTG--GHNITIFW
--------------------------------------------------------------------------------------------------------------------------
```

Connecting peptide, transmembrane and cytoplasmic domain

```
                  280                    *          300       *         320       *         340
             CP                    TM                        CYT
HLA-A2     : EPSSQPTIP-------------IVGIIAGLVLFGAV-ITGAVVAAVMWRRKSSDRKGGSYSQAASSDSAQGSDVSLTACKV----------
HLA-B27    : EPSSQSTVP-------------IVGIVAGLAVLAVV-VIGAVVAAVMCRRKSSGGKGGSYSQAACSDSAQGSDVSLTA-------------
HLA-Cw6    : EPSSQPTIP-------------IVGIVAGLAVLAVLAVLGAVMAVVMCRRKSSGGKGGSCSQAASSNSAQGSDESLIACKA----------
HLA-G      : KQSSLPTIP-------------IMGIVAGLVVLAAV-VTGAAVAAVLWRKKSSD-------------------------------------
sasaUBA    : TESEIKTN--WNDPN--------IVLIIGVVVALLLVVVAVVVGVVIWKKKSKK--GFVPASTSDTDSDNSGRAAQMT-------------
sasaUGA    : TESEIQTNRGVNTIGSAP--------IIGVVVALLVVVV-VVVGLVMWRRKKSK-KGFVPASTSDTDSENSGKGAQKI-------------
DR14 (UBA) : TEDEIRTN--NPTAP--------LGIIIGIVVAAVLLVAIAVAGFVVYRRHKGF-KP-VPQNTSDGGSDNSSRT-----------------
GA9 (U)    : DKTRTNREKPADST----------FIIIIIIIAVAVLVV-IIAAVVGFKVHRKRNAQRSSAKPSSSSSASTDGSEVTEELNPKP-------
ON5 (U)    : NWAEKPAD---------------MVTFISAAVVLLTVTIITAVAFVAYKKKKGE-KSECAPD---------DGSEQSERLNPQS-------
OL10 (UAA) : EKAKIRTNEDSSSQ----------MLIIIGVLAAVIAVILSVSAFILYKKKN-----AKRPPSPVDNKEIQEQMLQPENPSA---------
OL11 (UBA) : EKAKIRTNEGSSSQ----------MLIIIGVLVAVIAVVLSVSAFIFYKKMNFF-NLVKPPPSPVDNKEIQQQMLQPENPSA---------
Sturgeon(U): DPNMRSSSDVPESSSP--------IGLIAGVIVGVLAVAAVIIGVVIWKKRQG-GAQKNYTPAQTNEGSDTSSDTAP--------------
sasaSAA    : APKKN---------------------LANVLMAIVIIVSVVLILTVLFKYLVRRRAVGKSQS-----------------------------
AM33 (S)   : VAEEHS---------------------LLAVWISLVCILVIIGTGLVLRKFCRCGQRDTGI------------------------------
Catfish (S): GKEEKPFR--------------------LYVWITLGCVFIVTVVGLVIR--CILKSKDAGI------------------------------
sasaZDAa   : DGICCDCSSFN--------------AVVIGAVVITFIVVLILVVLFVLHRRGTIVIPGL------RTTATGNGVAFSGVNTS---------
GA18 (Z)   : DHKLPENGSP-------------IGVAVGVPVLVLVLAVVAGVLIFCYRKASSTSSTSSTSSTSSTSSTSSSNTDSIS-------------
OL7 (Z)    : DHQHPDNDAT------------GIIIGAAAGVVVLIAVGIVAVVIVMLKKKRKPSPSSSTSSMSSPENTELLKKNSNGRLCPFNNLTVNFH
DR3 (ZCA)  : NGEYLSEPP---------------IAIIAAIIGVLILLVAIGVTVWILKKKNIIGNKDEKRSMPNGSANYGRGSSA---------------
DR5 (ZFA)  : DGKCKDCLPNLNW--------------IWVVAGAVLMLGVVALLLVLLKKKIIDLRQRLSGSQSPLYQIQADSESEDYNH-----------
sasaLCA    : ESGAERVH-----------------LSTLSVLLVMLLILILLVTFICVKRRWSNTASQS--ELANVDAKVSEEMNLSSDSEN---------
sasaLDA    : ESGAERVH----------------LFILSAPLVMALIVILFCIFICLVRRIR-A-ASQNLLQLASVDALEADEMNLSSDSEKT--------
sasaLGA    : ESGAERVH-----------------LSTLSVLLMMLLILILLGIFICVKRRWSNTASQS--ELANVDAKVSEEINLSSDSET---------
DR12 (LAA) : DVAESDPGS--------------FSVSVVMGVLVFVGLSVLSITALIMRRKRRDTGSVSGTSQNQYVYAQTSVQDAT--------------
DR20 (LPA) : DLAEPHRNT-----------------IWIAVSVLLVCAIIVGLAMLIWKRYQTARQREN--EPNNHQTTHMN-------------------
TN3 (P)    : DGNTLDGRDIIYISDNW---ISLYILGSLAFLGALLVCVI----------------?----------------------------------
TR14 (P)   : DGNTLDGREILYATVDWPFWK-ILTIV-TFSCVFLLICTTAVVL-----------?----------------------------------
TR29 (P)   : DGKTLDGREILYATVVWPLWK-ILTIV-TFSCVCLLICTTALVL-----------?----------------------------------
Seabass (P): DGNTLDGR-NLLNMLTVH-WK-ILTAILGFVCIIFVITAISCGTIFLLKCVKKKS?----------------------------------
```

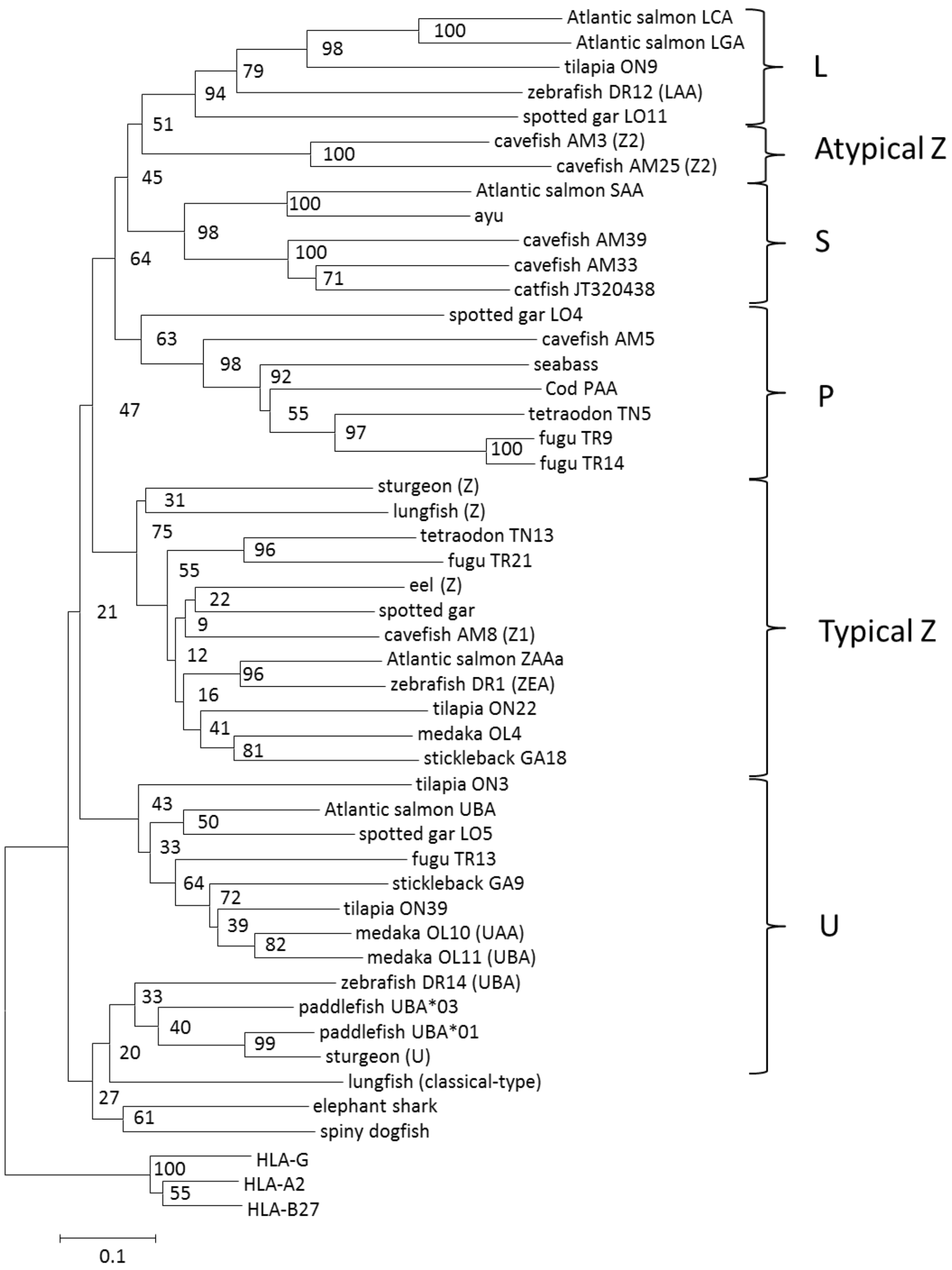

Text S8b. Phylogenetic tree of deduced alpha 2 domain amino acid sequences.

The tree is based on the hand-made alignment in S8a and rooted using the human HLA sequences. The evolutionary history was inferred using the Neighbor-Joining method [main text reference 95]. The percentages of replicate trees in which the associated taxa clustered together in the bootstrap test (1000 replicates) are shown next to the branches [96]. The tree is drawn to scale, with branch lengths in the same units as those of the evolutionary distances used to infer the phylogenetic tree. The evolutionary distances were computed using the p-distance method [97] and are in the units of the number of amino acid differences per site. All ambiguous positions were removed for each sequence pair. Evolutionary analyses were conducted in MEGA5 [98]. Sequence references can be found either in legend to S8a or in additional files 3: Text S1 and 4: Text S2. Atlantic salmon is *Salmo salar*, cavefish is *Astyanax mexicanus*, zebrafish is *Danio rerio*, tilapia is *Oreochromis niloticus*, stickleback is *Gasterosteus aculeatus*, tetraodon is *Tetraodon nigroviridis*, fugu is *Takifugu rubripes*, medaka is *Oryzias latipes*, spotted gar is *Lepisosteus oculatus*, ayu is *Plecoglossus altivelis*, catfish is *Ictalurus punctatus*, seabass is *Dicentrarchus labrax*, eel is *Anguilla japonica*, paddlefish is *Polyodon spatula*, sturgeon is *Acipenser sinensis*, lungfish is *Protopterus aethiopicus* and *Protopterus annectans*, the shark spiny dogfish is *Squalus acanthias* and the chimaera elephant shark is *Callorhinchus milii*.

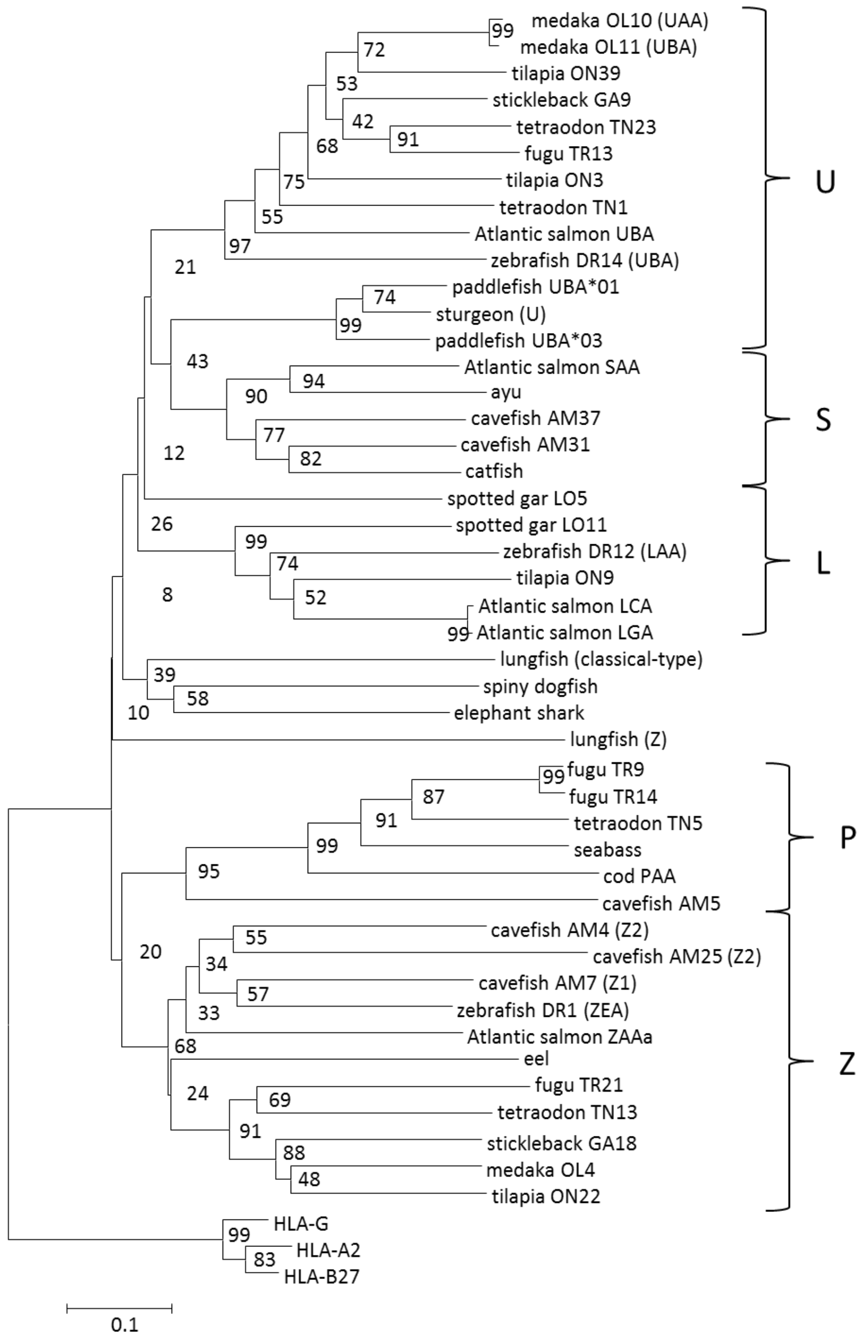

Text S8c. Phylogenetic tree of deduced alpha 3 domain amino acid sequences.

The tree is based on the hand-made alignment in Text S8a and rooted using the human HLA sequences. See legend to S8b for details. Bootstrap values in percentage from 1000 trials are shown on each node. Atlantic salmon is *Salmo salar*, cavefish is *Astyanax mexicanus*, zebrafish is *Danio rerio*, tilapia is *Oreochromis niloticus*, stickleback is *Gasterosteus aculeatus*, tetraodon is *Tetraodon nigroviridis*, fugu is *Takifugu rubripes*, medaka is *Oryzias latipes*, spotted gar is *Lepisosteus oculatus*, ayu is *Plecoglossus altivelis*, catfish is *Ictalurus punctatus*, seabass is *Dicentrarchus labrax*, eel is *Anguilla japonica*, paddlefish is *Polyodon spatula*, sturgeon is *Acipenser sinensis*, lungfish is *Protopterus aethiopicus* and *Protopterus annectans*, the shark spiny dogfish is *Squalus acanthias* and the chimaera elephant shark is *Callorhinchus milii*.

Text S8d. Phylogenetic distribution of MHCI lineages

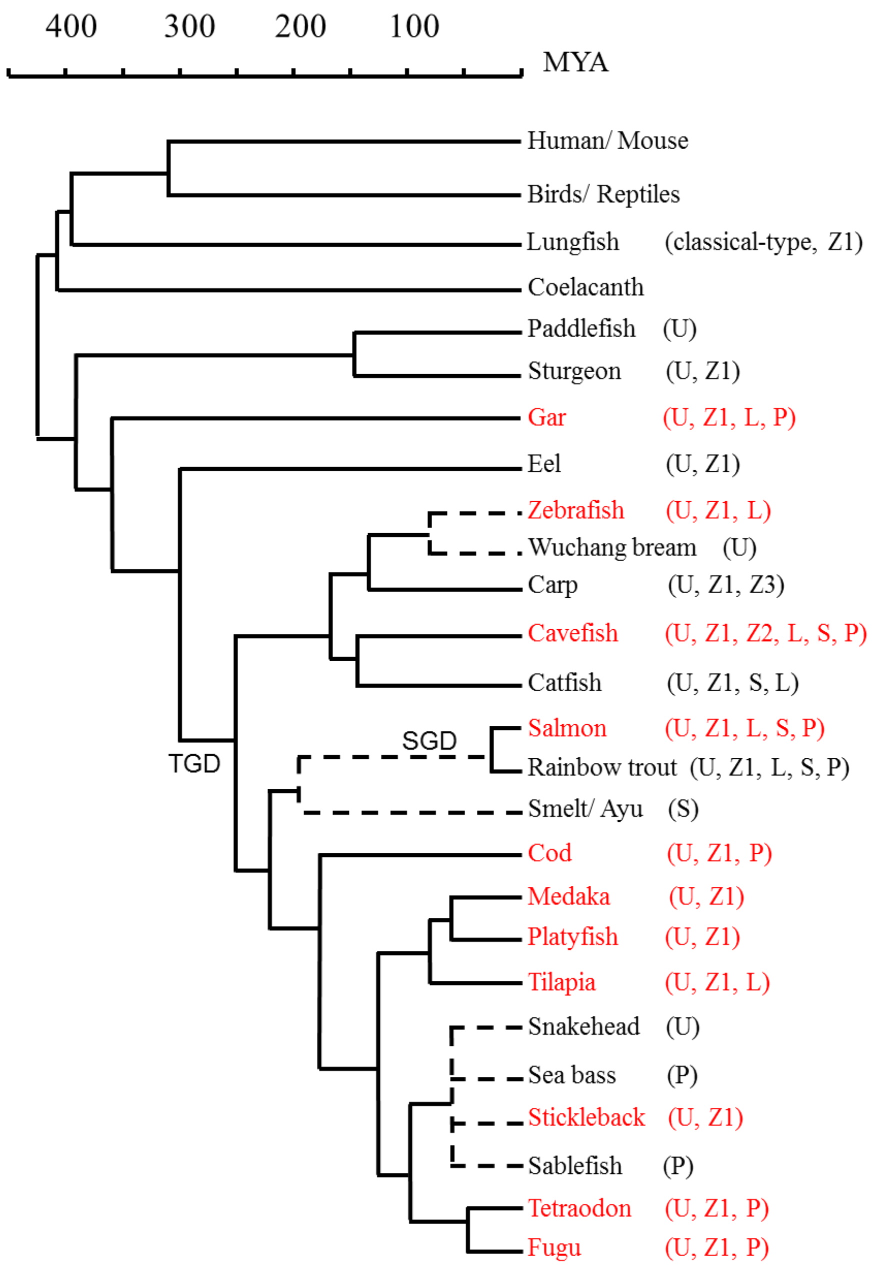

Text SF8d. Phylogenetic distribution of MHCI lineages

Species with sequenced genomes where we have analysed presence of all MHCI lineages in available genomes and transcriptomes are shown with red font. Species with limited genome and/or transcriptome resources where search and analyses have been restricted to certain lineages are shown with black font.

Presence of MHCI lineages are shown in parenthesis after the species name. Sequences can be found in additional files 3: Text S1 and 4: Text S2. Dotted lines relate to phylogenetic branch knots where the referenced literature was not informative on the absolute time of the event. Phylogeny references can be found in legend to Figure 1 in main text. In primitive bony fish our use of "U lineage" terminology does not refer to an actual pure lineage that is fully separate from some of the nonclassical lineages.
